# Analysis of hepatitis B virus integration identifies *KMT2B* as a novel cancer‐related gene in pancreatic cancer

**DOI:** 10.1002/ctm2.70424

**Published:** 2025-07-31

**Authors:** Mengge Li, Huimin Li, Dejun Liu, Shunan Liu, Hui Yuan, Yan Wu, Min Du, Yuan Fang, Jin Li, Hui Cong, Dan Zhao, Chunsun Fan, Qing Wang, Cenkai Shen, Yu Gan, Yongwei Sun, Hong Tu

**Affiliations:** ^1^ State Key Laboratory of Systems Medicine for Cancer Shanghai Cancer Institute Renji Hospital Shanghai Jiao Tong University School of Medicine Shanghai China; ^2^ Department of Medical Oncology The First Affiliated Hospital of USTC Division of Life Sciences and Medicine University of Science and Technology of China Hefei China; ^3^ Department of Biliary‐Pancreatic Surgery Renji Hospital Shanghai Jiao Tong University School of Medicine Shanghai China; ^4^ Organ Transplantation Center The First Affiliated Hospital of Kunming Medical University Kunming China; ^5^ Shanghai Medical College Fudan University Shanghai China

**Keywords:** FYN, hepatitis B virus, integration, KMT2B, pancreatic ductal adenocarcinoma

## Abstract

**Background:**

Pancreatic ductal adenocarcinoma (PDAC) is a highly aggressive malignancy with few well‐established risk factors. Emerging epidemiological evidence suggests a link between hepatitis B virus (HBV) infection and PDAC. However, the underlying mechanisms remain unclear.

**Methods:**

High‐throughput sequencing‐based approach was employed to identify HBV integrations in tumour and para‐tumour tissues of PDAC. The biological functions of KMT2B were evaluated in PDAC cell lines as well as in subcutaneous and orthotopic mouse models of PDAC. Chromatin immunoprecipitation sequencing and RNA sequencing were used to identify the pathway involved in PDAC development.

**Results:**

HBV integration was detected in approximately one‐third of HBV DNA‐positive PDAC and adjacent para‐tumour tissues. A total of 425 viral‒host junctions were identified, with the majority located in intergenic regions (51.29%), followed by introns (43.29%) and exons (2.35%) of the human genome. Lysine methyltransferase 2B (*KMT2B*, also known as *MLL4*), a gene frequently targeted by HBV integration in hepatocellular carcinoma, was also found to be interrupted by HBV in PDAC. KMT2B was significantly upregulated in PDAC and promoted malignant behaviours both in vitro and in vivo. Mechanistically, KMT2B exerts its oncogenic effects by regulating the downstream target gene *FYN* through histone H3K4 trimethylation, leading to the activation of the PI3K/Akt signalling pathway.

**Conclusion:**

HBV integration is a common event in HBV‐related PDAC and *KMT2B* has been identified as a novel PDAC‐related gene.

**Key points:**

- Hepatitis B virus (HBV) integrates in both tumour and adjacent para‐tumour tissues of pancreatic ductal adenocarcinoma (PDAC).
- *KMT2B*, a target gene of HBV integration, promotes PDAC proliferation and metastasis in vivo and in vitro experiments.
- KMT2B exerts its oncogenic effects by regulating the downstream target gene *FYN* via histone H3K4 trimethylation, activating the PI3K/Akt signalling pathway.

## Graphical abstract

HBV DNA integrates in multiple genes including *KMT2B* in pancreatic cancer. KMT2B upregulation caused by HBV integration or other mechanisms promotes FYN expression via histone H3K4 trimethylation at its promoter region. Increased FYN expression activates the PI3K/Akt signalling pathway, thus driving PDAC cell proliferation, migration and invasion.

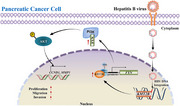

## 1 BACKGROUND

Pancreatic ductal adenocarcinoma (PDAC) is an aggressive and lethal malignancy with a poor prognosis. The aetiology of PDAC remains largely unknown, which hampers the development of effective prevention and treatment strategies for the disease. Established risk factors, including tobacco use, chronic pancreatitis, obesity and type 2 diabetes, collectively account for only half of all PDAC cases.^1^ This highlights the urgent need to identify other factors involved in PDAC development.

Emerging evidence indicates there is an association between hepatitis B virus (HBV) infection and PDAC.^2^, ^3^, ^4^, ^5^, ^6^, ^7^, ^8^ Meta‐analyses have revealed that the summary odds ratio (OR) of PDAC risk for HBV‐infected individuals is 1.24 (95% confidence interval [CI]: 1.07‒1.44) in case‒control studies and 1.57 (95% CI: 1.16‒2.13) in cohort studies.^9^ Notably, in HBeAg‐positive persons, the hazard ratio for PDAC reaches 5.73 (95% CI: 1.73‒19.05).^5^ Although the association between HBV and PDAC remains controversial in Europe and Oceania,^10^ it is worth noting that all studies from China, where HBV prevalence is as high as ∼6%, support HBV as a risk factor for PDAC.^11^ Moreover, HBV infection has been reported to influence the metastatic pattern and survival rates in Chinese patients with pancreatic cancer.^12^ Despite the findings from epidemiology studies, the association between HBV and PDAC is less well established compared its role in hepatocellular carcinoma (HCC).

HBV is a hepatotropic virus with the capacity to infect certain extrahepatic sites including the pancreas.^13^ HBV DNA, as well as the replicative covalently closed circular DNA (cccDNA),^4^, ^14^ has been detected in pancreatic cells using in situ hybridisation,^15^ Southern blot^14^ and PCR.^4^ Viral protein HBsAg and HBcAg have also been revealed in pancreatic acinar and islet cells by immunohistochemistry (IHC).^4^, ^15^, ^16^ These lines of evidence suggest a possible in situ influence of HBV on PDAC. Given that HBV DNA integration is one of the key mechanisms responsible for HCC, it is intriguing to explore whether HBV integration plays a role in PDAC development and progression.

Over the past decade, a large number of HBV integration sites have been isolated from HCC specimens using next‐generation sequencing‐based approaches.^17^, ^18^, ^19^ *TERT* ^20^, ^21^ and *MLL4* ^20^, ^22^ are the most commonly affected genes by HBV integration. Since one‐third of HBV preferential target genes are cancer related,^23^ insertional mutagenesis is considered a possible mechanism for HBV‐induced malignancy. Tracking the genes affected by HBV DNA integration has led to the identification of novel cellular genes and regulatory elements involved in HCC, such as *RARB*,^24^ *CCNA2*,^25^ *SERCA1* ^26^ and LINEs.^27^ Therefore, utilising HBV as a pro‐viral tag could serve as an astute strategy for identifying genes implicated in HBV‐related cancers.

To better understand the causal relationship between HBV infection and PDAC, we isolated HBV integration sites from PDAC tissues and adjacent non‐tumour tissues. *KMT2B*, also known as *MLL4*, which is a preferential target of HBV integration in HCC, was found to be disrupted by HBV DNA in a PDAC specimen. *KMT2B* is known for its role in histone H3K4 trimethylation (H3K4me3) and gene regulation. Although increased expression levels of *KMT2B* have been reported in various cancer types, including HCC, colorectal cancer and gastric cancer,^28^ its role in cancer development remains investigation. In this study, we explored the biological functions of *KMT2B* in PDAC. Our findings reveal a novel KMT2B/FYN/PI3K/Akt axis through which KMT2B promotes the malignant behaviour of pancreatic cancer.

## 2 METHODS

### 2.1 Patients and samples

Eighty‐six PDAC patients who underwent pancreatectomy at Renji Hospital, Shanghai Jiao Tong University School of Medicine (Shanghai, China), from January 2016 to December 2016 were recruited. The sample collection and preparation protocol were approved by the Renji Hospital Ethics Committee, and samples were obtained with informed consent. The research was conducted according to the principles of the Declaration of Helsinki. Fresh tissue samples were snap‐frozen in liquid nitrogen immediately upon surgical removal and maintained at ‒80°C until use.

### 2.2 Detection of HBV serum markers and HBV DNA

The presence of HBsAg, HBeAg and antibodies to HBsAg (anti‐HBs), HBeAg (anti‐HBe) and HBcAg (anti‐HBc) in serum was tested using the AXSYM HBV reagent pack (Abbott Laboratories). For detection of HBV DNA in pancreatic tissues, genomic DNA was extracted from 20 mg PDAC or para‐tumour tissues using a QIAamp DNA mini kit (Qiagen). The genomic DNA was amplified for the presence of HBV S and C genes by nested‐PCR with specific primers as described in our previous publication.^4^ PCR products were subjected to Sanger sequencing (BioSune) to confirm the correctness of amplification.

### 2.3 HBV capture sequencing and analysis

HIVID‐based HBV capture sequencing^18^, ^29^ was used to identify HBV integration according to the experimental procedure described previously.^29^ After removal of pair‐end reads, sequenced datasets were compared against both the HBV genome (NC_003977.1) and the human reference genome (hg19) using the Burrows‐Wheeler Aligner. Integration sites were determined through seeksv analysis.^30^ HBV capture sequencing data were uploaded to the Genome Sequence Archive for Human (GSA‐human, https://ngdc.cncb.ac.cn/gsa‐human) database with accession number of HRA005175.

### 2.4 Animal experiments

BALB/c nude mice were obtained from Sino‐British SIPPR/BK Lab Animal Ltd. To construct a subcutaneous tumour model of PDAC, PANC‐1‐KMT2B or PANC‐1‐VC cells (2 × 10^6^) were injected into the right flanks of 5‐week‐old male BALB/c nude mice. Tumour sizes were measured once a week starting on day 5 post‐inoculation. Tumour volume was calculated using the equation: volume = .5 × length × width^2^. The mice were sacrificed 53 days after tumour inoculation. The orthotopic PDAC models were established as described previously.^31^ Briefly, 3 × 10^6^ PANC‐1‐KMT2B or PANC‐1‐VC cells, or 2.5 × 10^6^ CFPAC‐1‐KMT2B or CFPAC‐1‐VC cells were inoculated into the head/neck of pancreases. The mice were sacrificed 42 days post‐inoculation. All mice were maintained under specific‐pathogen‐free conditions. The animal experiments were approved by the Animal Care and Use Committees of Shanghai Cancer Institute and performed in accordance with the ARRIVE guidelines.^32^

### 2.5 Tissue microarray and immunohistochemistry

To analyse KMT2B protein expression in human pancreatic tissues, a commercially available tissue microarray containing 80 pathologically confirmed PDAC tissues and 70 adjacent normal pancreatic tissues was subjected to IHC analysis. The samples were collected by the Shanghai National Engineering Research Center from Taizhou Hospital from 2010 to 2014 (approved by the Research Ethics Committee of Taizhou Hospital). The mice orthotopic tumour tissues were also subjected to IHC staining for KMT2B, Ki‐67 and FYN. The IHC staining was performed following a previous protocol.^33^ The primary antibodies used in this study included anti‐KMT2B (1:1000, Abcam), anti‐Ki‐67 (1:2000, Proteintech) and anti‐FYN antibodies (1:400, Proteintech). Haematoxylin and eosin staining was performed to observe the metastases in the lungs and livers of the PDAC orthotopic tumour‐bearing mice. The intensity of KMT2B and FYN staining was evaluated as follows: negative, 0 points; weak, 1 point; moderate, 2 points; and strongly positive, 3 points. The percentage of positive tumour cells was scored as 0 (<5%), 1 (<25%), 2 (25%‒50%), 3 (51%‒75%) and 4 (>75%). The final scores (0‒12) were obtained by multiplying the two scores.

### 2.6 Cell culture and treatment

The human PDAC cell lines AsPC‐1, BxPC‐3, PANC‐1 and CFPAC‐1, and the human normal pancreatic epithelial cell line (hTERT‐HPNE) were obtained from American Type Culture Collection. PANC‐1 and hTERT‐HPNE cells were cultured in high‐glucose Dulbecco's Modified Eagle's Medium (DMEM). BxPC‐3 and AsPC‐1 cells were cultured in Roswell Park Memorial Institute 1640 (RPMI‐1640). CFPAC‐1 cells were cultured in Iscove's Modified Dulbecco's Medium (IMDM). All medium were supplemented with 10% foetal bovine serum (FBS). Cells were cultured at 37°C under 5% CO_2_ and authenticated using short tandem repeat profiling by Biowing Applied Biotechnology Co., Ltd. In specific experiments, the indicated PDAC cells were treated with LY294002 (Cell Signaling Technology) or 5′‐methylthioadenosine (MTA, MedChemExpress) at final concentration of 20 or 500 µM.

### 2.7 Gene overexpression and knockdown

The KMT2B‐overexpressing PDAC cells (PANC‐1‐KMT2B and CFPAC‐1‐KMT2B) were generated by using a modified version of the CRISPR/dCas9 synergistic activation mediator (SAM) system according to the protocols by the Lim Lab.^34^ The PDAC cells infected with an empty virus (PANC‐1‐VC and CFPAC‐1‐VC) were used as control cells.

To construct the KMT2B‐knockdown PDAC cells (PANC‐1‐shKMT2B and CFPAC‐1‐shKMT2B), CFPAC‐1 and PANC‐1 cells were infected with lentivirus (Multiplicity of Infection[MOI] = 10), which expressed short hairpin RNAs (shRNAs) targeting KMT2B. Sequences of shRNA for KMT2B knockdown are listed in Table S1. To knockdown FYN, PDAC cells were transfected with short interfering RNAs (siRNAs) targeting FYN using Lipofectamine 2000 (Invitrogen). PDAC control cells were transfected with scrambled siRNA. The FYN‐targeted siRNAs sequences are listed in Table S2.

### 2.8 Cell Counting Kit‐8 (CCK‐8) assay

The PDAC cells were seeded in 96‐well plates (8 × 10^3^ cells/well), and their viability was assessed at different time points using CCK‐8 reagent (Dojindo Laboratories) according to the manufacturer's protocol.

### 2.9 Transwell assay

Cell migration and invasion abilities were assessed using transwell chambers with an 8 µm pore‐size culture membrane (Corning) covered with or without Matrigel (Corning) before use. For cell migration, 5 × 10^4^ cells were resuspended in 200 µL serum‐free medium and added to the upper compartment. For cell invasion, 1 × 10^5^ cells in 200 µL serum‐free culture medium were added to the compartment with a Matrigel‐coated membrane. Then, 700 µL of medium containing 10% FBS was added to the lower chambers. After incubation (8 h for CFPAC‐1 cells and 24 h for PANC‐1 cells), the cells on the lower surface of the membrane were stained with crystal violet, and the number of cells was then counted.

### 2.10 Western blot

The protein expression level of KMT2B, phospho‐Akt, Akt, FYN, H3K4me3, MMP1 or CCND1 in PDAC cells or mice orthotopic tumour tissues was determined by Western blot analysis. The primary antibodies were as follows: anti‐KMT2B (1:1000, Cell Signaling Technology), anti‐phospho‐Akt (1:1000, Phospho‐Ser473, Cell Signaling Technology), anti‐Akt (1:1000, Proteintech), anti‐MMP1 (1:1000, Proteintech), anti‐FYN (1:1000, Proteintech), anti‐H3K4me3 (1:1000, Abcam), anti‐CCND1 (1:500, Cell Signaling Technology), anti‐vinculin (1:10 000, Santa Cruz Biotechnology) and anti‐GAPDH antibody (1:50 000, Proteintech). The bands were quantified using ImageJ software and the quantitative results were presented as the relative expression levels of target proteins normalised to the corresponding loading controls or pan‐protein levels.

### 2.11 Quantitative reverse transcription PCR

To detect the relative expression of *KMT2B* and *FYN*, total RNA was isolated from the PDAC tissue specimens or cell lines with TRIzol reagent (Thermo Fisher) according to the manufacturer's protocol. The qPCR analyses were performed by using the FastStart Universal SYBR Green kit (Roche) on a StepOne Plus Real‐Time PCR System (Applied Biosystems, Thermo Fisher). The relative expression levels of the target genes were analysed by the 2(−ΔΔCt) method and normalised to actin. The primer sequences are listed in Table S3.

### 2.12 Public database

The mRNA expression levels of *KMT2B* in PDAC and non‐tumour samples from The Cancer Genome Atlas (TCGA) and Genotype‐Tissue Expression (GTEx) databases were analysed using Gene Expression Profiling Interactive Analysis 2 (GEPIA2) (http://gepia2.cancer‐pku.cn/). Significant differences were determined using filter criteria of a *p*‐value cutoff ≤.01 and a log2 fold change (log_2_FC) cutoff ≥.4. Pearson's correlation analysis was conducted to evaluate the relationship between *KMT2B* and *FYN* in PDAC through GEPIA2.

### 2.13 RNA sequencing and gene annotation

RNA sequencing (RNA‐seq) library preparation and sequencing of PANC‐1‐KMT2B and PANC‐1‐VC cells were performed using an Illumina Hiseq ×10 platform by BGI Genetic Corporation. High‐quality reads were aligned to the human reference genome (hg19) using hisat2. Gene expression was calculated from fragments per kilobase of transcript per million mapped reads by RNA‐Seq by Expectation‐Maximization (RSEM). The filter criteria of *p *< .001 and FC ≥ 2 were used to determine the differential expression of genes (DEGs). Kyoto Encyclopedia of Genes and Genomes (KEGG) pathway (https://www.kegg.jp/) enrichment of our RNA‐seq profiles was performed by Phyper (https://en.wikipedia.org/wiki/Hypergeometric_distribution) based on the hypergeometric test.

### 2.14 Chromatin immunoprecipitation sequencing and chromatin immunoprecipitation quantitative real‐time PCR

PANC‐1‐VC and PANC‐1‐KMT2B cells (1.6 × 10^7^) were harvested and crosslinked with 1% formaldehyde and then quenched with 125 mM glycine. Nuclear lysate was sonicated using a Bioruptor Pico sonicator (Diagenode) at ‘high power’ for 35 cycles at 4°C to obtain chromatin fragments with sizes ranging from 200 to 500 bp. For chromatin immunoprecipitation (ChIP) reactions, anti‐H3K4me3 (1:100, Abcam) or KMT2B (1:100, Cell Signaling Technology) antibodies were added to the chromatin lysate and incubated overnight at 4°C. Protein G Dynabeads (Cell Signaling Technology) were then added to the reaction mixture. After further incubation overnight at 4°C, the beads were collected. The ChIPed DNA was purified with a QIAquick Gel Extraction Kit (Qiagen). Libraries were prepared from ChIPed DNA and sequenced with the Illumina HiSeq 2500 platform by RiboBio. The reads were mapped to the human genome (hg19) by Bowtie2 software 38. The ChIP sequencing (ChIP‐seq) profiles of this study were uploaded to the NCBI database with an accession number as PRJNA985215. The ChIPed DNA and the input controls were also subjected to qPCR to validate the ChIP‐seq result. The primer sequences are listed in Table S4.

### 2.15 Dual luciferase reporter assay

The human *FYN* promoter regions spanning nucleotides ‒1783 to 23 (relative to the transcription initiation site) were amplified by PCR from PANC‐1 cells and cloned into the NheI/HindIII sites of the pGL3‐basic vector (Promega) to generate the *FYN* promoter luciferase reporter (pGL3‐FYN). PANC‐1‐VC cells or PANC‐1‐KMT2B cells were co‐transfected with pGL3‐FYN (2 µg) and pRL‐TK Renilla luciferase expression plasmid (2 µg). After 48 h transfection, the luciferase signals were determined with a Dual Luciferase Reporter Assay Kit (Promega) according to the manufacturer's instructions with the GloMax R luminometer (Promega).

### 2.16 Statistics

A binomial test was used to analyse the HBV integration frequency in PDAC and para‐tumour tissues between the observed and expected groups. Student's *t*‐test (for normally distributed variables) or the Wilcoxon rank sum test (for non‐normally distributed variables) was used to compare continuous variables. Fisher's exact test was applied to assess the association between categorical variables in a contingency table. The statistical analysis was performed using SPSS software version 22.0 (SPSS Inc.) or GraphPad Prism (GraphPad Software).

## 3 RESULTS

### 3.1 Detection of HBV DNA in pancreatic cancer and para‐tumour tissues

Forty‐three PDAC cases with HBV infection and nine cases without were included in the study. The viral S or C genes were amplified in 25.60% (11/43) of the tumour tissues and 27.90% (12/43) of the para‐tumour tissues (Figure S1 and Table S5). The vast majority (22/23) of HBV DNA‐positive tissues contained both HBV S and C genes. Of the seven HBsAg‐positive PDAC cases, 100% exhibited HBV DNA in their tumour or non‐tumour tissues. In contrast, among the 36 HBsAg‐negative cases, only six (16.60%) had HBV DNA present in the pancreatic tissues, suggesting that persistent HBV infection increases the likelihood of the virus affecting the pancreas. It is worth noting that all cases with HBV DNA in the pancreas were at the inactive stage of chronic hepatitis, suggesting that HBV DNA may persist in the pancreas for a long time even after HBeAg clearance. For the nine PDAC cases negative for HBV serum markers, none showed PCR‐positive results for the HBV S and C genes (Figure S1).

### 3.2 Identification of HBV DNA integration in PDAC and para‐tumour tissues

**FIGURE 1:**
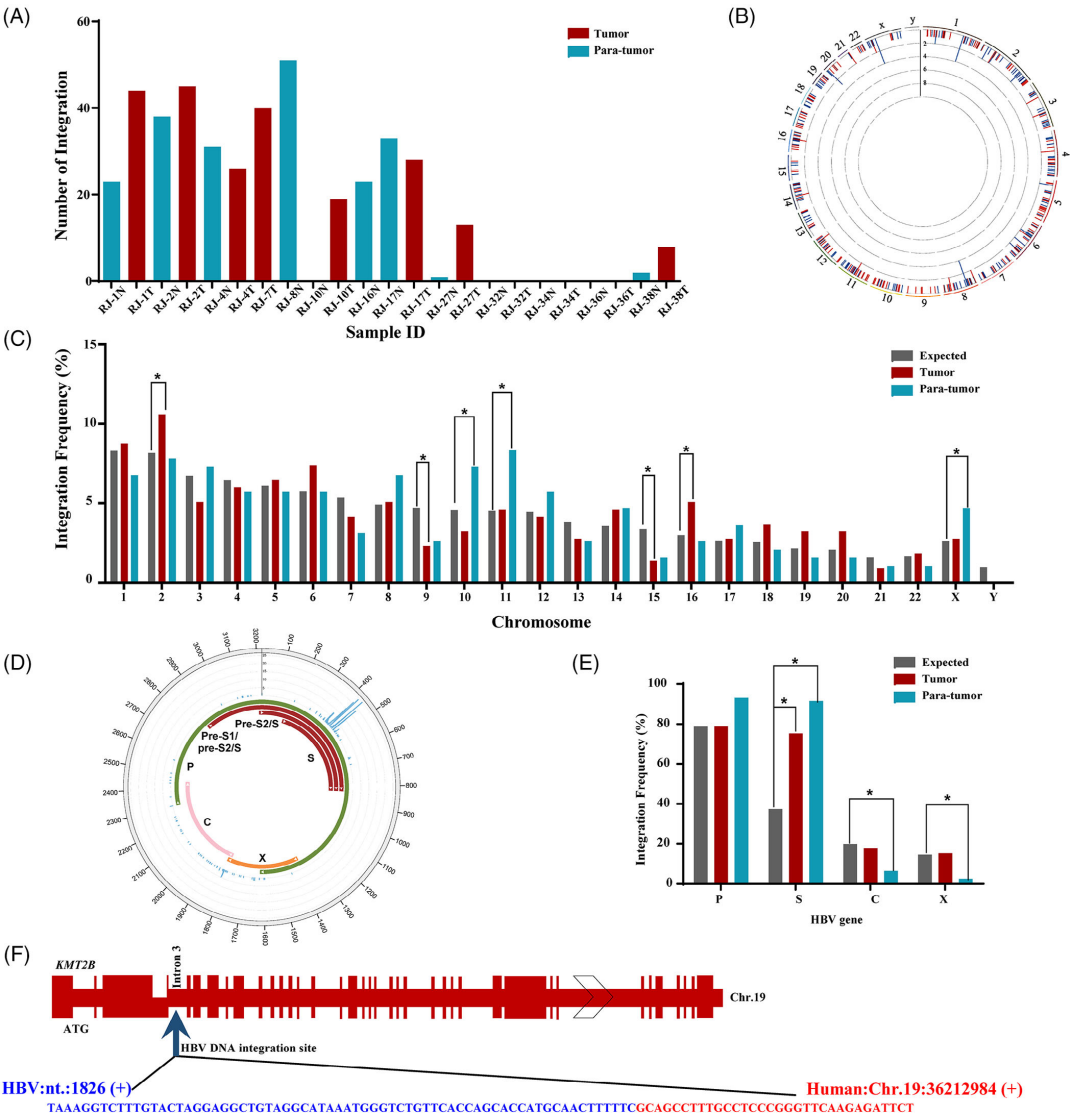
Analysis of hepatitis B virus (HBV) DNA integration in pancreatic ductal adenocarcinoma (PDAC) tumour and para‐tumour tissues. (A) The number of HBV integrations in each PDAC case. (B) Distribution of 425 HBV integration breakpoints identified from eight PDAC tissues and eight para‐tumour tissues throughout the human genome (hg19). Each bar represents the number of HBV integration at a particular human genomic locus. Red: HBV integrations in the gene regions; blue: HBV integrations in the intergenic regions. Histogram axis units represent the number of integrations. (C) The frequency of HBV integration in each chromosome. Red: observed frequency of HBV integration in tumour; blue: observed frequency of HBV integration in para‐tumour; grey: expected frequency of HBV integration (calculated using assuming uniform, random distribution). (D) Distribution of HBV integration sites in the HBV genome (NC_003977). Each bar represents the number of viral‒host junction sites at the HBV genome. The genes encoding HBV polymerase (green), core (pink), S (red) and X (orange) proteins are marked. (E) The frequency of HBV DNA integration breakpoints in HBV protein‐coding genes. Red: observed frequency of HBV integration in tumour; blue: observed frequency of integrated HBV DNA fragment in para‐tumour; grey: expected frequency of integrated HBV DNA fragment (calculated using assuming uniform, random distribution). (F) Map of the *KMT2B* gene with HBV DNA insertion in its third intron. ^*^ *p* < .05.

Genomic DNAs of 23 tissues with detectable HBV DNA were subjected to HIVID. HBV integration was found in 72.70% (8/11) of PDAC tissues and 66.70% (8/12) of para‐tumour tissues, among which six were paired tumour and non‐tumour samples (Figure S2). A total of 425 viral‒host junctions were identified (Figure 1A,B). Detailed information on these 425 integration sites is listed in Table S6. HBV integration frequencies were relatively uniform between cancerous and adjacent non‐cancerous tissues within the same case. Specifically, cases 1, 2, 4 and 17 exhibited high integration rates in both cancerous and adjacent non‐cancerous tissues, while cases 27 and 38 showed low integration rates in both tissue types (Figure 1A). As a result, there was no significant difference in the average number of integration sites between tumour and para‐tumour samples (27.63 ± 14.45 vs. 25.25 ± 17.14). Clinically, HBV integration was more frequently detected in well or moderately differentiated PDACs than in poorly differentiated PDACs (grade I‒II vs. grade III, 100% *vs*. 0%, Table S7). This aligns with our previous report that HBV antigens are typically observed in well‐differentiated PDACs.^4^

In PDAC, approximately half (50.22%) of HBV integrations occurred in intergenic regions, with the remainder distributed across intronic regions (45.29%), exonic regions (2.69%), 3′‐untranslated regions (1.35%) and gene upstream regions (.45%). A similar integration distribution pattern was observed in the adjacent non‐tumour tissues (Table 1). Seven protein‐coding genes (*PCDH10*, *QSER1*, *KCNJ16*, *KRTAP10‐9*, *SLC25A51*, *MROH2A* and *MYL4*) and two non‐coding RNA genes (*PRMT5‐AS1* and *LOC100507412*) were identified as being disrupted by HBV DNA integration within their exons (Table S6). Recurrent HBV integration sites, defined as those where the virus repeatedly targets the same gene or inserts into the vicinal intergenic sequences within a 50 kb distance across different tissue samples or cell populations from the same tissue are summarised in Table 2.

**TABLE 1:** Category of hepatitis B virus integration sites in the genome of pancreatic cancer tissues and para‐tumour tissues.

Category	Tumour tissue (%)	Para‐tumour tissue (%)	Total (%)
Exon	6 (2.69)	4 (1.98)	10 (2.35)
Intron	101 (45.29)	83 (41.09)	184 (43.29)
5′‐UTR	0 (0)	2 (0.99)	2 (0.47)
3′‐UTR	3 (1.35)	2 (0.99)	5 (1.18)
Upstream (<1 kb)	1 (0.45)	2 (0.99)	3 (0.71)
Downstream (<1 kb)	0 (0)	3 (1.49)	3 (0.71)
Intergenic	112 (50.22)	106 (52.48)	218 (51.29)
1‒10 kb	14 (6.28)	12 (5.94)	26 (6.12)
10‒50 kb	22 (9.87)	21 (10.4)	43 (10.12)
50‒100 kb	23 (10.31)	21 (10.4)	44 (10.35)
100‒500 kb	46 (20.63)	46 (22.77)	92(21.65)
>500 kb	1 (0.45)	1 (0.50)	2 (0.47)
Unknown	6 (2.69)	5 (2.48)	16 (3.76)
Total	223 (100.00)	202 (100.00)	425 (100.00)

Abbreviation: UTR, untranslated region.

**TABLE 2:** Recurrent hepatitis B virus (HBV) integration sites in pancreatic cancer tissues and para‐tumour tissues.

Gene (element)	Human genome	HBV genome	HBV gene	Gene type	Gene function	Sample
*HELQ*						
Intron 8	chr4:84360311	428	S/P	Protein coding	DNA repair	RJ‐7T
Intron 8	chr4:84360603	428	S/P			RJ‐7T
*KRTAP10‐9*						
Exon 1	chr21:46047598	1650	X/P	Protein coding	Keratinisation development	RJ‐38T
Exon 1	chr21:46047598	1650	X/P			RJ‐27T
*PNPT1*						
Intergenic	chr2:55851582	423	S/P	Protein coding	RNA metabolic processes	RJ‐7T
Intron 3	chr2:55913359	427	S/P			RJ‐17T
*SLC22A11*						
Intergenic	chr11:64298618	507	S/P	Protein coding	Anions transportation and excretion	RJ‐2T
Intron 1	chr11:64325096	646	S/P			RJ‐27T
*KSR2*						
Intron 3	chr12:118209807	396	S/P	Protein coding	ERK, JNK and NF‐κB pathways; MAP3K8‐mediated and MAP3K3‐mediated pathways; MEK/RAF pathway	RJ‐4N
Intron 3	chr12:118209887	398	S/P			RJ‐4N
*LOC100507412, RNA5‐8S5*						
Intergenic	chrUn_gl000220:131469	421	S/P	Non‐coding RNA, rRNA	Unknown, peptide chain elongation	RJ‐10T
Intergenic	chrUn_gl000220:132883	424	S/P			RJ‐1T
Intergenic	chrUn_gl000220:131665	427	S/P			RJ‐2T
Intergenic	chrUn_gl000220:131834	423	S/P			RJ‐4T
Intergenic	chrUn_gl000220:131880	427	S/P			RJ‐4T
Intergenic	chrUn_gl000220:127859	424	S/P			RJ‐16N
Intergenic	chrUn_gl000220:132067	423	S/P			RJ‐16N
Intergenic	chrUn_gl000220:132102	427	S/P			RJ‐16N
Intergenic	chrUn_gl000220:132142	423	S/P			RJ‐17N
*LOC100507412*						
Downstream	chrUn_gl000220:127352	424	S/P	Non‐coding RNA	Unknown	RJ‐1N
Exon	chrUn_gl000220:121616	1998	C			RJ‐2N
Downstream	chrUn_gl000220:127671	423	S/P			RJ‐8N

Abbreviations: ERK, extracellular regulated protein kinases; HELQ, helicase POLQ‐like; JNK, C‐Jun N‐terminal kinase; KRTAP10‐9, keratin associated protein 10‐9; KSR2, kinase suppressor of Ras 2; MAP3K8, mitogen‐activated protein kinase 8; MEK, mitogen‐activated protein kinase; N, para‐tumour tissues; NF‐κB, nuclear factor‐kappa B; PNPT1, polyribonucleotide nucleotidyltransferase 1; RAF, Raf‐1 proto‐oncogene, serine/threonine kinase; SLC22A11, solute carrier family 22 member 11; T, pancreatic cancer tissues.

HBV DNA was found to repeatedly integrate into five protein‐coding genes (*HELQ*, *KRTAP10‐9*, *PNPT1*, *SLC22A11* and *KSR2*), one non‐coding RNA gene (*LOC100507412*), and one ribosomal RNA‐coding gene (*RNA5‐8S5*). Notably, of the five protein‐coding genes repeatedly targeted by HBV, four were identified in tumour tissues, suggesting that cells with HBV integration may have undergone clonal expansion during the development of PDAC due to the growth advantage conferred by viral integration. An interesting observation is that HBV integration not only had its preferential target genes, but also had its particular insertional sites within those genes. For example, in sample 27T and 38T, HBV DNA integrated into the same position within the first exon of the *KRTAP10‐9* gene. In sample 7T, HBV DNA repeatedly inserted into intron 8 of the *HELQ* gene, with the two insertion events occurring just 292 bp apart. More strikingly, in six different samples, HBV inserted into a very close region for nine times. These results supported that HBV integration is not a random event in PDAC.

In PDAC, HBV DNA integration more frequently located in human chromosomes 2 and 16, and less frequently located in chromosomes 9 and 15 when compared to the expected integration frequency (*p* < .05, Figure 1C). Differently, in the para‐tumour tissues, integration occurred more frequently in chromosomes 10, 11 and X (*p* < .05, Figure 1C). In the context of viral genome, 75.30% (168/223) breakpoints in PDAC tissues were mapped to HBV S gene. This rate was significantly higher than its expected rate (*p* < .05, Figure 1D,E). Similar pattern was observed in the para‐tumour tissues, where 91.60% (185/202) integration took place in HBV S gene (Figure 1D,E).

### 3.3 Analysis of the co‐target genes of HBV integration in HCC and PDAC

To compare the HBV integration sites between PDAC and HCC, we first compiled viral‒host junction data from 48 published studies on HCC, creating a dataset that includes 11 493 HBV integration breakpoints and 3441 integration‐affected genes reported to date (Table S8). Forty genes were found to be co‐targeted by HBV DNA in both PDAC and HCC (Table S9). Most of these HBV co‐target genes (72.50%, 29/40) harboured viral DNA within their introns. The non‐coding RNA gene *LOC100507412*, which was integrated by HBV 12 times in PDAC, was also found to be repeatedly targeted by HBV five times in HCC (Table 2). Of note, *KMT2B*, one of the most preferential target genes for HBV integration in HCC, was interrupted by HBV DNA in one PDAC sample. The HBV‒*KMT2B* junction isolated from PDAC shared a very similar pattern to that observed in HCC, specifically the insertion of the 5′‐deleted X gene into the third intron of *KMT2B* (Figure 1F). This result implied that HBV integration targeting *KMT2B* plays a role in carcinogenesis across different cancer types.

### 3.4 KMT2B is upregulated in pancreatic cancer

**FIGURE 2:**
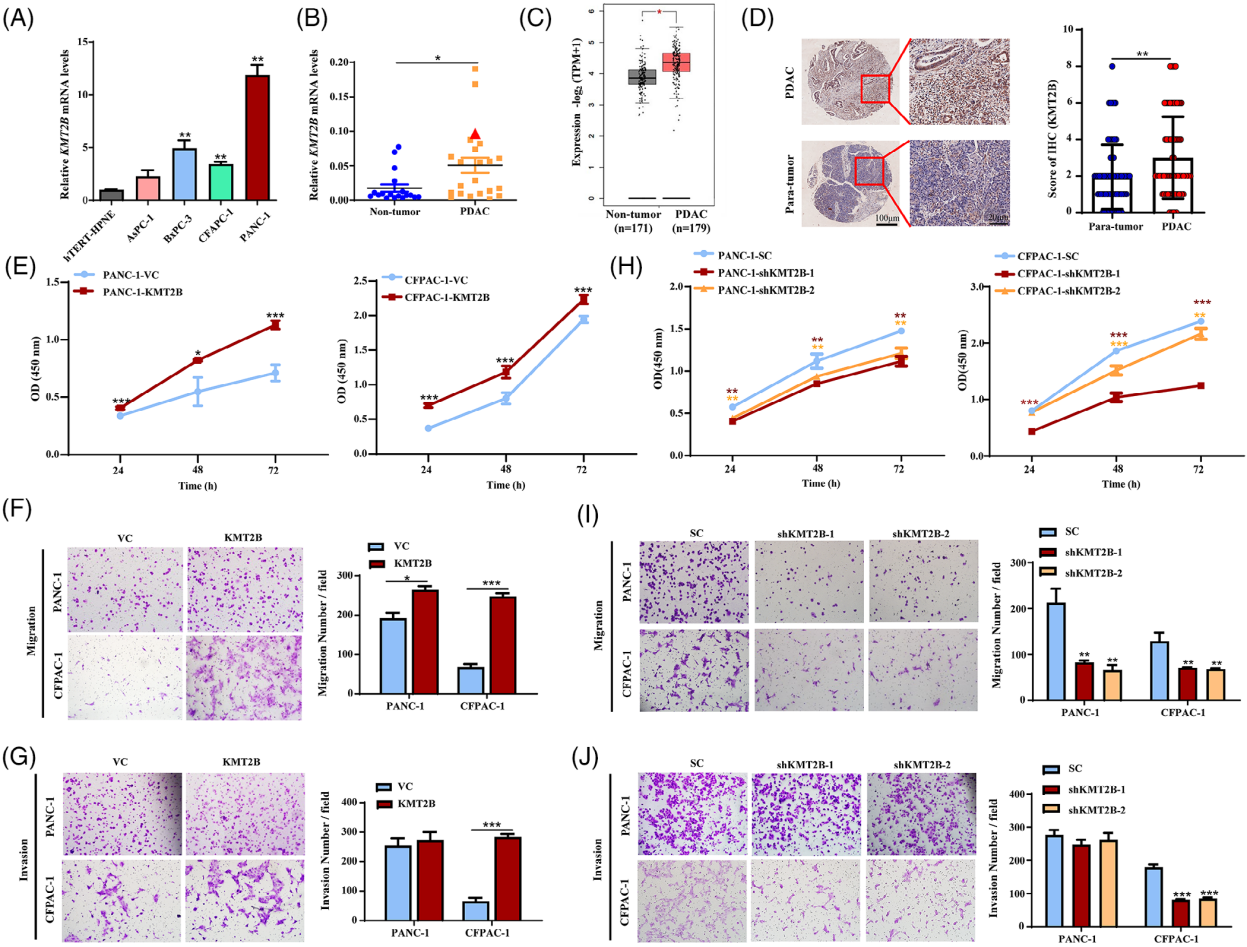
KMT2B is upregulated in pancreatic ductal adenocarcinoma (PDAC) and promotes the malignant behaviours of PDAC cells. (A) The mRNA level of *KMT2B* in a series of PDAC cell lines (AsPC‐1, BxPC‐3, CFPAC‐1 and PANC‐1) and the normal pancreatic dual cells (hTERT‐HPNE) revealed by qPCR. (B) Comparison of *KMT2B* mRNA levels between 22 PDAC tissues and 18 para‐tumour tissues. The red triangle represents the PDAC case (27T), which has hepatitis B virus (HBV) integration in *KMT2B* gene. (C) Comparison of *KMT2B* mRNA levels between 179 PDAC tissues and 171 normal pancreatic tissues using the data from The Cancer Genome Atlas (TCGA) and Genotype‐Tissue Expression (GTEx) datasets. (D) The immunohistochemistry (IHC) analysis of tissue microarray containing 80 PDAC tissues and 70 normal pancreatic tissues. Left: representative images of KMT2B revealed by IHC (scale bar, left: 20 µm; right: 100 µm). Right: quantitative analysis of KMT2B staining. (E) Cell proliferation of KMT2B‐overexpressing PDAC cells (PANC‐1‐KMT2B or CFPAC‐1‐KMT2B) and control cells (PANC‐1‐VC or CFPAC‐1‐VC) detected by CCK‐8 assay. The migration (F) and invasion (G) abilities of KMT2B‐overexpressing PDAC cells and control cells measured by transwell assay. Left: representative images of crystal violet staining of the cells migrating through the membranes. Right: quantitative analysis of the crystal violet‐stained cells. (H) Cell proliferation of KMT2B‐knockdown PDAC cells (PANC‐1‐sh*KMT2B* and CFPAC‐1‐sh*KMT2B*) and control cells (PANC‐1‐SC or CFPAC‐1‐SC) detected by CCK‐8. The migration (I) and invasion (J) abilities of KMT2B‐knockdown PDAC cells and control cells measured by transwell assay. Left: representative images of crystal violet staining of the cells migrating through the membranes. Right: quantitative analysis of the crystal violet‐stained cells. ^*^ *p *< .05; ^**^ *p* < .01; ^***^ *p* < .001.

The mRNA level of *KMT2B* in four PDAC cell lines (AsPC‐1, BxPC‐3, CFPAC‐1 and PANC‐1) and one normal pancreatic ductal cell line (hTERT‐HPNE) was examined by qPCR. The results showed that *KMT2B* expression was higher in all of the four PDAC cell lines compared to the normal pancreatic ductal cell line (Figure 2A). Similarly, a significantly increased mRNA level of *KMT2B* was shown in 22 human PDAC samples compared to the 18 non‐tumour tissue samples (*p* < .05, Figure 2B). The PDAC case with HBV integration in its *KMT2B* gene, as indicated by the triangle in Figure 2B, ranked among the top three highest *KMT2B* expression levels out of the 40 samples tested. Analysis of the TCGA and GTEx datasets containing 179 PDACs and 171 non‐tumour pancreatic tissues revealed there was an obvious elevation in the mRNA level of *KMT2B* in cancer tissues (*p* < .05, Figure 2C). IHC analysis of a tissue microarray, comprising 80 PDACs and 70 adjacent non‐tumour tissues, confirmed a significant elevation of KMT2B protein levels in PDACs (*p* < .01, Figure 2D). The association between *KMT2B* expression and PDAC patient survival showed inconsistencies across different public databases. In the KMplot database, *KMT2B* expression showed no correlation with overall survival (Figure S3A). Conversely, in the GEO dataset GSE62452, higher *KMT2B* expression was negatively associated (*p* < .05) with survival (i.e., correlated with shorter survival, Figure S3B). Paradoxically, in the TCGA dataset, PDAC patients with higher *KMT2B* expression exhibited significantly prolonged survival compared to those with lower expression (Figure S3C). Since patient survival outcomes depend not only on tumour biology but also on treatment efficacy, these inconsistent findings may be related to differences in treatment regimens among patients across the cohorts.

### 3.5 KMT2B promotes the malignant behaviour of PDAC

As KMT2B is a large protein with a molecular weight of 294 kDa, we employed CRISPR/dCas9 SAM system to achieve endogenous overexpression of KMT2B in PDAC cell lines. KMT2B overexpression (Figure S4A,B) significantly increased the cell proliferation rate (*p *< .05, Figure 2E) and migration abilities (*p *< .05, Figure 2F) in PANC‐1 and CFPAC‐1 cells, and it also enhanced the cell invasiveness in CFPAC‐1 cells (*p* < .001, Figure 2G). On the contrary, knocking down *KMT2B* by shRNA interference (Figure S5A,B) significantly inhibited cell proliferation, migration and invasion in PDAC cells (*p* < .05, Figure 2H‒J).

**FIGURE 3:**
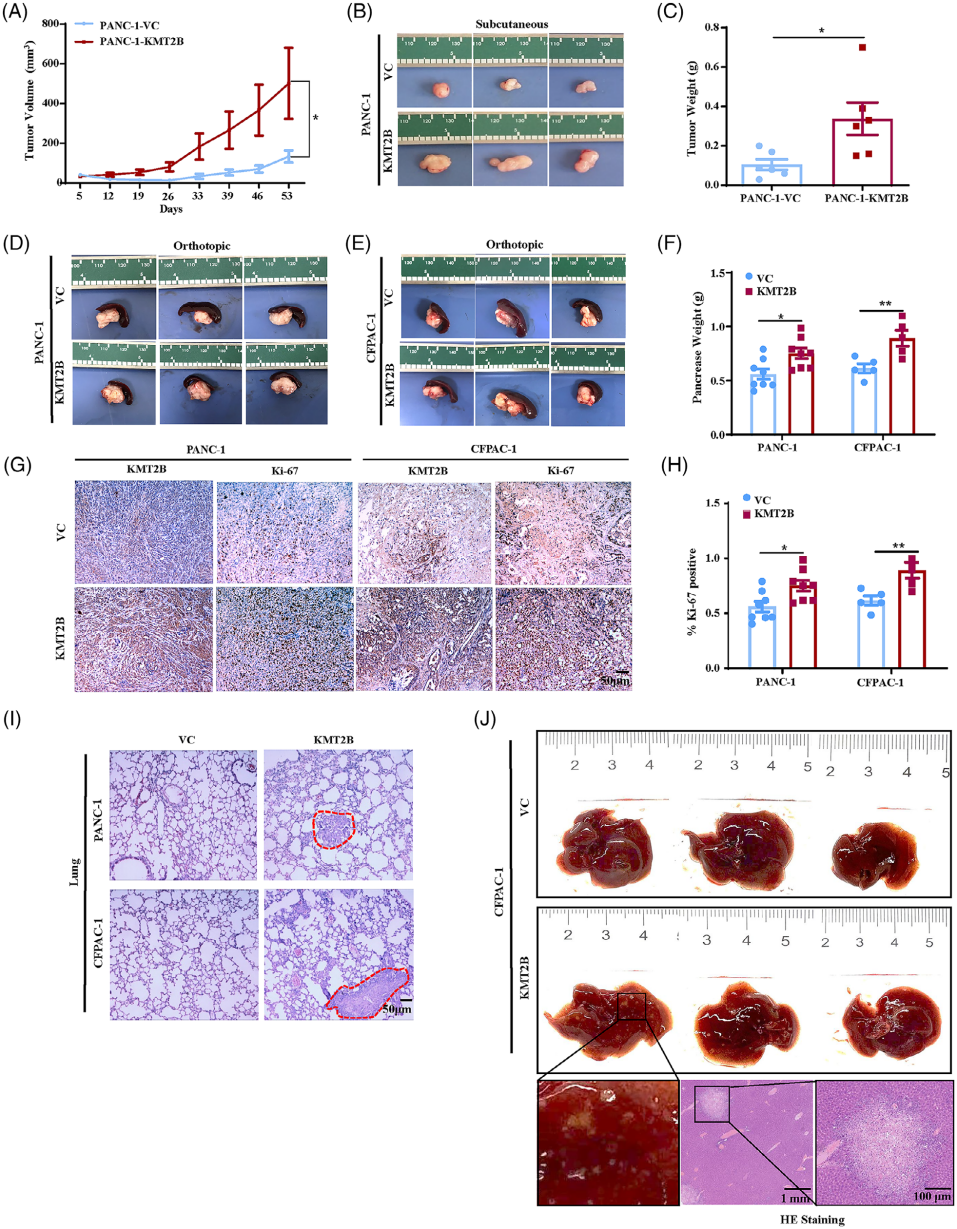
KMT2B promotes pancreatic ductal adenocarcinoma (PDAC) growth and metastasis in vivo. The subcutaneous tumour model formed by PANC‐1‐KMT2B and control PANC‐1‐VC cells in BALB/c nude mice (*n* = 7 per group). (A) The tumour growth curves based on tumour sizes measured at a 7‐day interval. The data are presented as the mean ± SEM (Standard Error of the Mean). (B) Representative images of tumours at the time of sacrifice 53 days after tumour cells inoculation. (C) The tumour weights at the time of sacrifice. The data are presented as the mean ± SEM. The orthotopic tumours formed by PANC‐1‐KMT2B and the control PANC‐1‐VC cells (*n* = 8 per group), and CFPAC‐1‐KMT2B and the control CFPAC‐1‐VC cells (*n* = 5 per group), respectively. (D) Representative images of pancreas collected from PANC‐1‐VC or PANC‐1‐KMT2B‐bearing mice at the time of sacrifice 42 days after tumour inoculation. (E) Representative images of tumours collected from CFPAC‐1‐VC or CFPAC‐1‐KMT2B‐bearing mice at the time of sacrifice 42 days after tumour inoculation. (F) Tumour weights of each group at the time of sacrifice. The data are presented as the mean ± SEM. (G) Representative images of immunohistochemistry (IHC) staining of KMT2B and Ki‐67 in the pancreatic tumours (scale bar, 50 µm). (H) Quantification of the percentage of Ki‐67‐positive cells. (I) Representative images of haematoxylin and eosin (H&E)‐stained lung sections. The red dashed line highlights the metastatic lesions in the lung (scale bar, 50 µm). (J) Representative images of the livers with metastatic nodules collected from orthotopic pancreatic tumour‐bearing mice of CFPAC‐1 cells (*n* = 8 per group). Black rectangle highlights metastatic tumours. Representative images of H&E‐stained liver sections. The black rectangle highlights metastatic lesions in the liver (scale bar, 100 µm). ^*^ *p *< .05; ^**^ *p* < .01.

The in vivo role of KMT2B in tumour growth and metastasis was evaluated in subcutaneous and orthotopic mouse models of human PDAC. In the subcutaneous model, tumours derived from PANC‐1‐KMT2B cells grew significantly faster than those in the control group (*p* < .05, Figure 3A). At the sacrifice time, overexpression of KMT2B resulted in a more than threefold increase in tumour weight compared to the control group (.11 ± .07 g vs.  .34 ± .20 g, *p* < .05, Figure 3B,C). Furthermore, KMT2B overexpression also significantly increased the tumour burden in orthotopic tumour models of PANC‐1 (.56 ± .14 g vs.  .75 ± .14 g, *p* < .05, Figure 3D,F) and CFPAC‐1 cells (.61 ± .09 g vs.  .89 ± .16 g, *p* < .05, Figure 3E,F; independent replicate experiment:  .43 ± .09 g vs.  .54 ± .18 g, *p* < .05, Figure S6A). Ki‐67 staining indicated that KMT2B promoted cancer cell proliferation (*p *< .05, Figure 3G,H). Liver metastases were observed in 25% (2/8) of PANC‐1‐KMT2B mice and 40% (2/5) of CFPAC‐1‐KMT2B mice, whereas no metastases were found in the control mice inoculated with PANC‐1 or CFPAC‐1 cells (Figure S6C). The metastatic nodules in the liver were pathologically confirmed with HE staining (Figure S6D). Micro‐metastatic lesions in lung were also observed in 12.50% (1/8) of PANC‐1‐KMT2B mice and 40% (2/5) of CFPAC‐1‐KMT2B mice, while none were found in the control mice (Figure 3I). In the independent replicate experiment using orthotopic PDAC model established with CFPAC‐1 cells, liver metastases were observed in 12.50% (1/8) of CFPAC‐1‐VC mice and 75% (6/8) of CFPAC‐1‐KMT2B mice (12.50% vs. 75%, *p* < .05, Figure 3J), confirming that KMT2B has the potential to promote PDAC's liver metastasis. Collectively, our in vitro and in vivo results clearly demonstrated that KMT2B plays an important role in promoting PDAC progression.

### 3.6 KMT2B exerts its pro‐tumour function via the PI3K/Akt pathway

**FIGURE 4:**
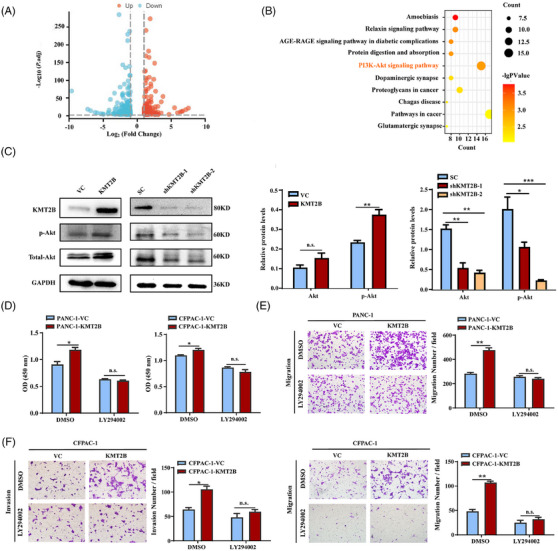
KMT2B promotes the malignant behaviours of pancreatic ductal adenocarcinoma (PDAC) cells through the PI3K/Akt pathway. (A) The volcano graph showing the differentially expression genes (DEGs; adjusted *p* ≤ .001) identified by RNA‐sequencing (RNA‐seq) between PANC‐1‐KMT2B and PANC‐1‐VC cells. (B) The top 10 pathways revealed by Kyoto Encyclopedia of Genes and Genomes (KEGG) enrichment analysis of DEGs. (C) Western blot analysis of the protein levels of Akt and p‐Akt in KMT2B‐overexpressing and KMT2B‐knockdown PANC‐1 cells. KMT2B was cleaved by Taspase 1 resulting in the generation of a C‐terminal fragment of 80 kDa. Right: quantitative analysis of the relative protein levels by ImageJ. (D) Cell proliferation of KMT2B‐overexpressing PDAC cells (PANC‐1‐KMT2B and CFPAC‐1‐KMT2B) and control cells (PANC‐1‐VC and CFPAC‐1‐VC) with or without the presence of 20 µM PI3K inhibitor LY294002 for 48 h measured by CCK‐8 assay. The migration (E) and invasion (F) abilities of KMT2B‐overexpressing PDAC cells and control cells with or without the presence of 20 µM LY294002 measured by transwell assay. Left: representative images of crystal violet staining of the cells that migrating through the membranes. Right: quantitative analysis of the crystal violet‐stained cells. ^*^ *p *< .05; ^**^ *p *< .01; ^***^ *p* < .001.

To understand the potential mechanisms underlying the tumour‐promoting function of KMT2B, we first compared the mRNA expression profiles between the PANC‐1‐KMT2B and the PANC‐1‐VC control cells. Three hundred and twenty‐three DEGs (163 upregulated and 160 downregulated) were identified based on the thresholds of FC ≥2 and adjusted *p* < .001 (Figure 4A). KEGG analysis of the DEGs revealed that the PI3K/Akt signalling pathway was significantly enriched in KMT2B‐overexpressing PDAC cells (adjusted *p* < .05, Figure 4B). Western blot analysis revealed that KMT2B overexpression substantially increased the total Akt and p‐Akt levels in PDAC cells, whereas knocking down of *KMT2B* reduced the levels of Akt and p‐Akt, confirming that KMT2B governs the PI3K/Akt signalling pathway (Figure 4C). *MMP1* and *CCND1*, two well‐established downstream effectors of Akt, were significantly upregulated in KMT2B‐overexpressing cells (Figure S7A,B). This suggests that the PI3K/Akt/CCND1 and PI3K/Akt/MMP1 axes may contribute to the enhancement of cell proliferation and invasion induced by KMT2B. To verify this, we treated the cells with the PI3K inhibitor LY294002. The results demonstrated that LY294002 successfully abolished KMT2B‐induced increases in cell proliferation, migration and invasion (Figure 4D‒F), confirming that PI3K mediates KMT2B‐induced pro‐tumour effects in PDAC.

### 3.7 FYN is a downstream target of KMT2B that activates the PI3K/Akt pathway

**FIGURE 5:**
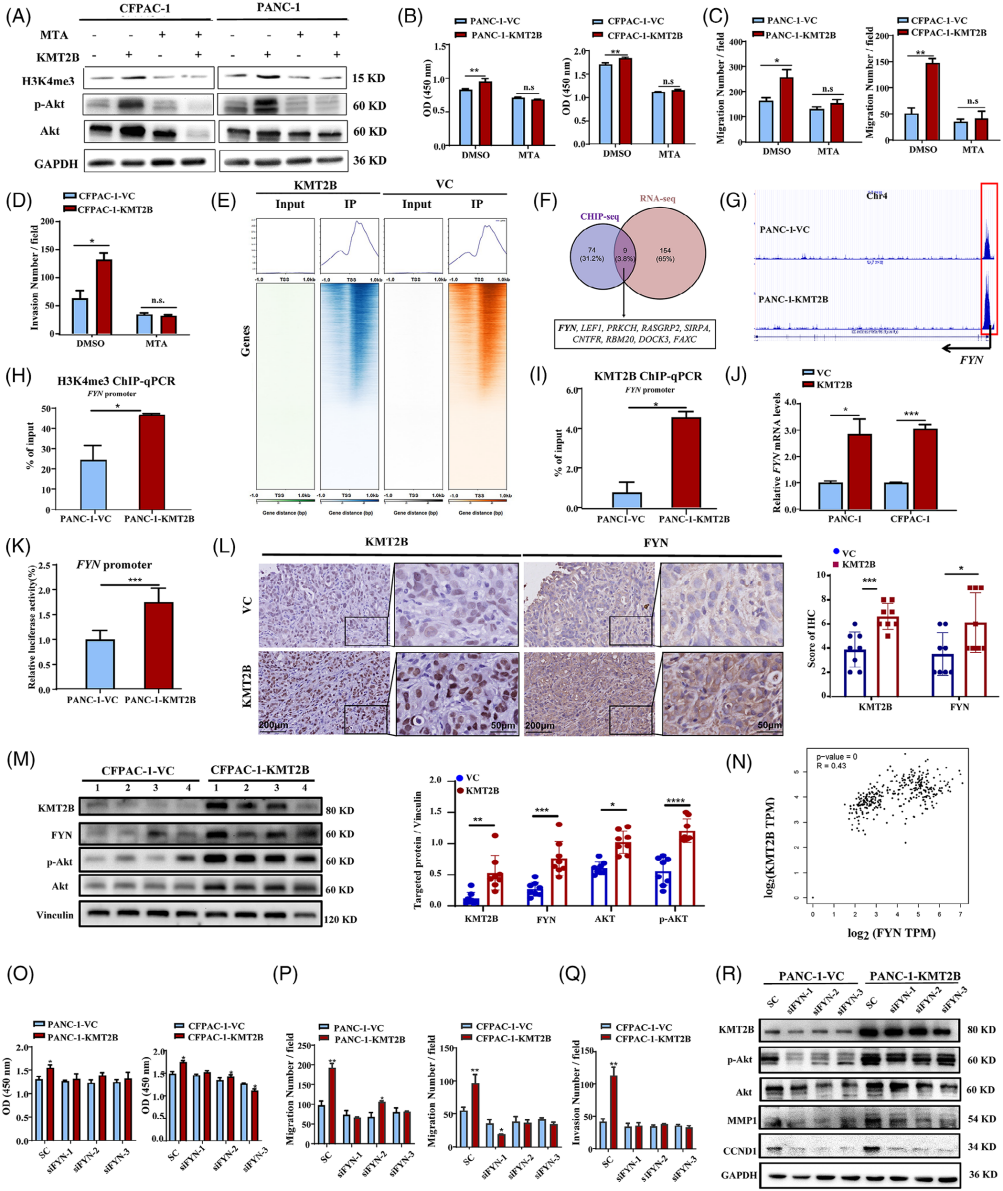
*FYN* is a target gene of KMT2B in pancreatic ductal adenocarcinoma (PDAC) cells. (A) Western blot analysis of the protein levels of H3K4me3, AKT and p‐Akt in KMT2B‐overexpressing PDAC cells (PANC‐1‐KMT2B and CFPAC‐1‐KMT2B cells) and control cells (PANC‐1‐VC and CFPAC‐1‐VC cells) with or without the presence of 500 µM MTA (a blocker specific for H3K4 methylation). (B) Cell proliferation of KMT2B‐overexpressing PDAC cells and control cells with or without the presence of 500 µM MTA for 48 h detected by CCK‐8 assay. The migration (C) and invasion (D) abilities of KMT2B‐overexpressing PDAC cells with or without the presence of 500 µM MTA measured by transwell assay. (E) The heatmaps and signal density plots centered on transcriptional start sites of genes that targeted by H3K4me3 in PANC‐1‐KMT2B and PANC‐1‐VC cells identified by H3K4me3‐based chromatin immunoprecipitation sequencing (ChIP‐seq). (F) Venn diagram of the upregulated genes (fold change ≥2, adjusted *p* < .001) in KMT2B‐overexpressing cells identified by RNA‐sequencing (RNA‐seq) and the genes with increased H3K4me3 enrichment at their promoter regions (fold change ≥2, *p* < .001) in KMT2B‐overexpressing cells identified by ChIP‐seq. (G) Visualisation of H3K4me3 enrichment at the promoter region of *FYN* gene in PANC‐1‐VC and PANC‐1‐KMT2B cells. Note that the signal strength for the H3K4me3 peak at the promoter region of *FYN* gene was higher in PANC‐1‐KMT2B cells than that in PANC‐1‐VC cells. (H) H3K4me3‐based chromatin immunoprecipitation quantitative real‐time PCR (ChIP‐qPCR) analysis of H3K4me3 enrichment at the promoter region of *FYN* gene in PANC‐1‐VC and PANC‐1‐KMT2B cells. (I) KMT2B‐based ChIP‐qPCR analysis of KMT2B enrichment at the promoter region of *FYN* gene in PANC‐1‐VC and PANC‐1‐KMT2B cells. (J) The mRNA level of *FYN* in KMT2B‐overexpressing PDAC cells and control cells revealed by qPCR. (K) The activity of *FYN* promoter in PANC‐1‐VC and PANC‐1‐KMT2B cells detected by luciferase reporter assay. The relative luciferase signal in the PANC‐1‐VC group was set to 1. (L) Immunohistochemistry (IHC) analysis of mice orthotopic tumour tissues formed by CFPAC‐1‐VC and CFPAC‐1‐KMT2B cells. Left: representative images of KMT2B revealed by IHC (scale bar, left: 200 µm; right: 50 µm). Right: quantitative analysis of KMT2B and FYN staining (*n* = 8 per group). (M) Western blot analysis of the protein levels of KMT2B, FYN, p‐Akt and Akt in mice orthotopic tumour tissues (*n* = 8 per group). Left: representative images; right: quantitative analysis of the protein level by ImageJ. (N) Pearson correlation analysis of *KMT2B* and *FYN* levels in PDAC tumour and para‐tumour tissues using the data from The Cancer Genome Atlas (TCGA) and Genotype‐Tissue Expression (GTEx) databases. (O) Cell proliferation of KMT2B‐overexpressing PDAC cells (PANC‐1‐KMT2B and CFPAC‐1‐KMT2B cells) and control cells (PANC‐1‐VC and CFPAC‐1‐VC cells) with or without FYN knockdown detected by CCK‐8 assay. The migration (P) and invasion (Q) abilities of KMT2B‐overexpressing PDAC cells and control cells with or without FYN knockdown measured by transwell assay. Quantitative analysis was performed by counting crystal violet‐stained cells that migrating through the membrane. (R) Western blot analysis of the protein levels of KMT2B, Akt, p‐Akt, MMP1 and CCND1 in PANC‐1‐VC and PANC‐1‐KMT2B cells with or without FYN knockdown. The data are presented as the mean ± SEM. ^*^ *p *< .05; ^**^ *p* < .01; ^***^ *p* < .001.

The primary function of KMT2B is the trimethylation of H3K4, a process associated with the regulation of gene transcription. As expected, we observed an increased level of H3K4me3 in KMT2B‐overexpressing PDAC cell lines (Figure 5A). Treatment with MTA, a specific blocker of H3K4 methylation, abolished the KMT2B‐induced proliferation, migration and invasion of PDAC cells (*p* < .05, Figure 5B‒D), indicating that histone modification was involved in the pro‐tumour effects of KMT2B. MTA treatment also led to a reduction in the elevated levels of Akt and p‐Akt caused by KMT2B overexpression (Figure 5A). To identify the genes which were regulated by KMT2B via histone methylation, we performed ChIP‐seq using anti‐H3K4me3 to precipitate the DNA‒protein complexes. A total of 2862 differential peaks (FC ≥2, adjusted *p* < .0005) were identified between PANC‐1‐KMT2B and PANC‐1‐VC cells, of which 549 peaks, representing 537 genes, were enriched within a 1 kb symmetrical region around the transcriptional start sites (TSS). Majority of these genes (508/537) exhibited higher H3K4me3 peaks in PANC‐1‐KMT2B cells than in the control PANC‐1‐VC cells (Figure 5E). A Venn diagram analysis revealed nine genes that exhibited both elevated H3K4me3 ChIP‐seq signals above the average level and upregulated mRNA expression as a result of KMT2B overexpression in RNA‐seq (Figure 5F). We noted with interests that FYN, a non‐receptor tyrosine kinase involved in the PI3K/Akt signalling pathway, was among the list of these nine genes (Figure 5F,G). The significant enrichment of H3K4me3 in the promoter region of *FYN* was further validated by chromatin immunoprecipitation quantitative real‐time PCR (ChIP‐qPCR) with anti‐H3K4me3 as the capture antibody (Figure 5H). In addition to using the anti‐H3K4me3, we also conducted ChIP‐qPCR with a specific antibody against KMT2B to confirm the association between KMT2B and *FYN*. The results again showed an elevated level of KMT2B binding at the promoter region of *FYN* in PANC‐1‐KMT2B cell (*p* < .05, Figure 5I). The increased mRNA level of *FYN* in KMT2B‐overexpressed PDAC cells was not only revealed by RNA‐seq, but also validated by qPCR analysis (*p* < .05, Figure 5J). Moreover, the luciferase assay demonstrated that KMT2B overexpression significantly increased the transcriptional activity of the *FYN* promoter (*p* < .001, Figure 5K). In the orthotopic model, IHC analyses showed that FYN expression was significantly higher in KMT2B‐overexpressing pancreatic tumours compared to control tumours (*p* < .05, Figure 5L). Meanwhile, the increased FYN expression was associated with the upregulation of p‐Akt and total Akt in KMT2B‐overexpressing tumour tissues (Figures 5M and S6B). Clinically, the data from TCGA and GTEx clearly showed that the mRNA level of *FYN* was positively correlated with *KMT2B* (adjusted *p *< .001, *R* = 0.43, Figure 5N). The survival curves based on *FYN* expression (Figure S3D) displayed patterns similar to those observed for *KMT2B* in PDAC patients (Figure S3C). All these results collectively indicated that FYN is a downstream target of KMT2B.

To elucidate the role of FYN in KMT2B‐induced PDAC progression, we knocked down *FYN* in PANC‐1 cells (*p* < .05, Figure S8A,B). This resulted in a significant reduction in the proliferation, migration and invasion capabilities of the PDAC cells induced by KMT2B (*p* < .05, Figure 5O‒Q). Additionally, *FYN* knockdown attenuated the KMT2B‐induced increases in p‐Akt and total Akt, as well as the downstream‐regulated genes such as *MMP1* and *CCND1* (Figure 5R).

**FIGURE 6:**
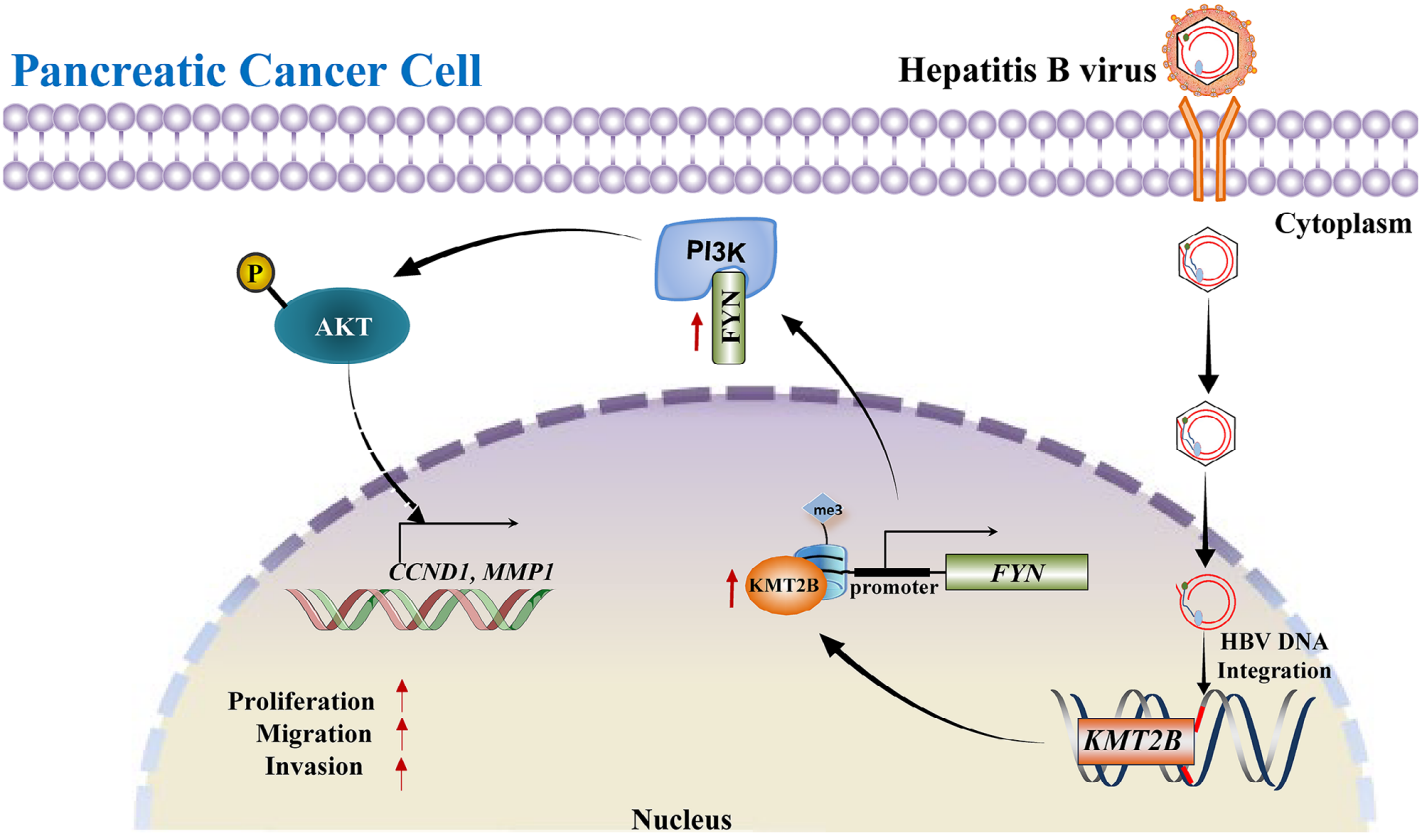
Schematic diagram illustrating the proposed mechanisms of the effects of the hepatitis B virus (HBV)‐integrated gene *KMT2B* on pancreatic ductal adenocarcinoma (PDAC). In pancreatic cancer cells, *KMT2B* gene was found to be targeted by HBV DNA. HBV integration in *KMT2B* induced the overexpression of KMT2B. Furthermore, KMT2B upregulation promoted FYN expression by maintaining the status of H3K4me3 at *FYN* promoter. Increased FYN expression activates the PI3K/Akt signalling and increased the expression of downstream genes such as *MMP1* and *CCND1*, resulting in the enhanced proliferation, migration and invasion of PDAC cells.

Taken together, we propose that HBV integration leads to aberrant KMT2B expression in PDAC, which results in the transcriptional activation of FYN through H3K4 trimethylation. The pro‐tumour effects of the KMT2B/FYN axis are mediated by the PI3K/Akt signalling pathway (Figure 6).

## 4 DISCUSSION

Although mounting evidence from epidemiology studies has suggested that chronic HBV infection is associated with PDAC,^4^, ^7^, ^13^, ^35^ the mechanism underlying this relationship remains unclear. We have reported before that HBV gene fragments, HBV replicative cccDNA and HBV‐specific antigens can all be detectable in PDAC specimens.^4^ In this study, we focused on HBV integration and successfully isolated 425 viral‒host junctions from PDAC and adjacent non‐tumour tissues. To our knowledge, this is the first study reporting the integration of HBV in pancreatic tissues.

HBV integration is a frequent event in HCC, intrahepatic cholangiocarcinoma (iCCA),^36^ non‐Hodgkin lymphoma (NHL)^37^ and gastric cancer.^38^ Here, we found that HBV integrants can be isolated in 72.70% of the HBV‐positive PDAC samples. This rate is lower than that in HCC (∼90%) but comparable to that in iCCA (41.70%‒70%) and NHL (50%). Compared with the cases in HCC, HBV integration in PDAC has some unique features. For instance, in HCC, the integration breakpoints are generally mapped to the 3′‐end of *HBx* gene which generates a C‐terminal truncated *HBx*. Differently, in PDAC, approximately 75% of the breakpoints located at the 3′‐end of HBV S gene (nt. 324–499), which phenomenon was also observed in NHL.^37^ This integration pattern resulted in a truncated form of HBsAg. Unlike the full‐length HBsAg, C‐terminal truncated middle S protein (MSt) has been reported to harbour transactivation activity,^39^ and may have oncogenic functions.^40^ Thus, it is speculated that the two different viral truncated proteins caused by HBV integration exerts their pro‐tumour abilities in a cell type‐dependent manner. While truncated X protein tends to promote cell growth in hepatocytes, the viral‒host chimeric DNA expressing truncated S protein may enhance the growth capability in pancreatic cells. Given that the majority of HCC cases with HBV integrations exhibit high expression levels of the S gene,^20^ it would be of interest in future studies to characterise the length of HBsAg and compare the MSt/S ratio in HBV‐integrated PDAC and HCC.

HBV integration was consistently present (100%) in well‐differentiated or moderately differentiated PDAC. Previously, we and other research groups have reported that HBV‐positive PDAC is associated with lower clinical stages and pathological grades.^4^, ^12^, ^41^, ^42^ Notably, a large‐scale clinical study found that chronic HBV infection was linked to significantly lower rates of both synchronous and metachronous metastases,^41^ as well as improved survival in patients with advanced PDAC compared to those without HBV infection.^12^, ^41^, ^42^ These findings suggest that PDAC associated with HBV infection or HBV DNA integration may exhibit relatively lower malignant potential than PDAC driven by other aetiologies.

We found that there is a comparable frequency of HBV integration in PDAC and adjacent pancreatic tissues. Indeed, multiple studies on HCC have also reported similar findings that HBV integration numbers per sample in non‐tumour liver tissues are comparable to^21^, ^43^ or even exceed those in tumour tissues.^20^, ^44^, ^45^ This phenomenon may be attributed to the fact that HBV DNA integration is an early event in HBV infection, occurring even prior to histologically detectable liver damage.^46^ Consequently, infected cells accumulate a substantial number of HBV integrations at the stage of cancer due to long‐standing chronic infection.^47^ Notably, while integration frequencies were comparable between PDAC and adjacent pancreatic tissues, their genomic distributions exhibited significant differences, particularly in chromosomes 2, 10, 11, 16 and X. Given that HBV‐induced genomic instability is a driver of hepatocarcinogenesis,^48^ we hypothesise that these distinct chromosomal integration patterns may contribute to the divergent cell fates between tumour and adjacent non‐tumour tissues.

On the arm of the host genome, some HBV integration hotspots were identified in PDAC and adjacent non‐tumour tissues. *LOC100507412*, a function unknown non‐coding RNA gene, was recurrently targeted by HBV DNA for 12 times in nine different tissue samples. For the remaining five HBV recurrent integration sites, four were identified from tumour tissues, suggesting a positive selection may occur during the development of PDAC. This notion is further supported by the fact that unlike the overall integration pattern where >50% breakpoints located in intergenic regions, HBV recurrent integration genes in PDAC or HBV co‐target genes shared in PDAC and HCC were all found to receive HBV DNA inside the genes. Enrichment of HBV integrations in gene elements increases the probability of insertional mutagenesis. Nevertheless, future research will employ CRISPR/Cas9 technology to generate site‐specific HBV knock‐in mouse models or cell lines, allowing us to accurately evaluate the contribution of HBV integration to PDAC initiation.

*KMT2B* is one of the most common HBV integration sites in HCC, with its frequency being second only to that of the *TERT* gene. HBV DNA integration into *KMT2B* is present in 7.70% of HCC tissues and 2.41% of non‐tumour tissues (OR, 3.39; *p* < .01),^49^ suggesting a positive selection of cells harbouring HBV integration in *KMT2B* gene during the development of HCC. HBV integration usually occurs in the third intron of *KMT2B*, resulting in increased expression of *KMT2B*.^20^, ^21^, ^50^ Here, we found that in one PDAC sample, an HBV integrant was also isolated from the third intron of *KMT2B*, and expression of *KMT2B* in that case was elevated. *KMT2B* gene encodes a histone methyltransferase responsible for trimethylation of H3K4, playing critical roles in the control of gene expression. KMT2B has been reported as a positive regulator of breast cancer,^51^ liver cancer^49^ and cervical cancer.^52^ This study is the first to provide in vitro, in vivo and clinical evidence supporting the involvement of KMT2B in PDAC progression.

As a member of the Src family protein kinases, *FYN* is a well‐defined cancer‐related gene that promotes cancer growth and metastasis through diverse biological functions, including cell growth, migration, apoptosis and epithelial‐to‐mesenchymal transition.^53^ FYN is highly expressed in many types of cancer such as glioblastoma, melanoma, squamous cell carcinoma, breast cancer, prostate cancer and PDAC.^53^ The intracellular level of FYN has been proposed to be regulated by transcription factors,^54^ miRNA^53^ and ubiquitinated degradation.^55^ Our results revealed H3K4 tri‐methylation as a novel paradigm for the transcriptional regulation of *FYN*. To date, only a few genes, namely, *c‐Myc* ^56^ and *EGF* ^52^ have been reported to be governed by *KMT2B*. Here, we found that overexpression of KMT2B induced enrichment of H3K4me3 at the *FYN* TSS and enhanced the transcription of *FYN* in PDAC cells. This finding opens up new avenues for the development of targeted therapy against FYN in cancers.

In conclusion, this study demonstrated a high prevalence of viral integration in HBV‐positive PDAC, shedding light on the causal relationship between HBV infection and PDAC. Our findings suggest that diligent monitoring for PDAC in HBV‐infected populations is necessary, and that prevention and intervention of HBV infection may be a practical strategy to reduce the incidence of PDAC in HBV‐endemic areas. By tracking HBV's preferential target genes, we identified *KMT2B* as a novel oncogene that exacerbates the malignant behaviour of PDAC through the KMT2B/FYN/PI3K/Akt axis. This finding provides new insights into the mechanisms of PDAC and reveals potential therapeutic targets.

## AUTHOR CONTRIBUTIONS

*Conception and design*: Yu Gan, Hong Tu and Mengge Li. *Methodology*: Mengge Li, Huimin Li, Dejun Liu, Hui Yuan, Yan Wu, Min Du, Yuan Fang, Jin Li, Hui Cong, Dan Zhao, Chunsun Fan, Qing Wang and Cenkai Shen. *Formal analysis*: Mengge Li and Huimin Li. *Investigation*: Huimin Li, Mengge Li, Hui Yuan, Yan Wu and Min Du. *Writing—original draft preparation*: Mengge Li, Huimin Li and Shunan Liu. *Resources*: Dejun Liu and Yongwei Sun. *Writing—review and editing and supervision*: Hong Tu and Yu Gan. *Funding acquisition*: Hong Tu, Yu Gan and Yongwei Sun.

## CONFLICT OF INTEREST STATEMENT

The authors declare they have no conflicts of interest.

## ETHICS STATEMENT

The collection of patients’ samples and preparation protocol were approved by the Renji Hospital Ethics Committee. The animal experiments were approved by the Animal Care and Use Committees of Shanghai Cancer Institute and performed in accordance with the ARRIVE guidelines.

## Supporting information



Supporting Information

Supporting Information

## Data Availability

HBV capture sequencing data were uploaded to the Genome Sequence Archive for Human (GSA‐human, https://ngdc.cncb.ac.cn/gsa‐human) database with accession number of HRA005175. The ChIP‐seq profiles of this study were uploaded to the NCBI database with an accession number as PRJNA985215. The remaining data are available within the article or Supporting Information.
