# Supplementary material for: Analysis of hepatitis B virus integration identifies KMT2B as a novel cancer‐related gene in pancreatic cancer
Source: Clin Transl Med. 2025 Jul 31;15(8):e70424. doi: 10.1002/ctm2.70424 (PMC12311841; doi:10.1002/ctm2.70424)
Supplement: Supplementary file 1 — Supporting Information [file CTM2-15-e70424-s002.pdf]

Supplementary Figures

Supplementary Figure 1

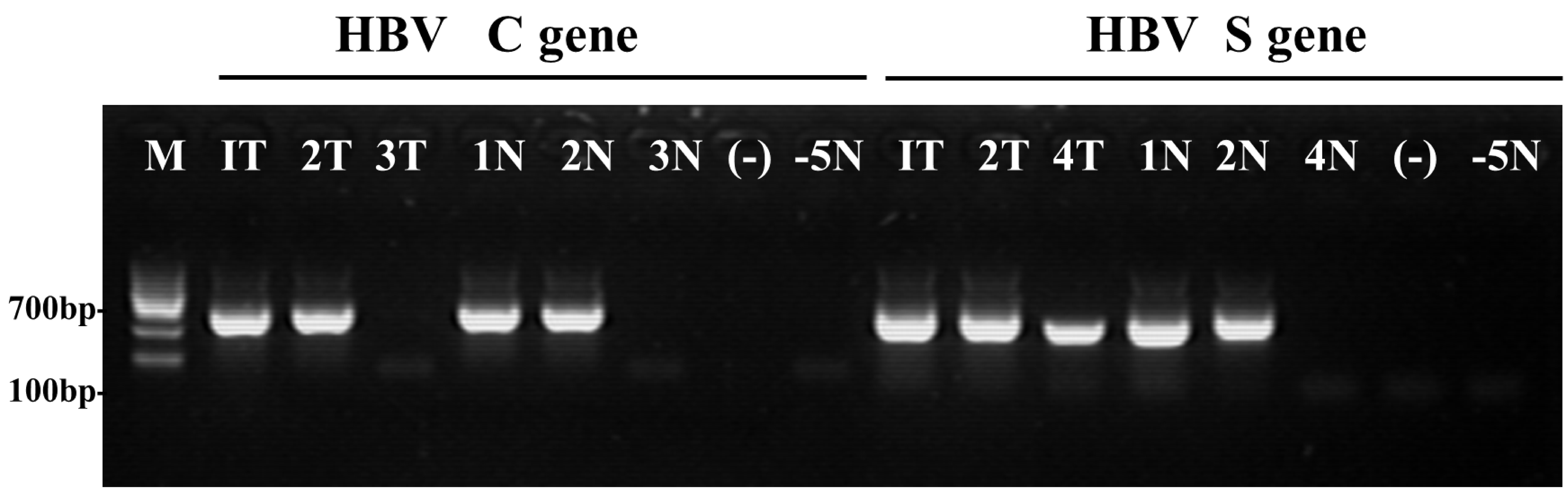

Supplementary Figure 1. PCR detection of HBV DNA in PDAC tumor and para-tumor tissues. Representative images of the PCR amplification of the HBV S gene and C gene in PDAC tumor and para-tumor tissues. The sample ID were marked above each lane in the gel. M: DNA marker, T: tumor, N: para-tumor, -: negative.

Supplementary Figure 2

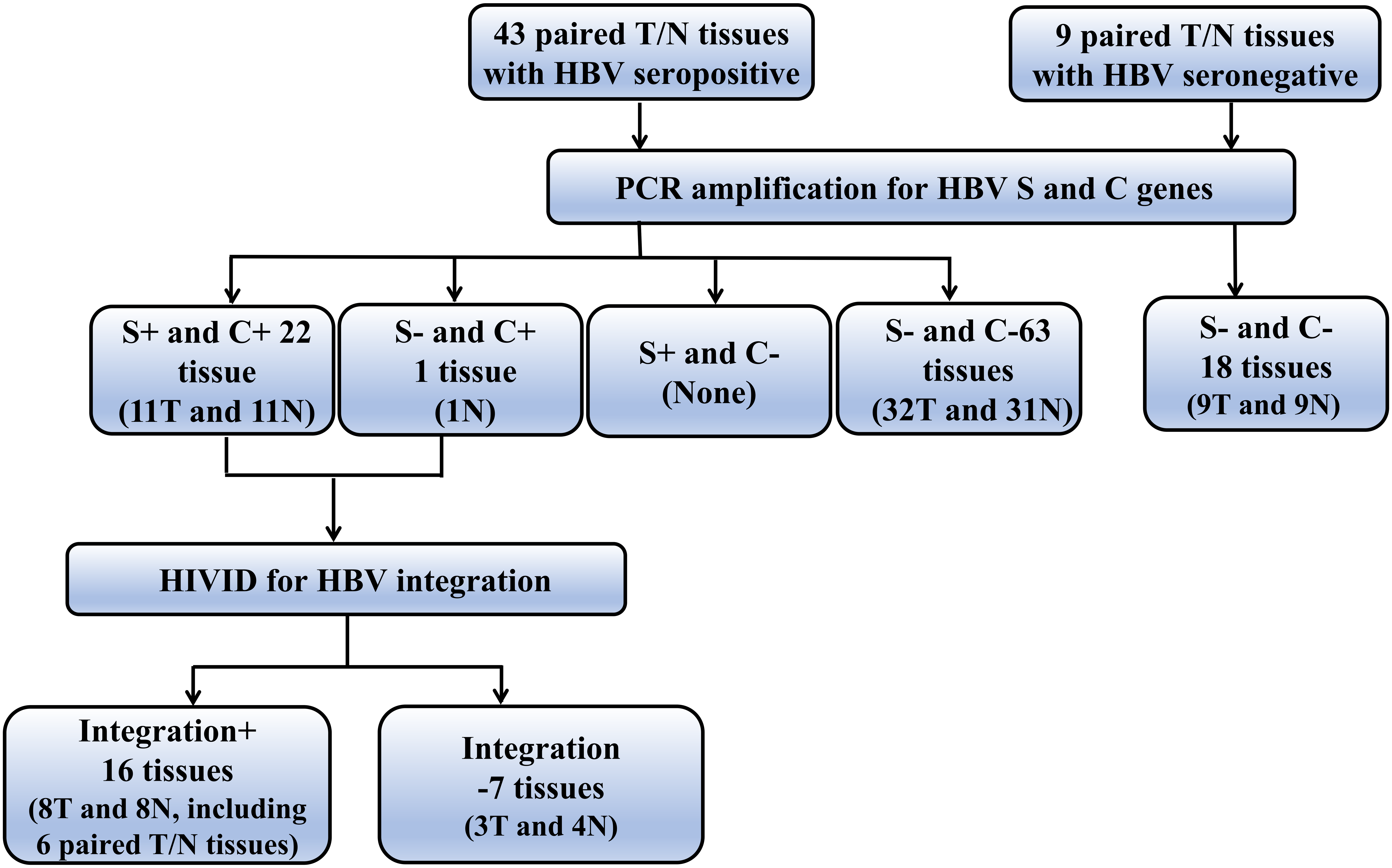

Supplementary Figure 2. Flowchart of the identification of HBV DNA integration in PDAC tumor and para-tumor tissues. T: tumor, N: non-tumor pancreatic tissue, S: HBV S gene, C: HBV C gene, +: positive, -: negative.

Supplementary Figure 3

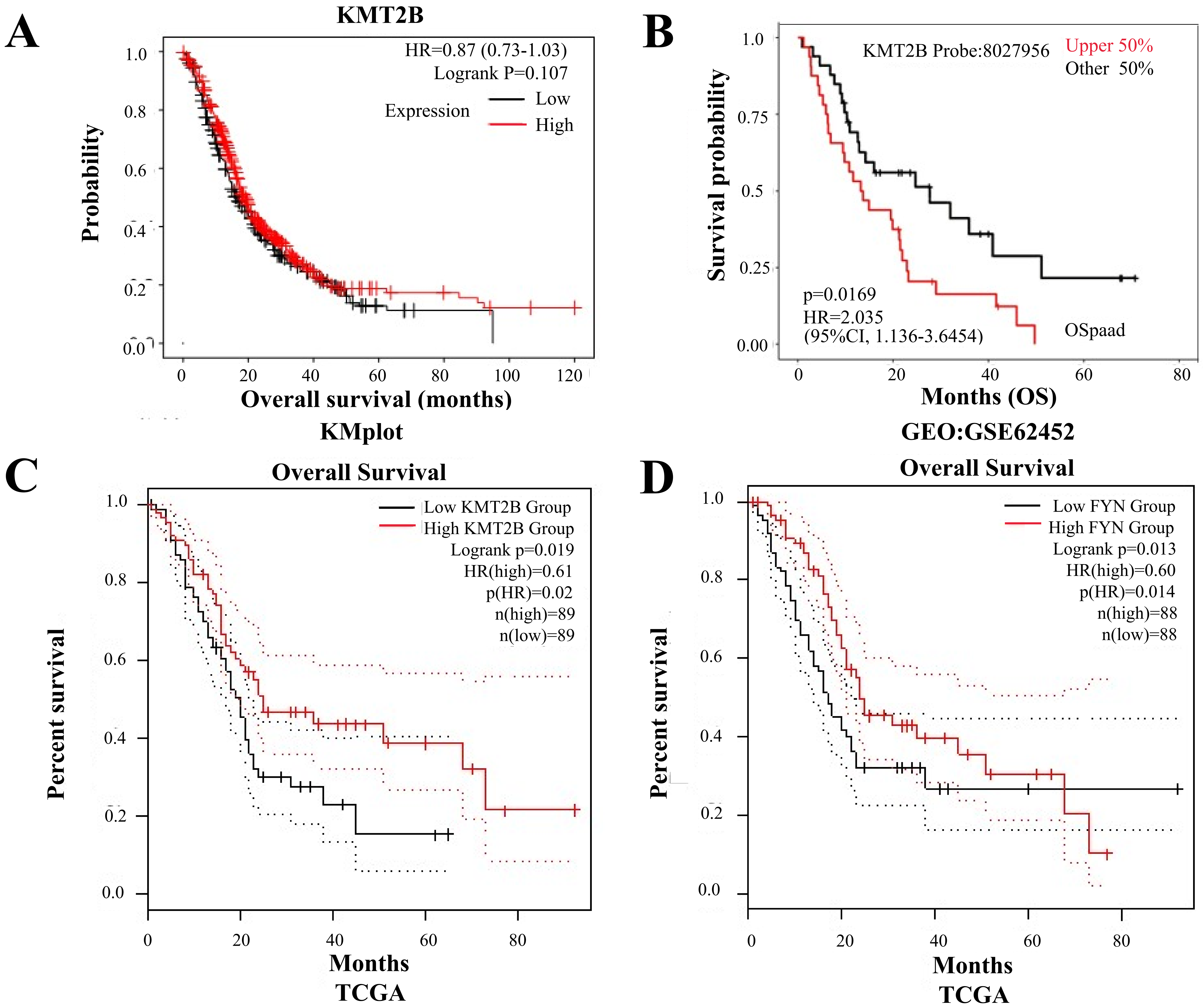

Supplementary Figure 3. Kaplan–Meier survival analysis of *KMT2B* expression in pancreatic cancer patients across different databases. (A) : Overall survival (OS) analysis of *KMT2B* in PDAC using the Kaplan–Meier Plotter database. Patients were divided into high and low *KMT2B* expression groups. No significant difference was observed. **(B):** OS analysis based on *KMT2B* from the GEO:GSE62452 database. Patients with higher *KMT2B* expression (upper 50%) showed significantly worse prognosis compared to the lower 50%. **(C):** Survival curves from TCGA dataset confirm the negative association between high *KMT2B* expression and overall survival in PDAC patients. **(D):** Survival curves from TCGA dataset confirm the negative association between high *FYN* expression and overall survival in PDAC patients. Solid lines represent survival curves, and dotted lines represent confidence intervals.

Supplementary Figure 4

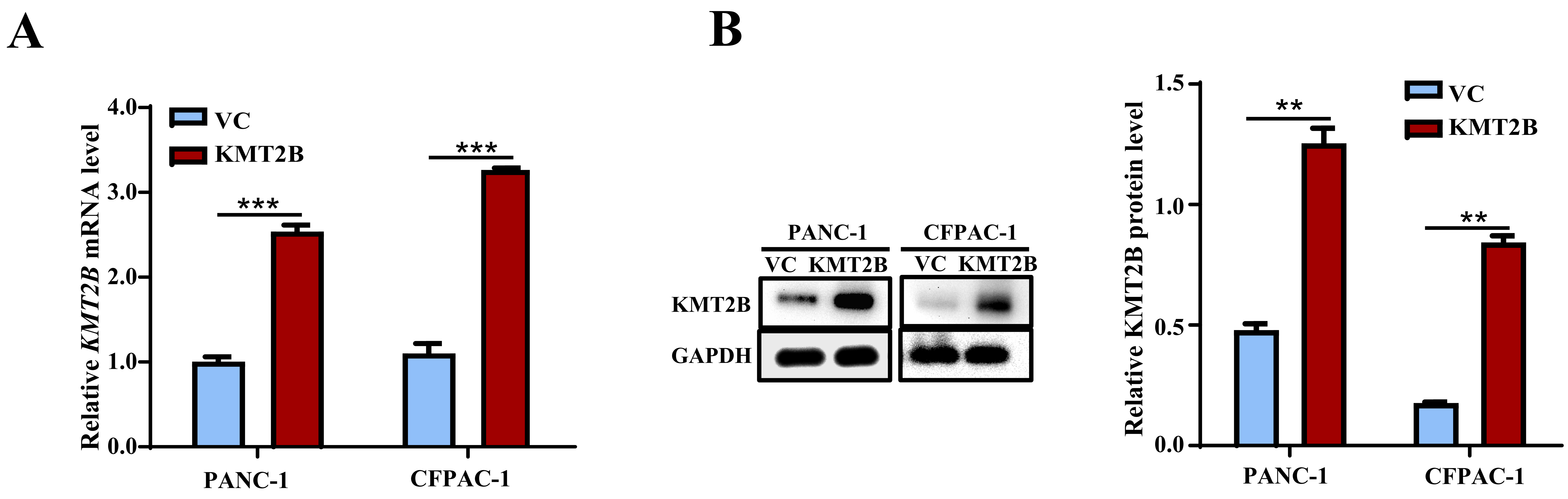

Supplementary Figure 4. The level of KMT2B in KMT2B-overexpressing PDAC cells. **(A):** The mRNA level of *KMT2B* in KMT2B-overexpressing PDAC cells (PANC-1-KMT2B or CFPAC-1-KMT2B) and control cells (PANC-1-VC or CFPAC-1-VC) revealed by qPCR. **(B):** The protein level of KMT2B in KMT2B-overexpressing PDAC cells and control cells detected by western blot. KMT2B was cleaved by Taspase 1 resulting in the generation of a C-terminal fragment of 80 kDa. Right: quantitative analysis of the protein level by ImageJ. *, $p < 0.05$; **, $p < 0.01$; ***, $p < 0.001$.

Supplementary Figure 5

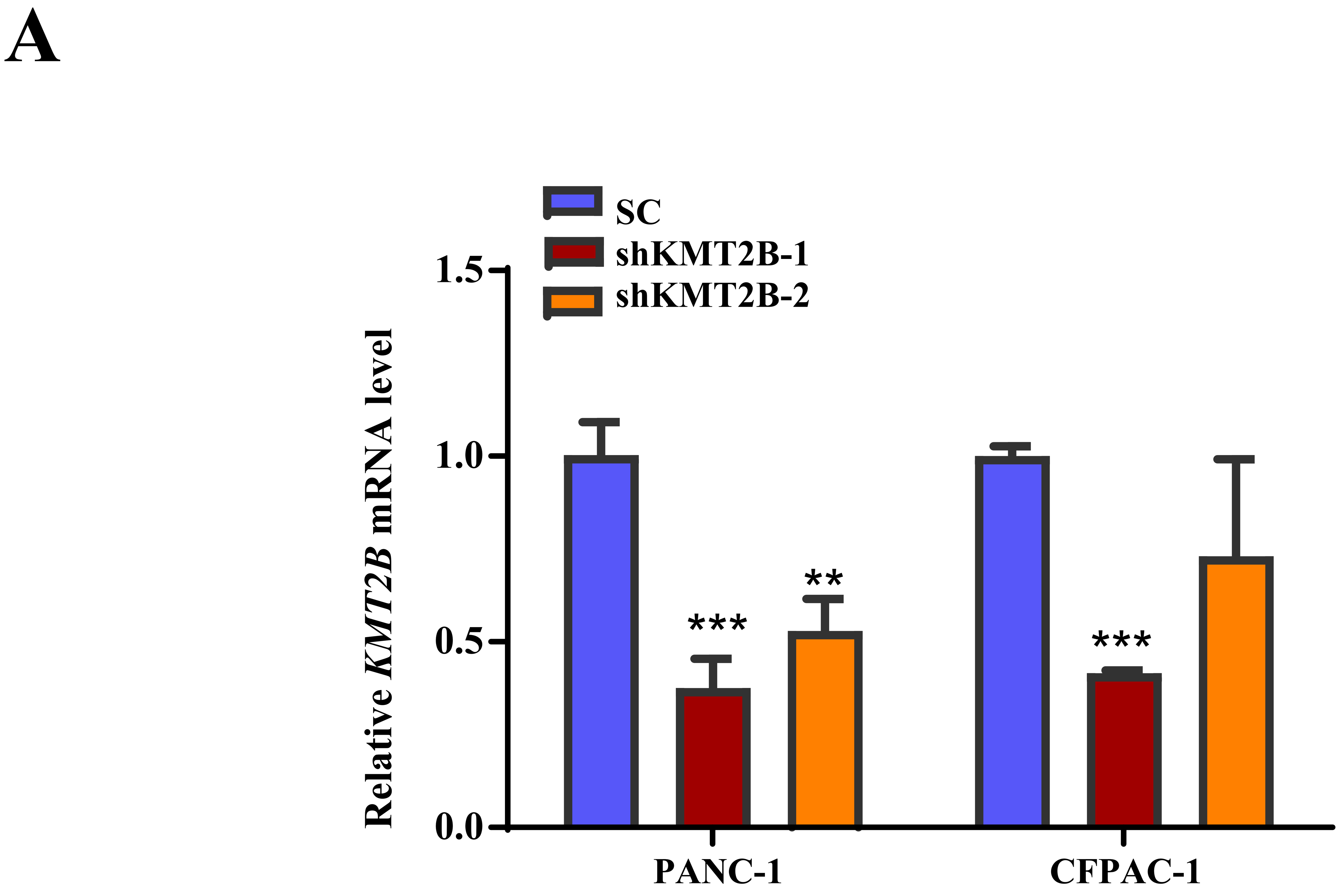

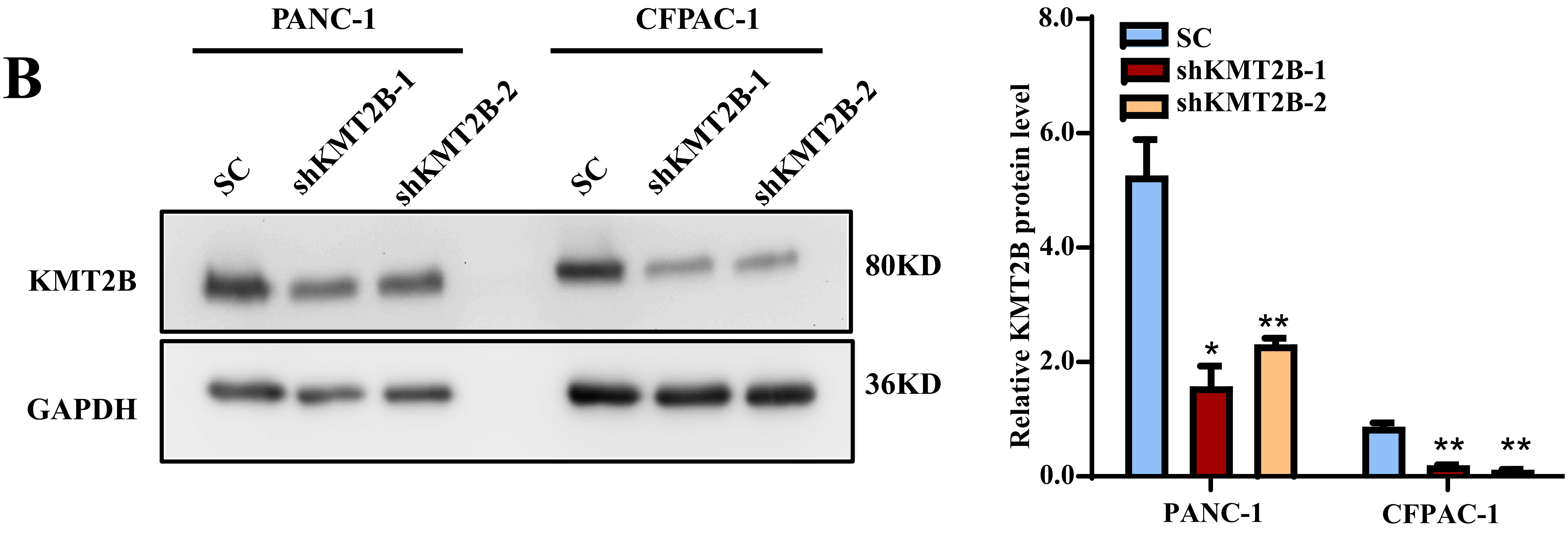

Supplementary Figure 5. The level of KMT2B in KMT2B-knockdown PDAC cells. (A): The mRNA level of *KMT2B* in KMT2B-knockdown PDAC cells (PANC-1-shKMT2B and CFPAC-1-shKMT2B) and control cells (PANC-1-SC or CFPAC-1-SC) revealed by qPCR. (B): The protein level of KMT2B in KMT2B-knockdown PDAC cells and control cells detected by western blot. Right: quantitative analysis of the protein level by ImageJ. *, $p < 0.05$; **, $p < 0.01$; ***, $p < 0.001$.

Supplementary Figure 6

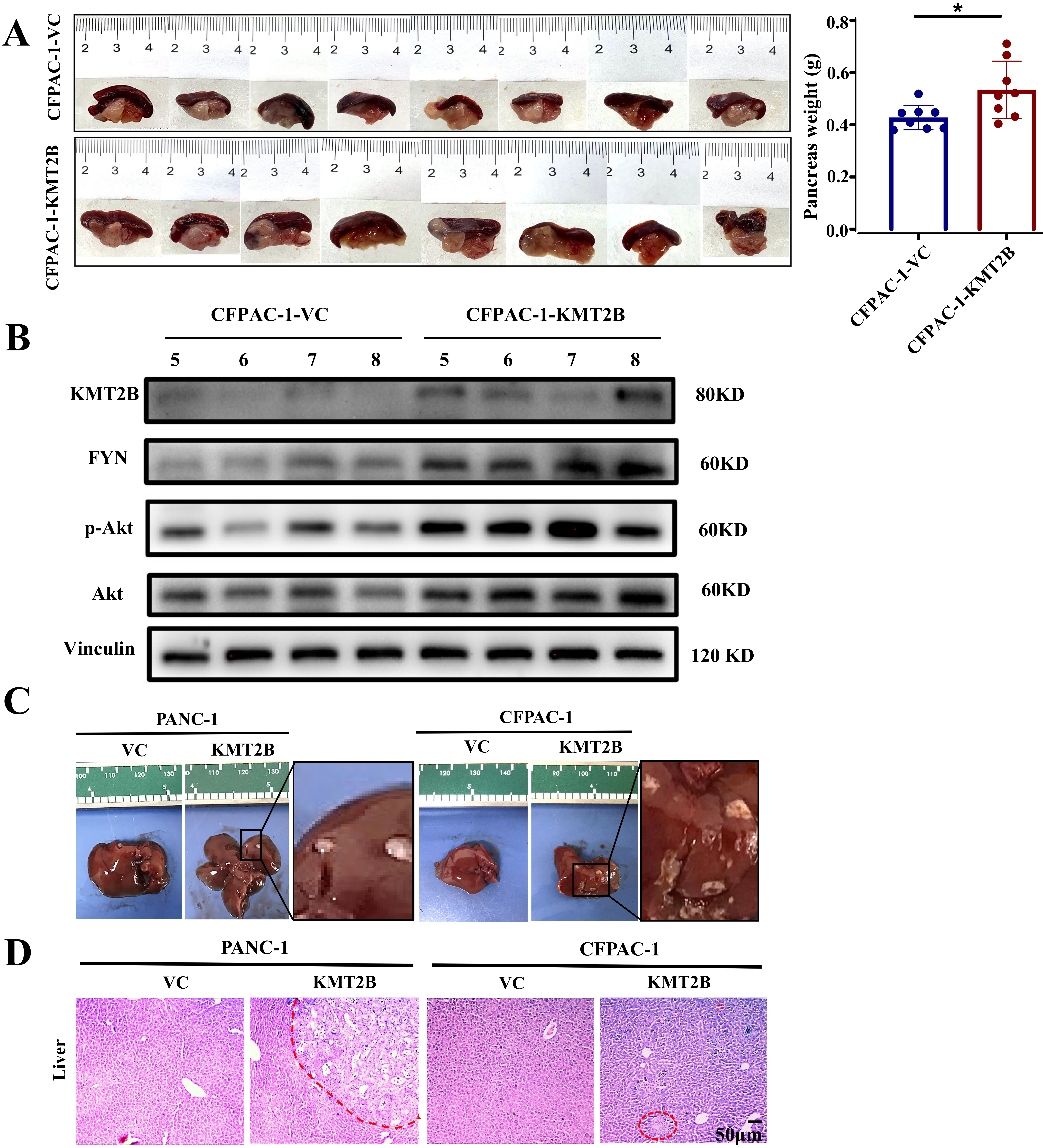

Supplementary Figure 6. The orthotopic tumor model formed by CFPAC-1-KMT2B and control CFPAC-1-VC cells in BALB/c nude mice (n = 8 per group). (A): The images of tumors at the time of sacrifice 31 days post tumor cells inoculation. Right: The tumor weights at the time of sacrifice. The data are presented as the mean ± SEM. **(B):** Western blot analysis of the protein levels of KMT2B, FYN, p-Akt and Akt in orthotopic tumor tissues. **(C):** Representative images of the livers with metastatic nodules collected from orthotopic pancreatic tumor-bearing mice of CFPAC-1 cell (n = 5 per group) and PANC-1 cell (n = 8 per group). Black rectangle highlights metastatic tumors. **(D):** Representative images of H&E-stained liver sections. The red dashed lines highlight metastatic lesions in the liver (Scale bar, 50 μm).*, $p < 0.05$; **, $p < 0.01$; ***, $p < 0.001$.

Supplementary Figure 7

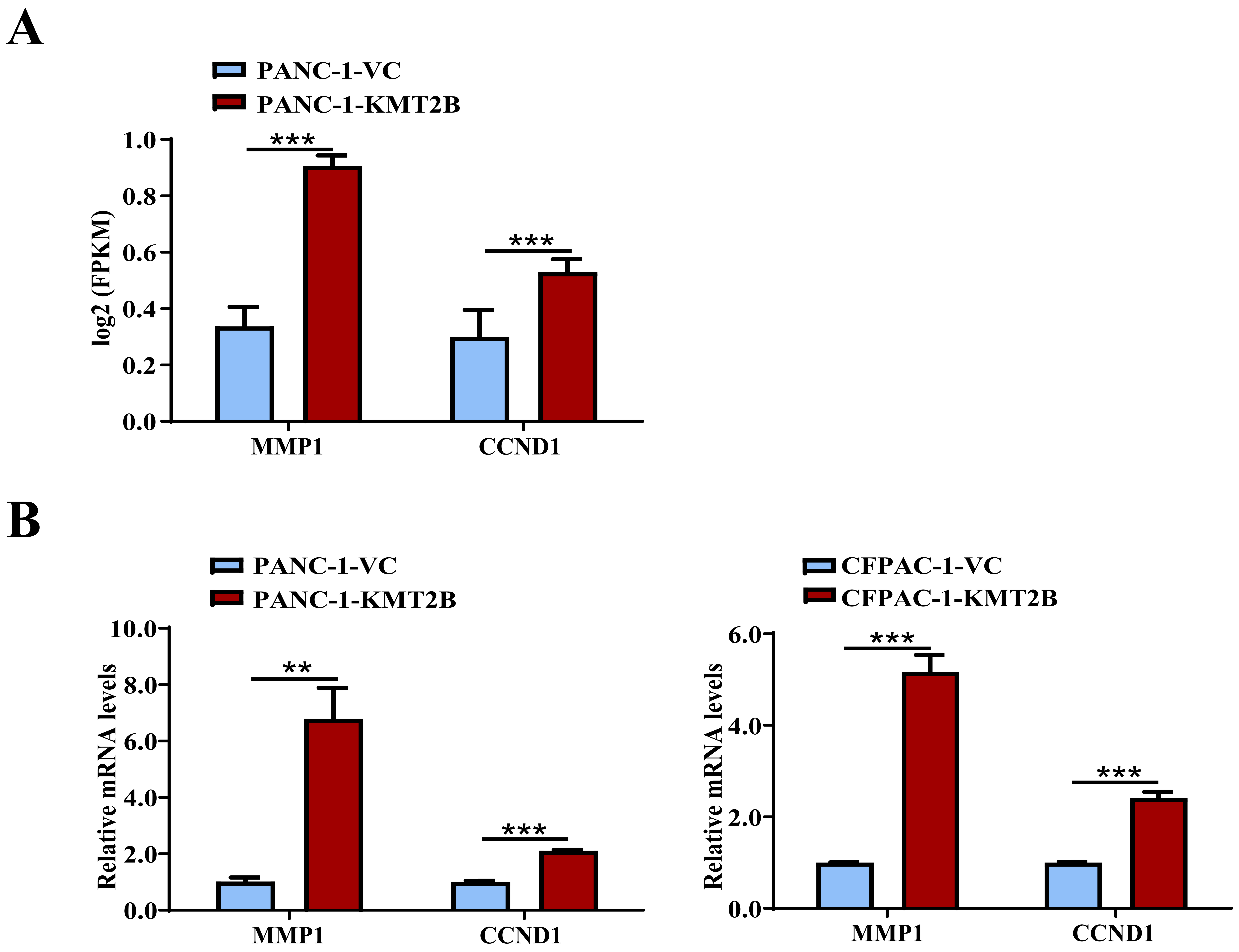

Supplementary Figure 7. The expression of *MMP1* and *CCND1* in KMT2B-overexpressing PDAC cells. (A): The FPKM value of *MMP1* and *CCND1* in KMT2B-overexpressing cell (PANC-1-KMT2B) and the control cell (PANC-1-VC) determined by RNA-seq. **(B):** The mRNA level of *MMP1* and *CCND1* in KMT2B-overexpressing PDAC cells (PANC-1-KMT2B or CFPAC-1-KMT2B) and control cells (PANC-1-VC or CFPAC-1-VC) revealed by qPCR. *, $p < 0.05$; **, $p < 0.01$; ***, $p < 0.001$.

Supplementary Figure 8

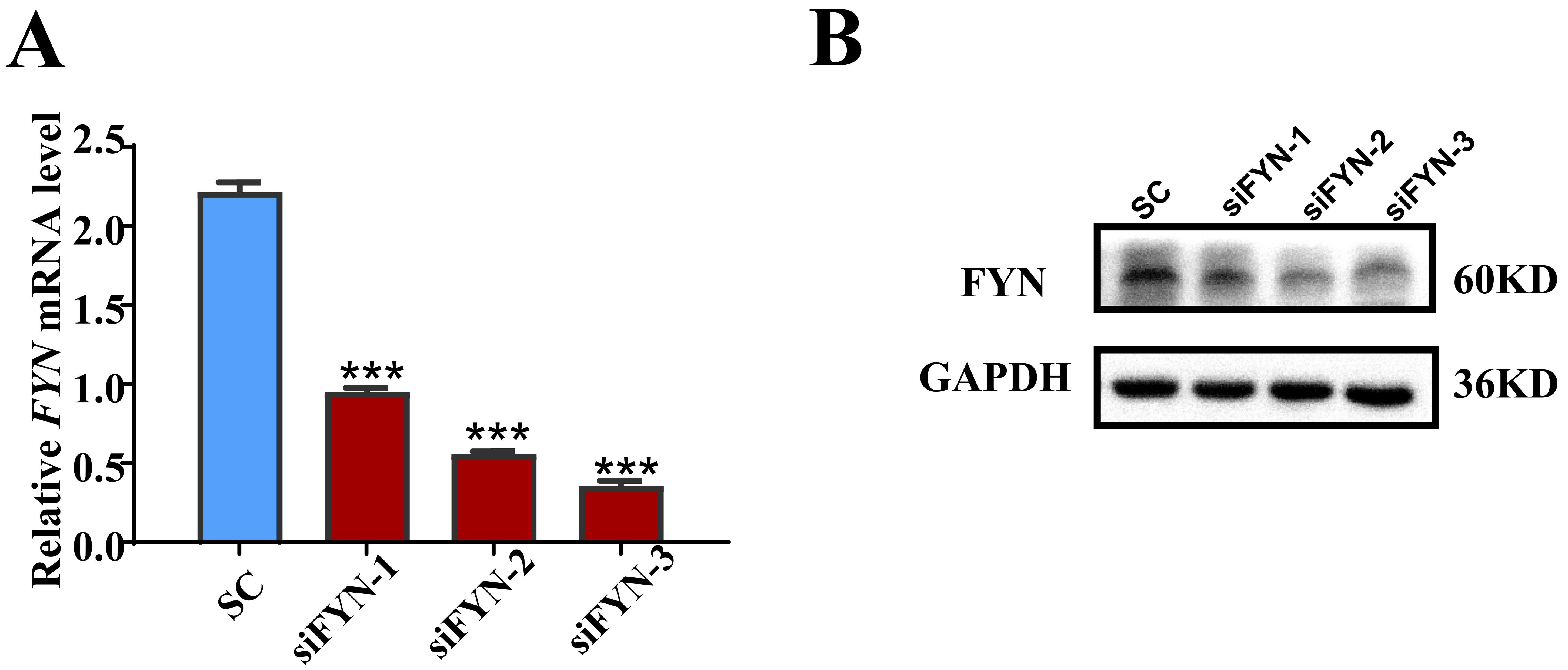

Supplementary Figure 8. The FYN level in PANC-1-KMT2B cells with FYN knockdown. (A): The mRNA level of *FYN* in PANC-1-KMT2B cell and PANC-1-VC cell with or without *FYN* knockdown revealed by qPCR. **(B):** The protein level of FYN in PANC-1-KMT2B cell and PANC-1-VC cell with or without FYN knockdown detected by western blot. *, $p < 0.05$; **, $p < 0.01$; ***, $p < 0.001$.
