# Supplementary material for: Analysis of hepatitis B virus integration identifies KMT2B as a novel cancer‐related gene in pancreatic cancer
Source: Clin Transl Med. 2025 Jul 31;15(8):e70424. doi: 10.1002/ctm2.70424 (PMC12311841; doi:10.1002/ctm2.70424)
Supplement: Supplementary file 2 — Supporting Information [file CTM2-15-e70424-s001.pdf]

Supplementary Table S1. Sequences of scramble control and shRNA targeted the gene *KMT2B* that used in this study.

Name	Nucleotide sequence
**shKMT2B-1**	5-CCTTCTCTTCTCCCTCCTTGT-3
**shKMT2B-2**	5-GCATCTTCCAGGATGACAAGA-3
**Scramble control**	5-CGTTTGTCCCTCCAGCATCT-3

Supplementary Table S2. Sequences of siRNA targeted the gene *FYN* that used in this study.

Name	Nucleotide sequence
**siFYN-1**	GGTGGATACTACATTACCA
**siFYN-2**	GAGACCATGTCAAACATTA
**siFYN-3**	GTGAACTCTTCGTCTCATA

Supplementary Table S3. RT-qPCR primer sequences used in this study.

Gene		Nucleotide sequence
KMT2B	Forward	5-CCGAGTCGAGGCTGCGTG-3
	Reverse	5-CTCGCTGGGATCGGAGCG-3
ACTIN	Forward	5-TGACGTGGACATCCGCAAAG-3
	Reverse	5-CTGGAAGGTGGACAGCGAGG-3
FYN	Forward	5-CTCAGCACTACCCCAGCTTC-3
	Reverse	5-ATCTCCTTCCGAGCTGTTCA-3
CCND1	Forward	5-ATGCCAACCTCCTCAACGAC-3
	Reverse	5-TCTGTTCCTCGCAGACCTCC-3
MMP1	Forward	5-GGTCTCTGAGGGTCAAGCAG-3
	Reverse	5-AGTTCATGAGCTGCAACACG-3

Supplementary Table S4. CHIP-qPCR primer sequences used in this study.

Gene		Nucleotide sequence
**FYN-chip**	Forward	5-GCACAACAACCTCGCCTCTA-3
	Reverse	5-GGCAGAGCATCAGCAAGAGT-3

Supplementary Table S5. The clinicopathological and laboratory features of pancreatic cancer patients for HBV DNA detection.

Sample ID	Gender	Age	Location	HBV marker					HBV DNA		HBV Genotype	Integration No.
				HBsAg	anti-HBs	HBeAg	anti-Hbe	anti-HBc	C gene	S gene		
11-1T	male	75	head	-	-	-	-	+	-	-	/	/
11-1N									-	-	/	/
12-1T	female	57	tail	-	+	-	+	-	-	-	/	/
12-1N									-	-	/	/
16-1T	male	59	head	-	+	-	-	+	-	-	/	/
16-1N									-	-	/	/
17-1T	female	74	head	-	+	-	-	+	-	-	/	/
17-1N									-	-	/	/
19T	male	51	body	-	+	-	-	+	-	-	/	/
19N									-	-	/	/
22T	male	60	body	-	-	-	-	+	-	-	/	/
22N									-	-	/	/
23T	male	52	tail	-	-	-	-	+	-	-	/	/
23N									-	-	/	/
24T	female	64	head	-	+	-	-	-	-	-	/	/
24N									-	-	/	/
25T	female	90	head	-	-	-	-	+	-	-	/	/
25N									-	-	/	/
26T	female	63	tail	-	-	-	-	+	-	-	/	/
26N									-	-	/	/
27T	male	72	head	-	+	-	-	+	+	+	C	13
27N									+	+	C	1
28T	female	59	tail	-	+	-	-	+	-	-	/	/
28N									-	-	/	/
29T	female	52	head	-	+	-	-	+	-	-	/	/
29N									-	-	/	/
30T	male	60	head	-	-	-	-	+	-	-	/	/
30N									-	-	/	/
31T	male	65	head	-	+	-	-	+	-	-	/	/
31N									-	-	/	/
32T	male	82	head	-	+	-	-	+	+	+	C	0
32N									+	+	C	0
33T	male	55	head	-	-	-	-	+	-	-	/	/
33N									-	-	/	/
34T	male	53	head	-	+	-	+	+	+	+	C	0
34N									+	+	C	0
35T	female	56	head	-	+	-	+	+	-	-	/	/
35N									-	-	/	/
36T	male	57	tail	+	-	-	+	+	+	+	B	0
36N									+	+	B	0
37T	female	70	head	-	+	-	+	+	-	-	/	/
37N									-	-	/	/
38T	female	69	head	+	-	-	+	+	+	+	B	8
38N									+	+	B	2

Supplementary Table S5 Continued

Sample ID	Gender	Age	Location	HBV marker					HBV DNA		HBV Genotype	Integration No.
				HBsAg	anti-HBs	HBeAg	anti-Hbe	anti-HBc	C gene	S gene		
39T	female	85	head	-	+	-	-	-	-	-	/	/
39N									-	-	/	/
40T	female	79	tail	-	+	-	-	+	-	-	/	/
40N									-	-	/	/
41T	male	67	head	-	+	-	-	+	-	-	/	/
41N									-	-	/	/
42T	female	68	tail	-	+	-	+	+	-	-	/	/
42N									-	-	/	/
1T	male	63	head	+	-	-	+	+	+	+	B	44
1N									+	+	B	23
2T	male	46	tail	+	-	-	+	+	+	+	B	45
2N									+	+	B	38
3T	female	60	tail	-	+	-	+	+	-	-	/	/
3N									-	-	/	/
4T	male	71	head	+	-	-	+	+	+	+	B	26
4N									+	-	B	31
5T	female	77	head	-	+	-	+	+	-	-	/	/
5N									-	-	/	/
6T	female	65	head	-	+	-	+	+	-	-	/	/
6N									-	-	/	/
7T	male	42	head	+	-	-	+	+	+	+	C	40
7N									-	-	/	/
8T	female	77	tail	+	-	-	+	+	-	-	/	/
8N									+	+	C	51
9T	male	83	head	-	+	-	+	+	-	-	/	/
9N									-	-	/	/
10T	female	75	tail	-	+	-	-	+	+	+	C	19
10N									+	+	C	0
11T	male	67	head	-	+	-	-	+	-	-	/	/
11N									-	-	/	/
12T	male	71	tail	-	+	-	-	+	-	-	/	/
12N									-	-	/	/
13T	female	69	head	-	+	-	-	+	-	-	/	/
13N									-	-	/	/
14T	female	91	head	-	+	-	-	+	-	-	/	/
14N									-	-	/	/
15T	male	71	tail	-	+	-	-	+	-	-	/	/
15N									-	-	/	/
16T	female	74	head	-	+	-	-	+	-	-	/	/
16N									+	+	C	23
17T	female	74	tail	-	-	-	+	+	+	+	C	28
17N									+	+	C	33

T for tumor; N for non-tumor; / for no test.

Supplementary Table S6. All HBV breakpoints in PDAC tissues and para-tumor tissues.

Sample ID	Left_chr	Left_pos	Left_strand	Right_chr	Right_pos	Right_strand	Gene element	Gene (distance)
1T	chr1	172668477	+	hbv	429	+	intergenic	FASLG(dist=32465),TNFSF18(dist=341883)
1T	chr1	245119370	+	hbv	1929	+	intergenic	HNRNPU(dist=91543),LOC101928068(dist=9136)
1T	chr1	242568032	-	hbv	418	+	intronic	PLD5
1T	chr10	26588005	+	hbv	2438	+	intronic	GAD2
1T	chr10	42400812	+	hbv	420	+	intergenic	NONE(dist=NONE),LOC441666(dist=426502)
1T	chr10	87731281	+	hbv	2222	-	intronic	GRID1
1T	chr10	19610052	-	hbv	459	+	intronic	MALRD1
1T	chr11	68020811	+	hbv	424	+	intergenic	SUV420H1(dist=39572),C11orf24(dist=7992)
1T	chr12	57890181	+	hbv	428	+	intronic	MARS
1T	chr12	88677967	+	hbv	2167	-	intergenic	TMTC3(dist=84303),KITLG(dist=208603)
1T	chr13	111091467	+	hbv	1439	-	intronic	COL4A2
1T	chr15	33987439	-	hbv	457	+	intronic	RYR3
1T	chr16	55018199	+	hbv	1987	+	intergenic	IRX5(dist=49804),IRX6(dist=340272)
1T	chr16	46400316	+	hbv	394	-	intergenic	NONE(dist=NONE),ANKRD26P1(dist=102933)
1T	chr17	64920649	+	hbv	1917	+	intergenic	CACNG5(dist=39254),CACNG4(dist=40331)
1T	chr18	47622971	+	hbv	446	-	intronic	MYO5B
1T	chr19	5394262	-	hbv	421	+	intergenic	PTPRS(dist=53448),ZNRF4(dist=61164)
1T	chr19	46463460	-	hbv	428	+	intronic	NOVA2
1T	chr2	176098948	+	hbv	423	+	intergenic	ATP5G3(dist=52458),KIAA1715(dist=691462)
1T	chr2	56268500	-	hbv	421	+	intergenic	MIR216B(dist=40570),LOC100129434(dist=132169)
1T	chr2	200467053	-	hbv	1827	+	intergenic	SATB2-AS1(dist=129572),LOC101927641(dist=5738)
1T	chr2	218138415	-	hbv	2000	+	intergenic	TNP1(dist=413633),DIRC3(dist=10331)
1T	chr2	242602867	-	hbv	2219	+	intronic	ATG4B
1T	chr20	1639113	+	hbv	1625	-	upstream	SIRPG
1T	chr20	18638570	+	hbv	1729	-	intronic	DTD1
1T	chr22	36629726	+	hbv	1827	+	intronic	APOL2
1T	chr22	45816532	+	hbv	426	+	intronic	RIBC2
1T	chr3	40650785	+	hbv	470	-	intergenic	ZNF621(dist=69500),CTNNB1(dist=590157)
1T	chr3	61928789	+	hbv	447	-	intronic	PTPRG
1T	chr5	161501745	+	hbv	459	+	intronic	GABRG2

Supplementary Table S6 Continued

Sample ID	Left_chr	Left_pos	Left_strand	Right_chr	Right_pos	Right_strand	Gene element	Gene (distance)
1T	chr6	77822920	-	hbv	427	+	intergenic	IMPG1(dist=1040525),HTR1B(dist=349028)
1T	chr7	81938917	+	hbv	2145	-	intronic	CACNA2D1
1T	chr8	40905236	-	hbv	457	+	intergenic	ZMAT4(dist=149893),SFRP1(dist=214240)
1T	chr8	116609085	-	hbv	458	+	intronic	TRPS1
1T	chr9	101825325	+	hbv	3110	+	intronic	COL15A1
1T	chrUn_gl000220	132883	+	hbv	424	+	intergenic	LOC100507412(dist=6187),RNA5-8S5(dist=23114)
1T	chrX	105942334	+	hbv	1837	+	intronic	RNF128
1T	hbv	1713	+	chr12	118852704	+	UTR3	SUDS3
1T	hbv	446	+	chr16	58115715	+	intergenic	MMP15(dist=34911),CFAP20(dist=31782)
1T	hbv	446	+	chr2	194659676	+	intergenic	PCGEM1(dist=1018051),LOC101927406(dist=549317)
1T	hbv	1854	+	chr2	22752005	+	intergenic	LOC645949(dist=818481),LOC102723362(dist=7346)
1T	hbv	443	+	chr4	140378214	+	intronic	RAB33B
1T	hbv	2233	+	chr6	16698795	+	intronic	ATXN1
1T	hbv	2453	+	chr8	20729546	+	intergenic	LZTS1-AS1(dist=581577),LOC286114(dist=101951)
2T	chr1	172751193	+	hbv	428	+	intergenic	FASLG(dist=115181),TNFSF18(dist=259167)
2T	chr1	228545257	+	hbv	420	+	intronic	OBSCN
2T	chr1	56468064	+	hbv	397	-	intergenic	MIR4422(dist=776668),PPAP2B(dist=492355)
2T	chr1	17947188	-	hbv	3084	+	intronic	ARHGEF10L
2T	chr1	49697473	-	hbv	419	+	intronic	AGBL4
2T	chr1	181312605	-	hbv	430	+	intergenic	GM140(dist=104865),CACNA1E(dist=140081)
2T	chr10	15752351	+	hbv	422	+	intronic	ITGA8
2T	chr10	84280865	+	hbv	423	+	intronic	NRG3
2T	chr11	64298618	+	hbv	507	-	intergenic	LOC100996455(dist=79490),SLC22A11(dist=24480)
2T	chr13	88803211	+	hbv	501	+	intergenic	LINC00397(dist=340436),LINC00433(dist=389873)
2T	chr14	69237614	-	hbv	423	+	intergenic	RAD51B(dist=174876),ZFP36L1(dist=16758)
2T	chr14	99416552	-	hbv	1833	+	intergenic	C14orf177(dist=232449),BCL11B(dist=219073)
2T	chr15	78314002	+	hbv	422	+	intronic	TBC1D2B
2T	chr16	82758785	+	hbv	394	-	intronic	CDH13
2T	chr17	45734220	-	hbv	529	+	intronic	KPNB1
2T	chr18	1503252	-	hbv	457	+	intergenic	LINC00470(dist=143622),METTL4(dist=1034272)
2T	chr19	7008323	+	hbv	429	+	intergenic	FLJ25758(dist=1897),MBD3L5(dist=22271)
2T	chr19	53296360	+	hbv	1	+	intergenic	ZNF600(dist=6326),ZNF28(dist=4301)
2T	chr19	12332626	+	hbv	446	-	intergenic	LOC100289333(dist=14235),ZNF44(dist=49999)

Supplementary Table S6 Continued

Sample ID	Left_chr	Left_pos	Left_strand	Right_chr	Right_pos	Right_strand	Gene element	Gene (distance)
2T	chr2	211807629	+	hbv	444	-	intergenic	CPS1(dist=263798),ERBB4(dist=432813)
2T	chr20	7344976	+	hbv	1871	+	ncRNA_intronic	LOC101929312
2T	chr3	32504697	+	hbv	426	+	intergenic	CMTM7(dist=8364),CMTM6(dist=18107)
2T	chr3	37752855	+	hbv	425	+	intronic	ITGA9
2T	chr3	176474956	+	hbv	396	-	intergenic	LINC01208(dist=121636),LINC01209(dist=56988)
2T	chr4	119214755	+	hbv	409	+	intronic	PRSS12
2T	chr4	32803660	+	hbv	2070	-	intergenic	PCDH7(dist=1655237),LOC101928622(dist=1094301)
2T	chr4	40794981	+	hbv	678	-	intronic	NSUN7
2T	chr4	190078828	+	hbv	447	-	intergenic	LINC01060(dist=555766),LINC01262(dist=501932)
2T	chr4	137807227	-	hbv	458	+	intergenic	LINC00613(dist=972392),PCDH18(dist=632846)
2T	chr4	186160403	-	hbv	427	+	intronic	SNX25
2T	chr5	58223885	+	hbv	420	+	intergenic	RAB3C(dist=76479),PDE4D(dist=40981)
2T	chr5	54637181	+	hbv	405	-	intronic	SKIV2L2
2T	chr5	89061569	+	hbv	446	-	intergenic	MEF2C-AS1(dist=733376),MIR3660(dist=250869)
2T	chr5	132057432	+	hbv	394	-	intronic	KIF3A
2T	chr5	157404820	+	hbv	446	-	intergenic	CLINT1(dist=118637),LOC101927697(dist=342892)
2T	chr7	8108459	-	hbv	429	+	intronic	GLCCI1
2T	chr8	84907867	+	hbv	497	-	intergenic	LINC01419(dist=586735),RALYL(dist=187586)
2T	chrUn_gl000220	131665	+	hbv	427	+	intergenic	LOC100507412(dist=4969),RNA5-8S5(dist=24332)
2T	chrX	20914893	+	hbv	429	+	intergenic	RPS6KA3(dist=630143),CNKSR2(dist=477643)
2T	hbv	2245	+	chr1	70087972	+	intergenic	DEPDC1-AS1(dist=1083662),LRRC7(dist=137886)
2T	hbv	445	+	chr11	58958500	+	intronic	DTX4
2T	hbv	446	+	chr11	113455807	+	intergenic	DRD2(dist=109806),TMPRSS5(dist=102461)
2T	hbv	1819	+	chr2	96739575	+	intergenic	GPAT2(dist=38848),ADRA2B(dist=39048)
2T	hbv	446	+	chr20	4619683	+	intergenic	ADRA1D(dist=390024),PRNP(dist=47114)
2T	hbv	1599	+	chr6	132336767	+	intergenic	CTGF(dist=64249),LINC01013(dist=118351)
4T	chr1	228823218	+	hbv	1828	+	intergenic	DUSP5P1(dist=35059),RHOU(dist=47606)
4T	chr1	81553616	+	hbv	414	-	intergenic	NONE(dist=NONE),LPHN2(dist=611839)
4T	chr1	91722679	+	hbv	394	-	intergenic	ZNF644(dist=234867),HFM1(dist=3644)
4T	chr11	30473612	-	hbv	1887	+	intronic	MPPED2
4T	chr12	82779603	+	hbv	421	+	intronic	METTL25
4T	chr12	130973450	-	hbv	428	+	intronic	RIMBP2
4T	chr14	32376774	-	hbv	422	+	intergenic	NUBPL(dist=46345),ARHGAP5-AS1(dist=167851)

Supplementary Table S6 Continued

Sample ID	Left_chr	Left_pos	Left_strand	Right_chr	Right_pos	Right_strand	Gene element	Gene (distance)
4T	chr14	46834580	-	hbv	415	+	ncRNA_intronic	LINC00871
4T	chr15	41043237	+	hbv	420	+	intronic	RMDN3
4T	chr17	7800379	+	hbv	1820	+	intronic	CHD3
4T	chr18	32455627	-	hbv	457	+	intronic	DTNA
4T	chr2	35670064	+	hbv	446	-	intergenic	MYADML(dist=1716780),LOC100288911(dist=911828)
4T	chr2	153513921	-	hbv	423	+	intronic	PRPF40A
4T	chr4	122790357	+	hbv	284	-	intronic	BBS7
4T	chr5	150719599	-	hbv	428	+	intronic	SLC36A2
4T	chr6	159524649	+	hbv	1829	+	intergenic	TAGAP(dist=58465),LOC101929122(dist=62282)
4T	chr7	61969534	+	hbv	419	+	intergenic	NONE(dist=NONE),ZNF733P(dist=782136)
4T	chr7	153165578	+	hbv	424	+	intergenic	LINC01287(dist=56259),DPP6(dist=418604)
4T	chr7	29421071	+	hbv	396	-	intronic	CHN2
4T	chrUn_gl000220	131834	+	hbv	423	+	intergenic	LOC100507412(dist=5138),RNA5-8S5(dist=24163)
4T	chrUn_gl000220	131880	+	hbv	427	+	intergenic	LOC100507412(dist=5184),RNA5-8S5(dist=24117)
4T	hbv	1939	+	chr11	109901660	+	intergenic	C11orf87(dist=601767),ZC3H12C(dist=62427)
4T	hbv	1814	+	chr16	52615211	+	ncRNA_intronic	CASC16
4T	hbv	471	+	chr2	194166174	+	intergenic	PCGEM1(dist=524549),LOC101927406(dist=1042819)
4T	hbv	444	+	chr5	114439618	+	intergenic	KCNN2(dist=607421),TRIM36(dist=20841)
4T	hbv	445	+	chr6	66189550	+	intronic	EYS
7T	chr1	89438388	-	hbv	428	+	intronic	CCBL2
7T	chr11	32954627	+	hbv	428	+	exonic	QSER1
7T	chr11	7511971	-	hbv	458	+	intronic	OLFML1
7T	chr13	41185980	-	hbv	458	+	intronic	FOXO1
7T	chr13	75451825	-	hbv	1828	+	intergenic	LINC00347(dist=320568),CTAGE11P(dist=360064)
7T	chr14	23390320	-	hbv	379	+	ncRNA_exonic	PRMT5-AS1
7T	chr14	51741052	-	hbv	457	+	intergenic	TMX1(dist=16680),LINC00640(dist=59059)
7T	chr16	28155849	+	hbv	429	+	intronic	XPO6
7T	chr16	49294493	+	hbv	424	+	intergenic	N4BP1(dist=650373),CBLN1(dist=17336)
7T	chr16	73629155	+	hbv	428	+	intergenic	LOC100506172(dist=173860),LOC101928035(dist=597136)
7T	chr18	30814914	+	hbv	1808	-	intronic	CCDC178
7T	chr18	4772241	-	hbv	424	+	intergenic	DLGAP1(dist=316975),C18orf42(dist=371431)
7T	chr2	55851582	+	hbv	427	+	intergenic	SMEK2(dist=6722),PNPT1(dist=9616)
7T	chr2	61756691	-	hbv	429	+	intronic	XPO1

Supplementary Table S6 Continued

Sample ID	Left_chr	Left_pos	Left_strand	Right_chr	Right_pos	Right_strand	Gene element	Gene (distance)
7T	chr2	242799186	-	hbv	1594	+	intronic	PDCD1
7T	chr20	8823460	+	hbv	422	+	intronic	PLCB1
7T	chr3	45793577	+	hbv	1827	+	intergenic	SACM1L(dist=6677),SLC6A20(dist=3364)
7T	chr3	127240837	+	hbv	476	-	ncRNA_intronic	LINC01471
7T	chr3	120602209	-	hbv	458	+	intergenic	GTF2E1(dist=100293),STXBP5L(dist=24841)
7T	chr4	25514755	+	hbv	429	+	intergenic	ANAPC4(dist=94635),SLC34A2(dist=142680)
7T	chr4	84360311	+	hbv	428	+	intronic	HELQ
7T	chr4	84360603	+	hbv	428	+	intronic	HELQ
7T	chr5	16604119	+	hbv	2987	-	intronic	FAM134B
7T	chr5	100460863	-	hbv	423	+	intergenic	ST8SIA4(dist=221874),SLCO4C1(dist=1108829)
7T	chr6	54975035	+	hbv	1829	+	intergenic	FAM83B(dist=165138),HCRTR2(dist=64036)
7T	chr6	63433768	+	hbv	349	-	intergenic	KHDRBS2(dist=437668),LGSN(dist=552088)
7T	chr6	82953527	+	hbv	448	-	intronic	IBTK
7T	chr6	5765324	-	hbv	1829	+	intronic	FARS2
7T	chr8	21831305	+	hbv	1737	+	intronic	XPO7
7T	chr9	6200479	+	hbv	427	+	intergenic	RANBP6(dist=184839),IL33(dist=15307)
7T	chr9	18576964	+	hbv	377	-	intronic	ADAMTSL1
7T	chr9	77388250	-	hbv	1831	+	intronic	TRPM6
7T	chrM	15786	+	hbv	421	+	intergenic	NONE(dist=NONE),NONE(dist=NONE)
7T	hbv	445	+	chr12	38222898	+	intergenic	NONE(dist=NONE),ALG10B(dist=487659)
7T	hbv	476	+	chr12	2070626	+	intronic	DCP1B
7T	hbv	2568	+	chr22	46796550	+	intronic	CELSR1
7T	hbv	393	+	chr5	49880932	+	intergenic	EMB(dist=143698),PARP8(dist=80801)
7T	hbv	399	+	chr5	14268706	+	intronic	TRIO
7T	hbv	446	+	chr6	130871555	+	intergenic	TMEM200A(dist=107345),SMLR1(dist=276990)
7T	hbv	446	+	chrX	23330449	+	intergenic	PTCHD1-AS(dist=19186),PTCHD1(dist=22536)
10T	chr1	180620221	+	hbv	438	-	intronic	XPR1
10T	chr1	32949949	-	hbv	424	+	intronic	ZBTB8B
10T	chr1	219985681	-	hbv	430	+	intergenic	LYPLAL1(dist=599474),RNU5F-1(dist=60938)
10T	chr13	65712286	-	hbv	421	+	intergenic	OR7E156P(dist=1395585),PCDH9(dist=1164680)
10T	chr2	141466946	-	hbv	424	+	intronic	LRP1B
10T	chr20	60571900	+	hbv	381	+	intronic	TAF4
10T	chr20	41379527	+	hbv	446	-	intronic	PTPRT

Supplementary Table S6 Continued

Sample ID	Left_chr	Left_pos	Left_strand	Right_chr	Right_pos	Right_strand	Gene element	Gene (distance)
10T	chr22	48733956	+	hbv	401	-	intergenic	MIR3201(dist=63729),FAM19A5(dist=151316)
10T	chr5	122480197	-	hbv	429	+	intronic	PRDM6
10T	chr6	144266630	+	hbv	423	+	intronic	PLAGL1
10T	chr7	92813112	-	hbv	457	+	intergenic	SAMD9L(dist=35432),HEPACAM2(dist=4787)
10T	chr9	127641788	-	hbv	402	+	UTR3	GOLGA1
10T	chrUn_gl000220	131469	+	hbv	421	+	intergenic	LOC100507412(dist=4773),RNA5-8S5(dist=24528)
10T	chrX	110421186	+	hbv	474	-	intronic	PAK3
10T	hbv	394	+	chr14	103488869	+	intronic	CDC42BPB
10T	hbv	446	+	chr14	73267551	+	intronic	DPF3
10T	hbv	396	+	chr18	44745760	+	intronic	SKOR2
10T	hbv	393	+	chr3	131481681	+	intronic	CPNE4
10T	hbv	393	+	chr4	134073893	+	exonic	PCDH10
17T	chr10	124811263	-	hbv	425	+	intronic	ACADSB
17T	chr11	133434884	-	hbv	1829	+	intergenic	OPCML(dist=32481),LOC646522(dist=218682)
17T	chr13	87053321	-	hbv	457	+	intergenic	SLITRK6(dist=679838),MIR4500HG(dist=1042921)
17T	chr16	59733557	+	hbv	1828	+	intergenic	GOT2(dist=965296),APOOP5(dist=54488)
17T	chr16	12290913	+	hbv	445	-	intronic	SNX29
17T	chr17	1508993	-	hbv	422	+	intronic	SLC43A2
17T	chr18	45922437	+	hbv	446	-	intergenic	ZBTB7C(dist=258757),CTIF(dist=142990)
17T	chr18	37180029	-	hbv	638	+	ncRNA_intronic	LINC00669
17T	chr19	31448116	+	hbv	1831	+	intergenic	ZNF536(dist=399151),TSHZ3(dist=317735)
17T	chr2	55913359	+	hbv	423	+	intronic	PNPT1
17T	chr2	189520914	+	hbv	425	+	intergenic	GULP1(dist=60262),DIRC1(dist=77551)
17T	chr2	228183710	+	hbv	447	-	ncRNA_intronic	LOC654841
17T	chr2	238628569	-	hbv	459	+	intronic	LRRFIP1
17T	chr3	81539293	+	hbv	423	+	UTR3	GBE1
17T	chr3	188757558	-	hbv	423	+	intergenic	TPRG1-AS1(dist=92130),TPRG1(dist=132205)
17T	chr6	106677273	-	hbv	422	+	intronic	ATG5
17T	chr7	135037385	+	hbv	428	+	intergenic	STRA8(dist=94141),CNOT4(dist=9162)
17T	chr7	131742372	+	hbv	447	-	intergenic	LOC101928782(dist=108660),PLXNA4(dist=65719)
17T	chr8	41019840	+	hbv	457	+	intergenic	ZMAT4(dist=264497),SFRP1(dist=99636)
17T	chr8	85899952	+	hbv	419	+	intergenic	RALYL(dist=65874),LRRCC1(dist=119371)
17T	chr8	112032790	+	hbv	428	+	ncRNA_intronic	LOC101927459

Supplementary Table S6 Continued

Sample ID	Left_chr	Left_pos	Left_strand	Right_chr	Right_pos	Right_strand	Gene element	Gene (distance)
17T	chrX	4684479	+	hbv	459	+	intergenic	LOC101928201(dist=132866),NLGN4X(dist=1123588)
17T	chrX	113664888	+	hbv	427	+	intergenic	LOC101928437(dist=901003),HTR2C(dist=153663)
17T	hbv	402	+	chr14	107027949	+	intergenic	LINC00221(dist=76420),NONE(dist=NONE)
17T	hbv	446	+	chr14	37417422	+	intronic	SLC25A21
17T	hbv	475	+	chr2	220745740	+	intergenic	SLC4A3(dist=239038),MIR4268(dist=25483)
17T	hbv	476	+	chr2	231531657	+	intergenic	SP100(dist=121340),LOC151475(dist=23979)
17T	hbv	446	+	chr6	163308413	+	intronic	PACRG
27T	chr1	56141666	+	hbv	1656	+	intergenic	MIR4422(dist=450270),PPAP2B(dist=818753)
27T	hbv	486	+	chr16	31350299	+	intergenic	ITGAM(dist=6086),ITGAX(dist=16210)
27T	hbv	1624	+	chr17	68129404	+	exonic	KCNJ16
27T	hbv	1826	+	chr19	36212984	+	intronic	MLL4
27T	hbv	1650	+	chr21	46047598	+	exonic	KRTAP10-9
27T	chr6	58778259	+	hbv	2308	+	intergenic	GUSBP4(dist=490535),NONE(dist=NONE)
27T	chr11	64325096	-	hbv	646	+	intronic	SLC22A11
27T	chr12	109284395	-	hbv	647	+	intronic	DAO
27T	chr2	60967143	-	hbv	639	+	intergenic	BCL11A(dist=186510),PAPOLG(dist=16222)
27T	chr5	54449075	+	hbv	641	+	intronic	CDC20B
27T	chr7	132861378	+	hbv	641	+	intergenic	CHCHD3(dist=94550),EXOC4(dist=76445)
27T	chr8	33540444	+	hbv	640	+	intergenic	DUSP26(dist=83005),LINC01288(dist=1100995)
27T	hbv	187	+	chr12	126634840	+	intergenic	LOC101927464(dist=46362),LOC100128554(dist=292187)
38T	chr6	58778259	+	hbv	2308	+	intergenic	GUSBP4(dist=490535),NONE(dist=NONE)
38T	chr1	56141666	+	hbv	1656	+	intergenic	MIR4422(dist=450270),PPAP2B(dist=818753)
38T	hbv	2159	+	chr17	17958160	+	intronic	C17orf39
38T	hbv	1650	+	chr21	46047598	+	exonic	KRTAP10-9
38T	chr4	149968553	-	hbv	1777	+	intergenic	NR3C2(dist=604881),DCLK2(dist=1030873)
38T	chr6	138882474	+	hbv	1919	+	intronic	NHSL1
38T	chr8	98999666	+	hbv	3145	+	intronic	MATN2
38T	chr8	120343397	-	hbv	2086	+	intergenic	MAL2(dist=85483),NOV(dist=85155)
1N	chr1	38625479	+	hbv	458	+	intergenic	MIR3659(dist=70478),LINC01343(dist=49227)
1N	chr11	14073505	-	hbv	430	+	intronic	SPON1
1N	chr13	110379466	+	hbv	394	-	intergenic	MYO16(dist=519111),LINC00676(dist=1155)
1N	chr16	69799439	+	hbv	422	+	intronic	WWP2
1N	chr17	32693521	+	hbv	446	-	intergenic	CCL1(dist=3269),C17orf102(dist=207621)

Supplementary Table S6 Continued

Sample ID	Left_chr	Left_pos	Left_strand	Right_chr	Right_pos	Right_strand	Gene element	Gene (distance)
1N	chr17	27402228	-	hbv	458	+	UTR5	TIAF1
1N	chr2	2170046	+	hbv	458	+	intronic	MYT1L
1N	chr2	227600248	+	hbv	457	+	UTR3	IRS1
1N	chr4	112926954	+	hbv	421	+	intergenic	PITX2(dist=1363675),C4orf32(dist=139599)
1N	chr5	81474410	-	hbv	422	+	intronic	ATG10
1N	chr5	126319497	-	hbv	428	+	intronic	03-Mar
1N	chr6	142288480	+	hbv	446	-	intergenic	MIR4465(dist=1283460),NMBR(dist=108265)
1N	chr8	4156861	+	hbv	445	-	intronic	CSMD1
1N	chr8	24094371	-	hbv	428	+	intergenic	STC1(dist=382051),ADAM28(dist=57209)
1N	chr9	77637130	-	hbv	419	+	intronic	C9orf41
1N	chrUn_gl000220	127352	+	hbv	428	+	downstream	LOC100507412
1N	hbv	393	+	chr11	128288379	+	intergenic	LOC101929497(dist=1081451),ETS1(dist=40277)
1N	hbv	446	+	chr11	107859009	+	intergenic	RAB39A(dist=24801),CUL5(dist=20399)
1N	hbv	395	+	chr12	1911490	+	intronic	CACNA2D4
1N	hbv	394	+	chr16	84568541	+	intergenic	TLDC1(dist=30253),COTL1(dist=30663)
1N	hbv	396	+	chr21	45337630	+	intronic	AGPAT3
1N	hbv	447	+	chr3	195092808	+	intronic	ACAP2
1N	hbv	393	+	chrX	26384219	+	intergenic	MAGEB5(dist=147832),VENTXP1(dist=192235)
2N	chr1	207266235	+	hbv	457	+	intronic	C4BPB
2N	chr1	212650426	+	hbv	425	+	intergenic	NENF(dist=30705),ATF3(dist=88250)
2N	chr1	47693928	-	hbv	1907	+	intronic	TAL1
2N	chr1	117930781	-	hbv	428	+	intronic	MAN1A2
2N	chr10	62618451	+	hbv	2360	+	intergenic	CDK1(dist=63841),RHOBTB1(dist=10747)
2N	chr10	129792111	+	hbv	458	+	intronic	PTPRE
2N	chr10	30198395	-	hbv	1965	+	intergenic	SVIL(dist=173665),KIAA1462(dist=103334)
2N	chr12	107670177	-	hbv	459	+	intergenic	CRY1(dist=182542),BTBD11(dist=42020)
2N	chr13	113529435	+	hbv	3140	+	intronic	ATP11A
2N	chr13	37738581	-	hbv	417	+	intergenic	CSNK1A1L(dist=58780),LINC01048(dist=317020)
2N	chr15	22519050	-	hbv	428	+	intergenic	OR4N3P(dist=104655),REREP3(dist=27515)
2N	chr16	53006343	+	hbv	424	+	intergenic	CASC16(dist=365456),CHD9(dist=82602)
2N	chr17	14427607	+	hbv	3112	+	intergenic	HS3ST3B1(dist=178115),CDRT7(dist=506685)
2N	chr18	48181860	+	hbv	447	-	intronic	MAPK4
2N	chr19	56313164	+	hbv	423	+	intronic	NLRP11

Supplementary Table S6 Continued

Sample ID	Left_chr	Left_pos	Left_strand	Right_chr	Right_pos	Right_strand	Gene element	Gene (distance)
2N	chr19	57071911	+	hbv	420	+	intergenic	ZFP28(dist=3741),ZNF470(dist=6979)
2N	chr2	60595548	+	hbv	457	+	intergenic	LOC101927285(dist=1089013),MIR4432(dist=18949)
2N	chr2	188516380	+	hbv	1979	-	intergenic	TFPI(dist=97161),GULP1(dist=640016)
2N	chr2	493241	-	hbv	425	+	intergenic	FAM150B(dist=204933),TMEM18(dist=174732)
2N	chr20	40561436	+	hbv	445	-	intergenic	CHD6(dist=314303),PTPRT(dist=139956)
2N	chr3	46252052	+	hbv	1	+	intergenic	CCR1(dist=2220),CCR3(dist=31820)
2N	chr3	61928789	+	hbv	447	-	intronic	PTPRG
2N	chr3	31502625	-	hbv	428	+	intergenic	GADL1(dist=566472),STT3B(dist=71368)
2N	chr4	109104728	-	hbv	421	+	intergenic	LEF1-AS1(dist=7142),RPL34-AS1(dist=354618)
2N	chr5	130497062	-	hbv	429	+	intronic	HINT1
2N	chr6	46228472	-	hbv	421	+	intronic	RCAN2
2N	chr6	140850965	-	hbv	457	+	intergenic	MIR3668(dist=324502),MIR4465(dist=153986)
2N	chr7	51921756	+	hbv	457	+	intergenic	COBL(dist=537241),POM121L12(dist=1181593)
2N	chr7	80600545	+	hbv	419	+	intergenic	SEMA3C(dist=51878),HGF(dist=730899)
2N	chr7	12020921	-	hbv	427	+	intergenic	THSD7A(dist=149097),TMEM106B(dist=229927)
2N	chr8	112032790	+	hbv	428	+	ncRNA_intronic	LOC101927459
2N	chr8	95064471	+	hbv	3160	-	intergenic	PDP1(dist=126175),CDH17(dist=74923)
2N	chrUn_gl000220	121616	+	hbv	1998	-	ncRNA_exonic	LOC100507412
2N	chrX	3868463	+	hbv	446	-	intergenic	LOC389906(dist=106528),LOC101928201(dist=676778)
2N	hbv	442	+	chr11	98907062	+	intronic	CNTN5
2N	hbv	446	+	chr18	35847218	+	intergenic	MIR4318(dist=610040),LINC00669(dist=939670)
2N	hbv	499	+	chr6	124184848	+	intronic	NKAIN2
2N	hbv	442	+	chr8	140669746	+	intronic	KCNK9
4N	chr1	59153335	-	hbv	417	+	intronic	MYSM1
4N	chr10	96138123	+	hbv	457	+	intergenic	NOC3L(dist=15440),TBC1D12(dist=24063)
4N	chr11	92014403	+	hbv	1827	+	intergenic	DISC1FP1(dist=1366183),FAT3(dist=70859)
4N	chr11	12423672	-	hbv	423	+	intronic	PARVA
4N	chr12	100432865	+	hbv	423	+	intronic	UHRF1BP1L
4N	chr12	118209807	+	hbv	396	-	intronic	KSR2
4N	chr12	118209887	+	hbv	398	-	intronic	KSR2
4N	chr14	43868789	-	hbv	423	+	intergenic	LRFN5(dist=1495037),FSCB(dist=1104565)
4N	chr15	71375954	+	hbv	446	-	intergenic	LRRC49(dist=33518),CT62(dist=26629)
4N	chr2	59992078	-	hbv	429	+	intergenic	LOC101927285(dist=485543),MIR4432(dist=622419)

Supplementary Table S6 Continued

Sample ID	Left_chr	Left_pos	Left_strand	Right_chr	Right_pos	Right_strand	Gene element	Gene (distance)
4N	chr2	80468517	-	hbv	3115	+	intronic	CTNNA2
4N	chr2	242988526	-	hbv	421	+	ncRNA_intronic	LINC01237
4N	chr20	8948464	+	hbv	426	+	intergenic	PLCB1(dist=82917),PLCB4(dist=101237)
4N	chr20	35681558	+	hbv	2492	-	intronic	RBL1
4N	chr22	23476573	+	hbv	428	+	intronic	RTDR1
4N	chr3	135262381	-	hbv	458	+	intergenic	EPHB1(dist=283074),PPP2R3A(dist=422134)
4N	chr3	142070133	-	hbv	430	+	intronic	XRN1
4N	chr4	68716504	+	hbv	457	+	intronic	TMPRSS11D
4N	chr5	22249859	-	hbv	457	+	intronic	CDH12
4N	chr5	93797758	-	hbv	379	+	intronic	KIAA0825
4N	chr6	141158309	-	hbv	423	+	intergenic	MIR4465(dist=153289),NMBR(dist=1238436)
4N	chr7	88602629	-	hbv	418	+	intronic	ZNF804B
4N	chr8	37440057	+	hbv	420	+	intergenic	LOC100507420(dist=61153),ZNF703(dist=113244)
4N	chr9	73247983	-	hbv	359	+	intronic	TRPM3
4N	hbv	446	+	chr1	85923675	+	intronic	DDAH1
4N	hbv	1665	+	chr10	128862025	+	intronic	DOCK1
4N	hbv	1854	+	chr12	76244598	+	intergenic	KRR1(dist=339180),PHLDA1(dist=174629)
4N	hbv	446	+	chr2	40471182	+	ncRNA_intronic	SLC8A1-AS1
4N	hbv	447	+	chr3	101542614	+	UTR3	NXPE3
4N	hbv	446	+	chr5	23333828	+	intergenic	CDH12(dist=480097),PRDM9(dist=173896)
4N	hbv	447	+	chr6	66256174	+	intronic	EYS
8N	chr1	20493187	+	hbv	429	+	intronic	PLA2G2C
8N	chr1	246271042	+	hbv	458	+	intronic	SMYD3
8N	chr1	184263590	+	hbv	447	-	intergenic	TSEN15(dist=220246),C1orf21(dist=92560)
8N	chr1	192830451	-	hbv	427	+	intergenic	RGS2(dist=49044),UCHL5(dist=151045)
8N	chr10	4574446	+	hbv	421	+	intergenic	LINC00703(dist=121642),LINC00704(dist=117931)
8N	chr10	107936056	+	hbv	424	+	intergenic	LOC101927549(dist=355965),SORCS1(dist=397365)
8N	chr10	71645424	+	hbv	396	-	intronic	COL13A1
8N	chr10	111670197	-	hbv	428	+	intronic	XPNPEP1
8N	chr11	46394803	+	hbv	429	+	intronic	DGKZ
8N	chr11	77158078	+	hbv	1827	+	intronic	PAK1
8N	chr11	85117918	+	hbv	440	-	intronic	DLG2
8N	chr12	5595059	+	hbv	429	+	intronic	NTF3

Supplementary Table S6 Continued

Sample ID	Left_chr	Left_pos	Left_strand	Right_chr	Right_pos	Right_strand	Gene element	Gene (distance)
8N	chr12	34150234	+	hbv	429	+	intergenic	
8N	chr14	102096201	+	hbv	457	+	intergenic	
8N	chr14	104046300	+	hbv	429	+	intronic	
8N	chr14	81349146	-	hbv	428	+	intronic	
8N	chr16	21287654	+	hbv	421	+	intronic	
8N	chr16	56446453	+	hbv	421	+	intronic	
8N	chr17	70403967	-	hbv	427	+	ncRNA_intronic	LINC00673
8N	chr18	19457058	+	hbv	429	+	intergenic	MIB1(dist=6140),GATA6-AS1(dist=289801)
8N	chr19	23494320	+	hbv	1829	+	intergenic	IPO5P1(dist=37267),ZNF91(dist=46178)
8N	chr2	234741830	+	hbv	403	+	exonic	MROH2A
8N	chr21	42475122	+	hbv	427	+	intergenic	DSCAM(dist=256083),LINC00323(dist=38305)
8N	chr22	30106845	-	hbv	424	+	intergenic	NF2(dist=12256),CABP7(dist=9499)
8N	chr3	132028338	+	hbv	420	+	intergenic	CPNE4(dist=269888),ACPP(dist=7873)
8N	chr3	164322908	+	hbv	324	+	intergenic	LINC01192(dist=1301819),SI(dist=373778)
8N	chr3	183608429	-	hbv	420	+	intergenic	PARL(dist=5736),ABCC5(dist=29295)
8N	chr4	160809384	+	hbv	421	+	intergenic	RAPGEF2(dist=528083),FSTL5(dist=1495660)
8N	chr4	186566104	+	hbv	421	+	intronic	SORBS2
8N	chr4	179094833	-	hbv	457	+	intergenic	LINC01098(dist=182929),NONE(dist=NONE)
8N	chr5	138185972	+	hbv	428	+	intronic	CTNNA1
8N	chr6	169675305	+	hbv	425	+	intergenic	THBS2(dist=21096),WDR27(dist=181998)
8N	chr6	36433638	-	hbv	420	+	intronic	KCTD20
8N	chr7	138238138	-	hbv	418	+	intronic	TRIM24
8N	chr8	68708985	+	hbv	426	+	intergenic	CPA6(dist=50365),PREX2(dist=155618)
8N	chr8	32566403	+	hbv	445	-	intronic	NRG1
8N	chr8	47558633	-	hbv	424	+	intergenic	NONE(dist=NONE),LINC00293(dist=193875)
8N	chr8	138862889	-	hbv	421	+	intergenic	LOC101927915(dist=437058),FAM135B(dist=279377)
8N	chr9	135547758	+	hbv	461	+	intronic	GTF3C4
8N	chrM	14972	-	hbv	1829	+	intergenic	NONE(dist=NONE),NONE(dist=NONE)
8N	chrUn_gl000220	127671	+	hbv	423	+	downstream	LOC100507412
8N	chrX	10373970	+	hbv	398	-	intergenic	CLCN4(dist=168271),MID1(dist=39380)
8N	chrX	148435119	+	hbv	446	-	intergenic	AFF2(dist=352926),IDS(dist=125176)
8N	hbv	446	+	chr10	30992651	+	ncRNA_intronic	SVILP1
8N	hbv	446	+	chr14	56576013	+	intergenic	LINC00520(dist=312621),PELI2(dist=9080)

Supplementary Table S6 Continued

Sample ID	Left_chr	Left_pos	Left_strand	Right_chr	Right_pos	Right_strand	Gene element	Gene (distance)
8N	hbv	396	+	chr2	76133942	+	intergenic	GCFC2(dist=195831),LRRTM4(dist=840908)
8N	hbv	476	+	chr3	110256892	+	intergenic	LINC01205(dist=1042878),PVRL3-AS1(dist=507271)
8N	hbv	395	+	chr5	2311387	+	ncRNA_intronic	LOC100506858
8N	hbv	447	+	chr8	25489921	+	intergenic	CDCA2(dist=124496),EBF2(dist=209325)
8N	hbv	442	+	chrX	22991645	+	ncRNA_intronic	PTCHD1-AS
8N	hbv	447	+	chrX	40172226	+	intergenic	LOC101927476(dist=25252),ATP6AP2(dist=267990)
16N	chr1	236325099	+	hbv	428	+	intronic	GPR137B
16N	chr10	99306794	+	hbv	423	+	intronic	UBTD1
16N	chr11	49910488	+	hbv	418	+	intergenic	LOC440040(dist=78519),OR4C13(dist=63455)
16N	chr11	74240800	+	hbv	1828	+	intergenic	LIPT2(dist=36045),POLD3(dist=62775)
16N	chr12	115455189	+	hbv	457	+	intergenic	TBX3(dist=333220),MED13L(dist=941192)
16N	chr12	87351076	-	hbv	423	+	intergenic	MGAT4C(dist=118395),MKRN9P(dist=825587)
16N	chr14	85970718	-	hbv	428	+	intergenic	LINC00911(dist=84300),FLRT2(dist=25770)
16N	chr15	61945218	+	hbv	445	-	intergenic	RORA(dist=423716),VPS13C(dist=199372)
16N	chr2	143656867	+	hbv	475	-	intronic	KYNU
16N	chr3	173630064	+	hbv	458	+	ncRNA_intronic	NLGN1-AS1
16N	chr4	168730616	+	hbv	422	+	intergenic	SPOCK3(dist=574875),ANXA10(dist=283072)
16N	chr4	54742642	-	hbv	421	+	intergenic	LNX1-AS2(dist=271094),RPL21P44(dist=109024)
16N	chr5	87305681	+	hbv	422	+	intergenic	CCNH(dist=596831),TMEM161B(dist=179769)
16N	chr6	36196128	+	hbv	349	+	intronic	BRPF3
16N	chr6	14035005	-	hbv	430	+	intergenic	RNF182(dist=54765),CD83(dist=82482)
16N	chr7	35548436	+	hbv	422	+	intergenic	LOC401324(dist=132350),HERPUD2(dist=123834)
16N	chr8	124957294	+	hbv	430	+	intronic	FER1L6
16N	chrUn_gl000220	127859	+	hbv	424	+	intergenic	LOC100507412(dist=1163),RNA5-8S5(dist=28138)
16N	chrUn_gl000220	132067	+	hbv	423	+	intergenic	LOC100507412(dist=5371),RNA5-8S5(dist=23930)
16N	chrUn_gl000220	132102	+	hbv	428	+	intergenic	LOC100507412(dist=5406),RNA5-8S5(dist=23895)
16N	chrX	110639109	-	hbv	428	+	ncRNA_intronic	RNU6-28P
16N	hbv	446	+	chr2	167431694	+	intergenic	SCN7A(dist=88213),XIRP2(dist=313303)
16N	hbv	447	+	chr9	5340027	+	upstream	RLN1
17N	chr1	93679396	+	hbv	423	+	intronic	CCDC18
17N	chr1	193467650	+	hbv	430	+	intergenic	LINC01031(dist=132567),NONE(dist=NONE)
17N	chr10	36570223	+	hbv	446	-	intergenic	LINC01452(dist=480375),ANKRD30A(dist=844562)
17N	chr10	93333432	+	hbv	394	-	ncRNA_intronic	HECTD2-AS1

Supplementary Table S6 Continued

Sample ID	Left_chr	Left_pos	Left_strand	Right_chr	Right_pos	Right_strand	Gene element	Gene (distance)
17N	chr10	129370769	+	hbv	394	-	intergenic	NPS(dist=19834),FOXI2(dist=164769)
17N	chr11	80823628	+	hbv	428	+	intergenic	LOC101928944(dist=349782),LOC101928989(dist=767265)
17N	chr11	46405826	+	hbv	447	-	downstream	CHRM4,MDK
17N	chr11	14370515	-	hbv	639	+	intronic	RRAS2
17N	chr11	32001831	-	hbv	430	+	intergenic	DKFZp686K1684(dist=93244),LOC100506675(dist=55694)
17N	chr13	48831854	+	hbv	457	+	intronic	ITM2B
17N	chr13	107622751	+	hbv	423	+	intergenic	LINC00443(dist=298223),FAM155A(dist=198128)
17N	chr14	51808283	+	hbv	457	+	ncRNA_intronic	LINC00640
17N	chr14	97740123	+	hbv	428	+	intergenic	LINC00618(dist=328392),LOC101929241(dist=185030)
17N	chr14	38143243	-	hbv	457	+	intergenic	FOXA1(dist=78918),SSTR1(dist=533961)
17N	chr17	34207944	-	hbv	2469	+	upstream	CCL5
17N	chr17	56681732	-	hbv	429	+	intronic	TEX14
17N	chr18	54980390	-	hbv	348	+	intergenic	BOD1L2(dist=162751),ST8SIA3(dist=39331)
17N	chr3	171236308	-	hbv	423	+	intergenic	TNIK(dist=58111),PLD1(dist=81887)
17N	chr4	55733439	+	hbv	425	+	intergenic	KIT(dist=126558),KDR(dist=210987)
17N	chr4	119711624	+	hbv	324	+	intronic	SEC24D
17N	chr4	29070920	+	hbv	393	-	intergenic	MIR4275(dist=249630),PCDH7(dist=1651110)
17N	chr5	90095658	+	hbv	423	+	intronic	GPR98
17N	chr5	177946828	-	hbv	423	+	intronic	COL23A1
17N	chr6	43812935	+	hbv	422	+	intergenic	VEGFA(dist=58712),LINC01512(dist=45830)
17N	chr8	5226549	+	hbv	426	+	intergenic	CSMD1(dist=374221),LOC100287015(dist=1034528)
17N	chrUn_gl000220	132142	+	hbv	423	+	intergenic	LOC100507412(dist=5446),RNA5-8S5(dist=23855)
17N	chrX	40321041	+	hbv	399	-	intergenic	LOC101927476(dist=174067),ATP6AP2(dist=119175)
17N	chrX	135546041	-	hbv	421	+	intergenic	GPR112(dist=46994),BRS3(dist=24084)
17N	hbv	399	+	chr11	131781306	+	UTR5	NTM
17N	hbv	396	+	chr2	22403238	+	intergenic	LOC645949(dist=469714),LOC102723362(dist=356113)
17N	hbv	441	+	chr2	135551813	+	intergenic	TMEM163(dist=75242),ACMSD(dist=44373)
17N	hbv	396	+	chr6	106801275	+	intergenic	ATG5(dist=27580),AIM1(dist=158455)
17N	hbv	444	+	chr9	37887966	+	exonic	SLC25A51
27N	chr17	45286882	-	hbv	1873	+	exonic	MYL4
38N	chr12	73019677	-	hbv	1768	+	intronic	TRHDE
38N	chr3	169564731	-	hbv	2193	+	intronic	LRRC31

Supplementary Table S7. Multivariate analysis of the HBV integration in PDAC patients (n=86)

**Parameters**	HBV serum marker - (n=43)	HBV serum marker + (n=43)	HBV integration + (n=10)	P value[a]	P value[b]
**Age**	66.2 ± 7.9	66.7 ± 11.7	66.3 ± 12.4	0.872	0.892
**Gender**				0.829	0.842
**Male**	20 (46.5%)	21 (48.8%)	5 (50.0%)		
**Female**	23 (53.5%)	22 (51.2%)	5 (50.0%)		
**Tumor Location**				1	0.87
**Head**	27 (62.8%)	27 (62.8%)	6 (60.0%)		
**Body and tail**	16 (37.2%)	16 (37.2%)	4 (40.0%)		
**Tumor Volume (mm^3)**	19.5 ± 22.0	11.7 ± 11.6	10.1 ± 7.6	**0.042**	0.192
**Histological Grade**				0.086	0.098
**I-II**	32 (74.4%)	39 (90.7%)	10 (100.0%)		
**III**	11 (25.6%)	4 (9.3%)	0 (0.0%)		
**Tumor Stage**				0.579	0.437
**I+II**	34 (79.1%)	36 (83.7%)	9 (90.0%)		
**III+IV**	9 (20.9%)	7 (16.3%)	1 (10.0%)		
**Vascular Invasion**				0.763	0.376
**I+II**	7 (16.3%)	6 (13.9%)	3 (30.0%)		
**III+IV**	36 (83.7%)	37 (86.1%)	7 (70.0%)		

Supplementary Table S7 Continued

Parameters	HBV serum marker - (n=43)	HBV serum marker + (n=43)	HBV integration + (n=10)	P value[a]	P value[b]
**Liver metastasis**				0.713	1.000
**Positive**	3 (6.9%)	5 (11.6%)	1 (10.0%)		
**Negative**	40 (93.1%)	38 (88.4%)	9 (90.0%)		
**Lymphatic Metastasis**				0.820	0.719
**Positive**	14 (32.5%)	15 (34.9%)	4 (40.0%)		
**Negative**	29 (67.5%)	28 (65.1%)	6 (60.0%)		

p value[a] for HBV makers positive pancreatic cancer (PDAC) and HBV makers negative PDAC.

p value[b] for HBV integration positive PDAC and HBV makers negative PDAC.

Supplementary Table S8. Pooled analysis of the viral-host junctions of hepatocellular carcinoma and non-tumor from 48 literatures

Chromosome	Integration site in host genome	Integration site in virus genome	Gene (distance, bp)	Regions	Methods	Author	PMID	Sample
2p24.1			EMX2-like	1.8 kb upstream to the EMX2-like ATG codon	Alu-PCR	Bréchot P et al.2003	12813464	Tumor
3p26			IP3R type 1	39th exon of IP3R type 1 gene	Alu-PCR	Bréchot P et al.2003	12813464	Tumor
12p11			IP3Rtype2	45 kb upstream to the IP3R type2 start codon	Alu-PCR	Bréchot P et al.2003	12813464	Tumor
3q25.3			IRAK2	6 kb upstream to the IRAK2 start codon	Alu-PCR	Bréchot P et al.2003	12813464	Tumor
22q11.22			MAPK1	20 kb upstream to the MAPK1 start codon	Alu-PCR	Bréchot P et al.2003	12813464	Tumor
9q21.1			NTRK2	13 kb downstream to the NTRK2 stop codon	Alu-PCR	Bréchot P et al.2003	12813464	Tumor
3q11.2			ST3GAL VI	3 kb upstream to the ST3GAL VI start codon	Alu-PCR	Bréchot P et al.2003	12813464	Tumor
14q21.1			TRUP	8 kb downstream to the TRUP stop codon	Alu-PCR	Bréchot P et al.2003	12813464	Tumor
chr5	1282714	342	TERT	exonic	RNA-seq	Chiu et al.2016	26867494	Tumor
chr5	1282739	458	TERT	exonic	RNA-seq	Chiu et al.2016	26867494	Tumor
chr5	1294780	460	TERT	exonic	RNA-seq	Chiu et al.2016	26867494	Tumor
chr11	116703541	344	APOC3	exonic	RNA-seq	Chiu et al.2016	26867494	Tumor
chr12	20704464	337	PDE3A	intronic	RNA-seq	Chiu et al.2016	26867494	Tumor
chr15	69563770	799	GLCE	3UTR	RNA-seq	Chiu et al.2016	26867494	Tumor
chr2	100020225	1284	REV1	exonic	RNA-seq	Chiu et al.2016	26867494	Tumor
chr4	149116013	458	NR3C2	exonic	RNA-seq	Chiu et al.2016	26867494	Tumor
chr4	149181270	458	NR3C2	splicing	RNA-seq	Chiu et al.2016	26867494	Tumor
chr4	149344265	1816	NR3C2	intronic	RNA-seq	Chiu et al.2016	26867494	Tumor
chr4	149344309	1566	NR3C2	intronic	RNA-seq	Chiu et al.2016	26867494	Tumor
chr4	149344354	1638	NR3C2	intronic	RNA-seq	Chiu et al.2016	26867494	Tumor
chr14	94847249	1650	SERPINA1	exonic	RNA-seq	Chiu et al.2016	26867494	Tumor
chr8	10286162	1629	MSRA	3UTR	RNA-seq	Chiu et al.2016	26867494	Tumor
chr1	8075501	323	ERRFI1	intronic	RNA-seq	Chiu et al.2016	26867494	Tumor
chr12	6647091	793	GAPDH	exonic	RNA-seq	Chiu et al.2016	26867494	Tumor
chr12	50482505	1546	SMARCD1	intronic	RNA-seq	Chiu et al.2016	26867494	Tumor
chr14	94849420	874	SERPINA1	exonic	RNA-seq	Chiu et al.2016	26867494	Tumor
chr16	72094539	1554	HP	exonic	RNA-seq	Chiu et al.2016	26867494	Tumor
chr16	72094663	483	HP	exonic	RNA-seq	Chiu et al.2016	26867494	Tumor
chr17	36872024	1516	MLLT6	exonic	RNA-seq	Chiu et al.2016	26867494	Tumor
chr17	79801120	243	P4HB	3UTR	RNA-seq	Chiu et al.2016	26867494	Tumor
chr19	45412501	366	APOE	exonic	RNA-seq	Chiu et al.2016	26867494	Tumor
chr19	49468650	1176	FTL	5UTR	RNA-seq	Chiu et al.2016	26867494	Tumor
chr19	59069834	287	UBE2M	5UTR	RNA-seq	Chiu et al.2016	26867494	Tumor
chr2	46540192	1566	EPAS1	intronic	RNA-seq	Chiu et al.2016	26867494	Tumor
chr20	56140866	170	PCK1	3UTR	RNA-seq	Chiu et al.2016	26867494	Tumor
chr22	38880661	1810	DDX17	3UTR	RNA-seq	Chiu et al.2016	26867494	Tumor
chr3	11079259	3291	SLC6A1	3UTR	RNA-seq	Chiu et al.2016	26867494	Tumor
chr3	125836916	1120	ALDH1L1	exonic	RNA-seq	Chiu et al.2016	26867494	Tumor
chr4	74279314	513	ALB	exonic	RNA-seq	Chiu et al.2016	26867494	Tumor
chr4	74283366	1932	ALB	exonic	RNA-seq	Chiu et al.2016	26867494	Tumor
chr4	74285401	1006	ALB	intronic	RNA-seq	Chiu et al.2016	26867494	Tumor
chr6	31321960	387	HLA-B	3UTR	RNA-seq	Chiu et al.2016	26867494	Tumor
chr6	142703147	385	GPR126	exonic	RNA-seq	Chiu et al.2016	26867494	Tumor
chr7	99361609	1860	CYP3A4	exonic	RNA-seq	Chiu et al.2016	26867494	Tumor
chr17	79801858	518	P4HB	3UTR	RNA-seq	Chiu et al.2016	26867494	Tumor
chr19	10260349	110	DNMT1	intronic	RNA-seq	Chiu et al.2016	26867494	Tumor
chr3	45785190	1828	SACM1L	3UTR	RNA-seq	Chiu et al.2016	26867494	Tumor
chr13	37450580	1844	SMAD9	intronic	RNA-seq	Chiu et al.2016	26867494	Tumor
chr19	36212642	1984	KMT2B	exonic	RNA-seq	Chiu et al.2016	26867494	Tumor
chr19	36212682	1831	KMT2B	exonic	RNA-seq	Chiu et al.2016	26867494	Tumor
chr19	36212778	1827	KMT2B	intronic	RNA-seq	Chiu et al.2016	26867494	Tumor
chr19	36213288	458	KMT2B	splicing	RNA-seq	Chiu et al.2016	26867494	Tumor
chr12	124809405	1945	NCOR2	3UTR	RNA-seq	Chiu et al.2016	26867494	Tumor
chr1	201104510	317	TMEM9	3UTR	RNA-seq	Chiu et al.2016	26867494	Tumor
chr12	82021685	142	PPFIA2	intronic	RNA-seq	Chiu et al.2016	26867494	Tumor
chr21	45666394	532	DNMT3L	exonic	RNA-seq	Chiu et al.2016	26867494	Tumor
chr3	72423905	370	RYBP	3UTR	RNA-seq	Chiu et al.2016	26867494	Tumor
chr4	122742247	458	CCNA2	splicing	RNA-seq	Chiu et al.2016	26867494	Tumor
chr4	122742542	1413	CCNA2	intronic	RNA-seq	Chiu et al.2016	26867494	Tumor
chr4	122742560	3177	CCNA2	intronic	RNA-seq	Chiu et al.2016	26867494	Tumor
chr4	122743559	282	CCNA2	exonic	RNA-seq	Chiu et al.2016	26867494	Tumor
chr4	155526071	437	FGG	exonic	RNA-seq	Chiu et al.2016	26867494	Tumor
chrX	70520535	1748	NONO	3UTR	RNA-seq	Chiu et al.2016	26867494	Tumor
chr1	151265228	3173	PI4KB	3UTR	RNA-seq	Chiu et al.2016	26867494	Tumor
chr10	72639446	1326	SGPL1	3UTR	RNA-seq	Chiu et al.2016	26867494	Tumor
chr12	65636246	1800	LEMD3	intronic	RNA-seq	Chiu et al.2016	26867494	Tumor
chr17	40133968	1737	DNAJC7	exonic	RNA-seq	Chiu et al.2016	26867494	Tumor
chr4	122742247	2449	CCNA2	splicing	RNA-seq	Chiu et al.2016	26867494	Tumor
chr4	122742792	1801	CCNA2	intronic	RNA-seq	Chiu et al.2016	26867494	Tumor
chr1	146644586	1773	PRKAB2	upstream	RNA-seq	Chiu et al.2016	26867494	Tumor
chr1	160264286	1528	COPA	exonic	RNA-seq	Chiu et al.2016	26867494	Tumor
chr1	161192714	758	APOA2	exonic	RNA-seq	Chiu et al.2016	26867494	Tumor
chr1	161192897	471	APOA2	intronic	RNA-seq	Chiu et al.2016	26867494	Tumor
chr1	161193156	898	APOA2	exonic	RNA-seq	Chiu et al.2016	26867494	Tumor
chr11	116703608	1823	APOC3	3UTR	RNA-seq	Chiu et al.2016	26867494	Tumor
chr12	9224964	302	A2M	exonic	RNA-seq	Chiu et al.2016	26867494	Tumor
chr13	45914258	313	TPT1	exonic	RNA-seq	Chiu et al.2016	26867494	Tumor
chr13	95271908	1789	GPR180	intronic	RNA-seq	Chiu et al.2016	26867494	Tumor
chr17	7013095	1708	ASGR2	intronic	RNA-seq	Chiu et al.2016	26867494	Tumor
chr17	8385212	3019	MYH10	intronic	RNA-seq	Chiu et al.2016	26867494	Tumor
chr17	8385213	1831	MYH10	intronic	RNA-seq	Chiu et al.2016	26867494	Tumor
chr17	79862445	898	PCYT2	3UTR	RNA-seq	Chiu et al.2016	26867494	Tumor
chr19	6719361	371	C3	exonic	RNA-seq	Chiu et al.2016	26867494	Tumor
chr19	56185749	1730	U2AF2	3UTR	RNA-seq	Chiu et al.2016	26867494	Tumor
chr3	5025011	423	BHLHE40	exonic	RNA-seq	Chiu et al.2016	26867494	Tumor
chr3	63378841	3019	SYNPR	intronic	RNA-seq	Chiu et al.2016	26867494	Tumor
chr3	186338722	422	AHSG	3UTR	RNA-seq	Chiu et al.2016	26867494	Tumor
chr3	193247712	3009	ATP13A4	intronic	RNA-seq	Chiu et al.2016	26867494	Tumor

Chromosome	Integration site in host genome	Integration site in virus genome	Gene (distance, bp)	Regions	Methods	Author	PMID	Sample
chr4	666304	398	ATP5I	exonic	RNA-seq	Chiu et al.2016	26867494	Tumor
chr4	74277742	634	ALB	exonic	RNA-seq	Chiu et al.2016	26867494	Tumor
chr4	74283248	380	ALB	exonic	RNA-seq	Chiu et al.2016	26867494	Tumor
chr4	74283336	268	ALB	exonic	RNA-seq	Chiu et al.2016	26867494	Tumor
chr4	74284027	436	ALB	exonic	RNA-seq	Chiu et al.2016	26867494	Tumor
chr5	180076114	1631	FLT4	intronic	RNA-seq	Chiu et al.2016	26867494	Tumor
chr6	17928712	1281	KIF13A	intronic	RNA-seq	Chiu et al.2016	26867494	Tumor
chr6	31700025	399	CLIC1	exonic	RNA-seq	Chiu et al.2016	26867494	Tumor
chr6	75950956	3350	COX7A2	exonic	RNA-seq	Chiu et al.2016	26867494	Tumor
chr6	160102549	341	SOD2	intronic	RNA-seq	Chiu et al.2016	26867494	Tumor
chr9	37432099	694	GRHPR	exonic	RNA-seq	Chiu et al.2016	26867494	Tumor
chr9	117086049	431	ORM1	exonic	RNA-seq	Chiu et al.2016	26867494	Tumor
chr9	127977077	842	RABEPK	intronic	RNA-seq	Chiu et al.2016	26867494	Tumor
chrX	114869286	1034	PLS3	exonic	RNA-seq	Chiu et al.2016	26867494	Tumor
17p11.2-12					Southern blot hybridization	BettyL et al. 1991		
10	112957941		ADRA2A	down	MAPS	Ding et al.2012	23236287	Tumor
12	38719823		ALG10B	in	MAPS	Ding et al.2012	23236287	Tumor
8	58214918		C8orf71	downstream	MAPS	Ding et al.2012	23236287	Tumor
4	122742990		CCNA2	in	MAPS	Ding et al.2012	23236287	Tumor
3	1171741		CNTN6	in	MAPS	Ding et al.2012	23236287	Tumor
10	96804432		CYP2C8	in	MAPS	Ding et al.2012	23236287	Tumor
16	23703217		ERN2	in	MAPS	Ding et al.2012	23236287	Tumor
7	173848		FAM20C	up	MAPS	Ding et al.2012	23236287	Tumor
2	216293206		FN1	in	MAPS	Ding et al.2012	23236287	Tumor
5	141379511		GNPDA1	down	MAPS	Ding et al.2012	23236287	Tumor
17	47157562		IGF2BP1	downstream	MAPS	Ding et al.2012	23236287	Tumor
7	65867088		LINC00174	upstream	MAPS	Ding et al.2012	23236287	Tumor
9	28048670		LINGO2	in	MAPS	Ding et al.2012	23236287	Tumor
10	45447575		LOC220980	in	MAPS	Ding et al.2012	23236287	Tumor
4	117330071		MIR1973	downstream	MAPS	Ding et al.2012	23236287	Tumor
19	36213719		KMT2B	in	MAPS	Ding et al.2012	23236287	Tumor
2	216856472		MREG	in	MAPS	Ding et al.2012	23236287	Tumor
15	102327821		OR4F6	upstream	MAPS	Ding et al.2012	23236287	Tumor
5	114599279		PGGT1B	upstream	MAPS	Ding et al.2012	23236287	Tumor
20	58053923		PHACTR3	up	MAPS	Ding et al.2012	23236287	Tumor
12	9089956		PHC1	in	MAPS	Ding et al.2012	23236287	Tumor
3	138408800		PIK3CB	in	MAPS	Ding et al.2012	23236287	Tumor
7	132358062		PLXNA4	up	MAPS	Ding et al.2012	23236287	Tumor
17	15087962		PMP22	downstream	MAPS	Ding et al.2012	23236287	Tumor
12	68942537		RAP1B	upstream	MAPS	Ding et al.2012	23236287	Tumor
1	8307107		SLC45A1	up	MAPS	Ding et al.2012	23236287	Tumor
5	135491847		SMAD5	in	MAPS	Ding et al.2012	23236287	Tumor
5	135491851		SMAD5	in	MAPS	Ding et al.2012	23236287	Tumor
5	1295135		TERT	in	MAPS	Ding et al.2012	23236287	Tumor
5	1295396		TERT	upstream	MAPS	Ding et al.2012	23236287	Tumor
5	1295441		TERT	up	MAPS	Ding et al.2012	23236287	Tumor
5	1295563		TERT	upstream	MAPS	Ding et al.2012	23236287	Tumor
5	1295715		TERT	up	MAPS	Ding et al.2012	23236287	Tumor
5	1298846		TERT	up	MAPS	Ding et al.2012	23236287	Tumor
5	12322895				MAPS	Ding et al.2012	23236287	Tumor
17	14551118				MAPS	Ding et al.2012	23236287	Tumor
9	31699788				MAPS	Ding et al.2012	23236287	Tumor
8	50495165				MAPS	Ding et al.2012	23236287	Tumor
13	82830019				MAPS	Ding et al.2012	23236287	Tumor
11	87566555				MAPS	Ding et al.2012	23236287	Tumor
15	95266422				MAPS	Ding et al.2012	23236287	Tumor
2	103820709				MAPS	Ding et al.2012	23236287	Tumor
15	91509760	1813	PRC1	Exon	RNA-seq	Dong et al. 2015	25901726	Nontumor
15	91509751	1796	PRC1	Exon	RNA-seq	Dong et al. 2015	25901726	Nontumor
10	102723937	1869	FAM178A	Exon	RNA-seq	Dong et al. 2015	25901726	Nontumor
1	151032547	1784	MLLT11	Exon	RNA-seq	Dong et al. 2015	25901726	Nontumor
7	74125369	464	GTF2I	Exon	RNA-seq	Dong et al. 2015	25901726	Nontumor
2	216253024	458	FN1	Exon	RNA-seq	Dong et al. 2015	25901726	Nontumor
17	10401079	456	MYH1	Exon	RNA-seq	Dong et al. 2015	25901726	Tumor
19	30313234	343	CCNE1	Exon	RNA-seq	Dong et al. 2015	25901726	Tumor
19	33517538	2087	RHPN2	Exon	RNA-seq	Dong et al. 2015	25901726	Tumor
19	33535159	2062	RHPN2	Exon	RNA-seq	Dong et al. 2015	25901726	Tumor
19	36213551	294	KMT2B	Exon	RNA-seq	Dong et al. 2015	25901726	Tumor
19	36213556	1825	KMT2B	Exon	RNA-seq	Dong et al. 2015	25901726	Tumor
19	36212238	1824	KMT2B	Exon	RNA-seq	Dong et al. 2015	25901726	Tumor
19	36212705	2934	KMT2B	Exon	RNA-seq	Dong et al. 2015	25901726	Tumor
13	45914856	323	TPT1	Exon	RNA-seq	Dong et al. 2015	25901726	Tumor
6	84320396	458	SNAP91	Exon	RNA-seq	Dong et al. 2015	25901726	Tumor
10	12070978	1824	UPF2	Exon	RNA-seq	Dong et al. 2015	25901726	Tumor
19	36212607	3139	KMT2B	Exon	RNA-seq	Dong et al. 2015	25901726	Tumor
2	43902713	1807	LOC728819	Exon	RNA-seq	Dong et al. 2015	25901726	Tumor
19	12841836	1828	C19orf43	Exon	RNA-seq	Dong et al. 2015	25901726	Tumor
1	161193408	1551	APOA2	Exon	RNA-seq	Dong et al. 2015	25901726	Tumor
1	161193413	422	APOA2	Exon	RNA-seq	Dong et al. 2015	25901726	Tumor
3	186338806	1109	AHSG	Exon	RNA-seq	Dong et al. 2015	25901726	Tumor
9	116838918	1526	AMBP	Exon	RNA-seq	Dong et al. 2015	25901726	Tumor
19	36212560	2103	KMT2B	Exon	RNA-seq	Dong et al. 2015	25901726	Tumor
19	36212705	1985	KMT2B	Exon	RNA-seq	Dong et al. 2015	25901726	Tumor
19	36212705	281	KMT2B	Exon	RNA-seq	Dong et al. 2015	25901726	Tumor
19	36213611	2297	KMT2B	Exon	RNA-seq	Dong et al. 2015	25901726	Tumor
19	36213620	1826	KMT2B	Exon	RNA-seq	Dong et al. 2015	25901726	Tumor
12	116534555	2449	MED13L	Exon	RNA-seq	Dong et al. 2015	25901726	Tumor
19	36212705	2298	KMT2B	Exon	RNA-seq	Dong et al. 2015	25901726	Tumor
17	13933678	457	COX10-AS1	Exon	RNA-seq	Dong et al. 2015	25901726	Tumor
19	8386583	1782	RPS28	Exon	RNA-seq	Dong et al. 2015	25901726	Tumor
7	70267023	1895	AUTS2	9137	NGS	Fujimoto et al. 2012	22634756	Tumor
5	1310429	1411	CLPTM1L	7570	NGS	Fujimoto et al. 2012	22634756	Tumor
4	79048012	1808	FRAS1	0	NGS	Fujimoto et al. 2012	22634756	Tumor
3	121439129	1361	GOLGB1	0	NGS	Fujimoto et al. 2012	22634756	Tumor

Chromosome	Integration site in host genome	Integration site in virus genome	Gene (distance, bp)	Regions	Methods	Author	PMID	Sample
9	24396870	1814	IZUMO3	146342	NGS	Fujimoto et al. 2012	22634756	Tumor
5	88432442	1735	NA	NA	NGS	Fujimoto et al. 2012	22634756	Tumor
4	151954261	1846	NONHSAG039094	5491	NGS	Fujimoto et al. 2012	22634756	Tumor
5	88754909	687	NONHSAG040983	7445	NGS	Fujimoto et al. 2012	22634756	Tumor
5	173125603	1879	NONHSAG042275,NONHSAG042273	0,0	NGS	Fujimoto et al. 2012	22634756	Tumor
6	68664024	1470	NONHSAG044066	1300	NGS	Fujimoto et al. 2012	22634756	Tumor
7	24499331	2141	NONHSAG047130	11540	NGS	Fujimoto et al. 2012	22634756	Tumor
7	52802419	1401	NONHSAG047596	36688	NGS	Fujimoto et al. 2012	22634756	Tumor
8	39200450	1771	NONHSAG050041	0	NGS	Fujimoto et al. 2012	22634756	Tumor
5	1275390	1835	TERT	0	NGS	Fujimoto et al. 2012	22634756	Tumor
5	1295172	1854	TERT	9	NGS	Fujimoto et al. 2012	22634756	Tumor
5	1293404	1828	TERT	0	NGS	Fujimoto et al. 2012	22634756	Tumor
20	30502092	2716	TTLL9	0	NGS	Fujimoto et al. 2012	22634756	Tumor
17	15412626	1675	TVP23C-CDRT4,TVP23C	0,0	NGS	Fujimoto et al. 2012	22634756	Tumor
12p			FR7	intronic	Alu-PCR	GozuacikD et al. 2001	11593432	Tumor
5p15.33			hTERT	5.2 kb upstream	Alu-PCR	GozuacikD et al. 2001	11593432	Tumor
5p 15.33			hTERT	10.8 kb upstream	Alu-PCR	GozuacikD et al. 2001	11593432	Tumor
20p12.3			MCM8	intronic	Alu-PCR	GozuacikD et al. 2001	11593432	Tumor
18p11.3			NMP84p	intronic	Alu-PCR	GozuacikD et al. 2001	11593432	Tumor
16p12.1			SERCA1	Third exon	Alu-PCR	GozuacikD et al. 2001	11593432	Tumor
Ip32.3			∝TRAP150	∝TRAP 150 gene	Alu-PCR	GozuacikD et al. 2001	11593432	Tumor
8	30611415	546	UBXN8	intron	Alu-PCR	JiangS et al.2012	22962577	Tumor
8	30614167	1292	UBXN8	intron	Alu-PCR	JiangS et al.2012	22962577	Tumor
12	113606808	1321	DDX54	intron	Alu-PCR	JiangS et al.2012	22962577	Tumor
10	114925588	1390	TCF7L2	exon	Alu-PCR	JiangS et al.2012	22962577	Tumor
19	14630475	1426	TECR	0-10kb	Alu-PCR	JiangS et al.2012	22962577	Tumor
4	35991775	1429	ARAP2	50-100kb	Alu-PCR	JiangS et al.2012	22962577	Tumor
7	7212747	1433	C1GALT1	<10kb	Alu-PCR	JiangS et al.2012	22962577	Tumor
6	111413970	1452	SLC16A10	intron	Alu-PCR	JiangS et al.2012	22962577	Tumor
4	190666407	1462		0-100kb	Alu-PCR	JiangS et al.2012	22962577	Tumor
6	50309423	1470		0-100kb	LM-PCR	JiangS et al.2012	22962577	Tumor
		1501		0-100kb	LM-PCR	JiangS et al.2012	22962577	Tumor
		1505		0-100kb	Alu-PCR	JiangS et al.2012	22962577	Tumor
		1552		0-100kb	LM-PCR	JiangS et al.2012	22962577	Tumor
9	34295985	1561	KIF24	intron	Alu-PCR	JiangS et al.2012	22962577	Tumor
2	116472918	1586	DPP10	intron	Alu-PCR	JiangS et al.2012	22962577	Tumor
4	100227882	1587	ADH1B	intron	Alu-PCR	JiangS et al.2012	22962577	Tumor
3	21147551	1592		0-100kb	LM-PCR	JiangS et al.2012	22962577	Tumor
		1595		0-100kb	Alu-PCR	JiangS et al.2012	22962577	Tumor
14	88387716	1600	GALC	10-50kb	LM-PCR	JiangS et al.2012	22962577	Tumor
		1604		0-100kb	Alu-PCR	JiangS et al.2012	22962577	Tumor
		1605		0-100kb	Alu-PCR	JiangS et al.2012	22962577	Tumor
9	43410389	1631		0-100kb	LM-PCR	JiangS et al.2012	22962577	Tumor
Y	17442168	1633		0-100kb	LM-PCR	JiangS et al.2012	22962577	Tumor
7	149467090	1654	ZNF467	intron	LM-PCR	JiangS et al.2012	22962577	Tumor
12	5951302	1664	ANO2	intron	LM-PCR	JiangS et al.2012	22962577	Tumor
17	47431923	1671	ZNF652	intron	Alu-PCR	JiangS et al.2012	22962577	Tumor
20	62597547	1679	ZNF512B	exon	LM-PCR	JiangS et al.2012	22962577	Tumor
14	92258480	1694	TC2N	intron	Alu-PCR	JiangS et al.2012	22962577	Tumor
5	1291059	1695	TERT	intron	LM-PCR	JiangS et al.2012	22962577	Tumor
		1698		0-100kb	LM-PCR	JiangS et al.2012	22962577	Tumor
		1698		0-100kb	LM-PCR	JiangS et al.2012	22962577	Tumor
		1702		0-100kb	Alu-PCR	JiangS et al.2012	22962577	Tumor
3	155498844	1703	C3orf33	intron	LM-PCR	JiangS et al.2012	22962577	Tumor
		1704		0-100kb	LM-PCR	JiangS et al.2012	22962577	Tumor
11	83163665	1705	DLG2	<10kb	Alu-PCR	JiangS et al.2012	22962577	Tumor
15	85449263	1712	SLC28A1	intron	Alu-PCR	JiangS et al.2012	22962577	Tumor
10	120628434	1717		0-100kb	Alu-PCR	JiangS et al.2012	22962577	Tumor
		1747		0-100kb	LM-PCR	JiangS et al.2012	22962577	Tumor
		1750		0-100kb	LM-PCR	JiangS et al.2012	22962577	Tumor
7	74614231	1755	GTF2IP1	intron	Alu-PCR	JiangS et al.2012	22962577	Tumor
1	63211678	1756	ATG4C	10-50kb	Alu-PCR	JiangS et al.2012	22962577	Tumor
14	92353742	1757	FBLN5	intron	Alu-PCR	JiangS et al.2012	22962577	Tumor
8	118467464	1783	MED30	50-100kb	LM-PCR	JiangS et al.2012	22962577	Tumor
US	ND	1783		0-100kb	LM-PCR	JiangS et al.2012	22962577	Tumor
15	31673527	1787	KLF13	<10kb	LM-PCR	JiangS et al.2012	22962577	Tumor
1	7735733	1789	CAMTA1	intron	LM-PCR	JiangS et al.2012	22962577	Tumor
2	216245759	1796	FN1	exon	LM-PCR	JiangS et al.2012	22962577	Tumor
		1802		0-100kb	LM-PCR	JiangS et al.2012	22962577	Tumor
8	9936640	1803	MSRA	intron	Alu-PCR	JiangS et al.2012	22962577	Tumor
1	178370986	1807	RASAL2	intron	LM-PCR	JiangS et al.2012	22962577	Tumor
7	6955583	1808	C7orf28B	50-100kb	Alu-PCR	JiangS et al.2012	22962577	Tumor
		1808		0-100kb	Alu-PCR	JiangS et al.2012	22962577	Tumor
		1815		0-100kb	Alu-PCR	JiangS et al.2012	22962577	Tumor
6	67876043	1817		0-100kb	LM-PCR	JiangS et al.2012	22962577	Tumor
1	47847259	1818	CMPK1	<10kb	Alu-PCR	JiangS et al.2012	22962577	Tumor
7	57446860	1818	MIR3147	10-50kb	LM-PCR	JiangS et al.2012	22962577	Tumor
17	16001031	1818	NCOR1	intron	LM-PCR	JiangS et al.2012	22962577	Tumor
3P21.1	50719003	1819	DOCK3	intron	LM-PCR	JiangS et al.2012	22962577	Tumor
10	109468840	1819		0-100kb	LM-PCR	JiangS et al.2012	22962577	Tumor
1	154239263	1819	UBAP2L	intron	Alu-PCR	JiangS et al.2012	22962577	Tumor
Y	28502748	1819		0-100kb	Alu-PCR	JiangS et al.2012	22962577	Tumor
15	60237451	1820	FOXB1	50-100kb	LM-PCR	JiangS et al.2012	22962577	Tumor
		1820		0-100kb	LM-PCR	JiangS et al.2012	22962577	Tumor
17	21536937	1820	C17orf51	50-100kb	LM-PCR	JiangS et al.2012	22962577	Tumor
3	1482111	1820	CNTN6	10-50kb	Alu-PCR	JiangS et al.2012	22962577	Tumor
3	1482107	1820	CNTN6	10-50kb	Alu-PCR	JiangS et al.2012	22962577	Tumor
14	91309115	1821	RPS6KA5	10-50kb	Alu-PCR	JiangS et al.2012	22962577	Tumor
5	1295758	1822	TERT	<10kb	LM-PCR	JiangS et al.2012	22962577	Tumor
21	20990375	1823		0-100kb	LM-PCR	JiangS et al.2012	22962577	Tumor
17	79004903	1823	FLJ90757	intron	LM-PCR	JiangS et al.2012	22962577	Tumor
4	74142783	1824	ANKRD17	10-50kb	LM-PCR	JiangS et al.2012	22962577	Tumor
1	26089496	1825	MAN1C1	exon	Alu-PCR	JiangS et al.2012	22962577	Tumor
1	173065047	1825	TNFSF18	10-50kb	LM-PCR	JiangS et al.2012	22962577	Tumor

Chromosome	Integration site in host genome	Integration site in virus genome	Gene (distance, bp)	Regions	Methods	Author	PMID	Sample
7	74113982	1826	GTF2I	intron	Alu-PCR	JiangS et al.2012	22962577	Tumor
4	103653592	1826	MANBA	intron	Alu-PCR	JiangS et al.2012	22962577	Tumor
9	139846185	1826	LCN12	<10kb	Alu-PCR	JiangS et al.2012	22962577	Tumor
9	139845988	1826	LCN12	<10kb	Alu-PCR	JiangS et al.2012	22962577	Tumor
12	51565194	1826	TFCP2	intron	Alu-PCR	JiangS et al.2012	22962577	Tumor
11	61874693	1826		0-100kb	Alu-PCR	JiangS et al.2012	22962577	Tumor
		1826		0-100kb	Alu-PCR	JiangS et al.2012	22962577	Tumor
9	139300273	1826	SDCCAG3	intron	Alu-PCR	JiangS et al.2012	22962577	Tumor
14	76997666	1826	ESRRB	10-50kb	LM-PCR	JiangS et al.2012	22962577	Tumor
9	26341802	1826		0-100kb	LM-PCR	JiangS et al.2012	22962577	Tumor
3	113632087	1827	GRAMD1C	intron	Alu-PCR	JiangS et al.2012	22962577	Tumor
3	182599673	1827	ATP11B	intron	Alu-PCR	JiangS et al.2012	22962577	Tumor
12	113606823	1832	DDX54	intron	Alu-PCR	JiangS et al.2012	22962577	Tumor
5	1240477	1834	SLC6A18	intron	LM-PCR	JiangS et al.2012	22962577	Tumor
16	89385728	1837	ANKRD11	intron	LM-PCR	JiangS et al.2012	22962577	Tumor
18	56110442	1838	MIR122	<10kb	Alu-PCR	JiangS et al.2012	22962577	Tumor
4	155171488	1838	DCHS2	intron	Alu-PCR	JiangS et al.2012	22962577	Tumor
11	64200314	1840	MIR1237	50-100kb	Alu-PCR	JiangS et al.2012	22962577	Tumor
9	136346366	1840	SLC2A6	<10kb	Alu-PCR	JiangS et al.2012	22962577	Tumor
9	136346366	1840	SLC2A6	<10kb	Alu-PCR	JiangS et al.2012	22962577	Tumor
10	7793620	1844	KIN	<10kb	LM-PCR	JiangS et al.2012	22962577	Tumor
5	99384998	1845		0-100kb	LM-PCR	JiangS et al.2012	22962577	Tumor
		1847		0-100kb	LM-PCR	JiangS et al.2012	22962577	Tumor
10	85767043	1860		0-100kb	Alu-PCR	JiangS et al.2012	22962577	Tumor
2	86622425	1868	KDM3A	10-50kb	Alu-PCR	JiangS et al.2012	22962577	Tumor
3	39026094	1877	SCN11A	10-50kb	Alu-PCR	JiangS et al.2012	22962577	Tumor
2	216251299	1940	FN1	exon	Alu-PCR	JiangS et al.2012	22962577	Tumor
		1792		0-100kb	Alu-PCR	JiangS et al.2012	22962577	Tumor
chr18	74177432	2817	ZNF516		NGS	JiangZ et al.2012	22267523	Tumor
chr5	178498276	1978	ZNF354C		NGS	JiangZ et al.2012	22267523	Tumor
chr11	32458297	3193	WT1-AS		NGS	JiangZ et al.2012	22267523	Tumor
chr18	9346022	357	TWSG1		NGS	JiangZ et al.2012	22267523	Tumor
chr8	145667697	724	TONSL		NGS	JiangZ et al.2012	22267523	Tumor
chr17	16846020	1256	TNFRSF13B		NGS	JiangZ et al.2012	22267523	Tumor
chr11	9302969	1389	TMEM41B		NGS	JiangZ et al.2012	22267523	Tumor
chr19	36538198	809	THAP8		NGS	JiangZ et al.2012	22267523	Tumor
chr4	106997742	1362	TBCK		NGS	JiangZ et al.2012	22267523	Tumor
chr3	10306019	2998	TATDN2		NGS	JiangZ et al.2012	22267523	Tumor
14	64506127	1824	SYNE2		NGS	JiangZ et al.2012	22267523	Tumor
chr12	64264301	1681	SRGAP1		NGS	JiangZ et al.2012	22267523	Tumor
chr3	43366166	503	SNRK		NGS	JiangZ et al.2012	22267523	Tumor
chr21	38107174	3133	SIM2		NGS	JiangZ et al.2012	22267523	Tumor
chr16	13250153	2309	SHISA9		NGS	JiangZ et al.2012	22267523	Tumor
chr6	72677722	1665	RIMS1		NGS	JiangZ et al.2012	22267523	Tumor
10	95351812	411	RBP4		NGS	JiangZ et al.2012	22267523	Tumor
chr5	119980115	2410	PRR16		NGS	JiangZ et al.2012	22267523	Tumor
chr1	84544386	1077	PRKACB		NGS	JiangZ et al.2012	22267523	Tumor
chr1	204235863	1194	PLEKHA6		NGS	JiangZ et al.2012	22267523	Tumor
chr11	3171133	2995	OSBPL5		NGS	JiangZ et al.2012	22267523	Tumor
chrX	123884874	1825	ODZ1		NGS	JiangZ et al.2012	22267523	Tumor
chr14	33482200	1101	NPAS3		NGS	JiangZ et al.2012	22267523	Tumor
chr18	55831619	2650	NEDD4L		NGS	JiangZ et al.2012	22267523	Tumor
chr13	52418625	2551	NCRNA00282		NGS	JiangZ et al.2012	22267523	Tumor
chr12	62949812	1631	MON2		NGS	JiangZ et al.2012	22267523	Tumor
chr19	36212638	1820	KMT2B		NGS	JiangZ et al.2012	22267523	Tumor
19	36212643	1826	KMT2B		NGS	JiangZ et al.2012	22267523	Tumor
chr3	169328692	592	MECOM		NGS	JiangZ et al.2012	22267523	Tumor
chr3	182810141	2758	MCCC1		NGS	JiangZ et al.2012	22267523	Tumor
chr8	86053216	1381	LRRCC1		NGS	JiangZ et al.2012	22267523	Tumor
chr2	141269654	1910	LRP1B		NGS	JiangZ et al.2012	22267523	Tumor
chr4	62501555	1581	LPHN3		NGS	JiangZ et al.2012	22267523	Tumor
chr16	48311098	494	LONP2		NGS	JiangZ et al.2012	22267523	Tumor
3	156807727	1757	LOC100498859		NGS	JiangZ et al.2012	22267523	Tumor
chr2	178244885	1809	LOC100130691		NGS	JiangZ et al.2012	22267523	Tumor
chr16	11660021	1483	LITAF		NGS	JiangZ et al.2012	22267523	Tumor
chr12	118260607	1469	KSR2		NGS	JiangZ et al.2012	22267523	Tumor
chr19	51520093	2758	KLK10		NGS	JiangZ et al.2012	22267523	Tumor
4	123168278	1764	KIAA1109		NGS	JiangZ et al.2012	22267523	Tumor
chr13	47470747	468	HTR2A		NGS	JiangZ et al.2012	22267523	Tumor
chr20	23350770	2624	GZF1		NGS	JiangZ et al.2012	22267523	Tumor
chr2	230812102	2879	FBXO36		NGS	JiangZ et al.2012	22267523	Tumor
chr12	93231381	2214	EEA1		NGS	JiangZ et al.2012	22267523	Tumor
chr11	9207420	552	DENND5A		NGS	JiangZ et al.2012	22267523	Tumor
chr12	57940723	2572	DCTN2		NGS	JiangZ et al.2012	22267523	Tumor
chr5	177713646	2143	COL23A1		NGS	JiangZ et al.2012	22267523	Tumor
chr9	101797435	3210	COL15A1		NGS	JiangZ et al.2012	22267523	Tumor
chr20	60027226	1095	CDH4		NGS	JiangZ et al.2012	22267523	Tumor
chr3	122093822	1608	CCDC58		NGS	JiangZ et al.2012	22267523	Tumor
11	57509323	518	C11orf31		NGS	JiangZ et al.2012	22267523	Tumor
chr3	49646406	1546	BSN		NGS	JiangZ et al.2012	22267523	Tumor
chr15	83924783	2084	BNC1		NGS	JiangZ et al.2012	22267523	Tumor
chr2	203271359	1340	BMPR2		NGS	JiangZ et al.2012	22267523	Tumor
chr7	138471957	518	ATP6V0A4		NGS	JiangZ et al.2012	22267523	Tumor
1	150825750	1812	ARNT		NGS	JiangZ et al.2012	22267523	Tumor
1	161192294	1067	APOA2		NGS	JiangZ et al.2012	22267523	Tumor
9	104184149	1640	ALDOB		NGS	JiangZ et al.2012	22267523	Tumor
chr15	87313495	3189	AGBL1		NGS	JiangZ et al.2012	22267523	Tumor
chr3	38509429	1434	ACVR2B		NGS	JiangZ et al.2012	22267523	Tumor
chr7	57904573	538	ZNF716(371308）		NGS	JiangZ et al.2012	22267523	Tumor
chr19	58300895	1430	ZNF586(8911）,ZNF552(17554）		NGS	JiangZ et al.2012	22267523	Tumor
chrX	6629992	638	VCX3A(176833）,HDHD1(336968）		NGS	JiangZ et al.2012	22267523	Tumor
chr4	190943675	1801	LOC100288255(1847）		NGS	JiangZ et al.2012	22267523	Tumor
chr16	90003219	214	TUBB3(714）,DEF8(11931）		NGS	JiangZ et al.2012	22267523	Tumor
chr19	49767939	633	TRPM4(52841）,SLC6A16(24952）		NGS	JiangZ et al.2012	22267523	Tumor

Chromosome	Integration site in host genome	Integration site in virus genome	Gene (distance, bp)	Regions	Methods	Author	PMID	Sample
chr2	120491911	655	TMEM177(52217）,PTPN4(25295）		NGS	JiangZ et al.2012	22267523	Tumor
chr2	135500630	496	TMEM163(24059）,ACMSD(95555）		NGS	JiangZ et al.2012	22267523	Tumor
chr15	70679616	913	TLE3(289360）,UACA(267276）		NGS	JiangZ et al.2012	22267523	Tumor
chr22	33298914	1799	TIMP3(39886）,LARGE(370147）		NGS	JiangZ et al.2012	22267523	Tumor
chr10	133233840	270	TCERG1L(123856）		NGS	JiangZ et al.2012	22267523	Tumor
chr15	92344374	472	SV2B(499835）,SLCO3A1(52563)		NGS	JiangZ et al.2012	22267523	Tumor
chrX	154003272	1212	SNORA36A(6340),MPP1(3686)		NGS	JiangZ et al.2012	22267523	Tumor
chrX	128662871	2966	SMARCA1(5411),OCRL(11380)		NGS	JiangZ et al.2012	22267523	Tumor
chr11	116987018	1555	SIK3(18025),PAFAH1B2(27981)		NGS	JiangZ et al.2012	22267523	Tumor
chr10	81710117	200	SFTPD(1256),LOC219347(95871)		NGS	JiangZ et al.2012	22267523	Tumor
chr5	80539780	1422	RASGRF2(17827),ZCCHC9(57621)		NGS	JiangZ et al.2012	22267523	Tumor
chr13	98347349	152	RAP2A(227097),IPO5(258579）		NGS	JiangZ et al.2012	22267523	Tumor
chr20	20999162	2791	RALGAPA2(305896）,PLK1S1(107461）		NGS	JiangZ et al.2012	22267523	Tumor
chr20	44554233	162	PLTP(13447）,PCIF1(9083)		NGS	JiangZ et al.2012	22267523	Tumor
chr16	57326953	1148	PLLP(8369),CCL22(65741)		NGS	JiangZ et al.2012	22267523	Tumor
chr12	33139135	1852	PKP2(89355),SYT10(389212)		NGS	JiangZ et al.2012	22267523	Tumor
chr7	127554049	2908	PAX4(298269),C7orf54(83512)		NGS	JiangZ et al.2012	22267523	Tumor
chr3	152577625	1268	P2RY1(21782),RAP2B(302403)		NGS	JiangZ et al.2012	22267523	Tumor
chr19	14946842	1047	OR7A5(7566),OR7A10(4917)		NGS	JiangZ et al.2012	22267523	Tumor
chr20	62775846	702	NPBWR2(37662),MYT1(19980)		NGS	JiangZ et al.2012	22267523	Tumor
chr3	116581304	1179	NCRNA00295(145417)		NGS	JiangZ et al.2012	22267523	Tumor
chr8	47795569	1773	NCRNA00293(28162),LOC100287846(305360)		NGS	JiangZ et al.2012	22267523	Tumor
chr15	88308333	2580	NCRNA00052(185416),NTRK3(111654)		NGS	JiangZ et al.2012	22267523	Tumor
chr17	22215662	220	MTRNR2L1(191671)		NGS	JiangZ et al.2012	22267523	Tumor
chr2	105839288	979	MRPS9(122870),GPR45(18911)		NGS	JiangZ et al.2012	22267523	Tumor
chr17	2309247	973	MNT(4989）,LOC284009(1027)		NGS	JiangZ et al.2012	22267523	Tumor
chr2	109810265	2941	MIR4265(52221）,MIR4266(119761)		NGS	JiangZ et al.2012	22267523	Tumor
chr5	163144289	823	MAT2B(197961）		NGS	JiangZ et al.2012	22267523	Tumor
chr1	219607157	230	LYPLAL1(220950）,SLC30A10(480448）		NGS	JiangZ et al.2012	22267523	Tumor
chr2	80627472	336	LRRTM1(95985）		NGS	JiangZ et al.2012	22267523	Tumor
chr2	139936648	1284	LOC647012(279904)		NGS	JiangZ et al.2012	22267523	Tumor
chr5	17430428	866	LOC401177(43009)		NGS	JiangZ et al.2012	22267523	Tumor
chr2	42179208	1307	LOC388942(58022),PKDCC(95952)		NGS	JiangZ et al.2012	22267523	Tumor
chr12	11740616	13	LOC338817(23281),ETV6(62171)		NGS	JiangZ et al.2012	22267523	Tumor
chr22	19672734	2803	LOC150185(118372),39329(29252)		NGS	JiangZ et al.2012	22267523	Tumor
chr13	19969828	2383	LOC100287114(48939),TPTE2(27190)		NGS	JiangZ et al.2012	22267523	Tumor
chr6	84964838	2970	KIAA1009(27503),TBX18(479318)		NGS	JiangZ et al.2012	22267523	Tumor
chr8	111042814	3064	KCNV1(55855)		NGS	JiangZ et al.2012	22267523	Tumor
chr8	37078086	155	KCNU1(284443),ZNF703(475214)		NGS	JiangZ et al.2012	22267523	Tumor
chr15	89336216	150	ISG20(137337),ACAN(10457)		NGS	JiangZ et al.2012	22267523	Tumor
chr5	90553996	705	GPR98(93963),ARRDC3(110544)		NGS	JiangZ et al.2012	22267523	Tumor
chr16	58472641	250	GINS3(32593),NDRG4(24907)		NGS	JiangZ et al.2012	22267523	Tumor
chr6	110196312	2103	FIG4(49678),GPR6(103985)		NGS	JiangZ et al.2012	22267523	Tumor
chr13	22465784	1049	FGF9(187144)		NGS	JiangZ et al.2012	22267523	Tumor
chr2	88952585	1620	EIF2AK3(25491),RPIA(38590)		NGS	JiangZ et al.2012	22267523	Tumor
chr4	105576742	3185	CXXC4(164275),TET2(490289)		NGS	JiangZ et al.2012	22267523	Tumor
chr20	31332821	1479	COMMD7(1007),DNMT3B(17369)		NGS	JiangZ et al.2012	22267523	Tumor
chr6	25139395	321	CMAH(775),LRRC16A(140260)		NGS	JiangZ et al.2012	22267523	Tumor
chr4	17551674	1509	CLRN2(22947),LAP3(27252)		NGS	JiangZ et al.2012	22267523	Tumor
chr16	78128721	2338	CLEC3A(62723),WWOX(4829)		NGS	JiangZ et al.2012	22267523	Tumor
chr22	19548532	20	CLDN5(35672),LOC150185(5120)		NGS	JiangZ et al.2012	22267523	Tumor
chr8	74082529	1337	C8orf84(77022),LOC100130301(71034)		NGS	JiangZ et al.2012	22267523	Tumor
chr5	102631150	871	C5orf30(16789),NUDT12(253405)		NGS	JiangZ et al.2012	22267523	Tumor
chr13	47095920	3025	C13orf18(134285),LRCH1(31375)		NGS	JiangZ et al.2012	22267523	Tumor
chr12	13667736	1387	C12orf36(138057),GRIN2B(46673)		NGS	JiangZ et al.2012	22267523	Tumor
chr5	72817366	1814	BTF3(15918),ANKRA2(30658)		NGS	JiangZ et al.2012	22267523	Tumor
chr13	107511485	406	ARGLU1(290971),FAM155A(309393)		NGS	JiangZ et al.2012	22267523	Tumor
chr8	108520648	1824	ANGPT1(10394),RSPO2(390895)		NGS	JiangZ et al.2012	22267523	Tumor
chr16	67525552	1619	AGRP(7836),FAM65A(37164)		NGS	JiangZ et al.2012	22267523	Tumor
chr4	171361840	1904	AADAT(350468),HSP90AA6P(140780)		NGS	JiangZ et al.2012	22267523	Tumor
chr17	32027932	3189	AA06(167177)		NGS	JiangZ et al.2012	22267523	Tumor
chr9	88144000	1306	AGTPBP1(17453)		NGS	JiangZ et al.2012	22267523	Tumor
chr16	46427790	786	ANKRD26P1(75458)		NGS	JiangZ et al.2012	22267523	Tumor
chr16	46428634	890	ANKRD26P1(74614)		NGS	JiangZ et al.2012	22267523	Tumor
chr20	11601261	1917	BTBD3(270215)		NGS	JiangZ et al.2012	22267523	Tumor
chr11	104641759	1830	CASP12(114685)		NGS	JiangZ et al.2012	22267523	Tumor
chr7	145730834	3077	CNTNAP2(82618)		NGS	JiangZ et al.2012	22267523	Tumor
chr7	145810089	177	CNTNAP2(3363)		NGS	JiangZ et al.2012	22267523	Tumor
chr14	22803629	2499	DAD1(230177)		NGS	JiangZ et al.2012	22267523	Tumor
chr5	118132717	2970	DTWD2(39851)		NGS	JiangZ et al.2012	22267523	Tumor
chrX	122289910	626	GRIA3(28185)		NGS	JiangZ et al.2012	22267523	Tumor
chr5	21321786	1765	GUSBP1(137802)		NGS	JiangZ et al.2012	22267523	Tumor
chr18	38886165	1581	KC6(174070)		NGS	JiangZ et al.2012	22267523	Tumor
chr8	65028375	2094	LOC100130155(257399)		NGS	JiangZ et al.2012	22267523	Tumor
chr2	108124657	1610	LOC729121(314862)		NGS	JiangZ et al.2012	22267523	Tumor
chr5	67508314	1871	PIK3R1(14147)		NGS	JiangZ et al.2012	22267523	Tumor
chr14	29829655	1720	PRKD1(216031)		NGS	JiangZ et al.2012	22267523	Tumor
chr16	6006462	1029	RBFOX1(62669)		NGS	JiangZ et al.2012	22267523	Tumor
chr1	191893651	1415	RGS18(233940)		NGS	JiangZ et al.2012	22267523	Tumor
chr4	64905157	1787	TECRL(239019)		NGS	JiangZ et al.2012	22267523	Tumor
chr7	47261170	1087	TNS3(53581)		NGS	JiangZ et al.2012	22267523	Tumor
chr12	34843277	413			NGS	JiangZ et al.2012	22267523	Tumor
chr2	89868269	2261			NGS	JiangZ et al.2012	22267523	Tumor
chr3	117450691	3			NGS	JiangZ et al.2012	22267523	Tumor
chr3	117661703	371			NGS	JiangZ et al.2012	22267523	Tumor
chr8	115564013	1563			NGS	JiangZ et al.2012	22267523	Tumor
chr7	125266438	1932			NGS	JiangZ et al.2012	22267523	Tumor
chr13	85662622	1255			NGS	JiangZ et al.2012	22267523	Tumor
chr6	165006651	734			NGS	JiangZ et al.2012	22267523	Tumor
chr12	38029560	1571			NGS	JiangZ et al.2012	22267523	Tumor
chr16	51909154	1574			NGS	JiangZ et al.2012	22267523	Tumor
11	104641790	1826			NGS	JiangZ et al.2012	22267523	Tumor
2	80252050	1822			NGS	JiangZ et al.2012	22267523	Tumor
18	3028907	1786			NGS	JiangZ et al.2012	22267523	Tumor

Chromosome	Integration site in host genome	Integration site in virus genome	Gene (distance, bp)	Regions	Methods	Author	PMID	Sample
13	63982129	1782			NGS	JiangZ et al.2012	22267523	Tumor
2	200072326	1804			NGS	JiangZ et al.2012	22267523	Tumor
4	137905378	1588			NGS	JiangZ et al.2012	22267523	Tumor
14	29829658	1721			NGS	JiangZ et al.2012	22267523	Tumor
3	152577585	3209			NGS	JiangZ et al.2012	22267523	Tumor
3	152588168	1530			NGS	JiangZ et al.2012	22267523	Tumor
16	51909115	1582			NGS	JiangZ et al.2012	22267523	Tumor
3	22616612	1810			NGS	JiangZ et al.2012	22267523	Tumor
11	95324517	1803			NGS	JiangZ et al.2012	22267523	Tumor
17	69180952	1826			NGS	JiangZ et al.2012	22267523	Tumor
19	14946840	932			NGS	JiangZ et al.2012	22267523	Tumor
3	152561718	2471			NGS	JiangZ et al.2012	22267523	Tumor
3	191715395	1731			NGS	JiangZ et al.2012	22267523	Tumor
7	127731893	1548			NGS	JiangZ et al.2012	22267523	Tumor
16	51906367	458			NGS	JiangZ et al.2012	22267523	Tumor
7	79034718	1806	MAGI2	intron	ViralFusionSeq	Kawai-Kitahata et al. 2016	26553052	Tumor
7	79034752	187	MAGI2	intron	ViralFusionSeq	Kawai-Kitahata et al. 2016	26553052	Tumor
7	143440443	1939	FAM115C	intron	ViralFusionSeq	Kawai-Kitahata et al. 2016	26553052	Tumor
5	1295478	3027	TERT	promoter	ViralFusionSeq	Kawai-Kitahata et al. 2016	26553052	Tumor
18	55739297	1486	NEDD4L	intron	ViralFusionSeq	Kawai-Kitahata et al. 2016	26553052	Tumor
18	55740106	2134	NEDD4L	intron	ViralFusionSeq	Kawai-Kitahata et al. 2016	26553052	Tumor
8	31613530	1828	NRG1	intron	ViralFusionSeq	Kawai-Kitahata et al. 2016	26553052	Tumor
X	97378582	1820		intergenic	ViralFusionSeq	Kawai-Kitahata et al. 2016	26553052	Tumor
5	1296199	1591	TERT	promoter	ViralFusionSeq	Kawai-Kitahata et al. 2016	26553052	Tumor
5	1295107	2655	TERT	promoter	ViralFusionSeq	Kawai-Kitahata et al. 2016	26553052	Tumor
13	111629371	517		intergenic	ViralFusionSeq	Kawai-Kitahata et al. 2016	26553052	Tumor
13	111524996	827		intergenic	HIVID	Kawai-Kitahata et al. 2016	26553052	Tumor
4	74371982	1748		intergenic	HIVID	Kawai-Kitahata et al. 2016	26553052	Tumor
4	74371982	2615		intergenic	HIVID	Kawai-Kitahata et al. 2016	26553052	Tumor
12	77698791	3054		intergenic	HIVID	Kawai-Kitahata et al. 2016	26553052	Tumor
19	36213178	1790	KMT2B	intron	HIVID	Kawai-Kitahata et al. 2016	26553052	Tumor
19	36213139	2649	KMT2B	intron	HIVID	Kawai-Kitahata et al. 2016	26553052	Tumor
1	38731528	2896		intergenic	HIVID	Kawai-Kitahata et al. 2016	26553052	Tumor
12	77694305	1784		intergenic	HIVID	Kawai-Kitahata et al. 2016	26553052	Tumor
15	97680556	1800		intergenic	HIVID	Kawai-Kitahata et al. 2016	26553052	Tumor
10	42386937	2320		intergenic	HIVID	Kawai-Kitahata et al. 2016	26553052	Tumor
5	16747	3042		intergenic	HIVID	Kawai-Kitahata et al. 2016	26553052	Tumor
5	1296234	1813	TERT	promoter	HIVID	Kawai-Kitahata et al. 2016	26553052	Tumor
3	175538091	1759		intergenic	HIVID	Kawai-Kitahata et al. 2016	26553052	Tumor
9	10680766	639		intergenic	HIVID	Kawai-Kitahata et al. 2016	26553052	Tumor
13	105978745	1996		intergenic	HIVID	Kawai-Kitahata et al. 2016	26553052	Tumor
17	10365133	1809	AK097500	intron	HIVID	Kawai-Kitahata et al. 2016	26553052	Tumor
4	127242302	827		intergenic	HIVID	Kawai-Kitahata et al. 2016	26553052	Tumor
20	59652277	2912		intergenic	HIVID	Kawai-Kitahata et al. 2016	26553052	Tumor
2	128186261	1456	PROC	exon	HIVID	Kawai-Kitahata et al. 2016	26553052	Tumor
4	74281973	2882	ALB	exon	HIVID	Kawai-Kitahata et al. 2016	26553052	Tumor
4	60802746	2512		intergenic	HIVID	Kawai-Kitahata et al. 2016	26553052	Tumor
9	32948812	2053		intergenic	HIVID	Kawai-Kitahata et al. 2016	26553052	Tumor
5	1296164	1001	TERT	promoter	HIVID	Kawai-Kitahata et al. 2016	26553052	Tumor
16	83128248	311	CDH13	intron	HIVID	Kawai-Kitahata et al. 2016	26553052	Tumor
1	121484075	487		intergenic	HIVID	Kawai-Kitahata et al. 2016	26553052	Tumor
1	104518984	2174		intergenic	HIVID	Kawai-Kitahata et al. 2016	26553052	Tumor
1	104518943	1590		intergenic	HIVID	Kawai-Kitahata et al. 2016	26553052	Tumor
6	104377406	1652		intergenic	HIVID	Kawai-Kitahata et al. 2016	26553052	Tumor
6	104377406	1582		intergenic	HIVID	Kawai-Kitahata et al. 2016	26553052	Tumor
5	1295166	1826	TERT	promoter	HIVID	Kawai-Kitahata et al. 2016	26553052	Tumor
5	1295198	2457	TERT	promoter	HIVID	Kawai-Kitahata et al. 2016	26553052	Tumor
5	136808817	1527	SPOCK1	intergenic	HIVID	Kawai-Kitahata et al. 2016	26553052	Tumor
5	136810801	2922	SPOCK1	intron	HIVID	Kawai-Kitahata et al. 2016	26553052	Tumor
14	94713666	1576	PPP4R4	intron	HIVID	Kawai-Kitahata et al. 2016	26553052	Tumor
14	94890983	2821		intergenic	HIVID	Kawai-Kitahata et al. 2016	26553052	Tumor
22	17503843	1798		intergenic	HIVID	Kawai-Kitahata et al. 2016	26553052	Tumor
22	17503830	1867		intergenic	HIVID	Kawai-Kitahata et al. 2016	26553052	Tumor
5	1276338	1826	TERT	intron	HIVID	Kawai-Kitahata et al. 2016	26553052	Tumor
5	1276301	2409	TERT	intron	HIVID	Kawai-Kitahata et al. 2016	26553052	Tumor
4	71457557	536		intergenic	HIVID	Kawai-Kitahata et al. 2016	26553052	Tumor
4	57252415	536	AASDH	intron	HIVID	Kawai-Kitahata et al. 2016	26553052	Tumor
		428			HIVID	Kawai-Kitahata et al. 2016	26553052	Tumor
18	246298	3063	THOC1	intron	HIVID	Kawai-Kitahata et al. 2016	26553052	Tumor
18	246320	709	THOC1	intron	HIVID	Kawai-Kitahata et al. 2016	26553052	Tumor
4	2983521	46	GRK4	intron	HIVID	Kawai-Kitahata et al. 2016	26553052	Tumor
4	2983625	397	GRK4	intron	HIVID	Kawai-Kitahata et al. 2016	26553052	Tumor
11	76866114	1833	MYO7A	intron	HIVID	Kawai-Kitahata et al. 2016	26553052	Tumor
3	64946665	2977	ADAMTS9-AS2	intron	HIVID	Kawai-Kitahata et al. 2016	26553052	Tumor
3	64946657	1568	ADAMTS9-AS2	intron	HIVID	Kawai-Kitahata et al. 2016	26553052	Tumor
18	68008348	1096		intergenic	HIVID	Kawai-Kitahata et al. 2016	26553052	Tumor
5	128745514	980		intergenic	HIVID	Kawai-Kitahata et al. 2016	26553052	Tumor
5	128745585	2662		intergenic	HIVID	Kawai-Kitahata et al. 2016	26553052	Tumor
17	25518202	2072		intergenic	HIVID	Kawai-Kitahata et al. 2016	26553052	Tumor
4	55950831	1790	KDR	intron	HIVID	Kawai-Kitahata et al. 2016	26553052	Tumor
4	55950887	765	KDR	intron	HIVID	Kawai-Kitahata et al. 2016	26553052	Tumor
5	1295162	1817	TERT	promoter	HIVID	Kawai-Kitahata et al. 2016	26553052	Tumor
5	1295202	139	TERT	promoter	HIVID	Kawai-Kitahata et al. 2016	26553052	Tumor
		2702			HIVID	Kawai-Kitahata et al. 2016	26553052	Tumor
3	74540169	394	CNTN3	intron	HIVID	Kawai-Kitahata et al. 2016	26553052	Tumor
11	80311251	2932		intergenic	HIVID	Kawai-Kitahata et al. 2016	26553052	Tumor
18	76175268	2001		intergenic	HIVID	Kawai-Kitahata et al. 2016	26553052	Tumor
		2030			HIVID	Kawai-Kitahata et al. 2016	26553052	Tumor
18	76175387	2001		intergenic	HIVID	Kawai-Kitahata et al. 2016	26553052	Tumor
		2030			HIVID	Kawai-Kitahata et al. 2016	26553052	Tumor
8	50329058	1780		intergenic	HIVID	Kawai-Kitahata et al. 2016	26553052	Tumor
2	31749116	1825		intergenic	HIVID	Kawai-Kitahata et al. 2016	26553052	Tumor
6	117647569	1313	ROS1	exon	HIVID	Kawai-Kitahata et al. 2016	26553052	Tumor
6	117647556	3083	ROS1	exon	HIVID	Kawai-Kitahata et al. 2016	26553052	Tumor

Chromosome	Integration site in host genome	Integration site in virus genome	Gene (distance, bp)	Regions	Methods	Author	PMID	Sample
2	32069716	3215		intergenic	HIVID	Kawai-Kitahata et al. 2016	26553052	Tumor
5	12579652	1429	CT49	intron	HIVID	Kawai-Kitahata et al. 2016	26553052	Tumor
17	31149419	806	MYO1D	intron	HIVID	Kawai-Kitahata et al. 2016	26553052	Tumor
19	24184912	520		intergenic	HIVID	Kawai-Kitahata et al. 2016	26553052	Tumor
14	48318506	1846		intergenic	HIVID	Kawai-Kitahata et al. 2016	26553052	Tumor
14	48318483	1827		intergenic	HIVID	Kawai-Kitahata et al. 2016	26553052	Tumor
12	127650953	405		intergenic	HIVID	Kawai-Kitahata et al. 2016	26553052	Tumor
5	1295400	1814	TERT	promoter	HIVID	Kawai-Kitahata et al. 2016	26553052	Tumor
5	1297376	2405	TERT	promoter	HIVID	Kawai-Kitahata et al. 2016	26553052	Tumor
10	126131994	1034		intergenic	HIVID	Kawai-Kitahata et al. 2016	26553052	Tumor
10	127050274	1034		intergenic	HIVID	Kawai-Kitahata et al. 2016	26553052	Tumor
		950			HIVID	Kawai-Kitahata et al. 2016	26553052	Tumor
13	65439986	2134		intergenic	HIVID	Kawai-Kitahata et al. 2016	26553052	Tumor
5	24026194	656	AX747383	intron	HIVID	Kawai-Kitahata et al. 2016	26553052	Tumor
5	1295164	1826	TERT	promoter	HIVID	Kawai-Kitahata et al. 2016	26553052	Tumor
16	55937291	2443	CES5A	intron	HIVID	Kawai-Kitahata et al. 2016	26553052	Tumor
5	3175793	930		intergenic	HIVID	Kawai-Kitahata et al. 2016	26553052	Tumor
9	77140636	2163	RORB	intron	HIVID	Kawai-Kitahata et al. 2016	26553052	Tumor
		1950			HIVID	Kawai-Kitahata et al. 2016	26553052	Tumor
9	77028511	2163		intergenic	HIVID	Kawai-Kitahata et al. 2016	26553052	Tumor
		1950			HIVID	Kawai-Kitahata et al. 2016	26553052	Tumor
5	172942977	1006		intergenic	HIVID	Kawai-Kitahata et al. 2016	26553052	Tumor
5	132204239	2813	UQCRQ	exon	HIVID	Kawai-Kitahata et al. 2016	26553052	Tumor
8	120290505	541		intergenic	HIVID	Kawai-Kitahata et al. 2016	26553052	Tumor
5	146877873	2149	DPYSL3	intron	HIVID	Kawai-Kitahata et al. 2016	26553052	Tumor
19	36213273	3206	KMT2B; KMT2B	exon	HIVID	Kawai-Kitahata et al. 2016	26553052	Tumor
19	36213281	1341	KMT2B; KMT2B	exon	HIVID	Kawai-Kitahata et al. 2016	26553052	Tumor
8	92531878	2058		intergenic	HIVID	Kawai-Kitahata et al. 2016	26553052	Tumor
		2005			HIVID	Kawai-Kitahata et al. 2016	26553052	Tumor
8	92530549	2058		intergenic	HIVID	Kawai-Kitahata et al. 2016	26553052	Tumor
3	175538091	1760		intergenic	HIVID	Kawai-Kitahata et al. 2016	26553052	Tumor
1	199334825	2848		intergenic	HIVID	Kawai-Kitahata et al. 2016	26553052	Tumor
11	76863582	1833	MYO7A	intron	HIVID	Kawai-Kitahata et al. 2016	26553052	Tumor
8	96196712	1715		intergenic	HIVID	Kawai-Kitahata et al. 2016	26553052	Tumor
1	55317630	958	DHCR24	exon	HIVID	Kawai-Kitahata et al. 2016	26553052	Tumor
7	95654315	1524	DYNC1I1	intron	HIVID	Kawai-Kitahata et al. 2016	26553052	Tumor
17	64468192	1229	PRKCA	intron	HIVID	Kawai-Kitahata et al. 2016	26553052	Tumor
5	1295113	124	TERT	exon	HIVID	Kawai-Kitahata et al. 2016	26553052	Tumor
5	1295153	1909	TERT	exon	HIVID	Kawai-Kitahata et al. 2016	26553052	Tumor
4	156837049	2595	TDO2	exon	HIVID	Kawai-Kitahata et al. 2016	26553052	Tumor
5	26231467	2693		intergenic	HIVID	Kawai-Kitahata et al. 2016	26553052	Tumor
X	109272781	3018		intergenic	HIVID	Kawai-Kitahata et al. 2016	26553052	Tumor
17	22022861	1986	MTRNR2L1	exon	HIVID	Kawai-Kitahata et al. 2016	26553052	Tumor
2	120397926	31	CFAP221	intron	HIVID	Kawai-Kitahata et al. 2016	26553052	Tumor
3	142676626	2220		intergenic	HIVID	Kawai-Kitahata et al. 2016	26553052	Tumor
8	73179518	2887		intergenic	HIVID	Kawai-Kitahata et al. 2016	26553052	Tumor
11	131585709	2266	NTM	intron	HIVID	Kawai-Kitahata et al. 2016	26553052	Tumor
6	9136966	2084		intergenic	HIVID	Kawai-Kitahata et al. 2016	26553052	Tumor
1	104028500	2255		intergenic	HIVID	Kawai-Kitahata et al. 2016	26553052	Tumor
17	22245465	2944		intergenic	HIVID	Kawai-Kitahata et al. 2016	26553052	Tumor
chr4	chr4:54908780-54915241(+)	467-711 (+)	CHIC2	*CHIC2* intron 3	HIVID	LauC et al.2014	24582836	Tumor
chr5	chr5:1295559-1295834 (+)	94-285 (+)	TERT	*TERT* upstream	HIVID	LauC et al.2014	24582836	Tumor
chr8	chr8:41493923-41494204 (+)	1539-1622 (+)	LINE1	LINE1 on chr8p11	HIVID	LauC et al.2014	24582836	Tumor
chr13	chr13:115070262-115070459	241-459 (+)	UPF3A	*UPF3A* exon 10	HIVID	LauC et al.2014	24582836	Tumor
	15bp partial homology to	1931-2190 (+)	RALGPS1	*RALGPS1* intron 3	HIVID	LauC et al.2014	24582836	Tumor
	68bp partial homology to	1212-1983 (+)	TRRAP	*TRRAP*	HIVID	LauC et al.2014	24582836	Tumor
chr16	chr16:30408209-30408356 (+)	1835-2021 (+)	ZNF48	*ZNF48* intron 2	HIVID	LauC et al.2014	24582836	Tumor
chr4	chr4:99949575-99949439 (-)	1671-1811 (+)	METAP1	*METAP1* intron 1	HIVID	LauC et al.2014	24582836	Tumor
chr11	chr11:68887645-68887577 (-)	1842-1936 (+)	LINE2	LINE2 on chr11q13	HIVID	LauC et al.2014	24582836	Tumor
chr11	chr11:14159090-14159048 (-)	1095-1164 (+)	TPCN2	*TPCN2* intron 21	HIVID	LauC et al.2014	24582836	Tumor
chr4	chr4:53063790-53063967 (+)	1805-1658 (-)	SINE	SINE on chr4q12	HIVID	LauC et al.2014	24582836	Tumor
chr1	3190799		PRDM16	Intron	HIVID	Li w et al.2013	23867110	Tumor
chr1	151503388		CGN	Intron	HIVID	Li w et al.2013	23867110	Tumor
chr1	151509176		CGN	Intron	HIVID	Li w et al.2013	23867110	Tumor
chr18	45256948				HIVID	Li w et al.2013	23867110	Tumor
chrX	14603545		GLRA2	Intron	HIVID	Li w et al.2013	23867110	Tumor
chrX	14603891		GLRA2	Intron	HIVID	Li w et al.2013	23867110	Tumor
chr19	54911046				HIVID	Li w et al.2013	23867110	Tumor
chr11	69687282				HIVID	Li w et al.2013	23867110	Tumor
chr11	69687305				HIVID	Li w et al.2013	23867110	Tumor
chr9	66971244				HIVID	Li w et al.2013	23867110	Tumor
chrX	144158308				HIVID	Li w et al.2013	23867110	Tumor
chr11	62425886		C11orf48	Promoter	HIVID	Li w et al.2013	23867110	Tumor
chr12	57280740				HIVID	Li w et al.2013	23867110	Tumor
chr12	57308160				HIVID	Li w et al.2013	23867110	Tumor
chr14	73560719		RBM25	Intron	HIVID	Li w et al.2013	23867110	Tumor
chr18	63841687				HIVID	Li w et al.2013	23867110	Tumor
chr19	19341549		NCAN	Intron	HIVID	Li w et al.2013	23867110	Tumor
chr19	19379792		TM6SF2	Intron	HIVID	Li w et al.2013	23867110	Tumor
chr19	30303499		CCNE1	Intron	HIVID	Li w et al.2013	23867110	Tumor
chr19	30303514		CCNE1	Intron	HIVID	Li w et al.2013	23867110	Tumor
chr5	158787933		LOC285626	ncRNA_Intronic	HIVID	Li w et al.2013	23867110	Tumor
chr5	158787954		LOC285626	ncRNA_Intronic	HIVID	Li w et al.2013	23867110	Tumor
chr6	40366232		LRFN2	Intron	HIVID	Li w et al.2013	23867110	Tumor
chr13	69086101				HIVID	Li w et al.2013	23867110	Tumor
chr2	15366500		NBAS	Intron	HIVID	Li w et al.2013	23867110	Tumor
chr2	15512471		NBAS	Intron	HIVID	Li w et al.2013	23867110	Tumor
chr19	36214007		KMT2B	CDS	HIVID	Li w et al.2013	23867110	Tumor
chr12	113741936		SLC24A6	Intron	HIVID	Li w et al.2013	23867110	Tumor
chr14	96085139				HIVID	Li w et al.2013	23867110	Tumor
chr14	96085213				HIVID	Li w et al.2013	23867110	Tumor
chr17	18287063		EVPLL	Intron	HIVID	Li w et al.2013	23867110	Tumor
chr17	74017304		EVPL	Intron	HIVID	Li w et al.2013	23867110	Tumor
chr19	30315005		CCNE1	3-UTR	HIVID	Li w et al.2013	23867110	Tumor

Chromosome	Integration site in host genome	Integration site in virus genome	Gene (distance, bp)	Regions	Methods	Author	PMID	Sample
chr19	30315366		CCNE1	Downstream	HIVID	Li w et al.2013	23867110	Tumor
chr13	88395490				HIVID	Li w et al.2013	23867110	Tumor
chr13	88395591				HIVID	Li w et al.2013	23867110	Tumor
chr13	91419424				HIVID	Li w et al.2013	23867110	Tumor
chr13	91419601				HIVID	Li w et al.2013	23867110	Tumor
chr13	91419633				HIVID	Li w et al.2013	23867110	Tumor
chr13	91419657				HIVID	Li w et al.2013	23867110	Tumor
chr16	89938592		SPIRE2	Downstream	HIVID	Li w et al.2013	23867110	Tumor
chr16	89938643		SPIRE2	Downstream	HIVID	Li w et al.2013	23867110	Tumor
chr2	198804023		PLCL1	Intron	HIVID	Li w et al.2013	23867110	Tumor
chr2	198804064		PLCL1	Intron	HIVID	Li w et al.2013	23867110	Tumor
chr20	13088550		SPTLC3	Intron	HIVID	Li w et al.2013	23867110	Tumor
chr20	13094195		SPTLC3	Intron	HIVID	Li w et al.2013	23867110	Tumor
chr5	103427837				HIVID	Li w et al.2013	23867110	Tumor
chr5	103429808				HIVID	Li w et al.2013	23867110	Tumor
chr3	192716140				HIVID	Li w et al.2013	23867110	Tumor
chr3	192716171				HIVID	Li w et al.2013	23867110	Tumor
chr5	1295410		TERT	Promoter	HIVID	Li w et al.2013	23867110	Tumor
chr5	1295463		TERT	Promoter	HIVID	Li w et al.2013	23867110	Tumor
chr18	107922		ROCK1P1	Promoter	HIVID	Li w et al.2013	23867110	Tumor
chr6	33827925				HIVID	Li w et al.2013	23867110	Tumor
chr20	47794908		STAU1	Intron	HIVID	Li w et al.2013	23867110	Tumor
chr8	60291544				HIVID	Li w et al.2013	23867110	Tumor
chr8	137398446				HIVID	Li w et al.2013	23867110	Tumor
chr16	46416617				HIVID	Li w et al.2013	23867110	Tumor
chr16	46425938				HIVID	Li w et al.2013	23867110	Tumor
chr8	34782289				HIVID	Li w et al.2013	23867110	Tumor
chr5	1297640				HIVID	Li w et al.2013	23867110	Tumor
chrX	145124068				HIVID	Li w et al.2013	23867110	Tumor
chr12	84442928				HIVID	Li w et al.2013	23867110	Tumor
chr12	120812094				HIVID	Li w et al.2013	23867110	Tumor
chr5	59252149		PDE4D	Intron	HIVID	Li w et al.2013	23867110	Tumor
chr10	42387797				HIVID	Li w et al.2013	23867110	Tumor
chr12	34372613				HIVID	Li w et al.2013	23867110	Tumor
chr12	34372684				HIVID	Li w et al.2013	23867110	Tumor
chr12	56868897		GLS2	Intron	HIVID	Li w et al.2013	23867110	Tumor
chr12	56869762		GLS2	Intron	HIVID	Li w et al.2013	23867110	Tumor
chr12	84251820				HIVID	Li w et al.2013	23867110	Tumor
chr13	49243137				HIVID	Li w et al.2013	23867110	Tumor
chr14	42967876				HIVID	Li w et al.2013	23867110	Tumor
chr3	7194529		GRM7	Intron	HIVID	Li w et al.2013	23867110	Tumor
chr3	196625712		SENP5	Intron	HIVID	Li w et al.2013	23867110	Tumor
chr5	17412293				HIVID	Li w et al.2013	23867110	Tumor
chr7	109739475				HIVID	Li w et al.2013	23867110	Tumor
chr7	109739516				HIVID	Li w et al.2013	23867110	Tumor
chr10	42387915				HIVID	Li w et al.2013	23867110	Tumor
chr19	36212741		KMT2B	Intron	HIVID	Li w et al.2013	23867110	Tumor
chr9	79323011		PRUNE2	CDS	HIVID	Li w et al.2013	23867110	Tumor
chr14	42989263				HIVID	Li w et al.2013	23867110	Tumor
chr7	134071835				HIVID	Li w et al.2013	23867110	Tumor
chr8	53342017				HIVID	Li w et al.2013	23867110	Tumor
chr1	164826325				HIVID	Li w et al.2013	23867110	Tumor
chr18	38204578				HIVID	Li w et al.2013	23867110	Tumor
chr21	11132388				HIVID	Li w et al.2013	23867110	Tumor
chr4	168808613				HIVID	Li w et al.2013	23867110	Tumor
chr5	1295178		TERT	Promoter	HIVID	Li w et al.2013	23867110	Tumor
chr5	1295339		TERT	Promoter	HIVID	Li w et al.2013	23867110	Tumor
chr5	1299593				HIVID	Li w et al.2013	23867110	Tumor
chr5	1298958				HIVID	Li w et al.2013	23867110	Tumor
chr16	49206000				HIVID	Li w et al.2013	23867110	Tumor
chr19	36212313		KMT2B	CDS	HIVID	Li w et al.2013	23867110	Tumor
chr2	110639781				HIVID	Li w et al.2013	23867110	Tumor
chr20	16043298				HIVID	Li w et al.2013	23867110	Tumor
chr4	143612477		INPP4B	Intron	HIVID	Li w et al.2013	23867110	Tumor
chr8	59068668				HIVID	Li w et al.2013	23867110	Tumor
chr14	36016829		RALGAPA1	Intron	HIVID	Li w et al.2013	23867110	Tumor
chr18	72836753				HIVID	Li w et al.2013	23867110	Tumor
chr18	72836897				HIVID	Li w et al.2013	23867110	Tumor
chr19	36213142		KMT2B	Intron	HIVID	Li w et al.2013	23867110	Tumor
chr5	159241641				HIVID	Li w et al.2013	23867110	Tumor
chr5	159412327				HIVID	Li w et al.2013	23867110	Tumor
chr8	69519928		C8orf34	Intron	HIVID	Li w et al.2013	23867110	Tumor
chr8	69523193		C8orf34	Intron	HIVID	Li w et al.2013	23867110	Tumor
chr1	118200608				HIVID	Li w et al.2013	23867110	Tumor
chr5	173929017				HIVID	Li w et al.2013	23867110	Tumor
chr6	30481166				HIVID	Li w et al.2013	23867110	Tumor
chr1	47404503		CYP4A11	Intron	HIVID	Li w et al.2013	23867110	Tumor
chr19	36212566		KMT2B	CDS	HIVID	Li w et al.2013	23867110	Tumor
chr8	101905732				HIVID	Li w et al.2013	23867110	Tumor
chrX	115777228				HIVID	Li w et al.2013	23867110	Tumor
chr1	10383		DDX11L1	Promoter	HIVID	Li w et al.2013	23867110	Tumor
chr12	82418				HIVID	Li w et al.2013	23867110	Tumor
chr19	62869		WASH5P	ncRNA_Intronic	HIVID	Li w et al.2013	23867110	Tumor
chr20	62918728		LINC00266-1	Promoter	HIVID	Li w et al.2013	23867110	Tumor
chrY	59363425				Alu-PCR	Li w et al.2013	23867110	Tumor
chr1	193632089				Alu-PCR	Li w et al.2013	23867110	Tumor
chr10	127005786				Alu-PCR	Li w et al.2013	23867110	Tumor
chr19	27923583				Alu-PCR	Li w et al.2013	23867110	Tumor
chr2	92310147				Alu-PCR	Li w et al.2013	23867110	Tumor
chr2	92313372				Alu-PCR	Li w et al.2013	23867110	Tumor
chr3	90450679				Alu-PCR	Li w et al.2013	23867110	Tumor
chr3	90450762				Alu-PCR	Li w et al.2013	23867110	Tumor
chr4	21872886		KCNIP4	Intron	Alu-PCR	Li w et al.2013	23867110	Tumor
chr9	69711099				Alu-PCR	Li w et al.2013	23867110	Tumor

Chromosome	Integration site in host genome	Integration site in virus genome	Gene (distance, bp)	Regions	Methods	Author	PMID	Sample
chr9	69711183				Alu-PCR	Li w et al.2013	23867110	Tumor
chrX	53179287				Alu-PCR	Li w et al.2013	23867110	Tumor
chrX	63450982		ASB12	Promoter	Alu-PCR	Li w et al.2013	23867110	Tumor
chr1	121485380				Alu-PCR	Li w et al.2013	23867110	Tumor
chr13	99639842		DOCK9	Intron	Alu-PCR	Li w et al.2013	23867110	Tumor
chr14	73450075		ZFYVE1	Intron	Alu-PCR	Li w et al.2013	23867110	Tumor
chrX	44801341		KDM6A	Intron	Alu-PCR	Li w et al.2013	23867110	Tumor
chr11	46180220				Alu-PCR	Li w et al.2013	23867110	Tumor
chr11	111827459		DIXDC1	Intron	Alu-PCR	Li w et al.2013	23867110	Tumor
chr11	118976122		C2CD2L	Promoter	Alu-PCR	Li w et al.2013	23867110	Tumor
chr17	18287138		EVPLL	Intron	Alu-PCR	Li w et al.2013	23867110	Tumor
chr17	29947476				Alu-PCR	Li w et al.2013	23867110	Tumor
chr17	74017380		EVPL	Intron	Alu-PCR	Li w et al.2013	23867110	Tumor
chr19	4165626		CREB3L3	Intron	Alu-PCR	Li w et al.2013	23867110	Tumor
chr2	234132145				Alu-PCR	Li w et al.2013	23867110	Tumor
chr3	42560005		VIPR1	Intron	Alu-PCR	Li w et al.2013	23867110	Tumor
chr6	93683084				Alu-PCR	Li w et al.2013	23867110	Tumor
chr12	38383335				Alu-PCR	Li w et al.2013	23867110	Tumor
chr12	38392271				Alu-PCR	Li w et al.2013	23867110	Tumor
chr12	38392321				Alu-PCR	Li w et al.2013	23867110	Tumor
chr12	21035362		SLCO1B3	Intron	Alu-PCR	Li w et al.2013	23867110	Tumor
chr15	57455366		TCF12	Intron	Alu-PCR	Li w et al.2013	23867110	Tumor
chr3	175781791				Alu-PCR	Li w et al.2013	23867110	Tumor
chr3	189864989				Alu-PCR	Li w et al.2013	23867110	Tumor
chr9	117939972		43800	Intron	Alu-PCR	Li w et al.2013	23867110	Tumor
chr3	140571184				Alu-PCR	Li w et al.2013	23867110	Tumor
chrY	13469129				Alu-PCR	Li w et al.2013	23867110	Tumor
chrY	13475776				Alu-PCR	Li w et al.2013	23867110	Tumor
chrY	13475871				Alu-PCR	Li w et al.2013	23867110	Tumor
chr1	18580850		IGSF21	Intron	Alu-PCR	Li w et al.2013	23867110	Tumor
chr12	95693				Alu-PCR	Li w et al.2013	23867110	Tumor
chr15	102521368				Alu-PCR	Li w et al.2013	23867110	Tumor
chr20	62918426				Alu-PCR	Li w et al.2013	23867110	Tumor
chr5	11580				Alu-PCR	Li w et al.2013	23867110	Tumor
chr8	43323328				Alu-PCR	Li w et al.2013	23867110	Tumor
chr17	14716384				Alu-PCR	Li w et al.2013	23867110	Tumor
chr17	51090043				Alu-PCR	Li w et al.2013	23867110	Tumor
chrX	115667429				Alu-PCR	Li w et al.2013	23867110	Tumor
chr5	1295375		TERT	Promoter	Alu-PCR	Li w et al.2013	23867110	Tumor
chr18	10111				Alu-PCR	Li w et al.2013	23867110	Tumor
chr4	191044087				Alu-PCR	Li w et al.2013	23867110	Tumor
chr5	1295121		TERT	5-UTR	Alu-PCR	Li w et al.2013	23867110	Tumor
chr10	18222385				Alu-PCR	Li w et al.2013	23867110	Tumor
chr10	42389516				Alu-PCR	Li w et al.2013	23867110	Tumor
chr10	42396415				Alu-PCR	Li w et al.2013	23867110	Tumor
chr10	42596862				Alu-PCR	Li w et al.2013	23867110	Tumor
chr10	42599291				Alu-PCR	Li w et al.2013	23867110	Tumor
chr12	129385352		GLT1D1	Intron	Alu-PCR	Li w et al.2013	23867110	Tumor
chr12	129385439		GLT1D1	Intron	Alu-PCR	Li w et al.2013	23867110	Tumor
chr4	76807187		PPEF2	Intron	Alu-PCR	Li w et al.2013	23867110	Tumor
chr4	76807283		PPEF2	Intron	Alu-PCR	Li w et al.2013	23867110	Tumor
chr5	52278431				Alu-PCR	Li w et al.2013	23867110	Tumor
chr5	103604810				Alu-PCR	Li w et al.2013	23867110	Tumor
chr7	154782383		PAXIP1	Intron	Alu-PCR	Li w et al.2013	23867110	Tumor
chrX	20156099		EIF1AX	Intron	Alu-PCR	Li w et al.2013	23867110	Tumor
chrX	20156148		EIF1AX	Intron	Alu-PCR	Li w et al.2013	23867110	Tumor
chr10	42387444				Alu-PCR	Li w et al.2013	23867110	Tumor
chr10	42388580				Alu-PCR	Li w et al.2013	23867110	Tumor
chr10	42391457				Alu-PCR	Li w et al.2013	23867110	Tumor
chr10	42596980				Alu-PCR	Li w et al.2013	23867110	Tumor
chr10	42598338				Alu-PCR	Li w et al.2013	23867110	Tumor
chr10	42599874				Alu-PCR	Li w et al.2013	23867110	Tumor
chr2	204666726				Alu-PCR	Li w et al.2013	23867110	Tumor
chr2	205395701				Alu-PCR	Li w et al.2013	23867110	Tumor
chr4	14065				Alu-PCR	Li w et al.2013	23867110	Tumor
chr4	191039677				Alu-PCR	Li w et al.2013	23867110	Tumor
chr8	43096000				Alu-PCR	Li w et al.2013	23867110	Tumor
chr8	43821906				Alu-PCR	Li w et al.2013	23867110	Tumor
chr1	5019596				Alu-PCR	Li w et al.2013	23867110	Tumor
chr1	121484075				Alu-PCR	Li w et al.2013	23867110	Tumor
chr1	121485094				Alu-PCR	Li w et al.2013	23867110	Tumor
chr19	27740218				Alu-PCR	Li w et al.2013	23867110	Tumor
chr5	1295295		TERT	Promoter	Alu-PCR	Li w et al.2013	23867110	Tumor
chr5	1307431				Alu-PCR	Li w et al.2013	23867110	Tumor
chr5	1295442		TERT	Promoter	Alu-PCR	Li w et al.2013	23867110	Tumor
chr2	110635802				Alu-PCR	Li w et al.2013	23867110	Tumor
chr2	111246881				Southern blot hybridization	Li w et al.2013	23867110	Tumor
chr2	111250860				Southern blot hybridization	Li w et al.2013	23867110	Tumor
chr2	113202130				Southern blot hybridization	Li w et al.2013	23867110	Tumor
chr1	10115		DDX11L1	Promoter	Alu-PCR	Li w et al.2013	23867110	Tumor
chr1	10216		DDX11L1	Promoter	Alu-PCR	Li w et al.2013	23867110	Tumor
chr1	18580849		IGSF21	Intron	Alu-PCR	Li w et al.2013	23867110	Tumor
chr1	249240292				Alu-PCR	Li w et al.2013	23867110	Tumor
chr10	42387234				Alu-PCR	Li w et al.2013	23867110	Tumor
chr10	42387705				Alu-PCR	Li w et al.2013	23867110	Tumor
chr10	42389892				Alu-PCR	Li w et al.2013	23867110	Tumor
chr10	42599664				Alu-PCR	Li w et al.2013	23867110	Tumor
chr10	96587309		CYP2C19	Intron	Alu-PCR	Li w et al.2013	23867110	Tumor
chr11	7199541				Alu-PCR	Li w et al.2013	23867110	Tumor
chr11	49749977		LOC440040	ncRNA_Intronic	Alu-PCR	Li w et al.2013	23867110	Tumor
chr16	33896652				Alu-PCR	Li w et al.2013	23867110	Tumor
chr17	17744113		TOM1L2	Promoter	Alu-PCR	Li w et al.2013	23867110	Tumor
chr20	25697180				Alu-PCR	Li w et al.2013	23867110	Tumor
chr7	36595481		AOAH	Intron	Alu-PCR	Li w et al.2013	23867110	Tumor

Chromosome	Integration site in host genome	Integration site in virus genome	Gene (distance, bp)	Regions	Methods	Author	PMID	Sample
chrX	154660457				Alu-PCR	Li w et al.2013	23867110	Tumor
chr2	89875220				Alu-PCR	Li w et al.2013	23867110	Tumor
chr2	89879870				Alu-PCR	Li w et al.2013	23867110	Tumor
chr2	89879976				Alu-PCR	Li w et al.2013	23867110	Tumor
chrY	13864642				Alu-PCR	Li w et al.2013	23867110	Tumor
chr1	10353		DDX11L1	Promoter	Alu-PCR	Li w et al.2013	23867110	Tumor
chr1	249240304				Alu-PCR	Li w et al.2013	23867110	Tumor
chr11	51575702				Alu-PCR	Li w et al.2013	23867110	Tumor
chr17	22261216				Alu-PCR	Li w et al.2013	23867110	Tumor
chr18	10022				Alu-PCR	Li w et al.2013	23867110	Tumor
chr4	191044236				Alu-PCR	Li w et al.2013	23867110	Tumor
chr5	11737				Alu-PCR	Li w et al.2013	23867110	Tumor
chr5	1295639		TERT	Promoter	Alu-PCR	Li w et al.2013	23867110	Tumor
chr5	1295732		TERT	Promoter	Alu-PCR	Li w et al.2013	23867110	Tumor
chr7	159128623				Alu-PCR	Li w et al.2013	23867110	Tumor
chr9	66782494				Alu-PCR	Li w et al.2013	23867110	Tumor
chr9	66782543				Alu-PCR	Li w et al.2013	23867110	Tumor
chr9	66977751				Alu-PCR	Li w et al.2013	23867110	Tumor
chr9	69704591				Alu-PCR	Li w et al.2013	23867110	Tumor
chrX	61845964				Alu-PCR	Li w et al.2013	23867110	Tumor
chrY	13458418				Alu-PCR	Li w et al.2013	23867110	Tumor
chrY	13861369				Alu-PCR	Li w et al.2013	23867110	Tumor
chrY	13861418				Alu-PCR	Li w et al.2013	23867110	Tumor
chr10	42391510				Alu-PCR	Li w et al.2013	23867110	Tumor
chr10	42396162				Alu-PCR	Li w et al.2013	23867110	Tumor
3p14.1			AL713702	int2	Alu-PCR	MinamiM et al.2005	15806150	Tumor
16q13.3			AXIN1	int2	Alu-PCR	MinamiM et al.2005	15806150	Tumor
3q13.12			BBX	int3	Alu-PCR	MinamiM et al.2005	15806150	Tumor
11q21			BC026191	int1	Alu-PCR	MinamiM et al.2005	15806150	Tumor
19q13.42			CDC42EP5	1.5 kb UP	Alu-PCR	MinamiM et al.2005	15806150	Tumor
5p15.2			CTNND2	int20	Alu-PCR	MinamiM et al.2005	15806150	Tumor
1p35.3			EYA3	int14	Alu-PCR	MinamiM et al.2005	15806150	Tumor
2q31.2			FLJ13946	150 kb UP	Alu-PCR	MinamiM et al.2005	15806150	Tumor
8q21.11			KCNB2	int5	Alu-PCR	MinamiM et al.2005	15806150	Tumor
15q13.3			KLF13	int1	Alu-PCR	MinamiM et al.2005	15806150	Tumor
19q13.42			LAIR2	28 kb UP	Alu-PCR	MinamiM et al.2005	15806150	Tumor
7q22.3			LHFPL3	8.6 kb DN	Alu-PCR	MinamiM et al.2005	15806150	Tumor
2q36.3			LOC283711	47 kb UP	NGS	MinamiM et al.2005	15806150	Tumor
1p36.23			MIG6	4kb DN	NGS	MinamiM et al.2005	15806150	Tumor
7q22.3			MLL5	98 kb UP	NGS	MinamiM et al.2005	15806150	Tumor
4p12			OCIA	6 kb UP	NGS	MinamiM et al.2005	15806150	Tumor
5q34			ODZ2	int7	NGS	MinamiM et al.2005	15806150	Tumor
1p36.23			PARK7	22 kb DN	NGS	MinamiM et al.2005	15806150	Tumor
2q31.2			PDE11A	int17	NGS	MinamiM et al.2005	15806150	Tumor
17q24			PITPNC1	int14 ;	NGS	MinamiM et al.2005	15806150	Tumor
11q21			SEST3	75 kb DN	NGS	MinamiM et al.2005	15806150	Tumor
18q22.2			SOCS4;	61 kb DN	NGS	MinamiM et al.2005	15806150	Tumor
11q21			SRP46	28 kb DN	NGS	MinamiM et al.2005	15806150	Tumor
7q22.3			SRPK2	int17	NGS	MinamiM et al.2005	15806150	Tumor
3p22.3			STAC	114kb UP	NGS	MinamiM et al.2005	15806150	Tumor
8q21.11			TERF1	168 kb UP	NGS	MinamiM et al.2005	15806150	Tumor
			APCL		NGS	MurakamiY et al.2005	16009689	Tumor
			BCL2L2		NGS	MurakamiY et al.2005	16009689	Tumor
			BIRC3		NGS	MurakamiY et al.2005	16009689	Tumor
			CASPR3		NGS	MurakamiY et al.2005	16009689	Tumor
			CCT		NGS	MurakamiY et al.2005	16009689	Tumor
			CHML		NGS	MurakamiY et al.2005	16009689	Tumor
			EVER2		NGS	MurakamiY et al.2005	16009689	Tumor
			FLJ20850		NGS	MurakamiY et al.2005	16009689	Tumor
			FLJ23027		NGS	MurakamiY et al.2005	16009689	Tumor
			FLJ39630		NGS	MurakamiY et al.2005	16009689	Tumor
			FN1		NGS	MurakamiY et al.2005	16009689	Tumor
			FR7		NGS	MurakamiY et al.2005	16009689	Tumor
			GA		NGS	MurakamiY et al.2005	16009689	Tumor
			GCHFR		NGS	MurakamiY et al.2005	16009689	Tumor
			GRID2		NGS	MurakamiY et al.2005	16009689	Tumor
			hMCM8		NGS	MurakamiY et al.2005	16009689	Tumor
			hTERT		NGS	MurakamiY et al.2005	16009689	Tumor
			hTERT		NGS	MurakamiY et al.2005	16009689	Tumor
			hTERT		NGS	MurakamiY et al.2005	16009689	Tumor
			IP3R 1		NGS	MurakamiY et al.2005	16009689	Tumor
			IRAK2		NGS	MurakamiY et al.2005	16009689	Tumor
			IRF2		NGS	MurakamiY et al.2005	16009689	Tumor
			ITPR2		NGS	MurakamiY et al.2005	16009689	Tumor
			KIF3C		NGS	MurakamiY et al.2005	16009689	Tumor
			LOC116166		NGS	MurakamiY et al.2005	16009689	Tumor
			LOC169831		NGS	MurakamiY et al.2005	16009689	Tumor
			LOC204740		NGS	MurakamiY et al.2005	16009689	Tumor
			LOC221026		NGS	MurakamiY et al.2005	16009689	Tumor
			LOC222792		NGS	MurakamiY et al.2005	16009689	Tumor
			LOC253820		NGS	MurakamiY et al.2005	16009689	Tumor
			LOC254277		NGS	MurakamiY et al.2005	16009689	Tumor
			LOC285866		NGS	MurakamiY et al.2005	16009689	Tumor
			LOC342193		NGS	MurakamiY et al.2005	16009689	Tumor
			LOC342670		NGS	MurakamiY et al.2005	16009689	Tumor
			LOC374740		NGS	MurakamiY et al.2005	16009689	Tumor
			LOC376426		NGS	MurakamiY et al.2005	16009689	Tumor
			LOC402235		NGS	MurakamiY et al.2005	16009689	Tumor
			LOC91948		NGS	MurakamiY et al.2005	16009689	Tumor
			MAPK1		NGS	MurakamiY et al.2005	16009689	Tumor
			MGMT		NGS	MurakamiY et al.2005	16009689	Tumor
			MLL2		NGS	MurakamiY et al.2005	16009689	Tumor
			KMT2B		NGS	MurakamiY et al.2005	16009689	Tumor
			MUC16		NGS	MurakamiY et al.2005	16009689	Tumor

Chromosome	Integration site in host genome	Integration site in virus genome	Gene (distance, bp)	Regions	Methods	Author	PMID	Sample
			NCF1		NGS	MurakamiY et al.2005	16009689	Tumor
			NEDD4L		NGS	MurakamiY et al.2005	16009689	Tumor
			NTRK2		NGS	MurakamiY et al.2005	16009689	Tumor
			ORM1		NGS	MurakamiY et al.2005	16009689	Tumor
			p84		NGS	MurakamiY et al.2005	16009689	Tumor
			PDE6D		NGS	MurakamiY et al.2005	16009689	Tumor
			PDGFB		NGS	MurakamiY et al.2005	16009689	Tumor
			PDGFRB		NGS	MurakamiY et al.2005	16009689	Tumor
			RAI17		NGS	MurakamiY et al.2005	16009689	Tumor
			SCARA3		NGS	MurakamiY et al.2005	16009689	Tumor
			SERCA1		NGS	MurakamiY et al.2005	16009689	Tumor
			SFXN5		NGS	MurakamiY et al.2005	16009689	Tumor
			SMOC1		NGS	MurakamiY et al.2005	16009689	Tumor
			ST3GAL IV		NGS	MurakamiY et al.2005	16009689	Tumor
			TACC-1		NGS	MurakamiY et al.2005	16009689	Tumor
			TCEA3		NGS	MurakamiY et al.2005	16009689	Tumor
			TRAP150		NGS	MurakamiY et al.2005	16009689	Tumor
8p23					NGS	Pineau P.et al.1996	8794383	Tumor
8q13					NGS	Pineau P.et al.1998	9519839	Tumor
10q22.1					NGS	Pineau P.et al.1998	9519839	Tumor
			STAT1	Intronic	NGS	SaittaC et al.2015	25677098	Tumor
			STAT1	Intronic	NGS	SaittaC et al.2015	25677098	Tumor
			STAT1	Intronic	NGS	SaittaC et al.2015	25677098	Tumor
			STAT1	Intronic	NGS	SaittaC et al.2015	25677098	Tumor
			TERT	Promoter	NGS	SaittaC et al.2015	25677098	Tumor
			TERT	Promoter	NGS	SaittaC et al.2015	25677098	Tumor
			TERT	Promoter	NGS	SaittaC et al.2015	25677098	Tumor
			TERT	Promoter	NGS	SaittaC et al.2015	25677098	Tumor
			TERT	Promoter	NGS	SaittaC et al.2015	25677098	Tumor
			TERT	Promoter	NGS	SaittaC et al.2015	25677098	Tumor
			TERT	Promoter	NGS	SaittaC et al.2015	25677098	Tumor
			TERT	Promoter	NGS	SaittaC et al.2015	25677098	Tumor
			TERT	Promoter	NGS	SaittaC et al.2015	25677098	Tumor
			TERT	Promoter	NGS	SaittaC et al.2015	25677098	Tumor
			TERT	Promoter	NGS	SaittaC et al.2015	25677098	Tumor
			ALB	Intronic	NGS	SaittaC et al.2015	25677098	Tumor
			ALB	Intronic	NGS	SaittaC et al.2015	25677098	Tumor
			ALB	Intronic	NGS	SaittaC et al.2015	25677098	Tumor
			ALB	Intronic	NGS	SaittaC et al.2015	25677098	Tumor
			ST18	Intronic	NGS	SaittaC et al.2015	25677098	Tumor
			ST18	Intronic	NGS	SaittaC et al.2015	25677098	Tumor
			ST18	Intronic	NGS	SaittaC et al.2015	25677098	Tumor
			ST18	Intronic	NGS	SaittaC et al.2015	25677098	Tumor
			STXBP4	Intronic	NGS	SaittaC et al.2015	25677098	Tumor
			STXBP4	Intronic	NGS	SaittaC et al.2015	25677098	Tumor
			PCMTD2	Intronic	NGS	SaittaC et al.2015	25677098	Tumor
			PCMTD2	Intronic	NGS	SaittaC et al.2015	25677098	Tumor
			PCMTD2	Intronic	NGS	SaittaC et al.2015	25677098	Tumor
			KMT2B	Intronic	NGS	SaittaC et al.2015	25677098	Tumor
			KMT2B	Intronic	NGS	SaittaC et al.2015	25677098	Tumor
			KMT2B	Intronic	NGS	SaittaC et al.2015	25677098	Tumor
			KMT2B	Intronic	NGS	SaittaC et al.2015	25677098	Tumor
			GPR116	Promoter	NGS	SaittaC et al.2015	25677098	Tumor
			GPR116	Promoter	NGS	SaittaC et al.2015	25677098	Tumor
			GPR116	Promoter	NGS	SaittaC et al.2015	25677098	Tumor
			FN1	Exonic	NGS	SaittaC et al.2015	25677098	Tumor
			FN1	Exonic	NGS	SaittaC et al.2015	25677098	Tumor
			FN1	Intronic	NGS	SaittaC et al.2015	25677098	Tumor
			FN1	Intronic	NGS	SaittaC et al.2015	25677098	Tumor
			SYT12	Intronic	NGS	SaittaC et al.2015	25677098	Tumor
			SYT12	Intronic	NGS	SaittaC et al.2015	25677098	Tumor
			MYC/PVT1	Integenic	NGS	SaittaC et al.2015	25677098	Tumor
			MYC/PVT1	Integenic	NGS	SaittaC et al.2015	25677098	Tumor
			MYC/PVT1	Integenic	NGS	SaittaC et al.2015	25677098	Tumor
			MYC/PVT1	Integenic	NGS	SaittaC et al.2015	25677098	Tumor
			MYC/PVT1	Integenic	NGS	SaittaC et al.2015	25677098	Tumor
			MYC/PVT1	Integenic	NGS	SaittaC et al.2015	25677098	Tumor
			MYC/PVT1	Integenic	NGS	SaittaC et al.2015	25677098	Tumor
			GATM	Intronic	NGS	SaittaC et al.2015	25677098	Tumor
			GATM	Intronic	NGS	SaittaC et al.2015	25677098	Tumor
			GATM	Intronic	NGS	SaittaC et al.2015	25677098	Tumor
			ME1	Intronic	NGS	SaittaC et al.2015	25677098	Tumor
1	247279437	3205	C1orf229	promoter	NGS	SungWK et al.2012	22634754	Tumor
1	247279144	1811	C1orf229	promoter	NGS	SungWK et al.2012	22634754	Tumor
1	247279175	301	C1orf229	promoter	NGS	SungWK et al.2012	22634754	Tumor
2	215692960	260		intergenic	NGS	SungWK et al.2012	22634754	Tumor
4	1709025	1960	SLBP	intron	NGS	SungWK et al.2012	22634754	Tumor
4	189028263	219	TRIML2	promoter	NGS	SungWK et al.2012	22634754	Tumor
7	97046779	2036		intergenic	NGS	SungWK et al.2012	22634754	Tumor
19	1264248	2994		intergenic	NGS	SungWK et al.2012	22634754	Tumor
19	1264316	105	CIRBP	promoter	NGS	SungWK et al.2012	22634754	Tumor
19	30277557	306		intergenic	NGS	SungWK et al.2012	22634754	Tumor
19	30277577	3218		intergenic	NGS	SungWK et al.2012	22634754	Tumor
19	30277595	1937		intergenic	NGS	SungWK et al.2012	22634754	Tumor
23	118015438	221		intergenic	NGS	SungWK et al.2012	22634754	Tumor
3	90335043	1816		intergenic	NGS	SungWK et al.2012	22634754	Tumor
5	1285509	356	hTERT	promoter	NGS	SungWK et al.2012	22634754	Tumor
5	1285517	2469	hTERT	promoter	NGS	SungWK et al.2012	22634754	Tumor
5	1295187	1810	hTERT	promoter	NGS	SungWK et al.2012	22634754	Tumor
6	40366163	1772	LRFN2	intron	NGS	SungWK et al.2012	22634754	Tumor
6	40366350	2438	LRFN2	intron	NGS	SungWK et al.2012	22634754	Tumor
1	142579573	2289		intergenic	NGS	SungWK et al.2012	22634754	Tumor
4	100226341	3218	ADH1A	intron	NGS	SungWK et al.2012	22634754	Tumor
4	100226394	1671	ADH1A	intron	NGS	SungWK et al.2012	22634754	Tumor

Chromosome	Integration site in host genome	Integration site in virus genome	Gene (distance, bp)	Regions	Methods	Author	PMID	Sample
5	1295121	1732	hTERT	intron	NGS	SungWK et al.2012	22634754	Tumor
5	1295217	2422	hTERT	promoter	NGS	SungWK et al.2012	22634754	Tumor
2	198804022	1809	PLCL1	intron	NGS	SungWK et al.2012	22634754	Tumor
2	198804063	159	PLCL1	intron	NGS	SungWK et al.2012	22634754	Tumor
5	103427838	1812		intergenic	NGS	SungWK et al.2012	22634754	Tumor
5	103429807	2050		intergenic	NGS	SungWK et al.2012	22634754	Tumor
13	88395590	181		intergenic	NGS	SungWK et al.2012	22634754	Tumor
13	88395609	2257		intergenic	NGS	SungWK et al.2012	22634754	Tumor
13	91419423	1935		intergenic	NGS	SungWK et al.2012	22634754	Tumor
13	91419632	2323		intergenic	NGS	SungWK et al.2012	22634754	Tumor
13	98711596	3083		intergenic	NGS	SungWK et al.2012	22634754	Tumor
13	98711747	29		intergenic	NGS	SungWK et al.2012	22634754	Tumor
16	89938586	384	TCF25	promoter	NGS	SungWK et al.2012	22634754	Tumor
16	89938642	1907	TCF25	promoter	NGS	SungWK et al.2012	22634754	Tumor
20	13088153	2486	SPTLC3	intron	NGS	SungWK et al.2012	22634754	Tumor
20	13088509	1820	SPTLC3	intron	NGS	SungWK et al.2012	22634754	Tumor
20	13094189	2325	SPTLC3	intron	NGS	SungWK et al.2012	22634754	Tumor
20	13094582	1987	SPTLC3	intron	NGS	SungWK et al.2012	22634754	Tumor
6	33827924	1325		intergenic	NGS	SungWK et al.2012	22634754	Tumor
18	107919	675	ROCK1	promoter	NGS	SungWK et al.2012	22634754	Tumor
13	53011792	1707	VPS36	intron	NGS	SungWK et al.2012	22634754	Tumor
8	60291556	924		intergenic	NGS	SungWK et al.2012	22634754	Tumor
8	137398209	2010		intergenic	NGS	SungWK et al.2012	22634754	Tumor
20	47794911	1673	STAU1	intron	NGS	SungWK et al.2012	22634754	Tumor
8	34782288	1922		intergenic	NGS	SungWK et al.2012	22634754	Tumor
16	46416802	2347		intergenic	NGS	SungWK et al.2012	22634754	Tumor
16	46425940	2505		intergenic	NGS	SungWK et al.2012	22634754	Tumor
5	1297651	14	hTERT	promoter	NGS	SungWK et al.2012	22634754	Tumor
5	1297639	3153	hTERT	promoter	NGS	SungWK et al.2012	22634754	Tumor
23	145124063	1815		intergenic	NGS	SungWK et al.2012	22634754	Tumor
3	100565966	1946	ABI3BP	promoter	NGS	SungWK et al.2012	22634754	Tumor
3	100565932	59	ABI3BP	promoter	NGS	SungWK et al.2012	22634754	Tumor
3	100565895	3148	ABI3BP	promoter	NGS	SungWK et al.2012	22634754	Tumor
3	144417689	56		intergenic	NGS	SungWK et al.2012	22634754	Tumor
5	1295723	1807	hTERT	promoter	NGS	SungWK et al.2012	22634754	Tumor
5	1319152	1852	CLPTM1L	intron	NGS	SungWK et al.2012	22634754	Tumor
10	42400656	780		intergenic	NGS	SungWK et al.2012	22634754	Tumor
5	59252107	2618	PDE4D	intron	NGS	SungWK et al.2012	22634754	Tumor
5	59252148	1826	PDE4D	intron	NGS	SungWK et al.2012	22634754	Tumor
12	84442929	1831		intergenic	NGS	SungWK et al.2012	22634754	Tumor
12	120812093	220	MSI1	promoter	NGS	SungWK et al.2012	22634754	Tumor
3	7194528	1363	GRM7	intron	NGS	SungWK et al.2012	22634754	Tumor
3	196625719	1901	SENP5	intron	NGS	SungWK et al.2012	22634754	Tumor
5	17412236	31		intergenic	NGS	SungWK et al.2012	22634754	Tumor
7	109739518	1761		intergenic	NGS	SungWK et al.2012	22634754	Tumor
10	42387856	1888		intergenic	NGS	SungWK et al.2012	22634754	Tumor
10	42394337	1905		intergenic	NGS	SungWK et al.2012	22634754	Tumor
12	34372612	2120		intergenic	NGS	SungWK et al.2012	22634754	Tumor
12	56868896	203	GLS2	intron	NGS	SungWK et al.2012	22634754	Tumor
12	56869742	3066	GLS2	intron	NGS	SungWK et al.2012	22634754	Tumor
12	84251819	644		intergenic	NGS	SungWK et al.2012	22634754	Tumor
13	49243132	723	CYSLTR2	intron	NGS	SungWK et al.2012	22634754	Tumor
14	42967875	1459		intergenic	NGS	SungWK et al.2012	22634754	Tumor
1	64616818	2738	AK096291	intron	NGS	SungWK et al.2012	22634754	Tumor
1	64616823	9	AK096291	intron	NGS	SungWK et al.2012	22634754	Tumor
1	64616819	2568	AK096291	intron	NGS	SungWK et al.2012	22634754	Tumor
5	1295365	740	hTERT	promoter	NGS	SungWK et al.2012	22634754	Tumor
5	1295367	752	hTERT	promoter	NGS	SungWK et al.2012	22634754	Tumor
8	56264215	2138	XKR4	intron	NGS	SungWK et al.2012	22634754	Tumor
9	79323010	1620	BMCC1	promoter	NGS	SungWK et al.2012	22634754	Tumor
10	42387937	539		intergenic	NGS	SungWK et al.2012	22634754	Tumor
19	36212740	1813	KMT2B	intron	NGS	SungWK et al.2012	22634754	Tumor
6	71303876	1825		intergenic	NGS	SungWK et al.2012	22634754	Tumor
6	71303937	166		intergenic	NGS	SungWK et al.2012	22634754	Tumor
19	36212892	1387	KMT2B	intron	NGS	SungWK et al.2012	22634754	Tumor
7	134071791	21		intergenic	NGS	SungWK et al.2012	22634754	Tumor
8	53342019	1304		intergenic	NGS	SungWK et al.2012	22634754	Tumor
14	42989009	2028		intergenic	NGS	SungWK et al.2012	22634754	Tumor
14	42989246	484		intergenic	NGS	SungWK et al.2012	22634754	Tumor
1	164826273	1725	PBX1	intron	NGS	SungWK et al.2012	22634754	Tumor
4	168808596	1057		intergenic	NGS	SungWK et al.2012	22634754	Tumor
5	1295400	2968	hTERT	promoter	NGS	SungWK et al.2012	22634754	Tumor
18	38204577	765		intergenic	NGS	SungWK et al.2012	22634754	Tumor
18	38204577	2401		intergenic	NGS	SungWK et al.2012	22634754	Tumor
21	11132385	736		intergenic	NGS	SungWK et al.2012	22634754	Tumor
5	1295605	1810	hTERT	promoter	NGS	SungWK et al.2012	22634754	Tumor
1	90826072	1847		intergenic	NGS	SungWK et al.2012	22634754	Tumor
9	119720873	1635	ASTN2	intron	NGS	SungWK et al.2012	22634754	Tumor
19	36212821	1526	KMT2B	intron	NGS	SungWK et al.2012	22634754	Tumor
19	36212826	2082	KMT2B	intron	NGS	SungWK et al.2012	22634754	Tumor
4	75116243	2872	MTHFD2L	intron	NGS	SungWK et al.2012	22634754	Tumor
4	75116693	1629	MTHFD2L	intron	NGS	SungWK et al.2012	22634754	Tumor
5	1296259	1894	hTERT	promoter	NGS	SungWK et al.2012	22634754	Tumor
5	1299589	1762	hTERT	promoter	NGS	SungWK et al.2012	22634754	Tumor
5	1299592	1820	hTERT	promoter	NGS	SungWK et al.2012	22634754	Tumor
5	1298957	1827	hTERT	promoter	NGS	SungWK et al.2012	22634754	Tumor
11	4676920	2304		intergenic	NGS	SungWK et al.2012	22634754	Tumor
11	4678147	1783		intergenic	NGS	SungWK et al.2012	22634754	Tumor
13	84766274	1874		intergenic	NGS	SungWK et al.2012	22634754	Tumor
13	84766393	1303		intergenic	NGS	SungWK et al.2012	22634754	Tumor
2	110639781	1849		intergenic	NGS	SungWK et al.2012	22634754	Tumor
4	143612455	1704	INPP4B	intron	NGS	SungWK et al.2012	22634754	Tumor
8	59068667	1878		intergenic	NGS	SungWK et al.2012	22634754	Tumor
16	49205999	1405		intergenic	NGS	SungWK et al.2012	22634754	Tumor

Chromosome	Integration site in host genome	Integration site in virus genome	Gene (distance, bp)	Regions	Methods	Author	PMID	Sample
19	36212312	227	KMT2B	intron	NGS	SungWK et al.2012	22634754	Tumor
19	36212331	1932	KMT2B	intron	NGS	SungWK et al.2012	22634754	Tumor
20	16043303	2461		intergenic	NGS	SungWK et al.2012	22634754	Tumor
1	174619720	1178	RABGAP1L	intron	NGS	SungWK et al.2012	22634754	Tumor
3	196625744	417	SENP5	intron	NGS	SungWK et al.2012	22634754	Tumor
3	197900733	2499	FAM157A	intron	NGS	SungWK et al.2012	22634754	Tumor
5	159241589	1469		intergenic	NGS	SungWK et al.2012	22634754	Tumor
5	159412324	1891		intergenic	NGS	SungWK et al.2012	22634754	Tumor
8	69519927	2838	C8orf34	intron	NGS	SungWK et al.2012	22634754	Tumor
8	69523219	1942	C8orf34	intron	NGS	SungWK et al.2012	22634754	Tumor
8	69523491	253	C8orf34	intron	NGS	SungWK et al.2012	22634754	Tumor
8	69528052	1429	C8orf34	intron	NGS	SungWK et al.2012	22634754	Tumor
10	42384373	443		intergenic	NGS	SungWK et al.2012	22634754	Tumor
14	36016828	1820	RALGAPA1	intron	NGS	SungWK et al.2012	22634754	Tumor
18	72836752	33		intergenic	NGS	SungWK et al.2012	22634754	Tumor
18	72836901	2035		intergenic	NGS	SungWK et al.2012	22634754	Tumor
19	36213015	774	KMT2B	intron	NGS	SungWK et al.2012	22634754	Tumor
19	36213273	1599	KMT2B	intron	NGS	SungWK et al.2012	22634754	Tumor
1	14860398	2386		intergenic	NGS	SungWK et al.2012	22634754	Tumor
2	207883313	1752		intergenic	NGS	SungWK et al.2012	22634754	Tumor
5	1295167	1830	hTERT	promoter	NGS	SungWK et al.2012	22634754	Tumor
6	72439844	1860		intergenic	NGS	SungWK et al.2012	22634754	Tumor
6	74850223	2680	AF086303	intron	NGS	SungWK et al.2012	22634754	Tumor
8	112834660	1826		intergenic	NGS	SungWK et al.2012	22634754	Tumor
10	879223	241	LARP4B	intron	NGS	SungWK et al.2012	22634754	Tumor
1	14220001	1780		intergenic	NGS	SungWK et al.2012	22634754	Tumor
5	95012131	1821	SPATA9	intron	NGS	SungWK et al.2012	22634754	Tumor
7	12427042	1577	VWDE	intron	NGS	SungWK et al.2012	22634754	Tumor
8	5841656	1718		intergenic	NGS	SungWK et al.2012	22634754	Tumor
8	67632214	1851	SGK3	intron	NGS	SungWK et al.2012	22634754	Tumor
8	90411435	1820		intergenic	NGS	SungWK et al.2012	22634754	Tumor
8	90412055	1468		intergenic	NGS	SungWK et al.2012	22634754	Tumor
8	104412731	1834	SLC25A32	intron	NGS	SungWK et al.2012	22634754	Tumor
8	109079440	2222	RSPO2	intron	NGS	SungWK et al.2012	22634754	Tumor
8	109956262	1828		intergenic	NGS	SungWK et al.2012	22634754	Tumor
8	115991677	1857		intergenic	NGS	SungWK et al.2012	22634754	Tumor
9	30651084	2109		intergenic	NGS	SungWK et al.2012	22634754	Tumor
10	42391858	1012		intergenic	NGS	SungWK et al.2012	22634754	Tumor
11	4588164	1828		intergenic	NGS	SungWK et al.2012	22634754	Tumor
16	27195945	256		intergenic	NGS	SungWK et al.2012	22634754	Tumor
19	23422780	1714	AK301230	intron	NGS	SungWK et al.2012	22634754	Tumor
22	37997820	644		intergenic	NGS	SungWK et al.2012	22634754	Tumor
5	1295518	1671	hTERT	promoter	NGS	SungWK et al.2012	22634754	Tumor
15	27031371	543	GABRB3	intron	NGS	SungWK et al.2012	22634754	Tumor
15	27031546	144	GABRB3	intron	NGS	SungWK et al.2012	22634754	Tumor
1	118200636	1710		intergenic	NGS	SungWK et al.2012	22634754	Tumor
1	118200706	275		intergenic	NGS	SungWK et al.2012	22634754	Tumor
6	43832278	2482		intergenic	NGS	SungWK et al.2012	22634754	Tumor
9	14938350	169		intergenic	NGS	SungWK et al.2012	22634754	Tumor
5	173929016	1827		intergenic	NGS	SungWK et al.2012	22634754	Tumor
6	30481176	1798		intergenic	NGS	SungWK et al.2012	22634754	Tumor
6	30481221	1624		intergenic	NGS	SungWK et al.2012	22634754	Tumor
5	1295607	94	hTERT	promoter	NGS	SungWK et al.2012	22634754	Tumor
18	61954447	1772	BC036306	intron	NGS	SungWK et al.2012	22634754	Tumor
18	61954985	2004	BC036306	intron	NGS	SungWK et al.2012	22634754	Tumor
1	47404506	2797	CYP4A11	intron	NGS	SungWK et al.2012	22634754	Tumor
8	101905731	371		intergenic	NGS	SungWK et al.2012	22634754	Tumor
10	42385538	2765		intergenic	NGS	SungWK et al.2012	22634754	Tumor
19	36212565	2240	KMT2B	intron	NGS	SungWK et al.2012	22634754	Tumor
19	36212602	1781	KMT2B	intron	NGS	SungWK et al.2012	22634754	Tumor
23	115777227	1387		intergenic	NGS	SungWK et al.2012	22634754	Tumor
4	32068	690		intergenic	NGS	SungWK et al.2012	22634754	Tumor
19	53142158	1621	ZNF83	promoter	NGS	SungWK et al.2012	22634754	Tumor
1	3190800	1930	PRDM16	intron	NGS	SungWK et al.2012	22634754	Tumor
1	151503456	2829	CGN	promoter	NGS	SungWK et al.2012	22634754	Tumor
1	151509234	2332	CGN	promoter	NGS	SungWK et al.2012	22634754	Tumor
18	45256947	1826		intergenic	NGS	SungWK et al.2012	22634754	Tumor
23	14603890	2053	GLRA3	intron	NGS	SungWK et al.2012	22634754	Tumor
23	14603891	2062	GLRA3	intron	NGS	SungWK et al.2012	22634754	Tumor
5	37812440	1891		intergenic	NGS	SungWK et al.2012	22634754	Tumor
5	37812520	1683		intergenic	NGS	SungWK et al.2012	22634754	Tumor
5	64014600	2616	SFRS12IP1	intron	NGS	SungWK et al.2012	22634754	Tumor
5	64014657	1811	SFRS12IP1	intron	NGS	SungWK et al.2012	22634754	Tumor
19	30304177	1779	CCNE1	intron	NGS	SungWK et al.2012	22634754	Tumor
13	63147352	1828		intergenic	NGS	SungWK et al.2012	22634754	Tumor
13	63147362	1818		intergenic	NGS	SungWK et al.2012	22634754	Tumor
15	88688212	1814	NTRK3	intron	NGS	SungWK et al.2012	22634754	Tumor
15	88688187	1826	NTRK3	intron	NGS	SungWK et al.2012	22634754	Tumor
12	119475798	2116	SRRM4	intron	NGS	SungWK et al.2012	22634754	Tumor
19	54910801	1561		intergenic	NGS	SungWK et al.2012	22634754	Tumor
19	54911045	1543		intergenic	NGS	SungWK et al.2012	22634754	Tumor
15	31629954	1810	KLF13	intron	NGS	SungWK et al.2012	22634754	Tumor
15	31629964	2714	KLF13	intron	NGS	SungWK et al.2012	22634754	Tumor
8	39087853	2148	ADAM32	intron	NGS	SungWK et al.2012	22634754	Tumor
19	36212723	1608	KMT2B	intron	NGS	SungWK et al.2012	22634754	Tumor
19	36212737	1560	KMT2B	intron	NGS	SungWK et al.2012	22634754	Tumor
2	220807737	59		intergenic	NGS	SungWK et al.2012	22634754	Tumor
2	220807986	2721		intergenic	NGS	SungWK et al.2012	22634754	Tumor
4	157678671	455		intergenic	NGS	SungWK et al.2012	22634754	Tumor
8	10875912	1929	XKR6	intron	NGS	SungWK et al.2012	22634754	Tumor
8	12270963	2833	FAM66D	intron	NGS	SungWK et al.2012	22634754	Tumor
13	22756743	592	AK054845	intron	NGS	SungWK et al.2012	22634754	Tumor
13	22948394	752		intergenic	NGS	SungWK et al.2012	22634754	Tumor
13	24964482	1762		intergenic	NGS	SungWK et al.2012	22634754	Tumor

Supplementary Table S8 Continued

Chromosome	Integration site in host genome	Integration site in virus genome	Gene (distance, bp)	Regions	Methods	Author	PMID	Sample
13	24964478	492		intergenic	NGS	SungWK et al.2012	22634754	Tumor
13	56044273	219		intergenic	NGS	SungWK et al.2012	22634754	Tumor
9	66971323	1657		intergenic	NGS	SungWK et al.2012	22634754	Tumor
11	69687299	1798		intergenic	NGS	SungWK et al.2012	22634754	Tumor
23	144158529	782		intergenic	NGS	SungWK et al.2012	22634754	Tumor
4	91202521	1826	FAM190A	intron	Cassette ligation–mediated	SungWK et al.2012	22634754	Tumor
5	1295112	2947	hTERT	intron	Cassette ligation–mediated	SungWK et al.2012	22634754	Tumor
8	91482800	2730		intergenic	Cassette ligation–mediated	SungWK et al.2012	22634754	Tumor
8	91482828	113		intergenic	Cassette ligation–mediated	SungWK et al.2012	22634754	Tumor
10	42385400	1880		intergenic	Cassette ligation–mediated	SungWK et al.2012	22634754	Tumor
10	42391029	1832		intergenic	Cassette ligation–mediated	SungWK et al.2012	22634754	Tumor
10	42394147	1867		intergenic	Cassette ligation–mediated	SungWK et al.2012	22634754	Tumor
15	30215085	1838		intergenic	Cassette ligation–mediated	SungWK et al.2012	22634754	Tumor
15	30215145	2443		intergenic	GS-FLX Titanium Sequenci	SungWK et al.2012	22634754	Tumor
17	21201604	1619	MAP2K3	intron	GS-FLX Titanium Sequenci	SungWK et al.2012	22634754	Tumor
11	62426036	2983		intergenic	GS-FLX Titanium Sequenci	SungWK et al.2012	22634754	Tumor
12	57280755	734		intergenic	GS-FLX Titanium Sequenci	SungWK et al.2012	22634754	Tumor
12	57308159	1239		intergenic	GS-FLX Titanium Sequenci	SungWK et al.2012	22634754	Tumor
13	75213064	1937		intergenic	GS-FLX Titanium Sequenci	SungWK et al.2012	22634754	Tumor
13	75214806	1722		intergenic	GS-FLX Titanium Sequenci	SungWK et al.2012	22634754	Tumor
14	73560650	1955	RBM25	intron	GS-FLX Titanium Sequenci	SungWK et al.2012	22634754	Tumor
18	63841663	2637		intergenic	GS-FLX Titanium Sequenci	SungWK et al.2012	22634754	Tumor
19	19341571	66	NCAN	intron	GS-FLX Titanium Sequenci	SungWK et al.2012	22634754	Tumor
19	19341767	2986	NCAN	intron	GS-FLX Titanium Sequenci	SungWK et al.2012	22634754	Tumor
19	19379764	2654	HAPLN4	intron	GS-FLX Titanium Sequenci	SungWK et al.2012	22634754	Tumor
19	30303494	1059	CCNE1	promoter	GS-FLX Titanium Sequenci	SungWK et al.2012	22634754	Tumor
19	30303498	1820	CCNE1	promoter	GS-FLX Titanium Sequenci	SungWK et al.2012	22634754	Tumor
23	113870720	2147	HTR2C	intron	GS-FLX Titanium Sequenci	SungWK et al.2012	22634754	Tumor
23	113875804	1766	HTR2C	intron	GS-FLX Titanium Sequenci	SungWK et al.2012	22634754	Tumor
4	57610960	1902		intergenic	GS-FLX Titanium Sequenci	SungWK et al.2012	22634754	Tumor
19	36212764	1526	KMT2B	intron	GS-FLX Titanium Sequenci	SungWK et al.2012	22634754	Tumor
19	36212793	1827	KMT2B	intron	GS-FLX Titanium Sequenci	SungWK et al.2012	22634754	Tumor
6	157208976	2176	ARID1B	intron	GS-FLX Titanium Sequenci	SungWK et al.2012	22634754	Tumor
6	157209000	1798	ARID1B	intron	GS-FLX Titanium Sequenci	SungWK et al.2012	22634754	Tumor
1	180361658	924	ACBD6	intron	GS-FLX Titanium Sequenci	SungWK et al.2012	22634754	Tumor
1	180361678	196	ACBD6	intron	GS-FLX Titanium Sequenci	SungWK et al.2012	22634754	Tumor
2	226810125	1627		intergenic	GS-FLX Titanium Sequenci	SungWK et al.2012	22634754	Tumor
5	1302542	1820		intergenic	GS-FLX Titanium Sequenci	SungWK et al.2012	22634754	Tumor
5	1302548	1824		intergenic	GS-FLX Titanium Sequenci	SungWK et al.2012	22634754	Tumor
5	8407974	2441	BC043282	intron	GS-FLX Titanium Sequenci	SungWK et al.2012	22634754	Tumor
6	4952218	704	CDYL	intron	GS-FLX Titanium Sequenci	SungWK et al.2012	22634754	Tumor
6	4951683	1827	CDYL	intron	GS-FLX Titanium Sequenci	SungWK et al.2012	22634754	Tumor
6	6068443	580		intergenic	GS-FLX Titanium Sequenci	SungWK et al.2012	22634754	Tumor
9	32179450	1827		intergenic	GS-FLX Titanium Sequenci	SungWK et al.2012	22634754	Tumor
11	41768443	2335		intergenic	GS-FLX Titanium Sequenci	SungWK et al.2012	22634754	Tumor
11	41906692	225		intergenic	GS-FLX Titanium Sequenci	SungWK et al.2012	22634754	Tumor
11	41906694	2308		intergenic	GS-FLX Titanium Sequenci	SungWK et al.2012	22634754	Tumor
11	41907626	1786		intergenic	GS-FLX Titanium Sequenci	SungWK et al.2012	22634754	Tumor
5	158787996	1852	AK097548	intron	GS-FLX Titanium Sequenci	SungWK et al.2012	22634754	Tumor
3	196625755	1826	SENP5	intron	GS-FLX Titanium Sequenci	SungWK et al.2012	22634754	Tumor
10	42387281	1914		intergenic	GS-FLX Titanium Sequenci	SungWK et al.2012	22634754	Tumor
17	10013676	2816	GAS7	intron	GS-FLX Titanium Sequenci	SungWK et al.2012	22634754	Tumor
17	10013673	1	GAS7	intron	GS-FLX Titanium Sequenci	SungWK et al.2012	22634754	Tumor
17	10269457	1800	MYH13	intron	GS-FLX Titanium Sequenci	SungWK et al.2012	22634754	Tumor
17	21743132	1467		intergenic	GS-FLX Titanium Sequenci	SungWK et al.2012	22634754	Tumor
17	21987709	1059		intergenic	GS-FLX Titanium Sequenci	SungWK et al.2012	22634754	Tumor
17	60408789	540		intergenic	GS-FLX Titanium Sequenci	SungWK et al.2012	22634754	Tumor
17	60408830	1857		intergenic	GS-FLX Titanium Sequenci	SungWK et al.2012	22634754	Tumor
2	15366499	1826	NBAS	intron	GS-FLX Titanium Sequenci	SungWK et al.2012	22634754	Tumor
2	15512470	1912	NBAS	intron	GS-FLX Titanium Sequenci	SungWK et al.2012	22634754	Tumor
13	69086031	754		intergenic	GS-FLX Titanium Sequenci	SungWK et al.2012	22634754	Tumor
19	36214006	2449	KMT2B	intron	GS-FLX Titanium Sequenci	SungWK et al.2012	22634754	Tumor
19	36214011	1616	KMT2B	intron	GS-FLX Titanium Sequenci	SungWK et al.2012	22634754	Tumor
1	121360123	1035		intergenic	GS-FLX Titanium Sequenci	SungWK et al.2012	22634754	Tumor
23	31137264	2928		intergenic	GS-FLX Titanium Sequenci	SungWK et al.2012	22634754	Tumor
23	31469842	2019	DMD	intron	GS-FLX Titanium Sequenci	SungWK et al.2012	22634754	Tumor
3	191648205	1974		intergenic	GS-FLX Titanium Sequenci	SungWK et al.2012	22634754	Tumor
5	1269360	812	hTERT	intron	GS-FLX Titanium Sequenci	SungWK et al.2012	22634754	Tumor
5	1269405	1950	hTERT	intron	GS-FLX Titanium Sequenci	SungWK et al.2012	22634754	Tumor
8	82390662	1798	FABP4	intron	GS-FLX Titanium Sequenci	SungWK et al.2012	22634754	Tumor
10	125472276	1159	DKFZp666J235	intron	Southern blot hybridization	SungWK et al.2012	22634754	Tumor
10	131711537	1566	EBF3	intron	Southern blot hybridization	SungWK et al.2012	22634754	Tumor
10	131726471	1147	EBF3	intron	Southern blot hybridization	SungWK et al.2012	22634754	Tumor
12	113741935	1922	SLC24A6	intron	Southern blot hybridization	SungWK et al.2012	22634754	Tumor
14	96085136	1721		intergenic	Southern blot hybridization	SungWK et al.2012	22634754	Tumor
14	96085338	1993		intergenic	Southern blot hybridization	SungWK et al.2012	22634754	Tumor
17	18287134	1833	EVPLL	intron	Southern blot hybridization	SungWK et al.2012	22634754	Tumor
18	3108768	1883	MYOM1	intron	Southern blot hybridization	SungWK et al.2012	22634754	Tumor
19	30315004	1798	CCNE1	intron	Southern blot hybridization	SungWK et al.2012	22634754	Tumor
19	30315371	230		intergenic	Southern blot hybridization	SungWK et al.2012	22634754	Tumor
19	30315376	3171		intergenic	Southern blot hybridization	SungWK et al.2012	22634754	Tumor
20	11962578	1892		intergenic	Southern blot hybridization	SungWK et al.2012	22634754	Tumor
2	182140560	2175	AK125001	intron	Southern blot hybridization	SungWK et al.2012	22634754	Tumor
18	109965	1757	ROCK1	intron	Southern blot hybridization	SungWK et al.2012	22634754	Tumor
21	9861617	1885		intergenic	Southern blot hybridization	SungWK et al.2012	22634754	Tumor
24	13468207	1785		intergenic	Southern blot hybridization	SungWK et al.2012	22634754	Tumor
24	58993181	1711		intergenic	Southern blot hybridization	SungWK et al.2012	22634754	Tumor
1	41256045	1803	KCNQ4	intron	HIVID	SungWK et al.2012	22634754	Tumor
4	52727435	1839	DCUN1D4	intron	HIVID	SungWK et al.2012	22634754	Tumor
8	58257849	1006	BC048118	intron	HIVID	SungWK et al.2012	22634754	Tumor
17	18834580	2237	PRPSAP2	intron	HIVID	SungWK et al.2012	22634754	Tumor
8	49853483	1517		intergenic	HIVID	SungWK et al.2012	22634754	Tumor
1	17842327	836		intergenic	HIVID	SungWK et al.2012	22634754	Tumor
1	17842436	1665		intergenic	HIVID	SungWK et al.2012	22634754	Tumor

Chromosome	Integration site in host genome	Integration site in virus genome	Gene (distance, bp)	Regions	Methods	Author	PMID	Sample
10	62130894	2860	ANK3	intron	HIVID	SungWK et al.2012	22634754	Tumor
10	62130934	2204	ANK3	intron	HIVID	SungWK et al.2012	22634754	Tumor
1	27898444	1826	AHDC1	intron	HIVID	SungWK et al.2012	22634754	Tumor
1	153943072	1720	SLC39A1	promoter	HIVID	SungWK et al.2012	22634754	Tumor
5	1295683	1835	hTERT	promoter	HIVID	SungWK et al.2012	22634754	Tumor
5	1295891	647	hTERT	promoter	HIVID	SungWK et al.2012	22634754	Tumor
16	56539205	2690	BBS2	intron	HIVID	SungWK et al.2012	22634754	Tumor
16	56539306	2196	BBS2	intron	HIVID	SungWK et al.2012	22634754	Tumor
2	52295318	2658		intergenic	HIVID	SungWK et al.2012	22634754	Tumor
4	765946	2449		intergenic	HIVID	SungWK et al.2012	22634754	Tumor
8	32314573	2373	NRG1	intron	HIVID	SungWK et al.2012	22634754	Tumor
13	86969110	1815		intergenic	HIVID	SungWK et al.2012	22634754	Tumor
13	86969208	2079		intergenic	HIVID	SungWK et al.2012	22634754	Tumor
1	80107938	1853		intergenic	HIVID	SungWK et al.2012	22634754	Tumor
3	192715997	1851		intergenic	HIVID	SungWK et al.2012	22634754	Tumor
3	192716260	2418		intergenic	HIVID	SungWK et al.2012	22634754	Tumor
5	1295382	2534	hTERT	promoter	HIVID	SungWK et al.2012	22634754	Tumor
5	1295496	2257	hTERT	promoter	HIVID	SungWK et al.2012	22634754	Tumor
10	107780218	2051		intergenic	HIVID	SungWK et al.2012	22634754	Tumor
1	245226299	2042	EFCAB2	intron	HIVID	SungWK et al.2012	22634754	Tumor
1	245226113	1761	EFCAB2	intron	HIVID	SungWK et al.2012	22634754	Tumor
7	109957462	165		intergenic	HIVID	SungWK et al.2012	22634754	Tumor
10	107298472	1795		intergenic	HIVID	SungWK et al.2012	22634754	Tumor
10	107298448	345		intergenic	HIVID	SungWK et al.2012	22634754	Tumor
13	30606159	2476		intergenic	HIVID	SungWK et al.2012	22634754	Tumor
24	58846331	2412		intergenic	HIVID	SungWK et al.2012	22634754	Tumor
24	58857118	2421		intergenic	HIVID	SungWK et al.2012	22634754	Tumor
5	1292392	2447	hTERT	intron	HIVID	SungWK et al.2012	22634754	Tumor
5	1292403	355	hTERT	intron	HIVID	SungWK et al.2012	22634754	Tumor
19	30297359	2839		intergenic	HIVID	SungWK et al.2012	22634754	Tumor
19	30298787	1931	CCNE1	promoter	HIVID	SungWK et al.2012	22634754	Tumor
4	146707000	985	ZNF827	intron	HIVID	SungWK et al.2012	22634754	Tumor
8	99118662	1737	HRSP12	intron	HIVID	SungWK et al.2012	22634754	Tumor
8	99118919	3184	HRSP12	intron	HIVID	SungWK et al.2012	22634754	Tumor
2	171571967	469	SP5	promoter	HIVID	SungWK et al.2012	22634754	Tumor
17	81195074	411		intergenic	HIVID	SungWK et al.2012	22634754	Tumor
8	42501862	1664		intergenic	HIVID	SungWK et al.2012	22634754	Tumor
8	42501977	2454		intergenic	HIVID	SungWK et al.2012	22634754	Tumor
17	17587733	1606	RAI1	intron	HIVID	SungWK et al.2012	22634754	Tumor
17	17634877	775	RAI1	intron	HIVID	SungWK et al.2012	22634754	Tumor
			LOC168991		HIVID	TamoriA et al. 2005	16115921	Tumor
			LOC255345		HIVID	TamoriA et al. 2005	16115921	Tumor
			calmodulin 1		HIVID	TamoriA et al. 2005	16115921	Tumor
			FLJ333655		HIVID	TamoriA et al. 2005	16115921	Tumor
			LOC220220		HIVID	TamoriA et al. 2005	16115921	Tumor
			LOC220272		HIVID	TamoriA et al. 2005	16115921	Tumor
			mixed lineage leukemia 2 (MLL2)		HIVID	TamoriA et al. 2005	16115921	Tumor
			ras-responsive element binding protein 1		HIVID	TamoriA et al. 2005	16115921	Tumor
10	NA		ADARB2	Intron 1	HIVID	Tohs et al. 2013		Tumor
10	NA		ADARB2	Intron 1	HIVID	Tohs et al. 2013		Tumor
11	NA		AIP	Intron 5	HIVID	Tohs et al. 2013		Tumor
18	NA		ALPK2	Intron 1	HIVID	Tohs et al. 2013		Tumor
10	NA		ATRNL1	Intron 26	HIVID	Tohs et al. 2013		Tumor
10	NA		BTAF1	Intron 34	HIVID	Tohs et al. 2013		Tumor
10	NA		C10orf129	11	HIVID	Tohs et al. 2013		Tumor
4	NA		CCNA2	Intron 2	HIVID	Tohs et al. 2013		Tumor
4	NA		CCNA2	Intron 2	HIVID	Tohs et al. 2013		Tumor
17	NA		CDK12	Intron 10	HIVID	Tohs et al. 2013		Tumor
4	NA		CYCSP14	807	HIVID	Tohs et al. 2013		Tumor
10	NA		CYP2C8	50	HIVID	Tohs et al. 2013		Tumor
20	NA		DEFB132	1	HIVID	Tohs et al. 2013		Tumor
3	NA		FANCD2	Intron 26	HIVID	Tohs et al. 2013		Tumor
10	NA		FAS	Intron 7	HIVID	Tohs et al. 2013		Tumor
18	NA		FGF7P1	22	HIVID	Tohs et al. 2013		Tumor
18	NA		GATA6	19	HIVID	Tohs et al. 2013		Tumor
18	NA		GATA6	19	HIVID	Tohs et al. 2013		Tumor
8	NA		GDF6	28	HIVID	Tohs et al. 2013		Tumor
8	NA		HEV1	9	HIVID	Tohs et al. 2013		Tumor
8	NA		HGSNAT	26	HIVID	Tohs et al. 2013		Tumor
5	NA		KIAA0947	30	HIVID	Tohs et al. 2013		Tumor
17	NA		KRT32	Exon 6	HIVID	Tohs et al. 2013		Tumor
10	NA		LIPF	12	HIVID	Tohs et al. 2013		Tumor
17	NA		LOC100129683	44	HIVID	Tohs et al. 2013		Tumor
4	NA		LOC100289626	193	HIVID	Tohs et al. 2013		Tumor
Y	NA		LOC100507426	0.075	HIVID	Tohs et al. 2013		Tumor
4	NA		LOC389223	30	HIVID	Tohs et al. 2013		Tumor
17	NA		MAP2K4	65	HIVID	Tohs et al. 2013		Tumor
17	NA		MSI2	18	HIVID	Tohs et al. 2013		Tumor
X	NA		MUM1L1	5	HIVID	Tohs et al. 2013		Tumor
15	NA		MYO9A	1	HIVID	Tohs et al. 2013		Tumor
10	NA		NCOA4/MSMB#	3/Intron 3#	HIVID	Tohs et al. 2013		Tumor
17	NA		NN1	Intron 6	HIVID	Tohs et al. 2013		Tumor
14	NA		POLE2	Intron 9	HIVID	Tohs et al. 2013		Tumor
1	NA		PRDM2	271	HIVID	Tohs et al. 2013		Tumor
8	NA		PRKDC	Intron 76	HIVID	Tohs et al. 2013		Tumor
2	NA		RBMS1	188	HIVID	Tohs et al. 2013		Tumor
2	NA		RDH14	258	HIVID	Tohs et al. 2013		Tumor
10	NA		REEP3	85	HIVID	Tohs et al. 2013		Tumor
4	NA		RPL17P20	21	HIVID	Tohs et al. 2013		Tumor
11	NA		RPS3	7	HIVID	Tohs et al. 2013		Tumor
7	NA		SLC26A5	Intron 18	HIVID	Tohs et al. 2013		Tumor
5	NA		SLC6A3	11	HIVID	Tohs et al. 2013		Tumor
5	NA		TERT	3	HIVID	Tohs et al. 2013		Tumor
5	NA		TERT	0.761	HIVID	Tohs et al. 2013		Tumor

Chromosome	Integration site in host genome	Integration site in virus genome	Gene (distance, bp)	Regions	Methods	Author	PMID	Sample
5	NA		TERT	0.387	HIVID	Tohs et al. 2013		Tumor
5	NA		TERT	0.027	HIVID	Tohs et al. 2013		Tumor
5	NA		TERT	0.847	HIVID	Tohs et al. 2013		Tumor
5	NA		TERT	0.55	HIVID	Tohs et al. 2013		Tumor
8	NA		TG	Exon 37	HIVID	Tohs et al. 2013		Tumor
4	NA		TRAM1L1	66	HIVID	Tohs et al. 2013		Tumor
6	NA		TRDN	18	HIVID	Tohs et al. 2013		Tumor
9	NA		TUSC1	591	HIVID	Tohs et al. 2013		Tumor
9	NA		UBAP2	Intron 12	HIVID	Tohs et al. 2013		Tumor
5	NA		ZDHHC11	Intron 11	HIVID	Tohs et al. 2013		Tumor
19	NA		ZNF607	9	HIVID	Tohs et al. 2013		Tumor
20p13			Chromosome 20 open reading frame 98	INT (same)	HIVID	WangY et al.2004	14712219	Tumor
8p12			GTF2E2	INT (same)	HIVID	WangY et al.2004	14712219	Tumor
1q42			Hypothetical protein MGC27277	INT (same)	HIVID	WangY et al.2004	14712219	Tumor
11q13			KIAA0769	INT (same)	HIVID	WangY et al.2004	14712219	Tumor
13q34			KIAA1802	INT (opposite)	HIVID	WangY et al.2004	14712219	Tumor
10q21.3			LOC256328	INT (opposite)	HIVID	WangY et al.2004	14712219	Tumor
19q13.1			Nil	Nil	HIVID	WangY et al.2004	14712219	Tumor
3p14–p21			Nil	Nil	HIVID	WangY et al.2004	14712219	Tumor
3p23			Nil	Nil	HIVID	WangY et al.2004	14712219	Tumor
6q16			Nil	Nil	HIVID	WangY et al.2004	14712219	Tumor
4p15.1			Nil	Nil	HIVID	WangY et al.2004	14712219	Tumor
18q12			Nil	Nil	HIVID	WangY et al.2004	14712219	Tumor
5q21			Nil	Nil	HIVID	WangY et al.2004	14712219	Tumor
7q35			Nil	Nil	HIVID	WangY et al.2004	14712219	Tumor
3q25			Nil	Nil	HIVID	WangY et al.2004	14712219	Tumor
8q23			Nil	Nil	HIVID	WangY et al.2004	14712219	Tumor
5p13			Nucleoporin	26 kb downstream (opposite)	HIVID	WangY et al.2004	14712219	Tumor
chr1	111480460	2984	CD53/LRIF1	intergenic	HIVID	YanH et al 2015	25627239	Tumor
chr8	60201119	637	TOX	intergenic	HIVID	YanH et al 2015	25627239	Tumor
chr11	66807124	1314	SYT12	intronic	HIVID	YanH et al 2015	25627239	Tumor
chr14	28563411	2984	FOXG1	intergenic	HIVID	YanH et al 2015	25627239	Tumor
chr6	83965334	835	ME1	intronic	HIVID	YanH et al 2015	25627239	Tumor
chr8	41554277	1138	ANK1	exonic	HIVID	YanH et al 2015	25627239	Tumor
chr8	155450	421	RPL23AP53	promoter	HIVID	YanH et al 2015	25627239	Tumor
chr8	35280436	1269	UNC5D	intronic	HIVID	YanH et al 2015	25627239	Tumor
chr8	128764911	660	MYC/PVT1	intergenic	HIVID	YanH et al 2015	25627239	Tumor
chr1	10360	409	DDX11L1,WASH7P	intergenic	HIVID	YanH et al 2015	25627239	Tumor
chr6	83965334	1825	ME1	intronic	HIVID	YanH et al 2015	25627239	Tumor
chr12	94198502	2197	CRADD	intronic	HIVID	YanH et al 2015	25627239	Tumor
chr17	5073365	1757	USP6	intronic	HIVID	YanH et al 2015	25627239	Tumor
chr1	111481230	2000	CD53/LRIF1	intergenic	HIVID	YanH et al 2015	25627239	Tumor
chr11	66807269	1589	SYT12	intronic	HIVID	YanH et al 2015	25627239	Tumor
chr1	25392522	3070	RUNX3/SYF2	intergenic	HIVID	YanH et al 2015	25627239	Tumor
chr2	80612761	704	CTNNA2	intronic	HIVID	YanH et al 2015	25627239	Tumor
chr6	138274873	657	TNFAIP3	intergenic	HIVID	YanH et al 2015	25627239	Tumor
chr1	96375021	1828	FLJ31662/PTBP2	intergenic	HIVID	YanH et al 2015	25627239	Tumor
chr6	143730976	1820	ADAT2	intergenic	HIVID	YanH et al 2015	25627239	Tumor
chr17	19451827	1882	SLC47A1	intronic	HIVID	YanH et al 2015	25627239	Tumor
chr19	4472702	1093	HDGFRP2	intronic	HIVID	YanH et al 2015	25627239	Tumor
chr1	106151059	1882	BC043293(unknown function)	Intronic	HIVID	YanH et al 2015	25627239	Tumor
chr8	54810076	2197	RGS20	intronic	HIVID	YanH et al 2015	25627239	Tumor
chr16	46419804	2803	ANKRD26P1	intergenic	HIVID	YanH et al 2015	25627239	Tumor
chr8	41350157	2279	GOLGA7	intronic	HIVID	YanH et al 2015	25627239	Tumor
chr8	54810086	1226	RGS20	intronic	HIVID	YanH et al 2015	25627239	Tumor
chr19	30278016	1822	C19orf12/CCNE1	intergenic	HIVID	YanH et al 2015	25627239	Tumor
chr2	216248136	2010	FN1	Exonic	HIVID	YanH et al 2015	25627239	Tumor
chr13	84655780	1710	SLITRK	intergenic	HIVID	YanH et al 2015	25627239	Tumor
chr1	111480380	370	BC038672(unknown function)	Intronic	HIVID	YanH et al 2015	25627239	Tumor
chr4	74273516	2464	ALB	intronic	HIVID	YanH et al 2015	25627239	Tumor
chr13	23463774	370	BASP1P1	intergenic	HIVID	YanH et al 2015	25627239	Tumor
chr2	216248919	1932	FN1	Exonic	HIVID	YanH et al 2015	25627239	Tumor
chr15	45462771	2464	SHF	intronic	HIVID	YanH et al 2015	25627239	Tumor
chr17	76531764	2174	DNAH17	intronic	HIVID	YanH et al 2015	25627239	Tumor
chr8	128764911	660	MYC/PVT1	intergenic	HIVID	YanH et al 2015	25627239	Tumor
chr8	128764911	660	MYC/PVT1	intergenic	HIVID	YanH et al 2015	25627239	Tumor
chr17	11427761	3070	SHISA6	intronic	HIVID	YanH et al 2015	25627239	Tumor
chr8	60201121	1907	TOX/CA8	intergenic	HIVID	YanH et al 2015	25627239	Tumor
chr8	128764911	660	MYC/PVT1	intergenic	HIVID	YanH et al 2015	25627239	Tumor
chr19	54156038	1840	DPRX/MIR512-2	intergenic	HIVID	YanH et al 2015	25627239	Tumor
chr19	54182535	657	MIR519E;MIR515-1,MIR515-2	promoter	HIVID	YanH et al 2015	25627239	Tumor
chr8	37645254	1828	PROSC	intergenic	HIVID	YanH et al 2015	25627239	Tumor
chr17	17466824	370	PEMT	intronic	HIVID	YanH et al 2015	25627239	Tumor
chr15	45663260	1798	GATM	intronic	HIVID	YanH et al 2015	25627239	Tumor
chr19	54182535	1807	MIR519E;MIR515-1,MIR515-2	promoter	HIVID	YanH et al 2015	25627239	Tumor
chr8	129229133	2387	PVT1	intergenic	HIVID	YanH et al 2015	25627239	Tumor
chr9	103256893	391	TMEFF1	intronic	HIVID	YanH et al 2015	25627239	Tumor
chr10	135524701	656	DUX2	intergenic	HIVID	YanH et al 2015	25627239	Tumor
chr8	128764911	660	MYC/PVT1	intergenic	HIVID	YanH et al 2015	25627239	Tumor
chr14	28563409	2378	FOXG1	intergenic	HIVID	YanH et al 2015	25627239	Tumor
chr19	4472700	1819	HDGFRP2	intronic	HIVID	YanH et al 2015	25627239	Tumor
chr22	43847861	474	MPPED1	intronic	HIVID	YanH et al 2015	25627239	Tumor
chr15	45663260	757	GATM	intronic	HIVID	YanH et al 2015	25627239	Tumor
chr2	216248944	967	FN1	intronic	HIVID	YanH et al 2015	25627239	Tumor
chr12	95412	1882	FAM138D	intergenic	HIVID	YanH et al 2015	25627239	Tumor
chr22	43847856	1219	MPPED1	intronic	HIVID	YanH et al 2015	25627239	Tumor
chr8	77491248	460	OC100192378	intergenic	HIVID	YanH et al 2015	25627239	Tumor
chr8	35280417	122	UNC5D	intronic	HIVID	YanH et al 2015	25627239	Tumor
chr15	45661997	92	GATM	intronic	HIVID	YanH et al 2015	25627239	Tumor
chr2	216248976	2032	FN1	intronic	HIVID	YanH et al 2015	25627239	Tumor
chr7	63071018	2803	MIR4283-1	intergenic	HIVID	YanH et al 2015	25627239	Tumor
chr8	119205008	1757	SAMD12	UTR3	HIVID	YanH et al 2015	25627239	Tumor
chr12	95339	1818	FAM138D	intergenic	HIVID	YanH et al 2015	25627239	Tumor
chr17	11427797	409	SHISA6	intronic	HIVID	YanH et al 2015	25627239	Tumor

Chromosome	Integration site in host genome	Integration site in virus genome	Gene (distance, bp)	Regions	Methods	Author	PMID	Sample
chr19	36213821	826	KMT2B	intronic	HIVID	YanH et al 2015	25627239	Tumor
chr7	118946560	1822	KCND2	intergenic	HIVID	YanH et al 2015	25627239	Tumor
chr8	37645249	1615	PROSC/GPR124	intergenic	HIVID	YanH et al 2015	25627239	Tumor
chr8	60201142	869	TOX/CA8	intergenic	HIVID	YanH et al 2015	25627239	Tumor
chr8	128764911	660	MYC/PVT1	intergenic	HIVID	YanH et al 2015	25627239	Tumor
chr14	24560713	1757	PCK2	Promoter	HIVID	YanH et al 2015	25627239	Tumor
chr4	91044191	1591	DUX4L4	intergenic	HIVID	YanH et al 2015	25627239	Tumor
chr1	21485152	473	EMBP1	Intergenic	HIVID	YanH et al 2015	25627239	Tumor
chr6	46816782	2225	GPR116	Promoter	HIVID	YanH et al 2015	25627239	Tumor
chr19	6212641	1467	KMT2B	intronic	HIVID	YanH et al 2015	25627239	Tumor
chr5	1295468	2318	TERT	Promoter	HIVID	YanH et al 2015	25627239	Tumor
chr5	10480	1403	intergenic	intergenic	HIVID	YanH et al 2015	25627239	Tumor
chr2	32117435	2477	MEMO1	Intronic	HIVID	YanH et al 2015	25627239	Tumor
chr6	48795259	460	SASH1	Exonic	HIVID	YanH et al 2015	25627239	Tumor
chr17	9239789	120	STX8	Intronic	HIVID	YanH et al 2015	25627239	Tumor
chr5	1295379	1561	TERT	Promoter	HIVID	YanH et al 2015	25627239	Tumor
chr4	74271597	2936	ALB	intronic	HIVID	YanH et al 2015	25627239	Tumor
chr17	9899107	556	Hs.730299	Exonic	HIVID	YanH et al 2015	25627239	Tumor
chr2	42751374	1369	MTA3	Intronic	HIVID	YanH et al 2015	25627239	Tumor
chr5	11784	1546	PLEKHG4B	intergenic	HIVID	YanH et al 2015	25627239	Tumor
chr8	50749782	1625	SNTG1	intergenic	HIVID	YanH et al 2015	25627239	Tumor
chr8	50749782	509	SNTG1	intergenic	HIVID	YanH et al 2015	25627239	Tumor
chr5	1295378	1560	TERT	Promoter	HIVID	YanH et al 2015	25627239	Tumor
chr21	519870	3062	AK311573	intronic	HIVID	YanH et al 2015	25627239	Tumor
chr20	62918375	666	PCMTD2 NONE,PLEKHG4B	intronic	HIVID	YanH et al 2015	25627239	Tumor
chr5	49896	504		intergenic	HIVID	YanH et al 2015	25627239	Tumor
chr4	74271706	24	ALB	intronic	HIVID	YanH et al 2015	25627239	Tumor
chr20	62918250	1486	PCMTD2	intronic	HIVID	YanH et al 2015	25627239	Tumor
chr4	48750056	2911	FRYL	intronic	HIVID	YanH et al 2015	25627239	Tumor
chr17	54794533	509	NOG	intergenic	HIVID	YanH et al 2015	25627239	Tumor
chr4	74271597	2936	ALB	intronic	HIVID	YanH et al 2015	25627239	Tumor
chr8	53919293	396	NPBWR1	intergenic	HIVID	YanH et al 2015	25627239	Tumor
chr19	32790314	1408	ZNF507	intergenic	HIVID	YanH et al 2015	25627239	Tumor
chr19	36212669	1818	KMT2B	intronic	HIVID	YanH et al 2015	25627239	Tumor
chr8	53919293	1826	NPBWR1	intergenic	HIVID	YanH et al 2015	25627239	Tumor
chr19	32790265	1555	ZNF507	intergenic	HIVID	YanH et al 2015	25627239	Tumor
chr21	39208145	1595	KCNJ6	intronic	HIVID	YanH et al 2015	25627239	Tumor
chr8	142911585	1357	MIR4472-1	intergenic	HIVID	YanH et al 2015	25627239	Tumor
chr2	191836589	1412	STAT1	Intronic	HIVID	YanH et al 2015	25627239	Tumor
chr10	135524437	574	DUX2	intergenic	HIVID	YanH et al 2015	25627239	Tumor
chr12	95436	1663	LOC100288778	Promoter	HIVID	YanH et al 2015	25627239	Tumor
chr17	3048221	1342	STXBP4	Intronic	HIVID	YanH et al 2015	25627239	Tumor
chr10	135524609	109	DUX2	intergenic	HIVID	YanH et al 2015	25627239	Tumor
chr5	1295500	460	TERT	Promoter	HIVID	YanH et al 2015	25627239	Tumor
chr10	135524639	1595	DUX2	intronic	HIVID	YanH et al 2015	25627239	Tumor
chr5	1295534	1288	TERT	Promoter	HIVID	YanH et al 2015	25627239	Tumor
chr12	95601	1574	LOC100288778	Promoter	HIVID	YanH et al 2015	25627239	Tumor
chr19	59098041	509	MGC2752(non-conding RNA)	intronic	HIVID	YanH et al 2015	25627239	Tumor
chr8	53048633	1468	ST18	intronic	HIVID	YanH et al 2015	25627239	Tumor
chr11	7886160	1467	SERGEF	intronic	HIVID	YanH et al 2015	25627239	Tumor
chr12	95438	574	LOC100288778	Promoter	HIVID	YanH et al 2015	25627239	Tumor
chr5	1307384	9	MIR4457	Promoter	HIVID	YanH et al 2015	25627239	Tumor
chr5	1295516	400	TERT	Promoter	HIVID	YanH et al 2015	25627239	Tumor
chr12	4368524	663	Hs.577711	Intronic	HIVID	YanH et al 2015	25627239	Tumor
chr2	191836387	3215	STAT1	Intronic	HIVID	YanH et al 2015	25627239	Tumor
chr5	82591572	1387	XRCC4	Intronic	HIVID	YanH et al 2015	25627239	Tumor
chr8	53048618	510	ST18	intronic	HIVID	YanH et al 2015	25627239	Tumor
chr5	1295529	1136	TERT	Promoter	HIVID	YanH et al 2015	25627239	Tumor
chr4	91043848	509	DUX4L4	intergenic	HIVID	YanH et al 2015	25627239	Tumor
chr5	51223925	712	GLRA1	intergenic	HIVID	YanH et al 2015	25627239	Tumor
chr16	69587	509	Hs.721474	Intronic	HIVID	YanH et al 2015	25627239	Tumor
chr5	1295549	911	TERT	Promoter	HIVID	YanH et al 2015	25627239	Tumor
chr4	91043875	666	DUX4L4	intergenic	HIVID	YanH et al 2015	25627239	Tumor
chr5	61056203	2148	GABRB2	intergenic	HIVID	YanH et al 2015	25627239	Tumor
chr16	69784	516	Hs.721474	Intronic	HIVID	YanH et al 2015	25627239	Tumor
chr6	46816782	489	GPR116	promoter	HIVID	YanH et al 2015	25627239	Tumor
chr4	91043883	588	DUX4L4	intergenic	HIVID	YanH et al 2015	25627239	Tumor
chr6	46816770	1244	GPR116	Promoter	HIVID	YanH et al 2015	25627239	Tumor
chr19	6212721	504	KMT2B	intronic	HIVID	YanH et al 2015	25627239	Tumor
chr20	62918250	504	PCMTD2	intronic	HIVID	YanH et al 2015	25627239	Tumor
chr2	191836387	2936	STAT1	Intronic	HIVID	YanH et al 2015	25627239	Tumor
chr22	5465915	137	PHF21B	intergenic	HIVID	YanH et al 2015	25627239	Tumor
chr16	4696050	1902	MGRN1	Intronic	HIVID	YanH et al 2015	25627239	Tumor
chr5	1295541	1442	TERT	Promoter	HIVID	YanH et al 2015	25627239	Tumor
chr2	91836431	1403	STAT1	Intronic	HIVID	YanH et al 2015	25627239	Tumor
chr5	1295741	1831	TERT	Promoter	HIVID	YanH et al 2015	25627239	Tumor
chr3	197900290	432	FAM157A	intronic	HIVID	YanH et al 2015	25627239	Tumor
chr5	1295737	1817	TERT	Promoter	HIVID	YanH et al 2015	25627239	Tumor
chr8	53048426	1589	ST18	intronic	HIVID	YanH et al 2015	25627239	Tumor
chr17	3048060	1532	STXBP4	Intronic	HIVID	YanH et al 2015	25627239	Tumor
chr22	745111	1157	PLXNB2	intronic	HIVID	YanH et al 2015	25627239	Tumor
chr17	9234192	1652	STX8	Intronic	HIVID	YanH et al 2015	25627239	Tumor
chr4	74271706	24	ALB	intronic	HIVID	YanH et al 2015	25627239	Tumor
chr19	36212743	446	KMT2B	intronic	HIVID	YanH et al 2015	25627239	Tumor
chr17	53048158	1561	STXBP4	Intronic	HIVID	YanH et al 2015	25627239	Tumor
chr16	69535	504	Hs.721474	Intronic	HIVID	YanH et al 2015	25627239	Tumor
chr8	53048633	630	ST18	intronic	HIVID	YanH et al 2015	25627239	Tumor
chr5	92171531	1553	LOC100129716	intergenic	HIVID	YanH et al 2015	25627239	Tumor
chr1	25241058	1423	DNAH14	intronic	HIVID	YanH et al 2015	25627239	Tumor
chr17	3048162	1461	STXBP4	Intronic	HIVID	YanH et al 2015	25627239	Tumor
8q13.2	68002219	1815	PREX2	Intron 1	MSRE-qPCR	Zhang et al 2015	25815780	Tumor
4p14	40572076	1821	RBM47	5'UTR	MSRE-qPCR	Zhang et al 2015	25815780	Tumor
20p12.3	5639689	2224			MSRE-qPCR	Zhang et al 2015	25815780	Tumor
4q12	52251977	1621			MSRE-qPCR	Zhang et al 2015	25815780	Tumor
19q13.12	35722065	1808	KMT2B	Exon 3	MSRE-qPCR	Zhang et al 2015	25815780	Tumor
2q31.1	170846582	1820	GAD1	Intron 9	MSRE-qPCR	Zhang et al 2015	25815780	Tumor

Chromosome	Integration site in host genome	Integration site in virus genome	Gene (distance, bp)	Regions	Methods	Author	PMID	Sample
19p13.11	17041317	1486			MSRE-qPCR	Zhang et al 2015	25815780	Tumor
2q11.2	97243605	1786	ANKRD36	Intron 69	MSRE-qPCR	Zhang et al 2015	25815780	Tumor
8q24.21	128187021	1825			MSRE-qPCR	Zhang et al 2015	25815780	Tumor
8q13.2	68002219	1815	PREX2	Intron 1	MSRE-qPCR	Zhang et al 2015	25815780	Tumor
4p14	40572076	1821	RBM47	5'UTR	MSRE-qPCR	Zhang et al 2015	25815780	Tumor
20p12.3	5639689	2230			MSRE-qPCR	Zhang et al 2015	25815780	Tumor
20q11.23	35833665	1612	PHF20	Intron 1	MSRE-qPCR	Zhang et al 2015	25815780	Tumor
4q12	52251977	1621			MSRE-qPCR	Zhang et al 2015	25815780	Tumor
19q13.12	35722065	1808	KMT2B	Exon 3	MSRE-qPCR	Zhang et al 2015	25815780	Tumor
16p11.2	28596750	1832	SULT1A2	5'UTR	MSRE-qPCR	Zhang et al 2015	25815780	Tumor
18q11.2	22003616	1742			MSRE-qPCR	Zhang et al 2015	25815780	Tumor
chr13	109670868		MYO16	intronic	HIVID	Zhao et al.2016	27703150	Tumor
chr13	109653766		MYO16	intronic	HIVID	Zhao et al.2016	27703150	Tumor
chr11	69358936		MYEOV(dist=294182),CCND1(dist=96937)	intergenic	HIVID	Zhao et al.2016	27703150	Tumor
chr11	60274903		MS4A13;MS4A12	promoter;downstream	HIVID	Zhao et al.2016	27703150	Tumor
chr11	65233890		MIR612(dist=21862),MALAT1(dist=31343)	intergenic	HIVID	Zhao et al.2016	27703150	Tumor
chr18	68776842		GTSCR1(dist=458749),RP11-510D19.1(dist=410358)	intergenic	HIVID	Zhao et al.2016	27703150	Tumor
chr6	145215807		UTRN(dist=41637),EPM2A(dist=730633)	intergenic	HIVID	Zhao et al.2016	27703150	Tumor
chr5	130803730		RAPGEF6	intronic	HIVID	Zhao et al.2016	27703150	Tumor
chr11	69340326		MYEOV(dist=275572),CCND1(dist=115547)	intergenic	HIVID	Zhao et al.2016	27703150	Tumor
chr11	69245764		MYEOV(dist=181010),CCND1(dist=210109)	intergenic	HIVID	Zhao et al.2016	27703150	Tumor
chr8	40961100		ZMAT4(dist=205757),SFRP1(dist=158376)	intergenic	HIVID	Zhao et al.2016	27703150	Tumor
chr4	39592356		UGDH-AS1	ncRNA_intronic	HIVID	Zhao et al.2016	27703150	Tumor
chr6	30056297		RNF39(dist=12669),TRIM31(dist=14377)	intergenic	HIVID	Zhao et al.2016	27703150	Tumor
chr6	30056259		RNF39(dist=12631),TRIM31(dist=14415)	intergenic	HIVID	Zhao et al.2016	27703150	Tumor
chr7	22431765		RAPGEF5(dist=35232),STEAP1B(dist=27298)	intergenic	HIVID	Zhao et al.2016	27703150	Tumor
chr2	238528797		LRRFIP1	promoter	HIVID	Zhao et al.2016	27703150	Tumor
chr2	238528754		LRRFIP1	promoter	HIVID	Zhao et al.2016	27703150	Tumor
chr8	42235995		DKK4;POLB	promoter;downstream	HIVID	Zhao et al.2016	27703150	Tumor
chr19	30284003		C19orf12(dist=77307),CCNE1(dist=18898)	intergenic	HIVID	Zhao et al.2016	27703150	Tumor
chr19	30284002		C19orf12(dist=77306),CCNE1(dist=18899)	intergenic	HIVID	Zhao et al.2016	27703150	Tumor
chr19	30283961		C19orf12(dist=77265),CCNE1(dist=18940)	intergenic	HIVID	Zhao et al.2016	27703150	Tumor
chr19	30283929		C19orf12(dist=77233),CCNE1(dist=18972)	intergenic	HIVID	Zhao et al.2016	27703150	Tumor
chr9	29293877		LINGO2(dist=80879),LOC401497(dist=1095056)	intergenic	HIVID	Zhao et al.2016	27703150	Tumor
chr9	29315962		LINGO2(dist=102964),LOC401497(dist=1072971)	intergenic	HIVID	Zhao et al.2016	27703150	Tumor
chr9	28332984		LINGO2	intronic	HIVID	Zhao et al.2016	27703150	Tumor
chr13	84491584		SLITRK1(dist=35056),LINC00333(dist=223153)	intergenic	HIVID	Zhao et al.2016	27703150	Tumor
chr17	11306537		SHISA6	intronic	HIVID	Zhao et al.2016	27703150	Tumor
chr8	53704683		RB1CC1(dist=77657),NPBWR1(dist=147785)	intergenic	HIVID	Zhao et al.2016	27703150	Tumor
chr18	10367		NONE(dist=NONE),ROCK1P1(dist=98698)	intergenic	HIVID	Zhao et al.2016	27703150	Tumor
chr5	8266577		MTRR(dist=365342),LOC729506(dist=67019)	intergenic	HIVID	Zhao et al.2016	27703150	Tumor
chr20	60765364		MTG2	intronic	HIVID	Zhao et al.2016	27703150	Tumor
chr5	10090419		LOC285692(dist=186483),FAM173B(dist=135201)	intergenic	HIVID	Zhao et al.2016	27703150	Tumor
chr5	3308178		LOC102467074(dist=126832),LINC01019(dist=109088)	intergenic	HIVID	Zhao et al.2016	27703150	Tumor
chr15	58874779		LIPC(dist=13706),ADAM10(dist=12624)	intergenic	HIVID	Zhao et al.2016	27703150	Tumor
chr15	58874640		LIPC(dist=13567),ADAM10(dist=12763)	intergenic	HIVID	Zhao et al.2016	27703150	Tumor
chr9	25212630		IZUMO3(dist=666956),TUSC1(dist=463757)	intergenic	HIVID	Zhao et al.2016	27703150	Tumor
chr4	191043995		DUX4(dist=30553),NONE(dist=NONE)	intergenic	HIVID	Zhao et al.2016	27703150	Tumor
chr5	2905501		C5orf38(dist=149990),LOC102467074(dist=272448)	intergenic	HIVID	Zhao et al.2016	27703150	Tumor
chr15	40835863		C15orf57	intronic	HIVID	Zhao et al.2016	27703150	Tumor
chr18	54813064		BOD1L2	promoter	HIVID	Zhao et al.2016	27703150	Tumor
chr5	1296005		TERT	promoter	HIVID	Zhao et al.2016	27703150	Tumor
chr8	167241		RPL23AP53	ncRNA_intronic	HIVID	Zhao et al.2016	27703150	Tumor
chr5	17329		NONE(dist=NONE),PLEKHG4B(dist=123044)	intergenic	HIVID	Zhao et al.2016	27703150	Tumor
chr5	17678		NONE(dist=NONE),PLEKHG4B(dist=122695)	intergenic	HIVID	Zhao et al.2016	27703150	Tumor
chr10	8813509		LINC00708(dist=503241),LINC00709(dist=504067)	intergenic	HIVID	Zhao et al.2016	27703150	Tumor
chr4	83543697		LINC00575;SCD5	promoter;downstream	HIVID	Zhao et al.2016	27703150	Tumor
chr19	36211655		KMT2B	exonic	HIVID	Zhao et al.2016	27703150	Tumor
chr19	36213210		KMT2B	intronic	HIVID	Zhao et al.2016	27703150	Tumor
chr20	53876693		DOK5(dist=608983),RP5-1010E17.2(dist=160181)	intergenic	HIVID	Zhao et al.2016	27703150	Tumor
chr10	110423		TUBB8(dist=15245),ZMYND11(dist=69982)	intergenic	HIVID	Zhao et al.2016	27703150	Tumor
chr20	50511797		RP5-1112F19.2(dist=32345),ZFP64(dist=188753)	intergenic	HIVID	Zhao et al.2016	27703150	Tumor
chr20	50506537		RP5-1112F19.2(dist=27085),ZFP64(dist=194013)	intergenic	HIVID	Zhao et al.2016	27703150	Tumor
chr22	50745111		PLXNB2	intronic	HIVID	Zhao et al.2016	27703150	Tumor
chr18	78016315		PARD6G(dist=10918),NONE(dist=NONE)	intergenic	HIVID	Zhao et al.2016	27703150	Tumor
chr18	78016288		PARD6G(dist=10891),NONE(dist=NONE)	intergenic	HIVID	Zhao et al.2016	27703150	Tumor
chr18	78016281		PARD6G(dist=10884),NONE(dist=NONE)	intergenic	HIVID	Zhao et al.2016	27703150	Tumor
chr18	78016239		PARD6G(dist=10842),NONE(dist=NONE)	intergenic	HIVID	Zhao et al.2016	27703150	Tumor
chr18	78016183		PARD6G(dist=10786),NONE(dist=NONE)	intergenic	HIVID	Zhao et al.2016	27703150	Tumor
chr2	203164193		NOP58	intronic	HIVID	Zhao et al.2016	27703150	Tumor
chr10	62374		NONE(dist=NONE),TUBB8(dist=30454)	intergenic	HIVID	Zhao et al.2016	27703150	Tumor
chr10	62723		NONE(dist=NONE),TUBB8(dist=30105)	intergenic	HIVID	Zhao et al.2016	27703150	Tumor
chr18	16789		NONE(dist=NONE),ROCK1P1(dist=92276)	intergenic	HIVID	Zhao et al.2016	27703150	Tumor
chr18	17138		NONE(dist=NONE),ROCK1P1(dist=91927)	intergenic	HIVID	Zhao et al.2016	27703150	Tumor
chr10	102157438		LINC00263(dist=9327),WNT8B(dist=65374)	intergenic	HIVID	Zhao et al.2016	27703150	Tumor
chr11	68684724		IGHMBP2	intronic	HIVID	Zhao et al.2016	27703150	Tumor
chr7	91273448		FZD1(dist=375316),MTERF(dist=228573)	intergenic	HIVID	Zhao et al.2016	27703150	Tumor
chr20	55114414		FAM209B(dist=2840),TFAP2C(dist=89944)	intergenic	HIVID	Zhao et al.2016	27703150	Tumor
chr1	121355057		EMBP1(dist=41371),NONE(dist=NONE)	intergenic	HIVID	Zhao et al.2016	27703150	Tumor
chr10	135524748		DUX4L7(dist=26290),NONE(dist=NONE)	intergenic	HIVID	Zhao et al.2016	27703150	Tumor
chr10	135524671		DUX4L7(dist=26213),NONE(dist=NONE)	intergenic	HIVID	Zhao et al.2016	27703150	Tumor
chr4	191043680		DUX4(dist=30238),NONE(dist=NONE)	intergenic	HIVID	Zhao et al.2016	27703150	Tumor
chr11	69448974		CCND1	promoter	HIVID	Zhao et al.2016	27703150	Tumor
chr7	66437786		TMEM248(dist=14248),SBDS(dist=14904)	intergenic	HIVID	Zhao et al.2016	27703150	Tumor
chr1	249240298		PGBD2(dist=26953),NONE(dist=NONE)	intergenic	HIVID	Zhao et al.2016	27703150	Tumor
chr1	249239984		PGBD2(dist=26639),NONE(dist=NONE)	intergenic	HIVID	Zhao et al.2016	27703150	Tumor
chr19	36213091		KMT2B	intronic	HIVID	Zhao et al.2016	27703150	Tumor
chr1	10104		DDX11L1;MIR6859-1,MIR6859-2,WASH7P	promoter;downstream	HIVID	Zhao et al.2016	27703150	Tumor
chr11	128995945		ARHGAP32	intronic	HIVID	Zhao et al.2016	27703150	Tumor
chr1	121355702		EMBP1(dist=42016),NONE(dist=NONE)	intergenic	HIVID	Zhao et al.2016	27703150	Tumor
chr5	140999357		DIAPH1;HDAC3	promoter;downstream	HIVID	Zhao et al.2016	27703150	Tumor
chr5	140999325		DIAPH1;HDAC3	promoter;downstream	HIVID	Zhao et al.2016	27703150	Tumor
chr2	145312211		ZEB2-AS1(dist=33746),TEX41(dist=113323)	intergenic	HIVID	Zhao et al.2016	27703150	Tumor
chr3	158257943		RSRC1	intronic	HIVID	Zhao et al.2016	27703150	Tumor
chr3	158254291		RSRC1	intronic	HIVID	Zhao et al.2016	27703150	Tumor
chr7	154724269		PAXIP1-AS2	ncRNA_intronic	HIVID	Zhao et al.2016	27703150	Tumor
chr18	78016327		PARD6G(dist=10930),NONE(dist=NONE)	intergenic	HIVID	Zhao et al.2016	27703150	Tumor

Chromosome	Integration site in host genome	Integration site in virus genome	Gene (distance, bp)	Regions	Methods	Author	PMID	Sample
chr18	78016289		PARD6G(dist=10892),NONE(dist=NONE)	intergenic	HIVID	Zhao et al.2016	27703150	Tumor
chr4	13974		NONE(dist=NONE),ZNF595(dist=39205)	intergenic	HIVID	Zhao et al.2016	27703150	Tumor
chr18	10025		NONE(dist=NONE),ROCK1P1(dist=99040)	intergenic	HIVID	Zhao et al.2016	27703150	Tumor
chr10	42383724		NONE(dist=NONE),LOC441666(dist=443590)	intergenic	HIVID	Zhao et al.2016	27703150	Tumor
chr10	42387672		NONE(dist=NONE),LOC441666(dist=439642)	intergenic	HIVID	Zhao et al.2016	27703150	Tumor
chr17	12628997		MYOCD	intronic	HIVID	Zhao et al.2016	27703150	Tumor
chr9	12986787		LURAP1L(dist=163728),MPDZ(dist=118916)	intergenic	HIVID	Zhao et al.2016	27703150	Tumor
chr9	12982390		LURAP1L(dist=159331),MPDZ(dist=123313)	intergenic	HIVID	Zhao et al.2016	27703150	Tumor
chr12	95491		LOC100288778(dist=4228),FAM138D(dist=52455)	intergenic	HIVID	Zhao et al.2016	27703150	Tumor
chr12	95485		LOC100288778(dist=4222),FAM138D(dist=52461)	intergenic	HIVID	Zhao et al.2016	27703150	Tumor
chr2	156643174		KCNJ3(dist=928310),AC093375.1(dist=233873)	intergenic	HIVID	Zhao et al.2016	27703150	Tumor
chr8	43092879		HGSNAT(dist=34909),POTEA(dist=54706)	intergenic	HIVID	Zhao et al.2016	27703150	Tumor
chr3	197900821		FAM157A	intronic	HIVID	Zhao et al.2016	27703150	Tumor
chr10	135524749		DUX4L7(dist=26291),NONE(dist=NONE)	intergenic	HIVID	Zhao et al.2016	27703150	Tumor
chr10	135524732		DUX4L7(dist=26274),NONE(dist=NONE)	intergenic	HIVID	Zhao et al.2016	27703150	Tumor
chr10	135524696		DUX4L7(dist=26238),NONE(dist=NONE)	intergenic	HIVID	Zhao et al.2016	27703150	Tumor
chr4	191044099		DUX4(dist=30657),NONE(dist=NONE)	intergenic	HIVID	Zhao et al.2016	27703150	Tumor
chr4	191039768		DUX4(dist=26326),NONE(dist=NONE)	intergenic	HIVID	Zhao et al.2016	27703150	Tumor
chr1	10397		DDX11L1;MIR6859-1,MIR6859-2,WASH7P	promoter;downstream	HIVID	Zhao et al.2016	27703150	Tumor
chr2	10238853		CYS1(dist=18315),RRM2(dist=23842)	intergenic	HIVID	Zhao et al.2016	27703150	Tumor
chr4	122744279		CCNA2	intronic	HIVID	Zhao et al.2016	27703150	Tumor
chr4	122743106		CCNA2	intronic	HIVID	Zhao et al.2016	27703150	Tumor
chr4	130666200		C4orf33(dist=632357),NONE(dist=NONE)	intergenic	HIVID	Zhao et al.2016	27703150	Tumor
chr9	66971110		PTGER4P2-CDK2AP2P2(dist=468080),RP11-381O7.3(dist=4626[illegible]	intergenic	HIVID	Zhao et al.2016	27703150	Tumor
chr9	66835090		PTGER4P2-CDK2AP2P2(dist=332060),RP11-381O7.3(dist=1822[illegible]	intergenic	HIVID	Zhao et al.2016	27703150	Tumor
chr9	66835046		PTGER4P2-CDK2AP2P2(dist=332016),RP11-381O7.3(dist=1823[illegible]	intergenic	HIVID	Zhao et al.2016	27703150	Tumor
chr11	55015246		NONE(dist=NONE),TRIM48(dist=14412)	intergenic	HIVID	Zhao et al.2016	27703150	Tumor
chr12	38248612		NONE(dist=NONE),ALG10B(dist=461945)	intergenic	HIVID	Zhao et al.2016	27703150	Tumor
chr12	38248665		NONE(dist=NONE),ALG10B(dist=461892)	intergenic	HIVID	Zhao et al.2016	27703150	Tumor
chr17	22223246		MTRNR2L1(dist=199255),NONE(dist=NONE)	intergenic	HIVID	Zhao et al.2016	27703150	Tumor
chr5	90622230		LUCAT1(dist=12011),ARRDC3(dist=42311)	intergenic	HIVID	Zhao et al.2016	27703150	Tumor
chr3	90313731		EPHA3(dist=782447),NONE(dist=NONE)	intergenic	HIVID	Zhao et al.2016	27703150	Tumor
chr2	133007593		ANKRD30BL	ncRNA_intronic	HIVID	Zhao et al.2016	27703150	Tumor
chr10	58549126		ZWINT(dist=428092),MIR3924(dist=515113)	intergenic	HIVID	Zhao et al.2016	27703150	Tumor
chr14	102341634		PPP2R5C	intronic	HIVID	Zhao et al.2016	27703150	Tumor
chr18	18517299		NONE(dist=NONE),ROCK1(dist=12404)	intergenic	HIVID	Zhao et al.2016	27703150	Tumor
chr18	18519126		NONE(dist=NONE),ROCK1(dist=10577)	intergenic	HIVID	Zhao et al.2016	27703150	Tumor
chr10	42428708		NONE(dist=NONE),LOC441666(dist=398606)	intergenic	HIVID	Zhao et al.2016	27703150	Tumor
chr20	26279175		LOC284801(dist=89306),NONE(dist=NONE)	intergenic	HIVID	Zhao et al.2016	27703150	Tumor
chr3	90474028		EPHA3(dist=942744),NONE(dist=NONE)	intergenic	HIVID	Zhao et al.2016	27703150	Tumor
chr7	143164275		EPHA1-AS1	ncRNA_intronic	HIVID	Zhao et al.2016	27703150	Tumor
chr1	171833290		DNM3	intronic	HIVID	Zhao et al.2016	27703150	Tumor
chr4	122763496		BBS7	intronic	HIVID	Zhao et al.2016	27703150	Tumor
chr4	122759715		BBS7	intronic	HIVID	Zhao et al.2016	27703150	Tumor
chr4	122763407		BBS7	intronic	HIVID	Zhao et al.2016	27703150	Tumor
chr2	92276760		ACTR3BP2(dist=146264),NONE(dist=NONE)	intergenic	HIVID	Zhao et al.2016	27703150	Tumor
chr2	92276682		ACTR3BP2(dist=146186),NONE(dist=NONE)	intergenic	HIVID	Zhao et al.2016	27703150	Tumor
chr7	152979641		ACTR3B(dist=427177),DPP6(dist=604778)	intergenic	HIVID	Zhao et al.2016	27703150	Tumor
chr2	166791621		TTC21B-AS1	ncRNA_intronic	HIVID	Zhao et al.2016	27703150	Tumor
chr9	9314169		PTPRD	intronic	HIVID	Zhao et al.2016	27703150	Tumor
chr9	29975735		LINGO2(dist=762737),LOC401497(dist=413198)	intergenic	HIVID	Zhao et al.2016	27703150	Tumor
chr5	101938880		LINC00492	ncRNA_intronic	HIVID	Zhao et al.2016	27703150	Tumor
chr2	202088189		CASP10	intronic	HIVID	Zhao et al.2016	27703150	Tumor
chr19	30843061		URI1(dist=335542),ZNF536(dist=20267)	intergenic	HIVID	Zhao et al.2016	27703150	Tumor
chr17	19727944		ULK2	intronic	HIVID	Zhao et al.2016	27703150	Tumor
chr2	182140518		UBE2E3(dist=212364),MIR4437(dist=29802)	intergenic	HIVID	Zhao et al.2016	27703150	Tumor
chr20	54484314		RP5-1010E17.2(dist=440579),CBLN4(dist=88099)	intergenic	HIVID	Zhao et al.2016	27703150	Tumor
chr5	11699		NONE(dist=NONE),PLEKHG4B(dist=128674)	intergenic	HIVID	Zhao et al.2016	27703150	Tumor
chr5	11705		NONE(dist=NONE),PLEKHG4B(dist=128668)	intergenic	HIVID	Zhao et al.2016	27703150	Tumor
chr5	11798		NONE(dist=NONE),PLEKHG4B(dist=128575)	intergenic	HIVID	Zhao et al.2016	27703150	Tumor
chr2	243152475		LOC728323(dist=50006),NONE(dist=NONE)	intergenic	HIVID	Zhao et al.2016	27703150	Tumor
chr3	87159286		LINC00506	ncRNA_intronic	HIVID	Zhao et al.2016	27703150	Tumor
chr3	87159333		LINC00506	ncRNA_intronic	HIVID	Zhao et al.2016	27703150	Tumor
chr13	92547780		GPC5	intronic	HIVID	Zhao et al.2016	27703150	Tumor
chr17	19674038		ALDH3A1(dist=22292),ULK2(dist=105)	intergenic	HIVID	Zhao et al.2016	27703150	Tumor
chr1	49433530		AGBL4	intronic	HIVID	Zhao et al.2016	27703150	Tumor
chr10	94923024		CYP26A1(dist=85383),MYOF(dist=143162)	intergenic	HIVID	Zhao et al.2016	27703150	Tumor
chr10	94967163		CYP26A1(dist=129522),MYOF(dist=99023)	intergenic	HIVID	Zhao et al.2016	27703150	Tumor
chr1	230181431		URB2(dist=385485),GALNT2(dist=21525)	intergenic	HIVID	Zhao et al.2016	27703150	Tumor
chr5	1295211		TERT	promoter	HIVID	Zhao et al.2016	27703150	Tumor
chr14	26189070		STXBP6(dist=669975),NOVA1(dist=726019)	intergenic	HIVID	Zhao et al.2016	27703150	Tumor
chr20	43941984		RBPJL	intronic	HIVID	Zhao et al.2016	27703150	Tumor
chr18	78016289		PARD6G(dist=10892),NONE(dist=NONE)	intergenic	HIVID	Zhao et al.2016	27703150	Tumor
chr18	78016183		PARD6G(dist=10786),NONE(dist=NONE)	intergenic	HIVID	Zhao et al.2016	27703150	Tumor
chr2	208683479		FZD5(dist=49336),PLEKHM3(dist=2533)	intergenic	HIVID	Zhao et al.2016	27703150	Tumor
chr3	45963363		FYCO1	intronic	HIVID	Zhao et al.2016	27703150	Tumor
chr3	61174446		FHIT	intronic	HIVID	Zhao et al.2016	27703150	Tumor
chr3	60993851		FHIT	intronic	HIVID	Zhao et al.2016	27703150	Tumor
chr10	135524749		DUX4L7(dist=26291),NONE(dist=NONE)	intergenic	HIVID	Zhao et al.2016	27703150	Tumor
chr10	135524743		DUX4L7(dist=26285),NONE(dist=NONE)	intergenic	HIVID	Zhao et al.2016	27703150	Tumor
chr10	135524699		DUX4L7(dist=26241),NONE(dist=NONE)	intergenic	HIVID	Zhao et al.2016	27703150	Tumor
chr2	114361014		DDX11L2	ncRNA_intronic	HIVID	Zhao et al.2016	27703150	Tumor
chr17	76681321		CYTH1	intronic	HIVID	Zhao et al.2016	27703150	Tumor
chr4	122742757		CCNA2	intronic	HIVID	Zhao et al.2016	27703150	Tumor
chr10	32758788		CCDC7	intronic	HIVID	Zhao et al.2016	27703150	Tumor
chr1	249239977		PGBD2(dist=26632),NONE(dist=NONE)	intergenic	HIVID	Zhao et al.2016	27703150	Tumor
chr1	249239891		PGBD2(dist=26546),NONE(dist=NONE)	intergenic	HIVID	Zhao et al.2016	27703150	Tumor
chr1	226709557		PARP1(dist=113756),C1orf95(dist=26944)	intergenic	HIVID	Zhao et al.2016	27703150	Tumor
chr4	10089		NONE(dist=NONE),ZNF595(dist=43090)	intergenic	HIVID	Zhao et al.2016	27703150	Tumor
chr8	62666701		MIR4470(dist=39283),NKAIN3(dist=494800)	intergenic	HIVID	Zhao et al.2016	27703150	Tumor
chr8	62666643		MIR4470(dist=39225),NKAIN3(dist=494858)	intergenic	HIVID	Zhao et al.2016	27703150	Tumor
chr2	243152501		LOC728323(dist=50032),NONE(dist=NONE)	intergenic	HIVID	Zhao et al.2016	27703150	Tumor
chr2	243152489		LOC728323(dist=50020),NONE(dist=NONE)	intergenic	HIVID	Zhao et al.2016	27703150	Tumor
chr20	62918558		LINC00266-1	promoter	HIVID	Zhao et al.2016	27703150	Tumor

Chromosome	Integration site in host genome	Integration site in virus genome	Gene (distance, bp)	Regions	Methods	Author	PMID	Sample
chr6	58777997		GUSBP4(dist=490273),NONE(dist=NONE)	intergenic	HIVID	Zhao et al.2016	27703150	Tumor
chr4	191044118		DUX4(dist=30676),NONE(dist=NONE)	intergenic	HIVID	Zhao et al.2016	27703150	Tumor
chr1	10093		DDX11L1;MIR6859-1,MIR6859-2,WASH7P	promoter;downstream	HIVID	Zhao et al.2016	27703150	Tumor
chr19	59098075		CENPBD1P1(dist=2313),NONE(dist=NONE)	intergenic	HIVID	Zhao et al.2016	27703150	Tumor
chr16	32156833		ZNF267(dist=228204),HERC2P4(dist=24531)	intergenic	HIVID	Zhao et al.2016	27703150	Tumor
chr16	32118492		ZNF267(dist=189863),HERC2P4(dist=62872)	intergenic	HIVID	Zhao et al.2016	27703150	Tumor
chr16	32118466		ZNF267(dist=189837),HERC2P4(dist=62898)	intergenic	HIVID	Zhao et al.2016	27703150	Tumor
chr16	32832031		TP53TG3B(dist=144583),SLC6A10P(dist=56766)	intergenic	HIVID	Zhao et al.2016	27703150	Tumor
chr16	32793296		TP53TG3B(dist=105848),SLC6A10P(dist=95501)	intergenic	HIVID	Zhao et al.2016	27703150	Tumor
chr5	1296902		TERT	promoter	HIVID	Zhao et al.2016	27703150	Tumor
chr21	10893497		TEKT4P2(dist=924904),TPTE(dist=13246)	intergenic	HIVID	Zhao et al.2016	27703150	Tumor
chr16	33099747		SLC6A10P(dist=203284),TP53TG3C(dist=105838)	intergenic	HIVID	Zhao et al.2016	27703150	Tumor
chr16	33061763		SLC6A10P(dist=165300),TP53TG3C(dist=143822)	intergenic	HIVID	Zhao et al.2016	27703150	Tumor
chr7	61760927		NONE(dist=NONE),ZNF733P(dist=990743)	intergenic	HIVID	Zhao et al.2016	27703150	Tumor
chr2	91661027		NONE(dist=NONE),LOC654342(dist=163682)	intergenic	HIVID	Zhao et al.2016	27703150	Tumor
chr10	42615730		NONE(dist=NONE),LOC441666(dist=211584)	intergenic	HIVID	Zhao et al.2016	27703150	Tumor
chr17	15804205		MEIS3P1(dist=111186),ADORA2B(dist=44026)	intergenic	HIVID	Zhao et al.2016	27703150	Tumor
chr16	34178034		LINC00273(dist=215531),UBE2MP1(dist=225768)	intergenic	HIVID	Zhao et al.2016	27703150	Tumor
chr3	156609394		LEKR1	intronic	HIVID	Zhao et al.2016	27703150	Tumor
chr9	70419973		FOXD4L5(dist=241158),FOXD4L2(dist=6650)	intergenic	HIVID	Zhao et al.2016	27703150	Tumor
chr9	70412045		FOXD4L5(dist=233230),FOXD4L2(dist=14578)	intergenic	HIVID	Zhao et al.2016	27703150	Tumor
chr9	42726993		FOXD4L2(dist=6651),RP11-381O7.3(dist=117377)	intergenic	HIVID	Zhao et al.2016	27703150	Tumor
chr9	42734921		FOXD4L2(dist=14579),RP11-381O7.3(dist=109449)	intergenic	HIVID	Zhao et al.2016	27703150	Tumor
chr9	42734895		FOXD4L2(dist=14553),RP11-381O7.3(dist=109475)	intergenic	HIVID	Zhao et al.2016	27703150	Tumor
chr2	132765560		C2orf27B(dist=206326),ANKRD30BL(dist=139604)	intergenic	HIVID	Zhao et al.2016	27703150	Tumor
chr12	133523592		ZNF605	intronic	HIVID	Zhao et al.2016	27703150	Tumor
chr12	133512087		ZNF605	intronic	HIVID	Zhao et al.2016	27703150	Tumor
chr18	78016327		PARD6G(dist=10930),NONE(dist=NONE)	intergenic	HIVID	Zhao et al.2016	27703150	Tumor
chr18	78016288		PARD6G(dist=10891),NONE(dist=NONE)	intergenic	HIVID	Zhao et al.2016	27703150	Tumor
chr18	78016229		PARD6G(dist=10832),NONE(dist=NONE)	intergenic	HIVID	Zhao et al.2016	27703150	Tumor
chr18	78016187		PARD6G(dist=10790),NONE(dist=NONE)	intergenic	HIVID	Zhao et al.2016	27703150	Tumor
chr5	10478		NONE(dist=NONE),PLEKHG4B(dist=129895)	intergenic	HIVID	Zhao et al.2016	27703150	Tumor
chr12	95450		LOC100288778(dist=4187),FAM138D(dist=52496)	intergenic	HIVID	Zhao et al.2016	27703150	Tumor
chr19	36213615		KMT2B	exonic	HIVID	Zhao et al.2016	27703150	Tumor
chr19	36210996		KMT2B	exonic	HIVID	Zhao et al.2016	27703150	Tumor
chr19	36211019		KMT2B	exonic	HIVID	Zhao et al.2016	27703150	Tumor
chr10	135524749		DUX4L7(dist=26291),NONE(dist=NONE)	intergenic	HIVID	Zhao et al.2016	27703150	Tumor
chr10	135524717		DUX4L7(dist=26259),NONE(dist=NONE)	intergenic	HIVID	Zhao et al.2016	27703150	Tumor
chr10	135524693		DUX4L7(dist=26235),NONE(dist=NONE)	intergenic	HIVID	Zhao et al.2016	27703150	Tumor
chr10	135524589		DUX4L7(dist=26131),NONE(dist=NONE)	intergenic	HIVID	Zhao et al.2016	27703150	Tumor
chr9	10062		DDX11L5;WASH1	promoter;downstream	HIVID	Zhao et al.2016	27703150	Tumor
chr9	10076		DDX11L5;WASH1	promoter;downstream	HIVID	Zhao et al.2016	27703150	Tumor
chr5	1295142		TERT	UTR5	HIVID	Zhao et al.2016	27703150	Tumor
chr15	95191513		MCTP2(dist=164332),LOC440311(dist=207079)	intergenic	HIVID	Zhao et al.2016	27703150	Tumor
chr7	36311496		EEPD1	intronic	HIVID	Zhao et al.2016	27703150	Tumor
chr11	69455211		CCND1	promoter	HIVID	Zhao et al.2016	27703150	Tumor
chr7	118409427		ANKRD7(dist=526643),KCND2(dist=1504295)	intergenic	HIVID	Zhao et al.2016	27703150	Tumor
chr5	1295163		TERT	promoter	HIVID	Zhao et al.2016	27703150	Tumor
chr7	124956705		RP11-3B12.2	ncRNA_intronic	HIVID	Zhao et al.2016	27703150	Tumor
chr10	126262824		LHPP	intronic	HIVID	Zhao et al.2016	27703150	Tumor
chr6	58777742		GUSBP4(dist=490018),NONE(dist=NONE)	intergenic	HIVID	Zhao et al.2016	27703150	Tumor
chr6	58774347		GUSBP4(dist=486623),NONE(dist=NONE)	intergenic	HIVID	Zhao et al.2016	27703150	Tumor
chr17	14975544		CDRT7(dist=40270),CDRT8(dist=32758)	intergenic	HIVID	Zhao et al.2016	27703150	Tumor
chr21	9696247		NONE(dist=NONE),MIR3648(dist=129585)	intergenic	HIVID	Zhao et al.2016	27703150	Tumor
chr4	49514597		CWH43(dist=450502),NONE(dist=NONE)	intergenic	HIVID	Zhao et al.2016	27703150	Tumor
chr1	143282912		ANKRD20A12P(dist=569307),MIR6077-1(dist=390009)	intergenic	HIVID	Zhao et al.2016	27703150	Tumor
chr2	162137457		AC009299.3(dist=31896),PSMD14(dist=27329)	intergenic	HIVID	Zhao et al.2016	27703150	Tumor
chr8	64361355		YTHDF3(dist=236009),RP11-579E24.1(dist=320633)	intergenic	HIVID	Zhao et al.2016	27703150	Tumor
chr10	28924312		WAC(dist=12271),BAMBI(dist=42112)	intergenic	HIVID	Zhao et al.2016	27703150	Tumor
chr5	1298871		TERT	promoter	HIVID	Zhao et al.2016	27703150	Tumor
chr21	10764263		TEKT4P2(dist=795670),TPTE(dist=142480)	intergenic	HIVID	Zhao et al.2016	27703150	Tumor
chr12	23792880		SOX5	intronic	HIVID	Zhao et al.2016	27703150	Tumor
chr3	165292420		SLITRK3(dist=377951),BCHE(dist=198272)	intergenic	HIVID	Zhao et al.2016	27703150	Tumor
chr2	198280664		SF3B1	intronic	HIVID	Zhao et al.2016	27703150	Tumor
chr7	83514110		SEMA3E(dist=235631),SEMA3A(dist=73549)	intergenic	HIVID	Zhao et al.2016	27703150	Tumor
chr7	83510884		SEMA3E(dist=232405),SEMA3A(dist=76775)	intergenic	HIVID	Zhao et al.2016	27703150	Tumor
chr8	43822257		POTEA(dist=603929),NONE(dist=NONE)	intergenic	HIVID	Zhao et al.2016	27703150	Tumor
chr12	33425524		PKP2(dist=375744),SYT10(dist=102824)	intergenic	HIVID	Zhao et al.2016	27703150	Tumor
chr12	33258939		PKP2(dist=209159),SYT10(dist=269409)	intergenic	HIVID	Zhao et al.2016	27703150	Tumor
chr12	33258319		PKP2(dist=208539),SYT10(dist=270029)	intergenic	HIVID	Zhao et al.2016	27703150	Tumor
chr2	195176679		PCGEM1(dist=1535054),AC018799.1(dist=32314)	intergenic	HIVID	Zhao et al.2016	27703150	Tumor
chr13	65260354		OR7E156P(dist=943653),PCDH9(dist=1616612)	intergenic	HIVID	Zhao et al.2016	27703150	Tumor
chr2	184658581		NUP35(dist=632169),ZNF804A(dist=804512)	intergenic	HIVID	Zhao et al.2016	27703150	Tumor
chr4	150838985		NR3C2(dist=1475313),DCLK2(dist=160441)	intergenic	HIVID	Zhao et al.2016	27703150	Tumor
chr5	51026		NONE(dist=NONE),PLEKHG4B(dist=89347)	intergenic	HIVID	Zhao et al.2016	27703150	Tumor
chr8	46840075		NONE(dist=NONE),LINC00293(dist=912433)	intergenic	HIVID	Zhao et al.2016	27703150	Tumor
chr17	22261430		MTRNR2L1(dist=237439),NONE(dist=NONE)	intergenic	HIVID	Zhao et al.2016	27703150	Tumor
chr17	22256945		MTRNR2L1(dist=232954),NONE(dist=NONE)	intergenic	HIVID	Zhao et al.2016	27703150	Tumor
chr17	22256911		MTRNR2L1(dist=232920),NONE(dist=NONE)	intergenic	HIVID	Zhao et al.2016	27703150	Tumor
chr17	22254566		MTRNR2L1(dist=230575),NONE(dist=NONE)	intergenic	HIVID	Zhao et al.2016	27703150	Tumor
chr17	22249811		MTRNR2L1(dist=225820),NONE(dist=NONE)	intergenic	HIVID	Zhao et al.2016	27703150	Tumor
chr17	22247160		MTRNR2L1(dist=223169),NONE(dist=NONE)	intergenic	HIVID	Zhao et al.2016	27703150	Tumor
chr2	221095463		MIR4268(dist=324177),EPHA4(dist=1187284)	intergenic	HIVID	Zhao et al.2016	27703150	Tumor
chr11	50723772		LOC646813(dist=343970),OR4A5(dist=687606)	intergenic	HIVID	Zhao et al.2016	27703150	Tumor
chr11	50723703		LOC646813(dist=343901),OR4A5(dist=687675)	intergenic	HIVID	Zhao et al.2016	27703150	Tumor
chr10	49078925		GLUD1P7(dist=120842),FAM25G(dist=124448)	intergenic	HIVID	Zhao et al.2016	27703150	Tumor
chr10	47371579		FAM35DP	promoter	HIVID	Zhao et al.2016	27703150	Tumor
chr10	46889180		FAM35BP	promoter	HIVID	Zhao et al.2016	27703150	Tumor
chr1	121357878		EMBP1(dist=44192),NONE(dist=NONE)	intergenic	HIVID	Zhao et al.2016	27703150	Tumor
chr1	121354990		EMBP1(dist=41304),NONE(dist=NONE)	intergenic	HIVID	Zhao et al.2016	27703150	Tumor
chr1	121479619		EMBP1(dist=165933),NONE(dist=NONE)	intergenic	HIVID	Zhao et al.2016	27703150	Tumor
chr12	7635416		CD163	intronic	HIVID	Zhao et al.2016	27703150	Tumor
chr12	28517564		CCDC91	intronic	HIVID	Zhao et al.2016	27703150	Tumor
chr7	92973584		CCDC132	intronic	HIVID	Zhao et al.2016	27703150	Tumor

Chromosome	Integration site in host genome	Integration site in virus genome	Gene (distance, bp)	Regions	Methods	Author	PMID	Sample
chr21	47742038		C21orf58	intronic	HIVID	Zhao et al.2016	27703150	Tumor
chr4	103013609		BANK1(dist=17640),SLC39A8(dist=158589)	intergenic	HIVID	Zhao et al.2016	27703150	Tumor
chr4	85351748		AGPAT9(dist=824721),NKX6-1(dist=62688)	intergenic	HIVID	Zhao et al.2016	27703150	Tumor
chr8	43092978		HGSNAT(dist=35008),POTEA(dist=54607)	intergenic	HIVID	Zhao et al.2016	27703150	Tumor
chr8	43092879		HGSNAT(dist=34909),POTEA(dist=54706)	intergenic	HIVID	Zhao et al.2016	27703150	Tumor
chr5	1296800		TERT	promoter	HIVID	Zhao et al.2016	27703150	Tumor
chr17	63226018		RGS9(dist=2197),AXIN2(dist=298665)	intergenic	HIVID	Zhao et al.2016	27703150	Tumor
chr1	66893816		PDE4B(dist=53554),SGIP1(dist=106009)	intergenic	HIVID	Zhao et al.2016	27703150	Tumor
chr1	66871450		PDE4B(dist=31188),SGIP1(dist=128375)	intergenic	HIVID	Zhao et al.2016	27703150	Tumor
chr17	21199265		MAP2K3	intronic	HIVID	Zhao et al.2016	27703150	Tumor
chr5	175362062		CPLX2(dist=51039),THOC3(dist=24472)	intergenic	HIVID	Zhao et al.2016	27703150	Tumor
chr19	30303748		CCNE1	intronic	HIVID	Zhao et al.2016	27703150	Tumor
chr19	56782454		ZSCAN5A(dist=42795),ZNF542(dist=97014)	intergenic	HIVID	Zhao et al.2016	27703150	Tumor
chr19	56779944		ZSCAN5A(dist=40285),ZNF542(dist=99524)	intergenic	HIVID	Zhao et al.2016	27703150	Tumor
chr2	235043985		SPP2(dist=58209),ARL4C(dist=357701)	intergenic	HIVID	Zhao et al.2016	27703150	Tumor
chr2	235043939		SPP2(dist=58163),ARL4C(dist=357747)	intergenic	HIVID	Zhao et al.2016	27703150	Tumor
chr19	56530690		NLRP5	intronic	HIVID	Zhao et al.2016	27703150	Tumor
chr7	109475239		C7orf66(dist=950602),EIF3IP1(dist=124045)	intergenic	HIVID	Zhao et al.2016	27703150	Tumor
chr2	31707284		XDH(dist=69673),SRD5A2(dist=42372)	intergenic	HIVID	Zhao et al.2016	27703150	Tumor
chr17	58111829		TBC1D3P1-DHX40P1(dist=15493),MIR4737(dist=8557)	intergenic	HIVID	Zhao et al.2016	27703150	Tumor
chr2	174836583		SP3	promoter	HIVID	Zhao et al.2016	27703150	Tumor
chr2	174836527		SP3	promoter	HIVID	Zhao et al.2016	27703150	Tumor
chr8	64747483		RP11-579E24.1(dist=49429),LINC00966(dist=538292)	intergenic	HIVID	Zhao et al.2016	27703150	Tumor
chr19	14210505		PRKACA	intronic	HIVID	Zhao et al.2016	27703150	Tumor
chr6	171041130		PDCD2(dist=147350),NONE(dist=NONE)	intergenic	HIVID	Zhao et al.2016	27703150	Tumor
chr4	149025688		NR3C2	intronic	HIVID	Zhao et al.2016	27703150	Tumor
chr8	25641		NONE(dist=NONE),OR4F21(dist=90445)	intergenic	HIVID	Zhao et al.2016	27703150	Tumor
chr17	58189430		LOC653653	promoter	HIVID	Zhao et al.2016	27703150	Tumor
chr9	99722075		HIATL2	ncRNA_intronic	HIVID	Zhao et al.2016	27703150	Tumor
chr9	99722198		HIATL2	ncRNA_intronic	HIVID	Zhao et al.2016	27703150	Tumor
chr9	97190452		HIATL1	intronic	HIVID	Zhao et al.2016	27703150	Tumor
chr17	81172446		FLJ43681	promoter	HIVID	Zhao et al.2016	27703150	Tumor
chr17	78115665		EIF4A3	intronic	HIVID	Zhao et al.2016	27703150	Tumor
chr17	59142773		BCAS3	intronic	HIVID	Zhao et al.2016	27703150	Tumor
chr11	129657137		BARX2(dist=334963),TMEM45B(dist=28604)	intergenic	HIVID	Zhao et al.2016	27703150	Tumor
chr19	30549731		URI1(dist=42212),ZNF536(dist=313597)	intergenic	HIVID	Zhao et al.2016	27703150	Tumor
chr19	30521393		URI1(dist=13874),ZNF536(dist=341935)	intergenic	HIVID	Zhao et al.2016	27703150	Tumor
chr6	118445789		SLC35F1	intronic	HIVID	Zhao et al.2016	27703150	Tumor
chr18	78016289		PARD6G(dist=10892),NONE(dist=NONE)	intergenic	HIVID	Zhao et al.2016	27703150	Tumor
chr18	78016281		PARD6G(dist=10884),NONE(dist=NONE)	intergenic	HIVID	Zhao et al.2016	27703150	Tumor
chr19	30303331		CCNE1	intronic	HIVID	Zhao et al.2016	27703150	Tumor
chr5	1295461		TERT	promoter	HIVID	Zhao et al.2016	27703150	Tumor
chr8	51424027		SNTG1	intronic	HIVID	Zhao et al.2016	27703150	Tumor
chr16	981993		LMF1	intronic	HIVID	Zhao et al.2016	27703150	Tumor
chr16	981450		LMF1	intronic	HIVID	Zhao et al.2016	27703150	Tumor
chr16	952703		LMF1	intronic	HIVID	Zhao et al.2016	27703150	Tumor
chr16	10523240		ATF7IP2	intronic	HIVID	Zhao et al.2016	27703150	Tumor
chr13	79271602		RNF219(dist=38288),LINC00331(dist=89852)	intergenic	HIVID	Zhao et al.2016	27703150	Tumor
chr18	9226780		ANKRD12	intronic	HIVID	Zhao et al.2016	27703150	Tumor
chr5	11487		NONE(dist=NONE),PLEKHG4B(dist=128886)	intergenic	HIVID	Zhao et al.2016	27703150	Tumor
chr5	11511		NONE(dist=NONE),PLEKHG4B(dist=128862)	intergenic	HIVID	Zhao et al.2016	27703150	Tumor
chr8	63064041		MIR4470(dist=436623),NKAIN3(dist=97460)	intergenic	HIVID	Zhao et al.2016	27703150	Tumor
chr2	243152622		LOC728323(dist=50153),NONE(dist=NONE)	intergenic	HIVID	Zhao et al.2016	27703150	Tumor
chr2	243152488		LOC728323(dist=50019),NONE(dist=NONE)	intergenic	HIVID	Zhao et al.2016	27703150	Tumor
chr12	95370		LOC100288778(dist=4107),FAM138D(dist=52576)	intergenic	HIVID	Zhao et al.2016	27703150	Tumor
chr11	175567		LINC01001(dist=43647),SCGB1C1(dist=17513)	intergenic	HIVID	Zhao et al.2016	27703150	Tumor
chr20	62918700		LINC00266-1	promoter	HIVID	Zhao et al.2016	27703150	Tumor
chr20	62918530		LINC00266-1	promoter	HIVID	Zhao et al.2016	27703150	Tumor
chr20	62918604		LINC00266-1	promoter	HIVID	Zhao et al.2016	27703150	Tumor
chr20	62918618		LINC00266-1	promoter	HIVID	Zhao et al.2016	27703150	Tumor
chr20	62918283		LINC00266-1	promoter	HIVID	Zhao et al.2016	27703150	Tumor
chr19	36213938		KMT2B	exonic	HIVID	Zhao et al.2016	27703150	Tumor
chr19	36213532		KMT2B	exonic	HIVID	Zhao et al.2016	27703150	Tumor
chr8	111557524		KCNV1(dist=570565),CSMD3(dist=1677635)	intergenic	HIVID	Zhao et al.2016	27703150	Tumor
chr4	191044089		DUX4(dist=30647),NONE(dist=NONE)	intergenic	HIVID	Zhao et al.2016	27703150	Tumor
chr1	10332		DDX11L1;MIR6859-1,MIR6859-2,WASH7P	promoter;downstream	HIVID	Zhao et al.2016	27703150	Tumor
chr1	10361		DDX11L1;MIR6859-1,MIR6859-2,WASH7P	promoter;downstream	HIVID	Zhao et al.2016	27703150	Tumor
chr1	10459		DDX11L1;MIR6859-1,MIR6859-2,WASH7P	promoter;downstream	HIVID	Zhao et al.2016	27703150	Tumor
chr1	10112		DDX11L1;MIR6859-1,MIR6859-2,WASH7P	promoter;downstream	HIVID	Zhao et al.2016	27703150	Tumor
chr5	1295173		TERT	promoter	HIVID	Zhao et al.2016	27703150	Tumor
chr5	1295206		TERT	promoter	HIVID	Zhao et al.2016	27703150	Tumor
chr1	98590276		MIR137HG(dist=75027),RP5-1070A16.1(dist=85991)	intergenic	HIVID	Zhao et al.2016	27703150	Tumor
chr5	1297784		TERT	promoter	HIVID	Zhao et al.2016	27703150	Tumor
chr17	15319133		TEKT3(dist=74175),TVP23C-CDRT4(dist=20199)	intergenic	HIVID	Zhao et al.2016	27703150	Tumor
chr12	118811165		SUDS3,TAOK3	promoter	HIVID	Zhao et al.2016	27703150	Tumor
chr8	79195239		PEX2(dist=1281959),PKIA(dist=233097)	intergenic	HIVID	Zhao et al.2016	27703150	Tumor
chr11	25521808		LUZP2(dist=417622),ANO3(dist=831870)	intergenic	HIVID	Zhao et al.2016	27703150	Tumor
chr11	25502312		LUZP2(dist=398126),ANO3(dist=851366)	intergenic	HIVID	Zhao et al.2016	27703150	Tumor
chr9	44962529		LINC01189(dist=571113),FAM27C(dist=27707)	intergenic	HIVID	Zhao et al.2016	27703150	Tumor
chr9	46085492		FAM27E2(dist=350509),FAM27E1(dist=300112)	intergenic	HIVID	Zhao et al.2016	27703150	Tumor
chr9	46418825		FAM27E1(dist=31452),KGFLP1(dist=268732)	intergenic	HIVID	Zhao et al.2016	27703150	Tumor
chr9	45699316		FAM27C(dist=707824),FAM27A(dist=27713)	intergenic	HIVID	Zhao et al.2016	27703150	Tumor
chr9	67821907		FAM27B(dist=27718),ANKRD20A1(dist=104854)	intergenic	HIVID	Zhao et al.2016	27703150	Tumor
chr5	31280277		CDH6	intronic	HIVID	Zhao et al.2016	27703150	Tumor
chr5	31279723		CDH6	intronic	HIVID	Zhao et al.2016	27703150	Tumor
chr5	31695810		C5orf22(dist=140645),PDZD2(dist=103221)	intergenic	HIVID	Zhao et al.2016	27703150	Tumor
chr2	133034706		ANKRD30BL(dist=19164),GPR39(dist=139441)	intergenic	HIVID	Zhao et al.2016	27703150	Tumor
chr16	47782621		PHKB(dist=47187),ABCC12(dist=334263)	intergenic	HIVID	Zhao et al.2016	27703150	Tumor
chr4	94303976		GRID2	intronic	HIVID	Zhao et al.2016	27703150	Tumor
chr5	42019936		FBXO4(dist=78264),LOC101926960(dist=135999)	intergenic	HIVID	Zhao et al.2016	27703150	Tumor
chr5	1296050		TERT	promoter	HIVID	Zhao et al.2016	27703150	Tumor
chr21	48119753		PRMT2(dist=34717),NONE(dist=NONE)	intergenic	HIVID	Zhao et al.2016	27703150	Tumor
chr1	249239747		PGBD2(dist=26402),NONE(dist=NONE)	intergenic	HIVID	Zhao et al.2016	27703150	Tumor
chr15	25679816		UBE3A	intronic	HIVID	Zhao et al.2016	27703150	Tumor

Supplementary Table S8 Continued

Chromosome	Integration site in host genome	Integration site in virus genome	Gene (distance, bp)	Regions	Methods	Author	PMID	Sample
chr6	149781946		ZC3H12D	intronic	HIVID	Zhao et al.2016	27703150	Tumor
chr9	73922925		TRPM3(dist=186411),TMEM2(dist=375357)	intergenic	HIVID	Zhao et al.2016	27703150	Tumor
chr6	37196159		TMEM217	intronic	HIVID	Zhao et al.2016	27703150	Tumor
chr20	55676662		TFAP2C(dist=462324),BMP7(dist=67147)	intergenic	HIVID	Zhao et al.2016	27703150	Tumor
chr20	55676620		TFAP2C(dist=462282),BMP7(dist=67189)	intergenic	HIVID	Zhao et al.2016	27703150	Tumor
chr17	56669404		TEX14	intronic	HIVID	Zhao et al.2016	27703150	Tumor
chr10	108707572		SORCS1	intronic	HIVID	Zhao et al.2016	27703150	Tumor
chr3	57850082		SLMAP	intronic	HIVID	Zhao et al.2016	27703150	Tumor
chr3	57850126		SLMAP	intronic	HIVID	Zhao et al.2016	27703150	Tumor
chr18	143188		ROCK1P1(dist=20966),USP14(dist=15295)	intergenic	HIVID	Zhao et al.2016	27703150	Tumor
chr8	53698062		RB1CC1(dist=71036),NPBWR1(dist=154406)	intergenic	HIVID	Zhao et al.2016	27703150	Tumor
chr17	17602387		RAI1	intronic	HIVID	Zhao et al.2016	27703150	Tumor
chr1	14533497		PRDM2(dist=381923),KAZN(dist=391716)	intergenic	HIVID	Zhao et al.2016	27703150	Tumor
chr1	249240561		PGBD2(dist=27216),NONE(dist=NONE)	intergenic	HIVID	Zhao et al.2016	27703150	Tumor
chr1	249239272		PGBD2(dist=25927),NONE(dist=NONE)	intergenic	HIVID	Zhao et al.2016	27703150	Tumor
chr18	10068		NONE(dist=NONE),ROCK1P1(dist=98997)	intergenic	HIVID	Zhao et al.2016	27703150	Tumor
chr5	11580		NONE(dist=NONE),PLEKHG4B(dist=128793)	intergenic	HIVID	Zhao et al.2016	27703150	Tumor
chr2	232330716		NCL,SNORD20,SNORD82	promoter	HIVID	Zhao et al.2016	27703150	Tumor
chr9	138958021		NACC2	intronic	HIVID	Zhao et al.2016	27703150	Tumor
chr16	58143528		MMP15(dist=62724),C16orf80(dist=3969)	intergenic	HIVID	Zhao et al.2016	27703150	Tumor
chr7	137860783		MIR4468(dist=52216),TRIM24(dist=284296)	intergenic	HIVID	Zhao et al.2016	27703150	Tumor
chr7	137860737		MIR4468(dist=52170),TRIM24(dist=284342)	intergenic	HIVID	Zhao et al.2016	27703150	Tumor
chr15	81315462		MESDC1(dist=19117),C15orf26(dist=111182)	intergenic	HIVID	Zhao et al.2016	27703150	Tumor
chr11	40820231		LRRC4C	intronic	HIVID	Zhao et al.2016	27703150	Tumor
chr11	40820182		LRRC4C	intronic	HIVID	Zhao et al.2016	27703150	Tumor
chr14	71976441		LOC145474(dist=20021),SIPA1L1(dist=19588)	intergenic	HIVID	Zhao et al.2016	27703150	Tumor
chr15	93416986		LOC100507217	promoter	HIVID	Zhao et al.2016	27703150	Tumor
chr18	71330209		LOC100505817(dist=313085),FBXO15(dist=410379)	intergenic	HIVID	Zhao et al.2016	27703150	Tumor
chr12	95741		LOC100288778(dist=4478),FAM138D(dist=52205)	intergenic	HIVID	Zhao et al.2016	27703150	Tumor
chr12	95497		LOC100288778(dist=4234),FAM138D(dist=52449)	intergenic	HIVID	Zhao et al.2016	27703150	Tumor
chr12	95442		LOC100288778(dist=4179),FAM138D(dist=52504)	intergenic	HIVID	Zhao et al.2016	27703150	Tumor
chr3	163836133		LINC01192(dist=815044),SI(dist=860553)	intergenic	HIVID	Zhao et al.2016	27703150	Tumor
chr17	19093183		GRAPL(dist=31035),EPN2(dist=47507)	intergenic	HIVID	Zhao et al.2016	27703150	Tumor
chr17	19093064		GRAPL(dist=30916),EPN2(dist=47626)	intergenic	HIVID	Zhao et al.2016	27703150	Tumor
chr17	19091824		GRAPL(dist=29676),EPN2(dist=48866)	intergenic	HIVID	Zhao et al.2016	27703150	Tumor
chr17	19091798		GRAPL(dist=29650),EPN2(dist=48892)	intergenic	HIVID	Zhao et al.2016	27703150	Tumor
chr17	19091705		GRAPL(dist=29557),EPN2(dist=48985)	intergenic	HIVID	Zhao et al.2016	27703150	Tumor
chr17	19015573		GRAP(dist=65237),GRAPL(dist=15209)	intergenic	HIVID	Zhao et al.2016	27703150	Tumor
chr17	19015493		GRAP(dist=65157),GRAPL(dist=15289)	intergenic	HIVID	Zhao et al.2016	27703150	Tumor
chr17	19015454		GRAP(dist=65118),GRAPL(dist=15328)	intergenic	HIVID	Zhao et al.2016	27703150	Tumor
chr17	18967073		GRAP(dist=16737),GRAPL(dist=63709)	intergenic	HIVID	Zhao et al.2016	27703150	Tumor
chr17	18966954		GRAP(dist=16618),GRAPL(dist=63828)	intergenic	HIVID	Zhao et al.2016	27703150	Tumor
chr17	18965722		GRAP(dist=15386),GRAPL(dist=65060)	intergenic	HIVID	Zhao et al.2016	27703150	Tumor
chr17	18965603		GRAP(dist=15267),GRAPL(dist=65179)	intergenic	HIVID	Zhao et al.2016	27703150	Tumor
chr15	42593196		GANC	intronic	HIVID	Zhao et al.2016	27703150	Tumor
chr5	161429517		GABRA1(dist=102552),GABRG2(dist=65131)	intergenic	HIVID	Zhao et al.2016	27703150	Tumor
chr9	20859360		FOCAD	intronic	HIVID	Zhao et al.2016	27703150	Tumor
chr17	16416473		FAM211A(dist=20968),ZNF287(dist=37158)	intergenic	HIVID	Zhao et al.2016	27703150	Tumor
chr17	16416452		FAM211A(dist=20947),ZNF287(dist=37179)	intergenic	HIVID	Zhao et al.2016	27703150	Tumor
chr17	16416437		FAM211A(dist=20932),ZNF287(dist=37194)	intergenic	HIVID	Zhao et al.2016	27703150	Tumor
chr4	111091000		ELOVL6	intronic	HIVID	Zhao et al.2016	27703150	Tumor
chr4	191044073		DUX4(dist=30631),NONE(dist=NONE)	intergenic	HIVID	Zhao et al.2016	27703150	Tumor
chr4	191044049		DUX4(dist=30607),NONE(dist=NONE)	intergenic	HIVID	Zhao et al.2016	27703150	Tumor
chr4	191044005		DUX4(dist=30563),NONE(dist=NONE)	intergenic	HIVID	Zhao et al.2016	27703150	Tumor
chr2	115575619		DPP10	intronic	HIVID	Zhao et al.2016	27703150	Tumor
chr7	51501567		COBL(dist=117052),POM121L12(dist=1601782)	intergenic	HIVID	Zhao et al.2016	27703150	Tumor
chr7	51501521		COBL(dist=117006),POM121L12(dist=1601828)	intergenic	HIVID	Zhao et al.2016	27703150	Tumor
chr22	19540550		CLDN5(dist=27690),LINC00895(dist=13103)	intergenic	HIVID	Zhao et al.2016	27703150	Tumor
chr22	19540511		CLDN5(dist=27651),LINC00895(dist=13142)	intergenic	HIVID	Zhao et al.2016	27703150	Tumor
chr22	19540325		CLDN5(dist=27465),LINC00895(dist=13328)	intergenic	HIVID	Zhao et al.2016	27703150	Tumor
chr19	59118971		CENPBD1P1(dist=23209),NONE(dist=NONE)	intergenic	HIVID	Zhao et al.2016	27703150	Tumor
chr19	59118903		CENPBD1P1(dist=23141),NONE(dist=NONE)	intergenic	HIVID	Zhao et al.2016	27703150	Tumor
chr19	59118665		CENPBD1P1(dist=22903),NONE(dist=NONE)	intergenic	HIVID	Zhao et al.2016	27703150	Tumor
chr17	20301029		CCDC144CP	ncRNA_intronic	HIVID	Zhao et al.2016	27703150	Tumor
chr17	20299817		CCDC144CP	ncRNA_intronic	HIVID	Zhao et al.2016	27703150	Tumor
chr17	18452554		CCDC144B	ncRNA_intronic	HIVID	Zhao et al.2016	27703150	Tumor
chr17	18449828		CCDC144B	ncRNA_intronic	HIVID	Zhao et al.2016	27703150	Tumor
chr17	16669759		CCDC144A	intronic	HIVID	Zhao et al.2016	27703150	Tumor
chr17	16672490		CCDC144A	intronic	HIVID	Zhao et al.2016	27703150	Tumor
chr20	12728563		BTBD3(dist=821320),RP5-1069C8.2(dist=117289)	intergenic	HIVID	Zhao et al.2016	27703150	Tumor
chr20	12728517		BTBD3(dist=821274),RP5-1069C8.2(dist=117335)	intergenic	HIVID	Zhao et al.2016	27703150	Tumor
chr5	172603475		BNIP1(dist=12085),NKX2-5(dist=55632)	intergenic	HIVID	Zhao et al.2016	27703150	Tumor
chr22	51069907		ARSA	promoter	HIVID	Zhao et al.2016	27703150	Tumor
chr3	5219182		ARL8B	intronic	HIVID	Zhao et al.2016	27703150	Tumor
chr1	94715287		ARHGAP29(dist=11980),ABCD3(dist=168646)	intergenic	HIVID	Zhao et al.2016	27703150	Tumor
chr12	133841897		ANHX(dist=29475),NONE(dist=NONE)	intergenic	HIVID	Zhao et al.2016	27703150	Tumor
chr6	11735794		ADTRP	intronic	HIVID	Zhao et al.2016	27703150	Tumor
chr6	11735599		ADTRP	intronic	HIVID	Zhao et al.2016	27703150	Tumor
chr2	146160936		TEX41(dist=326645),PABPC1P2(dist=1183689)	intergenic	HIVID	Zhao et al.2016	27703150	Tumor
chr2	146159722		TEX41(dist=325431),PABPC1P2(dist=1184903)	intergenic	HIVID	Zhao et al.2016	27703150	Tumor
chr8	95521881		KIAA1429	intronic	HIVID	Zhao et al.2016	27703150	Tumor
chr2	118745855		CCDC93	intronic	HIVID	Zhao et al.2016	27703150	Tumor
chr2	118745878		CCDC93	intronic	HIVID	Zhao et al.2016	27703150	Tumor
chr4	177622247		VEGFC	intronic	HIVID	Zhao et al.2016	27703150	Tumor
chr1	249240381		PGBD2(dist=27036),NONE(dist=NONE)	intergenic	HIVID	Zhao et al.2016	27703150	Tumor
chr7	10087		NONE(dist=NONE),LOC100507642(dist=139631)	intergenic	HIVID	Zhao et al.2016	27703150	Tumor
chr12	95625		LOC100288778(dist=4362),FAM138D(dist=52321)	intergenic	HIVID	Zhao et al.2016	27703150	Tumor
chr12	95435		LOC100288778(dist=4172),FAM138D(dist=52511)	intergenic	HIVID	Zhao et al.2016	27703150	Tumor
chr12	95256		LOC100288778(dist=3993),FAM138D(dist=52690)	intergenic	HIVID	Zhao et al.2016	27703150	Tumor
chr5	52258885		ITGA1(dist=9400),ITGA2(dist=26271)	intergenic	HIVID	Zhao et al.2016	27703150	Tumor
chr4	155588438		FGG(dist=54536),LRAT(dist=76725)	intergenic	HIVID	Zhao et al.2016	27703150	Tumor
chr4	155581820		FGG(dist=47918),LRAT(dist=83343)	intergenic	HIVID	Zhao et al.2016	27703150	Tumor
chr1	10106		DDX11L1;MIR6859-1,MIR6859-2,WASH7P	promoter;downstream	HIVID	Zhao et al.2016	27703150	Tumor
chr9	137525252		COL5A1	promoter	HIVID	Zhao et al.2016	27703150	Tumor

Chromosome	Integration site in host genome	Integration site in virus genome	Gene (distance, bp)	Regions	Methods	Author	PMID	Sample
chr3	126962719		C3orf56(dist=45692),RP11-88I21.2(dist=78431)	intergenic	HIVID	Zhao et al.2016	27703150	Tumor
chr4	102785997		BANK1	intronic	HIVID	Zhao et al.2016	27703150	Tumor
chr5	1292007		TERT	intronic	HIVID	Zhao et al.2016	27703150	Tumor
chr5	1291104		TERT	intronic	HIVID	Zhao et al.2016	27703150	Tumor
chr4	68386890		CENPC	intronic	HIVID	Zhao et al.2016	27703150	Tumor
chr4	68386799		CENPC	intronic	HIVID	Zhao et al.2016	27703150	Tumor
chr5	1297171		TERT	promoter	HIVID	Zhao et al.2016	27703150	Tumor
chr7	45821236		SEPT7P2(dist=12619),IGFBP1(dist=106723)	intergenic	HIVID	Zhao et al.2016	27703150	Tumor
chr17	20876654		RP11-344E13.3(dist=68111),USP22(dist=26252)	intergenic	HIVID	Zhao et al.2016	27703150	Tumor
chr1	249240442		PGBD2(dist=27097),NONE(dist=NONE)	intergenic	HIVID	Zhao et al.2016	27703150	Tumor
chr1	249240222		PGBD2(dist=26877),NONE(dist=NONE)	intergenic	HIVID	Zhao et al.2016	27703150	Tumor
chr1	249239954		PGBD2(dist=26609),NONE(dist=NONE)	intergenic	HIVID	Zhao et al.2016	27703150	Tumor
chr6	170941325		PDCD2(dist=47545),NONE(dist=NONE)	intergenic	HIVID	Zhao et al.2016	27703150	Tumor
chr8	124397		OR4F21	promoter	HIVID	Zhao et al.2016	27703150	Tumor
chr5	180786916		OR4F16,OR4F29,OR4F3	promoter	HIVID	Zhao et al.2016	27703150	Tumor
chr15	102408796		OR4F13P(dist=18269),OR4F4(dist=53549)	intergenic	HIVID	Zhao et al.2016	27703150	Tumor
chr19	36338387		NPHS1	intronic	HIVID	Zhao et al.2016	27703150	Tumor
chr18	10092		NONE(dist=NONE),ROCK1P1(dist=98973)	intergenic	HIVID	Zhao et al.2016	27703150	Tumor
chr18	10163		NONE(dist=NONE),ROCK1P1(dist=98902)	intergenic	HIVID	Zhao et al.2016	27703150	Tumor
chr6	114226		NONE(dist=NONE),LINC00266-3(dist=26038)	intergenic	HIVID	Zhao et al.2016	27703150	Tumor
chr12	95491		LOC100288778(dist=4228),FAM138D(dist=52455)	intergenic	HIVID	Zhao et al.2016	27703150	Tumor
chr12	95485		LOC100288778(dist=4222),FAM138D(dist=52461)	intergenic	HIVID	Zhao et al.2016	27703150	Tumor
chr12	95424		LOC100288778(dist=4161),FAM138D(dist=52522)	intergenic	HIVID	Zhao et al.2016	27703150	Tumor
chr17	20712913		LOC100287072(dist=71735),CCDC144NL(dist=53795)	intergenic	HIVID	Zhao et al.2016	27703150	Tumor
chr20	62918184		LINC00266-1	promoter	HIVID	Zhao et al.2016	27703150	Tumor
chr17	21323580		KCNJ12(dist=401),C17orf51(dist=107991)	intergenic	HIVID	Zhao et al.2016	27703150	Tumor
chr5	161086390		GABRB2(dist=111260),GABRA6(dist=26268)	intergenic	HIVID	Zhao et al.2016	27703150	Tumor
chr5	83413651		EDIL3	intronic	HIVID	Zhao et al.2016	27703150	Tumor
chr5	83413728		EDIL3	intronic	HIVID	Zhao et al.2016	27703150	Tumor
chr4	191044042		DUX4(dist=30600),NONE(dist=NONE)	intergenic	HIVID	Zhao et al.2016	27703150	Tumor
chr4	191044030		DUX4(dist=30588),NONE(dist=NONE)	intergenic	HIVID	Zhao et al.2016	27703150	Tumor
chr1	10350		DDX11L1;MIR6859-1,MIR6859-2,WASH7P	promoter;downstream	HIVID	Zhao et al.2016	27703150	Tumor
chr1	10039		DDX11L1;MIR6859-1,MIR6859-2,WASH7P	promoter;downstream	HIVID	Zhao et al.2016	27703150	Tumor
chr1	10247		DDX11L1;MIR6859-1,MIR6859-2,WASH7P	promoter;downstream	HIVID	Zhao et al.2016	27703150	Tumor
chr1	10397		DDX11L1;MIR6859-1,MIR6859-2,WASH7P	promoter;downstream	HIVID	Zhao et al.2016	27703150	Tumor
chr12	120427384		CCDC64	promoter	HIVID	Zhao et al.2016	27703150	Tumor
chr8	50079987		C8orf22(dist=91345),SNTG1(dist=742362)	intergenic	HIVID	Zhao et al.2016	27703150	Tumor
chr5	175957501		RNF44	intronic	HIVID	Zhao et al.2016	27703150	Tumor
chr5	175957551		RNF44	intronic	HIVID	Zhao et al.2016	27703150	Tumor
chr13	36887303		SPG20	intronic	HIVID	Zhao et al.2016	27703150	Tumor
chr13	36887274		SPG20	intronic	HIVID	Zhao et al.2016	27703150	Tumor
chr13	28770879		PAN3	intronic	HIVID	Zhao et al.2016	27703150	Tumor
chr13	28769165		PAN3	intronic	HIVID	Zhao et al.2016	27703150	Tumor
chr3	139568020		NMNAT3(dist=171135),CLSTN2(dist=86007)	intergenic	HIVID	Zhao et al.2016	27703150	Tumor
chr3	139567986		NMNAT3(dist=171101),CLSTN2(dist=86041)	intergenic	HIVID	Zhao et al.2016	27703150	Tumor
chr7	74496509		WBSCR16	promoter	HIVID	Zhao et al.2016	27703150	Tumor
chr7	74496884		WBSCR16	promoter	HIVID	Zhao et al.2016	27703150	Tumor
chr7	40788892		SUGCT	intronic	HIVID	Zhao et al.2016	27703150	Tumor
chr12	98853156		SLC9A7P1	promoter	HIVID	Zhao et al.2016	27703150	Tumor
chr12	98852510		SLC9A7P1	promoter	HIVID	Zhao et al.2016	27703150	Tumor
chr1	249240028		PGBD2(dist=26683),NONE(dist=NONE)	intergenic	HIVID	Zhao et al.2016	27703150	Tumor
chr20	21996243		PAX1(dist=297119),RP11-125P18.1(dist=38485)	intergenic	HIVID	Zhao et al.2016	27703150	Tumor
chr15	24092011		NDN(dist=159561),PWRN2(dist=317915)	intergenic	HIVID	Zhao et al.2016	27703150	Tumor
chr7	74279718		GTF2IRD2(dist=11846),STAG3L2(dist=18374)	intergenic	HIVID	Zhao et al.2016	27703150	Tumor
chr7	74279351		GTF2IRD2(dist=11479),STAG3L2(dist=18741)	intergenic	HIVID	Zhao et al.2016	27703150	Tumor
chr9	10228		DDX11L5;WASH1	promoter;downstream	HIVID	Zhao et al.2016	27703150	Tumor
chr9	10285		DDX11L5;WASH1	promoter;downstream	HIVID	Zhao et al.2016	27703150	Tumor
chr5	27201524		CDH9(dist=162835),LINC01021(dist=270875)	intergenic	HIVID	Zhao et al.2016	27703150	Tumor
chr15	62361661		C2CD4A	UTR3	HIVID	Zhao et al.2016	27703150	Tumor
chr5	7523347		ADCY2	intronic	HIVID	Zhao et al.2016	27703150	Tumor
chr8	71888214		XKR9(dist=240037),EYA1(dist=221454)	intergenic	HIVID	Zhao et al.2016	27703150	Tumor
chr8	36624420		UNC5D(dist=972239),KCNU1(dist=17422)	intergenic	HIVID	Zhao et al.2016	27703150	Tumor
chr2	40104589		THUMPD2(dist=98173),SLC8A1-AS1(dist=40185)	intergenic	HIVID	Zhao et al.2016	27703150	Tumor
chr17	37291832		PLXDC1	intronic	HIVID	Zhao et al.2016	27703150	Tumor
chr16	70081297		PDXDC2P	ncRNA_intronic	HIVID	Zhao et al.2016	27703150	Tumor
chr1	17581924		PADI3	intronic	HIVID	Zhao et al.2016	27703150	Tumor
chr19	36213915		KMT2B	exonic	HIVID	Zhao et al.2016	27703150	Tumor
chr11	38512020		C11orf74(dist=1831179),LRRC4C(dist=1623731)	intergenic	HIVID	Zhao et al.2016	27703150	Tumor
chr17	29207531		ATAD5	intronic	HIVID	Zhao et al.2016	27703150	Tumor
chr11	110523389		ARHGAP20	intronic	HIVID	Zhao et al.2016	27703150	Tumor
chr11	105468270		CARD18(dist=457809),GRIA4(dist=12530)	intergenic	HIVID	Zhao et al.2016	27703150	Tumor
chr5	60653212		ZSWIM6	intronic	HIVID	Zhao et al.2016	27703150	Tumor
chr5	60653126		ZSWIM6	intronic	HIVID	Zhao et al.2016	27703150	Tumor
chr8	141374255		TRAPPC9	intronic	HIVID	Zhao et al.2016	27703150	Tumor
chr13	24227741		TNFRSF19	intronic	HIVID	Zhao et al.2016	27703150	Tumor
chr9	8034425		TMEM261(dist=234626),PTPRD(dist=279821)	intergenic	HIVID	Zhao et al.2016	27703150	Tumor
chr5	1295393		TERT	promoter	HIVID	Zhao et al.2016	27703150	Tumor
chr9	27243573		TEK(dist=13401),LINC00032(dist=2109)	intergenic	HIVID	Zhao et al.2016	27703150	Tumor
chr1	185218692		SWT1	intronic	HIVID	Zhao et al.2016	27703150	Tumor
chr9	136356040		SLC2A6(dist=11764),TMEM8C(dist=23668)	intergenic	HIVID	Zhao et al.2016	27703150	Tumor
chr12	130923006		RIMBP2	exonic	HIVID	Zhao et al.2016	27703150	Tumor
chr1	30324282		PTPRU(dist=670957),MATN1(dist=859842)	intergenic	HIVID	Zhao et al.2016	27703150	Tumor
chr9	10384090		PTPRD	intronic	HIVID	Zhao et al.2016	27703150	Tumor
chr19	52731682		PPP2R1A(dist=2004),ZNF766(dist=41142)	intergenic	HIVID	Zhao et al.2016	27703150	Tumor
chr1	40005106		PPIEL	ncRNA_intronic	HIVID	Zhao et al.2016	27703150	Tumor
chr4	31581363		PCDH7(dist=432940),NONE(dist=NONE)	intergenic	HIVID	Zhao et al.2016	27703150	Tumor
chr7	56427893		NUPR1L(dist=243803),LOC650226(dist=63504)	intergenic	HIVID	Zhao et al.2016	27703150	Tumor
chr17	9132609		NTN1	intronic	HIVID	Zhao et al.2016	27703150	Tumor
chr8	84837528		NONE(dist=NONE),RALYL(dist=257925)	intergenic	HIVID	Zhao et al.2016	27703150	Tumor
chr4	61389758		NONE(dist=NONE),LPHN3(dist=973081)	intergenic	HIVID	Zhao et al.2016	27703150	Tumor
chr4	61389782		NONE(dist=NONE),LPHN3(dist=973057)	intergenic	HIVID	Zhao et al.2016	27703150	Tumor
chr17	5597627		NLRP1(dist=109795),LOC339166(dist=77927)	intergenic	HIVID	Zhao et al.2016	27703150	Tumor
chr3	195536566		MUC4	intronic	HIVID	Zhao et al.2016	27703150	Tumor
chr7	63203994		MIR4283-1(dist=122447),LINC01005(dist=280802)	intergenic	HIVID	Zhao et al.2016	27703150	Tumor

Chromosome	Integration site in host genome	Integration site in virus genome	Gene (distance, bp)	Regions	Methods	Author	PMID	Sample
chr4	128393992		MIR2054(dist=1965530),INTU(dist=160095)	intergenic	HIVID	Zhao et al.2016	27703150	Tumor
chr14	62636074		LINC00644(dist=29383),KCNH5(dist=537217)	intergenic	HIVID	Zhao et al.2016	27703150	Tumor
chr2	143787787		KYNU	intronic	HIVID	Zhao et al.2016	27703150	Tumor
chr9	111144564		KLF4(dist=892517),ACTL7B(dist=472305)	intergenic	HIVID	Zhao et al.2016	27703150	Tumor
chr6	3921508		FAM50B(dist=69957),PRPF4B(dist=100061)	intergenic	HIVID	Zhao et al.2016	27703150	Tumor
chr13	43453033		FAM216B(dist=87348),EPSTI1(dist=7491)	intergenic	HIVID	Zhao et al.2016	27703150	Tumor
chr1	44730274		ERI3	intronic	HIVID	Zhao et al.2016	27703150	Tumor
chr9	1575912		DMRT2(dist=518358),SMARCA2(dist=439307)	intergenic	HIVID	Zhao et al.2016	27703150	Tumor
chr16	76590785		CNTNAP4	intronic	HIVID	Zhao et al.2016	27703150	Tumor
chr22	19183316		CLTCL1	intronic	HIVID	Zhao et al.2016	27703150	Tumor
chr12	2016917		CACNA2D4	intronic	HIVID	Zhao et al.2016	27703150	Tumor
chr19	16607635		C19orf44	intronic	HIVID	Zhao et al.2016	27703150	Tumor
chr1	91316454		BARHL2(dist=133660),ZNF644(dist=64403)	intergenic	HIVID	Zhao et al.2016	27703150	Tumor
chr5	90894076		ARRDC3-AS1(dist=177544),NR2F1-AS1(dist=1850986)	intergenic	HIVID	Zhao et al.2016	27703150	Tumor
chr2	133027212		ANKRD30BL(dist=11670),GPR39(dist=146935)	intergenic	HIVID	Zhao et al.2016	27703150	Tumor
chr2	4051325		AC107070.1(dist=29703),AC022311.1(dist=624483)	intergenic	HIVID	Zhao et al.2016	27703150	Tumor
chr2	12420980		AC096559.1	ncRNA_intronic	HIVID	Zhao et al.2016	27703150	Tumor
chr7	48348631		ABCA13	intronic	HIVID	Zhao et al.2016	27703150	Tumor
chr4	171366311		AADAT(dist=354773),RP11-344G13.1(dist=595442)	intergenic	HIVID	Zhao et al.2016	27703150	Tumor
chr7	123145762		IQUB	intronic	HIVID	Zhao et al.2016	27703150	Tumor
chr5	1298669		TERT	promoter	HIVID	Zhao et al.2016	27703150	Tumor
chr11	9681486		SWAP70	promoter	HIVID	Zhao et al.2016	27703150	Tumor
chr1	14627426		PRDM2(dist=475852),KAZN(dist=297787)	intergenic	HIVID	Zhao et al.2016	27703150	Tumor
chr14	44619512		NONE(dist=NONE),FSCB(dist=353842)	intergenic	HIVID	Zhao et al.2016	27703150	Tumor
chr9	25783281		TUSC1(dist=104425),LOC100506422(dist=283392)	intergenic	HIVID	Zhao et al.2016	27703150	Tumor
chr17	7578702		TP53	UTR5	HIVID	Zhao et al.2016	27703150	Tumor
chr17	7578681		TP53	UTR5	HIVID	Zhao et al.2016	27703150	Tumor
chr17	7578646		TP53	UTR5	HIVID	Zhao et al.2016	27703150	Tumor
chr8	66456154		LOC286186	ncRNA_intronic	HIVID	Zhao et al.2016	27703150	Tumor
chr8	66455966		LOC286186	ncRNA_intronic	HIVID	Zhao et al.2016	27703150	Tumor
chr4	189542292		LINC01060(dist=19230),FRG1(dist=1319682)	intergenic	HIVID	Zhao et al.2016	27703150	Tumor
chr5	72266465		FCHO2	intronic	HIVID	Zhao et al.2016	27703150	Tumor
chr4	38881808		FAM114A1	intronic	HIVID	Zhao et al.2016	27703150	Tumor
chr17	46935421		CALCOCO2	intronic	HIVID	Zhao et al.2016	27703150	Tumor
chr9	76045846		ANXA1(dist=260539),MIR6130(dist=322194)	intergenic	HIVID	Zhao et al.2016	27703150	Tumor
chr8	43828013		POTEA(dist=609685),NONE(dist=NONE)	intergenic	HIVID	Zhao et al.2016	27703150	Tumor
chr8	46838977		NONE(dist=NONE),LINC00293(dist=913531)	intergenic	HIVID	Zhao et al.2016	27703150	Tumor
chr10	76337294		ADK	intronic	HIVID	Zhao et al.2016	27703150	Tumor
chr7	57619915		ZNF716(dist=86650),NONE(dist=NONE)	intergenic	HIVID	Zhao et al.2016	27703150	Tumor
chr7	80847585		SEMA3C(dist=298918),HGF(dist=483859)	intergenic	HIVID	Zhao et al.2016	27703150	Tumor
chr7	80843203		SEMA3C(dist=294536),HGF(dist=488241)	intergenic	HIVID	Zhao et al.2016	27703150	Tumor
chr19	36212910		KMT2B	intronic	HIVID	Zhao et al.2016	27703150	Tumor
chr19	36212885		KMT2B	intronic	HIVID	Zhao et al.2016	27703150	Tumor
chr8	77156095		HNF4G(dist=677034),LINC01111(dist=162794)	intergenic	HIVID	Zhao et al.2016	27703150	Tumor
chr2	136882268		CXCR4	promoter	HIVID	Zhao et al.2016	27703150	Tumor
chr8	102002488		YWHAZ(dist=36865),FLJ42969(dist=61794)	intergenic	HIVID	Zhao et al.2016	27703150	Tumor
chr6	83095303		TPBG(dist=18170),UBE3D(dist=506883)	intergenic	HIVID	Zhao et al.2016	27703150	Tumor
chr18	65464355		RP11-638L3.1	ncRNA_intronic	HIVID	Zhao et al.2016	27703150	Tumor
chr18	65462957		RP11-638L3.1	ncRNA_intronic	HIVID	Zhao et al.2016	27703150	Tumor
chr3	77287826		ROBO2	intronic	HIVID	Zhao et al.2016	27703150	Tumor
chr8	114283575		CSMD3	intronic	HIVID	Zhao et al.2016	27703150	Tumor
chr5	19908649		CDH18	intronic	HIVID	Zhao et al.2016	27703150	Tumor
chr12	101348806		ANO4	intronic	HIVID	Zhao et al.2016	27703150	Tumor
chr8	132492876		ADCY8(dist=440041),EFR3A(dist=423480)	intergenic	HIVID	Zhao et al.2016	27703150	Tumor
chr6	139601279		TXLNB	intronic	HIVID	Zhao et al.2016	27703150	Tumor
chr8	55395507		SOX17(dist=22051),RP1(dist=133120)	intergenic	HIVID	Zhao et al.2016	27703150	Tumor
chr16	90182666		PRDM7(dist=40328),NONE(dist=NONE)	intergenic	HIVID	Zhao et al.2016	27703150	Tumor
chr7	21060		NONE(dist=NONE),LOC100507642(dist=128658)	intergenic	HIVID	Zhao et al.2016	27703150	Tumor
chr6	124808257		NKAIN2	intronic	HIVID	Zhao et al.2016	27703150	Tumor
chr1	39858709		MACF1	intronic	HIVID	Zhao et al.2016	27703150	Tumor
chr11	180435		LINC01001(dist=48515),SCGB1C1(dist=12645)	intergenic	HIVID	Zhao et al.2016	27703150	Tumor
chr13	22612977		LINC00424(dist=160678),LINC00540(dist=171447)	intergenic	HIVID	Zhao et al.2016	27703150	Tumor
chr13	22612943		LINC00424(dist=160644),LINC00540(dist=171481)	intergenic	HIVID	Zhao et al.2016	27703150	Tumor
chr9	141121328		FAM157B	intronic	HIVID	Zhao et al.2016	27703150	Tumor
chr3	197894513		FAM157A	intronic	HIVID	Zhao et al.2016	27703150	Tumor
chr5	1289847		TERT	intronic	HIVID	Zhao et al.2016	27703150	Tumor
chr19	59022628		SLC27A5	intronic	HIVID	Zhao et al.2016	27703150	Tumor
chr8	43825065		POTEA(dist=606737),NONE(dist=NONE)	intergenic	HIVID	Zhao et al.2016	27703150	Tumor
chr1	249239989		PGBD2(dist=26644),NONE(dist=NONE)	intergenic	HIVID	Zhao et al.2016	27703150	Tumor
chr8	46839143		NONE(dist=NONE),LINC00293(dist=913365)	intergenic	HIVID	Zhao et al.2016	27703150	Tumor
chr17	22252047		MTRNR2L1(dist=228056),NONE(dist=NONE)	intergenic	HIVID	Zhao et al.2016	27703150	Tumor
chr6	53914188		MLIP	intronic	HIVID	Zhao et al.2016	27703150	Tumor
chr12	95279		LOC100288778(dist=4016),FAM138D(dist=52667)	intergenic	HIVID	Zhao et al.2016	27703150	Tumor
chr9	34510039		DNAI1	intronic	HIVID	Zhao et al.2016	27703150	Tumor
chr11	90832873		DISC1FP1(dist=184653),FAT3(dist=1252389)	intergenic	HIVID	Zhao et al.2016	27703150	Tumor
chr2	211463891		CPS1	intronic	HIVID	Zhao et al.2016	27703150	Tumor
chr2	98856030		VWA3B	intronic	HIVID	Zhao et al.2016	27703150	Tumor
chr7	127721392		SND1	intronic	HIVID	Zhao et al.2016	27703150	Tumor
chr3	196625733		SENP5	intronic	HIVID	Zhao et al.2016	27703150	Tumor
chr4	66175588		RP11-707A18.1(dist=305370),EPHA5(dist=9693)	intergenic	HIVID	Zhao et al.2016	27703150	Tumor
chr1	112258402		RAP1A(dist=2301),FAM212B(dist=6284)	intergenic	HIVID	Zhao et al.2016	27703150	Tumor
chr10	42387304		NONE(dist=NONE),LOC441666(dist=440010)	intergenic	HIVID	Zhao et al.2016	27703150	Tumor
chr10	42387776		NONE(dist=NONE),LOC441666(dist=439538)	intergenic	HIVID	Zhao et al.2016	27703150	Tumor
chr2	34835102		MYADML(dist=881818),LOC100288911(dist=1746790)	intergenic	HIVID	Zhao et al.2016	27703150	Tumor
chr13	84862716		LINC00333	ncRNA_intronic	HIVID	Zhao et al.2016	27703150	Tumor
chr19	36213669		KMT2B	intronic	HIVID	Zhao et al.2016	27703150	Tumor
chr10	15926764		FAM188A(dist=24245),PTER(dist=552178)	intergenic	HIVID	Zhao et al.2016	27703150	Tumor
chr10	15926713		FAM188A(dist=24194),PTER(dist=552229)	intergenic	HIVID	Zhao et al.2016	27703150	Tumor
chr11	70292876		CTTN(dist=10186),SHANK2(dist=21085)	intergenic	HIVID	Zhao et al.2016	27703150	Tumor
chr11	70292875		CTTN(dist=10185),SHANK2(dist=21086)	intergenic	HIVID	Zhao et al.2016	27703150	Tumor
chr11	70292806		CTTN(dist=10116),SHANK2(dist=21155)	intergenic	HIVID	Zhao et al.2016	27703150	Tumor
chr11	70292781		CTTN(dist=10091),SHANK2(dist=21180)	intergenic	HIVID	Zhao et al.2016	27703150	Tumor
chr3	3446566		CRBN(dist=225165),LRRN1(dist=394555)	intergenic	HIVID	Zhao et al.2016	27703150	Tumor
chr19	23374186		ZNF730(dist=44172),ZNF724P(dist=32421)	intergenic	HIVID	Zhao et al.2016	27703150	Tumor

Chromosome	Integration site in host genome	Integration site in virus genome	Gene (distance, bp)	Regions	Methods	Author	PMID	Sample
chr19	12672770		ZNF564(dist=10414),ZNF490(dist=14150)	intergenic	HIVID	Zhao et al.2016	27703150	Tumor
chr9	35405078		UNC13B	UTR3	HIVID	Zhao et al.2016	27703150	Tumor
chr9	35381884		UNC13B	intronic	HIVID	Zhao et al.2016	27703150	Tumor
chr9	35405074		UNC13B	UTR3	HIVID	Zhao et al.2016	27703150	Tumor
chr9	35392051		UNC13B	intronic	HIVID	Zhao et al.2016	27703150	Tumor
chr11	68814436		TPCN2	promoter	HIVID	Zhao et al.2016	27703150	Tumor
chr11	68823485		TPCN2	intronic	HIVID	Zhao et al.2016	27703150	Tumor
chr2	193067980		TMEFF2	promoter	HIVID	Zhao et al.2016	27703150	Tumor
chr5	1295329		TERT	promoter	HIVID	Zhao et al.2016	27703150	Tumor
chr7	23918973		STK31(dist=46843),NPY(dist=404834)	intergenic	HIVID	Zhao et al.2016	27703150	Tumor
chr19	11173617		SMARCA4(dist=659),LDLR(dist=26421)	intergenic	HIVID	Zhao et al.2016	27703150	Tumor
chr9	35975460		OR2S2(dist=17309),RECK(dist=61450)	intergenic	HIVID	Zhao et al.2016	27703150	Tumor
chr9	35975121		OR2S2(dist=16970),RECK(dist=61789)	intergenic	HIVID	Zhao et al.2016	27703150	Tumor
chr9	35972234		OR2S2(dist=14083),RECK(dist=64676)	intergenic	HIVID	Zhao et al.2016	27703150	Tumor
chr9	35970260		OR2S2(dist=12109),RECK(dist=66650)	intergenic	HIVID	Zhao et al.2016	27703150	Tumor
chr1	248851906		OR14I1	promoter	HIVID	Zhao et al.2016	27703150	Tumor
chr9	35890279		OR13J1(dist=19881),HRCT1(dist=15910)	intergenic	HIVID	Zhao et al.2016	27703150	Tumor
chr9	35786559		NPR2	promoter	HIVID	Zhao et al.2016	27703150	Tumor
chr18	18519295		NONE(dist=NONE),ROCK1(dist=10408)	intergenic	HIVID	Zhao et al.2016	27703150	Tumor
chr5	43577293		NNT-AS1	ncRNA_intronic	HIVID	Zhao et al.2016	27703150	Tumor
chr11	69258649		MYEOV(dist=193895),CCND1(dist=197224)	intergenic	HIVID	Zhao et al.2016	27703150	Tumor
chr11	68751353		MRGPRD	promoter	HIVID	Zhao et al.2016	27703150	Tumor
chr11	123294165		MIR4493(dist=41945),GRAMD1B(dist=102179)	intergenic	HIVID	Zhao et al.2016	27703150	Tumor
chr9	31671745		LOC401497(dist=1263293),ACO1(dist=712856)	intergenic	HIVID	Zhao et al.2016	27703150	Tumor
chr11	68737025		IGHMBP2(dist=28956),MRGPRD(dist=10465)	intergenic	HIVID	Zhao et al.2016	27703150	Tumor
chr5	45693544		HCN1	intronic	HIVID	Zhao et al.2016	27703150	Tumor
chr11	69598033		FGF4	promoter	HIVID	Zhao et al.2016	27703150	Tumor
chr11	69590385		FGF4	promoter	HIVID	Zhao et al.2016	27703150	Tumor
chr11	69659983		FGF3(dist=25791),ANO1-AS2(dist=258557)	intergenic	HIVID	Zhao et al.2016	27703150	Tumor
chr11	69657720		FGF3(dist=23528),ANO1-AS2(dist=260820)	intergenic	HIVID	Zhao et al.2016	27703150	Tumor
chr11	69652356		FGF3(dist=18164),ANO1-AS2(dist=266184)	intergenic	HIVID	Zhao et al.2016	27703150	Tumor
chr11	70086068		FADD(dist=32560),PPFIA1(dist=30738)	intergenic	HIVID	Zhao et al.2016	27703150	Tumor
chr19	56213216		EPN1(dist=6083),NLRP9(dist=6582)	intergenic	HIVID	Zhao et al.2016	27703150	Tumor
chr19	56213062		EPN1(dist=5929),NLRP9(dist=6736)	intergenic	HIVID	Zhao et al.2016	27703150	Tumor
chr8	132936196		EFR3A	intronic	HIVID	Zhao et al.2016	27703150	Tumor
chr11	68640779		CPT1A(dist=31380),MRPL21(dist=17967)	intergenic	HIVID	Zhao et al.2016	27703150	Tumor
chr11	68626754		CPT1A(dist=17355),MRPL21(dist=31992)	intergenic	HIVID	Zhao et al.2016	27703150	Tumor
chr9	35629332		CD72(dist=10908),SIT1(dist=19965)	intergenic	HIVID	Zhao et al.2016	27703150	Tumor
chr11	69475216		CCND1(dist=5974),ORAOV1(dist=5116)	intergenic	HIVID	Zhao et al.2016	27703150	Tumor
chr11	69475132		CCND1(dist=5890),ORAOV1(dist=5200)	intergenic	HIVID	Zhao et al.2016	27703150	Tumor
chr11	69475108		CCND1(dist=5866),ORAOV1(dist=5224)	intergenic	HIVID	Zhao et al.2016	27703150	Tumor
chr11	57001420		APLNR	UTR3	HIVID	Zhao et al.2016	27703150	Tumor
chr11	70030139		ANO1	intronic	HIVID	Zhao et al.2016	27703150	Tumor
chr11	70024475		ANO1	intronic	HIVID	Zhao et al.2016	27703150	Tumor
chr10	39125646		ACTR3BP5(dist=134275),NONE(dist=NONE)	intergenic	HIVID	Zhao et al.2016	27703150	Tumor
chr2	92320128		ACTR3BP2(dist=189632),NONE(dist=NONE)	intergenic	HIVID	Zhao et al.2016	27703150	Tumor
chr2	92275968		ACTR3BP2(dist=145472),NONE(dist=NONE)	intergenic	HIVID	Zhao et al.2016	27703150	Tumor
chr5	1298026		TERT	promoter	HIVID	Zhao et al.2016	27703150	Tumor
chr5	1295677		TERT	promoter	HIVID	Zhao et al.2016	27703150	Tumor
chr4	54201437		SCFD2	intronic	HIVID	Zhao et al.2016	27703150	Tumor
chr4	54201620		SCFD2	intronic	HIVID	Zhao et al.2016	27703150	Tumor
chr7	125391102		RP11-3B12.2(dist=371727),GRM8(dist=687550)	intergenic	HIVID	Zhao et al.2016	27703150	Tumor
chr7	125390972		RP11-3B12.2(dist=371597),GRM8(dist=687680)	intergenic	HIVID	Zhao et al.2016	27703150	Tumor
chr4	55089508		PDGFRA	promoter	HIVID	Zhao et al.2016	27703150	Tumor
chr18	18517560		NONE(dist=NONE),ROCK1(dist=12143)	intergenic	HIVID	Zhao et al.2016	27703150	Tumor
chr18	18517612		NONE(dist=NONE),ROCK1(dist=12091)	intergenic	HIVID	Zhao et al.2016	27703150	Tumor
chr10	42385121		NONE(dist=NONE),LOC441666(dist=442193)	intergenic	HIVID	Zhao et al.2016	27703150	Tumor
chr10	42388554		NONE(dist=NONE),LOC441666(dist=438760)	intergenic	HIVID	Zhao et al.2016	27703150	Tumor
chr10	42390692		NONE(dist=NONE),LOC441666(dist=436622)	intergenic	HIVID	Zhao et al.2016	27703150	Tumor
chr19	27890679		NONE(dist=NONE),LINC00662(dist=390722)	intergenic	HIVID	Zhao et al.2016	27703150	Tumor
chr19	27890719		NONE(dist=NONE),LINC00662(dist=390682)	intergenic	HIVID	Zhao et al.2016	27703150	Tumor
chr14	64878429		MIR548AZ	ncRNA_intronic	HIVID	Zhao et al.2016	27703150	Tumor
chr2	92269185		ACTR3BP2(dist=138689),NONE(dist=NONE)	intergenic	HIVID	Zhao et al.2016	27703150	Tumor
chr12	119320260		SUDS3(dist=464420),SRRM4(dist=99040)	intergenic	HIVID	Zhao et al.2016	27703150	Tumor
chr6	67527518		SLC25A51P1(dist=1028142),BAI3(dist=1818114)	intergenic	HIVID	Zhao et al.2016	27703150	Tumor
chr9	32305418		LOC401497(dist=1896966),ACO1(dist=79183)	intergenic	HIVID	Zhao et al.2016	27703150	Tumor
chr3	163825628		LINC01192(dist=804539),SI(dist=871058)	intergenic	HIVID	Zhao et al.2016	27703150	Tumor
chr17	50912563		CA10(dist=675186),C17orf112(dist=150317)	intergenic	HIVID	Zhao et al.2016	27703150	Tumor
chr5	1295505		TERT	promoter	HIVID	Zhao et al.2016	27703150	Tumor
chr5	1291131		TERT	intronic	HIVID	Zhao et al.2016	27703150	Tumor
chr5	1287442		TERT	intronic	HIVID	Zhao et al.2016	27703150	Tumor
chr13	105879662		MIR548AS(dist=1944814),DAOA-AS1(dist=231744)	intergenic	HIVID	Zhao et al.2016	27703150	Tumor
chr18	33450975		GALNT1(dist=159177),MIR187(dist=33806)	intergenic	HIVID	Zhao et al.2016	27703150	Tumor
chr4	10962242		CLNK(dist=275856),MIR572(dist=408209)	intergenic	HIVID	Zhao et al.2016	27703150	Tumor
chr5	1296527		TERT	promoter	HIVID	Zhao et al.2016	27703150	Tumor
chr1	246588454		SMYD3	intronic	HIVID	Zhao et al.2016	27703150	Tumor
chr5	26265310		RP11-730N24.1(dist=1424618),CDH9(dist=615399)	intergenic	HIVID	Zhao et al.2016	27703150	Tumor
chr5	14870		NONE(dist=NONE),PLEKHG4B(dist=125503)	intergenic	HIVID	Zhao et al.2016	27703150	Tumor
chr16	46419804		NONE(dist=NONE),ANKRD26P1(dist=83445)	intergenic	HIVID	Zhao et al.2016	27703150	Tumor
chr7	57034020		MIR4283-1(dist=10449),ZNF479(dist=153306)	intergenic	HIVID	Zhao et al.2016	27703150	Tumor
chr7	63071017		LOC100287834(dist=211598),MIR4283-1(dist=10451)	intergenic	HIVID	Zhao et al.2016	27703150	Tumor
chr10	135524746		DUX4L7(dist=26288),NONE(dist=NONE)	intergenic	HIVID	Zhao et al.2016	27703150	Tumor
chr21	10819575		TEKT4P2(dist=850982),TPTE(dist=87168)	intergenic	HIVID	Zhao et al.2016	27703150	Tumor
chr17	22258207		MTRNR2L1(dist=234216),NONE(dist=NONE)	intergenic	HIVID	Zhao et al.2016	27703150	Tumor
chr17	22255829		MTRNR2L1(dist=231838),NONE(dist=NONE)	intergenic	HIVID	Zhao et al.2016	27703150	Tumor
chr2	89872144		MIR4436A(dist=760176),LOC654342(dist=1952565)	intergenic	HIVID	Zhao et al.2016	27703150	Tumor
chr2	89872095		MIR4436A(dist=760127),LOC654342(dist=1952614)	intergenic	HIVID	Zhao et al.2016	27703150	Tumor
chr19	36212974		KMT2B	intronic	HIVID	Zhao et al.2016	27703150	Tumor
chr19	36212907		KMT2B	intronic	HIVID	Zhao et al.2016	27703150	Tumor
chr4	49109775		CWH43(dist=45680),NONE(dist=NONE)	intergenic	HIVID	Zhao et al.2016	27703150	Tumor
chr16	46510297		ANKRD26P1	ncRNA_intronic	HIVID	Zhao et al.2016	27703150	Tumor
chr5	1295556		TERT	promoter	HIVID	Zhao et al.2016	27703150	Tumor
chr13	87817825		SLITRK6(dist=1444342),MIR4500HG(dist=278417)	intergenic	HIVID	Zhao et al.2016	27703150	Tumor
chr20	38716725		RP11-101E14.2(dist=79554),MAFB(dist=597763)	intergenic	HIVID	Zhao et al.2016	27703150	Tumor

Supplementary Table S8 Continued

Chromosome	Integration site in host genome	Integration site in virus genome	Gene (distance, bp)	Regions	Methods	Author	PMID	Sample
chr1	67452497		MIER1	UTR3	HIVID	Zhao et al.2016	27703150	Tumor
chr15	99847272		LRRC28	intronic	HIVID	Zhao et al.2016	27703150	Tumor
chr15	99847308		LRRC28	intronic	HIVID	Zhao et al.2016	27703150	Tumor
chr4	180744065		LINC01098(dist=1832161),LINC00290(dist=1241178)	intergenic	HIVID	Zhao et al.2016	27703150	Tumor
chr4	171733444		AADAT(dist=721906),RP11-344G13.1(dist=228309)	intergenic	HIVID	Zhao et al.2016	27703150	Tumor
chr5	1295723		TERT	promoter	HIVID	Zhao et al.2016	27703150	Tumor
chr5	1295713		TERT	promoter	HIVID	Zhao et al.2016	27703150	Tumor
chr19	27732081		NONE(dist=NONE),LINC00662(dist=549320)	intergenic	HIVID	Zhao et al.2016	27703150	Tumor
chr1	121485099		EMBP1(dist=171413),NONE(dist=NONE)	intergenic	HIVID	Zhao et al.2016	27703150	Tumor
chr1	121484080		EMBP1(dist=170394),NONE(dist=NONE)	intergenic	HIVID	Zhao et al.2016	27703150	Tumor
chr8	38953040		ADAM9	intronic	HIVID	Zhao et al.2016	27703150	Tumor
chr8	38966498		ADAM32	intronic	HIVID	Zhao et al.2016	27703150	Tumor
chr8	43094785		HGSNAT(dist=36815),POTEA(dist=52800)	intergenic	HIVID	Zhao et al.2016	27703150	Tumor
chr10	135524737		DUX4L7(dist=26279),NONE(dist=NONE)	intergenic	HIVID	Zhao et al.2016	27703150	Tumor
chr7	57572816		ZNF716(dist=39551),NONE(dist=NONE)	intergenic	HIVID	Zhao et al.2016	27703150	Tumor
chr7	57572763		ZNF716(dist=39498),NONE(dist=NONE)	intergenic	HIVID	Zhao et al.2016	27703150	Tumor
chr8	81682992		ZNF704	intronic	HIVID	Zhao et al.2016	27703150	Tumor
chr8	81751349		ZNF704	intronic	HIVID	Zhao et al.2016	27703150	Tumor
chr2	217733796		TNP1	promoter	HIVID	Zhao et al.2016	27703150	Tumor
chr5	1296011		TERT	promoter	HIVID	Zhao et al.2016	27703150	Tumor
chr18	10526		NONE(dist=NONE),ROCK1P1(dist=98539)	intergenic	HIVID	Zhao et al.2016	27703150	Tumor
chr18	10532		NONE(dist=NONE),ROCK1P1(dist=98533)	intergenic	HIVID	Zhao et al.2016	27703150	Tumor
chr18	10554		NONE(dist=NONE),ROCK1P1(dist=98511)	intergenic	HIVID	Zhao et al.2016	27703150	Tumor
chr5	11492		NONE(dist=NONE),PLEKHG4B(dist=128881)	intergenic	HIVID	Zhao et al.2016	27703150	Tumor
chr5	11516		NONE(dist=NONE),PLEKHG4B(dist=128857)	intergenic	HIVID	Zhao et al.2016	27703150	Tumor
chr5	11575		NONE(dist=NONE),PLEKHG4B(dist=128798)	intergenic	HIVID	Zhao et al.2016	27703150	Tumor
chr8	62666701		MIR4470(dist=39283),NKAIN3(dist=494800)	intergenic	HIVID	Zhao et al.2016	27703150	Tumor
chr8	62666624		MIR4470(dist=39206),NKAIN3(dist=494877)	intergenic	HIVID	Zhao et al.2016	27703150	Tumor
chr5	141281008		LOC729080	promoter	HIVID	Zhao et al.2016	27703150	Tumor
chr12	95718		LOC100288778(dist=4455),FAM138D(dist=52228)	intergenic	HIVID	Zhao et al.2016	27703150	Tumor
chr12	95544		LOC100288778(dist=4281),FAM138D(dist=52402)	intergenic	HIVID	Zhao et al.2016	27703150	Tumor
chr12	95532		LOC100288778(dist=4269),FAM138D(dist=52414)	intergenic	HIVID	Zhao et al.2016	27703150	Tumor
chr12	95502		LOC100288778(dist=4239),FAM138D(dist=52444)	intergenic	HIVID	Zhao et al.2016	27703150	Tumor
chr1	10022		DDX11L1;MIR6859-1,MIR6859-2,WASH7P	promoter;downstream	HIVID	Zhao et al.2016	27703150	Tumor
chr5	1297918		TERT	promoter	HIVID	Zhao et al.2016	27703150	Tumor
chr11	126244219		ST3GAL4	intronic	HIVID	Zhao et al.2016	27703150	Tumor
chr8	106634614		ZFPM2	intronic	HIVID	Zhao et al.2016	27703150	Tumor
chr5	1300473		TERT;MIR4457	promoter;downstream	HIVID	Zhao et al.2016	27703150	Tumor
chr1	249240608		PGBD2(dist=27263),NONE(dist=NONE)	intergenic	HIVID	Zhao et al.2016	27703150	Tumor
chr5	11495		NONE(dist=NONE),PLEKHG4B(dist=128878)	intergenic	HIVID	Zhao et al.2016	27703150	Tumor
chr5	11581		NONE(dist=NONE),PLEKHG4B(dist=128792)	intergenic	HIVID	Zhao et al.2016	27703150	Tumor
chr5	13331		NONE(dist=NONE),PLEKHG4B(dist=127042)	intergenic	HIVID	Zhao et al.2016	27703150	Tumor
chr7	10036		NONE(dist=NONE),LOC100507642(dist=139682)	intergenic	HIVID	Zhao et al.2016	27703150	Tumor
chr7	10085		NONE(dist=NONE),LOC100507642(dist=139633)	intergenic	HIVID	Zhao et al.2016	27703150	Tumor
chr2	243152478		LOC728323(dist=50009),NONE(dist=NONE)	intergenic	HIVID	Zhao et al.2016	27703150	Tumor
chr12	95739		LOC100288778(dist=4476),FAM138D(dist=52207)	intergenic	HIVID	Zhao et al.2016	27703150	Tumor
chr12	95477		LOC100288778(dist=4214),FAM138D(dist=52469)	intergenic	HIVID	Zhao et al.2016	27703150	Tumor
chr12	95433		LOC100288778(dist=4170),FAM138D(dist=52513)	intergenic	HIVID	Zhao et al.2016	27703150	Tumor
chr16	55419353		IRX6(dist=54681),MMP2(dist=93728)	intergenic	HIVID	Zhao et al.2016	27703150	Tumor
chr1	10007		DDX11L1;MIR6859-1,MIR6859-2,WASH7P	promoter;downstream	HIVID	Zhao et al.2016	27703150	Tumor
chr13	72567990		DACH1(dist=126660),MZT1(dist=714505)	intergenic	HIVID	Zhao et al.2016	27703150	Tumor
chr8	43835966		POTEA(dist=617638),NONE(dist=NONE)	intergenic	HIVID	Zhao et al.2016	27703150	Tumor
chr8	46853785		NONE(dist=NONE),LINC00293(dist=898723)	intergenic	HIVID	Zhao et al.2016	27703150	Tumor
chr21	21759010		AL109763.2(dist=1626880),LINC00320(dist=355898)	intergenic	HIVID	Zhao et al.2016	27703150	Tumor
chr9	122899169		BRINP1(dist=767430),MIR147A(dist=108088)	intergenic	HIVID	Zhao et al.2016	27703150	Tumor
chr9	122899136		BRINP1(dist=767397),MIR147A(dist=108121)	intergenic	HIVID	Zhao et al.2016	27703150	Tumor
chr17	800078		NXN	intronic	HIVID	Zhao et al.2016	27703150	Tumor
chr19	23387775		ZNF730(dist=57761),ZNF724P(dist=18832)	intergenic	HIVID	Zhao et al.2016	27703150	Tumor
chr5	1295767		TERT	promoter	HIVID	Zhao et al.2016	27703150	Tumor
chr12	66204424		RPSAP52	ncRNA_intronic	HIVID	Zhao et al.2016	27703150	Tumor
chr12	66204457		RPSAP52	ncRNA_intronic	HIVID	Zhao et al.2016	27703150	Tumor
chr13	25660843		PABPC3	promoter	HIVID	Zhao et al.2016	27703150	Tumor
chr19	195556		OR4F17(dist=83960),LINC01002(dist=1460)	intergenic	HIVID	Zhao et al.2016	27703150	Tumor
chr7	61077927		NONE(dist=NONE),ZNF733P(dist=1673743)	intergenic	HIVID	Zhao et al.2016	27703150	Tumor
chr10	42384953		NONE(dist=NONE),LOC441666(dist=442361)	intergenic	HIVID	Zhao et al.2016	27703150	Tumor
chr10	42388337		NONE(dist=NONE),LOC441666(dist=438977)	intergenic	HIVID	Zhao et al.2016	27703150	Tumor
chr10	42396855		NONE(dist=NONE),LOC441666(dist=430459)	intergenic	HIVID	Zhao et al.2016	27703150	Tumor
chr10	42597106		NONE(dist=NONE),LOC441666(dist=230208)	intergenic	HIVID	Zhao et al.2016	27703150	Tumor
chr15	20120731		NONE(dist=NONE),CHEK2P2(dist=367266)	intergenic	HIVID	Zhao et al.2016	27703150	Tumor
chr8	2411345		MYOM2(dist=317965),CSMD1(dist=381530)	intergenic	HIVID	Zhao et al.2016	27703150	Tumor
chr7	100209988		MOSPD3	intronic	HIVID	Zhao et al.2016	27703150	Tumor
chr12	66451372		MIR6074(dist=33866),LLPH(dist=65477)	intergenic	HIVID	Zhao et al.2016	27703150	Tumor
chr20	17970300		MGME1	intronic	HIVID	Zhao et al.2016	27703150	Tumor
chr20	17970361		MGME1	intronic	HIVID	Zhao et al.2016	27703150	Tumor
chr1	255427		LOC729737(dist=114861),LOC100133331(dist=68465)	intergenic	HIVID	Zhao et al.2016	27703150	Tumor
chr5	180756540		LOC100133331(dist=1344),OR4F16(dist=37748)	intergenic	HIVID	Zhao et al.2016	27703150	Tumor
chr19	23480439		LOC100132815(dist=23386),ZNF91(dist=60059)	intergenic	HIVID	Zhao et al.2016	27703150	Tumor
chr19	23480192		LOC100132815(dist=23139),ZNF91(dist=60306)	intergenic	HIVID	Zhao et al.2016	27703150	Tumor
chr16	55295833		IRX5(dist=327438),IRX6(dist=62638)	intergenic	HIVID	Zhao et al.2016	27703150	Tumor
chr16	55285414		IRX5(dist=317019),IRX6(dist=73057)	intergenic	HIVID	Zhao et al.2016	27703150	Tumor
chr16	55284730		IRX5(dist=316335),IRX6(dist=73741)	intergenic	HIVID	Zhao et al.2016	27703150	Tumor
chr16	55280177		IRX5(dist=311782),IRX6(dist=78294)	intergenic	HIVID	Zhao et al.2016	27703150	Tumor
chr4	59817037		IGFBP7-AS1(dist=1745572),NONE(dist=NONE)	intergenic	HIVID	Zhao et al.2016	27703150	Tumor
chr6	160521754		IGF2R	intronic	HIVID	Zhao et al.2016	27703150	Tumor
chr1	91853008		HFM1	intronic	HIVID	Zhao et al.2016	27703150	Tumor
chr19	24561159		HAVCR1P1(dist=214910),NONE(dist=NONE)	intergenic	HIVID	Zhao et al.2016	27703150	Tumor
chr19	24561083		HAVCR1P1(dist=214834),NONE(dist=NONE)	intergenic	HIVID	Zhao et al.2016	27703150	Tumor
chr6	58329493		GUSBP4(dist=41769),NONE(dist=NONE)	intergenic	HIVID	Zhao et al.2016	27703150	Tumor
chr11	69593160		FGF4	promoter	HIVID	Zhao et al.2016	27703150	Tumor
chr11	69554420		FGF19(dist=35314),FGF4(dist=33377)	intergenic	HIVID	Zhao et al.2016	27703150	Tumor
chr1	155287180		FDPS	intronic	HIVID	Zhao et al.2016	27703150	Tumor
chr19	36135668		ETV2	exonic	HIVID	Zhao et al.2016	27703150	Tumor
chr4	49582451		CWH43(dist=518356),NONE(dist=NONE)	intergenic	HIVID	Zhao et al.2016	27703150	Tumor
chr6	75956171		COX7A2;TMEM30A	promoter;downstream	HIVID	Zhao et al.2016	27703150	Tumor

Chromosome	Integration site in host genome	Integration site in virus genome	Gene (distance, bp)	Regions	Methods	Author	PMID	Sample
chr6	75956191		COX7A2;TMEM30A	promoter;downstream	HIVID	Zhao et al.2016	27703150	Tumor
chr11	69463394		CCND1	intronic	HIVID	Zhao et al.2016	27703150	Tumor
chr6	7742003		BMP6	intronic	HIVID	Zhao et al.2016	27703150	Tumor
chr16	403697		AXIN1	promoter	HIVID	Zhao et al.2016	27703150	Tumor
chr1	143421546		ANKRD20A12P(dist=707941),MIR6077-1(dist=251375)	intergenic	HIVID	Zhao et al.2016	27703150	Tumor
chr1	143166416		ANKRD20A12P(dist=452811),MIR6077-1(dist=506505)	intergenic	HIVID	Zhao et al.2016	27703150	Tumor
chr19	22486366		ZNF729	intronic	HIVID	Zhao et al.2016	27703150	Tumor
chr9	25852179		TUSC1(dist=173323),LOC100506422(dist=214494)	intergenic	HIVID	Zhao et al.2016	27703150	Tumor
chr5	1295338		TERT	promoter	HIVID	Zhao et al.2016	27703150	Tumor
chr19	18482700		PGPEP1(dist=1937),GDF15(dist=14268)	intergenic	HIVID	Zhao et al.2016	27703150	Tumor
chr12	67630		NONE(dist=NONE),LOC100288778(dist=20354)	intergenic	HIVID	Zhao et al.2016	27703150	Tumor
chr17	22230164		MTRNR2L1(dist=206173),NONE(dist=NONE)	intergenic	HIVID	Zhao et al.2016	27703150	Tumor
chr19	23505464		LOC100132815(dist=48411),ZNF91(dist=35034)	intergenic	HIVID	Zhao et al.2016	27703150	Tumor
chr3	84836276		LINC00971	ncRNA_intronic	HIVID	Zhao et al.2016	27703150	Tumor
chr3	84833936		LINC00971	ncRNA_intronic	HIVID	Zhao et al.2016	27703150	Tumor
chr15	102495111		FAM138E	ncRNA_exonic	HIVID	Zhao et al.2016	27703150	Tumor
chr9	35842		FAM138C	ncRNA_exonic	HIVID	Zhao et al.2016	27703150	Tumor
chr2	114334982		FAM138B	ncRNA_exonic	HIVID	Zhao et al.2016	27703150	Tumor
chr19	77668		FAM138A,FAM138F	ncRNA_exonic	HIVID	Zhao et al.2016	27703150	Tumor
chr1	36059		FAM138A,FAM138F	ncRNA_exonic	HIVID	Zhao et al.2016	27703150	Tumor
chr4	56357471		CLOCK	intronic	HIVID	Zhao et al.2016	27703150	Tumor
chr9	21960812		C9orf53;CDKN2A	promoter;downstream	HIVID	Zhao et al.2016	27703150	Tumor
chr5	1295345		TERT	promoter	HIVID	Zhao et al.2016	27703150	Tumor
chr3	159357913		IQCJ-SCHIP1,SCHIP1	intronic	HIVID	Zhao et al.2016	27703150	Tumor
chr3	159358050		IQCJ-SCHIP1,SCHIP1	intronic	HIVID	Zhao et al.2016	27703150	Tumor
chr20	43066110		HNF4A(dist=4625),RP5-1013A22.2(dist=11307)	intergenic	HIVID	Zhao et al.2016	27703150	Tumor
chr3	155670602		GMPS(dist=15082),KCNAB1(dist=167735)	intergenic	HIVID	Zhao et al.2016	27703150	Tumor
chr4	122744484		CCNA2	intronic	HIVID	Zhao et al.2016	27703150	Tumor
chr4	122743582		CCNA2	exonic	HIVID	Zhao et al.2016	27703150	Tumor
chr20	43102668		C20orf62,TTPAL	promoter	HIVID	Zhao et al.2016	27703150	Tumor
chr6	38912788		RP1-207H1.3	ncRNA_intronic	HIVID	Zhao et al.2016	27703150	Tumor
chr5	10481		NONE(dist=NONE),PLEKHG4B(dist=129892)	intergenic	HIVID	Zhao et al.2016	27703150	Tumor
chr5	10504		NONE(dist=NONE),PLEKHG4B(dist=129869)	intergenic	HIVID	Zhao et al.2016	27703150	Tumor
chr5	11580		NONE(dist=NONE),PLEKHG4B(dist=128793)	intergenic	HIVID	Zhao et al.2016	27703150	Tumor
chr5	11684		NONE(dist=NONE),PLEKHG4B(dist=128689)	intergenic	HIVID	Zhao et al.2016	27703150	Tumor
chr5	11705		NONE(dist=NONE),PLEKHG4B(dist=128668)	intergenic	HIVID	Zhao et al.2016	27703150	Tumor
chr5	11785		NONE(dist=NONE),PLEKHG4B(dist=128588)	intergenic	HIVID	Zhao et al.2016	27703150	Tumor
chr12	95624		LOC100288778(dist=4361),FAM138D(dist=52322)	intergenic	HIVID	Zhao et al.2016	27703150	Tumor
chr12	95434		LOC100288778(dist=4171),FAM138D(dist=52512)	intergenic	HIVID	Zhao et al.2016	27703150	Tumor
chr12	94816		LOC100288778(dist=3553),FAM138D(dist=53130)	intergenic	HIVID	Zhao et al.2016	27703150	Tumor
chr12	94693		LOC100288778(dist=3430),FAM138D(dist=53253)	intergenic	HIVID	Zhao et al.2016	27703150	Tumor
chr11	128174857		KIRREL3-AS3(dist=1298904),ETS1(dist=153799)	intergenic	HIVID	Zhao et al.2016	27703150	Tumor
chr3	197900756		FAM157A	intronic	HIVID	Zhao et al.2016	27703150	Tumor
chr15	102520328		DDX11L9;MIR6859-1,MIR6859-2,WASH3P	promoter;downstream	HIVID	Zhao et al.2016	27703150	Tumor
chr9	11021		DDX11L5;WASH1	promoter;downstream	HIVID	Zhao et al.2016	27703150	Tumor
chr1	10115		DDX11L1;MIR6859-1,MIR6859-2,WASH7P	promoter;downstream	HIVID	Zhao et al.2016	27703150	Tumor
chr1	10137		DDX11L1;MIR6859-1,MIR6859-2,WASH7P	promoter;downstream	HIVID	Zhao et al.2016	27703150	Tumor
chr12	133841770		ANHX(dist=29348),NONE(dist=NONE)	intergenic	HIVID	Zhao et al.2016	27703150	Tumor
chr5	1296133		TERT	promoter	HIVID	Zhao et al.2016	27703150	Tumor
chr2	65543447		SPRED2	intronic	HIVID	Zhao et al.2016	27703150	Tumor
chr4	25798564		SEL1L3	intronic	HIVID	Zhao et al.2016	27703150	Tumor
chr4	29847410		MIR4275(dist=1026120),PCDH7(dist=874620)	intergenic	HIVID	Zhao et al.2016	27703150	Tumor
chr2	103715647		TMEM182(dist=281509),LOC100287010(dist=1279661)	intergenic	HIVID	Zhao et al.2016	27703150	Tumor
chr7	115902995		TES(dist=4158),CAV2(dist=236660)	intergenic	HIVID	Zhao et al.2016	27703150	Tumor
chr5	1295914		TERT	promoter	HIVID	Zhao et al.2016	27703150	Tumor
chr21	10834226		TEKT4P2(dist=865633),TPTE(dist=72517)	intergenic	HIVID	Zhao et al.2016	27703150	Tumor
chr21	10834177		TEKT4P2(dist=865584),TPTE(dist=72566)	intergenic	HIVID	Zhao et al.2016	27703150	Tumor
chr7	53341438		POM121L12(dist=236820),FLJ45974(dist=381764)	intergenic	HIVID	Zhao et al.2016	27703150	Tumor
chr7	61739554		NONE(dist=NONE),ZNF733P(dist=1012116)	intergenic	HIVID	Zhao et al.2016	27703150	Tumor
chr7	61739611		NONE(dist=NONE),ZNF733P(dist=1012059)	intergenic	HIVID	Zhao et al.2016	27703150	Tumor
chr7	61739631		NONE(dist=NONE),ZNF733P(dist=1012039)	intergenic	HIVID	Zhao et al.2016	27703150	Tumor
chr20	29829712		MLLT10P1(dist=191574),DEFB115(dist=15755)	intergenic	HIVID	Zhao et al.2016	27703150	Tumor
chr20	29829609		MLLT10P1(dist=191471),DEFB115(dist=15858)	intergenic	HIVID	Zhao et al.2016	27703150	Tumor
chr20	29829488		MLLT10P1(dist=191350),DEFB115(dist=15979)	intergenic	HIVID	Zhao et al.2016	27703150	Tumor
chr2	89867506		MIR4436A(dist=755538),LOC654342(dist=1957203)	intergenic	HIVID	Zhao et al.2016	27703150	Tumor
chr8	43093898		HGSNAT(dist=35928),POTEA(dist=53687)	intergenic	HIVID	Zhao et al.2016	27703150	Tumor
chr8	43093046		HGSNAT(dist=35076),POTEA(dist=54539)	intergenic	HIVID	Zhao et al.2016	27703150	Tumor
chr4	49659575		CWH43(dist=595480),NONE(dist=NONE)	intergenic	HIVID	Zhao et al.2016	27703150	Tumor
chr4	49122762		CWH43(dist=58667),NONE(dist=NONE)	intergenic	HIVID	Zhao et al.2016	27703150	Tumor
chr4	49122713		CWH43(dist=58618),NONE(dist=NONE)	intergenic	HIVID	Zhao et al.2016	27703150	Tumor
chr4	49644651		CWH43(dist=580556),NONE(dist=NONE)	intergenic	HIVID	Zhao et al.2016	27703150	Tumor
chr4	49111748		CWH43(dist=47653),NONE(dist=NONE)	intergenic	HIVID	Zhao et al.2016	27703150	Tumor
chr4	49111649		CWH43(dist=47554),NONE(dist=NONE)	intergenic	HIVID	Zhao et al.2016	27703150	Tumor
chr4	49104567		CWH43(dist=40472),NONE(dist=NONE)	intergenic	HIVID	Zhao et al.2016	27703150	Tumor
chr4	49104468		CWH43(dist=40373),NONE(dist=NONE)	intergenic	HIVID	Zhao et al.2016	27703150	Tumor
chr4	49096991		CWH43(dist=32896),NONE(dist=NONE)	intergenic	HIVID	Zhao et al.2016	27703150	Tumor
chr4	49096961		CWH43(dist=32866),NONE(dist=NONE)	intergenic	HIVID	Zhao et al.2016	27703150	Tumor
chr4	49096881		CWH43(dist=32786),NONE(dist=NONE)	intergenic	HIVID	Zhao et al.2016	27703150	Tumor
chr4	49095366		CWH43(dist=31271),NONE(dist=NONE)	intergenic	HIVID	Zhao et al.2016	27703150	Tumor
chr4	49095252		CWH43(dist=31157),NONE(dist=NONE)	intergenic	HIVID	Zhao et al.2016	27703150	Tumor
chr5	1267042		TERT	intronic	HIVID	Zhao et al.2016	27703150	Tumor
chr5	1267066		TERT	intronic	HIVID	Zhao et al.2016	27703150	Tumor
chr1	57111071		PRKAA2	exonic	HIVID	Zhao et al.2016	27703150	Tumor
chr2	18560017		KCNS3(dist=445792),NT5C1B-RDH14(dist=175972)	intergenic	HIVID	Zhao et al.2016	27703150	Tumor
chr9	137492820		RXRA(dist=160389),COL5A1(dist=40831)	intergenic	HIVID	Zhao et al.2016	27703150	Tumor
chr9	137492775		RXRA(dist=160344),COL5A1(dist=40876)	intergenic	HIVID	Zhao et al.2016	27703150	Tumor
chr11	51588974		OR4C46(dist=72763),NONE(dist=NONE)	intergenic	HIVID	Zhao et al.2016	27703150	Tumor
chr11	51586927		OR4C46(dist=70716),NONE(dist=NONE)	intergenic	HIVID	Zhao et al.2016	27703150	Tumor
chr8	46840116		NONE(dist=NONE),LINC00293(dist=912392)	intergenic	HIVID	Zhao et al.2016	27703150	Tumor
chr14	38693129		SSTR1(dist=10861),CLEC14A(dist=30076)	intergenic	HIVID	Zhao et al.2016	27703150	Tumor
chr18	8885691		SOGA2(dist=52916),NDUFV2(dist=216937)	intergenic	HIVID	Zhao et al.2016	27703150	Tumor
chr2	201427409		SGOL2	intronic	HIVID	Zhao et al.2016	27703150	Tumor
chr9	66758760		PTGER4P2-CDK2AP2P2(dist=255730),RP11-381O7.3(dist=2586	intergenic	HIVID	Zhao et al.2016	27703150	Tumor
chr9	66754816		PTGER4P2-CDK2AP2P2(dist=251786),RP11-381O7.3(dist=2625:	intergenic	HIVID	Zhao et al.2016	27703150	Tumor

Chromosome	Integration site in host genome	Integration site in virus genome	Gene (distance, bp)	Regions	Methods	Author	PMID	Sample
chr9	66739960		PTGER4P2-CDK2AP2P2(dist=236930),RP11-381O7.3(dist=2774	intergenic	HIVID	Zhao et al.2016	27703150	Tumor
chr11	35618144		PAMR1(dist=66296),FJX1(dist=21591)	intergenic	HIVID	Zhao et al.2016	27703150	Tumor
chr11	131305313		NTM	intronic	HIVID	Zhao et al.2016	27703150	Tumor
chr15	20053493		NONE(dist=NONE),CHEK2P2(dist=434504)	intergenic	HIVID	Zhao et al.2016	27703150	Tumor
chr15	20057321		NONE(dist=NONE),CHEK2P2(dist=430676)	intergenic	HIVID	Zhao et al.2016	27703150	Tumor
chr21	14376486		NONE(dist=NONE),ANKRD30BP2(dist=34001)	intergenic	HIVID	Zhao et al.2016	27703150	Tumor
chr3	5559063		MIR4790(dist=267123),AC069277.1(dist=1114982)	intergenic	HIVID	Zhao et al.2016	27703150	Tumor
chr18	15364068		LOC644669(dist=38150),NONE(dist=NONE)	intergenic	HIVID	Zhao et al.2016	27703150	Tumor
chr9	69938947		LOC100133920(dist=273998),FOXD4L5(dist=236760)	intergenic	HIVID	Zhao et al.2016	27703150	Tumor
chr9	69934995		LOC100133920(dist=270046),FOXD4L5(dist=240712)	intergenic	HIVID	Zhao et al.2016	27703150	Tumor
chr9	69920151		LOC100133920(dist=255202),FOXD4L5(dist=255556)	intergenic	HIVID	Zhao et al.2016	27703150	Tumor
chr16	65535282		LINC00922	ncRNA_intronic	HIVID	Zhao et al.2016	27703150	Tumor
chr18	5972976		L3MBTL4	intronic	HIVID	Zhao et al.2016	27703150	Tumor
chr17	28338452		EFCAB5	intronic	HIVID	Zhao et al.2016	27703150	Tumor
chr2	132952869		ANKRD30BL	ncRNA_intronic	HIVID	Zhao et al.2016	27703150	Tumor
chr1	191312055		RP11-463J7.2(dist=541267),RGS18(dist=815537)	intergenic	HIVID	Zhao et al.2016	27703150	Tumor
chr18	78016289		PARD6G(dist=10892),NONE(dist=NONE)	intergenic	HIVID	Zhao et al.2016	27703150	Tumor
chr12	133705731		ZNF891	intronic	HIVID	Zhao et al.2016	27703150	Tumor
chr12	133705706		ZNF891	intronic	HIVID	Zhao et al.2016	27703150	Tumor
chr17	33288856		ZNF830	exonic	HIVID	Zhao et al.2016	27703150	Tumor
chr4	48972		ZNF595,ZNF718	promoter	HIVID	Zhao et al.2016	27703150	Tumor
chr8	123814145		ZHX2	intronic	HIVID	Zhao et al.2016	27703150	Tumor
chr10	81198537		ZCCHC24	intronic	HIVID	Zhao et al.2016	27703150	Tumor
chr17	80582190		WDR45B	intronic	HIVID	Zhao et al.2016	27703150	Tumor
chr17	80581162		WDR45B	intronic	HIVID	Zhao et al.2016	27703150	Tumor
chr18	10217022		VAPA(dist=257004),APCDD1(dist=237603)	intergenic	HIVID	Zhao et al.2016	27703150	Tumor
chr3	196625771		SENP5	intronic	HIVID	Zhao et al.2016	27703150	Tumor
chr10	71928852		SAR1A	intronic	HIVID	Zhao et al.2016	27703150	Tumor
chr16	33876801		RNU6-76P(dist=313558),LINC00273(dist=84251)	intergenic	HIVID	Zhao et al.2016	27703150	Tumor
chr4	1107728		RNF212;TMED11P	promoter;downstream	HIVID	Zhao et al.2016	27703150	Tumor
chr4	1107760		RNF212;TMED11P	promoter;downstream	HIVID	Zhao et al.2016	27703150	Tumor
chr6	46267218		RCAN2	intronic	HIVID	Zhao et al.2016	27703150	Tumor
chr6	108371620		OSTM1	intronic	HIVID	Zhao et al.2016	27703150	Tumor
chr10	42384471		NONE(dist=NONE),LOC441666(dist=442843)	intergenic	HIVID	Zhao et al.2016	27703150	Tumor
chr10	42384544		NONE(dist=NONE),LOC441666(dist=442770)	intergenic	HIVID	Zhao et al.2016	27703150	Tumor
chr10	42387281		NONE(dist=NONE),LOC441666(dist=440033)	intergenic	HIVID	Zhao et al.2016	27703150	Tumor
chr10	42394840		NONE(dist=NONE),LOC441666(dist=432474)	intergenic	HIVID	Zhao et al.2016	27703150	Tumor
chr10	42533167		NONE(dist=NONE),LOC441666(dist=294147)	intergenic	HIVID	Zhao et al.2016	27703150	Tumor
chr10	42596803		NONE(dist=NONE),LOC441666(dist=230511)	intergenic	HIVID	Zhao et al.2016	27703150	Tumor
chr10	42596993		NONE(dist=NONE),LOC441666(dist=230321)	intergenic	HIVID	Zhao et al.2016	27703150	Tumor
chr10	42598161		NONE(dist=NONE),LOC441666(dist=229153)	intergenic	HIVID	Zhao et al.2016	27703150	Tumor
chr2	36034033		NONE(dist=NONE),LOC100288911(dist=547859)	intergenic	HIVID	Zhao et al.2016	27703150	Tumor
chr19	27733876		NONE(dist=NONE),LINC00662(dist=547525)	intergenic	HIVID	Zhao et al.2016	27703150	Tumor
chr19	56407216		NLRP4(dist=13996),NLRP13(dist=95)	intergenic	HIVID	Zhao et al.2016	27703150	Tumor
chr11	69227218		MYEOV(dist=162464),CCND1(dist=228655)	intergenic	HIVID	Zhao et al.2016	27703150	Tumor
chr11	69226562		MYEOV(dist=161808),CCND1(dist=229311)	intergenic	HIVID	Zhao et al.2016	27703150	Tumor
chr8	97121652		LOC100500773(dist=161076),GDF6(dist=32906)	intergenic	HIVID	Zhao et al.2016	27703150	Tumor
chr8	91418062		LINC00534(dist=17875),LINC01030(dist=186941)	intergenic	HIVID	Zhao et al.2016	27703150	Tumor
chr8	91417988		LINC00534(dist=17801),LINC01030(dist=187015)	intergenic	HIVID	Zhao et al.2016	27703150	Tumor
chr1	75650434		LHX8(dist=23216),SLC44A5(dist=17382)	intergenic	HIVID	Zhao et al.2016	27703150	Tumor
chr2	239074021		KLHL30(dist=12474),ILKAP(dist=5022)	intergenic	HIVID	Zhao et al.2016	27703150	Tumor
chr1	201182487		IGFN1	exonic	HIVID	Zhao et al.2016	27703150	Tumor
chr11	69622868		FGF4(dist=32697),FGF3(dist=1868)	intergenic	HIVID	Zhao et al.2016	27703150	Tumor
chr11	69532347		FGF19(dist=13241),FGF4(dist=55450)	intergenic	HIVID	Zhao et al.2016	27703150	Tumor
chr11	69532314		FGF19(dist=13208),FGF4(dist=55483)	intergenic	HIVID	Zhao et al.2016	27703150	Tumor
chr11	69532308		FGF19(dist=13202),FGF4(dist=55489)	intergenic	HIVID	Zhao et al.2016	27703150	Tumor
chr11	69532282		FGF19(dist=13176),FGF4(dist=55515)	intergenic	HIVID	Zhao et al.2016	27703150	Tumor
chr11	69532213		FGF19(dist=13107),FGF4(dist=55584)	intergenic	HIVID	Zhao et al.2016	27703150	Tumor
chr2	15084433		FAM84A(dist=304265),NBAS(dist=222599)	intergenic	HIVID	Zhao et al.2016	27703150	Tumor
chr2	15084394		FAM84A(dist=304226),NBAS(dist=222638)	intergenic	HIVID	Zhao et al.2016	27703150	Tumor
chr1	118313518		FAM46C(dist=142507),GDAP2(dist=92589)	intergenic	HIVID	Zhao et al.2016	27703150	Tumor
chr3	170677212		EIF5A2(dist=50786),SLC2A2(dist=36925)	intergenic	HIVID	Zhao et al.2016	27703150	Tumor
chr5	158350034		EBF1	intronic	HIVID	Zhao et al.2016	27703150	Tumor
chr2	218465364		DIRC3	ncRNA_exonic	HIVID	Zhao et al.2016	27703150	Tumor
chr10	127555687		DHX32	exonic	HIVID	Zhao et al.2016	27703150	Tumor
chr1	69127268		DEPDC1(dist=164364),LRRC7(dist=1098590)	intergenic	HIVID	Zhao et al.2016	27703150	Tumor
chr4	49137653		CWH43(dist=73558),NONE(dist=NONE)	intergenic	HIVID	Zhao et al.2016	27703150	Tumor
chr11	99840254		CNTN5	intronic	HIVID	Zhao et al.2016	27703150	Tumor
chr12	105399597		C12orf45(dist=11092),ALDH1L2(dist=13965)	intergenic	HIVID	Zhao et al.2016	27703150	Tumor
chr13	70805076		ATXN8OS(dist=91191),LINC00348(dist=784197)	intergenic	HIVID	Zhao et al.2016	27703150	Tumor
chr7	105395562		ATXN7L1	intronic	HIVID	Zhao et al.2016	27703150	Tumor
chr5	148989121		ARHGEF37	exonic	HIVID	Zhao et al.2016	27703150	Tumor
chr12	34845814		ALG10(dist=664578),NONE(dist=NONE)	intergenic	HIVID	Zhao et al.2016	27703150	Tumor
chr12	34835930		ALG10(dist=654694),NONE(dist=NONE)	intergenic	HIVID	Zhao et al.2016	27703150	Tumor
chr6	135611443		AHI1	intronic	HIVID	Zhao et al.2016	27703150	Tumor
chr16	4171537		ADCY9	promoter	HIVID	Zhao et al.2016	27703150	Tumor
chr16	77627318		ADAMTS18(dist=158307),NUDT7(dist=129071)	intergenic	HIVID	Zhao et al.2016	27703150	Tumor
chr13	62573493		PCDH20(dist=583838),LINC00358(dist=4165)	intergenic	HIVID	Zhao et al.2016	27703150	Tumor
chr4	16622379		MIR548AX	ncRNA_intronic	HIVID	Zhao et al.2016	27703150	Tumor
chr5	1295090		TERT	exonic	HIVID	Zhao et al.2016	27703150	Tumor
chr5	1295128		TERT	UTR5	HIVID	Zhao et al.2016	27703150	Tumor
chr18	10288172		VAPA(dist=328154),APCDD1(dist=166453)	intergenic	HIVID	Zhao et al.2016	27703150	Tumor
chr8	60832269		TOX(dist=800502),CA8(dist=269154)	intergenic	HIVID	Zhao et al.2016	27703150	Tumor
chr17	26643906		KRT18P55,TMEM97	promoter	HIVID	Zhao et al.2016	27703150	Tumor
chr17	26643883		KRT18P55,TMEM97	promoter	HIVID	Zhao et al.2016	27703150	Tumor
chr8	12902177		KIAA1456(dist=14893),DLC1(dist=38695)	intergenic	HIVID	Zhao et al.2016	27703150	Tumor
chr8	12902156		KIAA1456(dist=14872),DLC1(dist=38716)	intergenic	HIVID	Zhao et al.2016	27703150	Tumor
chr8	7277561		DEFB4B;DEFB103A,DEFB103B	promoter;downstream	HIVID	Zhao et al.2016	27703150	Tumor
chr8	7749027		DEFB4A;DEFB103A,DEFB103B	promoter;downstream	HIVID	Zhao et al.2016	27703150	Tumor
chr10	113165805		ADRA2A(dist=325143),GPAM(dist=743817)	intergenic	HIVID	Zhao et al.2016	27703150	Tumor
chr5	1295128		TERT	UTR5	HIVID	Zhao et al.2016	27703150	Tumor
chr5	1295107		TERT	UTR5	HIVID	Zhao et al.2016	27703150	Tumor
chr5	1295078		TERT	exonic	HIVID	Zhao et al.2016	27703150	Tumor
chr5	1295165		TERT	promoter	HIVID	Zhao et al.2016	27703150	Tumor

Chromosome	Integration site in host genome	Integration site in virus genome	Gene (distance, bp)	Regions	Methods	Author	PMID	Sample
chr7	139546409		TBXAS1	intronic	HIVID	Zhao et al.2016	27703150	Tumor
chr7	139724359		PARP12	UTR3	HIVID	Zhao et al.2016	27703150	Tumor
chr17	22260359		MTRNR2L1(dist=236368),NONE(dist=NONE)	intergenic	HIVID	Zhao et al.2016	27703150	Tumor
chr17	22257980		MTRNR2L1(dist=233989),NONE(dist=NONE)	intergenic	HIVID	Zhao et al.2016	27703150	Tumor
chr17	22253223		MTRNR2L1(dist=229232),NONE(dist=NONE)	intergenic	HIVID	Zhao et al.2016	27703150	Tumor
chr17	22246089		MTRNR2L1(dist=222098),NONE(dist=NONE)	intergenic	HIVID	Zhao et al.2016	27703150	Tumor
chr9	24691633		IZUMO3(dist=145959),TUSC1(dist=984754)	intergenic	HIVID	Zhao et al.2016	27703150	Tumor
chr9	24690697		IZUMO3(dist=145023),TUSC1(dist=985690)	intergenic	HIVID	Zhao et al.2016	27703150	Tumor
chr6	1842577		GMDS	intronic	HIVID	Zhao et al.2016	27703150	Tumor
chr8	127963517		FAM84B(dist=392806),PCAT1(dist=61882)	intergenic	HIVID	Zhao et al.2016	27703150	Tumor
chr8	127963441		FAM84B(dist=392730),PCAT1(dist=61958)	intergenic	HIVID	Zhao et al.2016	27703150	Tumor
chr6	334185		DUSP22	intronic	HIVID	Zhao et al.2016	27703150	Tumor
chr6	340143		DUSP22	intronic	HIVID	Zhao et al.2016	27703150	Tumor
chr3	74630180		CNTN3(dist=59837),MIR4444-1(dist=633447)	intergenic	HIVID	Zhao et al.2016	27703150	Tumor
chr3	74630146		CNTN3(dist=59803),MIR4444-1(dist=633481)	intergenic	HIVID	Zhao et al.2016	27703150	Tumor
chr19	49464519		BAX	splicing	HIVID	Zhao et al.2016	27703150	Tumor
chr19	49464520		BAX	intronic	HIVID	Zhao et al.2016	27703150	Tumor
chr17	33478240		UNC45B	intronic	HIVID	Zhao et al.2016	27703150	Tumor
chr8	15438807		TUSC3	intronic	HIVID	Zhao et al.2016	27703150	Tumor
chr1	48616839		SKINTL	ncRNA_intronic	HIVID	Zhao et al.2016	27703150	Tumor
chr2	53014149		NRXN1(dist=1754475),ASB3(dist=882968)	intergenic	HIVID	Zhao et al.2016	27703150	Tumor
chr8	16528668		MSR1(dist=478368),FGF20(dist=321666)	intergenic	HIVID	Zhao et al.2016	27703150	Tumor
chr4	63022755		LPHN3(dist=84587),NONE(dist=NONE)	intergenic	HIVID	Zhao et al.2016	27703150	Tumor
chr8	36956460		KCNU1(dist=162817),ZNF703(dist=596841)	intergenic	HIVID	Zhao et al.2016	27703150	Tumor
chr7	158924325		VIPR2	intronic	HIVID	Zhao et al.2016	27703150	Tumor
chr17	22174574		MTRNR2L1(dist=150583),NONE(dist=NONE)	intergenic	HIVID	Zhao et al.2016	27703150	Tumor
chr7	72803658		FKBP6(dist=31012),FZD9(dist=44451)	intergenic	HIVID	Zhao et al.2016	27703150	Tumor
chr9	874889		DMRT1	intronic	HIVID	Zhao et al.2016	27703150	Tumor
chr3	1169427		CNTN6	intronic	HIVID	Zhao et al.2016	27703150	Tumor
chr3	1169398		CNTN6	intronic	HIVID	Zhao et al.2016	27703150	Tumor
chr20	34546559		CNBD2,SCAND1;PHF20	promoter;downstream	HIVID	Zhao et al.2016	27703150	Tumor
chr8	108860545		ANGPT1(dist=350291),RSPO2(dist=50999)	intergenic	HIVID	Zhao et al.2016	27703150	Tumor
chr16	30714792		SRCAP	intronic	HIVID	Zhao et al.2016	27703150	Tumor
chr8	82943497		SNX16(dist=188976),NONE(dist=NONE)	intergenic	HIVID	Zhao et al.2016	27703150	Tumor
chr8	82943418		SNX16(dist=188897),NONE(dist=NONE)	intergenic	HIVID	Zhao et al.2016	27703150	Tumor
chr11	69035539		RP11-554A11.8(dist=96509),MYEOV(dist=26083)	intergenic	HIVID	Zhao et al.2016	27703150	Tumor
chr16	33894108		RNU6-76P(dist=330865),LINC00273(dist=66944)	intergenic	HIVID	Zhao et al.2016	27703150	Tumor
chr11	70203275		PPFIA1	intronic	HIVID	Zhao et al.2016	27703150	Tumor
chr11	70201816		PPFIA1	exonic	HIVID	Zhao et al.2016	27703150	Tumor
chr11	70201794		PPFIA1	exonic	HIVID	Zhao et al.2016	27703150	Tumor
chr3	87969099		POU1F1(dist=643362),HTR1F(dist=62627)	intergenic	HIVID	Zhao et al.2016	27703150	Tumor
chr14	20023768		POTEM	promoter	HIVID	Zhao et al.2016	27703150	Tumor
chr22	16291473		POTEH	promoter	HIVID	Zhao et al.2016	27703150	Tumor
chr14	19549860		POTEG	promoter	HIVID	Zhao et al.2016	27703150	Tumor
chr11	73382226		PLEKHB1(dist=8362),RAB6A(dist=4457)	intergenic	HIVID	Zhao et al.2016	27703150	Tumor
chr11	73381001		PLEKHB1(dist=7137),RAB6A(dist=5682)	intergenic	HIVID	Zhao et al.2016	27703150	Tumor
chr11	73380920		PLEKHB1(dist=7056),RAB6A(dist=5763)	intergenic	HIVID	Zhao et al.2016	27703150	Tumor
chr11	69483663		ORAOV1	intronic	HIVID	Zhao et al.2016	27703150	Tumor
chr10	42398594		NONE(dist=NONE),LOC441666(dist=428720)	intergenic	HIVID	Zhao et al.2016	27703150	Tumor
chr10	42398684		NONE(dist=NONE),LOC441666(dist=428630)	intergenic	HIVID	Zhao et al.2016	27703150	Tumor
chr19	27732757		NONE(dist=NONE),LINC00662(dist=548644)	intergenic	HIVID	Zhao et al.2016	27703150	Tumor
chr19	27735282		NONE(dist=NONE),LINC00662(dist=546119)	intergenic	HIVID	Zhao et al.2016	27703150	Tumor
chr19	27738691		NONE(dist=NONE),LINC00662(dist=542710)	intergenic	HIVID	Zhao et al.2016	27703150	Tumor
chr16	46442795		NONE(dist=NONE),ANKRD26P1(dist=60454)	intergenic	HIVID	Zhao et al.2016	27703150	Tumor
chr16	46442851		NONE(dist=NONE),ANKRD26P1(dist=60398)	intergenic	HIVID	Zhao et al.2016	27703150	Tumor
chr2	89849667		MIR4436A(dist=737699),LOC654342(dist=1975042)	intergenic	HIVID	Zhao et al.2016	27703150	Tumor
chr2	89849613		MIR4436A(dist=737645),LOC654342(dist=1975096)	intergenic	HIVID	Zhao et al.2016	27703150	Tumor
chr18	1610065		LINC00470(dist=250435),METTL4(dist=927459)	intergenic	HIVID	Zhao et al.2016	27703150	Tumor
chr18	1609831		LINC00470(dist=250201),METTL4(dist=927693)	intergenic	HIVID	Zhao et al.2016	27703150	Tumor
chr11	69681597		FGF3(dist=47405),ANO1-AS2(dist=236943)	intergenic	HIVID	Zhao et al.2016	27703150	Tumor
chr11	69586808		FGF19(dist=67702),FGF4(dist=989)	intergenic	HIVID	Zhao et al.2016	27703150	Tumor
chr16	30689298		FBRS(dist=7167),LOC730183(dist=19727)	intergenic	HIVID	Zhao et al.2016	27703150	Tumor
chr1	121485373		EMBP1(dist=171687),NONE(dist=NONE)	intergenic	HIVID	Zhao et al.2016	27703150	Tumor
chr16	22241621		EEF2K	intronic	HIVID	Zhao et al.2016	27703150	Tumor
chr15	55802300		DYX1C1	promoter	HIVID	Zhao et al.2016	27703150	Tumor
chr11	71571421		DEFB108B(dist=22813),LOC100133315(dist=5134)	intergenic	HIVID	Zhao et al.2016	27703150	Tumor
chr5	1295706		TERT	promoter	HIVID	Zhao et al.2016	27703150	Tumor
chr9	11214540		PTPRD(dist=601817),TYRP1(dist=1478846)	intergenic	HIVID	Zhao et al.2016	27703150	Tumor
chr5	30893993		LOC101929681(dist=1497910),CDH6(dist=299769)	intergenic	HIVID	Zhao et al.2016	27703150	Tumor
chr5	30893080		LOC101929681(dist=1496997),CDH6(dist=300682)	intergenic	HIVID	Zhao et al.2016	27703150	Tumor
chr8	59288051		LOC101929528(dist=103391),UBXN2B(dist=35772)	intergenic	HIVID	Zhao et al.2016	27703150	Tumor
chr14	97405605		LINC00618	promoter	HIVID	Zhao et al.2016	27703150	Tumor
chr4	186120571		KIAA1430	intronic	HIVID	Zhao et al.2016	27703150	Tumor
chr18	39461035		KC6(dist=360474),PIK3C3(dist=74164)	intergenic	HIVID	Zhao et al.2016	27703150	Tumor
chr4	185377075		IRF2	intronic	HIVID	Zhao et al.2016	27703150	Tumor
chr7	118723463		ANKRD7(dist=840679),KCND2(dist=1190259)	intergenic	HIVID	Zhao et al.2016	27703150	Tumor
chr4	133218890		NONE(dist=NONE),PCDH10(dist=851580)	intergenic	HIVID	Zhao et al.2016	27703150	Tumor
chr4	65710263		TECRL(dist=435085),RP11-707A18.1(dist=69736)	intergenic	HIVID	Zhao et al.2016	27703150	Tumor
chr4	65784258		RP11-707A18.1	ncRNA_intronic	HIVID	Zhao et al.2016	27703150	Tumor
chr10	42400597		NONE(dist=NONE),LOC441666(dist=426717)	intergenic	HIVID	Zhao et al.2016	27703150	Tumor
chr10	42529883		NONE(dist=NONE),LOC441666(dist=297431)	intergenic	HIVID	Zhao et al.2016	27703150	Tumor
chr10	42532596		NONE(dist=NONE),LOC441666(dist=294718)	intergenic	HIVID	Zhao et al.2016	27703150	Tumor
chr5	1296487		TERT	promoter	HIVID	Zhao et al.2016	27703150	Tumor
chr1	27788923		WASF2	intronic	HIVID	Zhao et al.2016	27703150	Tumor
chr1	28028949		IFI6(dist=30225),FAM76A(dist=23541)	intergenic	HIVID	Zhao et al.2016	27703150	Tumor
chr5	1295750		TERT	promoter	HIVID	Zhao et al.2016	27703150	Tumor
chr5	1295780		TERT	promoter	HIVID	Zhao et al.2016	27703150	Tumor
chr8	57275943		SDR16C5(dist=42702),SDR16C6P(dist=11334)	intergenic	HIVID	Zhao et al.2016	27703150	Tumor
chr17	12612108		MYOCD	intronic	HIVID	Zhao et al.2016	27703150	Tumor
chr14	63214814		KCNH5	intronic	HIVID	Zhao et al.2016	27703150	Tumor
chr1	100188402		FRRS1	intronic	HIVID	Zhao et al.2016	27703150	Tumor
chr18	2856318		EMILIN2	intronic	HIVID	Zhao et al.2016	27703150	Tumor
chr1	61401139		C1orf87(dist=861697),NFIA(dist=141807)	intergenic	HIVID	Zhao et al.2016	27703150	Tumor
chr2	237143672		ASB18	intronic	HIVID	Zhao et al.2016	27703150	Tumor

Chromosome	Integration site in host genome	Integration site in virus genome	Gene (distance, bp)	Regions	Methods	Author	PMID	Sample
chr10	45774327		ANKRD30BP3(dist=92838),OR13A1(dist=23775)	intergenic	HIVID	Zhao et al.2016	27703150	Tumor
chr13	29128519		FLT1(dist=59254),POMP(dist=104622)	intergenic	HIVID	Zhao et al.2016	27703150	Tumor
chr9	43041926		FAM95B1	promoter	HIVID	Zhao et al.2016	27703150	Tumor
chr9	42459977		FAM95B1	promoter	HIVID	Zhao et al.2016	27703150	Tumor
chr3	184317497		EPHB3(dist=17301),MAGEF1(dist=110658)	intergenic	HIVID	Zhao et al.2016	27703150	Tumor
chr9	69467407		ANKRD20A4(dist=42298),LOC100133920(dist=183954)	intergenic	HIVID	Zhao et al.2016	27703150	Tumor
chr19	36213620		KMT2B;KMT2B	exonic;splicing	HIVID	Zhao et al.2016	27703150	Tumor
chr7	106917740		COG5	intronic	HIVID	Zhao et al.2016	27703150	Tumor
chr5	1295721		TERT	promoter	HIVID	Zhao et al.2016	27703150	Tumor
chr5	1295582		TERT	promoter	HIVID	Zhao et al.2016	27703150	Tumor
chr8	103864871		AZIN1	intronic	HIVID	Zhao et al.2016	27703150	Tumor
chr16	87558560		ZCCHC14(dist=33100),JPH3(dist=77933)	intergenic	HIVID	Zhao et al.2016	27703150	Tumor
chr2	98856030		VWA3B	intronic	HIVID	Zhao et al.2016	27703150	Tumor
chr3	17607571		TBC1D5	intronic	HIVID	Zhao et al.2016	27703150	Tumor
chr3	47714913		SMARCC1	intronic	HIVID	Zhao et al.2016	27703150	Tumor
chr3	47714823		SMARCC1	intronic	HIVID	Zhao et al.2016	27703150	Tumor
chr21	48119872		PRMT2(dist=34836),NONE(dist=NONE)	intergenic	HIVID	Zhao et al.2016	27703150	Tumor
chr2	194773377		PCGEM1(dist=1131752),AC018799.1(dist=435616)	intergenic	HIVID	Zhao et al.2016	27703150	Tumor
chr13	64365167		OR7E156P(dist=48466),NONE(dist=NONE)	intergenic	HIVID	Zhao et al.2016	27703150	Tumor
chr5	10423		NONE(dist=NONE),PLEKHG4B(dist=129950)	intergenic	HIVID	Zhao et al.2016	27703150	Tumor
chr5	10453		NONE(dist=NONE),PLEKHG4B(dist=129920)	intergenic	HIVID	Zhao et al.2016	27703150	Tumor
chr5	11483		NONE(dist=NONE),PLEKHG4B(dist=128890)	intergenic	HIVID	Zhao et al.2016	27703150	Tumor
chr5	11521		NONE(dist=NONE),PLEKHG4B(dist=128852)	intergenic	HIVID	Zhao et al.2016	27703150	Tumor
chr5	11576		NONE(dist=NONE),PLEKHG4B(dist=128797)	intergenic	HIVID	Zhao et al.2016	27703150	Tumor
chr2	44171391		LRPPRC	intronic	HIVID	Zhao et al.2016	27703150	Tumor
chr2	44171315		LRPPRC	intronic	HIVID	Zhao et al.2016	27703150	Tumor
chr3	188473658		LPP	intronic	HIVID	Zhao et al.2016	27703150	Tumor
chr12	95539		LOC100288778(dist=4276),FAM138D(dist=52407)	intergenic	HIVID	Zhao et al.2016	27703150	Tumor
chr12	95503		LOC100288778(dist=4240),FAM138D(dist=52443)	intergenic	HIVID	Zhao et al.2016	27703150	Tumor
chr12	95485		LOC100288778(dist=4222),FAM138D(dist=52461)	intergenic	HIVID	Zhao et al.2016	27703150	Tumor
chr2	97268548		KANSL3	exonic	HIVID	Zhao et al.2016	27703150	Tumor
chr12	67426565		GRIP1(dist=353640),CAND1(dist=236496)	intergenic	HIVID	Zhao et al.2016	27703150	Tumor
chr1	59841513		FGGY	intronic	HIVID	Zhao et al.2016	27703150	Tumor
chr1	59841552		FGGY	intronic	HIVID	Zhao et al.2016	27703150	Tumor
chr3	197900735		FAM157A	intronic	HIVID	Zhao et al.2016	27703150	Tumor
chr3	197900821		FAM157A	intronic	HIVID	Zhao et al.2016	27703150	Tumor
chr3	197900342		FAM157A	intronic	HIVID	Zhao et al.2016	27703150	Tumor
chr10	135524694		DUX4L7(dist=26236),NONE(dist=NONE)	intergenic	HIVID	Zhao et al.2016	27703150	Tumor
chr1	10003		DDX11L1;MIR6859-1,MIR6859-2,WASH7P	promoter;downstream	HIVID	Zhao et al.2016	27703150	Tumor
chr3	145685168		C3orf58(dist=1973958),PLOD2(dist=102060)	intergenic	HIVID	Zhao et al.2016	27703150	Tumor
chr3	145684772		C3orf58(dist=1973562),PLOD2(dist=102456)	intergenic	HIVID	Zhao et al.2016	27703150	Tumor
chr6	157237351		ARID1B	intronic	HIVID	Zhao et al.2016	27703150	Tumor
chr12	133841653		ANHX(dist=29231),NONE(dist=NONE)	intergenic	HIVID	Zhao et al.2016	27703150	Tumor
chr3	115076141		ZBTB20(dist=210014),GAP43(dist=266010)	intergenic	HIVID	Zhao et al.2016	27703150	Tumor
chr3	115076053		ZBTB20(dist=209926),GAP43(dist=266098)	intergenic	HIVID	Zhao et al.2016	27703150	Tumor
chr2	98856030		VWA3B	intronic	HIVID	Zhao et al.2016	27703150	Tumor
chr5	1295392		TERT	promoter	HIVID	Zhao et al.2016	27703150	Tumor
chr5	145876461		TCERG1	intronic	HIVID	Zhao et al.2016	27703150	Tumor
chr11	13158769		RASSF10(dist=125116),ARNTL(dist=140556)	intergenic	HIVID	Zhao et al.2016	27703150	Tumor
chr1	36901722		OSCP1	intronic	HIVID	Zhao et al.2016	27703150	Tumor
chr11	68791582		MRGPRF(dist=10732),TPCN2(dist=24768)	intergenic	HIVID	Zhao et al.2016	27703150	Tumor
chr14	50207046		KLHDC1	intronic	HIVID	Zhao et al.2016	27703150	Tumor
chr2	97268554		KANSL3	exonic	HIVID	Zhao et al.2016	27703150	Tumor
chr2	97274973		KANSL3	intronic	HIVID	Zhao et al.2016	27703150	Tumor
chr19	3567642		HMG20B	promoter	HIVID	Zhao et al.2016	27703150	Tumor
chr9	14900981		FREM1	intronic	HIVID	Zhao et al.2016	27703150	Tumor
chr2	96833076		DUSP2(dist=21897),STARD7(dist=17527)	intergenic	HIVID	Zhao et al.2016	27703150	Tumor
chr2	96833065		DUSP2(dist=21886),STARD7(dist=17538)	intergenic	HIVID	Zhao et al.2016	27703150	Tumor
chr2	96833011		DUSP2(dist=21832),STARD7(dist=17592)	intergenic	HIVID	Zhao et al.2016	27703150	Tumor
chr17	20288995		CCDC144CP	ncRNA_intronic	HIVID	Zhao et al.2016	27703150	Tumor
chr17	18463373		CCDC144B	ncRNA_intronic	HIVID	Zhao et al.2016	27703150	Tumor
chr12	107758083		BTBD11	intronic	HIVID	Zhao et al.2016	27703150	Tumor
chr2	133022658		ANKRD30BL,MIR663B	promoter	HIVID	Zhao et al.2016	27703150	Tumor
chr6	43392921		ABCC10,MIR6780B	promoter	HIVID	Zhao et al.2016	27703150	Tumor
chr5	1296194		TERT	promoter	HIVID	Zhao et al.2016	27703150	Tumor
chr5	1295116		TERT	UTR5	HIVID	Zhao et al.2016	27703150	Tumor
chr16	13130312		SHISA9	intronic	HIVID	Zhao et al.2016	27703150	Tumor
chr8	68900931		PREX2	intronic	HIVID	Zhao et al.2016	27703150	Tumor
chr10	42391024		NONE(dist=NONE),LOC441666(dist=436290)	intergenic	HIVID	Zhao et al.2016	27703150	Tumor
chr10	42396098		NONE(dist=NONE),LOC441666(dist=431216)	intergenic	HIVID	Zhao et al.2016	27703150	Tumor
chr9	30421552		LOC401497(dist=13100),ACO1(dist=1963049)	intergenic	HIVID	Zhao et al.2016	27703150	Tumor
chr9	30420582		LOC401497(dist=12130),ACO1(dist=1964019)	intergenic	HIVID	Zhao et al.2016	27703150	Tumor
chr15	22065820		LOC339010,POTEB,POTEB2	intronic	HIVID	Zhao et al.2016	27703150	Tumor
chr3	48781358		IP6K2(dist=26647),PRKAR2A(dist=6735)	intergenic	HIVID	Zhao et al.2016	27703150	Tumor
chr3	197201628		DLG1-AS1(dist=171007),BDH1(dist=35026)	intergenic	HIVID	Zhao et al.2016	27703150	Tumor
chr5	1297123		TERT	promoter	HIVID	Zhao et al.2016	27703150	Tumor
chr5	1297584		TERT	promoter	HIVID	Zhao et al.2016	27703150	Tumor
chr5	1297089		TERT	promoter	HIVID	Zhao et al.2016	27703150	Tumor
chr14	35113453		SNX6(dist=14087),CFL2(dist=66135)	intergenic	HIVID	Zhao et al.2016	27703150	Tumor
chr22	36140961		RBFOX2	intronic	HIVID	Zhao et al.2016	27703150	Tumor
chr22	36156432		RBFOX2	intronic	HIVID	Zhao et al.2016	27703150	Tumor
chr13	62198455		PCDH20(dist=208800),LINC00358(dist=379203)	intergenic	HIVID	Zhao et al.2016	27703150	Tumor
chr4	10045		NONE(dist=NONE),ZNF595(dist=43134)	intergenic	HIVID	Zhao et al.2016	27703150	Tumor
chr12	65690497		MSRB3	intronic	HIVID	Zhao et al.2016	27703150	Tumor
chr8	56949666		LYN(dist=24660),RPS20(dist=31073)	intergenic	HIVID	Zhao et al.2016	27703150	Tumor
chr5	29934240		LOC101929681(dist=538157),CDH6(dist=1259522)	intergenic	HIVID	Zhao et al.2016	27703150	Tumor
chr6	77878289		IMPG1(dist=1095894),HTR1B(dist=293659)	intergenic	HIVID	Zhao et al.2016	27703150	Tumor
chr1	10248		DDX11L1;MIR6859-1,MIR6859-2,WASH7P	promoter;downstream	HIVID	Zhao et al.2016	27703150	Tumor
chr1	10376		DDX11L1;MIR6859-1,MIR6859-2,WASH7P	promoter;downstream	HIVID	Zhao et al.2016	27703150	Tumor
chr1	34690133		C1orf94(dist=5402),GJB5(dist=530515)	intergenic	HIVID	Zhao et al.2016	27703150	Tumor
chr8	108859321		ANGPT1(dist=349067),RSPO2(dist=52223)	intergenic	HIVID	Zhao et al.2016	27703150	Tumor
chr5	1276360		TERT	intronic	HIVID	Zhao et al.2016	27703150	Tumor
chr10	84316519		NRG3	intronic	HIVID	Zhao et al.2016	27703150	Tumor
chr8	46849322		NONE(dist=NONE),LINC00293(dist=903186)	intergenic	HIVID	Zhao et al.2016	27703150	Tumor

Chromosome	Integration site in host genome	Integration site in virus genome	Gene (distance, bp)	Regions	Methods	Author	PMID	Sample
chr8	37290809		KCNU1(dist=497166),ZNF703(dist=262492)	intergenic	HIVID	Zhao et al.2016	27703150	Tumor
chr1	121485120		EMBP1(dist=171434),NONE(dist=NONE)	intergenic	HIVID	Zhao et al.2016	27703150	Tumor
chr1	121484101		EMBP1(dist=170415),NONE(dist=NONE)	intergenic	HIVID	Zhao et al.2016	27703150	Tumor
chr12	34813113		ALG10(dist=631877),NONE(dist=NONE)	intergenic	HIVID	Zhao et al.2016	27703150	Tumor
chr12	34804474		ALG10(dist=623238),NONE(dist=NONE)	intergenic	HIVID	Zhao et al.2016	27703150	Tumor
chr5	1295129		TERT	UTR5	HIVID	Zhao et al.2016	27703150	Tumor
chr12	118949047		SUDS3(dist=93207),SRRM4(dist=470253)	intergenic	HIVID	Zhao et al.2016	27703150	Tumor
chr16	69799		MIR6859-1,MIR6859-2;DDX11L10	promoter;downstream	HIVID	Zhao et al.2016	27703150	Tumor
chr16	69835		MIR6859-1,MIR6859-2;DDX11L10	promoter;downstream	HIVID	Zhao et al.2016	27703150	Tumor
chr16	69751		MIR6859-1,MIR6859-2;DDX11L10	promoter;downstream	HIVID	Zhao et al.2016	27703150	Tumor
chr1	220021773		LYPLAL1(dist=635566),RNU5F-1(dist=24846)	intergenic	HIVID	Zhao et al.2016	27703150	Tumor
chr1	220021748		LYPLAL1(dist=635541),RNU5F-1(dist=24871)	intergenic	HIVID	Zhao et al.2016	27703150	Tumor
chr8	38245770		LETM2	intronic	HIVID	Zhao et al.2016	27703150	Tumor
chr4	6107760		JAKMIP1	intronic	HIVID	Zhao et al.2016	27703150	Tumor
chr2	205321430		ICOS(dist=495132),PARD3B(dist=89086)	intergenic	HIVID	Zhao et al.2016	27703150	Tumor
chr4	191043807		DUX4(dist=30365),NONE(dist=NONE)	intergenic	HIVID	Zhao et al.2016	27703150	Tumor
chr22	24533599		CABIN1	intronic	HIVID	Zhao et al.2016	27703150	Tumor
chr22	24532875		CABIN1	intronic	HIVID	Zhao et al.2016	27703150	Tumor
chr4	178408072		AGA(dist=44415),LINC01098(dist=241839)	intergenic	HIVID	Zhao et al.2016	27703150	Tumor
chr8	589721		TDRP(dist=93940),ERICH1(dist=24479)	intergenic	HIVID	Zhao et al.2016	27703150	Tumor
chr12	131645958		RP11-638F5.1	promoter	HIVID	Zhao et al.2016	27703150	Tumor
chr11	38787991		NONE(dist=NONE),LRRC4C(dist=1347760)	intergenic	HIVID	Zhao et al.2016	27703150	Tumor
chr12	66451372		MIR6074(dist=33866),LLPH(dist=65477)	intergenic	HIVID	Zhao et al.2016	27703150	Tumor
chr10	6052612		IL15RA(dist=32462),IL2RA(dist=45)	intergenic	HIVID	Zhao et al.2016	27703150	Tumor
chr5	52777374		FST	intronic	HIVID	Zhao et al.2016	27703150	Tumor
chr19	11009663		CARM1	intronic	HIVID	Zhao et al.2016	27703150	Tumor
chr8	39588318		ADAM18(dist=735),ADAM2(dist=12937)	intergenic	HIVID	Zhao et al.2016	27703150	Tumor
chr5	1295881		TERT	promoter	HIVID	Zhao et al.2016	27703150	Tumor
chr3	139485273		NMNAT3(dist=88388),CLSTN2(dist=168754)	intergenic	HIVID	Zhao et al.2016	27703150	Tumor
chr5	1295420		TERT	promoter	HIVID	Zhao et al.2016	27703150	Tumor
chr4	68266721		RP11-807H7.1(dist=1707617),CENPC(dist=71268)	intergenic	HIVID	Zhao et al.2016	27703150	Tumor
chr4	68266633		RP11-807H7.1(dist=1707529),CENPC(dist=71356)	intergenic	HIVID	Zhao et al.2016	27703150	Tumor
chr12	132411090		PUS1;ULK1	promoter;downstream	HIVID	Zhao et al.2016	27703150	Tumor
chr5	10442		NONE(dist=NONE),PLEKHG4B(dist=129931)	intergenic	HIVID	Zhao et al.2016	27703150	Tumor
chr6	61914260		NONE(dist=NONE),MTRNR2L9(dist=369748)	intergenic	HIVID	Zhao et al.2016	27703150	Tumor
chr6	61914349		NONE(dist=NONE),MTRNR2L9(dist=369659)	intergenic	HIVID	Zhao et al.2016	27703150	Tumor
chr19	27732034		NONE(dist=NONE),LINC00662(dist=549367)	intergenic	HIVID	Zhao et al.2016	27703150	Tumor
chr12	95550		LOC100288778(dist=4287),FAM138D(dist=52396)	intergenic	HIVID	Zhao et al.2016	27703150	Tumor
chr12	95546		LOC100288778(dist=4283),FAM138D(dist=52400)	intergenic	HIVID	Zhao et al.2016	27703150	Tumor
chr12	95484		LOC100288778(dist=4221),FAM138D(dist=52462)	intergenic	HIVID	Zhao et al.2016	27703150	Tumor
chr3	197900823		FAM157A	intronic	HIVID	Zhao et al.2016	27703150	Tumor
chr1	121485435		EMBP1(dist=171749),NONE(dist=NONE)	intergenic	HIVID	Zhao et al.2016	27703150	Tumor
chr1	121485392		EMBP1(dist=171706),NONE(dist=NONE)	intergenic	HIVID	Zhao et al.2016	27703150	Tumor
chr1	121485052		EMBP1(dist=171366),NONE(dist=NONE)	intergenic	HIVID	Zhao et al.2016	27703150	Tumor
chr2	114361058		DDX11L2	ncRNA_intronic	HIVID	Zhao et al.2016	27703150	Tumor
chr2	114360956		DDX11L2	ncRNA_intronic	HIVID	Zhao et al.2016	27703150	Tumor
chr2	114360975		DDX11L2	ncRNA_intronic	HIVID	Zhao et al.2016	27703150	Tumor
chr12	34852738		ALG10(dist=671502),NONE(dist=NONE)	intergenic	HIVID	Zhao et al.2016	27703150	Tumor
chr12	34852650		ALG10(dist=671414),NONE(dist=NONE)	intergenic	HIVID	Zhao et al.2016	27703150	Tumor
chr7	61974921		NONE(dist=NONE),ZNF733P(dist=776749)	intergenic	HIVID	Zhao et al.2016	27703150	Tumor
chr10	42398698		NONE(dist=NONE),LOC441666(dist=428616)	intergenic	HIVID	Zhao et al.2016	27703150	Tumor
chr19	27733212		NONE(dist=NONE),LINC00662(dist=548189)	intergenic	HIVID	Zhao et al.2016	27703150	Tumor
chr19	27736949		NONE(dist=NONE),LINC00662(dist=544452)	intergenic	HIVID	Zhao et al.2016	27703150	Tumor
chr19	27737967		NONE(dist=NONE),LINC00662(dist=543434)	intergenic	HIVID	Zhao et al.2016	27703150	Tumor
chr5	1299924		TERT;MIR4457	promoter;downstream	HIVID	Zhao et al.2016	27703150	Tumor
chr22	18039331		SLC25A18;CECR2	promoter;downstream	HIVID	Zhao et al.2016	27703150	Tumor
chr3	196625648		SENP5	intronic	HIVID	Zhao et al.2016	27703150	Tumor
chr11	51570038		OR4C46(dist=53827),NONE(dist=NONE)	intergenic	HIVID	Zhao et al.2016	27703150	Tumor
chr22	17065013		OR11H1(dist=615209),CCT8L2(dist=6635)	intergenic	HIVID	Zhao et al.2016	27703150	Tumor
chr10	42599820		NONE(dist=NONE),LOC441666(dist=227494)	intergenic	HIVID	Zhao et al.2016	27703150	Tumor
chr17	22261294		MTRNR2L1(dist=237303),NONE(dist=NONE)	intergenic	HIVID	Zhao et al.2016	27703150	Tumor
chr9	6832340		KDM4C	intronic	HIVID	Zhao et al.2016	27703150	Tumor
chr17	21855643		FAM27L(dist=29144),FLJ36000(dist=48419)	intergenic	HIVID	Zhao et al.2016	27703150	Tumor
chr22	18024931		CECR2	intronic	HIVID	Zhao et al.2016	27703150	Tumor
chr10	109828998		SORCS1(dist=904532),RNU6-53P(dist=1101417)	intergenic	HIVID	Zhao et al.2016	27703150	Tumor
chr3	126962719		C3orf56(dist=45692),RP11-88I21.2(dist=78431)	intergenic	HIVID	Zhao et al.2016	27703150	Tumor
chr8	35280415		UNC5D	intronic	HIVID	Zhao et al.2016	27703150	Tumor
chr8	41350357		GOLGA7	intronic	HIVID	Zhao et al.2016	27703150	Tumor
chr1	111480380		CD53(dist=37822),LRIF1(dist=9432)	intergenic	HIVID	Zhao et al.2016	27703150	Tumor
chr12	53578518		ZNF740	intronic	HIVID	Zhao et al.2016	27703150	Tumor
chr16	56939369		SLC12A3	intronic	HIVID	Zhao et al.2016	27703150	Tumor
chr1	35668568		SFPQ	promoter	HIVID	Zhao et al.2016	27703150	Tumor
chr7	72510936		PMS2P5	ncRNA_intronic	HIVID	Zhao et al.2016	27703150	Tumor
chr10	105669175		OBFC1	intronic	HIVID	Zhao et al.2016	27703150	Tumor
chr10	42378082		NONE(dist=NONE),LOC441666(dist=449232)	intergenic	HIVID	Zhao et al.2016	27703150	Tumor
chr10	42378123		NONE(dist=NONE),LOC441666(dist=449191)	intergenic	HIVID	Zhao et al.2016	27703150	Tumor
chr16	57053402		NLRC5	intronic	HIVID	Zhao et al.2016	27703150	Tumor
chr13	35882660		NBEA	intronic	HIVID	Zhao et al.2016	27703150	Tumor
chr13	35882757		NBEA	intronic	HIVID	Zhao et al.2016	27703150	Tumor
chr6	150735695		IYD(dist=9930),PLEKHG1(dist=185304)	intergenic	HIVID	Zhao et al.2016	27703150	Tumor
chr5	61730880		IPO11	intronic	HIVID	Zhao et al.2016	27703150	Tumor
chr8	77284015		HNF4G(dist=804954),LINC01111(dist=34874)	intergenic	HIVID	Zhao et al.2016	27703150	Tumor
chr8	77283987		HNF4G(dist=804926),LINC01111(dist=34902)	intergenic	HIVID	Zhao et al.2016	27703150	Tumor
chr8	43096944		HGSNAT(dist=38974),POTEA(dist=50641)	intergenic	HIVID	Zhao et al.2016	27703150	Tumor
chr8	43093876		HGSNAT(dist=35906),POTEA(dist=53709)	intergenic	HIVID	Zhao et al.2016	27703150	Tumor
chr8	43093002		HGSNAT(dist=35032),POTEA(dist=54583)	intergenic	HIVID	Zhao et al.2016	27703150	Tumor
chr1	91934351		HFM1(dist=63925),CDC7(dist=32053)	intergenic	HIVID	Zhao et al.2016	27703150	Tumor
chr16	74871815		FA2H(dist=63086),WDR59(dist=35656)	intergenic	HIVID	Zhao et al.2016	27703150	Tumor
chr4	49659606		CWH43(dist=595511),NONE(dist=NONE)	intergenic	HIVID	Zhao et al.2016	27703150	Tumor
chr4	49659565		CWH43(dist=595470),NONE(dist=NONE)	intergenic	HIVID	Zhao et al.2016	27703150	Tumor
chr4	49646831		CWH43(dist=582736),NONE(dist=NONE)	intergenic	HIVID	Zhao et al.2016	27703150	Tumor
chr4	49646790		CWH43(dist=582695),NONE(dist=NONE)	intergenic	HIVID	Zhao et al.2016	27703150	Tumor
chr7	155343869		CNPY1(dist=17330),RBM33(dist=93334)	intergenic	HIVID	Zhao et al.2016	27703150	Tumor
chr19	30303503		CCNE1	intronic	HIVID	Zhao et al.2016	27703150	Tumor

Chromosome	Integration site in host genome	Integration site in virus genome	Gene (distance, bp)	Regions	Methods	Author	PMID	Sample
chr3	86434244		CADM2(dist=310665),RNU6-69P(dist=56070)	intergenic	HIVID	Zhao et al.2016	27703150	Tumor
chr7	111989087		ZNF277(dist=5098),IFRD1(dist=74112)	intergenic	HIVID	Zhao et al.2016	27703150	Tumor
chr8	146241975		ZNF252P-AS1(dist=10543),C8orf33(dist=35849)	intergenic	HIVID	Zhao et al.2016	27703150	Tumor
chr9	10200468		PTPRD	intronic	HIVID	Zhao et al.2016	27703150	Tumor
chr9	10444339		PTPRD	intronic	HIVID	Zhao et al.2016	27703150	Tumor
chr4	24305761		PPARGC1A(dist=414061),MIR573(dist=216054)	intergenic	HIVID	Zhao et al.2016	27703150	Tumor
chr19	727214		PALM	intronic	HIVID	Zhao et al.2016	27703150	Tumor
chr5	11845		NONE(dist=NONE),PLEKHG4B(dist=128528)	intergenic	HIVID	Zhao et al.2016	27703150	Tumor
chr10	42391443		NONE(dist=NONE),LOC441666(dist=435871)	intergenic	HIVID	Zhao et al.2016	27703150	Tumor
chr6	41326965		NCR2(dist=8340),FOXP4(dist=187199)	intergenic	HIVID	Zhao et al.2016	27703150	Tumor
chr6	41326883		NCR2(dist=8258),FOXP4(dist=187281)	intergenic	HIVID	Zhao et al.2016	27703150	Tumor
chr5	13136076		LINC01194(dist=330781),DNAH5(dist=554361)	intergenic	HIVID	Zhao et al.2016	27703150	Tumor
chr13	86161361		LINC00351(dist=42564),SLITRK6(dist=205561)	intergenic	HIVID	Zhao et al.2016	27703150	Tumor
chr2	20383105		LAPTM4A(dist=131316),SDC1(dist=17453)	intergenic	HIVID	Zhao et al.2016	27703150	Tumor
chr10	135524594		DUX4L7(dist=26136),NONE(dist=NONE)	intergenic	HIVID	Zhao et al.2016	27703150	Tumor
chr7	111578583		DOCK4	intronic	HIVID	Zhao et al.2016	27703150	Tumor
chr13	115087715		CHAMP1	intronic	HIVID	Zhao et al.2016	27703150	Tumor
chr13	115012692		CDC16	intronic	HIVID	Zhao et al.2016	27703150	Tumor
chr4	122741100		CCNA2	intronic	HIVID	Zhao et al.2016	27703150	Tumor
chr4	122741076		CCNA2	intronic	HIVID	Zhao et al.2016	27703150	Tumor
chr18	55514520		ATP8B1(dist=44193),NEDD4L(dist=197090)	intergenic	HIVID	Zhao et al.2016	27703150	Tumor
chr2	232056607		ARMC9	promoter	HIVID	Zhao et al.2016	27703150	Tumor
chr2	232058571		ARMC9	promoter	HIVID	Zhao et al.2016	27703150	Tumor
chr19	39135109		ACTN4;EIF3K	promoter;downstream	HIVID	Zhao et al.2016	27703150	Tumor
chr7	11668104		THSD7A	intronic	HIVID	Zhao et al.2016	27703150	Tumor
chr5	1295121		TERT	UTR5	HIVID	Zhao et al.2016	27703150	Tumor
chr5	1295071		TERT	exonic	HIVID	Zhao et al.2016	27703150	Tumor
chr16	1106514		SOX8(dist=69535),SSTR5-AS1(dist=7568)	intergenic	HIVID	Zhao et al.2016	27703150	Tumor
chr5	121590187		LOC100505841(dist=71829),SNCAIP(dist=57633)	intergenic	HIVID	Zhao et al.2016	27703150	Tumor
chr5	27937756		LINC01021(dist=441248),LSP1P3(dist=989221)	intergenic	HIVID	Zhao et al.2016	27703150	Tumor
chr17	15034581		CDRT8(dist=25077),PMP22(dist=98513)	intergenic	HIVID	Zhao et al.2016	27703150	Tumor
chr5	1299220		TERT	promoter	HIVID	Zhao et al.2016	27703150	Tumor
chr5	1299262		TERT	promoter	HIVID	Zhao et al.2016	27703150	Tumor
chr4	67416818		RP11-807H7.1(dist=857714),CENPC(dist=921171)	intergenic	HIVID	Zhao et al.2016	27703150	Tumor
chr4	67591805		RP11-807H7.1(dist=1032701),CENPC(dist=746184)	intergenic	HIVID	Zhao et al.2016	27703150	Tumor
chr10	42387974		NONE(dist=NONE),LOC441666(dist=439340)	intergenic	HIVID	Zhao et al.2016	27703150	Tumor
chr10	42391511		NONE(dist=NONE),LOC441666(dist=435803)	intergenic	HIVID	Zhao et al.2016	27703150	Tumor
chr16	9836561		MIR7641-2(dist=178313),GRIN2A(dist=10704)	intergenic	HIVID	Zhao et al.2016	27703150	Tumor
chr16	9835820		MIR7641-2(dist=177572),GRIN2A(dist=11445)	intergenic	HIVID	Zhao et al.2016	27703150	Tumor
chr5	172609128		BNIP1(dist=17738),NKX2-5(dist=49979)	intergenic	HIVID	Zhao et al.2016	27703150	Tumor
chr9	67320747		AQP7P1(dist=31255),FAM27E3(dist=464197)	intergenic	HIVID	Zhao et al.2016	27703150	Tumor
chr5	5910453		KIAA0947(dist=420106),FLJ33360(dist=400101)	intergenic	HIVID	Zhao et al.2016	27703150	Tumor
chr18	33737367		ELP2	intronic	HIVID	Zhao et al.2016	27703150	Tumor
chr19	36212967		KMT2B	intronic	HIVID	Zhao et al.2016	27703150	Tumor
chr9	123289739		CDK5RAP2	intronic	HIVID	Zhao et al.2016	27703150	Tumor
chr2	227265802		LOC646736(dist=221024),MIR5702(dist=257624)	intergenic	HIVID	Zhao et al.2016	27703150	Tumor
chr19	36212956		KMT2B	intronic	HIVID	Zhao et al.2016	27703150	Tumor
chr19	36212927		KMT2B	intronic	HIVID	Zhao et al.2016	27703150	Tumor
chr19	36212950		KMT2B	intronic	HIVID	Zhao et al.2016	27703150	Tumor
chr19	36212992		KMT2B	intronic	HIVID	Zhao et al.2016	27703150	Tumor
chr2	211519161		CPS1	intronic	HIVID	Zhao et al.2016	27703150	Tumor
chr7	139198706		CLEC2L	promoter	HIVID	Zhao et al.2016	27703150	Tumor
chr5	1303477		TERT;MIR4457	promoter;downstream	HIVID	Zhao et al.2016	27703150	Tumor
chr21	10845809		TEKT4P2(dist=877216),TPTE(dist=60934)	intergenic	HIVID	Zhao et al.2016	27703150	Tumor
chr21	10845743		TEKT4P2(dist=877150),TPTE(dist=61000)	intergenic	HIVID	Zhao et al.2016	27703150	Tumor
chr21	10832683		TEKT4P2(dist=864090),TPTE(dist=74060)	intergenic	HIVID	Zhao et al.2016	27703150	Tumor
chr21	10832623		TEKT4P2(dist=864030),TPTE(dist=74120)	intergenic	HIVID	Zhao et al.2016	27703150	Tumor
chr21	10832178		TEKT4P2(dist=863585),TPTE(dist=74565)	intergenic	HIVID	Zhao et al.2016	27703150	Tumor
chr21	10832142		TEKT4P2(dist=863549),TPTE(dist=74601)	intergenic	HIVID	Zhao et al.2016	27703150	Tumor
chr21	10832118		TEKT4P2(dist=863525),TPTE(dist=74625)	intergenic	HIVID	Zhao et al.2016	27703150	Tumor
chr21	10819509		TEKT4P2(dist=850916),TPTE(dist=87234)	intergenic	HIVID	Zhao et al.2016	27703150	Tumor
chr21	10819493		TEKT4P2(dist=850900),TPTE(dist=87250)	intergenic	HIVID	Zhao et al.2016	27703150	Tumor
chr21	10819399		TEKT4P2(dist=850806),TPTE(dist=87344)	intergenic	HIVID	Zhao et al.2016	27703150	Tumor
chr1	4363250		RP13-614K11.1(dist=350607),RP5-1166F10.1(dist=108861)	intergenic	HIVID	Zhao et al.2016	27703150	Tumor
chr1	191858928		RP11-463J7.2(dist=1088140),RGS18(dist=268664)	intergenic	HIVID	Zhao et al.2016	27703150	Tumor
chr5	126921604		PRRC1(dist=30824),CTXN3(dist=63109)	intergenic	HIVID	Zhao et al.2016	27703150	Tumor
chr6	57403838		PRIM2	intronic	HIVID	Zhao et al.2016	27703150	Tumor
chr6	57403756		PRIM2	intronic	HIVID	Zhao et al.2016	27703150	Tumor
chr14	19636593		POTEG(dist=51651),DUXAP10(dist=13439)	intergenic	HIVID	Zhao et al.2016	27703150	Tumor
chr15	101924247		PCSK6	UTR3	HIVID	Zhao et al.2016	27703150	Tumor
chr4	61591966		NONE(dist=NONE),LPHN3(dist=770873)	intergenic	HIVID	Zhao et al.2016	27703150	Tumor
chr4	61592373		NONE(dist=NONE),LPHN3(dist=770466)	intergenic	HIVID	Zhao et al.2016	27703150	Tumor
chr4	60925243		NONE(dist=NONE),LPHN3(dist=1437596)	intergenic	HIVID	Zhao et al.2016	27703150	Tumor
chr10	42359353		NONE(dist=NONE),LOC441666(dist=467961)	intergenic	HIVID	Zhao et al.2016	27703150	Tumor
chr10	42359765		NONE(dist=NONE),LOC441666(dist=467549)	intergenic	HIVID	Zhao et al.2016	27703150	Tumor
chr10	42359808		NONE(dist=NONE),LOC441666(dist=467506)	intergenic	HIVID	Zhao et al.2016	27703150	Tumor
chr10	42360461		NONE(dist=NONE),LOC441666(dist=466853)	intergenic	HIVID	Zhao et al.2016	27703150	Tumor
chr10	42360521		NONE(dist=NONE),LOC441666(dist=466793)	intergenic	HIVID	Zhao et al.2016	27703150	Tumor
chr10	42376683		NONE(dist=NONE),LOC441666(dist=450631)	intergenic	HIVID	Zhao et al.2016	27703150	Tumor
chr10	42376721		NONE(dist=NONE),LOC441666(dist=450593)	intergenic	HIVID	Zhao et al.2016	27703150	Tumor
chr10	42380230		NONE(dist=NONE),LOC441666(dist=447084)	intergenic	HIVID	Zhao et al.2016	27703150	Tumor
chr10	42380306		NONE(dist=NONE),LOC441666(dist=447008)	intergenic	HIVID	Zhao et al.2016	27703150	Tumor
chr10	42380316		NONE(dist=NONE),LOC441666(dist=446998)	intergenic	HIVID	Zhao et al.2016	27703150	Tumor
chr10	42382883		NONE(dist=NONE),LOC441666(dist=444431)	intergenic	HIVID	Zhao et al.2016	27703150	Tumor
chr10	42382928		NONE(dist=NONE),LOC441666(dist=444386)	intergenic	HIVID	Zhao et al.2016	27703150	Tumor
chr10	42800169		NONE(dist=NONE),LOC441666(dist=27145)	intergenic	HIVID	Zhao et al.2016	27703150	Tumor
chr10	42800209		NONE(dist=NONE),LOC441666(dist=27105)	intergenic	HIVID	Zhao et al.2016	27703150	Tumor
chr10	42800248		NONE(dist=NONE),LOC441666(dist=27066)	intergenic	HIVID	Zhao et al.2016	27703150	Tumor
chr19	27732079		NONE(dist=NONE),LINC00662(dist=549322)	intergenic	HIVID	Zhao et al.2016	27703150	Tumor
chr20	29818462		MLLT10P1(dist=180324),DEFB115(dist=27005)	intergenic	HIVID	Zhao et al.2016	27703150	Tumor
chr20	29818404		MLLT10P1(dist=180266),DEFB115(dist=27063)	intergenic	HIVID	Zhao et al.2016	27703150	Tumor
chr2	89879140		MIR4436A(dist=767172),LOC654342(dist=1945569)	intergenic	HIVID	Zhao et al.2016	27703150	Tumor
chr2	89879092		MIR4436A(dist=767124),LOC654342(dist=1945617)	intergenic	HIVID	Zhao et al.2016	27703150	Tumor
chr2	89877898		MIR4436A(dist=765930),LOC654342(dist=1946811)	intergenic	HIVID	Zhao et al.2016	27703150	Tumor

Chromosome	Integration site in host genome	Integration site in virus genome	Gene (distance, bp)	Regions	Methods	Author	PMID	Sample
chr2	89877528		MIR4436A(dist=765560),LOC654342(dist=1947181)	intergenic	HIVID	Zhao et al.2016	27703150	Tumor
chr2	89876642		MIR4436A(dist=764674),LOC654342(dist=1948067)	intergenic	HIVID	Zhao et al.2016	27703150	Tumor
chr2	89875282		MIR4436A(dist=763314),LOC654342(dist=1949427)	intergenic	HIVID	Zhao et al.2016	27703150	Tumor
chr2	89870287		MIR4436A(dist=758319),LOC654342(dist=1954422)	intergenic	HIVID	Zhao et al.2016	27703150	Tumor
chr10	59269017		MIR3924(dist=204698),IPMK(dist=682261)	intergenic	HIVID	Zhao et al.2016	27703150	Tumor
chr10	38782504		LINC00999(dist=41423),ACTR3BP5(dist=207223)	intergenic	HIVID	Zhao et al.2016	27703150	Tumor
chr10	38782440		LINC00999(dist=41359),ACTR3BP5(dist=207287)	intergenic	HIVID	Zhao et al.2016	27703150	Tumor
chr10	38782260		LINC00999(dist=41179),ACTR3BP5(dist=207467)	intergenic	HIVID	Zhao et al.2016	27703150	Tumor
chr10	38782200		LINC00999(dist=41119),ACTR3BP5(dist=207527)	intergenic	HIVID	Zhao et al.2016	27703150	Tumor
chr10	38776419		LINC00999(dist=35338),ACTR3BP5(dist=213308)	intergenic	HIVID	Zhao et al.2016	27703150	Tumor
chr15	96445183		LINC00924(dist=394107),NR2F2-AS1(dist=364433)	intergenic	HIVID	Zhao et al.2016	27703150	Tumor
chr5	4164496		IRX1(dist=562979),LOC101929153(dist=609098)	intergenic	HIVID	Zhao et al.2016	27703150	Tumor
chr2	213002526		ERBB4	intronic	HIVID	Zhao et al.2016	27703150	Tumor
chr1	121485435		EMBP1(dist=171749),NONE(dist=NONE)	intergenic	HIVID	Zhao et al.2016	27703150	Tumor
chr1	121485255		EMBP1(dist=171569),NONE(dist=NONE)	intergenic	HIVID	Zhao et al.2016	27703150	Tumor
chr14	19938798		DUXAP10(dist=13464),POTEM(dist=45156)	intergenic	HIVID	Zhao et al.2016	27703150	Tumor
chr4	49157195		CWH43(dist=93100),NONE(dist=NONE)	intergenic	HIVID	Zhao et al.2016	27703150	Tumor
chr4	49157164		CWH43(dist=93069),NONE(dist=NONE)	intergenic	HIVID	Zhao et al.2016	27703150	Tumor
chr4	49156651		CWH43(dist=92556),NONE(dist=NONE)	intergenic	HIVID	Zhao et al.2016	27703150	Tumor
chr4	49151894		CWH43(dist=87799),NONE(dist=NONE)	intergenic	HIVID	Zhao et al.2016	27703150	Tumor
chr4	49151819		CWH43(dist=87724),NONE(dist=NONE)	intergenic	HIVID	Zhao et al.2016	27703150	Tumor
chr4	49151787		CWH43(dist=87692),NONE(dist=NONE)	intergenic	HIVID	Zhao et al.2016	27703150	Tumor
chr4	49151539		CWH43(dist=87444),NONE(dist=NONE)	intergenic	HIVID	Zhao et al.2016	27703150	Tumor
chr4	49149611		CWH43(dist=85516),NONE(dist=NONE)	intergenic	HIVID	Zhao et al.2016	27703150	Tumor
chr4	49149565		CWH43(dist=85470),NONE(dist=NONE)	intergenic	HIVID	Zhao et al.2016	27703150	Tumor
chr4	49146582		CWH43(dist=82487),NONE(dist=NONE)	intergenic	HIVID	Zhao et al.2016	27703150	Tumor
chr4	49145635		CWH43(dist=81540),NONE(dist=NONE)	intergenic	HIVID	Zhao et al.2016	27703150	Tumor
chr4	49139321		CWH43(dist=75226),NONE(dist=NONE)	intergenic	HIVID	Zhao et al.2016	27703150	Tumor
chr4	49134923		CWH43(dist=70828),NONE(dist=NONE)	intergenic	HIVID	Zhao et al.2016	27703150	Tumor
chr4	49126036		CWH43(dist=61941),NONE(dist=NONE)	intergenic	HIVID	Zhao et al.2016	27703150	Tumor
chr4	49125943		CWH43(dist=61848),NONE(dist=NONE)	intergenic	HIVID	Zhao et al.2016	27703150	Tumor
chr4	49650566		CWH43(dist=586471),NONE(dist=NONE)	intergenic	HIVID	Zhao et al.2016	27703150	Tumor
chr4	49650498		CWH43(dist=586403),NONE(dist=NONE)	intergenic	HIVID	Zhao et al.2016	27703150	Tumor
chr4	49647189		CWH43(dist=583094),NONE(dist=NONE)	intergenic	HIVID	Zhao et al.2016	27703150	Tumor
chr4	49640180		CWH43(dist=576085),NONE(dist=NONE)	intergenic	HIVID	Zhao et al.2016	27703150	Tumor
chr4	49639941		CWH43(dist=575846),NONE(dist=NONE)	intergenic	HIVID	Zhao et al.2016	27703150	Tumor
chr4	49637184		CWH43(dist=573089),NONE(dist=NONE)	intergenic	HIVID	Zhao et al.2016	27703150	Tumor
chr4	49120671		CWH43(dist=56576),NONE(dist=NONE)	intergenic	HIVID	Zhao et al.2016	27703150	Tumor
chr4	49120646		CWH43(dist=56551),NONE(dist=NONE)	intergenic	HIVID	Zhao et al.2016	27703150	Tumor
chr4	49120585		CWH43(dist=56490),NONE(dist=NONE)	intergenic	HIVID	Zhao et al.2016	27703150	Tumor
chr4	49115853		CWH43(dist=51758),NONE(dist=NONE)	intergenic	HIVID	Zhao et al.2016	27703150	Tumor
chr4	49115772		CWH43(dist=51677),NONE(dist=NONE)	intergenic	HIVID	Zhao et al.2016	27703150	Tumor
chr4	49104775		CWH43(dist=40680),NONE(dist=NONE)	intergenic	HIVID	Zhao et al.2016	27703150	Tumor
chr4	49104698		CWH43(dist=40603),NONE(dist=NONE)	intergenic	HIVID	Zhao et al.2016	27703150	Tumor
chr22	16206430		BMS1P18(dist=34165),POTEH(dist=49902)	intergenic	HIVID	Zhao et al.2016	27703150	Tumor
chr10	39083856		ACTR3BP5(dist=92485),NONE(dist=NONE)	intergenic	HIVID	Zhao et al.2016	27703150	Tumor
chr10	39083798		ACTR3BP5(dist=92427),NONE(dist=NONE)	intergenic	HIVID	Zhao et al.2016	27703150	Tumor
chr3	90076493		EPHA3(dist=545209),NONE(dist=NONE)	intergenic	HIVID	Zhao et al.2016	27703150	Tumor
chr5	1296156		TERT	promoter	HIVID	Zhao et al.2016	27703150	Tumor
chr2	65262332		SLC1A4(dist=11332),CEP68(dist=21163)	intergenic	HIVID	Zhao et al.2016	27703150	Tumor
chr18	78016280		PARD6G(dist=10883),NONE(dist=NONE)	intergenic	HIVID	Zhao et al.2016	27703150	Tumor
chr18	78016274		PARD6G(dist=10877),NONE(dist=NONE)	intergenic	HIVID	Zhao et al.2016	27703150	Tumor
chr4	53247336		SPATA18(dist=283878),USP46(dist=209791)	intergenic	HIVID	Zhao et al.2016	27703150	Tumor
chr4	54024530		SCFD2	intronic	HIVID	Zhao et al.2016	27703150	Tumor
chr5	26125496		RP11-730N24.1(dist=1284804),CDH9(dist=755213)	intergenic	HIVID	Zhao et al.2016	27703150	Tumor
chr2	130794726		FAR2P1	ncRNA_intronic	HIVID	Zhao et al.2016	27703150	Tumor
chr9	23758080		ELAVL2	intronic	HIVID	Zhao et al.2016	27703150	Tumor
chr16	68050100		DUS2,LOC100131303;DDX28	promoter;downstream	HIVID	Zhao et al.2016	27703150	Tumor
chr5	178188656		ZNF354A(dist=30953),AACSP1(dist=3208)	intergenic	HIVID	Zhao et al.2016	27703150	Tumor
chr5	176228562		UNC5A	promoter	HIVID	Zhao et al.2016	27703150	Tumor
chr5	176228622		UNC5A	promoter	HIVID	Zhao et al.2016	27703150	Tumor
chr5	178975658		RUFY1	promoter	HIVID	Zhao et al.2016	27703150	Tumor
chr8	43827149		POTEA(dist=608821),NONE(dist=NONE)	intergenic	HIVID	Zhao et al.2016	27703150	Tumor
chr8	43825289		POTEA(dist=606961),NONE(dist=NONE)	intergenic	HIVID	Zhao et al.2016	27703150	Tumor
chr8	43823421		POTEA(dist=605093),NONE(dist=NONE)	intergenic	HIVID	Zhao et al.2016	27703150	Tumor
chr1	243210821		PLD5(dist=522823),RP11-261C10.3(dist=8795)	intergenic	HIVID	Zhao et al.2016	27703150	Tumor
chr7	82689678		PCLO	intronic	HIVID	Zhao et al.2016	27703150	Tumor
chr8	46843107		NONE(dist=NONE),LINC00293(dist=909401)	intergenic	HIVID	Zhao et al.2016	27703150	Tumor
chr8	46844975		NONE(dist=NONE),LINC00293(dist=907533)	intergenic	HIVID	Zhao et al.2016	27703150	Tumor
chr8	46846843		NONE(dist=NONE),LINC00293(dist=905665)	intergenic	HIVID	Zhao et al.2016	27703150	Tumor
chr8	46848712		NONE(dist=NONE),LINC00293(dist=903796)	intergenic	HIVID	Zhao et al.2016	27703150	Tumor
chr8	46850581		NONE(dist=NONE),LINC00293(dist=901927)	intergenic	HIVID	Zhao et al.2016	27703150	Tumor
chr8	46852449		NONE(dist=NONE),LINC00293(dist=900059)	intergenic	HIVID	Zhao et al.2016	27703150	Tumor
chr6	140018		NONE(dist=NONE),LINC00266-3(dist=246)	intergenic	HIVID	Zhao et al.2016	27703150	Tumor
chr4	154340618		MND1(dist=4371),KIAA0922(dist=46880)	intergenic	HIVID	Zhao et al.2016	27703150	Tumor
chr13	57093909		MIR5007(dist=1345226),PRR20A(dist=621143)	intergenic	HIVID	Zhao et al.2016	27703150	Tumor
chr5	179725427		MAPK9;GFPT2	promoter;downstream	HIVID	Zhao et al.2016	27703150	Tumor
chr8	75002210		LY96(dist=60903),JPH1(dist=144729)	intergenic	HIVID	Zhao et al.2016	27703150	Tumor
chr5	180761106		LOC100133331(dist=5910),OR4F16(dist=33182)	intergenic	HIVID	Zhao et al.2016	27703150	Tumor
chr1	334491		LOC100133331(dist=5910),OR4F16(dist=33168)	intergenic	HIVID	Zhao et al.2016	27703150	Tumor
chr19	36213252		KMT2B	intronic	HIVID	Zhao et al.2016	27703150	Tumor
chr19	36211459		KMT2B	exonic	HIVID	Zhao et al.2016	27703150	Tumor
chr8	42629631		CHRNA6	promoter	HIVID	Zhao et al.2016	27703150	Tumor
chr19	10173122		C3P1	ncRNA_intronic	HIVID	Zhao et al.2016	27703150	Tumor
chr15	29214025		APBA2	intronic	HIVID	Zhao et al.2016	27703150	Tumor
chr8	57023147		RPS20(dist=36007),MOS(dist=2354)	intergenic	HIVID	Zhao et al.2016	27703150	Tumor
chr18	65417137		RP11-638L3.1	ncRNA_intronic	HIVID	Zhao et al.2016	27703150	Tumor
chr3	191475127		PYDC2(dist=295882),FGF12(dist=382055)	intergenic	HIVID	Zhao et al.2016	27703150	Tumor
chr3	191474941		PYDC2(dist=295696),FGF12(dist=382241)	intergenic	HIVID	Zhao et al.2016	27703150	Tumor
chr19	36212956		KMT2B	intronic	HIVID	Zhao et al.2016	27703150	Tumor
chr8	38955196		ADAM9	intronic	HIVID	Zhao et al.2016	27703150	Tumor
chr2	1099708		SNTG2	intronic	HIVID	Zhao et al.2016	27703150	Tumor
chr5	36945511		NIPBL	intronic	HIVID	Zhao et al.2016	27703150	Tumor
chr17	65223701		HELZ	intronic	HIVID	Zhao et al.2016	27703150	Tumor

Supplementary Table S8 Continued

Chromosome	Integration site in host genome	Integration site in virus genome	Gene (distance, bp)	Regions	Methods	Author	PMID	Sample
chr16	32163622		ZNF267(dist=234993),HERC2P4(dist=17742)	intergenic	HIVID	Zhao et al.2016	27703150	Tumor
chr16	32786508		TP53TG3B(dist=99060),SLC6A10P(dist=102289)	intergenic	HIVID	Zhao et al.2016	27703150	Tumor
chr19	32127865		THEG5(dist=43409),ZNF507(dist=708649)	intergenic	HIVID	Zhao et al.2016	27703150	Tumor
chr19	32127833		THEG5(dist=43377),ZNF507(dist=708681)	intergenic	HIVID	Zhao et al.2016	27703150	Tumor
chr5	1295123		TERT	UTR5	HIVID	Zhao et al.2016	27703150	Tumor
chr5	1295105		TERT	UTR5	HIVID	Zhao et al.2016	27703150	Tumor
chr5	1295081		TERT	exonic	HIVID	Zhao et al.2016	27703150	Tumor
chr16	33106535		SLC6A10P(dist=210072),TP53TG3C(dist=99050)	intergenic	HIVID	Zhao et al.2016	27703150	Tumor
chr7	61970161		NONE(dist=NONE),ZNF733P(dist=781509)	intergenic	HIVID	Zhao et al.2016	27703150	Tumor
chr10	42528105		NONE(dist=NONE),LOC441666(dist=299209)	intergenic	HIVID	Zhao et al.2016	27703150	Tumor
chr19	27733872		NONE(dist=NONE),LINC00662(dist=547529)	intergenic	HIVID	Zhao et al.2016	27703150	Tumor
chr19	27734211		NONE(dist=NONE),LINC00662(dist=547190)	intergenic	HIVID	Zhao et al.2016	27703150	Tumor
chr19	27734328		NONE(dist=NONE),LINC00662(dist=547073)	intergenic	HIVID	Zhao et al.2016	27703150	Tumor
chr19	27736707		NONE(dist=NONE),LINC00662(dist=544694)	intergenic	HIVID	Zhao et al.2016	27703150	Tumor
chr19	27736814		NONE(dist=NONE),LINC00662(dist=544587)	intergenic	HIVID	Zhao et al.2016	27703150	Tumor
chr19	27738629		NONE(dist=NONE),LINC00662(dist=542772)	intergenic	HIVID	Zhao et al.2016	27703150	Tumor
chr3	160030436		IFT80	intronic	HIVID	Zhao et al.2016	27703150	Tumor
chr15	28900991		HERC2P9	ncRNA_exonic	HIVID	Zhao et al.2016	27703150	Tumor
chr15	28459070		HERC2	exonic	HIVID	Zhao et al.2016	27703150	Tumor
chr17	19130020		GRAPL(dist=67872),EPN2(dist=10670)	intergenic	HIVID	Zhao et al.2016	27703150	Tumor
chr17	19054861		GRAPL	intronic	HIVID	Zhao et al.2016	27703150	Tumor
chr17	19003852		GRAP(dist=53516),GRAPL(dist=26930)	intergenic	HIVID	Zhao et al.2016	27703150	Tumor
chr1	121485201		EMBP1(dist=171515),NONE(dist=NONE)	intergenic	HIVID	Zhao et al.2016	27703150	Tumor
chr1	121484182		EMBP1(dist=170496),NONE(dist=NONE)	intergenic	HIVID	Zhao et al.2016	27703150	Tumor
chr15	86032860		AKAP13	intronic	HIVID	Zhao et al.2016	27703150	Tumor
chr15	86032872		AKAP13	intronic	HIVID	Zhao et al.2016	27703150	Tumor
chr8	126663502		TRIB1(dist=212855),LINC00861(dist=271265)	intergenic	HIVID	Zhao et al.2016	27703150	Tumor
chr12	85231052		TMTC2(dist=1702985),SLC6A15(dist=22215)	intergenic	HIVID	Zhao et al.2016	27703150	Tumor
chr12	85230999		TMTC2(dist=1702932),SLC6A15(dist=22268)	intergenic	HIVID	Zhao et al.2016	27703150	Tumor
chr12	33695936		SYT10(dist=103182),ALG10(dist=479280)	intergenic	HIVID	Zhao et al.2016	27703150	Tumor
chr12	33693084		SYT10(dist=100330),ALG10(dist=482132)	intergenic	HIVID	Zhao et al.2016	27703150	Tumor
chr9	113273807		SVEP1	intronic	HIVID	Zhao et al.2016	27703150	Tumor
chr13	87999907		SLITRK6(dist=1626424),MIR4500HG(dist=96335)	intergenic	HIVID	Zhao et al.2016	27703150	Tumor
chr13	87999864		SLITRK6(dist=1626381),MIR4500HG(dist=96378)	intergenic	HIVID	Zhao et al.2016	27703150	Tumor
chr3	164942033		SLITRK3(dist=27564),BCHE(dist=548659)	intergenic	HIVID	Zhao et al.2016	27703150	Tumor
chr3	164941986		SLITRK3(dist=27517),BCHE(dist=548706)	intergenic	HIVID	Zhao et al.2016	27703150	Tumor
chr4	1708951		SLBP	intronic	HIVID	Zhao et al.2016	27703150	Tumor
chr4	1709026		SLBP	intronic	HIVID	Zhao et al.2016	27703150	Tumor
chr14	68618535		RAD51B	intronic	HIVID	Zhao et al.2016	27703150	Tumor
chr14	68618577		RAD51B	intronic	HIVID	Zhao et al.2016	27703150	Tumor
chr10	42382507		NONE(dist=NONE),LOC441666(dist=444807)	intergenic	HIVID	Zhao et al.2016	27703150	Tumor
chr10	15253350		NMT2(dist=42655),FAM171A1(dist=294)	intergenic	HIVID	Zhao et al.2016	27703150	Tumor
chr10	15253308		NMT2(dist=42613),FAM171A1(dist=336)	intergenic	HIVID	Zhao et al.2016	27703150	Tumor
chr3	175948910		NAALADL2(dist=425482),LINC01208(dist=373026)	intergenic	HIVID	Zhao et al.2016	27703150	Tumor
chr12	66451465		MIR6074(dist=33959),LLPH(dist=65384)	intergenic	HIVID	Zhao et al.2016	27703150	Tumor
chr12	66451463		MIR6074(dist=33957),LLPH(dist=65386)	intergenic	HIVID	Zhao et al.2016	27703150	Tumor
chr12	66451373		MIR6074(dist=33867),LLPH(dist=65476)	intergenic	HIVID	Zhao et al.2016	27703150	Tumor
chr3	24581553		MIR4792(dist=18627),RARB(dist=888201)	intergenic	HIVID	Zhao et al.2016	27703150	Tumor
chr2	89875191		MIR4436A(dist=763223),LOC654342(dist=1949518)	intergenic	HIVID	Zhao et al.2016	27703150	Tumor
chr2	89875123		MIR4436A(dist=763155),LOC654342(dist=1949586)	intergenic	HIVID	Zhao et al.2016	27703150	Tumor
chr2	89875053		MIR4436A(dist=763085),LOC654342(dist=1949656)	intergenic	HIVID	Zhao et al.2016	27703150	Tumor
chr2	89870435		MIR4436A(dist=758467),LOC654342(dist=1954274)	intergenic	HIVID	Zhao et al.2016	27703150	Tumor
chr2	89870205		MIR4436A(dist=758237),LOC654342(dist=1954504)	intergenic	HIVID	Zhao et al.2016	27703150	Tumor
chr3	177455247		LINC00578	ncRNA_intronic	HIVID	Zhao et al.2016	27703150	Tumor
chr20	30913489		KIF3B	intronic	HIVID	Zhao et al.2016	27703150	Tumor
chr6	160521834		IGF2R	intronic	HIVID	Zhao et al.2016	27703150	Tumor
chr6	160521756		IGF2R	intronic	HIVID	Zhao et al.2016	27703150	Tumor
chr6	2414077		GMDS-AS1(dist=252),C6orf195(dist=208895)	intergenic	HIVID	Zhao et al.2016	27703150	Tumor
chr9	45023267		FAM27C(dist=31775),FAM27A(dist=703762)	intergenic	HIVID	Zhao et al.2016	27703150	Tumor
chr9	45023224		FAM27C(dist=31732),FAM27A(dist=703805)	intergenic	HIVID	Zhao et al.2016	27703150	Tumor
chr6	133760225		EYA4	intronic	HIVID	Zhao et al.2016	27703150	Tumor
chr6	133760193		EYA4	intronic	HIVID	Zhao et al.2016	27703150	Tumor
chr1	172147583		DNM3	intronic	HIVID	Zhao et al.2016	27703150	Tumor
chr1	172147618		DNM3	intronic	HIVID	Zhao et al.2016	27703150	Tumor
chr4	49636421		CWH43(dist=572326),NONE(dist=NONE)	intergenic	HIVID	Zhao et al.2016	27703150	Tumor
chr13	40370561		COG6(dist=4759),LINC00332(dist=385385)	intergenic	HIVID	Zhao et al.2016	27703150	Tumor
chr13	40370515		COG6(dist=4713),LINC00332(dist=385431)	intergenic	HIVID	Zhao et al.2016	27703150	Tumor
chr8	88532016		CNBD1(dist=137061),DCAF4L2(dist=350955)	intergenic	HIVID	Zhao et al.2016	27703150	Tumor
chr5	1296878		TERT	promoter	HIVID	Zhao et al.2016	27703150	Tumor
chr5	1285441		TERT	intronic	HIVID	Zhao et al.2016	27703150	Tumor
chr2	14765877		FAM84A	promoter	HIVID	Zhao et al.2016	27703150	Tumor
chr5	119782263		FAM170A(dist=810746),PRR16(dist=17756)	intergenic	HIVID	Zhao et al.2016	27703150	Tumor
chr16	49739640		ZNF423	intronic	HIVID	Zhao et al.2016	27703150	Tumor
chr17	22254209		MTRNR2L1(dist=230218),NONE(dist=NONE)	intergenic	HIVID	Zhao et al.2016	27703150	Tumor
chr5	4940781		LOC101929153(dist=165803),LINC01020(dist=93691)	intergenic	HIVID	Zhao et al.2016	27703150	Tumor
chr5	37889795		GDNF-AS1(dist=13895),EGFLAM(dist=368716)	intergenic	HIVID	Zhao et al.2016	27703150	Tumor
chr9	90121151		DAPK1	intronic	HIVID	Zhao et al.2016	27703150	Tumor
chr5	1295200		TERT	promoter	HIVID	Zhao et al.2016	27703150	Tumor
chr16	76138094		TERF2IP(dist=446753),CNTNAP4(dist=173082)	intergenic	HIVID	Zhao et al.2016	27703150	Tumor
chr20	22732805		LOC101929685(dist=144650),SSTR4(dist=283252)	intergenic	HIVID	Zhao et al.2016	27703150	Tumor
chr9	110845034		KLF4(dist=592987),ACTL7B(dist=771835)	intergenic	HIVID	Zhao et al.2016	27703150	Tumor
chr5	1299451		TERT;MIR4457	promoter;downstream	HIVID	Zhao et al.2016	27703150	Tumor
chr8	19183206		SH2D4A	intronic	HIVID	Zhao et al.2016	27703150	Tumor
chr18	69701307		RP11-510D19.1(dist=455115),CBLN2(dist=502608)	intergenic	HIVID	Zhao et al.2016	27703150	Tumor
chr18	69701033		RP11-510D19.1(dist=454841),CBLN2(dist=502882)	intergenic	HIVID	Zhao et al.2016	27703150	Tumor
chr13	59211154		PCDH17(dist=908089),DIAPH3(dist=1028567)	intergenic	HIVID	Zhao et al.2016	27703150	Tumor
chr1	176427843		PAPPA2	promoter	HIVID	Zhao et al.2016	27703150	Tumor
chr8	46841845		NONE(dist=NONE),LINC00293(dist=910663)	intergenic	HIVID	Zhao et al.2016	27703150	Tumor
chr11	69572603		FGF19(dist=53497),FGF4(dist=15194)	intergenic	HIVID	Zhao et al.2016	27703150	Tumor
chr11	69571095		FGF19(dist=51989),FGF4(dist=16702)	intergenic	HIVID	Zhao et al.2016	27703150	Tumor
chr20	26232967		LOC284801(dist=43098),NONE(dist=NONE)	intergenic	HIVID	Zhao et al.2016	27703150	Tumor
chr2	76225713		GCFC2(dist=287602),LRRTM4(dist=749137)	intergenic	HIVID	Zhao et al.2016	27703150	Tumor
chr17	21900836		FLJ36000	promoter	HIVID	Zhao et al.2016	27703150	Tumor
chr9	139757122		EDF1	splicing	HIVID	Zhao et al.2016	27703150	Tumor

Chromosome	Integration site in host genome	Integration site in virus genome	Gene (distance, bp)	Regions	Methods	Author	PMID	Sample
chr9	139757024		EDF1	intronic	HIVID	Zhao et al.2016	27703150	Tumor
chr5	1295562		TERT	promoter	HIVID	Zhao et al.2016	27703150	Tumor
chr5	24909762		RP11-730N24.1(dist=69070),CDH9(dist=1970947)	intergenic	HIVID	Zhao et al.2016	27703150	Tumor
chr5	24898289		RP11-730N24.1(dist=57597),CDH9(dist=1982420)	intergenic	HIVID	Zhao et al.2016	27703150	Tumor
chr5	24958575		RP11-730N24.1(dist=117883),CDH9(dist=1922134)	intergenic	HIVID	Zhao et al.2016	27703150	Tumor
chr5	24958552		RP11-730N24.1(dist=117860),CDH9(dist=1922157)	intergenic	HIVID	Zhao et al.2016	27703150	Tumor
chr1	45015935		RNF220	intronic	HIVID	Zhao et al.2016	27703150	Tumor
chr16	46405933		NONE(dist=NONE),ANKRD26P1(dist=97316)	intergenic	HIVID	Zhao et al.2016	27703150	Tumor
chr6	124758415		NKAIN2	intronic	HIVID	Zhao et al.2016	27703150	Tumor
chr4	191044028		DUX4(dist=30586),NONE(dist=NONE)	intergenic	HIVID	Zhao et al.2016	27703150	Tumor
chr7	141334619		AGK	intronic	HIVID	Zhao et al.2016	27703150	Tumor
chr7	141334576		AGK	intronic	HIVID	Zhao et al.2016	27703150	Tumor
chr6	9398523		RP11-314C16.1(dist=612845),TFAP2A(dist=998393)	intergenic	HIVID	Zhao et al.2016	27703150	Tumor
chr8	55521743		RP1	promoter	HIVID	Zhao et al.2016	27703150	Tumor
chr21	48119870		PRMT2(dist=34834),NONE(dist=NONE)	intergenic	HIVID	Zhao et al.2016	27703150	Tumor
chr4	10067		NONE(dist=NONE),ZNF595(dist=43112)	intergenic	HIVID	Zhao et al.2016	27703150	Tumor
chr18	10030		NONE(dist=NONE),ROCK1P1(dist=99035)	intergenic	HIVID	Zhao et al.2016	27703150	Tumor
chr7	10188		NONE(dist=NONE),LOC100507642(dist=139530)	intergenic	HIVID	Zhao et al.2016	27703150	Tumor
chr12	95480		LOC100288778(dist=4217),FAM138D(dist=52466)	intergenic	HIVID	Zhao et al.2016	27703150	Tumor
chr4	191044233		DUX4(dist=30791),NONE(dist=NONE)	intergenic	HIVID	Zhao et al.2016	27703150	Tumor
chr4	191044214		DUX4(dist=30772),NONE(dist=NONE)	intergenic	HIVID	Zhao et al.2016	27703150	Tumor
chr4	191044183		DUX4(dist=30741),NONE(dist=NONE)	intergenic	HIVID	Zhao et al.2016	27703150	Tumor
chr4	191043761		DUX4(dist=30319),NONE(dist=NONE)	intergenic	HIVID	Zhao et al.2016	27703150	Tumor
chr15	102521343		DDX11L9;MIR6859-1,MIR6859-2,WASH3P	promoter;downstream	HIVID	Zhao et al.2016	27703150	Tumor
chr9	10052		DDX11L5;WASH1	promoter;downstream	HIVID	Zhao et al.2016	27703150	Tumor
chr7	152595063		ACTR3B(dist=42599),DPP6(dist=989356)	intergenic	HIVID	Zhao et al.2016	27703150	Tumor
chr8	43095264		HGSNAT(dist=37294),POTEA(dist=52321)	intergenic	HIVID	Zhao et al.2016	27703150	Tumor
chr1	158105622		RP11-404O13.1	ncRNA_intronic	HIVID	Zhao et al.2016	27703150	Tumor
chr6	57452280		PRIM2	intronic	HIVID	Zhao et al.2016	27703150	Tumor
chr6	156365122		NOX3(dist=588085),ARID1B(dist=733942)	intergenic	HIVID	Zhao et al.2016	27703150	Tumor
chr1	108910007		NBPF4(dist=123304),NBPF6(dist=82897)	intergenic	HIVID	Zhao et al.2016	27703150	Tumor
chr1	108901163		NBPF4(dist=114460),NBPF6(dist=91741)	intergenic	HIVID	Zhao et al.2016	27703150	Tumor
chr5	85569933		NBPF22P	promoter	HIVID	Zhao et al.2016	27703150	Tumor
chr2	149922922		LYPD6B	intronic	HIVID	Zhao et al.2016	27703150	Tumor
chr13	79503350		LINC00331(dist=89165),RBM26(dist=389653)	intergenic	HIVID	Zhao et al.2016	27703150	Tumor
chr6	58779400		GUSBP4(dist=491676),NONE(dist=NONE)	intergenic	HIVID	Zhao et al.2016	27703150	Tumor
chr6	58776343		GUSBP4(dist=488619),NONE(dist=NONE)	intergenic	HIVID	Zhao et al.2016	27703150	Tumor
chr11	30875696		MPPED2(dist=267766),DCDC5(dist=9454)	intergenic	HIVID	Zhao et al.2016	27703150	Tumor
chr11	30875667		MPPED2(dist=267737),DCDC5(dist=9483)	intergenic	HIVID	Zhao et al.2016	27703150	Tumor
chr5	1303724		TERT;MIR4457	promoter;downstream	HIVID	Zhao et al.2016	27703150	Tumor
chr5	1295485		TERT	promoter	HIVID	Zhao et al.2016	27703150	Tumor
chr4	158964287		RP11-364P22.1(dist=466984),FAM198B(dist=81445)	intergenic	HIVID	Zhao et al.2016	27703150	Tumor
chr4	158964150		RP11-364P22.1(dist=466847),FAM198B(dist=81582)	intergenic	HIVID	Zhao et al.2016	27703150	Tumor
chr17	16142730		PIGL	intronic	HIVID	Zhao et al.2016	27703150	Tumor
chr17	16144181		PIGL	intronic	HIVID	Zhao et al.2016	27703150	Tumor
chr5	166187727		NONE(dist=NONE),LOC102557615(dist=144500)	intergenic	HIVID	Zhao et al.2016	27703150	Tumor
chr14	85091440		NONE(dist=NONE),LINC00911(dist=768783)	intergenic	HIVID	Zhao et al.2016	27703150	Tumor
chr8	47343110		NONE(dist=NONE),LINC00293(dist=409398)	intergenic	HIVID	Zhao et al.2016	27703150	Tumor
chr7	100632468		MUC12	intronic	HIVID	Zhao et al.2016	27703150	Tumor
chr18	19507779		MIB1(dist=56861),GATA6-AS1(dist=239080)	intergenic	HIVID	Zhao et al.2016	27703150	Tumor
chr10	12997828		CCDC3	intronic	HIVID	Zhao et al.2016	27703150	Tumor
chr7	43622737		STK17A	UTR5	HIVID	Zhao et al.2016	27703150	Tumor
chr7	43622603		STK17A	promoter	HIVID	Zhao et al.2016	27703150	Tumor
chr8	121856922		SNTB1(dist=32613),HAS2(dist=768349)	intergenic	HIVID	Zhao et al.2016	27703150	Tumor
chr8	138689645		NONE(dist=NONE),FAM135B(dist=452621)	intergenic	HIVID	Zhao et al.2016	27703150	Tumor
chr8	126983437		LINC00861(dist=19996),FAM84B(dist=581246)	intergenic	HIVID	Zhao et al.2016	27703150	Tumor
chr8	126982554		LINC00861(dist=19113),FAM84B(dist=582129)	intergenic	HIVID	Zhao et al.2016	27703150	Tumor
chr19	36212843		KMT2B	intronic	HIVID	Zhao et al.2016	27703150	Tumor
chr19	42745162		GSK3A	intronic	HIVID	Zhao et al.2016	27703150	Tumor
chr8	115439171		CSMD3(dist=989929),TRPS1(dist=981553)	intergenic	HIVID	Zhao et al.2016	27703150	Tumor
chr8	69666834		C8orf34	intronic	HIVID	Zhao et al.2016	27703150	Tumor
chr1	94509052		ABCA4	intronic	HIVID	Zhao et al.2016	27703150	Tumor
chr1	94509081		ABCA4	intronic	HIVID	Zhao et al.2016	27703150	Tumor
chr19	12486581		ZNF442(dist=10106),ZNF799(dist=14247)	intergenic	HIVID	Zhao et al.2016	27703150	Tumor
chr19	24208017		ZNF254	promoter	HIVID	Zhao et al.2016	27703150	Tumor
chr1	29559		WASH7P;FAM138A,FAM138F	promoter;downstream	HIVID	Zhao et al.2016	27703150	Tumor
chr19	71166		WASH5P;FAM138A,FAM138F	promoter;downstream	HIVID	Zhao et al.2016	27703150	Tumor
chr19	61141		WASH5P	ncRNA_exonic	HIVID	Zhao et al.2016	27703150	Tumor
chr2	114351472		WASH2P	ncRNA_intronic	HIVID	Zhao et al.2016	27703150	Tumor
chr2	114341481		WASH2P	ncRNA_exonic	HIVID	Zhao et al.2016	27703150	Tumor
chr9	29337		WASH1	intronic	HIVID	Zhao et al.2016	27703150	Tumor
chr9	138866309		UBAC1(dist=13083),NACC2(dist=32074)	intergenic	HIVID	Zhao et al.2016	27703150	Tumor
chr15	31385365		TRPM1	intronic	HIVID	Zhao et al.2016	27703150	Tumor
chr1	8001999		TNFRSF9	intronic	HIVID	Zhao et al.2016	27703150	Tumor
chr5	1272712		TERT	intronic	HIVID	Zhao et al.2016	27703150	Tumor
chr5	1273245		TERT	intronic	HIVID	Zhao et al.2016	27703150	Tumor
chr5	1273781		TERT	intronic	HIVID	Zhao et al.2016	27703150	Tumor
chr5	1273636		TERT	intronic	HIVID	Zhao et al.2016	27703150	Tumor
chr5	1272678		TERT	intronic	HIVID	Zhao et al.2016	27703150	Tumor
chr5	1273293		TERT	intronic	HIVID	Zhao et al.2016	27703150	Tumor
chr5	1272746		TERT	intronic	HIVID	Zhao et al.2016	27703150	Tumor
chr5	1273727		TERT	intronic	HIVID	Zhao et al.2016	27703150	Tumor
chr5	1273872		TERT	intronic	HIVID	Zhao et al.2016	27703150	Tumor
chr5	1272795		TERT	intronic	HIVID	Zhao et al.2016	27703150	Tumor
chr5	1272655		TERT	intronic	HIVID	Zhao et al.2016	27703150	Tumor
chr5	1272686		TERT	intronic	HIVID	Zhao et al.2016	27703150	Tumor
chr5	1273200		TERT	intronic	HIVID	Zhao et al.2016	27703150	Tumor
chr5	1272901		TERT	intronic	HIVID	Zhao et al.2016	27703150	Tumor
chr5	1272759		TERT	intronic	HIVID	Zhao et al.2016	27703150	Tumor
chr11	78996184		TENM4	intronic	HIVID	Zhao et al.2016	27703150	Tumor
chr2	85367885		TCF7L1	intronic	HIVID	Zhao et al.2016	27703150	Tumor
chr14	91499559		RPS6KA5	intronic	HIVID	Zhao et al.2016	27703150	Tumor
chr16	73981492		RP11-140I24.1(dist=526197),AC009120.4(dist=244799)	intergenic	HIVID	Zhao et al.2016	27703150	Tumor
chr16	73981410		RP11-140I24.1(dist=526115),AC009120.4(dist=244881)	intergenic	HIVID	Zhao et al.2016	27703150	Tumor

Chromosome	Integration site in host genome	Integration site in virus genome	Gene (distance, bp)	Regions	Methods	Author	PMID	Sample
chr16	73981383		RP11-140I24.1(dist=526088),AC009120.4(dist=244908)	intergenic	HIVID	Zhao et al.2016	27703150	Tumor
chr16	73981369		RP11-140I24.1(dist=526074),AC009120.4(dist=244922)	intergenic	HIVID	Zhao et al.2016	27703150	Tumor
chr1	167635166		RCSD1	intronic	HIVID	Zhao et al.2016	27703150	Tumor
chr21	48119752		PRMT2(dist=34716),NONE(dist=NONE)	intergenic	HIVID	Zhao et al.2016	27703150	Tumor
chr22	50745111		PLXNB2	intronic	HIVID	Zhao et al.2016	27703150	Tumor
chr18	78016319		PARD6G(dist=10922),NONE(dist=NONE)	intergenic	HIVID	Zhao et al.2016	27703150	Tumor
chr18	78016288		PARD6G(dist=10891),NONE(dist=NONE)	intergenic	HIVID	Zhao et al.2016	27703150	Tumor
chr18	78016187		PARD6G(dist=10790),NONE(dist=NONE)	intergenic	HIVID	Zhao et al.2016	27703150	Tumor
chr1	120543902		NOTCH2	intronic	HIVID	Zhao et al.2016	27703150	Tumor
chr18	10052		NONE(dist=NONE),ROCK1P1(dist=99013)	intergenic	HIVID	Zhao et al.2016	27703150	Tumor
chr18	18520257		NONE(dist=NONE),ROCK1(dist=9446)	intergenic	HIVID	Zhao et al.2016	27703150	Tumor
chr18	18520343		NONE(dist=NONE),ROCK1(dist=9360)	intergenic	HIVID	Zhao et al.2016	27703150	Tumor
chr5	11668		NONE(dist=NONE),PLEKHG4B(dist=128705)	intergenic	HIVID	Zhao et al.2016	27703150	Tumor
chr5	11686		NONE(dist=NONE),PLEKHG4B(dist=128687)	intergenic	HIVID	Zhao et al.2016	27703150	Tumor
chr5	11704		NONE(dist=NONE),PLEKHG4B(dist=128669)	intergenic	HIVID	Zhao et al.2016	27703150	Tumor
chr7	10086		NONE(dist=NONE),LOC100507642(dist=139632)	intergenic	HIVID	Zhao et al.2016	27703150	Tumor
chr7	10176		NONE(dist=NONE),LOC100507642(dist=139542)	intergenic	HIVID	Zhao et al.2016	27703150	Tumor
chr12	74128		NONE(dist=NONE),LOC100288778(dist=13856)	intergenic	HIVID	Zhao et al.2016	27703150	Tumor
chr1	6612603		NOL9	intronic	HIVID	Zhao et al.2016	27703150	Tumor
chr1	145277490		NBPF20,NBPF9,NOTCH2NL	intronic	HIVID	Zhao et al.2016	27703150	Tumor
chr1	145277489		NBPF20,NBPF9,NOTCH2NL	intronic	HIVID	Zhao et al.2016	27703150	Tumor
chr16	69222		MIR6859-1,MIR6859-2;DDX11L10	promoter;downstream	HIVID	Zhao et al.2016	27703150	Tumor
chr3	185025452		MAP3K13	intronic	HIVID	Zhao et al.2016	27703150	Tumor
chr2	243152578		LOC728323(dist=50109),NONE(dist=NONE)	intergenic	HIVID	Zhao et al.2016	27703150	Tumor
chr2	243152477		LOC728323(dist=50008),NONE(dist=NONE)	intergenic	HIVID	Zhao et al.2016	27703150	Tumor
chr12	95627		LOC100288778(dist=4364),FAM138D(dist=52319)	intergenic	HIVID	Zhao et al.2016	27703150	Tumor
chr12	95435		LOC100288778(dist=4172),FAM138D(dist=52511)	intergenic	HIVID	Zhao et al.2016	27703150	Tumor
chr12	86173		LOC100288778	promoter	HIVID	Zhao et al.2016	27703150	Tumor
chr1	235136980		LINC01132(dist=269590),TOMM20(dist=135678)	intergenic	HIVID	Zhao et al.2016	27703150	Tumor
chr1	200314024		LINC00862	ncRNA_intronic	HIVID	Zhao et al.2016	27703150	Tumor
chr20	62918272		LINC00266-1	promoter	HIVID	Zhao et al.2016	27703150	Tumor
chr9	38449508		IGFBPL1(dist=25064),FAM95C(dist=91056)	intergenic	HIVID	Zhao et al.2016	27703150	Tumor
chr9	38449398		IGFBPL1(dist=24954),FAM95C(dist=91166)	intergenic	HIVID	Zhao et al.2016	27703150	Tumor
chr16	28020247		GSG1L	intronic	HIVID	Zhao et al.2016	27703150	Tumor
chr6	133593952		EYA4	intronic	HIVID	Zhao et al.2016	27703150	Tumor
chr10	135524710		DUX4L7(dist=26252),NONE(dist=NONE)	intergenic	HIVID	Zhao et al.2016	27703150	Tumor
chr10	135524680		DUX4L7(dist=26222),NONE(dist=NONE)	intergenic	HIVID	Zhao et al.2016	27703150	Tumor
chr10	135524592		DUX4L7(dist=26134),NONE(dist=NONE)	intergenic	HIVID	Zhao et al.2016	27703150	Tumor
chr10	135524561		DUX4L7(dist=26103),NONE(dist=NONE)	intergenic	HIVID	Zhao et al.2016	27703150	Tumor
chr4	191043857		DUX4(dist=30415),NONE(dist=NONE)	intergenic	HIVID	Zhao et al.2016	27703150	Tumor
chr15	102521280		DDX11L9;MIR6859-1,MIR6859-2,WASH3P	promoter;downstream	HIVID	Zhao et al.2016	27703150	Tumor
chr9	10076		DDX11L5;WASH1	promoter;downstream	HIVID	Zhao et al.2016	27703150	Tumor
chr9	10142		DDX11L5;WASH1	promoter;downstream	HIVID	Zhao et al.2016	27703150	Tumor
chr9	10053		DDX11L5;WASH1	promoter;downstream	HIVID	Zhao et al.2016	27703150	Tumor
chr9	10153		DDX11L5;WASH1	promoter;downstream	HIVID	Zhao et al.2016	27703150	Tumor
chr1	10107		DDX11L1;MIR6859-1,MIR6859-2,WASH7P	promoter;downstream	HIVID	Zhao et al.2016	27703150	Tumor
chr13	95132330		DCT	promoter	HIVID	Zhao et al.2016	27703150	Tumor
chr21	47554615		COL6A2(dist=1852),FTCD(dist=1561)	intergenic	HIVID	Zhao et al.2016	27703150	Tumor
chr21	47554573		COL6A2(dist=1810),FTCD(dist=1603)	intergenic	HIVID	Zhao et al.2016	27703150	Tumor
chr21	47554500		COL6A2(dist=1737),FTCD(dist=1676)	intergenic	HIVID	Zhao et al.2016	27703150	Tumor
chr21	47554465		COL6A2(dist=1702),FTCD(dist=1711)	intergenic	HIVID	Zhao et al.2016	27703150	Tumor
chr19	59098046		CENPBD1P1(dist=2284),NONE(dist=NONE)	intergenic	HIVID	Zhao et al.2016	27703150	Tumor
chr19	59097932		CENPBD1P1(dist=2170),NONE(dist=NONE)	intergenic	HIVID	Zhao et al.2016	27703150	Tumor
chr1	22323755		CELA3A;CELA3B	promoter;downstream	HIVID	Zhao et al.2016	27703150	Tumor
chr15	39159805		C15orf53(dist=167566),C15orf54(dist=383080)	intergenic	HIVID	Zhao et al.2016	27703150	Tumor
chr15	39141822		C15orf53(dist=149583),C15orf54(dist=401063)	intergenic	HIVID	Zhao et al.2016	27703150	Tumor
chr1	151009963		BNIPL	UTR5	HIVID	Zhao et al.2016	27703150	Tumor
chr1	151009872		BNIPL	UTR5	HIVID	Zhao et al.2016	27703150	Tumor
chr19	36066754		ATP4A(dist=12194),HAUS5(dist=36892)	intergenic	HIVID	Zhao et al.2016	27703150	Tumor
chr19	36066691		ATP4A(dist=12131),HAUS5(dist=36955)	intergenic	HIVID	Zhao et al.2016	27703150	Tumor
chr2	133039237		ANKRD30BL(dist=23695),GPR39(dist=134910)	intergenic	HIVID	Zhao et al.2016	27703150	Tumor
chr5	1299849		TERT;MIR4457	promoter;downstream	HIVID	Zhao et al.2016	27703150	Tumor
chr5	39137075		FYB	intronic	HIVID	Zhao et al.2016	27703150	Tumor
chr1	192601042		RGS13	promoter	HIVID	Zhao et al.2016	27703150	Tumor
chr6	19457128		MIR548A1(dist=885017),ID4(dist=380473)	intergenic	HIVID	Zhao et al.2016	27703150	Tumor
chr9	114187443		KIAA0368	intronic	HIVID	Zhao et al.2016	27703150	Tumor
chr9	114186857		KIAA0368	intronic	HIVID	Zhao et al.2016	27703150	Tumor
chr17	62183303		ERN1	intronic	HIVID	Zhao et al.2016	27703150	Tumor
chr1	193194293		CDC73	intronic	HIVID	Zhao et al.2016	27703150	Tumor
chr19	30293497		CCNE1	promoter	HIVID	Zhao et al.2016	27703150	Tumor
chr19	30295159		CCNE1	promoter	HIVID	Zhao et al.2016	27703150	Tumor
chr13	76955967		C13orf45(dist=498019),KCTD12(dist=498337)	intergenic	HIVID	Zhao et al.2016	27703150	Tumor
chr7	88969000		ZNF804B(dist=2654),STEAP2-AS1(dist=354658)	intergenic	HIVID	Zhao et al.2016	27703150	Tumor
chr5	103170397		NUDT12(dist=271907),RAB9BP1(dist=1264778)	intergenic	HIVID	Zhao et al.2016	27703150	Tumor
chr14	49278930		LINC00648(dist=1014713),RPS29(dist=764460)	intergenic	HIVID	Zhao et al.2016	27703150	Tumor
chr3	50228217		GNAT1;SEMA3F	promoter;downstream	HIVID	Zhao et al.2016	27703150	Tumor
chr3	50228213		GNAT1;SEMA3F	promoter;downstream	HIVID	Zhao et al.2016	27703150	Tumor
chr13	36621174		DCLK1	intronic	HIVID	Zhao et al.2016	27703150	Tumor
chr19	30280428		C19orf12(dist=73732),CCNE1(dist=22473)	intergenic	HIVID	Zhao et al.2016	27703150	Tumor
chr2	204210287		ABI2	intronic	HIVID	Zhao et al.2016	27703150	Tumor
chr13	61789407		MIR3169(dist=15393),PCDH20(dist=194412)	intergenic	HIVID	Zhao et al.2016	27703150	Tumor
chr1	210062365		DIEXF(dist=31455),SYT14(dist=49154)	intergenic	HIVID	Zhao et al.2016	27703150	Tumor
chr1	210062330		DIEXF(dist=31420),SYT14(dist=49189)	intergenic	HIVID	Zhao et al.2016	27703150	Tumor
chr18	18517801		NONE(dist=NONE),ROCK1(dist=11902)	intergenic	HIVID	Zhao et al.2016	27703150	Tumor
chr7	117823227		LSM8	promoter	HIVID	Zhao et al.2016	27703150	Tumor
chr7	117823235		LSM8	promoter	HIVID	Zhao et al.2016	27703150	Tumor
chr21	9753889		NONE(dist=NONE),MIR3648(dist=71943)	intergenic	HIVID	Zhao et al.2016	27703150	Tumor
chr7	147176204		MIR548I4	ncRNA_intronic	HIVID	Zhao et al.2016	27703150	Tumor
chr4	341863		ZNF141	intronic	HIVID	Zhao et al.2016	27703150	Tumor
chr1	216389548		USH2A	intronic	HIVID	Zhao et al.2016	27703150	Tumor
chr5	38993315		RICTOR	intronic	HIVID	Zhao et al.2016	27703150	Tumor
chr9	66971213		PTGER4P2-CDK2AP2P2(dist=468183),RP11-381O7.3(dist=4616	intergenic	HIVID	Zhao et al.2016	27703150	Tumor
chr7	135271152		NUP205	intronic	HIVID	Zhao et al.2016	27703150	Tumor
chr7	117823227		LSM8	promoter	HIVID	Zhao et al.2016	27703150	Tumor

Supplementary Table S8 Continued

Chromosome	Integration site in host genome	Integration site in virus genome	Gene (distance, bp)	Regions	Methods	Author	PMID	Sample
chr6	154619560		IPCEF1	intronic	HIVID	Zhao et al.2016	27703150	Tumor
chr1	231435904		GNPAT(dist=22185),EXOC8(dist=32578)	intergenic	HIVID	Zhao et al.2016	27703150	Tumor
chr1	10270		DDX11L1;MIR6859-1,MIR6859-2,WASH7P	promoter;downstream	HIVID	Zhao et al.2016	27703150	Tumor
chr9	77964371		OSTF1(dist=202257),MIR548H3(dist=154141)	intergenic	HIVID	Zhao et al.2016	27703150	Tumor
chr16	46406787		NONE(dist=NONE),ANKRD26P1(dist=96462)	intergenic	HIVID	Zhao et al.2016	27703150	Tumor
chr16	46394867		NONE(dist=NONE),ANKRD26P1(dist=108382)	intergenic	HIVID	Zhao et al.2016	27703150	Tumor
chr4	178351553		NEIL3(dist=67461),AGA(dist=376)	intergenic	HIVID	Zhao et al.2016	27703150	Tumor
chr17	22257964		MTRNR2L1(dist=233973),NONE(dist=NONE)	intergenic	HIVID	Zhao et al.2016	27703150	Tumor
chr3	192559561		MB21D2	intronic	HIVID	Zhao et al.2016	27703150	Tumor
chr3	192559657		MB21D2	intronic	HIVID	Zhao et al.2016	27703150	Tumor
chr2	237981113		ACKR3(dist=490119),COPS8(dist=12971)	intergenic	HIVID	Zhao et al.2016	27703150	Tumor
chr13	114865110		RASA3	intronic	HIVID	Zhao et al.2016	27703150	Tumor
chr8	43354050		POTEA(dist=135722),NONE(dist=NONE)	intergenic	HIVID	Zhao et al.2016	27703150	Tumor
chr6	68732744		NONE(dist=NONE),BAI3(dist=612888)	intergenic	HIVID	Zhao et al.2016	27703150	Tumor
chr4	39411536		MIR1273H	ncRNA_intronic	HIVID	Zhao et al.2016	27703150	Tumor
chr15	26569501		LINC00929(dist=191317),GABRB3(dist=219193)	intergenic	HIVID	Zhao et al.2016	27703150	Tumor
chr14	37169652		SLC25A21	intronic	HIVID	Zhao et al.2016	27703150	Tumor
chr14	32025344		NUBPL	promoter	HIVID	Zhao et al.2016	27703150	Tumor
chr14	36915477		MBIP(dist=125595),SFTA3(dist=27017)	intergenic	HIVID	Zhao et al.2016	27703150	Tumor
chr17	48986568		TOB1-AS1(dist=40836),SPAG9(dist=52967)	intergenic	HIVID	Zhao et al.2016	27703150	Tumor
chr5	1307646		TERT(dist=12484),MIR4457(dist=1779)	intergenic	HIVID	Zhao et al.2016	27703150	Tumor
chr5	1295219		TERT	promoter	HIVID	Zhao et al.2016	27703150	Tumor
chr11	1677246		MOB2	intronic	HIVID	Zhao et al.2016	27703150	Tumor
chr12	66451463		MIR6074(dist=33957),LLPH(dist=65386)	intergenic	HIVID	Zhao et al.2016	27703150	Tumor
chr12	66451373		MIR6074(dist=33867),LLPH(dist=65476)	intergenic	HIVID	Zhao et al.2016	27703150	Tumor
chr17	13654719		HS3ST3A1(dist=149475),CDRT15P1(dist=273096)	intergenic	HIVID	Zhao et al.2016	27703150	Tumor
chr8	65095026		RP11-579E24.1(dist=396972),LINC00966(dist=190749)	intergenic	HIVID	Zhao et al.2016	27703150	Tumor
chr17	22261574		MTRNR2L1(dist=237583),NONE(dist=NONE)	intergenic	HIVID	Zhao et al.2016	27703150	Tumor
chr17	22258349		MTRNR2L1(dist=234358),NONE(dist=NONE)	intergenic	HIVID	Zhao et al.2016	27703150	Tumor
chr3	182607683		ATP11B	intronic	HIVID	Zhao et al.2016	27703150	Tumor
chr21	28737622		MIR5009	ncRNA_intronic	HIVID	Zhao et al.2016	27703150	Tumor
chr19	36213567		KMT2B	exonic	HIVID	Zhao et al.2016	27703150	Tumor
chr19	36213549		KMT2B	exonic	HIVID	Zhao et al.2016	27703150	Tumor
chr19	36213517		KMT2B	exonic	HIVID	Zhao et al.2016	27703150	Tumor
chr18	51364404		DCC(dist=302131),MBD2(dist=313567)	intergenic	HIVID	Zhao et al.2016	27703150	Tumor
chr5	1076688		SLC12A7	intronic	HIVID	Zhao et al.2016	27703150	Tumor
chr12	95479		LOC100288778(dist=4216),FAM138D(dist=52467)	intergenic	HIVID	Zhao et al.2016	27703150	Tumor
chr15	88161993		LINC00052(dist=39076),NTRK3(dist=257995)	intergenic	HIVID	Zhao et al.2016	27703150	Tumor
chr4	77673383		SHROOM3	intronic	HIVID	Zhao et al.2016	27703150	Tumor
chr1	116335823		CASQ2(dist=24397),NHLH2(dist=43176)	intergenic	HIVID	Zhao et al.2016	27703150	Tumor
chr2	237981113		ACKR3(dist=490119),COPS8(dist=12971)	intergenic	HIVID	Zhao et al.2016	27703150	Tumor
chr5	1293411		TERT	intronic	HIVID	Zhao et al.2016	27703150	Tumor
chr11	92081866		FAT3	promoter	HIVID	Zhao et al.2016	27703150	Tumor
chr7	61374390		NONE(dist=NONE),ZNF733P(dist=1377280)	intergenic	HIVID	Zhao et al.2016	27703150	Tumor
chr7	61374442		NONE(dist=NONE),ZNF733P(dist=1377228)	intergenic	HIVID	Zhao et al.2016	27703150	Tumor
chr5	49451693		NONE(dist=NONE),EMB(dist=240338)	intergenic	HIVID	Zhao et al.2016	27703150	Tumor
chr15	36586842		MIR4510(dist=367718),C15orf41(dist=284970)	intergenic	HIVID	Zhao et al.2016	27703150	Tumor
chr2	133028361		ANKRD30BL(dist=12819),GPR39(dist=145786)	intergenic	HIVID	Zhao et al.2016	27703150	Tumor
chr2	1314853		SNTG2	intronic	HIVID	Zhao et al.2016	27703150	Tumor
chr18	18519894		NONE(dist=NONE),ROCK1(dist=9809)	intergenic	HIVID	Zhao et al.2016	27703150	Tumor
chr18	18512806		NONE(dist=NONE),ROCK1(dist=16897)	intergenic	HIVID	Zhao et al.2016	27703150	Tumor
chr18	18517552		NONE(dist=NONE),ROCK1(dist=12151)	intergenic	HIVID	Zhao et al.2016	27703150	Tumor
chr18	18517615		NONE(dist=NONE),ROCK1(dist=12088)	intergenic	HIVID	Zhao et al.2016	27703150	Tumor
chr18	18519196		NONE(dist=NONE),ROCK1(dist=10507)	intergenic	HIVID	Zhao et al.2016	27703150	Tumor
chr5	9713406		LOC285692	ncRNA_intronic	HIVID	Zhao et al.2016	27703150	Tumor
chr4	143464444		INPP4B	intronic	HIVID	Zhao et al.2016	27703150	Tumor
chr20	11909683		BTBD3(dist=2440),RP5-1069C8.2(dist=936169)	intergenic	HIVID	Zhao et al.2016	27703150	Tumor
chr2	92309684		ACTR3BP2(dist=179188),NONE(dist=NONE)	intergenic	HIVID	Zhao et al.2016	27703150	Tumor
chr8	87268070		SLC7A13(dist=25466),WWP1(dist=86924)	intergenic	HIVID	Zhao et al.2016	27703150	Tumor
chr16	46405392		NONE(dist=NONE),ANKRD26P1(dist=97857)	intergenic	HIVID	Zhao et al.2016	27703150	Tumor
chr16	46393472		NONE(dist=NONE),ANKRD26P1(dist=109777)	intergenic	HIVID	Zhao et al.2016	27703150	Tumor
chr8	75164910		JPH1	intronic	HIVID	Zhao et al.2016	27703150	Tumor
chr1	221305879		HLX(dist=247479),C1orf140(dist=197391)	intergenic	HIVID	Zhao et al.2016	27703150	Tumor
chr7	52310223		COBL(dist=925708),POM121L12(dist=793126)	intergenic	HIVID	Zhao et al.2016	27703150	Tumor
chr8	38195808		WHSC1L1	intronic	HIVID	Zhao et al.2016	27703150	Tumor
chr5	1295169		TERT	promoter	HIVID	Zhao et al.2016	27703150	Tumor
chr8	38006801		STAR	intronic	HIVID	Zhao et al.2016	27703150	Tumor
chr8	143316463		TSNARE1	intronic	HIVID	Zhao et al.2016	27703150	Tumor
chr5	1296577		TERT	promoter	HIVID	Zhao et al.2016	27703150	Tumor
chr5	1295231		TERT	promoter	HIVID	Zhao et al.2016	27703150	Tumor
chr5	122517554		PRDM6	intronic	HIVID	Zhao et al.2016	27703150	Tumor
chr14	38345961		FOXA1(dist=281636),SSTR1(dist=331243)	intergenic	HIVID	Zhao et al.2016	27703150	Tumor
chr14	38345155		FOXA1(dist=280830),SSTR1(dist=332049)	intergenic	HIVID	Zhao et al.2016	27703150	Tumor
chr7	136728644		AC009264.1	ncRNA_intronic	HIVID	Zhao et al.2016	27703150	Tumor
chr14	61135190		SIX1(dist=19035),SIX4(dist=41066)	intergenic	HIVID	Zhao et al.2016	27703150	Tumor
chr10	77234888		RP11-399K21.14(dist=63806),C10orf11(dist=307631)	intergenic	HIVID	Zhao et al.2016	27703150	Tumor
chr8	43835509		POTEA(dist=617181),NONE(dist=NONE)	intergenic	HIVID	Zhao et al.2016	27703150	Tumor
chr8	43822429		POTEA(dist=604101),NONE(dist=NONE)	intergenic	HIVID	Zhao et al.2016	27703150	Tumor
chr14	61250918		MNAT1	intronic	HIVID	Zhao et al.2016	27703150	Tumor
chr14	61250828		MNAT1	intronic	HIVID	Zhao et al.2016	27703150	Tumor
chr21	9827166		MIR3687(dist=903),TEKT4P2(dist=80023)	intergenic	HIVID	Zhao et al.2016	27703150	Tumor
chr21	9825797		MIR3648,MIR3687	promoter	HIVID	Zhao et al.2016	27703150	Tumor
chr21	9825881		MIR3648	ncRNA_exonic	HIVID	Zhao et al.2016	27703150	Tumor
chr21	9825869		MIR3648	ncRNA_exonic	HIVID	Zhao et al.2016	27703150	Tumor
chr21	9825839		MIR3648	ncRNA_exonic	HIVID	Zhao et al.2016	27703150	Tumor
chr10	76649342		KAT6B	intronic	HIVID	Zhao et al.2016	27703150	Tumor
chr3	82090972		GBE1(dist=280022),NONE(dist=NONE)	intergenic	HIVID	Zhao et al.2016	27703150	Tumor
chr3	82090923		GBE1(dist=279973),NONE(dist=NONE)	intergenic	HIVID	Zhao et al.2016	27703150	Tumor
chr19	36212652		KMT2B	exonic	HIVID	Zhao et al.2016	27703150	Tumor
chr1	239403480		LINC01139(dist=754163),CHRM3(dist=388893)	intergenic	HIVID	Zhao et al.2016	27703150	Tumor
chr19	36212885		KMT2B	intronic	HIVID	Zhao et al.2016	27703150	Tumor
chr19	36212935		KMT2B	intronic	HIVID	Zhao et al.2016	27703150	Tumor
chr5	1295409		TERT	promoter	HIVID	Zhao et al.2016	27703150	Tumor
chr18	77773066		TXNL4A(dist=24534),RBFA(dist=21280)	intergenic	HIVID	Zhao et al.2016	27703150	Tumor

**Chromosome**	**Integration site in host genome**	**Integration site in virus genome**	**Gene (distance, bp)**	**Regions**	**Methods**	**Author**	**PMID**	**Sample**
chr5	1295656		TERT	promoter	HIVID	Zhao et al.2016	27703150	Tumor
chr5	1296187		TERT	promoter	HIVID	Zhao et al.2016	27703150	Tumor
chr7	105741915		SYPL1	intronic	HIVID	Zhao et al.2016	27703150	Tumor
chr7	105741884		SYPL1	intronic	HIVID	Zhao et al.2016	27703150	Tumor
chr9	65567689		SPATA31A5(dist=58079),PTGER4P2-CDK2AP2P2(dist=926580)	intergenic	HIVID	Zhao et al.2016	27703150	Tumor
chr9	41442640		SPATA31A4(dist=115243),SPATA31A5(dist=58039)	intergenic	HIVID	Zhao et al.2016	27703150	Tumor
chr12	127650453		RP11-575F12.1(dist=105511),RP11-955H22.3(dist=158247)	intergenic	HIVID	Zhao et al.2016	27703150	Tumor
chr9	66555993		PTGER4P2-CDK2AP2P2(dist=52963),RP11-381O7.3(dist=46138[illegible]	intergenic	HIVID	Zhao et al.2016	27703150	Tumor
chr17	31149571		MYO1D	intronic	HIVID	Zhao et al.2016	27703150	Tumor
chr17	31149488		MYO1D	intronic	HIVID	Zhao et al.2016	27703150	Tumor
chr17	31149508		MYO1D	intronic	HIVID	Zhao et al.2016	27703150	Tumor
chr9	39826606		LOC653501(dist=362080),SPATA31A1(dist=58369)	intergenic	HIVID	Zhao et al.2016	27703150	Tumor
chr9	41952313		LOC653501(dist=342769),GLIDR(dist=86)	intergenic	HIVID	Zhao et al.2016	27703150	Tumor
chr9	44404521		LINC01189(dist=13105),FAM27C(dist=585715)	intergenic	HIVID	Zhao et al.2016	27703150	Tumor
chr2	33141401		LINC00486	ncRNA_intronic	HIVID	Zhao et al.2016	27703150	Tumor
chr2	33141363		LINC00486	ncRNA_intronic	HIVID	Zhao et al.2016	27703150	Tumor
chr2	33141419		LINC00486	ncRNA_intronic	HIVID	Zhao et al.2016	27703150	Tumor
chr2	33141451		LINC00486	ncRNA_intronic	HIVID	Zhao et al.2016	27703150	Tumor
chr9	42021678		KGFLP2	promoter	HIVID	Zhao et al.2016	27703150	Tumor
chr9	46685365		KGFLP1	promoter	HIVID	Zhao et al.2016	27703150	Tumor
chr9	79186951		GCNT1(dist=64619),PRUNE2(dist=39341)	intergenic	HIVID	Zhao et al.2016	27703150	Tumor
chr3	2895675		CNTN4	intronic	HIVID	Zhao et al.2016	27703150	Tumor
chr3	53846536		CACNA1D(dist=44),CHDH(dist=3788)	intergenic	HIVID	Zhao et al.2016	27703150	Tumor
chr3	53846457		CACNA1D	UTR3	HIVID	Zhao et al.2016	27703150	Tumor
chr4	126031386		ANKRD50(dist=397499),FAT4(dist=206181)	intergenic	HIVID	Zhao et al.2016	27703150	Tumor
chr5	1296546		TERT	promoter	HIVID	Zhao et al.2016	27703150	Tumor
chr5	1296565		TERT	promoter	HIVID	Zhao et al.2016	27703150	Tumor
chr17	75158339		SEC14L1	intronic	HIVID	Zhao et al.2016	27703150	Tumor
chr12	127650988		RP11-575F12.1(dist=106046),RP11-955H22.3(dist=157712)	intergenic	HIVID	Zhao et al.2016	27703150	Tumor
chr12	127650932		RP11-575F12.1(dist=105990),RP11-955H22.3(dist=157768)	intergenic	HIVID	Zhao et al.2016	27703150	Tumor
chr12	127650866		RP11-575F12.1(dist=105924),RP11-955H22.3(dist=157834)	intergenic	HIVID	Zhao et al.2016	27703150	Tumor
chr8	10709002		PINX1(dist=11593),XKR6(dist=44655)	intergenic	HIVID	Zhao et al.2016	27703150	Tumor
chr10	42385241		NONE(dist=NONE),LOC441666(dist=442073)	intergenic	HIVID	Zhao et al.2016	27703150	Tumor
chr9	28624872		LINGO2	intronic	HIVID	Zhao et al.2016	27703150	Tumor
chr21	15457319		ANKRD20A11P(dist=104554),LIPI(dist=23816)	intergenic	HIVID	Zhao et al.2016	27703150	Tumor
chr5	1295256		TERT	promoter	HIVID	Zhao et al.2016	27703150	Tumor
chr19	5632865		SAFB	intronic	HIVID	Zhao et al.2016	27703150	Tumor
chr5	11496		NONE(dist=NONE),PLEKHG4B(dist=128877)	intergenic	HIVID	Zhao et al.2016	27703150	Tumor
chr5	11562		NONE(dist=NONE),PLEKHG4B(dist=128811)	intergenic	HIVID	Zhao et al.2016	27703150	Tumor
chr4	191044074		DUX4(dist=30632),NONE(dist=NONE)	intergenic	HIVID	Zhao et al.2016	27703150	Tumor
chr8	68319645		ARFGEF1(dist=63733),CPA6(dist=14760)	intergenic	HIVID	Zhao et al.2016	27703150	Tumor
chr5	1295800		TERT	promoter	HIVID	Zhao et al.2016	27703150	Tumor
chr16	49919812		ZNF423(dist=27982),CNEP1R1(dist=139305)	intergenic	HIVID	Zhao et al.2016	27703150	Tumor
chr3	167136194		ZBBX(dist=38109),SERPINI2(dist=23383)	intergenic	HIVID	Zhao et al.2016	27703150	Tumor
chr5	1298519		TERT	promoter	HIVID	Zhao et al.2016	27703150	Tumor
chr5	1296160		TERT	promoter	HIVID	Zhao et al.2016	27703150	Tumor
chr5	1298468		TERT	promoter	HIVID	Zhao et al.2016	27703150	Tumor
chr8	170453		RPL23AP53	ncRNA_intronic	HIVID	Zhao et al.2016	27703150	Tumor
chr16	90188753		PRDM7(dist=46415),NONE(dist=NONE)	intergenic	HIVID	Zhao et al.2016	27703150	Tumor
chr1	249240332		PGBD2(dist=26987),NONE(dist=NONE)	intergenic	HIVID	Zhao et al.2016	27703150	Tumor
chr1	249240303		PGBD2(dist=26958),NONE(dist=NONE)	intergenic	HIVID	Zhao et al.2016	27703150	Tumor
chr1	249240279		PGBD2(dist=26934),NONE(dist=NONE)	intergenic	HIVID	Zhao et al.2016	27703150	Tumor
chr1	249240051		PGBD2(dist=26706),NONE(dist=NONE)	intergenic	HIVID	Zhao et al.2016	27703150	Tumor
chr1	249240012		PGBD2(dist=26667),NONE(dist=NONE)	intergenic	HIVID	Zhao et al.2016	27703150	Tumor
chr11	65896547		PACS1	intronic	HIVID	Zhao et al.2016	27703150	Tumor
chr5	11756		NONE(dist=NONE),PLEKHG4B(dist=128617)	intergenic	HIVID	Zhao et al.2016	27703150	Tumor
chr5	11896		NONE(dist=NONE),PLEKHG4B(dist=128477)	intergenic	HIVID	Zhao et al.2016	27703150	Tumor
chr8	91867914		NECAB1	intronic	HIVID	Zhao et al.2016	27703150	Tumor
chr11	191830		LOC653486,ODF3,SCGB1C1	promoter	HIVID	Zhao et al.2016	27703150	Tumor
chr11	191910		LOC653486,ODF3,SCGB1C1	promoter	HIVID	Zhao et al.2016	27703150	Tumor
chr11	191800		LOC653486,ODF3,SCGB1C1	promoter	HIVID	Zhao et al.2016	27703150	Tumor
chr12	95341		LOC100288778(dist=4078),FAM138D(dist=52605)	intergenic	HIVID	Zhao et al.2016	27703150	Tumor
chr3	197751780		LMLN	intronic	HIVID	Zhao et al.2016	27703150	Tumor
chr8	137628302		KHDRBS3(dist=968454),FAM135B(dist=1513964)	intergenic	HIVID	Zhao et al.2016	27703150	Tumor
chr17	34936003		GGNBP2	intronic	HIVID	Zhao et al.2016	27703150	Tumor
chr15	102521277		DDX11L9;MIR6859-1,MIR6859-2,WASH3P	promoter;downstream	HIVID	Zhao et al.2016	27703150	Tumor
chr1	10365		DDX11L1;MIR6859-1,MIR6859-2,WASH7P	promoter;downstream	HIVID	Zhao et al.2016	27703150	Tumor
chr1	10464		DDX11L1;MIR6859-1,MIR6859-2,WASH7P	promoter;downstream	HIVID	Zhao et al.2016	27703150	Tumor
chr1	10033		DDX11L1;MIR6859-1,MIR6859-2,WASH7P	promoter;downstream	HIVID	Zhao et al.2016	27703150	Tumor
chr17	17183652		COPS3	intronic	HIVID	Zhao et al.2016	27703150	Tumor
chr17	17188480		COPS3	promoter	HIVID	Zhao et al.2016	27703150	Tumor
chr16	76821759		CNTNAP4(dist=228624),MIR4719(dist=81074)	intergenic	HIVID	Zhao et al.2016	27703150	Tumor
chr9	26851636		CAAP1	intronic	HIVID	Zhao et al.2016	27703150	Tumor
chr9	26851723		CAAP1	intronic	HIVID	Zhao et al.2016	27703150	Tumor
chr9	26851657		CAAP1	intronic	HIVID	Zhao et al.2016	27703150	Tumor
chr9	26851678		CAAP1	intronic	HIVID	Zhao et al.2016	27703150	Tumor
chr9	26851732		CAAP1	intronic	HIVID	Zhao et al.2016	27703150	Tumor
chr9	24811430		IZUMO3(dist=265756),TUSC1(dist=864957)	intergenic	HIVID	Zhao et al.2016	27703150	Tumor
chr15	53779444		ONECUT1(dist=697235),WDR72(dist=26494)	intergenic	HIVID	Zhao et al.2016	27703150	Tumor
chr15	53713362		ONECUT1(dist=631153),WDR72(dist=92576)	intergenic	HIVID	Zhao et al.2016	27703150	Tumor
chr17	22261609		MTRNR2L1(dist=237618),NONE(dist=NONE)	intergenic	HIVID	Zhao et al.2016	27703150	Tumor
chr18	2352941		LINC00470(dist=993311),METTL4(dist=184583)	intergenic	HIVID	Zhao et al.2016	27703150	Tumor
chr18	71803011		FBXO15	exonic	HIVID	Zhao et al.2016	27703150	Tumor
chr2	133028451		ANKRD30BL(dist=12909),GPR39(dist=145696)	intergenic	HIVID	Zhao et al.2016	27703150	Tumor
chr2	133028356		ANKRD30BL(dist=12814),GPR39(dist=145791)	intergenic	HIVID	Zhao et al.2016	27703150	Tumor
chr13	66789272		NONE(dist=NONE),PCDH9(dist=87694)	intergenic	HIVID	Zhao et al.2016	27703150	Tumor
chr17	22086492		MTRNR2L1(dist=62501),NONE(dist=NONE)	intergenic	HIVID	Zhao et al.2016	27703150	Tumor
chr3	182061415		LINC01206(dist=332951),FLJ46066(dist=103343)	intergenic	HIVID	Zhao et al.2016	27703150	Tumor
chr3	181966685		LINC01206(dist=238221),FLJ46066(dist=198073)	intergenic	HIVID	Zhao et al.2016	27703150	Tumor
chr3	7294807		GRM7	intronic	HIVID	Zhao et al.2016	27703150	Tumor
chr4	114441328		CAMK2D	intronic	HIVID	Zhao et al.2016	27703150	Tumor
chr16	31282400		ITGAM	exonic	HIVID	Zhao et al.2016	27703150	Tumor
chr8	52296331		PXDNL	intronic	HIVID	Zhao et al.2016	27703150	Tumor
chr11	59470973		PATL1(dist=34462),OR10V1(dist=9416)	intergenic	HIVID	Zhao et al.2016	27703150	Tumor

Chromosome	Integration site in host genome	Integration site in virus genome	Gene (distance, bp)	Regions	Methods	Author	PMID	Sample
chr7	114013265		NONE(dist=NONE),NONE(dist=NONE)	intergenic	HIVID	Zhao et al.2016	27703150	Tumor
chr7	114013219		NONE(dist=NONE),NONE(dist=NONE)	intergenic	HIVID	Zhao et al.2016	27703150	Tumor
chr5	30018263		LOC101929681(dist=622180),CDH6(dist=1175499)	intergenic	HIVID	Zhao et al.2016	27703150	Tumor
chr18	57006859		LMAN1	intronic	HIVID	Zhao et al.2016	27703150	Tumor
chr4	163504721		FSTL5(dist=419535),NAF1(dist=543139)	intergenic	HIVID	Zhao et al.2016	27703150	Tumor
chr7	133775388		EXOC4(dist=24875),LRGUK(dist=36717)	intergenic	HIVID	Zhao et al.2016	27703150	Tumor
chr7	133739428		EXOC4	intronic	HIVID	Zhao et al.2016	27703150	Tumor
chr14	19768104		DUXAP10(dist=75188),DUXAP10(dist=114364)	intergenic	HIVID	Zhao et al.2016	27703150	Tumor
chr2	47429731		AC073283.4	ncRNA_intronic	HIVID	Zhao et al.2016	27703150	Tumor
chr2	47420967		AC073283.4	ncRNA_intronic	HIVID	Zhao et al.2016	27703150	Tumor
chr2	47429765		AC073283.4	ncRNA_intronic	HIVID	Zhao et al.2016	27703150	Tumor
chr2	47429784		AC073283.4	ncRNA_intronic	HIVID	Zhao et al.2016	27703150	Tumor
chr2	47429805		AC073283.4	ncRNA_intronic	HIVID	Zhao et al.2016	27703150	Tumor
chr9	2590992		VLDLR-AS1	ncRNA_intronic	HIVID	Zhao et al.2016	27703150	Tumor
chr18	53689152		TCF4(dist=385964),RP11-456O19.4(dist=61435)	intergenic	HIVID	Zhao et al.2016	27703150	Tumor
chr8	67118305		LINC00967(dist=8751),RP11-346I3.4(dist=213517)	intergenic	HIVID	Zhao et al.2016	27703150	Tumor
chr2	224085040		KCNE4(dist=164683),SCG2(dist=376618)	intergenic	HIVID	Zhao et al.2016	27703150	Tumor
chr5	913153		TRIP13	intronic	HIVID	Zhao et al.2016	27703150	Tumor
chr5	1295363		TERT	promoter	HIVID	Zhao et al.2016	27703150	Tumor
chr8	43827052		POTEA(dist=608724),NONE(dist=NONE)	intergenic	HIVID	Zhao et al.2016	27703150	Tumor
chr17	17409193		PEMT	intronic	HIVID	Zhao et al.2016	27703150	Tumor
chr8	46844866		NONE(dist=NONE),LINC00293(dist=907642)	intergenic	HIVID	Zhao et al.2016	27703150	Tumor
chr13	80299574		NDFIP2(dist=169362),LINC01080(dist=285638)	intergenic	HIVID	Zhao et al.2016	27703150	Tumor
chr13	80299436		NDFIP2(dist=169224),LINC01080(dist=285776)	intergenic	HIVID	Zhao et al.2016	27703150	Tumor
chr17	17375840		MED9	promoter	HIVID	Zhao et al.2016	27703150	Tumor
chr4	122737861		CCNA2	UTR3	HIVID	Zhao et al.2016	27703150	Tumor
chr4	122737868		CCNA2	UTR3	HIVID	Zhao et al.2016	27703150	Tumor
chr17	21449855		C17orf51	intronic	HIVID	Zhao et al.2016	27703150	Tumor
chr5	1295503		TERT	promoter	HIVID	Zhao et al.2016	27703150	Tumor
chr5	1295500		TERT	promoter	HIVID	Zhao et al.2016	27703150	Tumor
chr7	31963246		PDE1C	intronic	HIVID	Zhao et al.2016	27703150	Tumor
chr7	31965606		PDE1C	intronic	HIVID	Zhao et al.2016	27703150	Tumor
chr7	31963179		PDE1C	intronic	HIVID	Zhao et al.2016	27703150	Tumor
chr7	6503858		KDELR2	intronic	HIVID	Zhao et al.2016	27703150	Tumor
chr15	78450807		IDH3A	intronic	HIVID	Zhao et al.2016	27703150	Tumor
chr13	94992005		GPC6	intronic	HIVID	Zhao et al.2016	27703150	Tumor
chr6	76245909		FILIP1(dist=42413),SENP6(dist=65713)	intergenic	HIVID	Zhao et al.2016	27703150	Tumor
chr6	76245874		FILIP1(dist=42378),SENP6(dist=65748)	intergenic	HIVID	Zhao et al.2016	27703150	Tumor
chr21	40667136		BRWD1	intronic	HIVID	Zhao et al.2016	27703150	Tumor
chr2	144561084		ARHGAP15(dist=35163),AC016910.1(dist=133550)	intergenic	HIVID	Zhao et al.2016	27703150	Tumor
chr8	55309670		MRPL15(dist=248596),SOX17(dist=60825)	intergenic	HIVID	Zhao et al.2016	27703150	Tumor
chr8	64583925		YTHDF3(dist=458579),RP11-579E24.1(dist=98063)	intergenic	HIVID	Zhao et al.2016	27703150	Tumor
chr5	1295751		TERT	promoter	HIVID	Zhao et al.2016	27703150	Tumor
chr8	57331126		SDR16C6P(dist=27857),PENK(dist=22387)	intergenic	HIVID	Zhao et al.2016	27703150	Tumor
chr9	66971174		PTGER4P2-CDK2AP2P2(dist=468144),RP11-381O7.3(dist=4620	intergenic	HIVID	Zhao et al.2016	27703150	Tumor
chr3	46816063		PRSS45(dist=29818),PRSS42(dist=55831)	intergenic	HIVID	Zhao et al.2016	27703150	Tumor
chr12	102704223		PMCH(dist=112609),IGF1(dist=85422)	intergenic	HIVID	Zhao et al.2016	27703150	Tumor
chr14	80401313		NRXN3(dist=66680),DIO2(dist=262555)	intergenic	HIVID	Zhao et al.2016	27703150	Tumor
chr14	80237449		NRXN3	intronic	HIVID	Zhao et al.2016	27703150	Tumor
chr10	42385227		NONE(dist=NONE),LOC441666(dist=442087)	intergenic	HIVID	Zhao et al.2016	27703150	Tumor
chr10	42392651		NONE(dist=NONE),LOC441666(dist=434663)	intergenic	HIVID	Zhao et al.2016	27703150	Tumor
chr16	46405134		NONE(dist=NONE),ANKRD26P1(dist=98115)	intergenic	HIVID	Zhao et al.2016	27703150	Tumor
chr16	46405207		NONE(dist=NONE),ANKRD26P1(dist=98042)	intergenic	HIVID	Zhao et al.2016	27703150	Tumor
chr16	46407828		NONE(dist=NONE),ANKRD26P1(dist=95421)	intergenic	HIVID	Zhao et al.2016	27703150	Tumor
chr16	46407949		NONE(dist=NONE),ANKRD26P1(dist=95300)	intergenic	HIVID	Zhao et al.2016	27703150	Tumor
chr16	46408721		NONE(dist=NONE),ANKRD26P1(dist=94528)	intergenic	HIVID	Zhao et al.2016	27703150	Tumor
chr16	46408891		NONE(dist=NONE),ANKRD26P1(dist=94358)	intergenic	HIVID	Zhao et al.2016	27703150	Tumor
chr16	46420783		NONE(dist=NONE),ANKRD26P1(dist=82466)	intergenic	HIVID	Zhao et al.2016	27703150	Tumor
chr16	46421745		NONE(dist=NONE),ANKRD26P1(dist=81504)	intergenic	HIVID	Zhao et al.2016	27703150	Tumor
chr16	46424557		NONE(dist=NONE),ANKRD26P1(dist=78692)	intergenic	HIVID	Zhao et al.2016	27703150	Tumor
chr16	46432835		NONE(dist=NONE),ANKRD26P1(dist=70414)	intergenic	HIVID	Zhao et al.2016	27703150	Tumor
chr16	46387375		NONE(dist=NONE),ANKRD26P1(dist=115874)	intergenic	HIVID	Zhao et al.2016	27703150	Tumor
chr16	46393214		NONE(dist=NONE),ANKRD26P1(dist=110035)	intergenic	HIVID	Zhao et al.2016	27703150	Tumor
chr16	46396029		NONE(dist=NONE),ANKRD26P1(dist=107220)	intergenic	HIVID	Zhao et al.2016	27703150	Tumor
chr16	46396994		NONE(dist=NONE),ANKRD26P1(dist=106255)	intergenic	HIVID	Zhao et al.2016	27703150	Tumor
chr16	46397844		NONE(dist=NONE),ANKRD26P1(dist=105405)	intergenic	HIVID	Zhao et al.2016	27703150	Tumor
chr16	46401837		NONE(dist=NONE),ANKRD26P1(dist=101412)	intergenic	HIVID	Zhao et al.2016	27703150	Tumor
chr16	46402802		NONE(dist=NONE),ANKRD26P1(dist=100447)	intergenic	HIVID	Zhao et al.2016	27703150	Tumor
chr4	63333001		LPHN3(dist=394833),TECRL(dist=1811176)	intergenic	HIVID	Zhao et al.2016	27703150	Tumor
chr5	155038398		KIF4B(dist=640713),SGCD(dist=715369)	intergenic	HIVID	Zhao et al.2016	27703150	Tumor
chr3	159144304		IQCJ-SCHIP1,SCHIP1	intronic	HIVID	Zhao et al.2016	27703150	Tumor
chr17	14872726		HS3ST3B1(dist=623234),CDRT7(dist=61566)	intergenic	HIVID	Zhao et al.2016	27703150	Tumor
chr1	79979625		ELTD1(dist=507130),NONE(dist=NONE)	intergenic	HIVID	Zhao et al.2016	27703150	Tumor
chr13	99597245		DOCK9	intronic	HIVID	Zhao et al.2016	27703150	Tumor
chr11	91906061		DISC1FP1(dist=1257841),FAT3(dist=179201)	intergenic	HIVID	Zhao et al.2016	27703150	Tumor
chr2	132981581		ANKRD30BL	ncRNA_intronic	HIVID	Zhao et al.2016	27703150	Tumor
chr5	139897855		ANKHD1,ANKHD1-EIF4EBP3	intronic	HIVID	Zhao et al.2016	27703150	Tumor
chr5	33728966		ADAMTS12	intronic	HIVID	Zhao et al.2016	27703150	Tumor
chr3	167198335		WDR49	intronic	HIVID	Zhao et al.2016	27703150	Tumor
chr5	1295244		TERT	promoter	HIVID	Zhao et al.2016	27703150	Tumor
chr5	1295282		TERT	promoter	HIVID	Zhao et al.2016	27703150	Tumor
chr19	49080827		SULT2B1	intronic	HIVID	Zhao et al.2016	27703150	Tumor
chr13	95530167		SOX21-AS1(dist=161968),ABCC4(dist=141916)	intergenic	HIVID	Zhao et al.2016	27703150	Tumor
chr3	63760613		SNTN(dist=109722),C3orf49(dist=44428)	intergenic	HIVID	Zhao et al.2016	27703150	Tumor
chr6	67497425		SLC25A51P1(dist=998049),BAI3(dist=1848207)	intergenic	HIVID	Zhao et al.2016	27703150	Tumor
chr2	148538823		PABPC1P2(dist=1190265),ACVR2A(dist=63263)	intergenic	HIVID	Zhao et al.2016	27703150	Tumor
chr10	42528071		NONE(dist=NONE),LOC441666(dist=299243)	intergenic	HIVID	Zhao et al.2016	27703150	Tumor
chr16	46411908		NONE(dist=NONE),ANKRD26P1(dist=91341)	intergenic	HIVID	Zhao et al.2016	27703150	Tumor
chr16	46415941		NONE(dist=NONE),ANKRD26P1(dist=87308)	intergenic	HIVID	Zhao et al.2016	27703150	Tumor
chr16	46422608		NONE(dist=NONE),ANKRD26P1(dist=80641)	intergenic	HIVID	Zhao et al.2016	27703150	Tumor
chr16	46429776		NONE(dist=NONE),ANKRD26P1(dist=73473)	intergenic	HIVID	Zhao et al.2016	27703150	Tumor
chr16	46429839		NONE(dist=NONE),ANKRD26P1(dist=73410)	intergenic	HIVID	Zhao et al.2016	27703150	Tumor
chr16	46389258		NONE(dist=NONE),ANKRD26P1(dist=113991)	intergenic	HIVID	Zhao et al.2016	27703150	Tumor
chr16	46395166		NONE(dist=NONE),ANKRD26P1(dist=108083)	intergenic	HIVID	Zhao et al.2016	27703150	Tumor

Chromosome	Integration site in host genome	Integration site in virus genome	Gene (distance, bp)	Regions	Methods	Author	PMID	Sample
chr13	100064924		MIR548AN	promoter	HIVID	Zhao et al.2016	27703150	Tumor
chr13	100065114		MIR548AN	promoter	HIVID	Zhao et al.2016	27703150	Tumor
chr9	7233279		KDM4C(dist=57631),TMEM261(dist=563212)	intergenic	HIVID	Zhao et al.2016	27703150	Tumor
chr10	100725909		HPSE2	intronic	HIVID	Zhao et al.2016	27703150	Tumor
chr10	100726692		HPSE2	intronic	HIVID	Zhao et al.2016	27703150	Tumor
chr16	5332286		FAM86A(dist=184465),MIR8065(dist=350182)	intergenic	HIVID	Zhao et al.2016	27703150	Tumor
chr8	62361661		CLVS1	intronic	HIVID	Zhao et al.2016	27703150	Tumor
chr11	27873654		BDNF(dist=130049),KIF18A(dist=168509)	intergenic	HIVID	Zhao et al.2016	27703150	Tumor
chr5	11480		NONE(dist=NONE),PLEKHG4B(dist=128893)	intergenic	HIVID	Zhao et al.2016	27703150	Tumor
chr5	11583		NONE(dist=NONE),PLEKHG4B(dist=128790)	intergenic	HIVID	Zhao et al.2016	27703150	Tumor
chr17	22258326		MTRNR2L1(dist=234335),NONE(dist=NONE)	intergenic	HIVID	Zhao et al.2016	27703150	Tumor
chr19	36212895		KMT2B	intronic	HIVID	Zhao et al.2016	27703150	Tumor
chr1	121356834		EMBP1(dist=43148),NONE(dist=NONE)	intergenic	HIVID	Zhao et al.2016	27703150	Tumor
chr17	3390842		ASPA	intronic	HIVID	Zhao et al.2016	27703150	Tumor
chr15	47059885		SQRDL(dist=1076393),SEMA6D(dist=416518)	intergenic	HIVID	Zhao et al.2016	27703150	Tumor
chr5	10000		NONE(dist=NONE),PLEKHG4B(dist=130373)	intergenic	HIVID	Zhao et al.2016	27703150	Tumor
chr15	20003555		NONE(dist=NONE),CHEK2P2(dist=484442)	intergenic	HIVID	Zhao et al.2016	27703150	Tumor
chr3	176122315		NAALADL2(dist=598887),LINC01208(dist=199621)	intergenic	HIVID	Zhao et al.2016	27703150	Tumor
chr19	36212786		KMT2B	intronic	HIVID	Zhao et al.2016	27703150	Tumor
chr19	36212842		KMT2B	intronic	HIVID	Zhao et al.2016	27703150	Tumor
chr19	36212922		KMT2B	intronic	HIVID	Zhao et al.2016	27703150	Tumor
chr15	20778072		GOLGA8CP	ncRNA_exonic	HIVID	Zhao et al.2016	27703150	Tumor
chr1	10073		DDX11L1;MIR6859-1,MIR6859-2,WASH7P	promoter;downstream	HIVID	Zhao et al.2016	27703150	Tumor
chr11	51572041		OR4C46(dist=55830),NONE(dist=NONE)	intergenic	HIVID	Zhao et al.2016	27703150	Tumor
chr11	51567277		OR4C46(dist=51066),NONE(dist=NONE)	intergenic	HIVID	Zhao et al.2016	27703150	Tumor
chr19	27750307		NONE(dist=NONE),LINC00662(dist=531094)	intergenic	HIVID	Zhao et al.2016	27703150	Tumor
chr19	27750345		NONE(dist=NONE),LINC00662(dist=531056)	intergenic	HIVID	Zhao et al.2016	27703150	Tumor
chr11	69167450		MYEOV(dist=102696),CCND1(dist=288423)	intergenic	HIVID	Zhao et al.2016	27703150	Tumor
chr17	22260820		MTRNR2L1(dist=236829),NONE(dist=NONE)	intergenic	HIVID	Zhao et al.2016	27703150	Tumor
chr17	22259249		MTRNR2L1(dist=235258),NONE(dist=NONE)	intergenic	HIVID	Zhao et al.2016	27703150	Tumor
chr17	22259158		MTRNR2L1(dist=235167),NONE(dist=NONE)	intergenic	HIVID	Zhao et al.2016	27703150	Tumor
chr17	22258439		MTRNR2L1(dist=234448),NONE(dist=NONE)	intergenic	HIVID	Zhao et al.2016	27703150	Tumor
chr17	22253301		MTRNR2L1(dist=229310),NONE(dist=NONE)	intergenic	HIVID	Zhao et al.2016	27703150	Tumor
chr17	22253215		MTRNR2L1(dist=229224),NONE(dist=NONE)	intergenic	HIVID	Zhao et al.2016	27703150	Tumor
chr17	22252111		MTRNR2L1(dist=228120),NONE(dist=NONE)	intergenic	HIVID	Zhao et al.2016	27703150	Tumor
chr17	22252022		MTRNR2L1(dist=228031),NONE(dist=NONE)	intergenic	HIVID	Zhao et al.2016	27703150	Tumor
chr17	22251305		MTRNR2L1(dist=227314),NONE(dist=NONE)	intergenic	HIVID	Zhao et al.2016	27703150	Tumor
chr17	22250839		MTRNR2L1(dist=226848),NONE(dist=NONE)	intergenic	HIVID	Zhao et al.2016	27703150	Tumor
chr17	22249776		MTRNR2L1(dist=225785),NONE(dist=NONE)	intergenic	HIVID	Zhao et al.2016	27703150	Tumor
chr17	22244979		MTRNR2L1(dist=220988),NONE(dist=NONE)	intergenic	HIVID	Zhao et al.2016	27703150	Tumor
chr17	22244945		MTRNR2L1(dist=220954),NONE(dist=NONE)	intergenic	HIVID	Zhao et al.2016	27703150	Tumor
chr17	22244888		MTRNR2L1(dist=220897),NONE(dist=NONE)	intergenic	HIVID	Zhao et al.2016	27703150	Tumor
chr16	27229423		KDM8	intronic	HIVID	Zhao et al.2016	27703150	Tumor
chr5	46367162		HCN1(dist=670942),NONE(dist=NONE)	intergenic	HIVID	Zhao et al.2016	27703150	Tumor
chr5	46367108		HCN1(dist=670888),NONE(dist=NONE)	intergenic	HIVID	Zhao et al.2016	27703150	Tumor
chr5	46367071		HCN1(dist=670851),NONE(dist=NONE)	intergenic	HIVID	Zhao et al.2016	27703150	Tumor
chr14	39937237		FBXO33(dist=35533),LOC644919(dist=1486679)	intergenic	HIVID	Zhao et al.2016	27703150	Tumor
chr3	90354707		EPHA3(dist=823423),NONE(dist=NONE)	intergenic	HIVID	Zhao et al.2016	27703150	Tumor
chr3	90354666		EPHA3(dist=823382),NONE(dist=NONE)	intergenic	HIVID	Zhao et al.2016	27703150	Tumor
chr1	121373281		EMBP1(dist=59595),NONE(dist=NONE)	intergenic	HIVID	Zhao et al.2016	27703150	Tumor
chr1	121357009		EMBP1(dist=43323),NONE(dist=NONE)	intergenic	HIVID	Zhao et al.2016	27703150	Tumor
chr1	121356719		EMBP1(dist=43033),NONE(dist=NONE)	intergenic	HIVID	Zhao et al.2016	27703150	Tumor
chr1	121355776		EMBP1(dist=42090),NONE(dist=NONE)	intergenic	HIVID	Zhao et al.2016	27703150	Tumor
chr1	121355272		EMBP1(dist=41586),NONE(dist=NONE)	intergenic	HIVID	Zhao et al.2016	27703150	Tumor
chr1	121355022		EMBP1(dist=41336),NONE(dist=NONE)	intergenic	HIVID	Zhao et al.2016	27703150	Tumor
chr1	121352274		EMBP1(dist=38588),NONE(dist=NONE)	intergenic	HIVID	Zhao et al.2016	27703150	Tumor
chr1	121352211		EMBP1(dist=38525),NONE(dist=NONE)	intergenic	HIVID	Zhao et al.2016	27703150	Tumor
chr1	121352171		EMBP1(dist=38485),NONE(dist=NONE)	intergenic	HIVID	Zhao et al.2016	27703150	Tumor
chr11	69479002		CCND1(dist=9760),ORAOV1(dist=1330)	intergenic	HIVID	Zhao et al.2016	27703150	Tumor
chr17	35554513		ACACA	intronic	HIVID	Zhao et al.2016	27703150	Tumor
chr17	35554443		ACACA	intronic	HIVID	Zhao et al.2016	27703150	Tumor
chr19	54009749		ZNF813(dist=12203),ZNF331(dist=14428)	intergenic	HIVID	Zhao et al.2016	27703150	Tumor
chr19	54561337		VSTM1	intronic	HIVID	Zhao et al.2016	27703150	Tumor
chr3	42008897		ULK4	promoter	HIVID	Zhao et al.2016	27703150	Tumor
chr2	130974447		TUBA3E(dist=18413),CCDC115(dist=121059)	intergenic	HIVID	Zhao et al.2016	27703150	Tumor
chr11	55025032		TRIM48	promoter	HIVID	Zhao et al.2016	27703150	Tumor
chr4	154093517		TRIM2	intronic	HIVID	Zhao et al.2016	27703150	Tumor
chr5	1307629		TERT(dist=12467),MIR4457(dist=1796)	intergenic	HIVID	Zhao et al.2016	27703150	Tumor
chr5	1307625		TERT(dist=12463),MIR4457(dist=1800)	intergenic	HIVID	Zhao et al.2016	27703150	Tumor
chr5	1307595		TERT(dist=12433),MIR4457(dist=1830)	intergenic	HIVID	Zhao et al.2016	27703150	Tumor
chr5	1307577		TERT(dist=12415),MIR4457(dist=1848)	intergenic	HIVID	Zhao et al.2016	27703150	Tumor
chr5	1307537		TERT(dist=12375),MIR4457(dist=1888)	intergenic	HIVID	Zhao et al.2016	27703150	Tumor
chr5	1307516		TERT(dist=12354),MIR4457(dist=1909)	intergenic	HIVID	Zhao et al.2016	27703150	Tumor
chr5	1307511		TERT(dist=12349),MIR4457(dist=1914)	intergenic	HIVID	Zhao et al.2016	27703150	Tumor
chr5	1307487		TERT(dist=12325),MIR4457(dist=1938)	intergenic	HIVID	Zhao et al.2016	27703150	Tumor
chr2	201325710		SPATS2L	intronic	HIVID	Zhao et al.2016	27703150	Tumor
chr8	83570452		SNX16(dist=815931),RALYL(dist=1525001)	intergenic	HIVID	Zhao et al.2016	27703150	Tumor
chr6	100905953		SIM1	intronic	HIVID	Zhao et al.2016	27703150	Tumor
chr1	234729304		RP5-855F14.1(dist=61779),IRF2BP2(dist=10711)	intergenic	HIVID	Zhao et al.2016	27703150	Tumor
chr16	33958002		RNU6-76P(dist=394759),LINC00273(dist=3050)	intergenic	HIVID	Zhao et al.2016	27703150	Tumor
chr16	33955267		RNU6-76P(dist=392024),LINC00273(dist=5785)	intergenic	HIVID	Zhao et al.2016	27703150	Tumor
chr9	66971337		PTGER4P2-CDK2AP2P2(dist=468307),RP11-381O7.3(dist=4603[illegible]	intergenic	HIVID	Zhao et al.2016	27703150	Tumor
chr9	66971200		PTGER4P2-CDK2AP2P2(dist=468170),RP11-381O7.3(dist=4617[illegible]	intergenic	HIVID	Zhao et al.2016	27703150	Tumor
chr11	123717339		OR6M1(dist=40282),TMEM225(dist=36294)	intergenic	HIVID	Zhao et al.2016	27703150	Tumor
chr11	48961136		OR4A47(dist=449862),TRIM49B(dist=92016)	intergenic	HIVID	Zhao et al.2016	27703150	Tumor
chr11	48953779		OR4A47(dist=442505),TRIM49B(dist=99373)	intergenic	HIVID	Zhao et al.2016	27703150	Tumor
chr11	48932551		OR4A47(dist=421277),TRIM49B(dist=120601)	intergenic	HIVID	Zhao et al.2016	27703150	Tumor
chr11	48932484		OR4A47(dist=421210),TRIM49B(dist=120668)	intergenic	HIVID	Zhao et al.2016	27703150	Tumor
chr11	48877525		OR4A47(dist=366251),TRIM49B(dist=175627)	intergenic	HIVID	Zhao et al.2016	27703150	Tumor
chr11	48859432		OR4A47(dist=348158),TRIM49B(dist=193720)	intergenic	HIVID	Zhao et al.2016	27703150	Tumor
chr11	48859359		OR4A47(dist=348085),TRIM49B(dist=193793)	intergenic	HIVID	Zhao et al.2016	27703150	Tumor
chr2	50626996		NRXN1	intronic	HIVID	Zhao et al.2016	27703150	Tumor
chr2	50626932		NRXN1	intronic	HIVID	Zhao et al.2016	27703150	Tumor
chr11	39434992		NONE(dist=NONE),LRRC4C(dist=700759)	intergenic	HIVID	Zhao et al.2016	27703150	Tumor

Chromosome	Integration site in host genome	Integration site in virus genome	Gene (distance, bp)	Regions	Methods	Author	PMID	Sample
chr10	42355381		NONE(dist=NONE),LOC441666(dist=471933)	intergenic	HIVID	Zhao et al.2016	27703150	Tumor
chr10	42355506		NONE(dist=NONE),LOC441666(dist=471808)	intergenic	HIVID	Zhao et al.2016	27703150	Tumor
chr8	46853717		NONE(dist=NONE),LINC00293(dist=898791)	intergenic	HIVID	Zhao et al.2016	27703150	Tumor
chr12	66451456		MIR6074(dist=33950),LLPH(dist=65393)	intergenic	HIVID	Zhao et al.2016	27703150	Tumor
chr12	66451439		MIR6074(dist=33933),LLPH(dist=65410)	intergenic	HIVID	Zhao et al.2016	27703150	Tumor
chr12	66451369		MIR6074(dist=33863),LLPH(dist=65480)	intergenic	HIVID	Zhao et al.2016	27703150	Tumor
chr2	170217908		LRP2	intronic	HIVID	Zhao et al.2016	27703150	Tumor
chr2	170217823		LRP2	intronic	HIVID	Zhao et al.2016	27703150	Tumor
chr2	243152622		LOC728323(dist=50153),NONE(dist=NONE)	intergenic	HIVID	Zhao et al.2016	27703150	Tumor
chr2	243152610		LOC728323(dist=50141),NONE(dist=NONE)	intergenic	HIVID	Zhao et al.2016	27703150	Tumor
chr2	243152544		LOC728323(dist=50075),NONE(dist=NONE)	intergenic	HIVID	Zhao et al.2016	27703150	Tumor
chr2	243152514		LOC728323(dist=50045),NONE(dist=NONE)	intergenic	HIVID	Zhao et al.2016	27703150	Tumor
chr17	70608319		LINC00511	ncRNA_intronic	HIVID	Zhao et al.2016	27703150	Tumor
chr18	1747041		LINC00470(dist=387411),METTL4(dist=790483)	intergenic	HIVID	Zhao et al.2016	27703150	Tumor
chr14	56114503		KTN1	intronic	HIVID	Zhao et al.2016	27703150	Tumor
chr17	25846584		KSR1	intronic	HIVID	Zhao et al.2016	27703150	Tumor
chr22	25489823		KIAA1671	intronic	HIVID	Zhao et al.2016	27703150	Tumor
chr11	77675455		INTS4	intronic	HIVID	Zhao et al.2016	27703150	Tumor
chr6	160521842		IGF2R	intronic	HIVID	Zhao et al.2016	27703150	Tumor
chr6	160521779		IGF2R	intronic	HIVID	Zhao et al.2016	27703150	Tumor
chr3	183746950		HTR3D	promoter	HIVID	Zhao et al.2016	27703150	Tumor
chr17	14907187		HS3ST3B1(dist=657695),CDRT7(dist=27105)	intergenic	HIVID	Zhao et al.2016	27703150	Tumor
chr6	105330800		HACE1(dist=23006),LINC00577(dist=53369)	intergenic	HIVID	Zhao et al.2016	27703150	Tumor
chr17	10146880		GAS7(dist=45012),MYH13(dist=57303)	intergenic	HIVID	Zhao et al.2016	27703150	Tumor
chr16	35270603		FLJ26245(dist=279608),NONE(dist=NONE)	intergenic	HIVID	Zhao et al.2016	27703150	Tumor
chr8	94735401		FAM92A1	intronic	HIVID	Zhao et al.2016	27703150	Tumor
chr5	137320636		FAM13B	intronic	HIVID	Zhao et al.2016	27703150	Tumor
chr3	90368936		EPHA3(dist=837652),NONE(dist=NONE)	intergenic	HIVID	Zhao et al.2016	27703150	Tumor
chr8	120694634		ENPP2(dist=43528),TAF2(dist=48380)	intergenic	HIVID	Zhao et al.2016	27703150	Tumor
chr11	118647385		DDX6	intronic	HIVID	Zhao et al.2016	27703150	Tumor
chr19	16031930		CYP4F11	intronic	HIVID	Zhao et al.2016	27703150	Tumor
chr10	126787260		CTBP2	intronic	HIVID	Zhao et al.2016	27703150	Tumor
chr12	39535733		CPNE8(dist=236313),KIF21A(dist=151297)	intergenic	HIVID	Zhao et al.2016	27703150	Tumor
chr12	109157219		CORO1C(dist=31893),SSH1(dist=19247)	intergenic	HIVID	Zhao et al.2016	27703150	Tumor
chr7	43722628		COA1	intronic	HIVID	Zhao et al.2016	27703150	Tumor
chr3	99834475		CMSS1	intronic	HIVID	Zhao et al.2016	27703150	Tumor
chr15	101034629		CERS3	intronic	HIVID	Zhao et al.2016	27703150	Tumor
chr16	61802288		CDH8	intronic	HIVID	Zhao et al.2016	27703150	Tumor
chr6	34618399		C6orf106	intronic	HIVID	Zhao et al.2016	27703150	Tumor
chr5	60956395		C5orf64	intronic	HIVID	Zhao et al.2016	27703150	Tumor
chr5	60956465		C5orf64	intronic	HIVID	Zhao et al.2016	27703150	Tumor
chr11	68051170		C11orf24(dist=11701),LRP5(dist=28938)	intergenic	HIVID	Zhao et al.2016	27703150	Tumor
chr17	59400142		BCAS3	intronic	HIVID	Zhao et al.2016	27703150	Tumor
chr2	170334468		BBS5	promoter	HIVID	Zhao et al.2016	27703150	Tumor
chr6	17092226		ATXN1(dist=330505),STMND1(dist=10263)	intergenic	HIVID	Zhao et al.2016	27703150	Tumor
chr7	149709581		ATP6V0E2(dist=131794),ACTR3C(dist=234720)	intergenic	HIVID	Zhao et al.2016	27703150	Tumor
chr2	232231737		ARMC9	intronic	HIVID	Zhao et al.2016	27703150	Tumor
chr11	120318374		ARHGEF12	intronic	HIVID	Zhao et al.2016	27703150	Tumor
chr2	133020974		ANKRD30BL,MIR663B	promoter	HIVID	Zhao et al.2016	27703150	Tumor
chr2	133028600		ANKRD30BL(dist=13058),GPR39(dist=145547)	intergenic	HIVID	Zhao et al.2016	27703150	Tumor
chr2	133028541		ANKRD30BL(dist=12999),GPR39(dist=145606)	intergenic	HIVID	Zhao et al.2016	27703150	Tumor
chr2	133028200		ANKRD30BL(dist=12658),GPR39(dist=145947)	intergenic	HIVID	Zhao et al.2016	27703150	Tumor
chr2	133028154		ANKRD30BL(dist=12612),GPR39(dist=145993)	intergenic	HIVID	Zhao et al.2016	27703150	Tumor
chr2	133027337		ANKRD30BL(dist=11795),GPR39(dist=146810)	intergenic	HIVID	Zhao et al.2016	27703150	Tumor
chr2	133027224		ANKRD30BL(dist=11682),GPR39(dist=146923)	intergenic	HIVID	Zhao et al.2016	27703150	Tumor
chr1	36377801		AGO1	intronic	HIVID	Zhao et al.2016	27703150	Tumor
chr2	155433707		AC009227.2(dist=119757),KCNJ3(dist=121386)	intergenic	HIVID	Zhao et al.2016	27703150	Tumor
chr2	155433675		AC009227.2(dist=119725),KCNJ3(dist=121418)	intergenic	HIVID	Zhao et al.2016	27703150	Tumor
chr3	141098009		ZBTB38	intronic	HIVID	Zhao et al.2016	27703150	Tumor
chr16	28169369		XPO6	intronic	HIVID	Zhao et al.2016	27703150	Tumor
chr16	69946946		WWP2	intronic	HIVID	Zhao et al.2016	27703150	Tumor
chr16	69946895		WWP2	intronic	HIVID	Zhao et al.2016	27703150	Tumor
chr1	241870621		WDR64	intronic	HIVID	Zhao et al.2016	27703150	Tumor
chr3	11726449		VGLL4	intronic	HIVID	Zhao et al.2016	27703150	Tumor
chr1	62894735		USP1	promoter	HIVID	Zhao et al.2016	27703150	Tumor
chr5	158709549		UBLCP1	intronic	HIVID	Zhao et al.2016	27703150	Tumor
chr10	134744574		TTC40	intronic	HIVID	Zhao et al.2016	27703150	Tumor
chr8	143298856		TSNARE1	intronic	HIVID	Zhao et al.2016	27703150	Tumor
chr4	8549982		TRMT44(dist=71700),GPR78(dist=32235)	intergenic	HIVID	Zhao et al.2016	27703150	Tumor
chr6	30442233		TRIM39-RPP21(dist=127598),HLA-E(dist=14950)	intergenic	HIVID	Zhao et al.2016	27703150	Tumor
chr7	145611251		TPK1(dist=1078105),CNTNAP2(dist=202202)	intergenic	HIVID	Zhao et al.2016	27703150	Tumor
chr7	19885181		TMEM196(dist=72777),AC005062.2(dist=73423)	intergenic	HIVID	Zhao et al.2016	27703150	Tumor
chr2	103911876		TMEM182(dist=477738),LOC100287010(dist=1083432)	intergenic	HIVID	Zhao et al.2016	27703150	Tumor
chr2	88483082		THNSL2	intronic	HIVID	Zhao et al.2016	27703150	Tumor
chr19	32809122		THEG5(dist=724666),ZNF507(dist=27392)	intergenic	HIVID	Zhao et al.2016	27703150	Tumor
chr19	32809078		THEG5(dist=724622),ZNF507(dist=27436)	intergenic	HIVID	Zhao et al.2016	27703150	Tumor
chr12	116230447		TBX3(dist=1108478),MED13L(dist=165934)	intergenic	HIVID	Zhao et al.2016	27703150	Tumor
chr2	159892270		TANC1	intronic	HIVID	Zhao et al.2016	27703150	Tumor
chr4	7352286		SORCS2	intronic	HIVID	Zhao et al.2016	27703150	Tumor
chr1	53566147		SLC1A7	intronic	HIVID	Zhao et al.2016	27703150	Tumor
chr22	43760025		SCUBE1(dist=20631),MPPED1(dist=47995)	intergenic	HIVID	Zhao et al.2016	27703150	Tumor
chr16	21887287		RRN3P1(dist=56792),UQCRC2(dist=77322)	intergenic	HIVID	Zhao et al.2016	27703150	Tumor
chr16	21887228		RRN3P1(dist=56733),UQCRC2(dist=77381)	intergenic	HIVID	Zhao et al.2016	27703150	Tumor
chr1	213596912		RPS6KC1(dist=150104),LINC00538(dist=501180)	intergenic	HIVID	Zhao et al.2016	27703150	Tumor
chr3	128347725		RPN1	intronic	HIVID	Zhao et al.2016	27703150	Tumor
chr17	1834192		RPA1(dist=31344),RTN4RL1(dist=3779)	intergenic	HIVID	Zhao et al.2016	27703150	Tumor
chr20	22451371		RP5-1004I9.1(dist=50090),LINC00261(dist=89821)	intergenic	HIVID	Zhao et al.2016	27703150	Tumor
chr6	4480311		RP3-400B16.1(dist=322692),NONE(dist=NONE)	intergenic	HIVID	Zhao et al.2016	27703150	Tumor
chr6	4480266		RP3-400B16.1(dist=322647),NONE(dist=NONE)	intergenic	HIVID	Zhao et al.2016	27703150	Tumor
chr6	4480188		RP3-400B16.1(dist=322569),NONE(dist=NONE)	intergenic	HIVID	Zhao et al.2016	27703150	Tumor
chr16	2318214		RNPS1	UTR5	HIVID	Zhao et al.2016	27703150	Tumor
chr13	79161580		RNF219-AS1	ncRNA_intronic	HIVID	Zhao et al.2016	27703150	Tumor
chr8	53661850		RB1CC1(dist=34824),NPBWR1(dist=190618)	intergenic	HIVID	Zhao et al.2016	27703150	Tumor
chr20	20441401		RALGAPA2	intronic	HIVID	Zhao et al.2016	27703150	Tumor

Supplementary Table S8 Continued

Chromosome	Integration site in host genome	Integration site in virus genome	Gene (distance, bp)	Regions	Methods	Author	PMID	Sample
chr17	29724590		RAB11FIP4	intronic	HIVID	Zhao et al.2016	27703150	Tumor
chr2	209271738		PTH2R	UTR5	HIVID	Zhao et al.2016	27703150	Tumor
chr8	128210882		PRNCR1(dist=106042),CCAT1(dist=8745)	intergenic	HIVID	Zhao et al.2016	27703150	Tumor
chr2	46185749		PRKCE	intronic	HIVID	Zhao et al.2016	27703150	Tumor
chr15	65154890		PLEKHO2	intronic	HIVID	Zhao et al.2016	27703150	Tumor
chr9	26928135		PLAA	exonic	HIVID	Zhao et al.2016	27703150	Tumor
chr7	106526831		PIK3CG	intronic	HIVID	Zhao et al.2016	27703150	Tumor
chr3	74117952		PDZRN3(dist=443880),CNTN3(dist=193770)	intergenic	HIVID	Zhao et al.2016	27703150	Tumor
chr2	178557846		PDE11A	intronic	HIVID	Zhao et al.2016	27703150	Tumor
chr5	50083842		PARP8	intronic	HIVID	Zhao et al.2016	27703150	Tumor
chr2	206153617		PARD3B	intronic	HIVID	Zhao et al.2016	27703150	Tumor
chr20	9785586		PAK7	intronic	HIVID	Zhao et al.2016	27703150	Tumor
chr11	74844975		OR2AT4(dist=44217),SLCO2B1(dist=17057)	intergenic	HIVID	Zhao et al.2016	27703150	Tumor
chr8	54184801		OPRK1(dist=20544),ATP6V1H(dist=443302)	intergenic	HIVID	Zhao et al.2016	27703150	Tumor
chr1	228575165		OBSCN(dist=8590),TRIM11(dist=6212)	intergenic	HIVID	Zhao et al.2016	27703150	Tumor
chr10	84099494		NRG3	intronic	HIVID	Zhao et al.2016	27703150	Tumor
chr1	52310539		NRD1	intronic	HIVID	Zhao et al.2016	27703150	Tumor
chr10	42385969		NONE(dist=NONE),LOC441666(dist=441345)	intergenic	HIVID	Zhao et al.2016	27703150	Tumor
chr10	42394450		NONE(dist=NONE),LOC441666(dist=432864)	intergenic	HIVID	Zhao et al.2016	27703150	Tumor
chr10	42394747		NONE(dist=NONE),LOC441666(dist=432567)	intergenic	HIVID	Zhao et al.2016	27703150	Tumor
chr10	42394813		NONE(dist=NONE),LOC441666(dist=432501)	intergenic	HIVID	Zhao et al.2016	27703150	Tumor
chr10	42394892		NONE(dist=NONE),LOC441666(dist=432422)	intergenic	HIVID	Zhao et al.2016	27703150	Tumor
chr10	42396598		NONE(dist=NONE),LOC441666(dist=430716)	intergenic	HIVID	Zhao et al.2016	27703150	Tumor
chr10	42597045		NONE(dist=NONE),LOC441666(dist=230269)	intergenic	HIVID	Zhao et al.2016	27703150	Tumor
chr10	42599765		NONE(dist=NONE),LOC441666(dist=227549)	intergenic	HIVID	Zhao et al.2016	27703150	Tumor
chr8	47316183		NONE(dist=NONE),LINC00293(dist=436325)	intergenic	HIVID	Zhao et al.2016	27703150	Tumor
chr12	38443509		NONE(dist=NONE),ALG10B(dist=267048)	intergenic	HIVID	Zhao et al.2016	27703150	Tumor
chr7	106012264		NAMPT(dist=86626),CCDC71L(dist=284947)	intergenic	HIVID	Zhao et al.2016	27703150	Tumor
chr2	16467305		MYCN(dist=380176),FAM49A(dist=263425)	intergenic	HIVID	Zhao et al.2016	27703150	Tumor
chr21	42829423		MX1	intronic	HIVID	Zhao et al.2016	27703150	Tumor
chr8	143044044		MROH5(dist=526714),MIR4472-1(dist=213656)	intergenic	HIVID	Zhao et al.2016	27703150	Tumor
chr6	40010777		MOCS1(dist=108487),LINC00951(dist=301307)	intergenic	HIVID	Zhao et al.2016	27703150	Tumor
chr8	89252741		MMP16	intronic	HIVID	Zhao et al.2016	27703150	Tumor
chr10	130791308		MKI67(dist=866840),MGMT(dist=474146)	intergenic	HIVID	Zhao et al.2016	27703150	Tumor
chr5	95720247		MIR583(dist=305331),PCSK1(dist=5793)	intergenic	HIVID	Zhao et al.2016	27703150	Tumor
chr14	64894223		MIR548AZ	ncRNA_intronic	HIVID	Zhao et al.2016	27703150	Tumor
chr1	247383227		MIR3916(dist=17865),VN1R5(dist=36147)	intergenic	HIVID	Zhao et al.2016	27703150	Tumor
chr2	102459211		MAP4K4	intronic	HIVID	Zhao et al.2016	27703150	Tumor
chr19	34666761		LSM14A	intronic	HIVID	Zhao et al.2016	27703150	Tumor
chr18	44096674		LOXHD1	intronic	HIVID	Zhao et al.2016	27703150	Tumor
chr1	112797289		LOC643355(dist=255826),CTTNBP2NL(dist=141511)	intergenic	HIVID	Zhao et al.2016	27703150	Tumor
chr1	112797177		LOC643355(dist=255714),CTTNBP2NL(dist=141623)	intergenic	HIVID	Zhao et al.2016	27703150	Tumor
chr5	2465284		LOC100506858(dist=152969),IRX2(dist=280995)	intergenic	HIVID	Zhao et al.2016	27703150	Tumor
chr11	115986746		LINC00900(dist=355828),BUD13(dist=632140)	intergenic	HIVID	Zhao et al.2016	27703150	Tumor
chr19	28766864		LINC00662(dist=482016),LINC00906(dist=689174)	intergenic	HIVID	Zhao et al.2016	27703150	Tumor
chr8	58304158		LINC00588(dist=106868),FAM110B(dist=602955)	intergenic	HIVID	Zhao et al.2016	27703150	Tumor
chr3	177238196		LINC00578	ncRNA_intronic	HIVID	Zhao et al.2016	27703150	Tumor
chr3	177238265		LINC00578	ncRNA_intronic	HIVID	Zhao et al.2016	27703150	Tumor
chr3	156777742		LEKR1(dist=13824),LINC00880(dist=21714)	intergenic	HIVID	Zhao et al.2016	27703150	Tumor
chr18	21381775		LAMA3	intronic	HIVID	Zhao et al.2016	27703150	Tumor
chr2	143627932		KYNU	promoter	HIVID	Zhao et al.2016	27703150	Tumor
chr2	143627981		KYNU	promoter	HIVID	Zhao et al.2016	27703150	Tumor
chr3	122233061		KPNA1	intronic	HIVID	Zhao et al.2016	27703150	Tumor
chr19	36212655		KMT2B	exonic	HIVID	Zhao et al.2016	27703150	Tumor
chr19	36213655		KMT2B	intronic	HIVID	Zhao et al.2016	27703150	Tumor
chr19	45850266		KLC3	intronic	HIVID	Zhao et al.2016	27703150	Tumor
chr5	155109232		KIF4B(dist=711547),SGCD(dist=644535)	intergenic	HIVID	Zhao et al.2016	27703150	Tumor
chr5	155109206		KIF4B(dist=711521),SGCD(dist=644561)	intergenic	HIVID	Zhao et al.2016	27703150	Tumor
chr5	155109183		KIF4B(dist=711498),SGCD(dist=644584)	intergenic	HIVID	Zhao et al.2016	27703150	Tumor
chr15	79832240		KIAA1024(dist=67598),ST20-MTHFS(dist=303649)	intergenic	HIVID	Zhao et al.2016	27703150	Tumor
chr5	5655856		KIAA0947(dist=165509),FLJ33360(dist=654698)	intergenic	HIVID	Zhao et al.2016	27703150	Tumor
chr8	137822152		KHDRBS3(dist=1162304),FAM135B(dist=1320114)	intergenic	HIVID	Zhao et al.2016	27703150	Tumor
chr4	44521677		KCTD8(dist=70853),YIPF7(dist=102677)	intergenic	HIVID	Zhao et al.2016	27703150	Tumor
chr1	154723169		KCNN3	intronic	HIVID	Zhao et al.2016	27703150	Tumor
chr21	39288963		KCNJ6	promoter	HIVID	Zhao et al.2016	27703150	Tumor
chr3	123966030		KALRN	intronic	HIVID	Zhao et al.2016	27703150	Tumor
chr2	102666324		IL1R2(dist=21440),IL1R1(dist=20512)	intergenic	HIVID	Zhao et al.2016	27703150	Tumor
chr2	102697328		IL1R1	intronic	HIVID	Zhao et al.2016	27703150	Tumor
chr3	118761453		IGSF11	intronic	HIVID	Zhao et al.2016	27703150	Tumor
chr4	58148353		IGFBP7-AS1(dist=76888),NONE(dist=NONE)	intergenic	HIVID	Zhao et al.2016	27703150	Tumor
chr4	58148304		IGFBP7-AS1(dist=76839),NONE(dist=NONE)	intergenic	HIVID	Zhao et al.2016	27703150	Tumor
chr4	59505688		IGFBP7-AS1(dist=1434223),NONE(dist=NONE)	intergenic	HIVID	Zhao et al.2016	27703150	Tumor
chr16	1653196		IFT140	intronic	HIVID	Zhao et al.2016	27703150	Tumor
chr22	30518599		HORMAD2	intronic	HIVID	Zhao et al.2016	27703150	Tumor
chr6	125628214		HDDC2	promoter	HIVID	Zhao et al.2016	27703150	Tumor
chr2	240524844		HDAC4(dist=202201),LOC150935(dist=159710)	intergenic	HIVID	Zhao et al.2016	27703150	Tumor
chr5	90560480		GPR98(dist=100447),LUCAT1(dist=38323)	intergenic	HIVID	Zhao et al.2016	27703150	Tumor
chr4	47448911		GABRB1(dist=20464),COMMD8(dist=3900)	intergenic	HIVID	Zhao et al.2016	27703150	Tumor
chr16	86741424		FOXL1(dist=126120),RP11-178L8.5(dist=564088)	intergenic	HIVID	Zhao et al.2016	27703150	Tumor
chr19	17873497		FCHO1	intronic	HIVID	Zhao et al.2016	27703150	Tumor
chr3	75502954		FAM86DP(dist=18688),MIR1324(dist=176960)	intergenic	HIVID	Zhao et al.2016	27703150	Tumor
chr8	128005677		FAM84B(dist=434966),PCAT1(dist=19722)	intergenic	HIVID	Zhao et al.2016	27703150	Tumor
chr1	166557166		FAM78B(dist=421208),FMO9P(dist=15987)	intergenic	HIVID	Zhao et al.2016	27703150	Tumor
chr11	73132018		FAM168A	intronic	HIVID	Zhao et al.2016	27703150	Tumor
chr22	45720437		FAM118A	intronic	HIVID	Zhao et al.2016	27703150	Tumor
chr6	131179416		EPB41L2	intronic	HIVID	Zhao et al.2016	27703150	Tumor
chr20	2733385		EBF4	intronic	HIVID	Zhao et al.2016	27703150	Tumor
chr1	172331722		DNM3	intronic	HIVID	Zhao et al.2016	27703150	Tumor
chr11	109117199		DDX10(dist=305542),C11orf87(dist=175647)	intergenic	HIVID	Zhao et al.2016	27703150	Tumor
chr2	3848849		DCDC2C	intronic	HIVID	Zhao et al.2016	27703150	Tumor
chr3	39307395		CX3CR1	exonic	HIVID	Zhao et al.2016	27703150	Tumor
chr17	56033481		CUEDC1	promoter	HIVID	Zhao et al.2016	27703150	Tumor
chr5	1966502		CTD-2194D22.4(dist=65898),MIR548BA(dist=17527)	intergenic	HIVID	Zhao et al.2016	27703150	Tumor
chr1	17253724		CROCC	intronic	HIVID	Zhao et al.2016	27703150	Tumor

Chromosome	Integration site in host genome	Integration site in virus genome	Gene (distance, bp)	Regions	Methods	Author	PMID	Sample
chr2	238270974		COL6A3	intronic	HIVID	Zhao et al.2016	27703150	Tumor
chr13	100346671		CLYBL	intronic	HIVID	Zhao et al.2016	27703150	Tumor
chr18	12990743		CEP192;SEH1L	promoter;downstream	HIVID	Zhao et al.2016	27703150	Tumor
chr1	214778733		CENPF	intronic	HIVID	Zhao et al.2016	27703150	Tumor
chr16	80826756		CDYL2	intronic	HIVID	Zhao et al.2016	27703150	Tumor
chr9	21969230		CDKN2A	intronic	HIVID	Zhao et al.2016	27703150	Tumor
chr3	107822862		CD47(dist=12927),LINC01215(dist=21049)	intergenic	HIVID	Zhao et al.2016	27703150	Tumor
chr16	89075320		CBFA2T3(dist=31816),ACSF3(dist=84897)	intergenic	HIVID	Zhao et al.2016	27703150	Tumor
chr18	20843501		CABLES1(dist=3067),TMEM241(dist=32478)	intergenic	HIVID	Zhao et al.2016	27703150	Tumor
chr11	68029550		C11orf24	exonic	HIVID	Zhao et al.2016	27703150	Tumor
chr6	38143506		BTBD9	intronic	HIVID	Zhao et al.2016	27703150	Tumor
chr1	55468295		BSND	intronic	HIVID	Zhao et al.2016	27703150	Tumor
chr1	55468189		BSND	intronic	HIVID	Zhao et al.2016	27703150	Tumor
chr17	31545475		ASIC2	intronic	HIVID	Zhao et al.2016	27703150	Tumor
chr1	27076734		ARID1A	intronic	HIVID	Zhao et al.2016	27703150	Tumor
chr12	34368013		ALG10(dist=186777),NONE(dist=NONE)	intergenic	HIVID	Zhao et al.2016	27703150	Tumor
chr21	21618088		AL109763.2(dist=1485958),LINC00320(dist=496820)	intergenic	HIVID	Zhao et al.2016	27703150	Tumor
chr16	53626934		AKTIP(dist=89764),RPGRIP1L(dist=6884)	intergenic	HIVID	Zhao et al.2016	27703150	Tumor
chr10	5065439		AKR1C2	promoter	HIVID	Zhao et al.2016	27703150	Tumor
chr3	123064670		ADCY5	intronic	HIVID	Zhao et al.2016	27703150	Tumor
chr2	92269763		ACTR3BP2(dist=139267),NONE(dist=NONE)	intergenic	HIVID	Zhao et al.2016	27703150	Tumor
chr2	92269646		ACTR3BP2(dist=139150),NONE(dist=NONE)	intergenic	HIVID	Zhao et al.2016	27703150	Tumor
chr2	5560697		AC022311.1(dist=856885),SOX11(dist=272102)	intergenic	HIVID	Zhao et al.2016	27703150	Tumor
chr2	196428405		AC006196.1(dist=802246),SLC39A10(dist=93127)	intergenic	HIVID	Zhao et al.2016	27703150	Tumor
chr17	1020572		ABR	intronic	HIVID	Zhao et al.2016	27703150	Tumor
chr7	87089249		ABCB4	intronic	HIVID	Zhao et al.2016	27703150	Tumor
chr1	94496253		ABCA4	intronic	HIVID	Zhao et al.2016	27703150	Tumor
chr16	87459177		ZCCHC14	intronic	HIVID	Zhao et al.2016	27703150	Tumor
chr4	4342804		ZBTB49(dist=19291),NSG1(dist=45179)	intergenic	HIVID	Zhao et al.2016	27703150	Tumor
chr15	55266167		UNC13C(dist=345361),RSL24D1(dist=207345)	intergenic	HIVID	Zhao et al.2016	27703150	Tumor
chr10	5425239		UCN3(dist=9070),TUBAL3(dist=9822)	intergenic	HIVID	Zhao et al.2016	27703150	Tumor
chr11	87810192		TMEM135(dist=770316),RAB38(dist=36223)	intergenic	HIVID	Zhao et al.2016	27703150	Tumor
chr11	87810130		TMEM135(dist=770254),RAB38(dist=36285)	intergenic	HIVID	Zhao et al.2016	27703150	Tumor
chr19	1707132		TCF3(dist=54804),ONECUT3(dist=46530)	intergenic	HIVID	Zhao et al.2016	27703150	Tumor
chr20	24559069		SYNDIG1	intronic	HIVID	Zhao et al.2016	27703150	Tumor
chr8	70951130		SLCO5A1(dist=203831),PRDM14(dist=12756)	intergenic	HIVID	Zhao et al.2016	27703150	Tumor
chr8	70951075		SLCO5A1(dist=203776),PRDM14(dist=12811)	intergenic	HIVID	Zhao et al.2016	27703150	Tumor
chr8	70951043		SLCO5A1(dist=203744),PRDM14(dist=12843)	intergenic	HIVID	Zhao et al.2016	27703150	Tumor
chr5	512163		SLC9A3	intronic	HIVID	Zhao et al.2016	27703150	Tumor
chr14	37603853		SLC25A21	intronic	HIVID	Zhao et al.2016	27703150	Tumor
chr4	1708958		SLBP	intronic	HIVID	Zhao et al.2016	27703150	Tumor
chr4	1709026		SLBP	intronic	HIVID	Zhao et al.2016	27703150	Tumor
chr21	45126132		RRP1B(dist=10172),PDXK(dist=12846)	intergenic	HIVID	Zhao et al.2016	27703150	Tumor
chr6	12685439		RNU6-48P(dist=292853),PHACTR1(dist=31598)	intergenic	HIVID	Zhao et al.2016	27703150	Tumor
chr19	43847135		PRG1	promoter	HIVID	Zhao et al.2016	27703150	Tumor
chr5	54808290		PPAP2A	intronic	HIVID	Zhao et al.2016	27703150	Tumor
chr17	79821060		P4HB;ARHGDIA	promoter;downstream	HIVID	Zhao et al.2016	27703150	Tumor
chr17	79817302		P4HB	intronic	HIVID	Zhao et al.2016	27703150	Tumor
chr3	190964387		OSTN	intronic	HIVID	Zhao et al.2016	27703150	Tumor
chr2	112147152		MIR4435-1HG	ncRNA_intronic	HIVID	Zhao et al.2016	27703150	Tumor
chr5	88960553		MEF2C-AS1(dist=632360),MIR3660(dist=351885)	intergenic	HIVID	Zhao et al.2016	27703150	Tumor
chr20	16036891		MACROD2(dist=3050),KIF16B(dist=215858)	intergenic	HIVID	Zhao et al.2016	27703150	Tumor
chr4	152004726		LRBA(dist=68077),RPS3A(dist=15999)	intergenic	HIVID	Zhao et al.2016	27703150	Tumor
chr17	44355042		LOC644172(dist=31888),LRRC37A(dist=17455)	intergenic	HIVID	Zhao et al.2016	27703150	Tumor
chr4	189779717		LINC01060(dist=256655),FRG1(dist=1082257)	intergenic	HIVID	Zhao et al.2016	27703150	Tumor
chr17	25846584		KSR1	intronic	HIVID	Zhao et al.2016	27703150	Tumor
chr17	25846597		KSR1	intronic	HIVID	Zhao et al.2016	27703150	Tumor
chr8	138057981		KHDRBS3(dist=1398133),FAM135B(dist=1084285)	intergenic	HIVID	Zhao et al.2016	27703150	Tumor
chr7	22819824		IL6(dist=48203),TOMM7(dist=32427)	intergenic	HIVID	Zhao et al.2016	27703150	Tumor
chr6	160521820		IGF2R	intronic	HIVID	Zhao et al.2016	27703150	Tumor
chr6	160521754		IGF2R	intronic	HIVID	Zhao et al.2016	27703150	Tumor
chr17	14458211		HS3ST3B1(dist=208719),CDRT7(dist=476081)	intergenic	HIVID	Zhao et al.2016	27703150	Tumor
chr17	13787417		HS3ST3A1(dist=282173),CDRT15P1(dist=140398)	intergenic	HIVID	Zhao et al.2016	27703150	Tumor
chr8	123334568		HAS2-AS1(dist=677004),ZHX2(dist=459333)	intergenic	HIVID	Zhao et al.2016	27703150	Tumor
chr8	123334499		HAS2-AS1(dist=676935),ZHX2(dist=459402)	intergenic	HIVID	Zhao et al.2016	27703150	Tumor
chr6	28499148		GPX5	intronic	HIVID	Zhao et al.2016	27703150	Tumor
chr9	4161079		GLIS3	intronic	HIVID	Zhao et al.2016	27703150	Tumor
chr6	17548589		CAP2	intronic	HIVID	Zhao et al.2016	27703150	Tumor
chr1	178627815		C1orf220(dist=109791),MIR4424(dist=19069)	intergenic	HIVID	Zhao et al.2016	27703150	Tumor
chr1	51673378		C1orf185(dist=59624),RNF11(dist=28567)	intergenic	HIVID	Zhao et al.2016	27703150	Tumor
chr10	103178795		BTRC	intronic	HIVID	Zhao et al.2016	27703150	Tumor
chr9	116109306		BSPRY,WDR31	promoter	HIVID	Zhao et al.2016	27703150	Tumor
chr11	129227201		ARHGAP32(dist=165108),BARX2(dist=18680)	intergenic	HIVID	Zhao et al.2016	27703150	Tumor
chr20	18708080		DTD1	intronic	HIVID	Zhao et al.2016	27703150	Tumor
chr12	10796485		STYK1	intronic	HIVID	Zhao et al.2016	27703150	Tumor
chr11	39286383		NONE(dist=NONE),LRRC4C(dist=849368)	intergenic	HIVID	Zhao et al.2016	27703150	Tumor
chr17	22257316		MTRNR2L1(dist=233325),NONE(dist=NONE)	intergenic	HIVID	Zhao et al.2016	27703150	Tumor
chr17	22250182		MTRNR2L1(dist=226191),NONE(dist=NONE)	intergenic	HIVID	Zhao et al.2016	27703150	Tumor
chr17	22245424		MTRNR2L1(dist=221433),NONE(dist=NONE)	intergenic	HIVID	Zhao et al.2016	27703150	Tumor
chr16	34252390		LINC00273(dist=289887),UBE2MP1(dist=151412)	intergenic	HIVID	Zhao et al.2016	27703150	Tumor
chr8	40749841		ZMAT4	intronic	HIVID	Zhao et al.2016	27703150	Tumor
chr8	59851036		TOX	intronic	HIVID	Zhao et al.2016	27703150	Tumor
chr12	57979584		PIP4K2C;KIF5A	promoter;downstream	HIVID	Zhao et al.2016	27703150	Tumor
chr7	151075013		NUB1	UTR3	HIVID	Zhao et al.2016	27703150	Tumor
chr7	151065491		NUB1	intronic	HIVID	Zhao et al.2016	27703150	Tumor
chr19	27732135		NONE(dist=NONE),LINC00662(dist=549266)	intergenic	HIVID	Zhao et al.2016	27703150	Tumor
chr19	27733824		NONE(dist=NONE),LINC00662(dist=547577)	intergenic	HIVID	Zhao et al.2016	27703150	Tumor
chr19	27736532		NONE(dist=NONE),LINC00662(dist=544869)	intergenic	HIVID	Zhao et al.2016	27703150	Tumor
chr19	27737226		NONE(dist=NONE),LINC00662(dist=544175)	intergenic	HIVID	Zhao et al.2016	27703150	Tumor
chr19	27739261		NONE(dist=NONE),LINC00662(dist=542140)	intergenic	HIVID	Zhao et al.2016	27703150	Tumor
chr11	24329290		MIR8054(dist=888554),LUZP2(dist=189226)	intergenic	HIVID	Zhao et al.2016	27703150	Tumor
chr4	165534792		MIR5684	ncRNA_intronic	HIVID	Zhao et al.2016	27703150	Tumor
chr21	9826399		MIR3687(dist=136),TEKT4P2(dist=80790)	intergenic	HIVID	Zhao et al.2016	27703150	Tumor
chr1	121485153		EMBP1(dist=171467),NONE(dist=NONE)	intergenic	HIVID	Zhao et al.2016	27703150	Tumor

Supplementary Table S8 Continued

Chromosome	Integration site in host genome	Integration site in virus genome	Gene (distance, bp)	Regions	Methods	Author	PMID	Sample
chr1	121484126		EMBP1(dist=170440),NONE(dist=NONE)	intergenic	HIVID	Zhao et al.2016	27703150	Tumor
chr21	10777747		TEKT4P2(dist=809154),TPTE(dist=128996)	intergenic	HIVID	Zhao et al.2016	27703150	Tumor
chr21	10777668		TEKT4P2(dist=809075),TPTE(dist=129075)	intergenic	HIVID	Zhao et al.2016	27703150	Tumor
chr17	19942615		SPECC1	intronic	HIVID	Zhao et al.2016	27703150	Tumor
chr3	118716678		IGSF11	intronic	HIVID	Zhao et al.2016	27703150	Tumor
chr3	118716654		IGSF11	intronic	HIVID	Zhao et al.2016	27703150	Tumor
chr9	20922208		FOCAD	intronic	HIVID	Zhao et al.2016	27703150	Tumor
chr10	39146628		ACTR3BP5(dist=155257),NONE(dist=NONE)	intergenic	HIVID	Zhao et al.2016	27703150	Tumor
chr8	35473157		UNC5D	intronic	HIVID	Zhao et al.2016	27703150	Tumor
chr8	35312131		UNC5D	intronic	HIVID	Zhao et al.2016	27703150	Tumor
chr5	104774588		RAB9BP1(dist=338789),LOC102467213(dist=1376310)	intergenic	HIVID	Zhao et al.2016	27703150	Tumor
chr16	49311655		N4BP1(dist=667535),CBLN1(dist=174)	intergenic	HIVID	Zhao et al.2016	27703150	Tumor
chr4	78776129		MRPL1	promoter	HIVID	Zhao et al.2016	27703150	Tumor
chr4	78808871		MRPL1	intronic	HIVID	Zhao et al.2016	27703150	Tumor
chr5	3404046		LOC102467074(dist=222700),LINC01019(dist=13220)	intergenic	HIVID	Zhao et al.2016	27703150	Tumor
chr5	1297513		TERT	promoter	HIVID	Zhao et al.2016	27703150	Tumor
chr5	11580		NONE(dist=NONE),PLEKHG4B(dist=128793)	intergenic	HIVID	Zhao et al.2016	27703150	Tumor
chr5	11682		NONE(dist=NONE),PLEKHG4B(dist=128691)	intergenic	HIVID	Zhao et al.2016	27703150	Tumor
chr5	11705		NONE(dist=NONE),PLEKHG4B(dist=128668)	intergenic	HIVID	Zhao et al.2016	27703150	Tumor
chr5	11805		NONE(dist=NONE),PLEKHG4B(dist=128568)	intergenic	HIVID	Zhao et al.2016	27703150	Tumor
chr7	10013		NONE(dist=NONE),LOC100507642(dist=139705)	intergenic	HIVID	Zhao et al.2016	27703150	Tumor
chr14	45779936		MIS18BP1(dist=57331),LINC00871(dist=753426)	intergenic	HIVID	Zhao et al.2016	27703150	Tumor
chr7	28020749		JAZF1	intronic	HIVID	Zhao et al.2016	27703150	Tumor
chr7	30939100		INMT-FAM188B(dist=7098),AQP1(dist=12315)	intergenic	HIVID	Zhao et al.2016	27703150	Tumor
chr13	94258442		GPC6	intronic	HIVID	Zhao et al.2016	27703150	Tumor
chr13	94093647		GPC6	intronic	HIVID	Zhao et al.2016	27703150	Tumor
chr3	197900339		FAM157A	intronic	HIVID	Zhao et al.2016	27703150	Tumor
chr4	191044274		DUX4(dist=30832),NONE(dist=NONE)	intergenic	HIVID	Zhao et al.2016	27703150	Tumor
chr15	102521208		DDX11L9;MIR6859-1,MIR6859-2,WASH3P	promoter;downstream	HIVID	Zhao et al.2016	27703150	Tumor
chr9	10351		DDX11L5;WASH1	promoter;downstream	HIVID	Zhao et al.2016	27703150	Tumor
chr1	10333		DDX11L1;MIR6859-1,MIR6859-2,WASH7P	promoter;downstream	HIVID	Zhao et al.2016	27703150	Tumor
chr2	137674999		CXCR4(dist=799274),THSD7B(dist=73463)	intergenic	HIVID	Zhao et al.2016	27703150	Tumor
chr10	11500358		CELF2(dist=121686),USP6NL(dist=2151)	intergenic	HIVID	Zhao et al.2016	27703150	Tumor
chr17	45266661		CDC27	UTR5	HIVID	Zhao et al.2016	27703150	Tumor
chr3	147691852		ZIC1(dist=557346),AGTR1(dist=723806)	intergenic	HIVID	Zhao et al.2016	27703150	Tumor
chr16	79192401		WWOX	intronic	HIVID	Zhao et al.2016	27703150	Tumor
chr5	1297446		TERT	promoter	HIVID	Zhao et al.2016	27703150	Tumor
chr5	1297581		TERT	promoter	HIVID	Zhao et al.2016	27703150	Tumor
chr11	51589085		OR4C46(dist=72874),NONE(dist=NONE)	intergenic	HIVID	Zhao et al.2016	27703150	Tumor
chr11	51587038		OR4C46(dist=70827),NONE(dist=NONE)	intergenic	HIVID	Zhao et al.2016	27703150	Tumor
chr7	158866062		VIPR2	intronic	HIVID	Zhao et al.2016	27703150	Tumor
chr7	158862322		VIPR2	intronic	HIVID	Zhao et al.2016	27703150	Tumor
chr1	219239334		TGFB2(dist=621373),RP11-135J2.4(dist=14983)	intergenic	HIVID	Zhao et al.2016	27703150	Tumor
chr1	219222896		TGFB2(dist=604935),RP11-135J2.4(dist=31421)	intergenic	HIVID	Zhao et al.2016	27703150	Tumor
chr5	1296031		TERT	promoter	HIVID	Zhao et al.2016	27703150	Tumor
chr5	1296061		TERT	promoter	HIVID	Zhao et al.2016	27703150	Tumor
chr6	150591703		PPP1R14C(dist=20175),IYD(dist=98325)	intergenic	HIVID	Zhao et al.2016	27703150	Tumor
chr12	62893835		MON2	intronic	HIVID	Zhao et al.2016	27703150	Tumor
chr8	56922889		LYN	UTR3	HIVID	Zhao et al.2016	27703150	Tumor
chr4	1709026		SLBP	intronic	HIVID	Zhao et al.2016	27703150	Tumor
chr15	77910867		LINGO1	intronic	HIVID	Zhao et al.2016	27703150	Tumor
chr2	233197922		DIS3L2	intronic	HIVID	Zhao et al.2016	27703150	Tumor
chr3	182581698		ATP11B	intronic	HIVID	Zhao et al.2016	27703150	Tumor
chr5	1295188		TERT	promoter	HIVID	Zhao et al.2016	27703150	Tumor
chr5	1295136		TERT	UTR5	HIVID	Zhao et al.2016	27703150	Tumor
chr5	1295219		TERT	promoter	HIVID	Zhao et al.2016	27703150	Tumor
chr7	129165573		SMKR1(dist=12800),NRF1(dist=85982)	intergenic	HIVID	Zhao et al.2016	27703150	Tumor
chr3	78621398		ROBO2(dist=922284),ROBO1(dist=24990)	intergenic	HIVID	Zhao et al.2016	27703150	Tumor
chr4	61764666		NONE(dist=NONE),LPHN3(dist=598173)	intergenic	HIVID	Zhao et al.2016	27703150	Tumor
chr10	42385005		NONE(dist=NONE),LOC441666(dist=442309)	intergenic	HIVID	Zhao et al.2016	27703150	Tumor
chr10	42388389		NONE(dist=NONE),LOC441666(dist=438925)	intergenic	HIVID	Zhao et al.2016	27703150	Tumor
chr10	42393732		NONE(dist=NONE),LOC441666(dist=433582)	intergenic	HIVID	Zhao et al.2016	27703150	Tumor
chr1	149035435		NBPF25P	ncRNA_intronic	HIVID	Zhao et al.2016	27703150	Tumor
chr10	38881754		LINC00999(dist=140673),ACTR3BP5(dist=107973)	intergenic	HIVID	Zhao et al.2016	27703150	Tumor
chr10	94750724		EXOC6	intronic	HIVID	Zhao et al.2016	27703150	Tumor
chr5	1333633		CLPTM1L	intronic	HIVID	Zhao et al.2016	27703150	Tumor
chr5	1333526		CLPTM1L	intronic	HIVID	Zhao et al.2016	27703150	Tumor
chr16	88620350		ZFPM1(dist=18776),ZC3H18(dist=16439)	intergenic	HIVID	Zhao et al.2016	27703150	Tumor
chr12	20264192		RP11-664H17.1(dist=12390),PDE3A(dist=257987)	intergenic	HIVID	Zhao et al.2016	27703150	Tumor
chr9	136106946		OBP2B(dist=22309),ABO(dist=23617)	intergenic	HIVID	Zhao et al.2016	27703150	Tumor
chr5	11581		NONE(dist=NONE),PLEKHG4B(dist=128792)	intergenic	HIVID	Zhao et al.2016	27703150	Tumor
chr5	11684		NONE(dist=NONE),PLEKHG4B(dist=128689)	intergenic	HIVID	Zhao et al.2016	27703150	Tumor
chr5	11702		NONE(dist=NONE),PLEKHG4B(dist=128671)	intergenic	HIVID	Zhao et al.2016	27703150	Tumor
chr2	234727497		MROH2A	intronic	HIVID	Zhao et al.2016	27703150	Tumor
chr2	243152576		LOC728323(dist=50107),NONE(dist=NONE)	intergenic	HIVID	Zhao et al.2016	27703150	Tumor
chr12	95641		LOC100288778(dist=4378),FAM138D(dist=52305)	intergenic	HIVID	Zhao et al.2016	27703150	Tumor
chr12	95635		LOC100288778(dist=4372),FAM138D(dist=52311)	intergenic	HIVID	Zhao et al.2016	27703150	Tumor
chr12	95437		LOC100288778(dist=4174),FAM138D(dist=52509)	intergenic	HIVID	Zhao et al.2016	27703150	Tumor
chr4	191044165		DUX4(dist=30723),NONE(dist=NONE)	intergenic	HIVID	Zhao et al.2016	27703150	Tumor
chr1	34838619		C1orf94(dist=153888),GJB5(dist=382029)	intergenic	HIVID	Zhao et al.2016	27703150	Tumor
chr17	51305297		C17orf112(dist=240285),KIF2B(dist=594942)	intergenic	HIVID	Zhao et al.2016	27703150	Tumor
chr19	4517611		PLIN4	exonic	HIVID	Zhao et al.2016	27703150	Tumor
chr19	4515320		PLIN4	intronic	HIVID	Zhao et al.2016	27703150	Tumor
chr5	171567872		STK10	intronic	HIVID	Zhao et al.2016	27703150	Tumor
chr1	249240404		PGBD2(dist=27059),NONE(dist=NONE)	intergenic	HIVID	Zhao et al.2016	27703150	Tumor
chr3	192631852		MB21D2	intronic	HIVID	Zhao et al.2016	27703150	Tumor
chr3	192600976		MB21D2	intronic	HIVID	Zhao et al.2016	27703150	Tumor
chr2	243152855		LOC728323(dist=50386),NONE(dist=NONE)	intergenic	HIVID	Zhao et al.2016	27703150	Tumor
chr19	36212800		KMT2B	intronic	HIVID	Zhao et al.2016	27703150	Tumor
chr19	36212735		KMT2B	intronic	HIVID	Zhao et al.2016	27703150	Tumor
chr19	36212819		KMT2B	intronic	HIVID	Zhao et al.2016	27703150	Tumor
chr10	135524236		DUX4L7(dist=25778),NONE(dist=NONE)	intergenic	HIVID	Zhao et al.2016	27703150	Tumor
chr1	10283		DDX11L1;MIR6859-1,MIR6859-2,WASH7P	promoter;downstream	HIVID	Zhao et al.2016	27703150	Tumor
chr8	50676061		C8orf22(dist=687419),SNTG1(dist=146288)	intergenic	HIVID	Zhao et al.2016	27703150	Tumor
chr16	33959484		RNU6-76P(dist=396241),LINC00273(dist=1568)	intergenic	HIVID	Zhao et al.2016	27703150	Tumor
chr17	22250317		MTRNR2L1(dist=226326),NONE(dist=NONE)	intergenic	HIVID	Zhao et al.2016	27703150	Tumor
chr17	22247938		MTRNR2L1(dist=223947),NONE(dist=NONE)	intergenic	HIVID	Zhao et al.2016	27703150	Tumor
chr22	35501134		ISX(dist=17754),HMGXB4(dist=152311)	intergenic	HIVID	Zhao et al.2016	27703150	Tumor
chr5	11349649		CTNND2	intronic	HIVID	Zhao et al.2016	27703150	Tumor
chr16	27069688		C16orf82	promoter	HIVID	Zhao et al.2016	27703150	Tumor
chr16	27071445		C16orf82	promoter	HIVID	Zhao et al.2016	27703150	Tumor
chr2	133023215		ANKRD30BL,MIR663B	promoter	HIVID	Zhao et al.2016	27703150	Tumor
chr2	133023284		ANKRD30BL,MIR663B	promoter	HIVID	Zhao et al.2016	27703150	Tumor
chr5	1295178		TERT	promoter	HIVID	Zhao et al.2016	27703150	Tumor
chr22	25000901		GGT1	intronic	HIVID	Zhao et al.2016	27703150	Tumor
chr9	101805494		COL15A1	intronic	HIVID	Zhao et al.2016	27703150	Tumor

Supplementary Table S8 Continued

Chromosome	Integration site in host genome	Integration site in virus genome	Gene (distance, bp)	Regions	Methods	Author	PMID	Sample
chr12	69774315		YEATS4	intronic	HIVID	Zhao et al.2016	27703150	Tumor
chr6	54536306		TINAG(dist=281356),FAM83B(dist=175263)	intergenic	HIVID	Zhao et al.2016	27703150	Tumor
chr5	1085343		SLC12A7	intronic	HIVID	Zhao et al.2016	27703150	Tumor
chr7	155961132		SHH(dist=356165),AC073133.1(dist=269351)	intergenic	HIVID	Zhao et al.2016	27703150	Tumor
chr5	162944407		MAT2B	intronic	HIVID	Zhao et al.2016	27703150	Tumor
chr5	162944403		MAT2B	intronic	HIVID	Zhao et al.2016	27703150	Tumor
chr1	59828049		FGGY	intronic	HIVID	Zhao et al.2016	27703150	Tumor
chr7	146432080		CNTNAP2	intronic	HIVID	Zhao et al.2016	27703150	Tumor
chr7	146432112		CNTNAP2	intronic	HIVID	Zhao et al.2016	27703150	Tumor
chr3	33559198		CLASP2	intronic	HIVID	Zhao et al.2016	27703150	Tumor
chr3	62759879		CADPS	intronic	HIVID	Zhao et al.2016	27703150	Tumor
chr8	82861144		SNX16(dist=106623),NONE(dist=NONE)	intergenic	HIVID	Zhao et al.2016	27703150	Tumor
chr5	1295275		TERT	promoter	HIVID	Zhao et al.2016	27703150	Tumor
chr5	1295245		TERT	promoter	HIVID	Zhao et al.2016	27703150	Tumor
chr1	64597273		ROR1	intronic	HIVID	Zhao et al.2016	27703150	Tumor
chr8	91913978		NECAB1	intronic	HIVID	Zhao et al.2016	27703150	Tumor
chr7	76943592		GSAP	intronic	HIVID	Zhao et al.2016	27703150	Tumor
chr19	12100466		ZNF763(dist=9268),ZNF433(dist=25066)	intergenic	HIVID	Zhao et al.2016	27703150	Tumor
chr19	12100435		ZNF763(dist=9237),ZNF433(dist=25097)	intergenic	HIVID	Zhao et al.2016	27703150	Tumor
chr3	147826592		ZIC1(dist=692086),AGTR1(dist=589066)	intergenic	HIVID	Zhao et al.2016	27703150	Tumor
chr10	106508859		SORCS3	intronic	HIVID	Zhao et al.2016	27703150	Tumor
chr16	33955267		RNU6-76P(dist=392024),LINC00273(dist=5785)	intergenic	HIVID	Zhao et al.2016	27703150	Tumor
chr8	52714185		PXDNL	intronic	HIVID	Zhao et al.2016	27703150	Tumor
chr3	174410037		NLGN1(dist=408898),NAALADL2(dist=167074)	intergenic	HIVID	Zhao et al.2016	27703150	Tumor
chr3	174409997		NLGN1(dist=408858),NAALADL2(dist=167114)	intergenic	HIVID	Zhao et al.2016	27703150	Tumor
chr8	127475540		LINC00861(dist=512099),FAM84B(dist=89143)	intergenic	HIVID	Zhao et al.2016	27703150	Tumor
chr1	91853149		HFM1	intronic	HIVID	Zhao et al.2016	27703150	Tumor
chr18	51063558		DCC(dist=1285),MBD2(dist=614413)	intergenic	HIVID	Zhao et al.2016	27703150	Tumor
chr15	37020299		C15orf41	intronic	HIVID	Zhao et al.2016	27703150	Tumor
chr15	37020128		C15orf41	intronic	HIVID	Zhao et al.2016	27703150	Tumor
chr2	133028370		ANKRD30BL(dist=12828),GPR39(dist=145777)	intergenic	HIVID	Zhao et al.2016	27703150	Tumor
chr17	20774732		RP11-344E13.3	ncRNA_intronic	HIVID	Zhao et al.2016	27703150	Tumor
chr11	51588979		OR4C46(dist=72768),NONE(dist=NONE)	intergenic	HIVID	Zhao et al.2016	27703150	Tumor
chr11	51586932		OR4C46(dist=70721),NONE(dist=NONE)	intergenic	HIVID	Zhao et al.2016	27703150	Tumor
chr18	18519828		NONE(dist=NONE),ROCK1(dist=9875)	intergenic	HIVID	Zhao et al.2016	27703150	Tumor
chr18	18519140		NONE(dist=NONE),ROCK1(dist=10563)	intergenic	HIVID	Zhao et al.2016	27703150	Tumor
chr20	60916222		LAMA5	intronic	HIVID	Zhao et al.2016	27703150	Tumor
chr20	60916112		LAMA5	intronic	HIVID	Zhao et al.2016	27703150	Tumor
chr19	36212428		KMT2B	exonic	HIVID	Zhao et al.2016	27703150	Tumor
chr19	36212579		KMT2B	exonic	HIVID	Zhao et al.2016	27703150	Tumor
chr16	22843401		HS3ST2	intronic	HIVID	Zhao et al.2016	27703150	Tumor
chr16	22843369		HS3ST2	intronic	HIVID	Zhao et al.2016	27703150	Tumor
chr3	135023399		EPHB1(dist=44092),PPP2R3A(dist=661116)	intergenic	HIVID	Zhao et al.2016	27703150	Tumor
chr8	67396281		C8orf46	promoter	HIVID	Zhao et al.2016	27703150	Tumor
chr8	67396612		C8orf46	promoter	HIVID	Zhao et al.2016	27703150	Tumor
chr8	67396277		C8orf46	promoter	HIVID	Zhao et al.2016	27703150	Tumor
chr16	53216803		CHD9	intronic	HIVID	Zhao et al.2016	27703150	Tumor
chr19	21746298		ZNF429(dist=25219),ZNF100(dist=160545)	intergenic	HIVID	Zhao et al.2016	27703150	Tumor
chr19	21746215		ZNF429(dist=25136),ZNF100(dist=160628)	intergenic	HIVID	Zhao et al.2016	27703150	Tumor
chr19	21746188		ZNF429(dist=25109),ZNF100(dist=160655)	intergenic	HIVID	Zhao et al.2016	27703150	Tumor
chr17	15467181		TVP23C,TVP23C-CDRT4;CDRT1	promoter;downstream	HIVID	Zhao et al.2016	27703150	Tumor
chr17	18684279		TVP23B;FBXW10	promoter;downstream	HIVID	Zhao et al.2016	27703150	Tumor
chr5	1298413		TERT	promoter	HIVID	Zhao et al.2016	27703150	Tumor
chr5	1295987		TERT	promoter	HIVID	Zhao et al.2016	27703150	Tumor
chr5	1298438		TERT	promoter	HIVID	Zhao et al.2016	27703150	Tumor
chr9	66589698		PTGER4P2-CDK2AP2P2(dist=86668),RP11-381O7.3(dist=42767[illegible]	intergenic	HIVID	Zhao et al.2016	27703150	Tumor
chr8	78989491		PEX2(dist=1076211),PKIA(dist=438845)	intergenic	HIVID	Zhao et al.2016	27703150	Tumor
chr11	124162764		OR8G5(dist=27001),OR8D1(dist=16972)	intergenic	HIVID	Zhao et al.2016	27703150	Tumor
chr7	61861178		NONE(dist=NONE),ZNF733P(dist=890492)	intergenic	HIVID	Zhao et al.2016	27703150	Tumor
chr10	42386985		NONE(dist=NONE),LOC441666(dist=440329)	intergenic	HIVID	Zhao et al.2016	27703150	Tumor
chr16	46407678		NONE(dist=NONE),ANKRD26P1(dist=95571)	intergenic	HIVID	Zhao et al.2016	27703150	Tumor
chr16	46422016		NONE(dist=NONE),ANKRD26P1(dist=81233)	intergenic	HIVID	Zhao et al.2016	27703150	Tumor
chr16	46434061		NONE(dist=NONE),ANKRD26P1(dist=69188)	intergenic	HIVID	Zhao et al.2016	27703150	Tumor
chr16	46389725		NONE(dist=NONE),ANKRD26P1(dist=113524)	intergenic	HIVID	Zhao et al.2016	27703150	Tumor
chr16	46389768		NONE(dist=NONE),ANKRD26P1(dist=113481)	intergenic	HIVID	Zhao et al.2016	27703150	Tumor
chr16	46389827		NONE(dist=NONE),ANKRD26P1(dist=113422)	intergenic	HIVID	Zhao et al.2016	27703150	Tumor
chr16	46395758		NONE(dist=NONE),ANKRD26P1(dist=107491)	intergenic	HIVID	Zhao et al.2016	27703150	Tumor
chr16	46401464		NONE(dist=NONE),ANKRD26P1(dist=101785)	intergenic	HIVID	Zhao et al.2016	27703150	Tumor
chr16	46401486		NONE(dist=NONE),ANKRD26P1(dist=101763)	intergenic	HIVID	Zhao et al.2016	27703150	Tumor
chr16	46401507		NONE(dist=NONE),ANKRD26P1(dist=101742)	intergenic	HIVID	Zhao et al.2016	27703150	Tumor
chr16	46401566		NONE(dist=NONE),ANKRD26P1(dist=101683)	intergenic	HIVID	Zhao et al.2016	27703150	Tumor
chr6	141060242		MIR4465(dist=55222),NMBR(dist=1336503)	intergenic	HIVID	Zhao et al.2016	27703150	Tumor
chr16	32500026		LOC390705(dist=198724),TP53TG3B(dist=184823)	intergenic	HIVID	Zhao et al.2016	27703150	Tumor
chr3	163053339		LINC01192(dist=32250),SI(dist=1643347)	intergenic	HIVID	Zhao et al.2016	27703150	Tumor
chr4	79201141		FRAS1	intronic	HIVID	Zhao et al.2016	27703150	Tumor
chr9	1216500		DMRT2(dist=158946),SMARCA2(dist=798719)	intergenic	HIVID	Zhao et al.2016	27703150	Tumor
chr9	44209411		CNTNAP3B(dist=286938),LINC01189(dist=177976)	intergenic	HIVID	Zhao et al.2016	27703150	Tumor
chr13	110826062		COL4A1	intronic	HIVID	Zhao et al.2016	27703150	Tumor
chr13	38061154		CSNK1A1L(dist=381353),LINC00547(dist=47923)	intergenic	HIVID	Zhao et al.2016	27703150	Tumor
chr4	1709025		SLBP	intronic	HIVID	Zhao et al.2016	27703150	Tumor
chr4	1708953		SLBP	intronic	HIVID	Zhao et al.2016	27703150	Tumor
chr10	44745233		LINC00841(dist=279878),LOC100130539(dist=42965)	intergenic	HIVID	Zhao et al.2016	27703150	Tumor
chr16	26980135		HS3ST4(dist=831126),C16orf82(dist=98084)	intergenic	HIVID	Zhao et al.2016	27703150	Tumor
chr16	26980081		HS3ST4(dist=831072),C16orf82(dist=98138)	intergenic	HIVID	Zhao et al.2016	27703150	Tumor
chr10	42384763		NONE(dist=NONE),LOC441666(dist=442551)	intergenic	HIVID	Zhao et al.2016	27703150	Tumor
chr10	42597212		NONE(dist=NONE),LOC441666(dist=230102)	intergenic	HIVID	Zhao et al.2016	27703150	Tumor
chr12	76408131		KRR1(dist=502713),PHLDA1(dist=11096)	intergenic	HIVID	Zhao et al.2016	27703150	Tumor
chr6	72553397		LINC00472(dist=422949),RIMS1(dist=43009)	intergenic	HIVID	Zhao et al.2016	27703150	Tumor
chr19	39291141		LGALS7B(dist=8747),LGALS4(dist=1170)	intergenic	HIVID	Zhao et al.2016	27703150	Tumor
chr5	14151475		TRIO	intronic	HIVID	Zhao et al.2016	27703150	Tumor
chr2	147220397		TEX41(dist=1386106),PABPC1P2(dist=124228)	intergenic	HIVID	Zhao et al.2016	27703150	Tumor
chr5	1295125		TERT	UTR5	HIVID	Zhao et al.2016	27703150	Tumor
chr5	1295052		TERT	exonic	HIVID	Zhao et al.2016	27703150	Tumor
chr5	1295081		TERT	exonic	HIVID	Zhao et al.2016	27703150	Tumor
chr21	10114446		TEKT4P2(dist=145853),TPTE(dist=792297)	intergenic	HIVID	Zhao et al.2016	27703150	Tumor
chr1	249240454		PGBD2(dist=27109),NONE(dist=NONE)	intergenic	HIVID	Zhao et al.2016	27703150	Tumor
chr1	249240443		PGBD2(dist=27098),NONE(dist=NONE)	intergenic	HIVID	Zhao et al.2016	27703150	Tumor
chr1	249240417		PGBD2(dist=27072),NONE(dist=NONE)	intergenic	HIVID	Zhao et al.2016	27703150	Tumor
chr1	249240347		PGBD2(dist=27002),NONE(dist=NONE)	intergenic	HIVID	Zhao et al.2016	27703150	Tumor
chr1	249240304		PGBD2(dist=26959),NONE(dist=NONE)	intergenic	HIVID	Zhao et al.2016	27703150	Tumor
chr21	34144166		PAXBP1	UTR5	HIVID	Zhao et al.2016	27703150	Tumor
chr3	162872896		OTOL1(dist=1651166),LINC01192(dist=22135)	intergenic	HIVID	Zhao et al.2016	27703150	Tumor
chr18	10349		NONE(dist=NONE),ROCK1P1(dist=98716)	intergenic	HIVID	Zhao et al.2016	27703150	Tumor
chr1	142569458		NONE(dist=NONE),ANKRD20A12P(dist=127963)	intergenic	HIVID	Zhao et al.2016	27703150	Tumor
chr17	19227984		EPN2	intronic	HIVID	Zhao et al.2016	27703150	Tumor
chr17	19239352		EPN2	UTR3	HIVID	Zhao et al.2016	27703150	Tumor
chr9	130620101		ENG;AK1	promoter;downstream	HIVID	Zhao et al.2016	27703150	Tumor
chr4	191043993		DUX4(dist=30551),NONE(dist=NONE)	intergenic	HIVID	Zhao et al.2016	27703150	Tumor
chr4	191043987		DUX4(dist=30545),NONE(dist=NONE)	intergenic	HIVID	Zhao et al.2016	27703150	Tumor
chr4	191043943		DUX4(dist=30501),NONE(dist=NONE)	intergenic	HIVID	Zhao et al.2016	27703150	Tumor
chr2	114360504		DDX11L2	ncRNA_intronic	HIVID	Zhao et al.2016	27703150	Tumor
chr1	10189		DDX11L1;MIR6859-1,MIR6859-2,WASH7P	promoter;downstream	HIVID	Zhao et al.2016	27703150	Tumor
chr1	10270		DDX11L1;MIR6859-1,MIR6859-2,WASH7P	promoter;downstream	HIVID	Zhao et al.2016	27703150	Tumor
chr1	143517108		ANKRD20A12P(dist=803503),MIR6077-1(dist=155813)	intergenic	HIVID	Zhao et al.2016	27703150	Tumor
chr1	142940533		ANKRD20A12P(dist=226928),MIR6077-1(dist=732388)	intergenic	HIVID	Zhao et al.2016	27703150	Tumor
chr5	1295303		TERT	promoter	HIVID	Zhao et al.2016	27703150	Tumor
chr5	1295240		TERT	promoter	HIVID	Zhao et al.2016	27703150	Tumor
chr15	92436735		SLCO3A1	intronic	HIVID	Zhao et al.2016	27703150	Tumor
chr12	66451463		MIR6074(dist=33957),LLPH(dist=65386)	intergenic	HIVID	Zhao et al.2016	27703150	Tumor
chr12	66451377		MIR6074(dist=33871),LLPH(dist=65472)	intergenic	HIVID	Zhao et al.2016	27703150	Tumor
chr18	7547714		LRRC30(dist=315672),PTPRM(dist=19600)	intergenic	HIVID	Zhao et al.2016	27703150	Tumor

Chromosome	Integration site in host genome	Integration site in virus genome	Gene (distance, bp)	Regions	Methods	Author	PMID	Sample
chr18	7541423		LRRC30(dist=309381),PTPRM(dist=25891)	intergenic	HIVID	Zhao et al.2016	27703150	Tumor
chr5	38086390		GDNF-AS1(dist=210490),EGFLAM(dist=172121)	intergenic	HIVID	Zhao et al.2016	27703150	Tumor
chr5	38085097		GDNF-AS1(dist=209197),EGFLAM(dist=173414)	intergenic	HIVID	Zhao et al.2016	27703150	Tumor
chr7	65833021		TPST1(dist=7583),LINC00174(dist=8010)	intergenic	HIVID	Zhao et al.2016	27703150	Tumor
chr7	65832814		TPST1(dist=7376),LINC00174(dist=8217)	intergenic	HIVID	Zhao et al.2016	27703150	Tumor
chr11	48894752		OR4A47(dist=383478),TRIM49B(dist=158400)	intergenic	HIVID	Zhao et al.2016	27703150	Tumor
chr11	48835193		OR4A47(dist=323919),TRIM49B(dist=217959)	intergenic	HIVID	Zhao et al.2016	27703150	Tumor
chr16	46388502		NONE(dist=NONE),ANKRD26P1(dist=114747)	intergenic	HIVID	Zhao et al.2016	27703150	Tumor
chr16	46400147		NONE(dist=NONE),ANKRD26P1(dist=103102)	intergenic	HIVID	Zhao et al.2016	27703150	Tumor
chr8	80379398		IL7(dist=661640),STMN2(dist=143651)	intergenic	HIVID	Zhao et al.2016	27703150	Tumor
chr12	324159		SLC6A12;SLC6A13	promoter;downstream	HIVID	Zhao et al.2016	27703150	Tumor
chr20	43941977		RBPJL	intronic	HIVID	Zhao et al.2016	27703150	Tumor
chr12	18731610		PIK3C2G	intronic	HIVID	Zhao et al.2016	27703150	Tumor
chr18	78016285		PARD6G(dist=10888),NONE(dist=NONE)	intergenic	HIVID	Zhao et al.2016	27703150	Tumor
chr18	10224		NONE(dist=NONE),ROCK1P1(dist=98841)	intergenic	HIVID	Zhao et al.2016	27703150	Tumor
chr5	10408		NONE(dist=NONE),PLEKHG4B(dist=129965)	intergenic	HIVID	Zhao et al.2016	27703150	Tumor
chr5	11522		NONE(dist=NONE),PLEKHG4B(dist=128851)	intergenic	HIVID	Zhao et al.2016	27703150	Tumor
chr12	9494918		LOC642846(dist=28234),DDX12P(dist=75369)	intergenic	HIVID	Zhao et al.2016	27703150	Tumor
chr12	95536		LOC100288778(dist=4273),FAM138D(dist=52410)	intergenic	HIVID	Zhao et al.2016	27703150	Tumor
chr17	21398570		KCNJ12(dist=75391),C17orf51(dist=33001)	intergenic	HIVID	Zhao et al.2016	27703150	Tumor
chr12	194327		IQSEC3	intronic	HIVID	Zhao et al.2016	27703150	Tumor
chr8	59119742		FAM110B(dist=57465),LOC101929528(dist=48588)	intergenic	HIVID	Zhao et al.2016	27703150	Tumor
chr12	15947636		EPS8	promoter	HIVID	Zhao et al.2016	27703150	Tumor
chr10	135524749		DUX4L7(dist=26291),NONE(dist=NONE)	intergenic	HIVID	Zhao et al.2016	27703150	Tumor
chr10	135524713		DUX4L7(dist=26255),NONE(dist=NONE)	intergenic	HIVID	Zhao et al.2016	27703150	Tumor
chr10	135524695		DUX4L7(dist=26237),NONE(dist=NONE)	intergenic	HIVID	Zhao et al.2016	27703150	Tumor
chr4	191043678		DUX4(dist=30236),NONE(dist=NONE)	intergenic	HIVID	Zhao et al.2016	27703150	Tumor
chr2	114361003		DDX11L2	ncRNA_intronic	HIVID	Zhao et al.2016	27703150	Tumor
chr1	10172		DDX11L1;MIR6859-1,MIR6859-2,WASH7P	promoter;downstream	HIVID	Zhao et al.2016	27703150	Tumor
chr12	7528374		CD163L1	exonic	HIVID	Zhao et al.2016	27703150	Tumor
chr12	7509443		CD163L1	intronic	HIVID	Zhao et al.2016	27703150	Tumor
chr12	7510647		CD163L1	intronic	HIVID	Zhao et al.2016	27703150	Tumor
chr7	48071495		C7orf57,SUN3	promoter	HIVID	Zhao et al.2016	27703150	Tumor
chr5	1297187		TERT	promoter	HIVID	Zhao et al.2016	27703150	Tumor
chr5	1297210		TERT	promoter	HIVID	Zhao et al.2016	27703150	Tumor
chr6	102342900		GRIK2	intronic	HIVID	Zhao et al.2016	27703150	Tumor
chr1	161735926		ATF6;DUSP12	promoter;downstream	HIVID	Zhao et al.2016	27703150	Tumor
chr2	216285386		FN1	intronic	HIVID	Zhao et al.2016	27703150	Tumor
chr20	52804485		CYP24A1(dist=13969),PFDN4(dist=20017)	intergenic	HIVID	Zhao et al.2016	27703150	Tumor
chr8	40539544		ZMAT4	intronic	HIVID	Zhao et al.2016	27703150	Tumor
chr8	40539453		ZMAT4	intronic	HIVID	Zhao et al.2016	27703150	Tumor
chr3	14478032		SLC6A6	intronic	HIVID	Zhao et al.2016	27703150	Tumor
chr1	188414290		PLA2G4A(dist=1456177),BRINP3(dist=1652507)	intergenic	HIVID	Zhao et al.2016	27703150	Tumor
chr5	44265844		NNT(dist=560176),FGF10(dist=39253)	intergenic	HIVID	Zhao et al.2016	27703150	Tumor
chr5	44265795		NNT(dist=560127),FGF10(dist=39302)	intergenic	HIVID	Zhao et al.2016	27703150	Tumor
chr1	5734169		MIR4417(dist=109966),MIR4689(dist=188563)	intergenic	HIVID	Zhao et al.2016	27703150	Tumor
chr20	16062493		MACROD2(dist=28652),KIF16B(dist=190256)	intergenic	HIVID	Zhao et al.2016	27703150	Tumor
chr5	84647013		EDIL3(dist=966328),NBPF22P(dist=931249)	intergenic	HIVID	Zhao et al.2016	27703150	Tumor
chr9	126434748		DENND1A	intronic	HIVID	Zhao et al.2016	27703150	Tumor
chr9	126434546		DENND1A	intronic	HIVID	Zhao et al.2016	27703150	Tumor
chr4	49155397		CWH43(dist=91302),NONE(dist=NONE)	intergenic	HIVID	Zhao et al.2016	27703150	Tumor
chr4	49659692		CWH43(dist=595597),NONE(dist=NONE)	intergenic	HIVID	Zhao et al.2016	27703150	Tumor
chr4	49105983		CWH43(dist=41888),NONE(dist=NONE)	intergenic	HIVID	Zhao et al.2016	27703150	Tumor
chr18	587739		CLUL1;CETN1	promoter;downstream	HIVID	Zhao et al.2016	27703150	Tumor
chr18	587775		CLUL1;CETN1	promoter;downstream	HIVID	Zhao et al.2016	27703150	Tumor
chr12	105031125		CHST11	intronic	HIVID	Zhao et al.2016	27703150	Tumor
chr8	41453075		AGPAT6	intronic	HIVID	Zhao et al.2016	27703150	Tumor
chr1	179897460		TOR1AIP1(dist=8248),CEP350(dist=26448)	intergenic	HIVID	Zhao et al.2016	27703150	Tumor
chr2	193462183		TMEFF2(dist=402539),PCGEM1(dist=152388)	intergenic	HIVID	Zhao et al.2016	27703150	Tumor
chr3	141875594		TFDP2;GK5	promoter;downstream	HIVID	Zhao et al.2016	27703150	Tumor
chr3	141875625		TFDP2;GK5	promoter;downstream	HIVID	Zhao et al.2016	27703150	Tumor
chr5	35795350		SPEF2	intronic	HIVID	Zhao et al.2016	27703150	Tumor
chr4	144434173		SMARCA5;SMARCA5-AS1	promoter;downstream	HIVID	Zhao et al.2016	27703150	Tumor
chr4	144434143		SMARCA5;SMARCA5-AS1	promoter;downstream	HIVID	Zhao et al.2016	27703150	Tumor
chr3	80918696		ROBO1(dist=1101637),GBE1(dist=620154)	intergenic	HIVID	Zhao et al.2016	27703150	Tumor
chr9	137091971		RNU6ATAC(dist=62285),RXRA(dist=126345)	intergenic	HIVID	Zhao et al.2016	27703150	Tumor
chr16	33516656		RNU6-76P	ncRNA_intronic	HIVID	Zhao et al.2016	27703150	Tumor
chr20	41780644		PTPRT	intronic	HIVID	Zhao et al.2016	27703150	Tumor
chr9	66950292		PTGER4P2-CDK2AP2P2(dist=447262),RP11-381O7.3(dist=6708[illegible]	intergenic	HIVID	Zhao et al.2016	27703150	Tumor
chr11	125616670		PATE1	intronic	HIVID	Zhao et al.2016	27703150	Tumor
chr2	206113924		PARD3B	intronic	HIVID	Zhao et al.2016	27703150	Tumor
chr13	66039880		OR7E156P(dist=1723179),PCDH9(dist=837086)	intergenic	HIVID	Zhao et al.2016	27703150	Tumor
chr11	51578816		OR4C46(dist=62605),NONE(dist=NONE)	intergenic	HIVID	Zhao et al.2016	27703150	Tumor
chr11	51570164		OR4C46(dist=53953),NONE(dist=NONE)	intergenic	HIVID	Zhao et al.2016	27703150	Tumor
chr7	9232525		NXPH1(dist=439932),PER4(dist=441375)	intergenic	HIVID	Zhao et al.2016	27703150	Tumor
chr7	9232494		NXPH1(dist=439901),PER4(dist=441406)	intergenic	HIVID	Zhao et al.2016	27703150	Tumor
chr7	61908681		NONE(dist=NONE),ZNF733P(dist=842989)	intergenic	HIVID	Zhao et al.2016	27703150	Tumor
chr21	9548352		NONE(dist=NONE),MIR3648(dist=277480)	intergenic	HIVID	Zhao et al.2016	27703150	Tumor
chr10	42727782		NONE(dist=NONE),LOC441666(dist=99532)	intergenic	HIVID	Zhao et al.2016	27703150	Tumor
chr15	20345798		NONE(dist=NONE),CHEK2P2(dist=142199)	intergenic	HIVID	Zhao et al.2016	27703150	Tumor
chr18	28180547		MIR302F(dist=301621),DSC3(dist=389505)	intergenic	HIVID	Zhao et al.2016	27703150	Tumor
chr4	64426114		LPHN3(dist=1487946),TECRL(dist=718063)	intergenic	HIVID	Zhao et al.2016	27703150	Tumor
chr15	21352414		LINC01193(dist=153781),LOC646214(dist=580100)	intergenic	HIVID	Zhao et al.2016	27703150	Tumor
chr9	102868129		INVS	intronic	HIVID	Zhao et al.2016	27703150	Tumor
chr7	54293525		HPVC1(dist=23411),VSTM2A(dist=316494)	intergenic	HIVID	Zhao et al.2016	27703150	Tumor
chr16	9915769		GRIN2A	intronic	HIVID	Zhao et al.2016	27703150	Tumor
chr9	70348315		FOXD4L5(dist=169500),FOXD4L2(dist=78308)	intergenic	HIVID	Zhao et al.2016	27703150	Tumor
chr9	42798308		FOXD4L2(dist=77966),RP11-381O7.3(dist=46062)	intergenic	HIVID	Zhao et al.2016	27703150	Tumor
chr1	118227618		FAM46C(dist=56607),GDAP2(dist=178489)	intergenic	HIVID	Zhao et al.2016	27703150	Tumor
chr2	180823763		CWC22	exonic	HIVID	Zhao et al.2016	27703150	Tumor
chr15	20511246		CHEK2P2(dist=14435),HERC2P3(dist=102404)	intergenic	HIVID	Zhao et al.2016	27703150	Tumor
chr4	77275415		CCDC158	intronic	HIVID	Zhao et al.2016	27703150	Tumor
chr4	77275382		CCDC158	intronic	HIVID	Zhao et al.2016	27703150	Tumor
chr6	93884171		CASC6(dist=1484025),EPHA7(dist=65569)	intergenic	HIVID	Zhao et al.2016	27703150	Tumor
chr22	22644609		BMS1P20	promoter	HIVID	Zhao et al.2016	27703150	Tumor
chr1	94283668		BCAR3	intronic	HIVID	Zhao et al.2016	27703150	Tumor
chr1	235702518		B3GALNT2(dist=34737),GNG4(dist=8467)	intergenic	HIVID	Zhao et al.2016	27703150	Tumor
chr9	67348126		AQP7P1(dist=58634),FAM27E3(dist=436818)	intergenic	HIVID	Zhao et al.2016	27703150	Tumor
chr5	390263		AHRR	intronic	HIVID	Zhao et al.2016	27703150	Tumor
chr3	183733261		ABCC5	intronic	HIVID	Zhao et al.2016	27703150	Tumor
chr8	60263549		TOX(dist=231782),CA8(dist=837874)	intergenic	HIVID	Zhao et al.2016	27703150	Tumor
chr8	62666702		MIR4470(dist=39284),NKAIN3(dist=494799)	intergenic	HIVID	Zhao et al.2016	27703150	Tumor
chr8	62666624		MIR4470(dist=39206),NKAIN3(dist=494877)	intergenic	HIVID	Zhao et al.2016	27703150	Tumor
chr5	177140361		LOC202181(dist=41083),FAM153A(dist=10004)	intergenic	HIVID	Zhao et al.2016	27703150	Tumor
chr5	175551834		LOC100507387	ncRNA_intronic	HIVID	Zhao et al.2016	27703150	Tumor
chr5	4202759		IRX1(dist=601242),LOC101929153(dist=570835)	intergenic	HIVID	Zhao et al.2016	27703150	Tumor
chr11	1945407		TNNT3	intronic	HIVID	Zhao et al.2016	27703150	Tumor
chr13	45966900		TPT1-AS1(dist=1282),SLC25A30(dist=554)	intergenic	HIVID	Zhao et al.2016	27703150	Tumor
chr18	11167287		PIEZO2(dist=18526),SLC35G4(dist=442270)	intergenic	HIVID	Zhao et al.2016	27703150	Tumor
chr18	78016289		PARD6G(dist=10892),NONE(dist=NONE)	intergenic	HIVID	Zhao et al.2016	27703150	Tumor

Chromosome	Integration site in host genome	Integration site in virus genome	Gene (distance, bp)	Regions	Methods	Author	PMID	Sample
chr18	78016280		PARD6G(dist=10883),NONE(dist=NONE)	intergenic	HIVID	Zhao et al.2016	27703150	Tumor
chr18	78016183		PARD6G(dist=10786),NONE(dist=NONE)	intergenic	HIVID	Zhao et al.2016	27703150	Tumor
chr13	35743594		NBEA	intronic	HIVID	Zhao et al.2016	27703150	Tumor
chr2	216265740		FN1	intronic	HIVID	Zhao et al.2016	27703150	Tumor
chr3	75706163		FLJ20518,FRG2C	promoter	HIVID	Zhao et al.2016	27703150	Tumor
chr20	62350035		ZGPAT	intronic	HIVID	Zhao et al.2016	27703150	Tumor
chr9	98829754		RP11-569G13.2	ncRNA_intronic	HIVID	Zhao et al.2016	27703150	Tumor
chr19	34002349		PEPD	intronic	HIVID	Zhao et al.2016	27703150	Tumor
chr12	66451371		MIR6074(dist=33865),LLPH(dist=65478)	intergenic	HIVID	Zhao et al.2016	27703150	Tumor
chr11	100735607		ARHGAP42	intronic	HIVID	Zhao et al.2016	27703150	Tumor
chr19	36213957		KMT2B	exonic	HIVID	Zhao et al.2016	27703150	Tumor
chr13	91553096		LINC00410	ncRNA_intronic	HIVID	Zhao et al.2016	27703150	Tumor
chr13	91551940		LINC00410	ncRNA_intronic	HIVID	Zhao et al.2016	27703150	Tumor
chr19	36212840		KMT2B	intronic	HIVID	Zhao et al.2016	27703150	Tumor
chr8	70094306		RP11-600K15.1(dist=77881),RP11-744J10.3(dist=242800)	intergenic	HIVID	Zhao et al.2016	27703150	Tumor
chr8	70094262		RP11-600K15.1(dist=77837),RP11-744J10.3(dist=242844)	intergenic	HIVID	Zhao et al.2016	27703150	Tumor
chr8	126370531		NSMCE2	intronic	HIVID	Zhao et al.2016	27703150	Tumor
chr19	36212907		KMT2B	intronic	HIVID	Zhao et al.2016	27703150	Tumor
chr19	36212875		KMT2B	intronic	HIVID	Zhao et al.2016	27703150	Tumor
chr19	36212829		KMT2B	intronic	HIVID	Zhao et al.2016	27703150	Tumor
chr19	36212773		KMT2B	intronic	HIVID	Zhao et al.2016	27703150	Tumor
chr8	137040286		KHDRBS3(dist=380438),NONE(dist=NONE)	intergenic	HIVID	Zhao et al.2016	27703150	Tumor
chr8	137040274		KHDRBS3(dist=380426),NONE(dist=NONE)	intergenic	HIVID	Zhao et al.2016	27703150	Tumor
chr2	47912638		KCNK12(dist=115168),MSH6(dist=97583)	intergenic	HIVID	Zhao et al.2016	27703150	Tumor
chr2	47912582		KCNK12(dist=115112),MSH6(dist=97639)	intergenic	HIVID	Zhao et al.2016	27703150	Tumor
chr8	131364465		ASAP1	intronic	HIVID	Zhao et al.2016	27703150	Tumor
chr8	131363656		ASAP1	intronic	HIVID	Zhao et al.2016	27703150	Tumor
chr4	148696118		ARHGAP10	intronic	HIVID	Zhao et al.2016	27703150	Tumor
chr4	148696075		ARHGAP10	intronic	HIVID	Zhao et al.2016	27703150	Tumor
chr6	26753448		ZNF322(dist=93468),GUSBP2(dist=85818)	intergenic	HIVID	Zhao et al.2016	27703150	Tumor
chr7	148932156		ZNF212;ZNF282	promoter;downstream	HIVID	Zhao et al.2016	27703150	Tumor
chr7	63963778		YWHAEP1(dist=67883),ZNF680(dist=16477)	intergenic	HIVID	Zhao et al.2016	27703150	Tumor
chr7	63963744		YWHAEP1(dist=67849),ZNF680(dist=16511)	intergenic	HIVID	Zhao et al.2016	27703150	Tumor
chr1	151157117		VPS72	intronic	HIVID	Zhao et al.2016	27703150	Tumor
chr6	42245720		TRERF1	intronic	HIVID	Zhao et al.2016	27703150	Tumor
chr5	1295173		TERT	promoter	HIVID	Zhao et al.2016	27703150	Tumor
chr5	1295252		TERT	promoter	HIVID	Zhao et al.2016	27703150	Tumor
chr5	1295263		TERT	promoter	HIVID	Zhao et al.2016	27703150	Tumor
chr5	1295225		TERT	promoter	HIVID	Zhao et al.2016	27703150	Tumor
chr5	1295215		TERT	promoter	HIVID	Zhao et al.2016	27703150	Tumor
chr5	70250736		SMN2(dist=1894),NAIP(dist=13574)	intergenic	HIVID	Zhao et al.2016	27703150	Tumor
chr5	70250685		SMN2(dist=1843),NAIP(dist=13625)	intergenic	HIVID	Zhao et al.2016	27703150	Tumor
chr7	107410689		SLC26A3	intronic	HIVID	Zhao et al.2016	27703150	Tumor
chr14	70273565		SLC10A1	promoter	HIVID	Zhao et al.2016	27703150	Tumor
chr7	155964078		SHH(dist=359111),AC073133.1(dist=266405)	intergenic	HIVID	Zhao et al.2016	27703150	Tumor
chr7	155964031		SHH(dist=359064),AC073133.1(dist=266452)	intergenic	HIVID	Zhao et al.2016	27703150	Tumor
chr4	184564392		RWDD4	intronic	HIVID	Zhao et al.2016	27703150	Tumor
chr12	120990838		RNF10	intronic	HIVID	Zhao et al.2016	27703150	Tumor
chr12	120990887		RNF10	intronic	HIVID	Zhao et al.2016	27703150	Tumor
chr9	80994271		PSAT1(dist=49262),RP11-165H23.1(dist=756067)	intergenic	HIVID	Zhao et al.2016	27703150	Tumor
chr9	80994214		PSAT1(dist=49205),RP11-165H23.1(dist=756124)	intergenic	HIVID	Zhao et al.2016	27703150	Tumor
chr19	54409358		PRKCG	intronic	HIVID	Zhao et al.2016	27703150	Tumor
chr8	43828805		POTEA(dist=610477),NONE(dist=NONE)	intergenic	HIVID	Zhao et al.2016	27703150	Tumor
chr16	47539144		PHKB	intronic	HIVID	Zhao et al.2016	27703150	Tumor
chr6	126903128		NONE(dist=NONE),RSPO3(dist=536920)	intergenic	HIVID	Zhao et al.2016	27703150	Tumor
chr8	46839145		NONE(dist=NONE),LINC00293(dist=913363)	intergenic	HIVID	Zhao et al.2016	27703150	Tumor
chr8	46848325		NONE(dist=NONE),LINC00293(dist=904183)	intergenic	HIVID	Zhao et al.2016	27703150	Tumor
chr8	46850194		NONE(dist=NONE),LINC00293(dist=902314)	intergenic	HIVID	Zhao et al.2016	27703150	Tumor
chr8	46855800		NONE(dist=NONE),LINC00293(dist=896708)	intergenic	HIVID	Zhao et al.2016	27703150	Tumor
chr8	46855961		NONE(dist=NONE),LINC00293(dist=896547)	intergenic	HIVID	Zhao et al.2016	27703150	Tumor
chr8	97262923		MTERFD1	intronic	HIVID	Zhao et al.2016	27703150	Tumor
chr13	56275119		MIR5007(dist=526436),PRR20A(dist=1439933)	intergenic	HIVID	Zhao et al.2016	27703150	Tumor
chr13	92778170		GPC5	intronic	HIVID	Zhao et al.2016	27703150	Tumor
chr16	81119828		GCSH	intronic	HIVID	Zhao et al.2016	27703150	Tumor
chr16	81119775		GCSH	intronic	HIVID	Zhao et al.2016	27703150	Tumor
chr17	41060663		G6PC	intronic	HIVID	Zhao et al.2016	27703150	Tumor
chr17	41060630		G6PC	intronic	HIVID	Zhao et al.2016	27703150	Tumor
chr17	41060722		G6PC	intronic	HIVID	Zhao et al.2016	27703150	Tumor
chr17	41060736		G6PC	intronic	HIVID	Zhao et al.2016	27703150	Tumor
chr17	41060833		G6PC	intronic	HIVID	Zhao et al.2016	27703150	Tumor
chr17	642121		FAM57A	intronic	HIVID	Zhao et al.2016	27703150	Tumor
chr6	133594075		EYA4	intronic	HIVID	Zhao et al.2016	27703150	Tumor
chr16	70407018		DDX19A	UTR3	HIVID	Zhao et al.2016	27703150	Tumor
chr19	41690887		CYP2S1	promoter	HIVID	Zhao et al.2016	27703150	Tumor
chr4	156935133		CTSO(dist=60085),PDGFC(dist=747630)	intergenic	HIVID	Zhao et al.2016	27703150	Tumor
chr1	25123651		CLIC4	intronic	HIVID	Zhao et al.2016	27703150	Tumor
chr5	66601080		CD180(dist=108463),LOC102467655(dist=884624)	intergenic	HIVID	Zhao et al.2016	27703150	Tumor
chr5	66601033		CD180(dist=108416),LOC102467655(dist=884671)	intergenic	HIVID	Zhao et al.2016	27703150	Tumor
chr19	30302051		CCNE1	promoter	HIVID	Zhao et al.2016	27703150	Tumor
chr19	30302082		CCNE1	promoter	HIVID	Zhao et al.2016	27703150	Tumor
chr19	30302107		CCNE1	promoter	HIVID	Zhao et al.2016	27703150	Tumor
chr7	3171991		CARD11(dist=88412),SDK1(dist=169089)	intergenic	HIVID	Zhao et al.2016	27703150	Tumor
chr7	3171938		CARD11(dist=88359),SDK1(dist=169142)	intergenic	HIVID	Zhao et al.2016	27703150	Tumor
chr5	126583179		C5orf63(dist=173995),MEGF10(dist=43277)	intergenic	HIVID	Zhao et al.2016	27703150	Tumor
chr9	136947876		BRD3(dist=14735),WDR5(dist=53334)	intergenic	HIVID	Zhao et al.2016	27703150	Tumor
chr21	30838393		BACH1-IT2(dist=92299),GRIK1(dist=70861)	intergenic	HIVID	Zhao et al.2016	27703150	Tumor
chr19	36066753		ATP4A(dist=12193),HAUS5(dist=36893)	intergenic	HIVID	Zhao et al.2016	27703150	Tumor
chr19	36066691		ATP4A(dist=12131),HAUS5(dist=36955)	intergenic	HIVID	Zhao et al.2016	27703150	Tumor
chr2	133039151		ANKRD30BL(dist=23609),GPR39(dist=134996)	intergenic	HIVID	Zhao et al.2016	27703150	Tumor
chr15	64776254		TRIP4(dist=28752),ZNF609(dist=15365)	intergenic	HIVID	Zhao et al.2016	27703150	Tumor
chr15	64776218		TRIP4(dist=28716),ZNF609(dist=15401)	intergenic	HIVID	Zhao et al.2016	27703150	Tumor
chr17	10858419		PIRT(dist=117001),SHISA6(dist=286321)	intergenic	HIVID	Zhao et al.2016	27703150	Tumor
chr1	17220362		MIR3675(dist=34846),CROCC(dist=28083)	intergenic	HIVID	Zhao et al.2016	27703150	Tumor
chr17	49309560		MBTD1	intronic	HIVID	Zhao et al.2016	27703150	Tumor

Chromosome	Integration site in host genome	Integration site in virus genome	Gene (distance, bp)	Regions	Methods	Author	PMID	Sample
chr17	49309557		MBTD1	intronic	HIVID	Zhao et al.2016	27703150	Tumor
chr10	8229152		GATA3(dist=111988),LINC00708(dist=72143)	intergenic	HIVID	Zhao et al.2016	27703150	Tumor
chr10	8229105		GATA3(dist=111941),LINC00708(dist=72190)	intergenic	HIVID	Zhao et al.2016	27703150	Tumor
chr1	17064879		FAM231A	promoter	HIVID	Zhao et al.2016	27703150	Tumor
chr1	16995576		FAM231A	promoter	HIVID	Zhao et al.2016	27703150	Tumor
chr1	16842912		CROCCP3(dist=23716),MIR3675(dist=32497)	intergenic	HIVID	Zhao et al.2016	27703150	Tumor
chr1	185054298		RNF2	intronic	HIVID	Zhao et al.2016	27703150	Tumor
chr5	105076925		RAB9BP1(dist=641126),LOC102467213(dist=1073973)	intergenic	HIVID	Zhao et al.2016	27703150	Tumor
chr13	67346498		PCDH9	intronic	HIVID	Zhao et al.2016	27703150	Tumor
chr13	108753611		FAM155A(dist=234151),LIG4(dist=106181)	intergenic	HIVID	Zhao et al.2016	27703150	Tumor
chr18	30534718		CCDC178	intronic	HIVID	Zhao et al.2016	27703150	Tumor
chr18	30529898		CCDC178	intronic	HIVID	Zhao et al.2016	27703150	Tumor
chr8	53032719		ST18	intronic	HIVID	Zhao et al.2016	27703150	Tumor
chr2	110533004		SOWAHC(dist=156440),RGPD6(dist=17331)	intergenic	HIVID	Zhao et al.2016	27703150	Tumor
chr21	48119887		PRMT2(dist=34851),NONE(dist=NONE)	intergenic	HIVID	Zhao et al.2016	27703150	Tumor
chr21	48119817		PRMT2(dist=34781),NONE(dist=NONE)	intergenic	HIVID	Zhao et al.2016	27703150	Tumor
chr4	55361966		PRMT2(dist=34781),NONE(dist=NONE)	intergenic	HIVID	Zhao et al.2016	27703150	Tumor
chr20	189501		DEFB128(dist=19237),DEFB129(dist=18398)	intergenic	HIVID	Zhao et al.2016	27703150	Tumor
chr2	100706259		AFF3	intronic	HIVID	Zhao et al.2016	27703150	Tumor
chr2	100706209		AFF3	intronic	HIVID	Zhao et al.2016	27703150	Tumor
chr2	191869761		STAT1	intronic	HIVID	Zhao et al.2016	27703150	Tumor
chr5	130803730		RAPGEF6	intronic	HIVID	Zhao et al.2016	27703150	Tumor
chr11	69340326		MYEOV(dist=275572),CCND1(dist=115547)	intergenic	HIVID	Zhao et al.2016	27703150	Tumor
chr5	1295450		TERT	promoter	HIVID	Zhao et al.2016	27703150	Tumor
chr5	146745742		STK32A	intronic	HIVID	Zhao et al.2016	27703150	Tumor
chr17	12952545		ELAC2(dist=31164),HS3ST3A1(dist=446461)	intergenic	HIVID	Zhao et al.2016	27703150	Tumor
chr1	223229120		DISP1(dist=49783),TLR5(dist=53628)	intergenic	HIVID	Zhao et al.2016	27703150	Tumor
chr1	223229085		DISP1(dist=49748),TLR5(dist=53663)	intergenic	HIVID	Zhao et al.2016	27703150	Tumor
chr8	6884070		DEFA1,DEFA1B,DEFA3;DEFA11P	promoter;downstream	HIVID	Zhao et al.2016	27703150	Tumor
chr8	50100320		C8orf22(dist=111678),SNTG1(dist=722029)	intergenic	HIVID	Zhao et al.2016	27703150	Tumor
chr13	77014602		C13orf45(dist=556654),KCTD12(dist=439702)	intergenic	HIVID	Zhao et al.2016	27703150	Tumor
chr5	153722087		GALNT10	intronic	HIVID	Zhao et al.2016	27703150	Tumor
chr11	85195150		DLG2	intronic	HIVID	Zhao et al.2016	27703150	Tumor
chr11	85195232		DLG2	intronic	HIVID	Zhao et al.2016	27703150	Tumor
chr19	30314880		CCNE1	UTR3	HIVID	Zhao et al.2016	27703150	Tumor
chr19	30314956		CCNE1	UTR3	HIVID	Zhao et al.2016	27703150	Tumor
chr13	109135126		TNFSF13B(dist=174294),MYO16(dist=113374)	intergenic	HIVID	Zhao et al.2016	27703150	Tumor
chr17	22262758		MTRNR2L1(dist=238767),NONE(dist=NONE)	intergenic	HIVID	Zhao et al.2016	27703150	Tumor
chr17	22260381		MTRNR2L1(dist=236390),NONE(dist=NONE)	intergenic	HIVID	Zhao et al.2016	27703150	Tumor
chr17	22257054		MTRNR2L1(dist=233063),NONE(dist=NONE)	intergenic	HIVID	Zhao et al.2016	27703150	Tumor
chr17	22252296		MTRNR2L1(dist=228305),NONE(dist=NONE)	intergenic	HIVID	Zhao et al.2016	27703150	Tumor
chr17	22250866		MTRNR2L1(dist=226875),NONE(dist=NONE)	intergenic	HIVID	Zhao et al.2016	27703150	Tumor
chr17	22248490		MTRNR2L1(dist=224499),NONE(dist=NONE)	intergenic	HIVID	Zhao et al.2016	27703150	Tumor
chr17	22246111		MTRNR2L1(dist=222120),NONE(dist=NONE)	intergenic	HIVID	Zhao et al.2016	27703150	Tumor
chr5	893284		TRIP13	intronic	HIVID	Zhao et al.2016	27703150	Tumor
chr1	249240217		PGBD2(dist=26872),NONE(dist=NONE)	intergenic	HIVID	Zhao et al.2016	27703150	Tumor
chr1	249239902		PGBD2(dist=26557),NONE(dist=NONE)	intergenic	HIVID	Zhao et al.2016	27703150	Tumor
chr10	85852821		NRG3(dist=1105886),GHITM(dist=46364)	intergenic	HIVID	Zhao et al.2016	27703150	Tumor
chr4	10038		NONE(dist=NONE),ZNF595(dist=43141)	intergenic	HIVID	Zhao et al.2016	27703150	Tumor
chr4	10117		NONE(dist=NONE),ZNF595(dist=43062)	intergenic	HIVID	Zhao et al.2016	27703150	Tumor
chr5	11583		NONE(dist=NONE),PLEKHG4B(dist=128790)	intergenic	HIVID	Zhao et al.2016	27703150	Tumor
chr9	42344829		KGFLP2(dist=325245),ANKRD20A2(dist=23474)	intergenic	HIVID	Zhao et al.2016	27703150	Tumor
chr4	191044164		DUX4(dist=30722),NONE(dist=NONE)	intergenic	HIVID	Zhao et al.2016	27703150	Tumor
chr9	69358446		CBWD5(dist=95816),ANKRD20A4(dist=23535)	intergenic	HIVID	Zhao et al.2016	27703150	Tumor
chr12	127645646		RP11-575F12.1(dist=100704),RP11-955H22.3(dist=163054)	intergenic	HIVID	Zhao et al.2016	27703150	Tumor
chr17	15748456		MEIS3P1(dist=55437),ADORA2B(dist=99775)	intergenic	HIVID	Zhao et al.2016	27703150	Tumor
chr19	36213578		KMT2B	exonic	HIVID	Zhao et al.2016	27703150	Tumor
chr19	36213558		KMT2B	exonic	HIVID	Zhao et al.2016	27703150	Tumor
chr15	83898558		HDGFRP3(dist=21788),BNC1(dist=26097)	intergenic	HIVID	Zhao et al.2016	27703150	Tumor
chr15	83898517		HDGFRP3(dist=21747),BNC1(dist=26138)	intergenic	HIVID	Zhao et al.2016	27703150	Tumor
chr15	83898476		HDGFRP3(dist=21706),BNC1(dist=26179)	intergenic	HIVID	Zhao et al.2016	27703150	Tumor
chr15	83898271		HDGFRP3(dist=21501),BNC1(dist=26384)	intergenic	HIVID	Zhao et al.2016	27703150	Tumor
chr17	13085194		ELAC2(dist=163813),HS3ST3A1(dist=313812)	intergenic	HIVID	Zhao et al.2016	27703150	Tumor
chr17	20566061		CDRT15L2(dist=81837),LOC100287072(dist=49698)	intergenic	HIVID	Zhao et al.2016	27703150	Tumor
chr13	19447162		ANKRD20A9P	promoter	HIVID	Zhao et al.2016	27703150	Tumor
chr21	15344650		ANKRD20A11P	ncRNA_intronic	HIVID	Zhao et al.2016	27703150	Tumor
chr1	70795601		ANKRD13C	intronic	HIVID	Zhao et al.2016	27703150	Tumor
chr1	60212		OR4F5	promoter	HIVID	Zhao et al.2016	27703150	Tumor
chr15	102470655		OR4F4	promoter	HIVID	Zhao et al.2016	27703150	Tumor
chr10	81661529		LOC100288974	promoter	HIVID	Zhao et al.2016	27703150	Tumor
chr6	169135275		SMOC2(dist=66601),THBS2(dist=480600)	intergenic	HIVID	Zhao et al.2016	27703150	Tumor
chr19	54142679		DPRX(dist=2416),MIR512-2(dist=27248)	intergenic	HIVID	Zhao et al.2016	27703150	Tumor
chr2	85337758		KCMF1(dist=51163),TCF7L1(dist=22825)	intergenic	HIVID	Zhao et al.2016	27703150	Tumor
chr17	13694699		HS3ST3A1(dist=189455),CDRT15P1(dist=233116)	intergenic	HIVID	Zhao et al.2016	27703150	Tumor
chr5	1300591		TERT;MIR4457	promoter;downstream	HIVID	Zhao et al.2016	27703150	Tumor
chr11	70791199		SHANK2	intronic	HIVID	Zhao et al.2016	27703150	Tumor
chr11	75287796		SERPINH1(dist=3947),MAP6(dist=10167)	intergenic	HIVID	Zhao et al.2016	27703150	Tumor
chr18	105548		ROCK1P1;MIR8078	promoter;downstream	HIVID	Zhao et al.2016	27703150	Tumor
chr4	97831710		PDHA2(dist=1069085),STPG2-AS1(dist=456367)	intergenic	HIVID	Zhao et al.2016	27703150	Tumor
chr18	78016326		PARD6G(dist=10929),NONE(dist=NONE)	intergenic	HIVID	Zhao et al.2016	27703150	Tumor
chr18	78016268		PARD6G(dist=10871),NONE(dist=NONE)	intergenic	HIVID	Zhao et al.2016	27703150	Tumor
chr10	92044730		LINC00865(dist=444112),HTR7(dist=455846)	intergenic	HIVID	Zhao et al.2016	27703150	Tumor
chr10	92044697		LINC00865(dist=444079),HTR7(dist=455879)	intergenic	HIVID	Zhao et al.2016	27703150	Tumor
chr11	71453369		KRTAP5-11(dist=159448),FAM86C1(dist=45188)	intergenic	HIVID	Zhao et al.2016	27703150	Tumor
chr11	71451860		KRTAP5-11(dist=157939),FAM86C1(dist=46697)	intergenic	HIVID	Zhao et al.2016	27703150	Tumor
chr6	58777501		GUSBP4(dist=489777),NONE(dist=NONE)	intergenic	HIVID	Zhao et al.2016	27703150	Tumor
chr6	58776532		GUSBP4(dist=488808),NONE(dist=NONE)	intergenic	HIVID	Zhao et al.2016	27703150	Tumor
chr3	129866866		FAM86HP(dist=36590),COL6A4P2(dist=64797)	intergenic	HIVID	Zhao et al.2016	27703150	Tumor
chr10	135524741		DUX4L7(dist=26283),NONE(dist=NONE)	intergenic	HIVID	Zhao et al.2016	27703150	Tumor
chr4	49142312		CWH43(dist=78217),NONE(dist=NONE)	intergenic	HIVID	Zhao et al.2016	27703150	Tumor
chr4	49658631		CWH43(dist=594536),NONE(dist=NONE)	intergenic	HIVID	Zhao et al.2016	27703150	Tumor
chr4	49658568		CWH43(dist=594473),NONE(dist=NONE)	intergenic	HIVID	Zhao et al.2016	27703150	Tumor
chr9	35454512		ATP8B5P	ncRNA_intronic	HIVID	Zhao et al.2016	27703150	Tumor
chr10	90700309		ACTA2	intronic	HIVID	Zhao et al.2016	27703150	Tumor

Chromosome	Integration site in host genome	Integration site in virus genome	Gene (distance, bp)	Regions	Methods	Author	PMID	Sample
chr2	65079930		AC007880.1	ncRNA_intronic	HIVID	Zhao et al.2016	27703150	Tumor
chr8	62647845		MIR4470(dist=20427),NKAIN3(dist=513656)	intergenic	HIVID	Zhao et al.2016	27703150	Tumor
chr9	26532582		LOC100506422(dist=414176),CAAP1(dist=308101)	intergenic	HIVID	Zhao et al.2016	27703150	Tumor
chr8	63932594		GGH	intronic	HIVID	Zhao et al.2016	27703150	Tumor
chr8	63932897		GGH	intronic	HIVID	Zhao et al.2016	27703150	Tumor
chr8	63932571		GGH	intronic	HIVID	Zhao et al.2016	27703150	Tumor
chr8	63932870		GGH	intronic	HIVID	Zhao et al.2016	27703150	Tumor
chr8	63932835		GGH	intronic	HIVID	Zhao et al.2016	27703150	Tumor
chr8	63932712		GGH	intronic	HIVID	Zhao et al.2016	27703150	Tumor
chr8	63932991		GGH	intronic	HIVID	Zhao et al.2016	27703150	Tumor
chr8	63932254		GGH	intronic	HIVID	Zhao et al.2016	27703150	Tumor
chr8	63934624		GGH	intronic	HIVID	Zhao et al.2016	27703150	Tumor
chr8	63932637		GGH	intronic	HIVID	Zhao et al.2016	27703150	Tumor
chr8	63932951		GGH	intronic	HIVID	Zhao et al.2016	27703150	Tumor
chr8	94171816		C8orf87	intronic	HIVID	Zhao et al.2016	27703150	Tumor
chr16	59939797		APOOP5(dist=150702),LOC729159(dist=452562)	intergenic	HIVID	Zhao et al.2016	27703150	Tumor
chr5	97332768		LOC102546227(dist=326013),RGMB(dist=772231)	intergenic	HIVID	Zhao et al.2016	27703150	Tumor
chr17	9792032		GLP2R	intronic	HIVID	Zhao et al.2016	27703150	Tumor
chr16	33959484		RNU6-76P(dist=396241),LINC00273(dist=1568)	intergenic	HIVID	Zhao et al.2016	27703150	Tumor
chr17	22252693		MTRNR2L1(dist=228702),NONE(dist=NONE)	intergenic	HIVID	Zhao et al.2016	27703150	Tumor
chr17	22250317		MTRNR2L1(dist=226326),NONE(dist=NONE)	intergenic	HIVID	Zhao et al.2016	27703150	Tumor
chr17	22247938		MTRNR2L1(dist=223947),NONE(dist=NONE)	intergenic	HIVID	Zhao et al.2016	27703150	Tumor
chr5	1312517		MIR4457;CLPTM1L	promoter;downstream	HIVID	Zhao et al.2016	27703150	Tumor
chr22	35501114		ISX(dist=17734),HMGXB4(dist=152331)	intergenic	HIVID	Zhao et al.2016	27703150	Tumor
chr5	11349649		CTNND2	intronic	HIVID	Zhao et al.2016	27703150	Tumor
chr4	122743030		CCNA2	intronic	HIVID	Zhao et al.2016	27703150	Tumor
chr4	122743054		CCNA2	intronic	HIVID	Zhao et al.2016	27703150	Tumor
chr10	132816		TUBB8(dist=37638),ZMYND11(dist=47589)	intergenic	HIVID	Zhao et al.2016	27703150	Tumor
chr18	105578		ROCK1P1;MIR8078	promoter;downstream	HIVID	Zhao et al.2016	27703150	Tumor
chr9	66971304		PTGER4P2-CDK2AP2P2(dist=468274),RP11-381O7.3(dist=4607	intergenic	HIVID	Zhao et al.2016	27703150	Tumor
chr18	18519733		NONE(dist=NONE),ROCK1(dist=9970)	intergenic	HIVID	Zhao et al.2016	27703150	Tumor
chr18	18520325		NONE(dist=NONE),ROCK1(dist=9378)	intergenic	HIVID	Zhao et al.2016	27703150	Tumor
chr2	172911286		METAP1D	intronic	HIVID	Zhao et al.2016	27703150	Tumor
chr5	86107665		LOC100505878(dist=62076),MIR4280(dist=303031)	intergenic	HIVID	Zhao et al.2016	27703150	Tumor
chr9	69711039		LOC100133920(dist=46090),FOXD4L5(dist=464668)	intergenic	HIVID	Zhao et al.2016	27703150	Tumor
chr2	50106616		FSHR(dist=724950),NRXN1(dist=39027)	intergenic	HIVID	Zhao et al.2016	27703150	Tumor
chr19	36204276		ZBTB32	intronic	HIVID	Zhao et al.2016	27703150	Tumor
chr7	129551161		UBE2H	intronic	HIVID	Zhao et al.2016	27703150	Tumor
chr7	129551226		UBE2H	intronic	HIVID	Zhao et al.2016	27703150	Tumor
chr1	1352708		TMEM88B;ANKRD65	promoter;downstream	HIVID	Zhao et al.2016	27703150	Tumor
chr18	9088987		SOGA2(dist=256212),NDUFV2(dist=13641)	intergenic	HIVID	Zhao et al.2016	27703150	Tumor
chr22	31752240		PATZ1	promoter	HIVID	Zhao et al.2016	27703150	Tumor
chr22	31752178		PATZ1	promoter	HIVID	Zhao et al.2016	27703150	Tumor
chr5	11480		NONE(dist=NONE),PLEKHG4B(dist=128893)	intergenic	HIVID	Zhao et al.2016	27703150	Tumor
chr5	11578		NONE(dist=NONE),PLEKHG4B(dist=128795)	intergenic	HIVID	Zhao et al.2016	27703150	Tumor
chr1	1352561		MRPL20,TMEM88B;ANKRD65	promoter;downstream	HIVID	Zhao et al.2016	27703150	Tumor
chr17	21194918		MAP2K3	intronic	HIVID	Zhao et al.2016	27703150	Tumor
chr12	95729		LOC100288778(dist=4466),FAM138D(dist=52217)	intergenic	HIVID	Zhao et al.2016	27703150	Tumor
chr12	95532		LOC100288778(dist=4269),FAM138D(dist=52414)	intergenic	HIVID	Zhao et al.2016	27703150	Tumor
chr2	33091990		LINC00486	ncRNA_intronic	HIVID	Zhao et al.2016	27703150	Tumor
chr2	33091956		LINC00486	ncRNA_intronic	HIVID	Zhao et al.2016	27703150	Tumor
chr19	36212753		KMT2B	intronic	HIVID	Zhao et al.2016	27703150	Tumor
chr2	206936041		INO80D	intronic	HIVID	Zhao et al.2016	27703150	Tumor
chr2	206935979		INO80D	intronic	HIVID	Zhao et al.2016	27703150	Tumor
chr15	100453064		DNM1P46(dist=105932),ADAMTS17(dist=58579)	intergenic	HIVID	Zhao et al.2016	27703150	Tumor
chr15	102521370		DDX11L9;MIR6859-1,MIR6859-2,WASH3P	promoter;downstream	HIVID	Zhao et al.2016	27703150	Tumor
chr7	1164342		C7orf50	intronic	HIVID	Zhao et al.2016	27703150	Tumor
chr7	1082005		C7orf50	intronic	HIVID	Zhao et al.2016	27703150	Tumor
chr20	4211730		ADRA1D	intronic	HIVID	Zhao et al.2016	27703150	Tumor
chr2	163646791		AC007740.1	ncRNA_intronic	HIVID	Zhao et al.2016	27703150	Tumor
chr5	1296347		TERT	promoter	HIVID	Zhao et al.2016	27703150	Tumor
chr5	1296260		TERT	promoter	HIVID	Zhao et al.2016	27703150	Tumor
chr17	77254469		RBFOX3	intronic	HIVID	Zhao et al.2016	27703150	Tumor
chr16	90188099		PRDM7(dist=45761),NONE(dist=NONE)	intergenic	HIVID	Zhao et al.2016	27703150	Tumor
chr1	249240267		PGBD2(dist=26922),NONE(dist=NONE)	intergenic	HIVID	Zhao et al.2016	27703150	Tumor
chr4	10073		NONE(dist=NONE),ZNF595(dist=43106)	intergenic	HIVID	Zhao et al.2016	27703150	Tumor
chr4	10079		NONE(dist=NONE),ZNF595(dist=43100)	intergenic	HIVID	Zhao et al.2016	27703150	Tumor
chr4	10169		NONE(dist=NONE),ZNF595(dist=43010)	intergenic	HIVID	Zhao et al.2016	27703150	Tumor
chr18	10036		NONE(dist=NONE),ROCK1P1(dist=99029)	intergenic	HIVID	Zhao et al.2016	27703150	Tumor
chr18	10054		NONE(dist=NONE),ROCK1P1(dist=99011)	intergenic	HIVID	Zhao et al.2016	27703150	Tumor
chr5	11484		NONE(dist=NONE),PLEKHG4B(dist=128889)	intergenic	HIVID	Zhao et al.2016	27703150	Tumor
chr5	11502		NONE(dist=NONE),PLEKHG4B(dist=128871)	intergenic	HIVID	Zhao et al.2016	27703150	Tumor
chr5	11589		NONE(dist=NONE),PLEKHG4B(dist=128784)	intergenic	HIVID	Zhao et al.2016	27703150	Tumor
chr7	16950		NONE(dist=NONE),LOC100507642(dist=132768)	intergenic	HIVID	Zhao et al.2016	27703150	Tumor
chr10	100116305		LOXL4(dist=88298),PYROXD2(dist=27017)	intergenic	HIVID	Zhao et al.2016	27703150	Tumor
chr12	95624		LOC100288778(dist=4361),FAM138D(dist=52322)	intergenic	HIVID	Zhao et al.2016	27703150	Tumor
chr12	95251		LOC100288778(dist=3988),FAM138D(dist=52695)	intergenic	HIVID	Zhao et al.2016	27703150	Tumor
chr11	176506		LINC01001(dist=44586),SCGB1C1(dist=16574)	intergenic	HIVID	Zhao et al.2016	27703150	Tumor
chr2	239931055		FLJ43879(dist=83090),HDAC4(dist=38809)	intergenic	HIVID	Zhao et al.2016	27703150	Tumor
chr9	141127136		FAM157B	intronic	HIVID	Zhao et al.2016	27703150	Tumor
chr4	191044232		DUX4(dist=30790),NONE(dist=NONE)	intergenic	HIVID	Zhao et al.2016	27703150	Tumor
chr4	191044208		DUX4(dist=30766),NONE(dist=NONE)	intergenic	HIVID	Zhao et al.2016	27703150	Tumor
chr4	191044111		DUX4(dist=30669),NONE(dist=NONE)	intergenic	HIVID	Zhao et al.2016	27703150	Tumor
chr4	191044092		DUX4(dist=30650),NONE(dist=NONE)	intergenic	HIVID	Zhao et al.2016	27703150	Tumor
chr15	102521337		DDX11L9;MIR6859-1,MIR6859-2,WASH3P	promoter;downstream	HIVID	Zhao et al.2016	27703150	Tumor
chr15	102521392		DDX11L9;MIR6859-1,MIR6859-2,WASH3P	promoter;downstream	HIVID	Zhao et al.2016	27703150	Tumor
chr15	102521361		DDX11L9;MIR6859-1,MIR6859-2,WASH3P	promoter;downstream	HIVID	Zhao et al.2016	27703150	Tumor
chr1	10008		DDX11L1;MIR6859-1,MIR6859-2,WASH7P	promoter;downstream	HIVID	Zhao et al.2016	27703150	Tumor
chr1	10181		DDX11L1;MIR6859-1,MIR6859-2,WASH7P	promoter;downstream	HIVID	Zhao et al.2016	27703150	Tumor
chr19	59118972		CENPBD1P1(dist=23210),NONE(dist=NONE)	intergenic	HIVID	Zhao et al.2016	27703150	Tumor
chr1	121484851		EMBP1(dist=171165),NONE(dist=NONE)	intergenic	HIVID	Zhao et al.2016	27703150	Tumor
chr10	135524748		DUX4L7(dist=26290),NONE(dist=NONE)	intergenic	HIVID	Zhao et al.2016	27703150	Tumor
chr10	135524730		DUX4L7(dist=26272),NONE(dist=NONE)	intergenic	HIVID	Zhao et al.2016	27703150	Tumor
chr4	49315972		CWH43(dist=251877),NONE(dist=NONE)	intergenic	HIVID	Zhao et al.2016	27703150	Tumor

Chromosome	Integration site in host genome	Integration site in virus genome	Gene (distance, bp)	Regions	Methods	Author	PMID	Sample
chr2	162136649		AC009299.3(dist=31088),PSMD14(dist=28137)	intergenic	HIVID	Zhao et al.2016	27703150	Tumor
chr1	10222377		UBE4B	intronic	HIVID	Zhao et al.2016	27703150	Tumor
chr15	78292082		TBC1D2B	intronic	HIVID	Zhao et al.2016	27703150	Tumor
chr9	131558455		TBC1D13	intronic	HIVID	Zhao et al.2016	27703150	Tumor
chr9	131558292		TBC1D13	intronic	HIVID	Zhao et al.2016	27703150	Tumor
chr9	131558378		TBC1D13	intronic	HIVID	Zhao et al.2016	27703150	Tumor
chr22	33169264		SYN3	intronic	HIVID	Zhao et al.2016	27703150	Tumor
chr20	62188377		SRMS;C20orf195,HELZ2	promoter;downstream	HIVID	Zhao et al.2016	27703150	Tumor
chr13	37420484		SMAD9	UTR3	HIVID	Zhao et al.2016	27703150	Tumor
chr11	70769951		SHANK2	intronic	HIVID	Zhao et al.2016	27703150	Tumor
chr1	156666776		NES(dist=19587),CRABP2(dist=2624)	intergenic	HIVID	Zhao et al.2016	27703150	Tumor
chr7	73533328		LIMK1	intronic	HIVID	Zhao et al.2016	27703150	Tumor
chr7	91105819		FZD1(dist=207687),MTERF(dist=396202)	intergenic	HIVID	Zhao et al.2016	27703150	Tumor
chr7	91105720		FZD1(dist=207588),MTERF(dist=396301)	intergenic	HIVID	Zhao et al.2016	27703150	Tumor
chr7	91104928		FZD1(dist=206796),MTERF(dist=397093)	intergenic	HIVID	Zhao et al.2016	27703150	Tumor
chr20	61939015		COL20A1	intronic	HIVID	Zhao et al.2016	27703150	Tumor
chr20	59829466		CDH4	intronic	HIVID	Zhao et al.2016	27703150	Tumor
chr20	32100706		CBFA2T2	intronic	HIVID	Zhao et al.2016	27703150	Tumor
chr20	62187633		C20orf195	exonic	HIVID	Zhao et al.2016	27703150	Tumor
chr3	35729869		ARPP21	intronic	HIVID	Zhao et al.2016	27703150	Tumor
chr16	76454968		CNTNAP4	intronic	HIVID	Zhao et al.2016	27703150	Tumor
chr16	76454880		CNTNAP4	intronic	HIVID	Zhao et al.2016	27703150	Tumor
chr3	153276811		C3orf79(dist=56325),ARHGEF26-AS1(dist=465379)	intergenic	HIVID	Zhao et al.2016	27703150	Tumor
chr5	91765851		ARRDC3-AS1(dist=1049319),NR2F1-AS1(dist=979211)	intergenic	HIVID	Zhao et al.2016	27703150	Tumor
chr19	36204675		ZBTB32	intronic	HIVID	Zhao et al.2016	27703150	Tumor
chr17	22253301		MTRNR2L1(dist=229310),NONE(dist=NONE)	intergenic	HIVID	Zhao et al.2016	27703150	Tumor
chr17	22253187		MTRNR2L1(dist=229196),NONE(dist=NONE)	intergenic	HIVID	Zhao et al.2016	27703150	Tumor
chr19	36212596		KMT2B	exonic	HIVID	Zhao et al.2016	27703150	Tumor
chr1	172813994		FASLG(dist=177982),TNFSF18(dist=196366)	intergenic	HIVID	Zhao et al.2016	27703150	Tumor
chr1	172810746		FASLG(dist=174734),TNFSF18(dist=199614)	intergenic	HIVID	Zhao et al.2016	27703150	Tumor
chr1	121354002		EMBP1(dist=40316),NONE(dist=NONE)	intergenic	HIVID	Zhao et al.2016	27703150	Tumor
chr1	121352302		EMBP1(dist=38616),NONE(dist=NONE)	intergenic	HIVID	Zhao et al.2016	27703150	Tumor
chr13	44766491		SMIM2(dist=31098),MIR8079(dist=3774)	intergenic	HIVID	Zhao et al.2016	27703150	Tumor
chr6	148677172		SASH1	intronic	HIVID	Zhao et al.2016	27703150	Tumor
chr6	148677207		SASH1	intronic	HIVID	Zhao et al.2016	27703150	Tumor
chr2	99327807		MGAT4A	intronic	HIVID	Zhao et al.2016	27703150	Tumor
chr3	119561134		GSK3B	intronic	HIVID	Zhao et al.2016	27703150	Tumor
chr15	100453064		DNM1P46(dist=105932),ADAMTS17(dist=58579)	intergenic	HIVID	Zhao et al.2016	27703150	Tumor
chr12	34458348		ALG10(dist=277112),NONE(dist=NONE)	intergenic	HIVID	Zhao et al.2016	27703150	Tumor
chr12	113885194		SDSL(dist=9113),LHX5(dist=15500)	intergenic	HIVID	Zhao et al.2016	27703150	Tumor
chr12	113885168		SDSL(dist=9087),LHX5(dist=15526)	intergenic	HIVID	Zhao et al.2016	27703150	Tumor
chr6	9477778		RP11-314C16.1(dist=692100),TFAP2A(dist=919138)	intergenic	HIVID	Zhao et al.2016	27703150	Tumor
chr2	192355872		MYO1B(dist=65757),NABP1(dist=186926)	intergenic	HIVID	Zhao et al.2016	27703150	Tumor
chr3	197899619		FAM157A	intronic	HIVID	Zhao et al.2016	27703150	Tumor
chr3	197899654		FAM157A	intronic	HIVID	Zhao et al.2016	27703150	Tumor
chr1	81350497		ELTD1(dist=1878002),LPHN2(dist=915585)	intergenic	HIVID	Zhao et al.2016	27703150	Tumor
chr1	81348271		ELTD1(dist=1875776),LPHN2(dist=917811)	intergenic	HIVID	Zhao et al.2016	27703150	Tumor
chr15	102521231		DDX11L9;MIR6859-1,MIR6859-2,WASH3P	promoter;downstream	HIVID	Zhao et al.2016	27703150	Tumor
chr16	89516211		ANKRD11	intronic	HIVID	Zhao et al.2016	27703150	Tumor
chr1	6333841		ACOT7	intronic	HIVID	Zhao et al.2016	27703150	Tumor
chr16	17298801		XYLT1	intronic	HIVID	Zhao et al.2016	27703150	Tumor
chr16	17298824		XYLT1	intronic	HIVID	Zhao et al.2016	27703150	Tumor
chr1	198296563		NEK7(dist=5015),ATP6V1G3(dist=195789)	intergenic	HIVID	Zhao et al.2016	27703150	Tumor
chr17	22261353		MTRNR2L1(dist=237362),NONE(dist=NONE)	intergenic	HIVID	Zhao et al.2016	27703150	Tumor
chr17	22257818		MTRNR2L1(dist=233827),NONE(dist=NONE)	intergenic	HIVID	Zhao et al.2016	27703150	Tumor
chr17	22254217		MTRNR2L1(dist=230226),NONE(dist=NONE)	intergenic	HIVID	Zhao et al.2016	27703150	Tumor
chr17	22254142		MTRNR2L1(dist=230151),NONE(dist=NONE)	intergenic	HIVID	Zhao et al.2016	27703150	Tumor
chr17	22253186		MTRNR2L1(dist=229195),NONE(dist=NONE)	intergenic	HIVID	Zhao et al.2016	27703150	Tumor
chr5	144698142		KCTD16(dist=841198),PRELID2(dist=437765)	intergenic	HIVID	Zhao et al.2016	27703150	Tumor
chr14	67494515		GPHN	intronic	HIVID	Zhao et al.2016	27703150	Tumor
chr1	121357005		EMBP1(dist=43319),NONE(dist=NONE)	intergenic	HIVID	Zhao et al.2016	27703150	Tumor
chr1	121355805		EMBP1(dist=42119),NONE(dist=NONE)	intergenic	HIVID	Zhao et al.2016	27703150	Tumor
chr1	121354003		EMBP1(dist=40317),NONE(dist=NONE)	intergenic	HIVID	Zhao et al.2016	27703150	Tumor
chr5	1295473		TERT	promoter	HIVID	Zhao et al.2016	27703150	Tumor
chr5	1295431		TERT	promoter	HIVID	Zhao et al.2016	27703150	Tumor
chr8	70745506		SLCO5A1	UTR5	HIVID	Zhao et al.2016	27703150	Tumor
chr11	85881770		PICALM(dist=100847),EED(dist=74036)	intergenic	HIVID	Zhao et al.2016	27703150	Tumor
chr11	85881723		PICALM(dist=100800),EED(dist=74083)	intergenic	HIVID	Zhao et al.2016	27703150	Tumor
chr5	11475		NONE(dist=NONE),PLEKHG4B(dist=128898)	intergenic	HIVID	Zhao et al.2016	27703150	Tumor
chr5	11584		NONE(dist=NONE),PLEKHG4B(dist=128789)	intergenic	HIVID	Zhao et al.2016	27703150	Tumor
chr5	11662		NONE(dist=NONE),PLEKHG4B(dist=128711)	intergenic	HIVID	Zhao et al.2016	27703150	Tumor
chr16	46425569		NONE(dist=NONE),ANKRD26P1(dist=77680)	intergenic	HIVID	Zhao et al.2016	27703150	Tumor
chr17	22253179		MTRNR2L1(dist=229188),NONE(dist=NONE)	intergenic	HIVID	Zhao et al.2016	27703150	Tumor
chr11	134946519		LOC283177(dist=570964),NONE(dist=NONE)	intergenic	HIVID	Zhao et al.2016	27703150	Tumor
chr12	95729		LOC100288778(dist=4466),FAM138D(dist=52217)	intergenic	HIVID	Zhao et al.2016	27703150	Tumor
chr12	95543		LOC100288778(dist=4280),FAM138D(dist=52403)	intergenic	HIVID	Zhao et al.2016	27703150	Tumor
chr15	26672067		LINC00929(dist=293883),GABRB3(dist=116627)	intergenic	HIVID	Zhao et al.2016	27703150	Tumor
chr15	26672036		LINC00929(dist=293852),GABRB3(dist=116658)	intergenic	HIVID	Zhao et al.2016	27703150	Tumor
chr13	88913534		LINC00397(dist=450759),LINC00433(dist=279550)	intergenic	HIVID	Zhao et al.2016	27703150	Tumor
chr19	36212932		KMT2B	intronic	HIVID	Zhao et al.2016	27703150	Tumor
chr11	88711682		GRM5	intronic	HIVID	Zhao et al.2016	27703150	Tumor
chr11	88711637		GRM5	intronic	HIVID	Zhao et al.2016	27703150	Tumor
chr19	35630538		FXYD1	splicing	HIVID	Zhao et al.2016	27703150	Tumor
chr16	53794344		FTO	intronic	HIVID	Zhao et al.2016	27703150	Tumor
chr1	10177		DDX11L1;MIR6859-1,MIR6859-2,WASH7P	promoter;downstream	HIVID	Zhao et al.2016	27703150	Tumor
chr4	49125412		CWH43(dist=61317),NONE(dist=NONE)	intergenic	HIVID	Zhao et al.2016	27703150	Tumor
chr10	87121398		CCSER2(dist=843121),GRID1-AS1(dist=216090)	intergenic	HIVID	Zhao et al.2016	27703150	Tumor
chr10	87121350		CCSER2(dist=843073),GRID1-AS1(dist=216138)	intergenic	HIVID	Zhao et al.2016	27703150	Tumor
chr5	1312517		MIR4457;CLPTM1L	promoter;downstream	HIVID	Zhao et al.2016	27703150	Tumor
chr4	122743030		CCNA2	intronic	HIVID	Zhao et al.2016	27703150	Tumor
chr4	122743054		CCNA2	intronic	HIVID	Zhao et al.2016	27703150	Tumor
chr6	118707082		SLC35F1(dist=68243),CEP85L(dist=74853)	intergenic	HIVID	Zhao et al.2016	27703150	Tumor
chr8	69060666		PREX2	intronic	HIVID	Zhao et al.2016	27703150	Tumor
chr19	36212626		KMT2B	exonic	HIVID	Zhao et al.2016	27703150	Tumor

Chromosome	Integration site in host genome	Integration site in virus genome	Gene (distance, bp)	Regions	Methods	Author	PMID	Sample
chr1	217137044		ESRRG	intronic	HIVID	Zhao et al.2016	27703150	Tumor
chr1	217137138		ESRRG	intronic	HIVID	Zhao et al.2016	27703150	Tumor
chr5	1295131		TERT	UTR5	HIVID	Zhao et al.2016	27703150	Tumor
chr3	18167842		LOC339862	intronic	HIVID	Zhao et al.2016	27703150	Tumor
chr3	18168250		LOC339862	intronic	HIVID	Zhao et al.2016	27703150	Tumor
chr13	66853284		NONE(dist=NONE),PCDH9(dist=23682)	intergenic	HIVID	Zhao et al.2016	27703150	Tumor
chr11	21563428		NELL1	intronic	HIVID	Zhao et al.2016	27703150	Tumor
chr2	141741849		LRP1B	intronic	HIVID	Zhao et al.2016	27703150	Tumor
chr6	66446619		EYS(dist=29501),SLC25A51P1(dist=51153)	intergenic	HIVID	Zhao et al.2016	27703150	Tumor
chr11	121422658		SORL1	intronic	HIVID	Zhao et al.2016	27703150	Tumor
chr21	37020906		LOC100506403(dist=67844),MIR802(dist=72107)	intergenic	HIVID	Zhao et al.2016	27703150	Tumor
chr9	66971092		PTGER4P2-CDK2AP2P2(dist=468062),RP11-381O7.3(dist=4628[illegible]	intergenic	HIVID	Zhao et al.2016	27703150	Tumor
chr18	18520279		NONE(dist=NONE),ROCK1(dist=9424)	intergenic	HIVID	Zhao et al.2016	27703150	Tumor
chr18	18519665		NONE(dist=NONE),ROCK1(dist=10038)	intergenic	HIVID	Zhao et al.2016	27703150	Tumor
chr12	37995094		NONE(dist=NONE),ALG10B(dist=715463)	intergenic	HIVID	Zhao et al.2016	27703150	Tumor
chr5	46359567		HCN1(dist=663347),NONE(dist=NONE)	intergenic	HIVID	Zhao et al.2016	27703150	Tumor
chr9	22712493		FLJ35282	ncRNA_intronic	HIVID	Zhao et al.2016	27703150	Tumor
chr9	22712729		FLJ35282	ncRNA_intronic	HIVID	Zhao et al.2016	27703150	Tumor
chr3	90461656		EPHA3(dist=930372),NONE(dist=NONE)	intergenic	HIVID	Zhao et al.2016	27703150	Tumor
chr3	139893282		CLSTN2	intronic	HIVID	Zhao et al.2016	27703150	Tumor
chr2	92298627		ACTR3BP2(dist=168131),NONE(dist=NONE)	intergenic	HIVID	Zhao et al.2016	27703150	Tumor
chr19	53831725		ZNF845	promoter	HIVID	Zhao et al.2016	27703150	Tumor
chr19	53831769		ZNF845	promoter	HIVID	Zhao et al.2016	27703150	Tumor
chr7	61744286		NONE(dist=NONE),ZNF733P(dist=1007384)	intergenic	HIVID	Zhao et al.2016	27703150	Tumor
chr7	61744324		NONE(dist=NONE),ZNF733P(dist=1007346)	intergenic	HIVID	Zhao et al.2016	27703150	Tumor
chr5	144552806		KCTD16(dist=695862),PRELID2(dist=583101)	intergenic	HIVID	Zhao et al.2016	27703150	Tumor
chr22	48907113		FAM19A5	intronic	HIVID	Zhao et al.2016	27703150	Tumor
chr1	22397725		CDC42	intronic	HIVID	Zhao et al.2016	27703150	Tumor
chr11	100676464		ARHGAP42	intronic	HIVID	Zhao et al.2016	27703150	Tumor
chr5	428156		AHRR	intronic	HIVID	Zhao et al.2016	27703150	Tumor
chr5	425784		AHRR	intronic	HIVID	Zhao et al.2016	27703150	Tumor
chr5	430538		AHRR	intronic	HIVID	Zhao et al.2016	27703150	Tumor
chr3	194048896		LINC00887(dist=18303),CPN2(dist=11598)	intergenic	HIVID	Zhao et al.2016	27703150	Tumor
chr3	193972122		HES1(dist=115721),LINC00887(dist=46867)	intergenic	HIVID	Zhao et al.2016	27703150	Tumor
chr16	17337492		XYLT1	intronic	HIVID	Zhao et al.2016	27703150	Tumor
chr20	46461894		SULF2(dist=46534),RP11-347D21.3(dist=146943)	intergenic	HIVID	Zhao et al.2016	27703150	Tumor
chr20	46526987		SULF2(dist=111627),RP11-347D21.3(dist=81850)	intergenic	HIVID	Zhao et al.2016	27703150	Tumor
chr5	10547		NONE(dist=NONE),PLEKHG4B(dist=129826)	intergenic	HIVID	Zhao et al.2016	27703150	Tumor
chr5	10565		NONE(dist=NONE),PLEKHG4B(dist=129808)	intergenic	HIVID	Zhao et al.2016	27703150	Tumor
chr5	11583		NONE(dist=NONE),PLEKHG4B(dist=128790)	intergenic	HIVID	Zhao et al.2016	27703150	Tumor
chr5	11700		NONE(dist=NONE),PLEKHG4B(dist=128673)	intergenic	HIVID	Zhao et al.2016	27703150	Tumor
chr5	11712		NONE(dist=NONE),PLEKHG4B(dist=128661)	intergenic	HIVID	Zhao et al.2016	27703150	Tumor
chr5	11784		NONE(dist=NONE),PLEKHG4B(dist=128589)	intergenic	HIVID	Zhao et al.2016	27703150	Tumor
chr7	10243		NONE(dist=NONE),LOC100507642(dist=139475)	intergenic	HIVID	Zhao et al.2016	27703150	Tumor
chr11	69167450		MYEOV(dist=102696),CCND1(dist=288423)	intergenic	HIVID	Zhao et al.2016	27703150	Tumor
chr12	95638		LOC100288778(dist=4375),FAM138D(dist=52308)	intergenic	HIVID	Zhao et al.2016	27703150	Tumor
chr12	95613		LOC100288778(dist=4350),FAM138D(dist=52333)	intergenic	HIVID	Zhao et al.2016	27703150	Tumor
chr12	95601		LOC100288778(dist=4338),FAM138D(dist=52345)	intergenic	HIVID	Zhao et al.2016	27703150	Tumor
chr12	95433		LOC100288778(dist=4170),FAM138D(dist=52513)	intergenic	HIVID	Zhao et al.2016	27703150	Tumor
chr11	175784		LINC01001(dist=43864),SCGB1C1(dist=17296)	intergenic	HIVID	Zhao et al.2016	27703150	Tumor
chr20	47128750		LINC00494(dist=129369),PREX1(dist=112043)	intergenic	HIVID	Zhao et al.2016	27703150	Tumor
chr20	47128685		LINC00494(dist=129304),PREX1(dist=112108)	intergenic	HIVID	Zhao et al.2016	27703150	Tumor
chr19	36212811		KMT2B	intronic	HIVID	Zhao et al.2016	27703150	Tumor
chr19	36212832		KMT2B	intronic	HIVID	Zhao et al.2016	27703150	Tumor
chr19	36212921		KMT2B	intronic	HIVID	Zhao et al.2016	27703150	Tumor
chr10	135524466		DUX4L7(dist=26008),NONE(dist=NONE)	intergenic	HIVID	Zhao et al.2016	27703150	Tumor
chr4	191043983		DUX4(dist=30541),NONE(dist=NONE)	intergenic	HIVID	Zhao et al.2016	27703150	Tumor
chr1	10110		DDX11L1;MIR6859-1,MIR6859-2,WASH7P	promoter;downstream	HIVID	Zhao et al.2016	27703150	Tumor
chr4	81894445		C4orf22(dist=9535),BMP3(dist=57674)	intergenic	HIVID	Zhao et al.2016	27703150	Tumor
chr15	80755812		ARNT2	intronic	HIVID	Zhao et al.2016	27703150	Tumor
chr12	133841775		ANHX(dist=29353),NONE(dist=NONE)	intergenic	HIVID	Zhao et al.2016	27703150	Tumor
chr5	1303793		TERT;MIR4457	promoter;downstream	HIVID	Zhao et al.2016	27703150	Tumor
chr5	1295776		TERT	promoter	HIVID	Zhao et al.2016	27703150	Tumor
chr13	105534853		MIR548AS(dist=1600005),DAOA-AS1(dist=576553)	intergenic	HIVID	Zhao et al.2016	27703150	Tumor
chr10	135524737		DUX4L7(dist=26279),NONE(dist=NONE)	intergenic	HIVID	Zhao et al.2016	27703150	Tumor
chr10	135524715		DUX4L7(dist=26257),NONE(dist=NONE)	intergenic	HIVID	Zhao et al.2016	27703150	Tumor
chr16	32701653		TP53TG3B(dist=14205),SLC6A10P(dist=187144)	intergenic	HIVID	Zhao et al.2016	27703150	Tumor
chr16	32701607		TP53TG3B(dist=14159),SLC6A10P(dist=187190)	intergenic	HIVID	Zhao et al.2016	27703150	Tumor
chr12	23792880		SOX5	intronic	HIVID	Zhao et al.2016	27703150	Tumor
chr21	48119806		PRMT2(dist=34770),NONE(dist=NONE)	intergenic	HIVID	Zhao et al.2016	27703150	Tumor
chr21	48119752		PRMT2(dist=34716),NONE(dist=NONE)	intergenic	HIVID	Zhao et al.2016	27703150	Tumor
chr16	47754992		PHKB(dist=19558),ABCC12(dist=361892)	intergenic	HIVID	Zhao et al.2016	27703150	Tumor
chr1	249240050		PGBD2(dist=26705),NONE(dist=NONE)	intergenic	HIVID	Zhao et al.2016	27703150	Tumor
chr1	249239950		PGBD2(dist=26605),NONE(dist=NONE)	intergenic	HIVID	Zhao et al.2016	27703150	Tumor
chr8	66730370		PDE7A	intronic	HIVID	Zhao et al.2016	27703150	Tumor
chr18	78016326		PARD6G(dist=10929),NONE(dist=NONE)	intergenic	HIVID	Zhao et al.2016	27703150	Tumor
chr18	78016280		PARD6G(dist=10883),NONE(dist=NONE)	intergenic	HIVID	Zhao et al.2016	27703150	Tumor
chr18	78016257		PARD6G(dist=10860),NONE(dist=NONE)	intergenic	HIVID	Zhao et al.2016	27703150	Tumor
chr18	78016230		PARD6G(dist=10833),NONE(dist=NONE)	intergenic	HIVID	Zhao et al.2016	27703150	Tumor
chr18	78016206		PARD6G(dist=10809),NONE(dist=NONE)	intergenic	HIVID	Zhao et al.2016	27703150	Tumor
chr5	11680		NONE(dist=NONE),PLEKHG4B(dist=128693)	intergenic	HIVID	Zhao et al.2016	27703150	Tumor
chr5	1314962		MIR4457;CLPTM1L	promoter;downstream	HIVID	Zhao et al.2016	27703150	Tumor
chr11	191897		LOC653486,ODF3,SCGB1C1	promoter	HIVID	Zhao et al.2016	27703150	Tumor
chr11	191805		LOC653486,ODF3,SCGB1C1	promoter	HIVID	Zhao et al.2016	27703150	Tumor
chr12	95531		LOC100288778(dist=4268),FAM138D(dist=52415)	intergenic	HIVID	Zhao et al.2016	27703150	Tumor
chr16	32250465		HERC2P4(dist=51031),TP53TG3D(dist=14185)	intergenic	HIVID	Zhao et al.2016	27703150	Tumor
chr16	32250419		HERC2P4(dist=50985),TP53TG3D(dist=14231)	intergenic	HIVID	Zhao et al.2016	27703150	Tumor
chr10	135524675		DUX4L7(dist=26217),NONE(dist=NONE)	intergenic	HIVID	Zhao et al.2016	27703150	Tumor
chr4	191044238		DUX4(dist=30796),NONE(dist=NONE)	intergenic	HIVID	Zhao et al.2016	27703150	Tumor
chr9	10079		DDX11L5;WASH1	promoter;downstream	HIVID	Zhao et al.2016	27703150	Tumor
chr9	10184		DDX11L5;WASH1	promoter;downstream	HIVID	Zhao et al.2016	27703150	Tumor
chr1	10239		DDX11L1;MIR6859-1,MIR6859-2,WASH7P	promoter;downstream	HIVID	Zhao et al.2016	27703150	Tumor
chr1	10333		DDX11L1;MIR6859-1,MIR6859-2,WASH7P	promoter;downstream	HIVID	Zhao et al.2016	27703150	Tumor
chr21	27577221		APP(dist=33775),CYYR1(dist=261307)	intergenic	HIVID	Zhao et al.2016	27703150	Tumor

Chromosome	Integration site in host genome	Integration site in virus genome	Gene (distance, bp)	Regions	Methods	Author	PMID	Sample
chr10	5027215		AKR1C1(dist=7057),AKR1C2(dist=2753)	intergenic	HIVID	Zhao et al.2016	27703150	Tumor
chr9	118066667		DEC1	intronic	HIVID	Zhao et al.2016	27703150	Tumor
chr9	118073554		DEC1	intronic	HIVID	Zhao et al.2016	27703150	Tumor
chr1	121485133		EMBP1(dist=171447),NONE(dist=NONE)	intergenic	HIVID	Zhao et al.2016	27703150	Tumor
chr1	121484114		EMBP1(dist=170428),NONE(dist=NONE)	intergenic	HIVID	Zhao et al.2016	27703150	Tumor
chr1	194896702		CDC73(dist=1672760),KCNT2(dist=1298208)	intergenic	HIVID	Zhao et al.2016	27703150	Tumor
chr9	38120925		SHB(dist=51715),ALDH1B1(dist=271736)	intergenic	HIVID	Zhao et al.2016	27703150	Tumor
chr5	145855469		TCERG1	intronic	HIVID	Zhao et al.2016	27703150	Tumor
chr5	132781090		FSTL4	intronic	HIVID	Zhao et al.2016	27703150	Tumor
chr5	1292401		TERT	intronic	HIVID	Zhao et al.2016	27703150	Tumor
chr3	179343887		NDUFB5(dist=1599),USP13(dist=27046)	intergenic	HIVID	Zhao et al.2016	27703150	Tumor
chr12	66677753		IRAK3(dist=29359),HELB(dist=18582)	intergenic	HIVID	Zhao et al.2016	27703150	Tumor
chr12	66241628		HMGA2	intronic	HIVID	Zhao et al.2016	27703150	Tumor
chr5	10000		NONE(dist=NONE),PLEKHG4B(dist=130373)	intergenic	HIVID	Zhao et al.2016	27703150	Tumor
chr16	46404256		NONE(dist=NONE),ANKRD26P1(dist=98993)	intergenic	HIVID	Zhao et al.2016	27703150	Tumor
chr16	46404502		NONE(dist=NONE),ANKRD26P1(dist=98747)	intergenic	HIVID	Zhao et al.2016	27703150	Tumor
chr16	46428198		NONE(dist=NONE),ANKRD26P1(dist=75051)	intergenic	HIVID	Zhao et al.2016	27703150	Tumor
chr16	46428543		NONE(dist=NONE),ANKRD26P1(dist=74706)	intergenic	HIVID	Zhao et al.2016	27703150	Tumor
chr17	22245451		MTRNR2L1(dist=221460),NONE(dist=NONE)	intergenic	HIVID	Zhao et al.2016	27703150	Tumor
chr16	33963435		LINC00273	promoter	HIVID	Zhao et al.2016	27703150	Tumor
chr6	55110069		HCRTR2	intronic	HIVID	Zhao et al.2016	27703150	Tumor
chr6	54933629		FAM83B(dist=123732),HCRTR2(dist=105442)	intergenic	HIVID	Zhao et al.2016	27703150	Tumor
chr2	133030533		ANKRD30BL(dist=14991),GPR39(dist=143614)	intergenic	HIVID	Zhao et al.2016	27703150	Tumor
chr2	133012843		ANKRD30BL	ncRNA_intronic	HIVID	Zhao et al.2016	27703150	Tumor
chr5	1295416		TERT	promoter	HIVID	Zhao et al.2016	27703150	Tumor
chr5	1295449		TERT	promoter	HIVID	Zhao et al.2016	27703150	Tumor
chr4	168235462		SPOCK3(dist=79721),ANXA10(dist=778226)	intergenic	HIVID	Zhao et al.2016	27703150	Tumor
chr2	40228639		SLC8A1-AS1	ncRNA_intronic	HIVID	Zhao et al.2016	27703150	Tumor
chr2	40228665		SLC8A1-AS1	ncRNA_intronic	HIVID	Zhao et al.2016	27703150	Tumor
chr19	31510569		ZNF536(dist=461604),DKFZp566F0947(dist=130214)	intergenic	HIVID	Zhao et al.2016	27703150	Tumor
chr3	147638617		ZIC1(dist=504111),AGTR1(dist=777041)	intergenic	HIVID	Zhao et al.2016	27703150	Tumor
chr8	123942221		ZHX2	intronic	HIVID	Zhao et al.2016	27703150	Tumor
chr2	98856030		VWA3B	intronic	HIVID	Zhao et al.2016	27703150	Tumor
chr8	35473157		UNC5D	intronic	HIVID	Zhao et al.2016	27703150	Tumor
chr7	2662198		TTYH3;IQCE	promoter;downstream	HIVID	Zhao et al.2016	27703150	Tumor
chr2	120456756		TMEM177(dist=17062),PTPN4(dist=60451)	intergenic	HIVID	Zhao et al.2016	27703150	Tumor
chr5	1295175		TERT	promoter	HIVID	Zhao et al.2016	27703150	Tumor
chr7	89325062		STEAP2-AS1	ncRNA_intronic	HIVID	Zhao et al.2016	27703150	Tumor
chr7	127721392		SND1	intronic	HIVID	Zhao et al.2016	27703150	Tumor
chr3	113336238		SIDT1	intronic	HIVID	Zhao et al.2016	27703150	Tumor
chr10	77257531		RP11-399K21.14(dist=86449),C10orf11(dist=284988)	intergenic	HIVID	Zhao et al.2016	27703150	Tumor
chr3	168748150		RP11-368I23.2(dist=108369),MECOM(dist=53137)	intergenic	HIVID	Zhao et al.2016	27703150	Tumor
chr3	168748108		RP11-368I23.2(dist=108327),MECOM(dist=53179)	intergenic	HIVID	Zhao et al.2016	27703150	Tumor
chr3	64561235		RP11-14D22.5	ncRNA_intronic	HIVID	Zhao et al.2016	27703150	Tumor
chr9	134537621		RAPGEF1	intronic	HIVID	Zhao et al.2016	27703150	Tumor
chr19	41336720		RAB4B-EGLN2(dist=22374),CYP2A6(dist=12723)	intergenic	HIVID	Zhao et al.2016	27703150	Tumor
chr1	170779185		PRRX1(dist=70644),MROH9(dist=125427)	intergenic	HIVID	Zhao et al.2016	27703150	Tumor
chr6	109724222		PPIL6	intronic	HIVID	Zhao et al.2016	27703150	Tumor
chr7	39572486		POU6F2(dist=68096),YAE1D1(dist=33489)	intergenic	HIVID	Zhao et al.2016	27703150	Tumor
chr12	41926432		PDZRN4	intronic	HIVID	Zhao et al.2016	27703150	Tumor
chr12	41869446		PDZRN4	intronic	HIVID	Zhao et al.2016	27703150	Tumor
chr8	117420		OR4F21	promoter	HIVID	Zhao et al.2016	27703150	Tumor
chr13	57230579		MIR5007(dist=1481896),PRR20A(dist=484473)	intergenic	HIVID	Zhao et al.2016	27703150	Tumor
chr8	129821686		MIR1208(dist=659252),LINC00977(dist=407027)	intergenic	HIVID	Zhao et al.2016	27703150	Tumor
chr9	130227284		LRSAM1	intronic	HIVID	Zhao et al.2016	27703150	Tumor
chr5	18215609		LOC401177(dist=828190),CDH18(dist=1257546)	intergenic	HIVID	Zhao et al.2016	27703150	Tumor
chr5	17698940		LOC401177(dist=311521),CDH18(dist=1774215)	intergenic	HIVID	Zhao et al.2016	27703150	Tumor
chr5	17698269		LOC401177(dist=310850),CDH18(dist=1774886)	intergenic	HIVID	Zhao et al.2016	27703150	Tumor
chr14	62783251		LINC00644(dist=176560),KCNH5(dist=390040)	intergenic	HIVID	Zhao et al.2016	27703150	Tumor
chr4	43479355		GRXCR1(dist=446680),KCTD8(dist=696565)	intergenic	HIVID	Zhao et al.2016	27703150	Tumor
chr13	101243807		GGACT	promoter	HIVID	Zhao et al.2016	27703150	Tumor
chr13	101243775		GGACT	promoter	HIVID	Zhao et al.2016	27703150	Tumor
chr5	44598417		FGF10-AS1(dist=184326),RP11-53O19.1(dist=146013)	intergenic	HIVID	Zhao et al.2016	27703150	Tumor
chr5	44598381		FGF10-AS1(dist=184290),RP11-53O19.1(dist=146049)	intergenic	HIVID	Zhao et al.2016	27703150	Tumor
chr7	19503945		FERD3L(dist=318901),TWISTNB(dist=231140)	intergenic	HIVID	Zhao et al.2016	27703150	Tumor
chr12	15947636		EPS8	promoter	HIVID	Zhao et al.2016	27703150	Tumor
chr3	58207783		DNASE1L3(dist=11053),ABHD6(dist=15476)	intergenic	HIVID	Zhao et al.2016	27703150	Tumor
chr1	16965766		CROCCP2,MST1P2	promoter	HIVID	Zhao et al.2016	27703150	Tumor
chr22	46821433		CELSR1	intronic	HIVID	Zhao et al.2016	27703150	Tumor
chr5	90892781		ARRDC3-AS1(dist=176249),NR2F1-AS1(dist=1852281)	intergenic	HIVID	Zhao et al.2016	27703150	Tumor
chr5	90892742		ARRDC3-AS1(dist=176210),NR2F1-AS1(dist=1852320)	intergenic	HIVID	Zhao et al.2016	27703150	Tumor
chr10	28186462		ARMC4	intronic	HIVID	Zhao et al.2016	27703150	Tumor
chr16	71519890		ZNF19	intronic	HIVID	Zhao et al.2016	27703150	Tumor
chr1	203790410		ZC3H11A	intronic	HIVID	Zhao et al.2016	27703150	Tumor
chr7	73314106		WBSCR28(dist=33883),ELN(dist=128013)	intergenic	HIVID	Zhao et al.2016	27703150	Tumor
chr5	1295560		TERT	promoter	HIVID	Zhao et al.2016	27703150	Tumor
chr13	81151195		SPRY2(dist=236109),NONE(dist=NONE)	intergenic	HIVID	Zhao et al.2016	27703150	Tumor
chr4	68265622		RP11-807H7.1(dist=1706518),CENPC(dist=72367)	intergenic	HIVID	Zhao et al.2016	27703150	Tumor
chr5	25284750		RP11-730N24.1(dist=444058),CDH9(dist=1595959)	intergenic	HIVID	Zhao et al.2016	27703150	Tumor
chr7	75585401		POR	intronic	HIVID	Zhao et al.2016	27703150	Tumor
chr4	95627355		PDLIM5(dist=37977),BMPR1B(dist=51773)	intergenic	HIVID	Zhao et al.2016	27703150	Tumor
chr11	55436955		OR4C6(dist=3383),OR5D13(dist=103959)	intergenic	HIVID	Zhao et al.2016	27703150	Tumor
chr2	10670516		ODC1(dist=81836),NOL10(dist=40376)	intergenic	HIVID	Zhao et al.2016	27703150	Tumor
chr7	61968586		NONE(dist=NONE),ZNF733P(dist=783084)	intergenic	HIVID	Zhao et al.2016	27703150	Tumor
chr19	27734485		NONE(dist=NONE),LINC00662(dist=546916)	intergenic	HIVID	Zhao et al.2016	27703150	Tumor
chr19	27738552		NONE(dist=NONE),LINC00662(dist=542849)	intergenic	HIVID	Zhao et al.2016	27703150	Tumor
chr19	27740244		NONE(dist=NONE),LINC00662(dist=541157)	intergenic	HIVID	Zhao et al.2016	27703150	Tumor
chr21	47648163		LSS	intronic	HIVID	Zhao et al.2016	27703150	Tumor
chr21	24565362		LINC00308(dist=1076515),D21S2088E(dist=168064)	intergenic	HIVID	Zhao et al.2016	27703150	Tumor
chr7	36421098		KIAA0895	intronic	HIVID	Zhao et al.2016	27703150	Tumor
chr3	42841649		HIGD1A	intronic	HIVID	Zhao et al.2016	27703150	Tumor
chr4	174229760		GALNT7	intronic	HIVID	Zhao et al.2016	27703150	Tumor
chr20	25824872		FAM182B(dist=42945),LOC101926935(dist=431)	intergenic	HIVID	Zhao et al.2016	27703150	Tumor
chr20	25824833		FAM182B(dist=42906),LOC101926935(dist=470)	intergenic	HIVID	Zhao et al.2016	27703150	Tumor

Chromosome	Integration site in host genome	Integration site in virus genome	Gene (distance, bp)	Regions	Methods	Author	PMID	Sample
chr1	121484780		EMBP1(dist=171094),NONE(dist=NONE)	intergenic	HIVID	Zhao et al.2016	27703150	Tumor
chr1	121484453		EMBP1(dist=170767),NONE(dist=NONE)	intergenic	HIVID	Zhao et al.2016	27703150	Tumor
chr10	22455303		DNAJC1(dist=162653),EBLN1(dist=42440)	intergenic	HIVID	Zhao et al.2016	27703150	Tumor
chr19	30303435		CCNE1	intronic	HIVID	Zhao et al.2016	27703150	Tumor
chr19	30303135		CCNE1	intronic	HIVID	Zhao et al.2016	27703150	Tumor
chr19	30303517		CCNE1	intronic	HIVID	Zhao et al.2016	27703150	Tumor
chr19	30303496		CCNE1	intronic	HIVID	Zhao et al.2016	27703150	Tumor
chr7	71530008		CALN1	intronic	HIVID	Zhao et al.2016	27703150	Tumor
chr5	393004		AHRR	intronic	HIVID	Zhao et al.2016	27703150	Tumor
chr2	8046608		AC007463.2	promoter	HIVID	Zhao et al.2016	27703150	Tumor
chr2	8046646		AC007463.2	promoter	HIVID	Zhao et al.2016	27703150	Tumor
chr17	18175848		MIEF2(dist=6753),TOP3A(dist=1387)	intergenic	HIVID	Zhao et al.2016	27703150	Tumor
chr15	34391947		EMC7	intronic	HIVID	Zhao et al.2016	27703150	Tumor
chr19	58289084		ZNF586	intronic	HIVID	Zhao et al.2016	27703150	Tumor
chr19	58289116		ZNF586	intronic	HIVID	Zhao et al.2016	27703150	Tumor
chr8	56692775		TGS1	intronic	HIVID	Zhao et al.2016	27703150	Tumor
chr6	83965335		ME1	intronic	HIVID	Zhao et al.2016	27703150	Tumor
chr6	84030784		ME1	intronic	HIVID	Zhao et al.2016	27703150	Tumor
chr21	22729127		NCAM2	intronic	HIVID	Zhao et al.2016	27703150	Tumor
chr21	22700059		NCAM2	intronic	HIVID	Zhao et al.2016	27703150	Tumor
chr13	106574177		LINC00343(dist=160034),LINC00460(dist=454734)	intergenic	HIVID	Zhao et al.2016	27703150	Tumor
chr3	2189976		CNTN4	intronic	HIVID	Zhao et al.2016	27703150	Tumor
chr11	65719622		TSGA10IP	intronic	HIVID	Zhao et al.2016	27703150	Tumor
chr9	66971200		PTGER4P2-CDK2AP2P2(dist=468170),RP11-381O7.3(dist=4617[illegible]	intergenic	HIVID	Zhao et al.2016	27703150	Tumor
chr5	1295625		TERT	promoter	HIVID	Zhao et al.2016	27703150	Tumor
chr20	32582608		RALY	intronic	HIVID	Zhao et al.2016	27703150	Tumor
chr8	48706388		PRKDC	intronic	HIVID	Zhao et al.2016	27703150	Tumor
chr16	17236325		XYLT1	intronic	HIVID	Zhao et al.2016	27703150	Tumor
chr16	17234963		XYLT1	intronic	HIVID	Zhao et al.2016	27703150	Tumor
chr18	14695844		POTEC(dist=152245),ANKRD30B(dist=52395)	intergenic	HIVID	Zhao et al.2016	27703150	Tumor
chr4	138349771		LINC00613(dist=1514936),PCDH18(dist=90303)	intergenic	HIVID	Zhao et al.2016	27703150	Tumor
chr1	25364215		RUNX3(dist=72714),SYF2(dist=184552)	intergenic	HIVID	Zhao et al.2016	27703150	Tumor
chr1	25392607		RUNX3(dist=101106),SYF2(dist=156160)	intergenic	HIVID	Zhao et al.2016	27703150	Tumor
chr1	25392584		RUNX3(dist=101083),SYF2(dist=156183)	intergenic	HIVID	Zhao et al.2016	27703150	Tumor
chr1	25392522		RUNX3(dist=101021),SYF2(dist=156245)	intergenic	HIVID	Zhao et al.2016	27703150	Tumor
chr5	177804498		COL23A1	intronic	HIVID	Zhao et al.2016	27703150	Tumor
chr5	1295267		TERT	promoter	HIVID	Zhao et al.2016	27703150	Tumor
chr5	1295218		TERT	promoter	HIVID	Zhao et al.2016	27703150	Tumor
chr5	1295202		TERT	promoter	HIVID	Zhao et al.2016	27703150	Tumor
chr5	1295263		TERT	promoter	HIVID	Zhao et al.2016	27703150	Tumor
chr17	18764582		PRPSAP2	intronic	HIVID	Zhao et al.2016	27703150	Tumor
chr8	54380909		OPRK1(dist=216652),ATP6V1H(dist=247194)	intergenic	HIVID	Zhao et al.2016	27703150	Tumor
chr8	54350100		OPRK1(dist=185843),ATP6V1H(dist=278003)	intergenic	HIVID	Zhao et al.2016	27703150	Tumor
chr11	63161833		MIR3680-1,MIR3680-2	ncRNA_intronic	HIVID	Zhao et al.2016	27703150	Tumor
chr2	155382315		AC009227.2(dist=68365),KCNJ3(dist=172778)	intergenic	HIVID	Zhao et al.2016	27703150	Tumor
chr2	155378561		AC009227.2(dist=64611),KCNJ3(dist=176532)	intergenic	HIVID	Zhao et al.2016	27703150	Tumor
chr5	157907401		LOC101927697(dist=70620),EBF1(dist=215522)	intergenic	HIVID	Zhao et al.2016	27703150	Tumor
chr6	1496549		FOXF2(dist=100717),FOXC1(dist=114132)	intergenic	HIVID	Zhao et al.2016	27703150	Tumor
chr5	171284619		FGF18(dist=399989),FBXW11(dist=3937)	intergenic	HIVID	Zhao et al.2016	27703150	Tumor
chr6	5617257		FARS2	intronic	HIVID	Zhao et al.2016	27703150	Tumor
chr19	54154083		DPRX(dist=13820),MIR512-2(dist=15844)	intergenic	HIVID	Zhao et al.2016	27703150	Tumor
chr4	47507716		ATP10D	intronic	HIVID	Zhao et al.2016	27703150	Tumor
chr8	84506156		SNX16(dist=1751635),RALYL(dist=589297)	intergenic	HIVID	Zhao et al.2016	27703150	Tumor
chr12	111880797		SH2B3	intronic	HIVID	Zhao et al.2016	27703150	Tumor
chr12	111880825		SH2B3	intronic	HIVID	Zhao et al.2016	27703150	Tumor
chr3	196625759		SENP5	intronic	HIVID	Zhao et al.2016	27703150	Tumor
chr11	51573396		OR4C46(dist=57185),NONE(dist=NONE)	intergenic	HIVID	Zhao et al.2016	27703150	Tumor
chr3	174356473		NLGN1(dist=355334),NAALADL2(dist=220638)	intergenic	HIVID	Zhao et al.2016	27703150	Tumor
chr17	22261559		MTRNR2L1(dist=237568),NONE(dist=NONE)	intergenic	HIVID	Zhao et al.2016	27703150	Tumor
chr17	22261236		MTRNR2L1(dist=237245),NONE(dist=NONE)	intergenic	HIVID	Zhao et al.2016	27703150	Tumor
chr17	22260917		MTRNR2L1(dist=236926),NONE(dist=NONE)	intergenic	HIVID	Zhao et al.2016	27703150	Tumor
chr17	22260816		MTRNR2L1(dist=236825),NONE(dist=NONE)	intergenic	HIVID	Zhao et al.2016	27703150	Tumor
chr17	22249026		MTRNR2L1(dist=225035),NONE(dist=NONE)	intergenic	HIVID	Zhao et al.2016	27703150	Tumor
chr16	59709356		GOT2(dist=941095),APOOP5(dist=78689)	intergenic	HIVID	Zhao et al.2016	27703150	Tumor
chr4	191043680		DUX4(dist=30238),NONE(dist=NONE)	intergenic	HIVID	Zhao et al.2016	27703150	Tumor
chr4	191043586		DUX4(dist=30144),NONE(dist=NONE)	intergenic	HIVID	Zhao et al.2016	27703150	Tumor
chr12	105712305		APPL2(dist=82297),C12orf75(dist=12109)	intergenic	HIVID	Zhao et al.2016	27703150	Tumor
chr2	106944539		UXS1(dist=133744),PLGLA(dist=54031)	intergenic	HIVID	Zhao et al.2016	27703150	Tumor
chr5	1296624		TERT	promoter	HIVID	Zhao et al.2016	27703150	Tumor
chr2	103201985		SLC9A4(dist=51554),SLC9A2(dist=34181)	intergenic	HIVID	Zhao et al.2016	27703150	Tumor
chr1	4110083		RP13-614K11.1(dist=97440),RP5-1166F10.1(dist=362028)	intergenic	HIVID	Zhao et al.2016	27703150	Tumor
chr10	89995347		PTEN(dist=266815),RNLS(dist=38274)	intergenic	HIVID	Zhao et al.2016	27703150	Tumor
chr5	118469		NONE(dist=NONE),PLEKHG4B(dist=21904)	intergenic	HIVID	Zhao et al.2016	27703150	Tumor
chr7	112979202		LINC00998(dist=220565),PPP1R3A(dist=537680)	intergenic	HIVID	Zhao et al.2016	27703150	Tumor
chr8	28455384		FZD3(dist=23599),EXTL3(dist=103606)	intergenic	HIVID	Zhao et al.2016	27703150	Tumor
chr2	216351907		FN1(dist=51116),LINC00607(dist=124379)	intergenic	HIVID	Zhao et al.2016	27703150	Tumor
chr1	217352039		ESRRG(dist=40942),GPATCH2(dist=251795)	intergenic	HIVID	Zhao et al.2016	27703150	Tumor
chr5	20914114		CDH18(dist=925761),GUSBP1(dist=545475)	intergenic	HIVID	Zhao et al.2016	27703150	Tumor
chr5	34288319		C1QTNF3-AMACR(dist=163686),RAI14(dist=368114)	intergenic	HIVID	Zhao et al.2016	27703150	Tumor
chr3	193036412		ATP13A5	intronic	HIVID	Zhao et al.2016	27703150	Tumor
chr13	59828449		PCDH17(dist=1525384),DIAPH3(dist=411272)	intergenic	HIVID	Zhao et al.2016	27703150	Tumor
chr13	59827939		PCDH17(dist=1524874),DIAPH3(dist=411782)	intergenic	HIVID	Zhao et al.2016	27703150	Tumor
chr2	124653556		NONE(dist=NONE),CNTNAP5(dist=129308)	intergenic	HIVID	Zhao et al.2016	27703150	Tumor
chr11	50739124		LOC646813(dist=359322),OR4A5(dist=672254)	intergenic	HIVID	Zhao et al.2016	27703150	Tumor
chr19	11477600		SWSAP1;LPPR2	promoter;downstream	HIVID	Zhao et al.2016	27703150	Tumor
chr1	211767444		SLC30A1(dist=15345),NEK2(dist=64155)	intergenic	HIVID	Zhao et al.2016	27703150	Tumor
chr9	66971262		PTGER4P2-CDK2AP2P2(dist=468232),RP11-381O7.3(dist=4611[illegible]	intergenic	HIVID	Zhao et al.2016	27703150	Tumor
chr9	112411309		PALM2	intronic	HIVID	Zhao et al.2016	27703150	Tumor
chr16	46408203		NONE(dist=NONE),ANKRD26P1(dist=95046)	intergenic	HIVID	Zhao et al.2016	27703150	Tumor
chr16	46402114		NONE(dist=NONE),ANKRD26P1(dist=101135)	intergenic	HIVID	Zhao et al.2016	27703150	Tumor
chr7	63258465		MIR4283-1(dist=176918),LINC01005(dist=226331)	intergenic	HIVID	Zhao et al.2016	27703150	Tumor
chr7	56847807		LOC101928401(dist=241984),LOC100130849(dist=95271)	intergenic	HIVID	Zhao et al.2016	27703150	Tumor
chr2	87792696		LINC00152	ncRNA_intronic	HIVID	Zhao et al.2016	27703150	Tumor
chr6	32508649		HLA-DRB5(dist=10643),HLA-DRB6(dist=11841)	intergenic	HIVID	Zhao et al.2016	27703150	Tumor

Chromosome	Integration site in host genome	Integration site in virus genome	Gene (distance, bp)	Regions	Methods	Author	PMID	Sample
chr17	9702645		DHRS7C	promoter	HIVID	Zhao et al.2016	27703150	Tumor
chr2	56747084		CCDC85A(dist=133775),VRK2(dist=1387702)	intergenic	HIVID	Zhao et al.2016	27703150	Tumor
chr3	8094599		AC087859.1(dist=36605),LMCD1-AS1(dist=168235)	intergenic	HIVID	Zhao et al.2016	27703150	Tumor
chr2	47309766		TTC7A(dist=6491),C2orf61(dist=4364)	intergenic	HIVID	Zhao et al.2016	27703150	Tumor
chr5	1295538		TERT	promoter	HIVID	Zhao et al.2016	27703150	Tumor
chr1	249239808		PGBD2(dist=26463),NONE(dist=NONE)	intergenic	HIVID	Zhao et al.2016	27703150	Tumor
chr1	249239755		PGBD2(dist=26410),NONE(dist=NONE)	intergenic	HIVID	Zhao et al.2016	27703150	Tumor
chr4	191044274		DUX4(dist=30832),NONE(dist=NONE)	intergenic	HIVID	Zhao et al.2016	27703150	Tumor
chr9	10341		DDX11L5;WASH1	promoter;downstream	HIVID	Zhao et al.2016	27703150	Tumor
chr4	49120896		CWH43(dist=56801),NONE(dist=NONE)	intergenic	HIVID	Zhao et al.2016	27703150	Tumor
chr4	49106837		CWH43(dist=42742),NONE(dist=NONE)	intergenic	HIVID	Zhao et al.2016	27703150	Tumor
chr4	49100216		CWH43(dist=36121),NONE(dist=NONE)	intergenic	HIVID	Zhao et al.2016	27703150	Tumor
chr5	1295200		TERT	promoter	HIVID	Zhao et al.2016	27703150	Tumor
chr5	1295149		TERT	UTR5	HIVID	Zhao et al.2016	27703150	Tumor
chr5	1295263		TERT	promoter	HIVID	Zhao et al.2016	27703150	Tumor
chr5	1295235		TERT	promoter	HIVID	Zhao et al.2016	27703150	Tumor
chr1	121113600		SRGAP2-AS1	ncRNA_intronic	HIVID	Zhao et al.2016	27703150	Tumor
chr1	206563829		SRGAP2,SRGAP2B,SRGAP2C,SRGAP2D	intronic	HIVID	Zhao et al.2016	27703150	Tumor
chr12	90979716		LINC00936(dist=873987),LINC00615(dist=332084)	intergenic	HIVID	Zhao et al.2016	27703150	Tumor
chr12	90979684		LINC00936(dist=873955),LINC00615(dist=332116)	intergenic	HIVID	Zhao et al.2016	27703150	Tumor
chr5	833246		ZDHHC11	intronic	HIVID	Zhao et al.2016	27703150	Tumor
chr13	20034369		TPTE2	intronic	HIVID	Zhao et al.2016	27703150	Tumor
chr2	103121603		SLC9A4	intronic	HIVID	Zhao et al.2016	27703150	Tumor
chr6	57844773		PRIM2(dist=331397),GUSBP4(dist=401386)	intergenic	HIVID	Zhao et al.2016	27703150	Tumor
chr6	57843306		PRIM2(dist=329930),GUSBP4(dist=402853)	intergenic	HIVID	Zhao et al.2016	27703150	Tumor
chr2	195102599		PCGEM1(dist=1460974),AC018799.1(dist=106394)	intergenic	HIVID	Zhao et al.2016	27703150	Tumor
chr2	243152582		LOC728323(dist=50113),NONE(dist=NONE)	intergenic	HIVID	Zhao et al.2016	27703150	Tumor
chr8	93777978		FLJ46284	ncRNA_intronic	HIVID	Zhao et al.2016	27703150	Tumor
chr5	1291916		TERT	intronic	HIVID	Zhao et al.2016	27703150	Tumor
chr8	42448033		SMIM19(dist=39893),CHRNB3(dist=104529)	intergenic	HIVID	Zhao et al.2016	27703150	Tumor
chr12	103909790		C12orf42(dist=20002),RP11-626I20.3(dist=31782)	intergenic	HIVID	Zhao et al.2016	27703150	Tumor
chr17	56117112		SRSF1(dist=32405),DYNLL2(dist=43668)	intergenic	HIVID	Zhao et al.2016	27703150	Tumor
chr1	151643728		SNX27	intronic	HIVID	Zhao et al.2016	27703150	Tumor
chr3	62217516		PTPRG	intronic	HIVID	Zhao et al.2016	27703150	Tumor
chr3	62217530		PTPRG	intronic	HIVID	Zhao et al.2016	27703150	Tumor
chr17	81089193		METRNL(dist=36602),FLJ43681(dist=85473)	intergenic	HIVID	Zhao et al.2016	27703150	Tumor
chr17	81070363		METRNL(dist=17772),FLJ43681(dist=104303)	intergenic	HIVID	Zhao et al.2016	27703150	Tumor
chr22	36024264		MB	promoter	HIVID	Zhao et al.2016	27703150	Tumor
chr4	122743388		CCNA2	intronic	HIVID	Zhao et al.2016	27703150	Tumor
chr17	66256018		ARSG	intronic	HIVID	Zhao et al.2016	27703150	Tumor
chr13	34239715		STARD13	intronic	HIVID	Zhao et al.2016	27703150	Tumor
chr5	1295454		TERT	promoter	HIVID	Zhao et al.2016	27703150	Tumor
chr5	1295492		TERT	promoter	HIVID	Zhao et al.2016	27703150	Tumor
chr11	51576967		OR4C46(dist=60756),NONE(dist=NONE)	intergenic	HIVID	Zhao et al.2016	27703150	Tumor
chr17	22252822		MTRNR2L1(dist=228831),NONE(dist=NONE)	intergenic	HIVID	Zhao et al.2016	27703150	Tumor
chr17	22250439		MTRNR2L1(dist=226448),NONE(dist=NONE)	intergenic	HIVID	Zhao et al.2016	27703150	Tumor
chr17	22248060		MTRNR2L1(dist=224069),NONE(dist=NONE)	intergenic	HIVID	Zhao et al.2016	27703150	Tumor
chr1	121359109		EMBP1(dist=45423),NONE(dist=NONE)	intergenic	HIVID	Zhao et al.2016	27703150	Tumor
chr1	121354369		EMBP1(dist=40683),NONE(dist=NONE)	intergenic	HIVID	Zhao et al.2016	27703150	Tumor
chr1	121478985		EMBP1(dist=165299),NONE(dist=NONE)	intergenic	HIVID	Zhao et al.2016	27703150	Tumor
chr1	63213698		DOCK7(dist=59659),ATG4C(dist=36079)	intergenic	HIVID	Zhao et al.2016	27703150	Tumor
chr1	63213694		DOCK7(dist=59655),ATG4C(dist=36083)	intergenic	HIVID	Zhao et al.2016	27703150	Tumor
chr5	21234370		CDH18(dist=1246017),GUSBP1(dist=225219)	intergenic	HIVID	Zhao et al.2016	27703150	Tumor
chr5	21234367		CDH18(dist=1246014),GUSBP1(dist=225222)	intergenic	HIVID	Zhao et al.2016	27703150	Tumor
chr8	132492876		ADCY8(dist=440041),EFR3A(dist=423480)	intergenic	HIVID	Zhao et al.2016	27703150	Tumor
chr1	10188	900	NONE(dist=NONE),DDX11L1(dist=1686)	intergenic	HIVID	Yang et al .2018	30271481	Tumor
chr1	10352	900	NONE(dist=NONE),DDX11L1(dist=1522)	intergenic	HIVID	Yang et al .2018	30271481	Tumor
chr1	10629	409	NONE(dist=NONE),DDX11L1(dist=1245)	intergenic	HIVID	Yang et al .2018	30271481	Tumor
chr1	10709	403	NONE(dist=NONE),DDX11L1(dist=1165)	intergenic	HIVID	Yang et al .2018	30271481	Tumor
chr1	10839	409	NONE(dist=NONE),DDX11L1(dist=1035)	intergenic	HIVID	Yang et al .2018	30271481	Tumor
chr1	15677949	2298	FHAD1	intronic	HIVID	Yang et al .2018	30271481	Tumor
chr1	121484558	694	EMBP1(dist=170872),NONE(dist=NONE)	intergenic	HIVID	Yang et al .2018	30271481	Tumor
chr1	187981102	609	PLA2G4A(dist=1022989),NONE(dist=NONE)	intergenic	HIVID	Yang et al .2018	30271481	Tumor
chr1	219274634	2948	LOC643723	ncRNA_intronic	HIVID	Yang et al .2018	30271481	Tumor
chr1	249239899	900	PGBD2(dist=26554),NONE(dist=NONE)	intergenic	HIVID	Yang et al .2018	30271481	Tumor
chr1	249240173	900	PGBD2(dist=26828),NONE(dist=NONE)	intergenic	HIVID	Yang et al .2018	30271481	Tumor
chr1	249240297	900	PGBD2(dist=26952),NONE(dist=NONE)	intergenic	HIVID	Yang et al .2018	30271481	Tumor
chr1	249240429	904	PGBD2(dist=27084),NONE(dist=NONE)	intergenic	HIVID	Yang et al .2018	30271481	Tumor
chr10	656499	668	DIP2C	intronic	HIVID	Yang et al .2018	30271481	Tumor
chr10	18604464	1818	CACNB2	intronic	HIVID	Yang et al .2018	30271481	Tumor
chr10	42383355	1168	NONE(dist=NONE),LOC441666(dist=443959)	intergenic	HIVID	Yang et al .2018	30271481	Tumor
chr10	42385975	1776	NONE(dist=NONE),LOC441666(dist=441339)	intergenic	HIVID	Yang et al .2018	30271481	Tumor
chr10	42387308	1825	NONE(dist=NONE),LOC441666(dist=440006)	intergenic	HIVID	Yang et al .2018	30271481	Tumor
chr10	42389867	1789	NONE(dist=NONE),LOC441666(dist=437447)	intergenic	HIVID	Yang et al .2018	30271481	Tumor
chr10	42391353	1825	NONE(dist=NONE),LOC441666(dist=435961)	intergenic	HIVID	Yang et al .2018	30271481	Tumor
chr10	42393895	1158	NONE(dist=NONE),LOC441666(dist=433419)	intergenic	HIVID	Yang et al .2018	30271481	Tumor
chr10	42394180	1590	NONE(dist=NONE),LOC441666(dist=433134)	intergenic	HIVID	Yang et al .2018	30271481	Tumor
chr10	42597051	1776	NONE(dist=NONE),LOC441666(dist=230263)	intergenic	HIVID	Yang et al .2018	30271481	Tumor
chr10	42597619	1158	NONE(dist=NONE),LOC441666(dist=229695)	intergenic	HIVID	Yang et al .2018	30271481	Tumor
chr10	42598234	1825	NONE(dist=NONE),LOC441666(dist=229080)	intergenic	HIVID	Yang et al .2018	30271481	Tumor
chr10	42598660	614	NONE(dist=NONE),LOC441666(dist=228654)	intergenic	HIVID	Yang et al .2018	30271481	Tumor
chr10	42599726	1825	NONE(dist=NONE),LOC441666(dist=227588)	intergenic	HIVID	Yang et al .2018	30271481	Tumor
chr10	66560725	637	REEP3(dist=1175842),ANXA2P3(dist=24560)	intergenic	HIVID	Yang et al .2018	30271481	Tumor
chr10	86201529	1942	FAM190B	intronic	HIVID	Yang et al .2018	30271481	Tumor
chr10	118722542	1942	KIAA1598	intronic	HIVID	Yang et al .2018	30271481	Tumor
chr11	175547	900	LOC100133161(dist=43627),SCGB1C1(dist=17533)	intergenic	HIVID	Yang et al .2018	30271481	Tumor
chr11	39989511	191	NONE(dist=NONE),LRRC4C(dist=146240)	intergenic	HIVID	Yang et al .2018	30271481	Tumor
chr11	134946114	409	LOC283177(dist=570559),NONE(dist=NONE)	intergenic	HIVID	Yang et al .2018	30271481	Tumor
chr11	134946201	409	LOC283177(dist=570646),NONE(dist=NONE)	intergenic	HIVID	Yang et al .2018	30271481	Tumor
chr12	94697	409	LOC100288778(dist=3434),FAM138D(dist=53249)	intergenic	HIVID	Yang et al .2018	30271481	Tumor
chr12	94820	409	LOC100288778(dist=3557),FAM138D(dist=53126)	intergenic	HIVID	Yang et al .2018	30271481	Tumor
chr12	94907	403	LOC100288778(dist=3644),FAM138D(dist=53039)	intergenic	HIVID	Yang et al .2018	30271481	Tumor
chr12	94992	404	LOC100288778(dist=3729),FAM138D(dist=52954)	intergenic	HIVID	Yang et al .2018	30271481	Tumor
chr12	95221	900	LOC100288778(dist=3958),FAM138D(dist=52725)	intergenic	HIVID	Yang et al .2018	30271481	Tumor

Chromosome	Integration site in host genome	Integration site in virus genome	Gene (distance, bp)	Regions	Methods	Author	PMID	Sample
chr12	95342	900	LOC100288778(dist=4079),FAM138D(dist=52604)	intergenic	HIVID	Yang et al .2018	30271481	Tumor
chr12	95424	900	LOC100288778(dist=4161),FAM138D(dist=52522)	intergenic	HIVID	Yang et al .2018	30271481	Tumor
chr12	95545	904	LOC100288778(dist=4282),FAM138D(dist=52401)	intergenic	HIVID	Yang et al .2018	30271481	Tumor
chr12	217255	904	IQSEC3	intronic	HIVID	Yang et al .2018	30271481	Tumor
chr12	61999640	518	SLC16A7(dist=1816005),FAM19A2(dist=102389)	intergenic	HIVID	Yang et al .2018	30271481	Tumor
chr12	66451372	2703	HMGA2(dist=91301),LLPH(dist=65477)	intergenic	HIVID	Yang et al .2018	30271481	Tumor
chr13	21727908	3162	SKA3	UTR3	HIVID	Yang et al .2018	30271481	Tumor
chr13	40370513	1942	COG6(dist=4711),LINC00332(dist=385433)	intergenic	HIVID	Yang et al .2018	30271481	Tumor
chr14	31356637	1818	LOC100506071	ncRNA_intronic	HIVID	Yang et al .2018	30271481	Tumor
chr15	102520332	409	DDX11L9(dist=1036),NONE(dist=NONE)	intergenic	HIVID	Yang et al .2018	30271481	Tumor
chr15	102520405	409	DDX11L9(dist=1109),NONE(dist=NONE)	intergenic	HIVID	Yang et al .2018	30271481	Tumor
chr15	102521282	904	DDX11L9(dist=1986),NONE(dist=NONE)	intergenic	HIVID	Yang et al .2018	30271481	Tumor
chr16	60573	403	DDX11L10	upstream	HIVID	Yang et al .2018	30271481	Tumor
chr16	24701311	1942	RBBP6(dist=117128),TNRC6A(dist=39738)	intergenic	HIVID	Yang et al .2018	30271481	Tumor
chr17	20606088	1079	CDRT15L2(dist=121864),CCDC144NL(dist=160620)	intergenic	HIVID	Yang et al .2018	30271481	Tumor
chr17	81195157	900	FLJ43681(dist=6584),NONE(dist=NONE)	intergenic	HIVID	Yang et al .2018	30271481	Tumor
chr18	10094	900	NONE(dist=NONE),ROCK1P1(dist=98971)	intergenic	HIVID	Yang et al .2018	30271481	Tumor
chr18	10169	900	NONE(dist=NONE),ROCK1P1(dist=98896)	intergenic	HIVID	Yang et al .2018	30271481	Tumor
chr18	10241	900	NONE(dist=NONE),ROCK1P1(dist=98824)	intergenic	HIVID	Yang et al .2018	30271481	Tumor
chr18	10334	900	NONE(dist=NONE),ROCK1P1(dist=98731)	intergenic	HIVID	Yang et al .2018	30271481	Tumor
chr18	10461	900	NONE(dist=NONE),ROCK1P1(dist=98604)	intergenic	HIVID	Yang et al .2018	30271481	Tumor
chr18	63740	900	NONE(dist=NONE),ROCK1P1(dist=45325)	intergenic	HIVID	Yang et al .2018	30271481	Tumor
chr18	27253791	2430	CDH2(dist=1496346),MIR302F(dist=625085)	intergenic	HIVID	Yang et al .2018	30271481	Tumor
chr19	46866565	1942	PPP5C	intronic	HIVID	Yang et al .2018	30271481	Tumor
chr2	35349236	1942	MYADML(dist=1395952),LOC100288911(dist=1232656)	intergenic	HIVID	Yang et al .2018	30271481	Tumor
chr2	67411412	1818	LOC644838	ncRNA_intronic	HIVID	Yang et al .2018	30271481	Tumor
chr2	83878716	862	LOC1720(dist=793823),FUNDC2P2(dist=639090)	intergenic	HIVID	Yang et al .2018	30271481	Tumor
chr2	83878722	1820	LOC1720(dist=793829),FUNDC2P2(dist=639084)	intergenic	HIVID	Yang et al .2018	30271481	Tumor
chr2	83878743	2332	LOC1720(dist=793850),FUNDC2P2(dist=639063)	intergenic	HIVID	Yang et al .2018	30271481	Tumor
chr2	88321181	1942	RGPD1(dist=35872),KRCC1(dist=5543)	intergenic	HIVID	Yang et al .2018	30271481	Tumor
chr2	89876037	2937	MIR4436A(dist=764069),LOC654342(dist=1948672)	intergenic	HIVID	Yang et al .2018	30271481	Tumor
chr2	114359761	409	DDX11L2	ncRNA_intronic	HIVID	Yang et al .2018	30271481	Tumor
chr2	117230894	1307	DPP10(dist=628568),DDX18(dist=1341361)	intergenic	HIVID	Yang et al .2018	30271481	Tumor
chr2	126513262	614	CNTNAP5(dist=840399),GYPC(dist=900249)	intergenic	HIVID	Yang et al .2018	30271481	Tumor
chr2	183926806	1942	NCKAP1(dist=23577),DUSP19(dist=16481)	intergenic	HIVID	Yang et al .2018	30271481	Tumor
chr2	187922140	1942	ZSWIM2(dist=208243),CALCRL(dist=285709)	intergenic	HIVID	Yang et al .2018	30271481	Tumor
chr2	194773324	1942	PCGEM1(dist=1131699),SLC39A10(dist=1748208)	intergenic	HIVID	Yang et al .2018	30271481	Tumor
chr2	202594636	1817	ALS2	intronic	HIVID	Yang et al .2018	30271481	Tumor
chr2	218080352	1942	TNP1(dist=355570),DIRC3(dist=68394)	intergenic	HIVID	Yang et al .2018	30271481	Tumor
chr2	243152516	900	LOC728323(dist=50047),NONE(dist=NONE)	intergenic	HIVID	Yang et al .2018	30271481	Tumor
chr20	8986283	1942	PLCB1(dist=120736),PLCB4(dist=63418)	intergenic	HIVID	Yang et al .2018	30271481	Tumor
chr20	37574873	1821	FAM83D	intronic	HIVID	Yang et al .2018	30271481	Tumor
chr20	62918182	900	PCMTD2(dist=10603),LINC00266-1(dist=3556)	intergenic	HIVID	Yang et al .2018	30271481	Tumor
chr20	62918533	900	PCMTD2(dist=10954),LINC00266-1(dist=3205)	intergenic	HIVID	Yang et al .2018	30271481	Tumor
chr21	9449020	1942	NONE(dist=NONE),MIR3648(dist=376812)	intergenic	HIVID	Yang et al .2018	30271481	Tumor
chr21	35074392	1942	ITSN1	intronic	HIVID	Yang et al .2018	30271481	Tumor
chr21	48119832	900	PRMT2(dist=34677),NONE(dist=NONE)	intergenic	HIVID	Yang et al .2018	30271481	Tumor
chr22	30395998	1742	MTMR3	intronic	HIVID	Yang et al .2018	30271481	Tumor
chr22	43261815	900	ARFGAP3(dist=8407),PACSIN2(dist=3957)	intergenic	HIVID	Yang et al .2018	30271481	Tumor
chr3	9496344	1821	SETD5	intronic	HIVID	Yang et al .2018	30271481	Tumor
chr3	31532885	1942	GADL1(dist=596732),STT3B(dist=41606)	intergenic	HIVID	Yang et al .2018	30271481	Tumor
chr3	71005879	1942	FOXP1	UTR3	HIVID	Yang et al .2018	30271481	Tumor
chr3	79824684	3131	ROBO1(dist=7625),GBE1(dist=1714166)	intergenic	HIVID	Yang et al .2018	30271481	Tumor
chr3	98412795	2834	CPOX(dist=100340),ST3GAL6-AS1(dist=20382)	intergenic	HIVID	Yang et al .2018	30271481	Tumor
chr3	99194829	395	DCBLD2(dist=574296),MIR548G(dist=78324)	intergenic	HIVID	Yang et al .2018	30271481	Tumor
chr3	120574945	1818	GTF2E1(dist=73029),STXBP5L(dist=52105)	intergenic	HIVID	Yang et al .2018	30271481	Tumor
chr3	182030309	1817	SOX2-OT(dist=571304),FLJ46066(dist=134449)	intergenic	HIVID	Yang et al .2018	30271481	Tumor
chr3	195283715	1942	PPP1R2(dist=13491),APOD(dist=11858)	intergenic	HIVID	Yang et al .2018	30271481	Tumor
chr3	196625694	1825	SENP5	intronic	HIVID	Yang et al .2018	30271481	Tumor
chr3	197900324	900	FAM157A	intronic	HIVID	Yang et al .2018	30271481	Tumor
chr3	197900912	900	FAM157A	intronic	HIVID	Yang et al .2018	30271481	Tumor
chr4	10114	900	NONE(dist=NONE),ZNF595(dist=43113)	intergenic	HIVID	Yang et al .2018	30271481	Tumor
chr4	23372657	1635	MIR548AJ2	ncRNA_intronic	HIVID	Yang et al .2018	30271481	Tumor
chr4	49129026	3006	CWH43(dist=64931),NONE(dist=NONE)	intergenic	HIVID	Yang et al .2018	30271481	Tumor
chr4	49136853	3006	CWH43(dist=72758),NONE(dist=NONE)	intergenic	HIVID	Yang et al .2018	30271481	Tumor
chr4	49147301	2937	CWH43(dist=83206),NONE(dist=NONE)	intergenic	HIVID	Yang et al .2018	30271481	Tumor
chr4	49151449	2876	CWH43(dist=87354),NONE(dist=NONE)	intergenic	HIVID	Yang et al .2018	30271481	Tumor
chr4	49514557	1540	CWH43(dist=450462),NONE(dist=NONE)	intergenic	HIVID	Yang et al .2018	30271481	Tumor
chr4	49650785	2876	CWH43(dist=586690),NONE(dist=NONE)	intergenic	HIVID	Yang et al .2018	30271481	Tumor
chr4	95039765	616	ATOH1(dist=288623),SMARCAD1(dist=88994)	intergenic	HIVID	Yang et al .2018	30271481	Tumor
chr4	95970566	1938	BMPR1B	intronic	HIVID	Yang et al .2018	30271481	Tumor
chr4	122434706	804	QRFPR(dist=132525),ANXA5(dist=154446)	intergenic	HIVID	Yang et al .2018	30271481	Tumor
chr4	140630218	1821	MGST2	intronic	HIVID	Yang et al .2018	30271481	Tumor
chr4	161348938	1157	RAPGEF2(dist=1067637),FSTL5(dist=956106)	intergenic	HIVID	Yang et al .2018	30271481	Tumor
chr4	191043758	904	DUX4L2(dist=30282),NONE(dist=NONE)	intergenic	HIVID	Yang et al .2018	30271481	Tumor
chr4	191043856	900	DUX4L2(dist=30380),NONE(dist=NONE)	intergenic	HIVID	Yang et al .2018	30271481	Tumor
chr4	191044118	900	DUX4L2(dist=30642),NONE(dist=NONE)	intergenic	HIVID	Yang et al .2018	30271481	Tumor
chr5	10525	900	NONE(dist=NONE),PLEKHG4B(dist=129848)	intergenic	HIVID	Yang et al .2018	30271481	Tumor
chr5	11353	900	NONE(dist=NONE),PLEKHG4B(dist=129020)	intergenic	HIVID	Yang et al .2018	30271481	Tumor
chr5	11732	900	NONE(dist=NONE),PLEKHG4B(dist=128641)	intergenic	HIVID	Yang et al .2018	30271481	Tumor
chr5	12464	403	NONE(dist=NONE),PLEKHG4B(dist=127909)	intergenic	HIVID	Yang et al .2018	30271481	Tumor
chr5	12542	409	NONE(dist=NONE),PLEKHG4B(dist=127831)	intergenic	HIVID	Yang et al .2018	30271481	Tumor
chr5	12629	403	NONE(dist=NONE),PLEKHG4B(dist=127744)	intergenic	HIVID	Yang et al .2018	30271481	Tumor
chr5	12716	409	NONE(dist=NONE),PLEKHG4B(dist=127657)	intergenic	HIVID	Yang et al .2018	30271481	Tumor
chr5	12803	409	NONE(dist=NONE),PLEKHG4B(dist=127570)	intergenic	HIVID	Yang et al .2018	30271481	Tumor
chr5	1301025	2109	TERT(dist=5863),MIR4457(dist=8400)	intergenic	HIVID	Yang et al .2018	30271481	Tumor
chr5	91334435	1416	ARRDC3-AS1(dist=617903),FLJ42709(dist=1410630)	intergenic	HIVID	Yang et al .2018	30271481	Tumor
chr5	132441789	1942	HSPA4(dist=1080),FSTL4(dist=90363)	intergenic	HIVID	Yang et al .2018	30271481	Tumor
chr5	136678646	2037	SPOCK1	intronic	HIVID	Yang et al .2018	30271481	Tumor
chr6	741275	2018	EXOC2(dist=48134),LOC285768(dist=219966)	intergenic	HIVID	Yang et al .2018	30271481	Tumor
chr6	741410	1441	EXOC2(dist=48269),LOC285768(dist=219831)	intergenic	HIVID	Yang et al .2018	30271481	Tumor
chr6	115687594	1019	HS3ST5(dist=1303553),FRK(dist=575099)	intergenic	HIVID	Yang et al .2018	30271481	Tumor
chr6	120716912	1457	LOC285762(dist=904445),C6orf170(dist=683715)	intergenic	HIVID	Yang et al .2018	30271481	Tumor
chr7	2056887	709	MAD1L1	intronic	HIVID	Yang et al .2018	30271481	Tumor

Supplementary Table S8 Continued

Chromosome	Integration site in host genome	Integration site in virus genome	Gene (distance, bp)	Regions	Methods	Author	PMID	Sample
chr7	102946298	2575	PMPCB	intronic	HIVID	Yang et al .2018	30271481	Tumor
chr7	153973727	2565	DPP6	intronic	HIVID	Yang et al .2018	30271481	Tumor
chr8	25640950	2750	CDCA2(dist=275525),EBF2(dist=58296)	intergenic	HIVID	Yang et al .2018	30271481	Tumor
chr8	42823576	1270	HOOK3	intronic	HIVID	Yang et al .2018	30271481	Tumor
chr8	43822084	542	POTEA(dist=603756),NONE(dist=NONE)	intergenic	HIVID	Yang et al .2018	30271481	Tumor
chr8	43823735	542	POTEA(dist=605407),NONE(dist=NONE)	intergenic	HIVID	Yang et al .2018	30271481	Tumor
chr8	43825607	542	POTEA(dist=607279),NONE(dist=NONE)	intergenic	HIVID	Yang et al .2018	30271481	Tumor
chr8	43829343	542	POTEA(dist=611015),NONE(dist=NONE)	intergenic	HIVID	Yang et al .2018	30271481	Tumor
chr8	43831210	539	POTEA(dist=612882),NONE(dist=NONE)	intergenic	HIVID	Yang et al .2018	30271481	Tumor
chr8	43834972	390	POTEA(dist=616644),NONE(dist=NONE)	intergenic	HIVID	Yang et al .2018	30271481	Tumor
chr8	43838683	481	POTEA(dist=620355),NONE(dist=NONE)	intergenic	HIVID	Yang et al .2018	30271481	Tumor
chr8	46839685	542	NONE(dist=NONE),LINC00293(dist=912823)	intergenic	HIVID	Yang et al .2018	30271481	Tumor
chr8	46839764	40	NONE(dist=NONE),LINC00293(dist=912744)	intergenic	HIVID	Yang et al .2018	30271481	Tumor
chr8	46841552	541	NONE(dist=NONE),LINC00293(dist=910956)	intergenic	HIVID	Yang et al .2018	30271481	Tumor
chr8	46843637	542	NONE(dist=NONE),LINC00293(dist=908871)	intergenic	HIVID	Yang et al .2018	30271481	Tumor
chr8	46845508	542	NONE(dist=NONE),LINC00293(dist=907000)	intergenic	HIVID	Yang et al .2018	30271481	Tumor
chr8	46846820	542	NONE(dist=NONE),LINC00293(dist=905688)	intergenic	HIVID	Yang et al .2018	30271481	Tumor
chr8	46847375	542	NONE(dist=NONE),LINC00293(dist=905133)	intergenic	HIVID	Yang et al .2018	30271481	Tumor
chr8	46849026	541	NONE(dist=NONE),LINC00293(dist=903482)	intergenic	HIVID	Yang et al .2018	30271481	Tumor
chr8	46852928	508	NONE(dist=NONE),LINC00293(dist=899580)	intergenic	HIVID	Yang et al .2018	30271481	Tumor
chr8	46854850	542	NONE(dist=NONE),LINC00293(dist=897658)	intergenic	HIVID	Yang et al .2018	30271481	Tumor
chr8	52648078	1942	PXDNL	intronic	HIVID	Yang et al .2018	30271481	Tumor
chr8	76765715	3096	HNF4G(dist=286654),ZFHX4-AS1(dist=757399)	intergenic	HIVID	Yang et al .2018	30271481	Tumor
chr8	83827169	1942	SNX16(dist=1072648),RALYL(dist=1268284)	intergenic	HIVID	Yang et al .2018	30271481	Tumor
chr8	95583885	1078	KIAA1429(dist=18139),LOC100288748(dist=65628)	intergenic	HIVID	Yang et al .2018	30271481	Tumor
chr8	95600675	468	KIAA1429(dist=34929),LOC100288748(dist=48838)	intergenic	HIVID	Yang et al .2018	30271481	Tumor
chr8	124342024	1821	ATAD2	intronic	HIVID	Yang et al .2018	30271481	Tumor
chr8	131924255	1828	ADCY8	intronic	HIVID	Yang et al .2018	30271481	Tumor
chr8	137492720	1754	KHDRBS3(dist=832872),FAM135B(dist=1649546)	intergenic	HIVID	Yang et al .2018	30271481	Tumor
chr9	10988	409	DDX11L5	upstream	HIVID	Yang et al .2018	30271481	Tumor
chr9	36621271	1942	MELK	intronic	HIVID	Yang et al .2018	30271481	Tumor
chr9	75147462	1942	TMC1	intronic	HIVID	Yang et al .2018	30271481	Tumor
chr9	81498453	1821	PSAT1(dist=553444),TLE4(dist=688425)	intergenic	HIVID	Yang et al .2018	30271481	Tumor
chr9	139818095	1818	TRAF2	intronic	HIVID	Yang et al .2018	30271481	Tumor
chrX	91142314	1752	PCDH11X	intronic	HIVID	Yang et al .2018	30271481	Tumor
chrX	98858836	1817	LOC442459	ncRNA_intronic	HIVID	Yang et al .2018	30271481	Tumor
chrX	155258812	409	IL9R(dist=18330),NONE(dist=NONE)	intergenic	HIVID	Yang et al .2018	30271481	Tumor
chrX	155258894	409	IL9R(dist=18412),NONE(dist=NONE)	intergenic	HIVID	Yang et al .2018	30271481	Tumor
chrX	155258986	409	IL9R(dist=18504),NONE(dist=NONE)	intergenic	HIVID	Yang et al .2018	30271481	Tumor
chrX	155260172	900	IL9R(dist=19690),NONE(dist=NONE)	intergenic	HIVID	Yang et al .2018	30271481	Tumor
chrY	9994	904	NONE(dist=NONE),PLCXD1(dist=138067)	intergenic	HIVID	Yang et al .2018	30271481	Tumor
chrY	4977137	1752	PCDH11Y	intronic	HIVID	Yang et al .2018	30271481	Tumor
chrY	58831985	1727	NONE(dist=NONE),SPRY3(dist=268472)	intergenic	HIVID	Yang et al .2018	30271481	Tumor
chrY	58838997	1727	NONE(dist=NONE),SPRY3(dist=261460)	intergenic	HIVID	Yang et al .2018	30271481	Tumor
chrY	58853324	1727	NONE(dist=NONE),SPRY3(dist=247133)	intergenic	HIVID	Yang et al .2018	30271481	Tumor
chrY	58878325	1727	NONE(dist=NONE),SPRY3(dist=222132)	intergenic	HIVID	Yang et al .2018	30271481	Tumor
chrY	58885403	1727	NONE(dist=NONE),SPRY3(dist=215054)	intergenic	HIVID	Yang et al .2018	30271481	Tumor
chrY	58888988	1727	NONE(dist=NONE),SPRY3(dist=211469)	intergenic	HIVID	Yang et al .2018	30271481	Tumor
chrY	58892573	1727	NONE(dist=NONE),SPRY3(dist=207884)	intergenic	HIVID	Yang et al .2018	30271481	Tumor
chrY	58899709	1727	NONE(dist=NONE),SPRY3(dist=200748)	intergenic	HIVID	Yang et al .2018	30271481	Tumor
chrY	58906846	1727	NONE(dist=NONE),SPRY3(dist=193611)	intergenic	HIVID	Yang et al .2018	30271481	Tumor
chrY	58910430	1727	NONE(dist=NONE),SPRY3(dist=190027)	intergenic	HIVID	Yang et al .2018	30271481	Tumor
chrY	58915983	1727	NONE(dist=NONE),SPRY3(dist=184474)	intergenic	HIVID	Yang et al .2018	30271481	Tumor
chrY	59361818	409	IL9R(dist=18330),NONE(dist=NONE)	intergenic	HIVID	Yang et al .2018	30271481	Tumor
chrY	59361903	404	IL9R(dist=18415),NONE(dist=NONE)	intergenic	HIVID	Yang et al .2018	30271481	Tumor
chrY	59361987	409	IL9R(dist=18499),NONE(dist=NONE)	intergenic	HIVID	Yang et al .2018	30271481	Tumor
chr1	33423267	521	RNF19B	intronic	HIVID	Yang et al .2018	30271481	Tumor
chr1	62757364	1798	KANK4	intronic	HIVID	Yang et al .2018	30271481	Tumor
chr1	142541422	1442	NONE(dist=NONE),ANKRD20A12P(dist=155999)	intergenic	HIVID	Yang et al .2018	30271481	Tumor
chr1	175801905	1789	TNR(dist=89153),RFWD2(dist=112062)	intergenic	HIVID	Yang et al .2018	30271481	Tumor
chr1	196155653	1793	NONE(dist=NONE),KCNT2(dist=39260)	intergenic	HIVID	Yang et al .2018	30271481	Tumor
chr1	208136119	217	CD34(dist=51436),PLXNA2(dist=59469)	intergenic	HIVID	Yang et al .2018	30271481	Tumor
chr10	42383563	2992	NONE(dist=NONE),LOC441666(dist=443751)	intergenic	HIVID	Yang et al .2018	30271481	Tumor
chr10	42385400	2987	NONE(dist=NONE),LOC441666(dist=441914)	intergenic	HIVID	Yang et al .2018	30271481	Tumor
chr10	42386542	2992	NONE(dist=NONE),LOC441666(dist=440772)	intergenic	HIVID	Yang et al .2018	30271481	Tumor
chr10	42386954	2989	NONE(dist=NONE),LOC441666(dist=440360)	intergenic	HIVID	Yang et al .2018	30271481	Tumor
chr10	42387715	2695	NONE(dist=NONE),LOC441666(dist=439599)	intergenic	HIVID	Yang et al .2018	30271481	Tumor
chr10	42389171	2962	NONE(dist=NONE),LOC441666(dist=438143)	intergenic	HIVID	Yang et al .2018	30271481	Tumor
chr10	42391039	2825	NONE(dist=NONE),LOC441666(dist=436275)	intergenic	HIVID	Yang et al .2018	30271481	Tumor
chr10	42391717	2695	NONE(dist=NONE),LOC441666(dist=435597)	intergenic	HIVID	Yang et al .2018	30271481	Tumor
chr10	42393276	2866	NONE(dist=NONE),LOC441666(dist=434038)	intergenic	HIVID	Yang et al .2018	30271481	Tumor
chr10	42396045	2992	NONE(dist=NONE),LOC441666(dist=431269)	intergenic	HIVID	Yang et al .2018	30271481	Tumor
chr10	42596972	2866	NONE(dist=NONE),LOC441666(dist=230342)	intergenic	HIVID	Yang et al .2018	30271481	Tumor
chr10	42598111	2768	NONE(dist=NONE),LOC441666(dist=229203)	intergenic	HIVID	Yang et al .2018	30271481	Tumor
chr10	42599615	290	NONE(dist=NONE),LOC441666(dist=227699)	intergenic	HIVID	Yang et al .2018	30271481	Tumor
chr10	42599866	2866	NONE(dist=NONE),LOC441666(dist=227448)	intergenic	HIVID	Yang et al .2018	30271481	Tumor
chr11	103357256	2363	DYNC2H1(dist=6665),MIR4693(dist=363378)	intergenic	HIVID	Yang et al .2018	30271481	Tumor
chr11	111879121	299	DIXDC1	intronic	HIVID	Yang et al .2018	30271481	Tumor
chr12	57171099	1009	HSD17B6	intronic	HIVID	Yang et al .2018	30271481	Tumor
chr12	66451372	2960	HMGA2(dist=91301),LLPH(dist=65477)	intergenic	HIVID	Yang et al .2018	30271481	Tumor
chr12	66451373	14	HMGA2(dist=91302),LLPH(dist=65476)	intergenic	HIVID	Yang et al .2018	30271481	Tumor
chr12	66451373	2960	HMGA2(dist=91302),LLPH(dist=65476)	intergenic	HIVID	Yang et al .2018	30271481	Tumor
chr13	104623487	2949	MIR548AS(dist=688639),DAOA-AS1(dist=1487919)	intergenic	HIVID	Yang et al .2018	30271481	Tumor
chr14	30955268	2417	PRKD1(dist=558369),G2E3(dist=73061)	intergenic	HIVID	Yang et al .2018	30271481	Tumor
chr14	78923748	2136	NRXN3	intronic	HIVID	Yang et al .2018	30271481	Tumor
chr16	33866141	1440	RNU6-76(dist=302898),LINC00273(dist=94911)	intergenic	HIVID	Yang et al .2018	30271481	Tumor
chr16	33869928	1442	RNU6-76(dist=306685),LINC00273(dist=91124)	intergenic	HIVID	Yang et al .2018	30271481	Tumor
chr16	33887669	1442	RNU6-76(dist=324426),LINC00273(dist=73383)	intergenic	HIVID	Yang et al .2018	30271481	Tumor
chr16	33899140	1436	RNU6-76(dist=335897),LINC00273(dist=61912)	intergenic	HIVID	Yang et al .2018	30271481	Tumor
chr16	33901650	1442	RNU6-76(dist=338407),LINC00273(dist=59402)	intergenic	HIVID	Yang et al .2018	30271481	Tumor
chr16	33902268	1387	RNU6-76(dist=339025),LINC00273(dist=58784)	intergenic	HIVID	Yang et al .2018	30271481	Tumor
chr16	70290927	1789	AARS	intronic	HIVID	Yang et al .2018	30271481	Tumor
chr17	10252503	1795	MYH13	intronic	HIVID	Yang et al .2018	30271481	Tumor
chr17	19662280	1798	ALDH3A1(dist=10534),ULK2(dist=11863)	intergenic	HIVID	Yang et al .2018	30271481	Tumor

Chromosome	Integration site in host genome	Integration site in virus genome	Gene (distance, bp)	Regions	Methods	Author	PMID	Sample
chr17	25263276	1337	NONE(dist=NONE),MIR4522(dist=357660)	intergenic	HIVID	Yang et al .2018	30271481	Tumor
chr17	25267037	1133	NONE(dist=NONE),MIR4522(dist=353899)	intergenic	HIVID	Yang et al .2018	30271481	Tumor
chr17	25267847	1442	NONE(dist=NONE),MIR4522(dist=353089)	intergenic	HIVID	Yang et al .2018	30271481	Tumor
chr17	55278554	439	AKAP1(dist=79844),MSI2(dist=55377)	intergenic	HIVID	Yang et al .2018	30271481	Tumor
chr18	54624481	3115	WDR7	intronic	HIVID	Yang et al .2018	30271481	Tumor
chr19	48583918	1051	PLA2G4C	intronic	HIVID	Yang et al .2018	30271481	Tumor
chr2	10580055	1223	ODC1	downstream	HIVID	Yang et al .2018	30271481	Tumor
chr2	216852202	1787	MREG	intronic	HIVID	Yang et al .2018	30271481	Tumor
chr20	17669342	870	RRBP1(dist=6414),BANF2(dist=4978)	intergenic	HIVID	Yang et al .2018	30271481	Tumor
chr20	22186187	2860	LOC100270679(dist=130895),LOC284788(dist=194784)	intergenic	HIVID	Yang et al .2018	30271481	Tumor
chr21	10086270	1442	TEKT4P2(dist=117677),TPTE(dist=820473)	intergenic	HIVID	Yang et al .2018	30271481	Tumor
chr21	10780414	1248	TEKT4P2(dist=811821),TPTE(dist=126329)	intergenic	HIVID	Yang et al .2018	30271481	Tumor
chr21	10781804	1443	TEKT4P2(dist=813211),TPTE(dist=124939)	intergenic	HIVID	Yang et al .2018	30271481	Tumor
chr21	10788024	1375	TEKT4P2(dist=819431),TPTE(dist=118719)	intergenic	HIVID	Yang et al .2018	30271481	Tumor
chr21	10799030	1442	TEKT4P2(dist=830437),TPTE(dist=107713)	intergenic	HIVID	Yang et al .2018	30271481	Tumor
chr21	10801112	1387	TEKT4P2(dist=832519),TPTE(dist=105631)	intergenic	HIVID	Yang et al .2018	30271481	Tumor
chr21	31005264	234	GRIK1	intronic	HIVID	Yang et al .2018	30271481	Tumor
chr22	22824389	3036	LOC96610(dist=147065),ZNF280B(dist=14383)	intergenic	HIVID	Yang et al .2018	30271481	Tumor
chr22	22824422	1824	LOC96610(dist=147098),ZNF280B(dist=14350)	intergenic	HIVID	Yang et al .2018	30271481	Tumor
chr4	49110242	1248	CWH43(dist=46147),NONE(dist=NONE)	intergenic	HIVID	Yang et al .2018	30271481	Tumor
chr4	65117979	2624	NONE(dist=NONE),TECRL(dist=26198)	intergenic	HIVID	Yang et al .2018	30271481	Tumor
chr4	133835690	1182	NONE(dist=NONE),PCDH10(dist=234780)	intergenic	HIVID	Yang et al .2018	30271481	Tumor
chr5	318225	532	AHRR	intronic	HIVID	Yang et al .2018	30271481	Tumor
chr5	318336	532	AHRR	intronic	HIVID	Yang et al .2018	30271481	Tumor
chr5	11414125	1050	CTNND2	intronic	HIVID	Yang et al .2018	30271481	Tumor
chr5	140318779	104	PCDHA1,PCDHA10,PCDHA11,PCDHA12,PCDHA13,PCDHA2,P	intronic	HIVID	Yang et al .2018	30271481	Tumor
chr6	741459	1441	EXOC2(dist=48318),LOC285768(dist=219782)	intergenic	HIVID	Yang et al .2018	30271481	Tumor
chr6	166244656	1789	PDE10A(dist=169068),LINC00473(dist=92880)	intergenic	HIVID	Yang et al .2018	30271481	Tumor
chr7	114228831	3154	FOXP2	intronic	HIVID	Yang et al .2018	30271481	Tumor
chr8	93425174	104	RUNX1T1(dist=309720),FLJ46284(dist=300016)	intergenic	HIVID	Yang et al .2018	30271481	Tumor
chr9	100997271	1789	TBC1D2	intronic	HIVID	Yang et al .2018	30271481	Tumor
chr9	126229718	1789	DENND1A	intronic	HIVID	Yang et al .2018	30271481	Tumor
chrY	13140133	1440	NONE(dist=NONE),GYG2P1(dist=1377782)	intergenic	HIVID	Yang et al .2018	30271481	Tumor
chrY	13446517	1442	NONE(dist=NONE),GYG2P1(dist=1071398)	intergenic	HIVID	Yang et al .2018	30271481	Tumor
chrY	13448803	1337	NONE(dist=NONE),GYG2P1(dist=1069112)	intergenic	HIVID	Yang et al .2018	30271481	Tumor
chrY	13832521	1442	NONE(dist=NONE),GYG2P1(dist=685394)	intergenic	HIVID	Yang et al .2018	30271481	Tumor
chrY	28784360	1226	TTTY3B(dist=904825),NONE(dist=NONE)	intergenic	HIVID	Yang et al .2018	30271481	Tumor
chrY	58979366	1226	NONE(dist=NONE),SPRY3(dist=121091)	intergenic	HIVID	Yang et al .2018	30271481	Tumor
chrY	58980674	1248	NONE(dist=NONE),SPRY3(dist=119783)	intergenic	HIVID	Yang et al .2018	30271481	Tumor
chr1	10000	2744	NONE(dist=NONE),DDX11L1(dist=1874)	intergenic	HIVID	Yang et al .2018	30271481	Tumor
chr1	21116030	2275	HP1BP3(dist=2849),EIF4G3(dist=16755)	intergenic	HIVID	Yang et al .2018	30271481	Tumor
chr1	69200831	2373	DEPDC1(dist=238032),LRRC7(dist=1025027)	intergenic	HIVID	Yang et al .2018	30271481	Tumor
chr1	86219069	1958	COL24A1	intronic	HIVID	Yang et al .2018	30271481	Tumor
chr1	87145954	2081	CLCA3P(dist=24895),SH3GLB1(dist=24299)	intergenic	HIVID	Yang et al .2018	30271481	Tumor
chr1	91681340	1958	ZNF644(dist=193528),HFM1(dist=44983)	intergenic	HIVID	Yang et al .2018	30271481	Tumor
chr1	104578385	2081	AMY1A(dist=277074),LOC100129138(dist=37260)	intergenic	HIVID	Yang et al .2018	30271481	Tumor
chr1	121275097	2018	EMBP1	ncRNA_intronic	HIVID	Yang et al .2018	30271481	Tumor
chr1	144039515	2018	SRGAP2B	ncRNA_intronic	HIVID	Yang et al .2018	30271481	Tumor
chr1	149051123	1130	LOC645166(dist=98069),LOC388692(dist=228353)	intergenic	HIVID	Yang et al .2018	30271481	Tumor
chr1	149053754	1959	LOC645166(dist=100700),LOC388692(dist=225722)	intergenic	HIVID	Yang et al .2018	30271481	Tumor
chr1	150331854	2326	PRPF3(dist=6150),RPRD2(dist=5136)	intergenic	HIVID	Yang et al .2018	30271481	Tumor
chr1	171440457	2275	FMO4(dist=129234),PRRC2C(dist=14209)	intergenic	HIVID	Yang et al .2018	30271481	Tumor
chr1	174203468	1958	RABGAP1L	intronic	HIVID	Yang et al .2018	30271481	Tumor
chr1	179821843	476	TOR1AIP2	UTR5	HIVID	Yang et al .2018	30271481	Tumor
chr1	179822409	222	TOR1AIP2	intronic	HIVID	Yang et al .2018	30271481	Tumor
chr1	179824548	1952	TOR1AIP2	intronic	HIVID	Yang et al .2018	30271481	Tumor
chr1	180669415	1958	XPR1	intronic	HIVID	Yang et al .2018	30271481	Tumor
chr1	187313962	1958	PLA2G4A(dist=355849),NONE(dist=NONE)	intergenic	HIVID	Yang et al .2018	30271481	Tumor
chr1	197681762	2014	DENND1B	intronic	HIVID	Yang et al .2018	30271481	Tumor
chr1	218183631	2018	LINC00210(dist=89485),RRP15(dist=274998)	intergenic	HIVID	Yang et al .2018	30271481	Tumor
chr1	224640012	2326	WDR26(dist=18011),CNIH3(dist=164167)	intergenic	HIVID	Yang et al .2018	30271481	Tumor
chr10	13259658	2373	MCM10(dist=6554),UCMA(dist=4109)	intergenic	HIVID	Yang et al .2018	30271481	Tumor
chr10	21307815	2326	NEBL	intronic	HIVID	Yang et al .2018	30271481	Tumor
chr10	33800839	1958	NRP1(dist=177006),LOC100505583(dist=247802)	intergenic	HIVID	Yang et al .2018	30271481	Tumor
chr10	42387032	1848	NONE(dist=NONE),LOC441666(dist=440282)	intergenic	HIVID	Yang et al .2018	30271481	Tumor
chr10	91666538	1959	LOC643529(dist=65920),HTR7(dist=834038)	intergenic	HIVID	Yang et al .2018	30271481	Tumor
chr10	97708263	1958	LOC728558	ncRNA_intronic	HIVID	Yang et al .2018	30271481	Tumor
chr10	103486455	2326	FBXW4(dist=31712),FGF8(dist=43432)	intergenic	HIVID	Yang et al .2018	30271481	Tumor
chr10	113348542	1958	ADRA2A(dist=507880),GPAM(dist=561080)	intergenic	HIVID	Yang et al .2018	30271481	Tumor
chr10	119593461	1958	EMX2(dist=284404),RAB11FIP2(dist=170966)	intergenic	HIVID	Yang et al .2018	30271481	Tumor
chr10	123645273	2260	ATE1	intronic	HIVID	Yang et al .2018	30271481	Tumor
chr10	133537343	1958	TCERG1L(dist=427359),FLJ46300(dist=67391)	intergenic	HIVID	Yang et al .2018	30271481	Tumor
chr10	135524590	2970	DUX2(dist=26098),NONE(dist=NONE)	intergenic	HIVID	Yang et al .2018	30271481	Tumor
chr11	23337901	2055	LOC100500938(dist=455929),LUZP2(dist=1180615)	intergenic	HIVID	Yang et al .2018	30271481	Tumor
chr11	29176643	2018	METTL15(dist=821589),KCNA4(dist=854645)	intergenic	HIVID	Yang et al .2018	30271481	Tumor
chr11	31338297	1959	DCDC1	intronic	HIVID	Yang et al .2018	30271481	Tumor
chr11	43117577	2014	LOC100507205(dist=842337),HNRNPKP3(dist=165477)	intergenic	HIVID	Yang et al .2018	30271481	Tumor
chr11	54966696	1959	NONE(dist=NONE),TRIM48(dist=62962)	intergenic	HIVID	Yang et al .2018	30271481	Tumor
chr11	85040769	2018	DLG2	intronic	HIVID	Yang et al .2018	30271481	Tumor
chr11	87052138	2018	TMEM135(dist=12262),RAB38(dist=794277)	intergenic	HIVID	Yang et al .2018	30271481	Tumor
chr11	93774890	1958	HEPHL1	intronic	HIVID	Yang et al .2018	30271481	Tumor
chr11	104547187	1992	PDGFD(dist=512160),CASP12(dist=209258)	intergenic	HIVID	Yang et al .2018	30271481	Tumor
chr11	130812377	2081	SNX19(dist=25995),NTM(dist=427994)	intergenic	HIVID	Yang et al .2018	30271481	Tumor
chr12	95505	2744	LOC100288778(dist=4242),FAM138D(dist=52441)	intergenic	HIVID	Yang et al .2018	30271481	Tumor
chr12	51025170	2326	DIP2B	intronic	HIVID	Yang et al .2018	30271481	Tumor
chr12	62340088	1958	FAM19A2	intronic	HIVID	Yang et al .2018	30271481	Tumor
chr12	64241384	2326	SRGAP1	intronic	HIVID	Yang et al .2018	30271481	Tumor
chr12	66976914	2055	GRIP1	intronic	HIVID	Yang et al .2018	30271481	Tumor
chr12	113063862	1958	PTPN11(dist=116145),RPH3A(dist=165687)	intergenic	HIVID	Yang et al .2018	30271481	Tumor
chr12	121649638	2326	P2RX4	intronic	HIVID	Yang et al .2018	30271481	Tumor
chr12	123125294	2326	KNTC1(dist=14347),HCAR2(dist=60546)	intergenic	HIVID	Yang et al .2018	30271481	Tumor
chr12	127178458	2780	LOC100128554(dist=221127),LOC387895(dist=36789)	intergenic	HIVID	Yang et al .2018	30271481	Tumor
chr12	133841520	3020	ANHX(dist=29098),NONE(dist=NONE)	intergenic	HIVID	Yang et al .2018	30271481	Tumor
chr13	31881344	1959	B3GALTL	intronic	HIVID	Yang et al .2018	30271481	Tumor

Chromosome	Integration site in host genome	Integration site in virus genome	Gene (distance, bp)	Regions	Methods	Author	PMID	Sample
chr13	50575426	576	DLEU2	ncRNA_intronic	HIVID	Yang et al .2018	30271481	Tumor
chr14	22580262	2382	OR4E2(dist=446024),DAD1(dist=453545)	intergenic	HIVID	Yang et al .2018	30271481	Tumor
chr14	30955269	2417	PRKD1(dist=558370),G2E3(dist=73060)	intergenic	HIVID	Yang et al .2018	30271481	Tumor
chr15	44393205	2326	FRMD5	intronic	HIVID	Yang et al .2018	30271481	Tumor
chr15	45347040	2081	SORD	intronic	HIVID	Yang et al .2018	30271481	Tumor
chr15	66743577	2018	MAP2K1	intronic	HIVID	Yang et al .2018	30271481	Tumor
chr15	97809849	2373	SPATA8(dist=481004),LOC91948(dist=475997)	intergenic	HIVID	Yang et al .2018	30271481	Tumor
chr16	9679413	1958	MIR548X(dist=350610),GRIN2A(dist=167852)	intergenic	HIVID	Yang et al .2018	30271481	Tumor
chr16	34843913	2018	LOC100130700(dist=103073),RNA5SP411(dist=136981)	intergenic	HIVID	Yang et al .2018	30271481	Tumor
chr16	47679188	1958	PHKB	intronic	HIVID	Yang et al .2018	30271481	Tumor
chr16	48803680	2063	N4BP1(dist=159560),CBLN1(dist=508149)	intergenic	HIVID	Yang et al .2018	30271481	Tumor
chr16	60912748	1958	LOC644649(dist=1123653),CDH8(dist=773167)	intergenic	HIVID	Yang et al .2018	30271481	Tumor
chr16	70372449	2323	LOC100506083	ncRNA_intronic	HIVID	Yang et al .2018	30271481	Tumor
chr16	83675836	2018	CDH13	intronic	HIVID	Yang et al .2018	30271481	Tumor
chr16	89995531	2326	TUBB3	intronic	HIVID	Yang et al .2018	30271481	Tumor
chr17	11658129	1959	DNAH9	intronic	HIVID	Yang et al .2018	30271481	Tumor
chr17	54084996	1959	PCTP(dist=230248),ANKFN1(dist=145840)	intergenic	HIVID	Yang et al .2018	30271481	Tumor
chr17	74306238	2326	PRPSAP1	downstream	HIVID	Yang et al .2018	30271481	Tumor
chr18	540436	2081	COLEC12(dist=39707),CETN1(dist=39933)	intergenic	HIVID	Yang et al .2018	30271481	Tumor
chr18	8058517	2018	PTPRM	intronic	HIVID	Yang et al .2018	30271481	Tumor
chr18	38427068	1958	LINC00669(dist=1095109),KC6(dist=633168)	intergenic	HIVID	Yang et al .2018	30271481	Tumor
chr18	66526637	2018	CCDC102B	intronic	HIVID	Yang et al .2018	30271481	Tumor
chr18	68353500	2018	SOCS6(dist=356066),LOC100505776(dist=833700)	intergenic	HIVID	Yang et al .2018	30271481	Tumor
chr18	69324904	1958	LOC100505776(dist=78712),CBLN2(dist=879011)	intergenic	HIVID	Yang et al .2018	30271481	Tumor
chr18	76858232	1958	ATP9B	intronic	HIVID	Yang et al .2018	30271481	Tumor
chr18	78016183	2865	PARD6G(dist=10786),NONE(dist=NONE)	intergenic	HIVID	Yang et al .2018	30271481	Tumor
chr19	14321571	2326	LPHN1(dist=4574),CD97(dist=170385)	intergenic	HIVID	Yang et al .2018	30271481	Tumor
chr19	29955068	1958	LOC284395	ncRNA_intronic	HIVID	Yang et al .2018	30271481	Tumor
chr19	30633127	1958	URI1(dist=125608),ZNF536(dist=230201)	intergenic	HIVID	Yang et al .2018	30271481	Tumor
chr19	48570585	2326	PLA2G4C	intronic	HIVID	Yang et al .2018	30271481	Tumor
chr19	54900979	2326	LAIR1(dist=24258),TTYH1(dist=25626)	intergenic	HIVID	Yang et al .2018	30271481	Tumor
chr2	3184873	2067	MYT1L(dist=849828),TSSC1(dist=7868)	intergenic	HIVID	Yang et al .2018	30271481	Tumor
chr2	27777601	2326	GCKR(dist=31051),C2orf16(dist=21788)	intergenic	HIVID	Yang et al .2018	30271481	Tumor
chr2	30532334	2326	LBH(dist=49435),LCLAT1(dist=137789)	intergenic	HIVID	Yang et al .2018	30271481	Tumor
chr2	39394148	117	SOS1(dist=46544),CDKL4(dist=11540)	intergenic	HIVID	Yang et al .2018	30271481	Tumor
chr2	44643874	2269	CAMKMT	intronic	HIVID	Yang et al .2018	30271481	Tumor
chr2	53626813	1958	NONE(dist=NONE),ASB3(dist=270304)	intergenic	HIVID	Yang et al .2018	30271481	Tumor
chr2	55429550	2326	CLHC1	intronic	HIVID	Yang et al .2018	30271481	Tumor
chr2	87998719	1958	MIR4435-1(dist=69366),RGPD1(dist=56760)	intergenic	HIVID	Yang et al .2018	30271481	Tumor
chr2	106752316	2018	UXS1	intronic	HIVID	Yang et al .2018	30271481	Tumor
chr2	118508646	2018	DPP10(dist=1906320),DDX18(dist=63609)	intergenic	HIVID	Yang et al .2018	30271481	Tumor
chr2	123285067	693	TSN(dist=759639),CNTNAP5(dist=1497797)	intergenic	HIVID	Yang et al .2018	30271481	Tumor
chr2	134967825	1958	MIR3679(dist=83062),MGAT5(dist=44005)	intergenic	HIVID	Yang et al .2018	30271481	Tumor
chr2	150806744	2018	MMADHC(dist=362414),RND3(dist=517963)	intergenic	HIVID	Yang et al .2018	30271481	Tumor
chr2	155846245	2018	KCNJ3(dist=131381),NR4A2(dist=1334699)	intergenic	HIVID	Yang et al .2018	30271481	Tumor
chr2	164979068	2018	FIGN(dist=386555),GRB14(dist=370255)	intergenic	HIVID	Yang et al .2018	30271481	Tumor
chr2	187533113	2326	ITGAV	intronic	HIVID	Yang et al .2018	30271481	Tumor
chr2	200963800	2326	C2orf47(dist=134953),SPATS2L(dist=206804)	intergenic	HIVID	Yang et al .2018	30271481	Tumor
chr2	223012861	2018	EPHA4(dist=575851),PAX3(dist=51745)	intergenic	HIVID	Yang et al .2018	30271481	Tumor
chr2	223015387	2018	EPHA4(dist=578377),PAX3(dist=49219)	intergenic	HIVID	Yang et al .2018	30271481	Tumor
chr20	4368244	1958	ADRA1D(dist=138585),PRNP(dist=298553)	intergenic	HIVID	Yang et al .2018	30271481	Tumor
chr20	5361367	2018	PROKR2(dist=66352),LINC00658(dist=51236)	intergenic	HIVID	Yang et al .2018	30271481	Tumor
chr20	12782760	1959	BTBD3(dist=875517),SPTLC3(dist=206867)	intergenic	HIVID	Yang et al .2018	30271481	Tumor
chr20	19069860	1958	C20orf78(dist=259021),SLC24A3(dist=123430)	intergenic	HIVID	Yang et al .2018	30271481	Tumor
chr20	19401688	1958	SLC24A3	intronic	HIVID	Yang et al .2018	30271481	Tumor
chr20	48791579	2326	TMEM189-UBE2V1(dist=21244),CEBPB(dist=15541)	intergenic	HIVID	Yang et al .2018	30271481	Tumor
chr20	52271358	2326	ZNF217(dist=71722),SUMO1P1(dist=219682)	intergenic	HIVID	Yang et al .2018	30271481	Tumor
chr21	21572882	2018	TMPRSS15(dist=1796912),LINC00320(dist=542031)	intergenic	HIVID	Yang et al .2018	30271481	Tumor
chr21	48119752	2865	PRMT2(dist=34597),NONE(dist=NONE)	intergenic	HIVID	Yang et al .2018	30271481	Tumor
chr22	25164381	2326	PIWIL3	intronic	HIVID	Yang et al .2018	30271481	Tumor
chr22	31553686	2319	PLA2G3(dist=17217),MIR3928(dist=2362)	intergenic	HIVID	Yang et al .2018	30271481	Tumor
chr22	40698495	2326	TNRC6B	intronic	HIVID	Yang et al .2018	30271481	Tumor
chr3	2049149	2373	CNTN6(dist=603871),CNTN4(dist=91401)	intergenic	HIVID	Yang et al .2018	30271481	Tumor
chr3	10130827	2326	C3orf24,FANCD2	intronic	HIVID	Yang et al .2018	30271481	Tumor
chr3	46825663	2018	PRSS45(dist=39418),PRSS42(dist=46231)	intergenic	HIVID	Yang et al .2018	30271481	Tumor
chr3	53400387	2018	DCP1A(dist=18733),CACNA1D(dist=128644)	intergenic	HIVID	Yang et al .2018	30271481	Tumor
chr3	54429406	2018	CACNA2D3	intronic	HIVID	Yang et al .2018	30271481	Tumor
chr3	106354927	2896	CBLB(dist=767040),LOC100302640(dist=473710)	intergenic	HIVID	Yang et al .2018	30271481	Tumor
chr3	108473108	1958	DZIP3(dist=59415),RETNLB(dist=1378)	intergenic	HIVID	Yang et al .2018	30271481	Tumor
chr3	110722620	1959	FLJ25363(dist=1508606),PVRL3-AS1(dist=41543)	intergenic	HIVID	Yang et al .2018	30271481	Tumor
chr3	138708732	2326	C3orf72(dist=35902),PRR23A(dist=14072)	intergenic	HIVID	Yang et al .2018	30271481	Tumor
chr3	158357168	1958	MLF1(dist=32919),GFM1(dist=5149)	intergenic	HIVID	Yang et al .2018	30271481	Tumor
chr3	162959690	2018	LOC647107	ncRNA_intronic	HIVID	Yang et al .2018	30271481	Tumor
chr3	183457771	1959	YEATS2	intronic	HIVID	Yang et al .2018	30271481	Tumor
chr3	183646060	2326	ABCC5	intronic	HIVID	Yang et al .2018	30271481	Tumor
chr3	195554652	2326	MUC4(dist=15808),TNK2(dist=35584)	intergenic	HIVID	Yang et al .2018	30271481	Tumor
chr4	27378377	2018	STIM2(dist=351374),MIR4275(dist=1442827)	intergenic	HIVID	Yang et al .2018	30271481	Tumor
chr4	32519894	1959	PCDH7(dist=1371471),NONE(dist=NONE)	intergenic	HIVID	Yang et al .2018	30271481	Tumor
chr4	37106109	1958	DTHD1(dist=758731),MIR4801(dist=137423)	intergenic	HIVID	Yang et al .2018	30271481	Tumor
chr4	48172926	2326	TEC	intronic	HIVID	Yang et al .2018	30271481	Tumor
chr4	59443517	2018	LOC255130(dist=1372052),NONE(dist=NONE)	intergenic	HIVID	Yang et al .2018	30271481	Tumor
chr4	77750453	2018	SHROOM3(dist=46048),SOWAHB(dist=65629)	intergenic	HIVID	Yang et al .2018	30271481	Tumor
chr4	88485129	1958	SPARCL1(dist=34474),DSPP(dist=44552)	intergenic	HIVID	Yang et al .2018	30271481	Tumor
chr4	88824601	2326	HSP90AB3P(dist=9434),SPP1(dist=72201)	intergenic	HIVID	Yang et al .2018	30271481	Tumor
chr4	94530449	1959	GRID2	intronic	HIVID	Yang et al .2018	30271481	Tumor
chr4	95039763	558	ATOH1(dist=288621),SMARCAD1(dist=88996)	intergenic	HIVID	Yang et al .2018	30271481	Tumor
chr4	95039787	504	ATOH1(dist=288645),SMARCAD1(dist=88972)	intergenic	HIVID	Yang et al .2018	30271481	Tumor
chr4	95046218	1538	ATOH1(dist=295076),SMARCAD1(dist=82541)	intergenic	HIVID	Yang et al .2018	30271481	Tumor
chr4	95059789	2263	ATOH1(dist=308647),SMARCAD1(dist=68970)	intergenic	HIVID	Yang et al .2018	30271481	Tumor
chr4	106229460	2018	TET2(dist=28500),PPA2(dist=60774)	intergenic	HIVID	Yang et al .2018	30271481	Tumor
chr4	120870948	2018	PDE5A(dist=320967),MAD2L1(dist=109631)	intergenic	HIVID	Yang et al .2018	30271481	Tumor
chr4	145669363	2018	HHIP(dist=9482),ANAPC10(dist=246364)	intergenic	HIVID	Yang et al .2018	30271481	Tumor
chr4	156970012	2018	CTSO(dist=94964),PDGFC(dist=712751)	intergenic	HIVID	Yang et al .2018	30271481	Tumor
chr4	159395667	2018	TMEM144(dist=219228),RXFP1(dist=47199)	intergenic	HIVID	Yang et al .2018	30271481	Tumor

Chromosome	Integration site in host genome	Integration site in virus genome	Gene (distance, bp)	Regions	Methods	Author	PMID	Sample
chr4	167495995	1958	TLL1(dist=470386),SPOCK3(dist=158541)	intergenic	HIVID	Yang et al .2018	30271481	Tumor
chr4	175704644	1962	GLRA3	intronic	HIVID	Yang et al .2018	30271481	Tumor
chr4	191044185	2744	DUX4L2(dist=30709),NONE(dist=NONE)	intergenic	HIVID	Yang et al .2018	30271481	Tumor
chr5	6970622	1958	MIR4278(dist=142588),MIR4454(dist=298794)	intergenic	HIVID	Yang et al .2018	30271481	Tumor
chr5	9261857	1958	SEMA5A	intronic	HIVID	Yang et al .2018	30271481	Tumor
chr5	10614857	2326	ANKRD33B	intronic	HIVID	Yang et al .2018	30271481	Tumor
chr5	32736195	2326	NPR3	intronic	HIVID	Yang et al .2018	30271481	Tumor
chr5	42167335	1958	FBXO4(dist=225663),GHR(dist=256542)	intergenic	HIVID	Yang et al .2018	30271481	Tumor
chr5	53478042	1958	ARL15	intronic	HIVID	Yang et al .2018	30271481	Tumor
chr5	64005928	2018	FAM159B	intronic	HIVID	Yang et al .2018	30271481	Tumor
chr5	81322736	2326	ATG10	intronic	HIVID	Yang et al .2018	30271481	Tumor
chr5	84655512	1958	EDIL3(dist=974901),NBPF22P(dist=922750)	intergenic	HIVID	Yang et al .2018	30271481	Tumor
chr5	90569370	2018	GPR98(dist=109337),ARRDC3(dist=95171)	intergenic	HIVID	Yang et al .2018	30271481	Tumor
chr5	110452744	558	WDR36	intronic	HIVID	Yang et al .2018	30271481	Tumor
chr5	121579333	2063	LOC100505841(dist=60975),SNCAIP(dist=68487)	intergenic	HIVID	Yang et al .2018	30271481	Tumor
chr5	154789717	2326	KIF4B(dist=392032),SGCD(dist=964050)	intergenic	HIVID	Yang et al .2018	30271481	Tumor
chr5	157722751	2326	CLINT1(dist=436568),EBF1(dist=400172)	intergenic	HIVID	Yang et al .2018	30271481	Tumor
chr5	166394853	2018	NONE(dist=NONE),ODZ2(dist=316990)	intergenic	HIVID	Yang et al .2018	30271481	Tumor
chr5	169554724	2326	FOXI1(dist=17995),C5orf58(dist=105226)	intergenic	HIVID	Yang et al .2018	30271481	Tumor
chr6	16861170	1958	ATXN1(dist=99449),FLJ23152(dist=241319)	intergenic	HIVID	Yang et al .2018	30271481	Tumor
chr6	19769985	1958	MIR548A1(dist=1197874),ID4(dist=67616)	intergenic	HIVID	Yang et al .2018	30271481	Tumor
chr6	51744413	1958	PKHD1	intronic	HIVID	Yang et al .2018	30271481	Tumor
chr6	54004514	151	MLIP	intronic	HIVID	Yang et al .2018	30271481	Tumor
chr6	121488806	2018	C6orf170	intronic	HIVID	Yang et al .2018	30271481	Tumor
chr6	125161717	1958	NKAIN2(dist=14931),STL(dist=67675)	intergenic	HIVID	Yang et al .2018	30271481	Tumor
chr6	131866483	2280	AKAP7(dist=261808),ARG1(dist=27861)	intergenic	HIVID	Yang et al .2018	30271481	Tumor
chr6	133791293	2018	EYA4	intronic	HIVID	Yang et al .2018	30271481	Tumor
chr6	159969311	2018	FNDC1(dist=276171),SOD2(dist=130838)	intergenic	HIVID	Yang et al .2018	30271481	Tumor
chr7	1189649	2326	C7orf50(dist=11756),ZFAND2A(dist=2894)	intergenic	HIVID	Yang et al .2018	30271481	Tumor
chr7	7509713	2381	COL28A1	intronic	HIVID	Yang et al .2018	30271481	Tumor
chr7	24042108	2018	STK31(dist=169978),NPY(dist=281699)	intergenic	HIVID	Yang et al .2018	30271481	Tumor
chr7	61291751	2018	NONE(dist=NONE),LOC643955(dist=1459919)	intergenic	HIVID	Yang et al .2018	30271481	Tumor
chr7	62112511	463	NONE(dist=NONE),LOC643955(dist=639159)	intergenic	HIVID	Yang et al .2018	30271481	Tumor
chr7	93418026	2018	MIR4652(dist=71709),TFPI2(dist=96683)	intergenic	HIVID	Yang et al .2018	30271481	Tumor
chr7	95222918	2140	PDK4	intronic	HIVID	Yang et al .2018	30271481	Tumor
chr7	99411691	1958	CYP3A4(dist=29880),CYP3A43(dist=13945)	intergenic	HIVID	Yang et al .2018	30271481	Tumor
chr7	106668067	2326	PIK3CG(dist=120475),PRKAR2B(dist=17111)	intergenic	HIVID	Yang et al .2018	30271481	Tumor
chr7	111608213	2018	DOCK4	intronic	HIVID	Yang et al .2018	30271481	Tumor
chr7	120461533	3172	TSPAN12	intronic	HIVID	Yang et al .2018	30271481	Tumor
chr7	127604372	2018	SND1	intronic	HIVID	Yang et al .2018	30271481	Tumor
chr7	140762881	2018	MRPS33(dist=48100),TMEM178B(dist=11151)	intergenic	HIVID	Yang et al .2018	30271481	Tumor
chr8	4866809	2326	CSMD1(dist=14481),LOC100287015(dist=1394268)	intergenic	HIVID	Yang et al .2018	30271481	Tumor
chr8	25588922	1958	CDCA2(dist=223497),EBF2(dist=110324)	intergenic	HIVID	Yang et al .2018	30271481	Tumor
chr8	41838691	2326	KAT6A	intronic	HIVID	Yang et al .2018	30271481	Tumor
chr8	51078788	2016	SNTG1	intronic	HIVID	Yang et al .2018	30271481	Tumor
chr8	59832115	1958	TOX	intronic	HIVID	Yang et al .2018	30271481	Tumor
chr8	96565527	2018	LOC100616530	ncRNA_intronic	HIVID	Yang et al .2018	30271481	Tumor
chr8	114071523	2416	CSMD3	intronic	HIVID	Yang et al .2018	30271481	Tumor
chr8	114686300	2016	CSMD3(dist=237058),TRPS1(dist=1734424)	intergenic	HIVID	Yang et al .2018	30271481	Tumor
chr8	120612105	2326	ENPP2	intronic	HIVID	Yang et al .2018	30271481	Tumor
chr8	132125293	2068	ADCY8(dist=72458),EFR3A(dist=791063)	intergenic	HIVID	Yang et al .2018	30271481	Tumor
chr9	16466778	2018	BNC2	intronic	HIVID	Yang et al .2018	30271481	Tumor
chr9	28112973	2018	LINGO2	intronic	HIVID	Yang et al .2018	30271481	Tumor
chr9	33966632	2125	UBAP2	intronic	HIVID	Yang et al .2018	30271481	Tumor
chr9	36013525	2743	OR2S2(dist=55374),RECK(dist=23385)	intergenic	HIVID	Yang et al .2018	30271481	Tumor
chr9	88427530	1958	LOC389765	ncRNA_intronic	HIVID	Yang et al .2018	30271481	Tumor
chr9	113558245	1958	MUSK	intronic	HIVID	Yang et al .2018	30271481	Tumor
chrX	6503122	2018	VCX3A(dist=49963),HDHD1(dist=463839)	intergenic	HIVID	Yang et al .2018	30271481	Tumor
chrX	9966731	2326	LOC100288814(dist=30689),WWC3(dist=17064)	intergenic	HIVID	Yang et al .2018	30271481	Tumor
chrX	20354891	1958	RPS6KA3(dist=70141),CNKSR2(dist=1037645)	intergenic	HIVID	Yang et al .2018	30271481	Tumor
chrX	34838561	2018	TMEM47(dist=163156),FAM47B(dist=122352)	intergenic	HIVID	Yang et al .2018	30271481	Tumor
chrX	43460165	2326	PPP1R2P9(dist=822679),MAOA(dist=53990)	intergenic	HIVID	Yang et al .2018	30271481	Tumor
chrX	54178851	2326	FAM120C	intronic	HIVID	Yang et al .2018	30271481	Tumor
chrX	56278056	2326	KLF8	intronic	HIVID	Yang et al .2018	30271481	Tumor
chrX	58160769	2018	ZXDA(dist=223702),NONE(dist=NONE)	intergenic	HIVID	Yang et al .2018	30271481	Tumor
chrX	65357812	1958	VSIG4(dist=97845),HEPH(dist=24621)	intergenic	HIVID	Yang et al .2018	30271481	Tumor
chrX	79022159	2018	ITM2A(dist=399110),TBX22(dist=248096)	intergenic	HIVID	Yang et al .2018	30271481	Tumor
chrX	79789831	2018	FAM46D(dist=89021),BRWD3(dist=135156)	intergenic	HIVID	Yang et al .2018	30271481	Tumor
chrX	85204886	150	CHM	intronic	HIVID	Yang et al .2018	30271481	Tumor
chrX	85815778	1959	DACH2	intronic	HIVID	Yang et al .2018	30271481	Tumor
chrX	109666414	2326	AMMECR1,RGAG1	intronic	HIVID	Yang et al .2018	30271481	Tumor
chrX	120398713	2018	GLUD2(dist=214917),GRIA3(dist=1919383)	intergenic	HIVID	Yang et al .2018	30271481	Tumor
chrX	122886376	2326	THOC2(dist=19472),XIAP(dist=107286)	intergenic	HIVID	Yang et al .2018	30271481	Tumor
chrX	126501073	1958	CXorf64(dist=545305),ACTRT1(dist=683868)	intergenic	HIVID	Yang et al .2018	30271481	Tumor
chrX	130097768	1958	ENOX2(dist=60560),ARHGAP36(dist=94448)	intergenic	HIVID	Yang et al .2018	30271481	Tumor
chrX	145063084	2326	CXorf1(dist=151714),MIR890(dist=12709)	intergenic	HIVID	Yang et al .2018	30271481	Tumor
chrX	148054369	2018	AFF2	intronic	HIVID	Yang et al .2018	30271481	Tumor
chrX	153483642	2326	OPN1MW(dist=21290),OPN1MW2(dist=1561)	intergenic	HIVID	Yang et al .2018	30271481	Tumor
chrX	155260333	2745	IL9R(dist=19851),NONE(dist=NONE)	intergenic	HIVID	Yang et al .2018	30271481	Tumor
chrY	5065084	2018	PCDH11Y	intronic	HIVID	Yang et al .2018	30271481	Tumor
chrY	5475276	2018	PCDH11Y	intronic	HIVID	Yang et al .2018	30271481	Tumor
chrY	9428402	2018	TSPY3(dist=60117),RBMY3AP(dist=19928)	intergenic	HIVID	Yang et al .2018	30271481	Tumor
chrY	9783598	1958	TTTY23(dist=34027),NONE(dist=NONE)	intergenic	HIVID	Yang et al .2018	30271481	Tumor
chrY	15292176	2081	DDX3Y(dist=259786),UTY(dist=68083)	intergenic	HIVID	Yang et al .2018	30271481	Tumor
chrY	59363338	2744	IL9R(dist=19850),NONE(dist=NONE)	intergenic	HIVID	Yang et al .2018	30271481	Tumor
chr1	73957846	236	NEGR1(dist=1209569),LRRIQ3(dist=533856)	intergenic	HIVID	Yang et al .2018	30271481	Tumor
chr14	30955269	2417	PRKD1(dist=558370),G2E3(dist=73060)	intergenic	HIVID	Yang et al .2018	30271481	Tumor
chr14	94542629	560	DDX24	intronic	HIVID	Yang et al .2018	30271481	Tumor
chr17	15943040	2824	NCOR1	intronic	HIVID	Yang et al .2018	30271481	Tumor
chr17	15943095	2426	NCOR1	intronic	HIVID	Yang et al .2018	30271481	Tumor
chr17	15943285	1823	NCOR1	intronic	HIVID	Yang et al .2018	30271481	Tumor
chr17	20606088	1079	CDRT15L2(dist=121864),CCDC144NL(dist=160620)	intergenic	HIVID	Yang et al .2018	30271481	Tumor
chr3	154189341	256	GPR149(dist=41837),MME(dist=608095)	intergenic	HIVID	Yang et al .2018	30271481	Tumor
chr4	95039765	616	ATOH1(dist=288623),SMARCAD1(dist=88994)	intergenic	HIVID	Yang et al .2018	30271481	Tumor

Chromosome	Integration site in host genome	Integration site in virus genome	Gene (distance, bp)	Regions	Methods	Author	PMID	Sample
chr6	69073237	494	NONE(dist=NONE),BAI3(dist=272395)	intergenic	HIVID	Yang et al .2018	30271481	Tumor
chr6	154695991	2269	IPCEF1(dist=18091),CNKSR3(dist=30442)	intergenic	HIVID	Yang et al .2018	30271481	Tumor
chr10	42386955	2992	NONE(dist=NONE),LOC441666(dist=440359)	intergenic	HIVID	Yang et al .2018	30271481	Tumor
chr14	30955270	2417	PRKD1(dist=558371),G2E3(dist=73059)	intergenic	HIVID	Yang et al .2018	30271481	Tumor
chr4	95039765	616	ATOH1(dist=288623),SMARCAD1(dist=88994)	intergenic	HIVID	Yang et al .2018	30271481	Tumor
chr4	95046268	1538	ATOH1(dist=295126),SMARCAD1(dist=82491)	intergenic	HIVID	Yang et al .2018	30271481	Tumor
chr1	10110	2390	NONE(dist=NONE),DDX11L1(dist=1764)	intergenic	HIVID	Yang et al .2018	30271481	Tumor
chr1	185664789	285	LOC100288079(dist=360618),HMCN1(dist=38894)	intergenic	HIVID	Yang et al .2018	30271481	Tumor
chr1	185674893	1832	LOC100288079(dist=370722),HMCN1(dist=28790)	intergenic	HIVID	Yang et al .2018	30271481	Tumor
chr1	247267270	1765	ZNF669	exonic	HIVID	Yang et al .2018	30271481	Tumor
chr10	42385614	1816	NONE(dist=NONE),LOC441666(dist=441700)	intergenic	HIVID	Yang et al .2018	30271481	Tumor
chr10	42387169	1816	NONE(dist=NONE),LOC441666(dist=440145)	intergenic	HIVID	Yang et al .2018	30271481	Tumor
chr10	42387718	1620	NONE(dist=NONE),LOC441666(dist=439596)	intergenic	HIVID	Yang et al .2018	30271481	Tumor
chr10	42389385	1769	NONE(dist=NONE),LOC441666(dist=437929)	intergenic	HIVID	Yang et al .2018	30271481	Tumor
chr10	42393009	1816	NONE(dist=NONE),LOC441666(dist=434305)	intergenic	HIVID	Yang et al .2018	30271481	Tumor
chr10	42596705	1816	NONE(dist=NONE),LOC441666(dist=230609)	intergenic	HIVID	Yang et al .2018	30271481	Tumor
chr10	42598063	1816	NONE(dist=NONE),LOC441666(dist=229251)	intergenic	HIVID	Yang et al .2018	30271481	Tumor
chr10	53089051	2985	PRKG1	intronic	HIVID	Yang et al .2018	30271481	Tumor
chr10	88512743	1837	LDB3(dist=16919),BMPR1A(dist=3653)	intergenic	HIVID	Yang et al .2018	30271481	Tumor
chr10	88541379	234	BMPR1A	intronic	HIVID	Yang et al .2018	30271481	Tumor
chr11	14702850	1823	PDE3B	intronic	HIVID	Yang et al .2018	30271481	Tumor
chr11	30193847	1574	KCNA4(dist=155270),FSHB(dist=58716)	intergenic	HIVID	Yang et al .2018	30271481	Tumor
chr11	46132675	409	PHF21A	intronic	HIVID	Yang et al .2018	30271481	Tumor
chr11	94556414	1973	AMOTL1	intronic	HIVID	Yang et al .2018	30271481	Tumor
chr12	2250290	439	CACNA1C	intronic	HIVID	Yang et al .2018	30271481	Tumor
chr14	81239082	1828	CEP128	intronic	HIVID	Yang et al .2018	30271481	Tumor
chr15	90454180	1829	C15orf38,C15orf38-AP3S2	intronic	HIVID	Yang et al .2018	30271481	Tumor
chr15	90475471	1815	C15orf38-AP3S2(dist=19249),ZNF710(dist=69281)	intergenic	HIVID	Yang et al .2018	30271481	Tumor
chr16	75368094	1814	CFDP1	intronic	HIVID	Yang et al .2018	30271481	Tumor
chr17	12388503	1819	MAP2K4(dist=341452),LINC00670(dist=64782)	intergenic	HIVID	Yang et al .2018	30271481	Tumor
chr17	22253631	1802	MTRNR2L1(dist=229640),NONE(dist=NONE)	intergenic	HIVID	Yang et al .2018	30271481	Tumor
chr17	22256010	1802	MTRNR2L1(dist=232019),NONE(dist=NONE)	intergenic	HIVID	Yang et al .2018	30271481	Tumor
chr18	23682640	677	SS18(dist=12029),PSMA8(dist=31176)	intergenic	HIVID	Yang et al .2018	30271481	Tumor
chr18	71132732	984	LOC100505817(dist=115608),FBXO15(dist=607856)	intergenic	HIVID	Yang et al .2018	30271481	Tumor
chr18	71276484	936	LOC100505817(dist=259360),FBXO15(dist=464104)	intergenic	HIVID	Yang et al .2018	30271481	Tumor
chr19	21788223	1823	ZNF429(dist=67144),ZNF100(dist=118620)	intergenic	HIVID	Yang et al .2018	30271481	Tumor
chr19	36212468	1704	KMT2B	exonic	HIVID	Yang et al .2018	30271481	Tumor
chr2	99818030	1010	MRPL30(dist=2010),LYG2(dist=40681)	intergenic	HIVID	Yang et al .2018	30271481	Tumor
chr2	99818050	1754	MRPL30(dist=2030),LYG2(dist=40661)	intergenic	HIVID	Yang et al .2018	30271481	Tumor
chr20	30564095	1557	XKR7	intronic	HIVID	Yang et al .2018	30271481	Tumor
chr20	30564101	1808	XKR7	intronic	HIVID	Yang et al .2018	30271481	Tumor
chr22	49082817	1917	FAM19A5	intronic	HIVID	Yang et al .2018	30271481	Tumor
chr3	192952816	1040	MB21D2(dist=316866),HRASLS(dist=6101)	intergenic	HIVID	Yang et al .2018	30271481	Tumor
chr3	196625630	1816	SENP5	intronic	HIVID	Yang et al .2018	30271481	Tumor
chr4	9671023	1707	MIR548I2(dist=113086),DRD5(dist=112235)	intergenic	HIVID	Yang et al .2018	30271481	Tumor
chr4	18491815	598	LCORL(dist=468332),SLIT2(dist=1763420)	intergenic	HIVID	Yang et al .2018	30271481	Tumor
chr4	43198639	1577	GRXCR1(dist=165964),KCTD8(dist=977281)	intergenic	HIVID	Yang et al .2018	30271481	Tumor
chr4	67590201	2075	LOC100144602(dist=1031097),CENPC1(dist=747788)	intergenic	HIVID	Yang et al .2018	30271481	Tumor
chr4	166602912	970	CPE(dist=183430),TLL1(dist=191498)	intergenic	HIVID	Yang et al .2018	30271481	Tumor
chr5	1286631	124	TERT	intronic	HIVID	Yang et al .2018	30271481	Tumor
chr5	1296885	140	TERT(dist=1723),MIR4457(dist=12540)	intergenic	HIVID	Yang et al .2018	30271481	Tumor
chr5	129935404	418	CHSY3(dist=413077),HINT1(dist=559471)	intergenic	HIVID	Yang et al .2018	30271481	Tumor
chr7	92156773	807	PEX1	intronic	HIVID	Yang et al .2018	30271481	Tumor
chr8	51787482	301	SNTG1(dist=82055),PXDNL(dist=444655)	intergenic	HIVID	Yang et al .2018	30271481	Tumor
chr9	28517157	2064	LINGO2	intronic	HIVID	Yang et al .2018	30271481	Tumor
chrX	134778636	1739	DDX26B(dist=62176),CT45A1(dist=68549)	intergenic	HIVID	Yang et al .2018	30271481	Tumor
chrY	59363287	1814	IL9R(dist=19799),NONE(dist=NONE)	intergenic	HIVID	Yang et al .2018	30271481	Tumor
chr14	78981628	1828	NRXN3	intronic	HIVID	Yang et al .2018	30271481	Tumor
chr15	90475471	1815	C15orf38-AP3S2(dist=19249),ZNF710(dist=69281)	intergenic	HIVID	Yang et al .2018	30271481	Tumor
chr16	46392261	3201	NONE(dist=NONE),ANKRD26P1(dist=110988)	intergenic	HIVID	Yang et al .2018	30271481	Tumor
chr16	46398075	3201	NONE(dist=NONE),ANKRD26P1(dist=105174)	intergenic	HIVID	Yang et al .2018	30271481	Tumor
chr16	46404181	3201	NONE(dist=NONE),ANKRD26P1(dist=99068)	intergenic	HIVID	Yang et al .2018	30271481	Tumor
chr16	46428618	3201	NONE(dist=NONE),ANKRD26P1(dist=74631)	intergenic	HIVID	Yang et al .2018	30271481	Tumor
chr18	71814075	2306	FBXO15	intronic	HIVID	Yang et al .2018	30271481	Tumor
chr2	171274896	1765	MYO3B	intronic	HIVID	Yang et al .2018	30271481	Tumor
chr2	171274934	1828	MYO3B	intronic	HIVID	Yang et al .2018	30271481	Tumor
chr4	9671023	1707	MIR548I2(dist=113086),DRD5(dist=112235)	intergenic	HIVID	Yang et al .2018	30271481	Tumor
chr6	154477149	1433	IPCEF1	UTR3	HIVID	Yang et al .2018	30271481	Tumor
chr7	76835294	41	CCDC146	intronic	HIVID	Yang et al .2018	30271481	Tumor
chr8	51787544	311	SNTG1(dist=82117),PXDNL(dist=444593)	intergenic	HIVID	Yang et al .2018	30271481	Tumor
chr8	97587834	1551	SDC2	intronic	HIVID	Yang et al .2018	30271481	Tumor
chr8	97587863	1757	SDC2	intronic	HIVID	Yang et al .2018	30271481	Tumor
chr17	14688203	2004	HS3ST3B1(dist=438711),CDRT7(dist=246089)	intergenic	HIVID	Yang et al .2018	30271481	Tumor
chr19	36212805	2553	KMT2B	intronic	HIVID	Yang et al .2018	30271481	Tumor
chr19	36212865	1814	KMT2B	intronic	HIVID	Yang et al .2018	30271481	Tumor
chr2	74184935	1849	DGUOK	intronic	HIVID	Yang et al .2018	30271481	Tumor
chr2	74184982	917	DGUOK	intronic	HIVID	Yang et al .2018	30271481	Tumor
chr4	180746440	1825	LOC285501(dist=1834536),LINC00290(dist=1238803)	intergenic	HIVID	Yang et al .2018	30271481	Tumor
chr5	38576265	312	LIFR	intronic	HIVID	Yang et al .2018	30271481	Tumor
chr8	18864613	3144	PSD3	intronic	HIVID	Yang et al .2018	30271481	Tumor
chr8	123092449	260	HAS2-AS1(dist=434885),ZHX2(dist=701452)	intergenic	HIVID	Yang et al .2018	30271481	Tumor
chr9	128759021	662	PBX3(dist=29366),FAM125B(dist=330102)	intergenic	HIVID	Yang et al .2018	30271481	Tumor
chr1	185674893	1832	LOC100288079(dist=370722),HMCN1(dist=28790)	intergenic	HIVID	Yang et al .2018	30271481	Tumor
chr10	88512743	1837	LDB3(dist=16919),BMPR1A(dist=3653)	intergenic	HIVID	Yang et al .2018	30271481	Tumor
chr10	88512748	2325	LDB3(dist=16924),BMPR1A(dist=3648)	intergenic	HIVID	Yang et al .2018	30271481	Tumor
chr10	88541377	235	BMPR1A	intronic	HIVID	Yang et al .2018	30271481	Tumor
chr12	893268	1957	WNK1	intronic	HIVID	Yang et al .2018	30271481	Tumor
chr12	2250290	439	CACNA1C	intronic	HIVID	Yang et al .2018	30271481	Tumor
chr12	3098348	2634	TEAD4	intronic	HIVID	Yang et al .2018	30271481	Tumor
chr12	20691684	26	PDE3A	intronic	HIVID	Yang et al .2018	30271481	Tumor
chr12	27236556	2364	C12orf71(dist=1101),STK38L(dist=160522)	intergenic	HIVID	Yang et al .2018	30271481	Tumor
chr12	27236720	753	C12orf71(dist=1265),STK38L(dist=160358)	intergenic	HIVID	Yang et al .2018	30271481	Tumor
chr12	29031735	730	CCDC91(dist=328636),FAR2(dist=344863)	intergenic	HIVID	Yang et al .2018	30271481	Tumor
chr12	50261607	1263	FAIM2	UTR3	HIVID	Yang et al .2018	30271481	Tumor

Chromosome	Integration site in host genome	Integration site in virus genome	Gene (distance, bp)	Regions	Methods	Author	PMID	Sample
chr12	55986209	2876	OR2AP1(dist=17081),OR10P1(dist=44467)	intergenic	HIVID	Yang et al .2018	30271481	Tumor
chr12	88431764	2902	C12orf29	intronic	HIVID	Yang et al .2018	30271481	Tumor
chr12	113319973	2189	RPH3A	intronic	HIVID	Yang et al .2018	30271481	Tumor
chr15	90475471	1815	C15orf38-AP3S2(dist=19249),ZNF710(dist=69281)	intergenic	HIVID	Yang et al .2018	30271481	Tumor
chr16	67674887	1457	CTCF(dist=1799),RLTPR(dist=4143)	intergenic	HIVID	Yang et al .2018	30271481	Tumor
chr16	67709429	1041	GFOD2	exonic	HIVID	Yang et al .2018	30271481	Tumor
chr18	71132732	984	LOC100505817(dist=115608),FBXO15(dist=607856)	intergenic	HIVID	Yang et al .2018	30271481	Tumor
chr19	36212557	1808	KMT2B	exonic	HIVID	Yang et al .2018	30271481	Tumor
chr19	36212696	2229	KMT2B	exonic	HIVID	Yang et al .2018	30271481	Tumor
chr2	99818050	1793	MRPL30(dist=2030),LYG2(dist=40661)	intergenic	HIVID	Yang et al .2018	30271481	Tumor
chr22	16598657	438	OR11H1(dist=148853),CCT8L2(dist=472991)	intergenic	HIVID	Yang et al .2018	30271481	Tumor
chr22	49082817	1917	FAM19A5	intronic	HIVID	Yang et al .2018	30271481	Tumor
chr3	141188454	2436	ZBTB38(dist=19822),RASA2(dist=17472)	intergenic	HIVID	Yang et al .2018	30271481	Tumor
chr4	9671023	1707	MIR548I2(dist=113086),DRD5(dist=112235)	intergenic	HIVID	Yang et al .2018	30271481	Tumor
chr4	166602910	970	CPE(dist=183428),TLL1(dist=191500)	intergenic	HIVID	Yang et al .2018	30271481	Tumor
chr5	1295701	1813	TERT	upstream	HIVID	Yang et al .2018	30271481	Tumor
chr5	1295777	1691	TERT	upstream	HIVID	Yang et al .2018	30271481	Tumor
chr5	86979814	256	CCNH(dist=270964),TMEM161B(dist=511209)	intergenic	HIVID	Yang et al .2018	30271481	Tumor
chr5	129935404	418	CHSY3(dist=413077),HINT1(dist=559471)	intergenic	HIVID	Yang et al .2018	30271481	Tumor
chr6	163098968	1145	PARK2	intronic	HIVID	Yang et al .2018	30271481	Tumor
chr8	3420958	2170	CSMD1	intronic	HIVID	Yang et al .2018	30271481	Tumor
chr8	51787482	301	SNTG1(dist=82055),PXDNL(dist=444655)	intergenic	HIVID	Yang et al .2018	30271481	Tumor
chr8	97587863	1757	SDC2	intronic	HIVID	Yang et al .2018	30271481	Tumor
chr9	29769211	2226	LINGO2(dist=556213),LOC401497(dist=619722)	intergenic	HIVID	Yang et al .2018	30271481	Tumor
chr19	36212696	2229	KMT2B	exonic	HIVID	Yang et al .2018	30271481	Tumor
chr19	36212890	359	KMT2B	intronic	HIVID	Yang et al .2018	30271481	Tumor
chr19	36212929	1784	KMT2B	intronic	HIVID	Yang et al .2018	30271481	Tumor
chr4	18774216	1782	LCORL(dist=750733),SLIT2(dist=1481019)	intergenic	HIVID	Yang et al .2018	30271481	Tumor
chr4	37604183	348	RELL1	intronic	HIVID	Yang et al .2018	30271481	Tumor
chr1	10058	2390	NONE(dist=NONE),DDX11L1(dist=1816)	intergenic	HIVID	Yang et al .2018	30271481	Tumor
chr1	10132	2390	NONE(dist=NONE),DDX11L1(dist=1742)	intergenic	HIVID	Yang et al .2018	30271481	Tumor
chr1	10297	2390	NONE(dist=NONE),DDX11L1(dist=1577)	intergenic	HIVID	Yang et al .2018	30271481	Tumor
chr1	10381	2390	NONE(dist=NONE),DDX11L1(dist=1493)	intergenic	HIVID	Yang et al .2018	30271481	Tumor
chr1	14023155	2408	PDPN(dist=78703),PRDM2(dist=3580)	intergenic	HIVID	Yang et al .2018	30271481	Tumor
chr1	95985542	2158	FLJ31662(dist=40630),PTBP2(dist=1201633)	intergenic	HIVID	Yang et al .2018	30271481	Tumor
chr1	121484859	1837	EMBP1(dist=171173),NONE(dist=NONE)	intergenic	HIVID	Yang et al .2018	30271481	Tumor
chr1	188626072	1822	PLA2G4A(dist=1667959),FAM5C(dist=1440725)	intergenic	HIVID	Yang et al .2018	30271481	Tumor
chr1	204114513	1640	ETNK2	intronic	HIVID	Yang et al .2018	30271481	Tumor
chr1	207140660	2152	FCAMR	intronic	HIVID	Yang et al .2018	30271481	Tumor
chr1	247267270	1703	ZNF669	exonic	HIVID	Yang et al .2018	30271481	Tumor
chr1	249239891	2389	PGBD2(dist=26546),NONE(dist=NONE)	intergenic	HIVID	Yang et al .2018	30271481	Tumor
chr1	249240051	2388	PGBD2(dist=26706),NONE(dist=NONE)	intergenic	HIVID	Yang et al .2018	30271481	Tumor
chr1	249240132	2390	PGBD2(dist=26787),NONE(dist=NONE)	intergenic	HIVID	Yang et al .2018	30271481	Tumor
chr1	249240220	2390	PGBD2(dist=26875),NONE(dist=NONE)	intergenic	HIVID	Yang et al .2018	30271481	Tumor
chr1	249240303	2329	PGBD2(dist=26958),NONE(dist=NONE)	intergenic	HIVID	Yang et al .2018	30271481	Tumor
chr1	249240384	2390	PGBD2(dist=27039),NONE(dist=NONE)	intergenic	HIVID	Yang et al .2018	30271481	Tumor
chr10	42391525	1671	NONE(dist=NONE),LOC441666(dist=435789)	intergenic	HIVID	Yang et al .2018	30271481	Tumor
chr10	88512743	1837	LDB3(dist=16919),BMPR1A(dist=3653)	intergenic	HIVID	Yang et al .2018	30271481	Tumor
chr10	88512748	2325	LDB3(dist=16924),BMPR1A(dist=3648)	intergenic	HIVID	Yang et al .2018	30271481	Tumor
chr10	88541379	234	BMPR1A	intronic	HIVID	Yang et al .2018	30271481	Tumor
chr10	135524466	2385	DUX2(dist=25974),NONE(dist=NONE)	intergenic	HIVID	Yang et al .2018	30271481	Tumor
chr11	175777	2390	LOC100133161(dist=43857),SCGB1C1(dist=17303)	intergenic	HIVID	Yang et al .2018	30271481	Tumor
chr11	14702850	1823	PDE3B	intronic	HIVID	Yang et al .2018	30271481	Tumor
chr11	30193847	1574	KCNA4(dist=155270),FSHB(dist=58716)	intergenic	HIVID	Yang et al .2018	30271481	Tumor
chr11	34382985	1103	ABTB2(dist=3430),CAT(dist=77487)	intergenic	HIVID	Yang et al .2018	30271481	Tumor
chr11	46132145	2803	PHF21A	intronic	HIVID	Yang et al .2018	30271481	Tumor
chr11	46132644	398	PHF21A	intronic	HIVID	Yang et al .2018	30271481	Tumor
chr11	46132935	1061	PHF21A	intronic	HIVID	Yang et al .2018	30271481	Tumor
chr11	94556414	1973	AMOTL1	intronic	HIVID	Yang et al .2018	30271481	Tumor
chr11	105842635	1828	GRIA4	intronic	HIVID	Yang et al .2018	30271481	Tumor
chr11	105842655	1765	GRIA4	exonic	HIVID	Yang et al .2018	30271481	Tumor
chr12	95162	2390	LOC100288778(dist=3899),FAM138D(dist=52784)	intergenic	HIVID	Yang et al .2018	30271481	Tumor
chr12	95360	2390	LOC100288778(dist=4097),FAM138D(dist=52586)	intergenic	HIVID	Yang et al .2018	30271481	Tumor
chr12	95433	2355	LOC100288778(dist=4170),FAM138D(dist=52513)	intergenic	HIVID	Yang et al .2018	30271481	Tumor
chr12	95505	2390	LOC100288778(dist=4242),FAM138D(dist=52441)	intergenic	HIVID	Yang et al .2018	30271481	Tumor
chr12	95587	2390	LOC100288778(dist=4324),FAM138D(dist=52359)	intergenic	HIVID	Yang et al .2018	30271481	Tumor
chr12	95661	2390	LOC100288778(dist=4398),FAM138D(dist=52285)	intergenic	HIVID	Yang et al .2018	30271481	Tumor
chr12	2250290	439	CACNA1C	intronic	HIVID	Yang et al .2018	30271481	Tumor
chr12	20691809	2713	PDE3A	intronic	HIVID	Yang et al .2018	30271481	Tumor
chr12	22999862	1936	ETNK1(dist=156254),SOX5(dist=685369)	intergenic	HIVID	Yang et al .2018	30271481	Tumor
chr12	27236556	2364	C12orf71(dist=1101),STK38L(dist=160522)	intergenic	HIVID	Yang et al .2018	30271481	Tumor
chr12	27236720	753	C12orf71(dist=1265),STK38L(dist=160358)	intergenic	HIVID	Yang et al .2018	30271481	Tumor
chr12	50261607	1263	FAIM2	UTR3	HIVID	Yang et al .2018	30271481	Tumor
chr12	66451372	446	HMGA2(dist=91301),LLPH(dist=65477)	intergenic	HIVID	Yang et al .2018	30271481	Tumor
chr12	88431764	2962	C12orf29	intronic	HIVID	Yang et al .2018	30271481	Tumor
chr12	108656694	2234	WSCD2(dist=12381),CMKLR1(dist=25127)	intergenic	HIVID	Yang et al .2018	30271481	Tumor
chr12	113319973	2189	RPH3A	intronic	HIVID	Yang et al .2018	30271481	Tumor
chr12	132435137	348	EP400	intronic	HIVID	Yang et al .2018	30271481	Tumor
chr12	133841523	2390	ANHX(dist=29101),NONE(dist=NONE)	intergenic	HIVID	Yang et al .2018	30271481	Tumor
chr12	133841626	2390	ANHX(dist=29204),NONE(dist=NONE)	intergenic	HIVID	Yang et al .2018	30271481	Tumor
chr12	133841751	2390	ANHX(dist=29329),NONE(dist=NONE)	intergenic	HIVID	Yang et al .2018	30271481	Tumor
chr12	133841835	2390	ANHX(dist=29413),NONE(dist=NONE)	intergenic	HIVID	Yang et al .2018	30271481	Tumor
chr13	48916909	554	RB1	intronic	HIVID	Yang et al .2018	30271481	Tumor
chr13	49171925	1823	LINC00462(dist=16888),CYSLTR2(dist=109026)	intergenic	HIVID	Yang et al .2018	30271481	Tumor
chr13	108124534	2703	FAM155A	intronic	HIVID	Yang et al .2018	30271481	Tumor
chr14	58722083	1611	PSMA3	intronic	HIVID	Yang et al .2018	30271481	Tumor
chr14	90098396	2004	FOXN3(dist=12902),EFCAB11(dist=165073)	intergenic	HIVID	Yang et al .2018	30271481	Tumor
chr14	90766405	508	C14orf102	intronic	HIVID	Yang et al .2018	30271481	Tumor
chr15	80755812	2390	ARNT2	intronic	HIVID	Yang et al .2018	30271481	Tumor
chr15	102521231	2390	DDX11L9(dist=1935),NONE(dist=NONE)	intergenic	HIVID	Yang et al .2018	30271481	Tumor
chr15	102521314	2390	DDX11L9(dist=2018),NONE(dist=NONE)	intergenic	HIVID	Yang et al .2018	30271481	Tumor
chr16	11342764	2881	CLEC16A(dist=66718),SOCS1(dist=5510)	intergenic	HIVID	Yang et al .2018	30271481	Tumor
chr16	11356934	1747	SOCS1(dist=6895),TNP2(dist=4780)	intergenic	HIVID	Yang et al .2018	30271481	Tumor
chr16	25771462	2390	HS3ST4	intronic	HIVID	Yang et al .2018	30271481	Tumor

Chromosome	Integration site in host genome	Integration site in virus genome	Gene (distance, bp)	Regions	Methods	Author	PMID	Sample
chr16	47795253	2018	PHKB(dist=59819),ABCC12(dist=321631)	intergenic	HIVID	Yang et al .2018	30271481	Tumor
chr16	75368094	1814	CFDP1	intronic	HIVID	Yang et al .2018	30271481	Tumor
chr16	76328148	395	CNTNAP4	intronic	HIVID	Yang et al .2018	30271481	Tumor
chr16	89160458	1188	ACSF3	intronic	HIVID	Yang et al .2018	30271481	Tumor
chr17	17345171	451	SMCR9(dist=18443),MED9(dist=35129)	intergenic	HIVID	Yang et al .2018	30271481	Tumor
chr18	63728	2390	NONE(dist=NONE),ROCK1P1(dist=45337)	intergenic	HIVID	Yang et al .2018	30271481	Tumor
chr18	23682635	677	SS18(dist=12024),PSMA8(dist=31181)	intergenic	HIVID	Yang et al .2018	30271481	Tumor
chr18	35050536	1904	CELF4	intronic	HIVID	Yang et al .2018	30271481	Tumor
chr18	62275757	451	LOC284294(dist=184930),CDH7(dist=1141731)	intergenic	HIVID	Yang et al .2018	30271481	Tumor
chr18	71132732	984	LOC100505817(dist=115608),FBXO15(dist=607856)	intergenic	HIVID	Yang et al .2018	30271481	Tumor
chr19	21788223	1823	ZNF429(dist=67144),ZNF100(dist=118620)	intergenic	HIVID	Yang et al .2018	30271481	Tumor
chr19	36212541	1548	KMT2B	exonic	HIVID	Yang et al .2018	30271481	Tumor
chr19	36212557	1805	KMT2B	exonic	HIVID	Yang et al .2018	30271481	Tumor
chr19	46758278	562	RNU6-66	ncRNA_intronic	HIVID	Yang et al .2018	30271481	Tumor
chr19	48606219	2379	PLA2G4C	intronic	HIVID	Yang et al .2018	30271481	Tumor
chr19	55013368	2702	LAIR2	upstream	HIVID	Yang et al .2018	30271481	Tumor
chr19	59118901	2360	MGC2752(dist=23139),NONE(dist=NONE)	intergenic	HIVID	Yang et al .2018	30271481	Tumor
chr2	27569026	2414	GTF3C2	intronic	HIVID	Yang et al .2018	30271481	Tumor
chr2	32040266	3157	SRD5A2(dist=234226),MEMO1(dist=52628)	intergenic	HIVID	Yang et al .2018	30271481	Tumor
chr2	98619009	2021	TMEM131(dist=6655),VWA3B(dist=84586)	intergenic	HIVID	Yang et al .2018	30271481	Tumor
chr2	99818030	1010	MRPL30(dist=2010),LYG2(dist=40681)	intergenic	HIVID	Yang et al .2018	30271481	Tumor
chr2	99818050	1793	MRPL30(dist=2030),LYG2(dist=40661)	intergenic	HIVID	Yang et al .2018	30271481	Tumor
chr2	112622362	236	ANAPC1	intronic	HIVID	Yang et al .2018	30271481	Tumor
chr2	177957993	608	LOC375295(dist=455691),HNRNPA3(dist=119429)	intergenic	HIVID	Yang et al .2018	30271481	Tumor
chr2	182140588	2390	UBE2E3(dist=212438),MIR4437(dist=29732)	intergenic	HIVID	Yang et al .2018	30271481	Tumor
chr2	198560350	451	RFTN2(dist=19766),MARS2(dist=9678)	intergenic	HIVID	Yang et al .2018	30271481	Tumor
chr2	243152479	2390	LOC728323(dist=50010),NONE(dist=NONE)	intergenic	HIVID	Yang et al .2018	30271481	Tumor
chr2	243152550	2390	LOC728323(dist=50081),NONE(dist=NONE)	intergenic	HIVID	Yang et al .2018	30271481	Tumor
chr2	243152624	2390	LOC728323(dist=50155),NONE(dist=NONE)	intergenic	HIVID	Yang et al .2018	30271481	Tumor
chr20	15072226	1731	MACROD2	intronic	HIVID	Yang et al .2018	30271481	Tumor
chr20	62918382	2390	PCMTD2(dist=10803),LINC00266-1(dist=3356)	intergenic	HIVID	Yang et al .2018	30271481	Tumor
chr21	40323718	563	ETS2(dist=126840),PSMG1(dist=223654)	intergenic	HIVID	Yang et al .2018	30271481	Tumor
chr21	48119792	2390	PRMT2(dist=34637),NONE(dist=NONE)	intergenic	HIVID	Yang et al .2018	30271481	Tumor
chr3	56939291	192	ARHGEF3	intronic	HIVID	Yang et al .2018	30271481	Tumor
chr3	62684570	2313	CADPS	intronic	HIVID	Yang et al .2018	30271481	Tumor
chr3	76645486	1207	ZNF717(dist=811231),ROBO2(dist=443808)	intergenic	HIVID	Yang et al .2018	30271481	Tumor
chr3	81256118	86	ROBO1(dist=1439059),GBE1(dist=282732)	intergenic	HIVID	Yang et al .2018	30271481	Tumor
chr3	130651061	1139	ATP2C1	intronic	HIVID	Yang et al .2018	30271481	Tumor
chr3	174080430	524	NLGN1(dist=79314),NAALADL2(dist=496681)	intergenic	HIVID	Yang et al .2018	30271481	Tumor
chr3	193152238	1782	ATP13A4	intronic	HIVID	Yang et al .2018	30271481	Tumor
chr4	10185	2390	NONE(dist=NONE),ZNF595(dist=43042)	intergenic	HIVID	Yang et al .2018	30271481	Tumor
chr4	6425625	2390	PPP2R2C	intronic	HIVID	Yang et al .2018	30271481	Tumor
chr4	9671023	1656	MIR548I2(dist=113086),DRD5(dist=112235)	intergenic	HIVID	Yang et al .2018	30271481	Tumor
chr4	10321937	2548	WDR1(dist=203364),ZNF518B(dist=119567)	intergenic	HIVID	Yang et al .2018	30271481	Tumor
chr4	18491815	598	LCORL(dist=468332),SLIT2(dist=1763420)	intergenic	HIVID	Yang et al .2018	30271481	Tumor
chr4	23211361	2026	MIR548AJ2	ncRNA_intronic	HIVID	Yang et al .2018	30271481	Tumor
chr4	32731316	1593	PCDH7(dist=1582893),NONE(dist=NONE)	intergenic	HIVID	Yang et al .2018	30271481	Tumor
chr4	56985044	1806	CEP135(dist=85515),KIAA1211(dist=51317)	intergenic	HIVID	Yang et al .2018	30271481	Tumor
chr4	118629313	2326	TRAM1L1(dist=622577),NDST3(dist=326187)	intergenic	HIVID	Yang et al .2018	30271481	Tumor
chr4	146927527	473	ZNF827(dist=67920),LOC100505545(dist=103080)	intergenic	HIVID	Yang et al .2018	30271481	Tumor
chr4	166602912	970	CPE(dist=183430),TLL1(dist=191498)	intergenic	HIVID	Yang et al .2018	30271481	Tumor
chr4	191043857	2390	DUX4L2(dist=30381),NONE(dist=NONE)	intergenic	HIVID	Yang et al .2018	30271481	Tumor
chr4	191043979	2390	DUX4L2(dist=30503),NONE(dist=NONE)	intergenic	HIVID	Yang et al .2018	30271481	Tumor
chr4	191044096	2390	DUX4L2(dist=30620),NONE(dist=NONE)	intergenic	HIVID	Yang et al .2018	30271481	Tumor
chr4	191044191	2390	DUX4L2(dist=30715),NONE(dist=NONE)	intergenic	HIVID	Yang et al .2018	30271481	Tumor
chr5	10067	2390	NONE(dist=NONE),PLEKHG4B(dist=130306)	intergenic	HIVID	Yang et al .2018	30271481	Tumor
chr5	10140	2390	NONE(dist=NONE),PLEKHG4B(dist=130233)	intergenic	HIVID	Yang et al .2018	30271481	Tumor
chr5	10211	2390	NONE(dist=NONE),PLEKHG4B(dist=130162)	intergenic	HIVID	Yang et al .2018	30271481	Tumor
chr5	10298	2390	NONE(dist=NONE),PLEKHG4B(dist=130075)	intergenic	HIVID	Yang et al .2018	30271481	Tumor
chr5	10451	2390	NONE(dist=NONE),PLEKHG4B(dist=129922)	intergenic	HIVID	Yang et al .2018	30271481	Tumor
chr5	10523	2390	NONE(dist=NONE),PLEKHG4B(dist=129850)	intergenic	HIVID	Yang et al .2018	30271481	Tumor
chr5	10793	2390	NONE(dist=NONE),PLEKHG4B(dist=129580)	intergenic	HIVID	Yang et al .2018	30271481	Tumor
chr5	10967	2390	NONE(dist=NONE),PLEKHG4B(dist=129406)	intergenic	HIVID	Yang et al .2018	30271481	Tumor
chr5	11061	2390	NONE(dist=NONE),PLEKHG4B(dist=129312)	intergenic	HIVID	Yang et al .2018	30271481	Tumor
chr5	11195	2390	NONE(dist=NONE),PLEKHG4B(dist=129178)	intergenic	HIVID	Yang et al .2018	30271481	Tumor
chr5	11341	2390	NONE(dist=NONE),PLEKHG4B(dist=129032)	intergenic	HIVID	Yang et al .2018	30271481	Tumor
chr5	11503	2390	NONE(dist=NONE),PLEKHG4B(dist=128870)	intergenic	HIVID	Yang et al .2018	30271481	Tumor
chr5	11585	2390	NONE(dist=NONE),PLEKHG4B(dist=128788)	intergenic	HIVID	Yang et al .2018	30271481	Tumor
chr5	11724	2390	NONE(dist=NONE),PLEKHG4B(dist=128649)	intergenic	HIVID	Yang et al .2018	30271481	Tumor
chr5	1295537	1742	TERT	upstream	HIVID	Yang et al .2018	30271481	Tumor
chr5	1295701	1813	TERT	upstream	HIVID	Yang et al .2018	30271481	Tumor
chr5	5913725	1848	KIAA0947(dist=423378),FLJ33360(dist=396829)	intergenic	HIVID	Yang et al .2018	30271481	Tumor
chr5	11282985	2416	CTNND2	intronic	HIVID	Yang et al .2018	30271481	Tumor
chr5	43600358	1961	LOC100652772	ncRNA_intronic	HIVID	Yang et al .2018	30271481	Tumor
chr5	57872031	800	GAPT(dist=79846),RAB3C(dist=6908)	intergenic	HIVID	Yang et al .2018	30271481	Tumor
chr5	64711643	2380	ADAMTS6	intronic	HIVID	Yang et al .2018	30271481	Tumor
chr5	77993759	2907	LHFPL2(dist=49111),ARSB(dist=79278)	intergenic	HIVID	Yang et al .2018	30271481	Tumor
chr5	86418346	2037	MIR4280(dist=7575),RASA1(dist=145724)	intergenic	HIVID	Yang et al .2018	30271481	Tumor
chr5	86979814	256	CCNH(dist=270964),TMEM161B(dist=511209)	intergenic	HIVID	Yang et al .2018	30271481	Tumor
chr5	143981058	2353	KCTD16(dist=124114),PRELID2(dist=1157524)	intergenic	HIVID	Yang et al .2018	30271481	Tumor
chr5	177279704	2048	FAM153A(dist=72199),LOC728554(dist=22558)	intergenic	HIVID	Yang et al .2018	30271481	Tumor
chr6	192044	40	NONE(dist=NONE),DUSP22(dist=100057)	intergenic	HIVID	Yang et al .2018	30271481	Tumor
chr6	37597935	2122	MIR4462(dist=74737),MDGA1(dist=2349)	intergenic	HIVID	Yang et al .2018	30271481	Tumor
chr6	38912793	2390	LOC100131047	ncRNA_intronic	HIVID	Yang et al .2018	30271481	Tumor
chr6	114229335	526	FLJ34503	ncRNA_intronic	HIVID	Yang et al .2018	30271481	Tumor
chr6	116977435	2018	ZUFSP	intronic	HIVID	Yang et al .2018	30271481	Tumor
chr7	10080	2390	NONE(dist=NONE),FAM20C(dist=182889)	intergenic	HIVID	Yang et al .2018	30271481	Tumor
chr7	10209	2390	NONE(dist=NONE),FAM20C(dist=182760)	intergenic	HIVID	Yang et al .2018	30271481	Tumor
chr7	434981	3171	LOC442497(dist=12136),PDGFA(dist=101916)	intergenic	HIVID	Yang et al .2018	30271481	Tumor
chr7	435233	146	LOC442497(dist=12388),PDGFA(dist=101664)	intergenic	HIVID	Yang et al .2018	30271481	Tumor
chr7	13823991	2390	ARL4A(dist=1093433),ETV1(dist=106865)	intergenic	HIVID	Yang et al .2018	30271481	Tumor
chr7	54748286	2903	LOC285878(dist=108867),SEC61G(dist=71654)	intergenic	HIVID	Yang et al .2018	30271481	Tumor
chr7	69802798	1973	AUTS2	intronic	HIVID	Yang et al .2018	30271481	Tumor
chr7	151386181	2166	PRKAG2	intronic	HIVID	Yang et al .2018	30271481	Tumor

Chromosome	Integration site in host genome	Integration site in virus genome	Gene (distance, bp)	Regions	Methods	Author	PMID	Sample
chr8	24611446	958	ADAM7(dist=244369),NEFM(dist=159828)	intergenic	HIVID	Yang et al .2018	30271481	Tumor
chr8	28326656	2018	FBXO16	intronic	HIVID	Yang et al .2018	30271481	Tumor
chr8	51787482	301	SNTG1(dist=82055),PXDNL(dist=444655)	intergenic	HIVID	Yang et al .2018	30271481	Tumor
chr8	56757525	1671	TGS1(dist=19520),LYN(dist=34861)	intergenic	HIVID	Yang et al .2018	30271481	Tumor
chr8	77793630	136	ZFHX4(dist=14109),PEX2(dist=98864)	intergenic	HIVID	Yang et al .2018	30271481	Tumor
chr9	9862162	1125	PTPRD	intronic	HIVID	Yang et al .2018	30271481	Tumor
chr9	33299172	2941	NFX1	intronic	HIVID	Yang et al .2018	30271481	Tumor
chr9	108151714	1837	SLC44A1	UTR3	HIVID	Yang et al .2018	30271481	Tumor
chr9	108258749	1135	FSD1L	intronic	HIVID	Yang et al .2018	30271481	Tumor
chr9	109851426	2229	MIR548Q(dist=2710),RAD23B(dist=194091)	intergenic	HIVID	Yang et al .2018	30271481	Tumor
chrX	5931750	724	NLGN4X	intronic	HIVID	Yang et al .2018	30271481	Tumor
chrX	61685241	486	NONE(dist=NONE),SPIN4(dist=881866)	intergenic	HIVID	Yang et al .2018	30271481	Tumor
chrX	62226276	593	NONE(dist=NONE),SPIN4(dist=340831)	intergenic	HIVID	Yang et al .2018	30271481	Tumor
chrX	118140820	125	LONRF3	intronic	HIVID	Yang et al .2018	30271481	Tumor
chrX	134778636	1739	DDX26B(dist=62176),CT45A1(dist=68549)	intergenic	HIVID	Yang et al .2018	30271481	Tumor
chrX	136297337	2701	GPR101(dist=183504),ZIC3(dist=351009)	intergenic	HIVID	Yang et al .2018	30271481	Tumor
chrX	155260024	2390	IL9R(dist=19542),NONE(dist=NONE)	intergenic	HIVID	Yang et al .2018	30271481	Tumor
chrX	155260144	2390	IL9R(dist=19662),NONE(dist=NONE)	intergenic	HIVID	Yang et al .2018	30271481	Tumor
chrX	155260216	2390	IL9R(dist=19734),NONE(dist=NONE)	intergenic	HIVID	Yang et al .2018	30271481	Tumor
chrX	155260348	2390	IL9R(dist=19866),NONE(dist=NONE)	intergenic	HIVID	Yang et al .2018	30271481	Tumor
chrY	59363048	2390	IL9R(dist=19560),NONE(dist=NONE)	intergenic	HIVID	Yang et al .2018	30271481	Tumor
chrY	59363138	2390	IL9R(dist=19650),NONE(dist=NONE)	intergenic	HIVID	Yang et al .2018	30271481	Tumor
chrY	59363210	2390	IL9R(dist=19722),NONE(dist=NONE)	intergenic	HIVID	Yang et al .2018	30271481	Tumor
chrY	59363282	2390	IL9R(dist=19794),NONE(dist=NONE)	intergenic	HIVID	Yang et al .2018	30271481	Tumor
chrY	59363287	1814	IL9R(dist=19799),NONE(dist=NONE)	intergenic	HIVID	Yang et al .2018	30271481	Tumor
chrY	59363354	2390	IL9R(dist=19866),NONE(dist=NONE)	intergenic	HIVID	Yang et al .2018	30271481	Tumor
chr10	110211	383	TUBB8(dist=15033),ZMYND11(dist=70194)	intergenic	HIVID	Yang et al .2018	30271481	Tumor
chr12	95433	383	LOC100288778(dist=4170),FAM138D(dist=52513)	intergenic	HIVID	Yang et al .2018	30271481	Tumor
chr12	27236556	2369	C12orf71(dist=1101),STK38L(dist=160522)	intergenic	HIVID	Yang et al .2018	30271481	Tumor
chr13	68377564	1345	PCDH9(dist=573096),LINC00550(dist=1057853)	intergenic	HIVID	Yang et al .2018	30271481	Tumor
chr15	91757981	536	SV2B	intronic	HIVID	Yang et al .2018	30271481	Tumor
chr17	19490636	2591	SLC47A1(dist=8290),ALDH3A2(dist=61428)	intergenic	HIVID	Yang et al .2018	30271481	Tumor
chr17	19494011	1818	SLC47A1(dist=11665),ALDH3A2(dist=58053)	intergenic	HIVID	Yang et al .2018	30271481	Tumor
chr18	28021	510	NONE(dist=NONE),ROCK1P1(dist=81044)	intergenic	HIVID	Yang et al .2018	30271481	Tumor
chr18	63875	383	NONE(dist=NONE),ROCK1P1(dist=45190)	intergenic	HIVID	Yang et al .2018	30271481	Tumor
chr19	18472690	1116	PGPEP1	intronic	HIVID	Yang et al .2018	30271481	Tumor
chr19	18472696	534	PGPEP1	intronic	HIVID	Yang et al .2018	30271481	Tumor
chr19	18472702	1042	PGPEP1	intronic	HIVID	Yang et al .2018	30271481	Tumor
chr19	18472702	1113	PGPEP1	intronic	HIVID	Yang et al .2018	30271481	Tumor
chr19	18634749	1674	ELL(dist=1812),FKBP8(dist=7819)	intergenic	HIVID	Yang et al .2018	30271481	Tumor
chr2	113669959	325	IL37	upstream	HIVID	Yang et al .2018	30271481	Tumor
chr2	114360987	383	DDX11L2	ncRNA_intronic	HIVID	Yang et al .2018	30271481	Tumor
chr2	174373351	653	CDCA7(dist=139633),SP3(dist=397836)	intergenic	HIVID	Yang et al .2018	30271481	Tumor
chr4	55722219	1769	KIT(dist=115338),KDR(dist=222207)	intergenic	HIVID	Yang et al .2018	30271481	Tumor
chr4	67589460	2649	LOC100144602(dist=1030356),CENPC1(dist=748529)	intergenic	HIVID	Yang et al .2018	30271481	Tumor
chr4	67590201	2075	LOC100144602(dist=1031097),CENPC1(dist=747788)	intergenic	HIVID	Yang et al .2018	30271481	Tumor
chr5	1295701	1813	TERT	upstream	HIVID	Yang et al .2018	30271481	Tumor
chr5	1296775	116	TERT(dist=1613),MIR4457(dist=12650)	intergenic	HIVID	Yang et al .2018	30271481	Tumor
chr6	154477153	1433	IPCEF1	UTR3	HIVID	Yang et al .2018	30271481	Tumor
chr6	163098968	1145	PARK2	intronic	HIVID	Yang et al .2018	30271481	Tumor
chr8	146301090	681	C8orf33(dist=19674),NONE(dist=NONE)	intergenic	HIVID	Yang et al .2018	30271481	Tumor
chr9	141054293	383	TUBBP5	ncRNA_intronic	HIVID	Yang et al .2018	30271481	Tumor
chrX	104182110	118	IL1RAPL2	intronic	HIVID	Yang et al .2018	30271481	Tumor
chr17	22254153	472	MTRNR2L1(dist=230162),NONE(dist=NONE)	intergenic	HIVID	Yang et al .2018	30271481	Tumor
chr17	22256501	646	MTRNR2L1(dist=232510),NONE(dist=NONE)	intergenic	HIVID	Yang et al .2018	30271481	Tumor
chr4	9671023	1707	MIR548I2(dist=113086),DRD5(dist=112235)	intergenic	HIVID	Yang et al .2018	30271481	Tumor
chr5	1286766	152	TERT	intronic	HIVID	Yang et al .2018	30271481	Tumor
chr5	1286871	2630	TERT	intronic	HIVID	Yang et al .2018	30271481	Tumor
chr7	92156773	807	PEX1	intronic	HIVID	Yang et al .2018	30271481	Tumor
chr8	51787482	301	SNTG1(dist=82055),PXDNL(dist=444655)	intergenic	HIVID	Yang et al .2018	30271481	Tumor
chr9	28517176	1955	LINGO2	intronic	HIVID	Yang et al .2018	30271481	Tumor
chr1	121484859	1837	EMBP1(dist=171173),NONE(dist=NONE)	intergenic	HIVID	Yang et al .2018	30271481	Tumor
chr1	121485035	2876	EMBP1(dist=171349),NONE(dist=NONE)	intergenic	HIVID	Yang et al .2018	30271481	Tumor
chr1	167854971	2513	ADCY10	intronic	HIVID	Yang et al .2018	30271481	Tumor
chr1	214893080	2511	CENPF(dist=55166),KCNK2(dist=285805)	intergenic	HIVID	Yang et al .2018	30271481	Tumor
chr10	36382467	1576	FZD8(dist=452105),ANKRD30A(dist=1032318)	intergenic	HIVID	Yang et al .2018	30271481	Tumor
chr10	53089051	2985	PRKG1	intronic	HIVID	Yang et al .2018	30271481	Tumor
chr10	88512743	1837	LDB3(dist=16919),BMPR1A(dist=3653)	intergenic	HIVID	Yang et al .2018	30271481	Tumor
chr11	117838931	2511	TMPRSS13(dist=38763),IL10RA(dist=18175)	intergenic	HIVID	Yang et al .2018	30271481	Tumor
chr12	111056000	2511	TCTN1	intronic	HIVID	Yang et al .2018	30271481	Tumor
chr13	68377562	1349	PCDH9(dist=573094),LINC00550(dist=1057855)	intergenic	HIVID	Yang et al .2018	30271481	Tumor
chr13	81435702	2511	SPRY2(dist=520616),NONE(dist=NONE)	intergenic	HIVID	Yang et al .2018	30271481	Tumor
chr14	38351463	421	FOXA1(dist=287138),SSTR1(dist=325741)	intergenic	HIVID	Yang et al .2018	30271481	Tumor
chr15	54065789	2511	WDR72(dist=13930),UNC13C(dist=239312)	intergenic	HIVID	Yang et al .2018	30271481	Tumor
chr15	75726887	2511	SIN3A	intronic	HIVID	Yang et al .2018	30271481	Tumor
chr15	86173133	2511	AKAP13	intronic	HIVID	Yang et al .2018	30271481	Tumor
chr15	101496792	2511	LRRK1	intronic	HIVID	Yang et al .2018	30271481	Tumor
chr17	12388503	1819	MAP2K4(dist=341452),LINC00670(dist=64782)	intergenic	HIVID	Yang et al .2018	30271481	Tumor
chr17	17102225	2511	MPRIP(dist=6263),PLD6(dist=2084)	intergenic	HIVID	Yang et al .2018	30271481	Tumor
chr17	19490799	2511	SLC47A1(dist=8453),ALDH3A2(dist=61265)	intergenic	HIVID	Yang et al .2018	30271481	Tumor
chr17	19493935	1818	SLC47A1(dist=11589),ALDH3A2(dist=58129)	intergenic	HIVID	Yang et al .2018	30271481	Tumor
chr17	19494006	1818	SLC47A1(dist=11660),ALDH3A2(dist=58058)	intergenic	HIVID	Yang et al .2018	30271481	Tumor
chr17	65878073	2511	BPTF	intronic	HIVID	Yang et al .2018	30271481	Tumor
chr19	15243496	2511	ILVBL(dist=6919),NOTCH3(dist=26948)	intergenic	HIVID	Yang et al .2018	30271481	Tumor
chr19	36212555	2179	KMT2B	exonic	HIVID	Yang et al .2018	30271481	Tumor
chr19	36213045	1826	KMT2B	intronic	HIVID	Yang et al .2018	30271481	Tumor
chr19	36967104	2511	ZNF566	intronic	HIVID	Yang et al .2018	30271481	Tumor
chr19	49390391	2511	TULP2	intronic	HIVID	Yang et al .2018	30271481	Tumor
chr2	120249210	2511	SCTR	intronic	HIVID	Yang et al .2018	30271481	Tumor
chr20	1182406	2511	TMEM74B(dist=17289),C20orf202(dist=1692)	intergenic	HIVID	Yang et al .2018	30271481	Tumor
chr21	21668275	2511	TMPRSS15(dist=1892305),LINC00320(dist=446638)	intergenic	HIVID	Yang et al .2018	30271481	Tumor
chr21	44516909	2511	U2AF1	intronic	HIVID	Yang et al .2018	30271481	Tumor
chr22	17921108	1078	CECR3(dist=173485),CECR2(dist=35520)	intergenic	HIVID	Yang et al .2018	30271481	Tumor
chr22	18505610	2959	MICAL3	intronic	HIVID	Yang et al .2018	30271481	Tumor

Chromosome	Integration site in host genome	Integration site in virus genome	Gene (distance, bp)	Regions	Methods	Author	PMID	Sample
chr3	58356622	2511	PXK	intronic	HIVID	Yang et al .2018	30271481	Tumor
chr4	55722014	2876	KIT(dist=115133),KDR(dist=222412)	intergenic	HIVID	Yang et al .2018	30271481	Tumor
chr4	55722215	1820	KIT(dist=115334),KDR(dist=222211)	intergenic	HIVID	Yang et al .2018	30271481	Tumor
chr4	67589454	2649	LOC100144602(dist=1030350),CENPC1(dist=748535)	intergenic	HIVID	Yang et al .2018	30271481	Tumor
chr4	67590191	2075	LOC100144602(dist=1031087),CENPC1(dist=747798)	intergenic	HIVID	Yang et al .2018	30271481	Tumor
chr4	174101601	2511	GALNT7	intronic	HIVID	Yang et al .2018	30271481	Tumor
chr5	1286766	152	TERT	intronic	HIVID	Yang et al .2018	30271481	Tumor
chr5	1286871	2630	TERT	intronic	HIVID	Yang et al .2018	30271481	Tumor
chr5	1295538	1741	TERT	upstream	HIVID	Yang et al .2018	30271481	Tumor
chr5	1296828	116	TERT(dist=1666),MIR4457(dist=12597)	intergenic	HIVID	Yang et al .2018	30271481	Tumor
chr5	1296970	564	TERT(dist=1808),MIR4457(dist=12455)	intergenic	HIVID	Yang et al .2018	30271481	Tumor
chr5	1296997	138	TERT(dist=1835),MIR4457(dist=12428)	intergenic	HIVID	Yang et al .2018	30271481	Tumor
chr5	134767201	1826	H2AFY(dist=31624),C5orf20(dist=12703)	intergenic	HIVID	Yang et al .2018	30271481	Tumor
chr5	170630378	2420	RANBP17	intronic	HIVID	Yang et al .2018	30271481	Tumor
chr6	37009269	2511	FGD2(dist=12424),PIM1(dist=128653)	intergenic	HIVID	Yang et al .2018	30271481	Tumor
chr6	58779434	2261	GUSBP4(dist=491710),NONE(dist=NONE)	intergenic	HIVID	Yang et al .2018	30271481	Tumor
chr6	128169222	421	THEMIS	intronic	HIVID	Yang et al .2018	30271481	Tumor
chr7	77306365	2511	PTPN12(dist=36977),RSBN1L-AS1(dist=6803)	intergenic	HIVID	Yang et al .2018	30271481	Tumor
chr7	92156773	807	PEX1	intronic	HIVID	Yang et al .2018	30271481	Tumor
chr7	101397113	2511	MYL10(dist=124537),CUX1(dist=62071)	intergenic	HIVID	Yang et al .2018	30271481	Tumor
chr7	128143948	2511	METTL2B	downstream	HIVID	Yang et al .2018	30271481	Tumor
chr8	51787482	301	SNTG1(dist=82055),PXDNL(dist=444655)	intergenic	HIVID	Yang et al .2018	30271481	Tumor
chr9	29769211	2226	LINGO2(dist=556213),LOC401497(dist=619722)	intergenic	HIVID	Yang et al .2018	30271481	Tumor
chrX	69439474	1587	DGAT2L6(dist=13921),AWAT1(dist=15031)	intergenic	HIVID	Yang et al .2018	30271481	Tumor
chrX	119045213	2511	AKAP14	intronic	HIVID	Yang et al .2018	30271481	Tumor
chrX	129225299	2511	ELF4	intronic	HIVID	Yang et al .2018	30271481	Tumor
chrY	58822105	2402	NONE(dist=NONE),SPRY3(dist=278352)	intergenic	HIVID	Yang et al .2018	30271481	Tumor
chrY	58825663	2402	NONE(dist=NONE),SPRY3(dist=274794)	intergenic	HIVID	Yang et al .2018	30271481	Tumor
chrY	58829227	2402	NONE(dist=NONE),SPRY3(dist=271230)	intergenic	HIVID	Yang et al .2018	30271481	Tumor
chrY	58832816	2402	NONE(dist=NONE),SPRY3(dist=267641)	intergenic	HIVID	Yang et al .2018	30271481	Tumor
chrY	58836350	2402	NONE(dist=NONE),SPRY3(dist=264107)	intergenic	HIVID	Yang et al .2018	30271481	Tumor
chrY	58837843	2802	NONE(dist=NONE),SPRY3(dist=262614)	intergenic	HIVID	Yang et al .2018	30271481	Tumor
chrY	58839824	2402	NONE(dist=NONE),SPRY3(dist=260633)	intergenic	HIVID	Yang et al .2018	30271481	Tumor
chrY	58857693	2400	NONE(dist=NONE),SPRY3(dist=242764)	intergenic	HIVID	Yang et al .2018	30271481	Tumor
chrY	58861269	2402	NONE(dist=NONE),SPRY3(dist=239188)	intergenic	HIVID	Yang et al .2018	30271481	Tumor
chrY	58866399	2823	NONE(dist=NONE),SPRY3(dist=234058)	intergenic	HIVID	Yang et al .2018	30271481	Tumor
chrY	58872019	2402	NONE(dist=NONE),SPRY3(dist=228438)	intergenic	HIVID	Yang et al .2018	30271481	Tumor
chrY	58873542	2803	NONE(dist=NONE),SPRY3(dist=226915)	intergenic	HIVID	Yang et al .2018	30271481	Tumor
chrY	58875563	2402	NONE(dist=NONE),SPRY3(dist=224894)	intergenic	HIVID	Yang et al .2018	30271481	Tumor
chrY	58891352	2823	NONE(dist=NONE),SPRY3(dist=209105)	intergenic	HIVID	Yang et al .2018	30271481	Tumor
chrY	58893398	2402	NONE(dist=NONE),SPRY3(dist=207059)	intergenic	HIVID	Yang et al .2018	30271481	Tumor
chrY	58898490	2823	NONE(dist=NONE),SPRY3(dist=201967)	intergenic	HIVID	Yang et al .2018	30271481	Tumor
chrY	58916810	2402	NONE(dist=NONE),SPRY3(dist=183647)	intergenic	HIVID	Yang et al .2018	30271481	Tumor
chr1	31529482	1765	PUM1	intronic	HIVID	Yang et al .2018	30271481	Tumor
chr14	41101498	2311	FBXO33(dist=1199794),LRFN5(dist=975266)	intergenic	HIVID	Yang et al .2018	30271481	Tumor
chr14	41462121	2888	FBXO33(dist=1560417),LRFN5(dist=614643)	intergenic	HIVID	Yang et al .2018	30271481	Tumor
chr15	49676946	636	FAM227B	intronic	HIVID	Yang et al .2018	30271481	Tumor
chr17	11366196	1752	SHISA6	intronic	HIVID	Yang et al .2018	30271481	Tumor
chr20	26188865	1815	MIR663A	ncRNA_exonic	HIVID	Yang et al .2018	30271481	Tumor
chr5	1295801	506	TERT	upstream	HIVID	Yang et al .2018	30271481	Tumor
chr5	1295801	1897	TERT	upstream	HIVID	Yang et al .2018	30271481	Tumor
chr5	32416121	1765	ZFR	intronic	HIVID	Yang et al .2018	30271481	Tumor
chr1	209819207	1606	LAMB3	intronic	HIVID	Yang et al .2018	30271481	Tumor
chr11	80228228	1314	ODZ4(dist=1076533),MIR4300(dist=1373555)	intergenic	HIVID	Yang et al .2018	30271481	Tumor
chr11	80228231	636	ODZ4(dist=1076536),MIR4300(dist=1373552)	intergenic	HIVID	Yang et al .2018	30271481	Tumor
chr14	99038674	1818	C14orf64(dist=594213),C14orf177(dist=139276)	intergenic	HIVID	Yang et al .2018	30271481	Tumor
chr15	53655145	1948	ONECUT1(dist=572936),WDR72(dist=150793)	intergenic	HIVID	Yang et al .2018	30271481	Tumor
chr17	2638923	1835	KIAA0664(dist=23996),MIR1253(dist=12449)	intergenic	HIVID	Yang et al .2018	30271481	Tumor
chr17	21365046	1765	KCNJ12(dist=41867),C17orf51(dist=66525)	intergenic	HIVID	Yang et al .2018	30271481	Tumor
chr17	38239834	573	THRA	intronic	HIVID	Yang et al .2018	30271481	Tumor
chr20	49109504	1835	LOC284751(dist=178048),PTPN1(dist=17387)	intergenic	HIVID	Yang et al .2018	30271481	Tumor
chr20	49118602	865	LOC284751(dist=187146),PTPN1(dist=8289)	intergenic	HIVID	Yang et al .2018	30271481	Tumor
chr3	66604323	1775	LRIG1(dist=53478),KBTBD8(dist=444404)	intergenic	HIVID	Yang et al .2018	30271481	Tumor
chr5	1296403	682	TERT(dist=1241),MIR4457(dist=13022)	intergenic	HIVID	Yang et al .2018	30271481	Tumor
chr5	74971395	2510	POC5	intronic	HIVID	Yang et al .2018	30271481	Tumor
chr5	176539895	1765	FGFR4(dist=14769),NSD1(dist=20185)	intergenic	HIVID	Yang et al .2018	30271481	Tumor
chr8	97429197	1830	PTDSS1(dist=82423),SDC2(dist=76685)	intergenic	HIVID	Yang et al .2018	30271481	Tumor
chr8	97514694	1819	SDC2	intronic	HIVID	Yang et al .2018	30271481	Tumor
chr1	45313075	968	PTCH2(dist=4459),EIF2B3(dist=3119)	intergenic	HIVID	Yang et al .2018	30271481	Tumor
chr1	121485035	2876	EMBP1(dist=171349),NONE(dist=NONE)	intergenic	HIVID	Yang et al .2018	30271481	Tumor
chr1	142890392	700	ANKRD20A12P(dist=176787),LOC100130000(dist=796738)	intergenic	HIVID	Yang et al .2018	30271481	Tumor
chr1	185244982	1637	SWT1	intronic	HIVID	Yang et al .2018	30271481	Tumor
chr1	224527983	707	NVL(dist=10092),CNIH4(dist=16612)	intergenic	HIVID	Yang et al .2018	30271481	Tumor
chr10	42385047	1304	NONE(dist=NONE),LOC441666(dist=442267)	intergenic	HIVID	Yang et al .2018	30271481	Tumor
chr10	42385534	958	NONE(dist=NONE),LOC441666(dist=441780)	intergenic	HIVID	Yang et al .2018	30271481	Tumor
chr10	42387255	958	NONE(dist=NONE),LOC441666(dist=440059)	intergenic	HIVID	Yang et al .2018	30271481	Tumor
chr10	42388428	1312	NONE(dist=NONE),LOC441666(dist=438886)	intergenic	HIVID	Yang et al .2018	30271481	Tumor
chr10	42389388	1007	NONE(dist=NONE),LOC441666(dist=437926)	intergenic	HIVID	Yang et al .2018	30271481	Tumor
chr10	42391101	958	NONE(dist=NONE),LOC441666(dist=436213)	intergenic	HIVID	Yang et al .2018	30271481	Tumor
chr10	42392442	1312	NONE(dist=NONE),LOC441666(dist=434872)	intergenic	HIVID	Yang et al .2018	30271481	Tumor
chr10	42393774	1312	NONE(dist=NONE),LOC441666(dist=433540)	intergenic	HIVID	Yang et al .2018	30271481	Tumor
chr10	42395692	1312	NONE(dist=NONE),LOC441666(dist=431622)	intergenic	HIVID	Yang et al .2018	30271481	Tumor
chr10	42396831	1222	NONE(dist=NONE),LOC441666(dist=430483)	intergenic	HIVID	Yang et al .2018	30271481	Tumor
chr10	42399351	3182	NONE(dist=NONE),LOC441666(dist=427963)	intergenic	HIVID	Yang et al .2018	30271481	Tumor
chr10	42400197	3182	NONE(dist=NONE),LOC441666(dist=427117)	intergenic	HIVID	Yang et al .2018	30271481	Tumor
chr10	42400644	4	NONE(dist=NONE),LOC441666(dist=426670)	intergenic	HIVID	Yang et al .2018	30271481	Tumor
chr10	42400704	3026	NONE(dist=NONE),LOC441666(dist=426610)	intergenic	HIVID	Yang et al .2018	30271481	Tumor
chr10	42400726	4	NONE(dist=NONE),LOC441666(dist=426588)	intergenic	HIVID	Yang et al .2018	30271481	Tumor
chr10	42526624	707	NONE(dist=NONE),LOC441666(dist=300690)	intergenic	HIVID	Yang et al .2018	30271481	Tumor
chr10	42527377	4	NONE(dist=NONE),LOC441666(dist=299937)	intergenic	HIVID	Yang et al .2018	30271481	Tumor
chr10	42527566	805	NONE(dist=NONE),LOC441666(dist=299748)	intergenic	HIVID	Yang et al .2018	30271481	Tumor
chr10	42538586	4	NONE(dist=NONE),LOC441666(dist=288728)	intergenic	HIVID	Yang et al .2018	30271481	Tumor
chr10	42541644	4	NONE(dist=NONE),LOC441666(dist=285670)	intergenic	HIVID	Yang et al .2018	30271481	Tumor
chr10	42544702	4	NONE(dist=NONE),LOC441666(dist=282612)	intergenic	HIVID	Yang et al .2018	30271481	Tumor

Chromosome	Integration site in host genome	Integration site in virus genome	Gene (distance, bp)	Regions	Methods	Author	PMID	Sample
chr10	42598039	972	NONE(dist=NONE),LOC441666(dist=229275)	intergenic	HIVID	Yang et al .2018	30271481	Tumor
chr10	42600390	1312	NONE(dist=NONE),LOC441666(dist=226924)	intergenic	HIVID	Yang et al .2018	30271481	Tumor
chr10	70492708	1803	CCAR1	intronic	HIVID	Yang et al .2018	30271481	Tumor
chr10	70492716	2340	CCAR1	intronic	HIVID	Yang et al .2018	30271481	Tumor
chr11	24649990	2496	LUZP2	intronic	HIVID	Yang et al .2018	30271481	Tumor
chr11	24654658	1840	LUZP2	intronic	HIVID	Yang et al .2018	30271481	Tumor
chr11	33092890	354	TCP11L1	intronic	HIVID	Yang et al .2018	30271481	Tumor
chr11	76492586	1828	TSKU	upstream	HIVID	Yang et al .2018	30271481	Tumor
chr12	27236556	2364	C12orf71(dist=1101),STK38L(dist=160522)	intergenic	HIVID	Yang et al .2018	30271481	Tumor
chr14	80728588	1828	DIO2-AS1	ncRNA_intronic	HIVID	Yang et al .2018	30271481	Tumor
chr14	80728588	2582	DIO2-AS1	ncRNA_intronic	HIVID	Yang et al .2018	30271481	Tumor
chr14	84359569	969	NONE(dist=NONE),FLRT2(dist=1636919)	intergenic	HIVID	Yang et al .2018	30271481	Tumor
chr14	84359582	1562	NONE(dist=NONE),FLRT2(dist=1636906)	intergenic	HIVID	Yang et al .2018	30271481	Tumor
chr15	35659215	1637	ANP32AP1(dist=128951),ATPBD4(dist=3955)	intergenic	HIVID	Yang et al .2018	30271481	Tumor
chr15	50386657	638	ATP8B4	intronic	HIVID	Yang et al .2018	30271481	Tumor
chr16	5563260	2812	FAM86A(dist=415471),RBFOX1(dist=505872)	intergenic	HIVID	Yang et al .2018	30271481	Tumor
chr16	47898814	707	PHKB(dist=163380),ABCC12(dist=218070)	intergenic	HIVID	Yang et al .2018	30271481	Tumor
chr16	88812032	1826	PIEZO1	intronic	HIVID	Yang et al .2018	30271481	Tumor
chr19	36213045	1826	KMT2B	intronic	HIVID	Yang et al .2018	30271481	Tumor
chr2	204301398	1828	RAPH1	UTR3	HIVID	Yang et al .2018	30271481	Tumor
chr20	49118504	629	LOC284751(dist=187048),PTPN1(dist=8387)	intergenic	HIVID	Yang et al .2018	30271481	Tumor
chr21	10164472	704	TEKT4P2(dist=195879),TPTE(dist=742271)	intergenic	HIVID	Yang et al .2018	30271481	Tumor
chr22	42443115	2204	WBP2NL(dist=18638),NAGA(dist=11223)	intergenic	HIVID	Yang et al .2018	30271481	Tumor
chr3	16144581	2646	MIR563(dist=229225),GALNTL2(dist=71603)	intergenic	HIVID	Yang et al .2018	30271481	Tumor
chr3	16148514	1563	MIR563(dist=233158),GALNTL2(dist=67670)	intergenic	HIVID	Yang et al .2018	30271481	Tumor
chr3	54012109	707	SELK(dist=86120),CACNA2D3(dist=144584)	intergenic	HIVID	Yang et al .2018	30271481	Tumor
chr3	59391280	2471	C3orf67(dist=355565),FHIT(dist=343756)	intergenic	HIVID	Yang et al .2018	30271481	Tumor
chr4	67590201	2075	LOC100144602(dist=1031097),CENPC1(dist=747788)	intergenic	HIVID	Yang et al .2018	30271481	Tumor
chr4	68264799	2786	LOC100144602(dist=1705695),CENPC1(dist=73190)	intergenic	HIVID	Yang et al .2018	30271481	Tumor
chr4	68265857	2795	LOC100144602(dist=1706753),CENPC1(dist=72132)	intergenic	HIVID	Yang et al .2018	30271481	Tumor
chr5	80886413	1821	SSBP2	intronic	HIVID	Yang et al .2018	30271481	Tumor
chr5	80892866	1806	SSBP2	intronic	HIVID	Yang et al .2018	30271481	Tumor
chr5	156085135	707	SGCD	intronic	HIVID	Yang et al .2018	30271481	Tumor
chr6	78435692	707	HTR1B(dist=262572),IRAK1BP1(dist=1141497)	intergenic	HIVID	Yang et al .2018	30271481	Tumor
chr7	16237791	707	ISPD	intronic	HIVID	Yang et al .2018	30271481	Tumor
chr8	51787482	301	SNTG1(dist=82055),PXDNL(dist=444655)	intergenic	HIVID	Yang et al .2018	30271481	Tumor
chr8	144370022	707	GLI4(dist=10921),ZNF696(dist=3537)	intergenic	HIVID	Yang et al .2018	30271481	Tumor
chr9	106256997	1802	CYLC2(dist=476227),SMC2(dist=599544)	intergenic	HIVID	Yang et al .2018	30271481	Tumor
chr9	106257025	2591	CYLC2(dist=476255),SMC2(dist=599516)	intergenic	HIVID	Yang et al .2018	30271481	Tumor
chrX	134778636	1739	DDX26B(dist=62176),CT45A1(dist=68549)	intergenic	HIVID	Yang et al .2018	30271481	Tumor
chrX	144802927	707	SPANXN1(dist=465199),SLITRK2(dist=96420)	intergenic	HIVID	Yang et al .2018	30271481	Tumor
chrY	58822105	2402	NONE(dist=NONE),SPRY3(dist=278352)	intergenic	HIVID	Yang et al .2018	30271481	Tumor
chrY	58825663	2402	NONE(dist=NONE),SPRY3(dist=274794)	intergenic	HIVID	Yang et al .2018	30271481	Tumor
chrY	58839824	2402	NONE(dist=NONE),SPRY3(dist=260633)	intergenic	HIVID	Yang et al .2018	30271481	Tumor
chrY	58846978	2402	NONE(dist=NONE),SPRY3(dist=253479)	intergenic	HIVID	Yang et al .2018	30271481	Tumor
chrY	58850572	2402	NONE(dist=NONE),SPRY3(dist=249885)	intergenic	HIVID	Yang et al .2018	30271481	Tumor
chrY	58857695	2402	NONE(dist=NONE),SPRY3(dist=242762)	intergenic	HIVID	Yang et al .2018	30271481	Tumor
chrY	58872019	2402	NONE(dist=NONE),SPRY3(dist=228438)	intergenic	HIVID	Yang et al .2018	30271481	Tumor
chrY	58875563	2402	NONE(dist=NONE),SPRY3(dist=224894)	intergenic	HIVID	Yang et al .2018	30271481	Tumor
chrY	58879152	2402	NONE(dist=NONE),SPRY3(dist=221305)	intergenic	HIVID	Yang et al .2018	30271481	Tumor
chrY	58882706	2402	NONE(dist=NONE),SPRY3(dist=217751)	intergenic	HIVID	Yang et al .2018	30271481	Tumor
chrY	58889819	2402	NONE(dist=NONE),SPRY3(dist=210638)	intergenic	HIVID	Yang et al .2018	30271481	Tumor
chrY	58893398	2402	NONE(dist=NONE),SPRY3(dist=207059)	intergenic	HIVID	Yang et al .2018	30271481	Tumor
chrY	58902025	2793	NONE(dist=NONE),SPRY3(dist=198432)	intergenic	HIVID	Yang et al .2018	30271481	Tumor
chrY	58904100	2402	NONE(dist=NONE),SPRY3(dist=196357)	intergenic	HIVID	Yang et al .2018	30271481	Tumor
chrY	58911257	2402	NONE(dist=NONE),SPRY3(dist=189200)	intergenic	HIVID	Yang et al .2018	30271481	Tumor
chrY	58916810	2402	NONE(dist=NONE),SPRY3(dist=183647)	intergenic	HIVID	Yang et al .2018	30271481	Tumor
chr10	42369007	2345	NONE(dist=NONE),LOC441666(dist=458307)	intergenic	HIVID	Yang et al .2018	30271481	Tumor
chr10	103106759	1828	FLJ41350(dist=108143),BTRC(dist=7031)	intergenic	HIVID	Yang et al .2018	30271481	Tumor
chr13	78145584	1828	SCEL	intronic	HIVID	Yang et al .2018	30271481	Tumor
chr19	36212668	2266	KMT2B	exonic	HIVID	Yang et al .2018	30271481	Tumor
chr19	36212696	2220	KMT2B	exonic	HIVID	Yang et al .2018	30271481	Tumor
chr2	198383740	1828	HSPE1-MOB4,MOB4	intronic	HIVID	Yang et al .2018	30271481	Tumor
chr20	33261921	1828	PIGU	intronic	HIVID	Yang et al .2018	30271481	Tumor
chr20	52919750	1828	PFDN4(dist=83258),DOK5(dist=172516)	intergenic	HIVID	Yang et al .2018	30271481	Tumor
chr21	11028652	1828	BAGE2,BAGE3,BAGE4,BAGE5	intronic	HIVID	Yang et al .2018	30271481	Tumor
chr4	49103948	2345	CWH43(dist=39853),NONE(dist=NONE)	intergenic	HIVID	Yang et al .2018	30271481	Tumor
chr4	49104004	1765	CWH43(dist=39909),NONE(dist=NONE)	intergenic	HIVID	Yang et al .2018	30271481	Tumor
chr4	49104374	3093	CWH43(dist=40279),NONE(dist=NONE)	intergenic	HIVID	Yang et al .2018	30271481	Tumor
chr4	49109732	2345	CWH43(dist=45637),NONE(dist=NONE)	intergenic	HIVID	Yang et al .2018	30271481	Tumor
chr4	49109787	1765	CWH43(dist=45692),NONE(dist=NONE)	intergenic	HIVID	Yang et al .2018	30271481	Tumor
chr4	49109846	2345	CWH43(dist=45751),NONE(dist=NONE)	intergenic	HIVID	Yang et al .2018	30271481	Tumor
chr4	49111144	2345	CWH43(dist=47049),NONE(dist=NONE)	intergenic	HIVID	Yang et al .2018	30271481	Tumor
chr4	49111200	1765	CWH43(dist=47105),NONE(dist=NONE)	intergenic	HIVID	Yang et al .2018	30271481	Tumor
chr4	49111593	3093	CWH43(dist=47498),NONE(dist=NONE)	intergenic	HIVID	Yang et al .2018	30271481	Tumor
chr4	49117573	3093	CWH43(dist=53478),NONE(dist=NONE)	intergenic	HIVID	Yang et al .2018	30271481	Tumor
chr4	49122066	3063	CWH43(dist=57971),NONE(dist=NONE)	intergenic	HIVID	Yang et al .2018	30271481	Tumor
chr4	49122089	1765	CWH43(dist=57994),NONE(dist=NONE)	intergenic	HIVID	Yang et al .2018	30271481	Tumor
chr4	49133721	3093	CWH43(dist=69626),NONE(dist=NONE)	intergenic	HIVID	Yang et al .2018	30271481	Tumor
chr4	49134093	3093	CWH43(dist=69998),NONE(dist=NONE)	intergenic	HIVID	Yang et al .2018	30271481	Tumor
chr4	49648038	3093	CWH43(dist=583943),NONE(dist=NONE)	intergenic	HIVID	Yang et al .2018	30271481	Tumor
chr4	103762567	1765	UBE2D3	intronic	HIVID	Yang et al .2018	30271481	Tumor
chr4	103762601	2055	UBE2D3	intronic	HIVID	Yang et al .2018	30271481	Tumor
chr8	141532032	3020	CHRAC1(dist=4780),EIF2C2(dist=9232)	intergenic	HIVID	Yang et al .2018	30271481	Tumor
chr8	141532035	1612	CHRAC1(dist=4783),EIF2C2(dist=9229)	intergenic	HIVID	Yang et al .2018	30271481	Tumor
chr9	90879933	1695	FAM75C2(dist=130033),SPIN1(dist=123364)	intergenic	HIVID	Yang et al .2018	30271481	Tumor
chr9	90879986	2530	FAM75C2(dist=130086),SPIN1(dist=123311)	intergenic	HIVID	Yang et al .2018	30271481	Tumor
chr10	44022671	876	ZNF487P(dist=44664),ZNF239(dist=29122)	intergenic	HIVID	Yang et al .2018	30271481	Tumor
chr11	5152909	1825	OR52A5	downstream	HIVID	Yang et al .2018	30271481	Tumor
chr11	5152963	1809	OR52A5	exonic	HIVID	Yang et al .2018	30271481	Tumor
chr11	55026411	2847	NONE(dist=NONE),TRIM48(dist=3247)	intergenic	HIVID	Yang et al .2018	30271481	Tumor
chr18	49758819	755	LOC100287225(dist=669980),DCC(dist=107723)	intergenic	HIVID	Yang et al .2018	30271481	Tumor
chr18	49767356	1395	LOC100287225(dist=678517),DCC(dist=99186)	intergenic	HIVID	Yang et al .2018	30271481	Tumor
chr19	27737931	1221	NONE(dist=NONE),LINC00662(dist=543470)	intergenic	HIVID	Yang et al .2018	30271481	Tumor

Chromosome	Integration site in host genome	Integration site in virus genome	Gene (distance, bp)	Regions	Methods	Author	PMID	Sample
chr2	34993597	876	MYADML(dist=1040313),LOC100288911(dist=1588295)	intergenic	HIVID	Yang et al .2018	30271481	Tumor
chr20	56139933	115	PCK1	intronic	HIVID	Yang et al .2018	30271481	Tumor
chr20	56139936	1485	PCK1	intronic	HIVID	Yang et al .2018	30271481	Tumor
chr3	23071964	876	ZNF385D(dist=1279148),UBE2E2(dist=172820)	intergenic	HIVID	Yang et al .2018	30271481	Tumor
chr3	157509543	1731	C3orf55(dist=190522),SHOX2(dist=304257)	intergenic	HIVID	Yang et al .2018	30271481	Tumor
chr4	68265863	1221	LOC100144602(dist=1706759),CENPC1(dist=72126)	intergenic	HIVID	Yang et al .2018	30271481	Tumor
chr5	136444302	3074	SPOCK1	intronic	HIVID	Yang et al .2018	30271481	Tumor
chr8	71126926	2991	NCOA2	intronic	HIVID	Yang et al .2018	30271481	Tumor
chr8	71126934	2713	NCOA2	intronic	HIVID	Yang et al .2018	30271481	Tumor
chr1	10000	1816	NONE(dist=NONE),DDX11L1(dist=1874)	intergenic	HIVID	Yang et al .2018	30271481	Tumor
chr1	10082	1811	NONE(dist=NONE),DDX11L1(dist=1792)	intergenic	HIVID	Yang et al .2018	30271481	Tumor
chr1	10171	1814	NONE(dist=NONE),DDX11L1(dist=1703)	intergenic	HIVID	Yang et al .2018	30271481	Tumor
chr1	10350	1651	NONE(dist=NONE),DDX11L1(dist=1524)	intergenic	HIVID	Yang et al .2018	30271481	Tumor
chr1	10360	1811	NONE(dist=NONE),DDX11L1(dist=1514)	intergenic	HIVID	Yang et al .2018	30271481	Tumor
chr1	18829348	1936	KLHDC7A(dist=16868),PAX7(dist=128152)	intergenic	HIVID	Yang et al .2018	30271481	Tumor
chr1	65176730	168	CACHD1(dist=17989),RAVER2(dist=34048)	intergenic	HIVID	Yang et al .2018	30271481	Tumor
chr1	68970934	73	DEPDC1(dist=8135),LRRC7(dist=1254924)	intergenic	HIVID	Yang et al .2018	30271481	Tumor
chr1	78594425	1811	GIPC2	intronic	HIVID	Yang et al .2018	30271481	Tumor
chr1	105428312	1936	LOC100129138(dist=808619),NONE(dist=NONE)	intergenic	HIVID	Yang et al .2018	30271481	Tumor
chr1	121355023	1802	EMBP1(dist=41337),NONE(dist=NONE)	intergenic	HIVID	Yang et al .2018	30271481	Tumor
chr1	121458831	1802	EMBP1(dist=145145),NONE(dist=NONE)	intergenic	HIVID	Yang et al .2018	30271481	Tumor
chr1	121462562	1801	EMBP1(dist=148876),NONE(dist=NONE)	intergenic	HIVID	Yang et al .2018	30271481	Tumor
chr1	121468159	1802	EMBP1(dist=154473),NONE(dist=NONE)	intergenic	HIVID	Yang et al .2018	30271481	Tumor
chr1	155548496	162	ASH1L-AS1(dist=14761),MSTO1(dist=31465)	intergenic	HIVID	Yang et al .2018	30271481	Tumor
chr1	185664789	285	LOC100288079(dist=360618),HMCN1(dist=38894)	intergenic	HIVID	Yang et al .2018	30271481	Tumor
chr1	196571994	265	KCNT2	intronic	HIVID	Yang et al .2018	30271481	Tumor
chr1	226166757	1936	LEFTY2(dist=37674),SDE2(dist=3646)	intergenic	HIVID	Yang et al .2018	30271481	Tumor
chr1	249239927	1814	PGBD2(dist=26582),NONE(dist=NONE)	intergenic	HIVID	Yang et al .2018	30271481	Tumor
chr1	249240074	1814	PGBD2(dist=26729),NONE(dist=NONE)	intergenic	HIVID	Yang et al .2018	30271481	Tumor
chr1	249240224	1814	PGBD2(dist=26879),NONE(dist=NONE)	intergenic	HIVID	Yang et al .2018	30271481	Tumor
chr1	249240307	1814	PGBD2(dist=26962),NONE(dist=NONE)	intergenic	HIVID	Yang et al .2018	30271481	Tumor
chr1	249240400	1814	PGBD2(dist=27055),NONE(dist=NONE)	intergenic	HIVID	Yang et al .2018	30271481	Tumor
chr1	249240541	1809	PGBD2(dist=27196),NONE(dist=NONE)	intergenic	HIVID	Yang et al .2018	30271481	Tumor
chr10	34889877	1936	PARD3	intronic	HIVID	Yang et al .2018	30271481	Tumor
chr10	88512748	2325	LDB3(dist=16924),BMPR1A(dist=3648)	intergenic	HIVID	Yang et al .2018	30271481	Tumor
chr10	88541379	234	BMPR1A	intronic	HIVID	Yang et al .2018	30271481	Tumor
chr10	115897800	1936	C10orf118	intronic	HIVID	Yang et al .2018	30271481	Tumor
chr10	135524696	1814	DUX2(dist=26204),NONE(dist=NONE)	intergenic	HIVID	Yang et al .2018	30271481	Tumor
chr11	175547	1814	LOC100133161(dist=43627),SCGB1C1(dist=17533)	intergenic	HIVID	Yang et al .2018	30271481	Tumor
chr11	175743	1813	LOC100133161(dist=43823),SCGB1C1(dist=17337)	intergenic	HIVID	Yang et al .2018	30271481	Tumor
chr11	5152963	1809	OR52A5	exonic	HIVID	Yang et al .2018	30271481	Tumor
chr11	14702727	395	PDE3B	intronic	HIVID	Yang et al .2018	30271481	Tumor
chr11	14702811	1765	PDE3B	intronic	HIVID	Yang et al .2018	30271481	Tumor
chr11	27797291	194	BDNF(dist=53686),KIF18A(dist=244872)	intergenic	HIVID	Yang et al .2018	30271481	Tumor
chr11	30193666	694	KCNA4(dist=155089),FSHB(dist=58897)	intergenic	HIVID	Yang et al .2018	30271481	Tumor
chr11	30193778	1574	KCNA4(dist=155201),FSHB(dist=58785)	intergenic	HIVID	Yang et al .2018	30271481	Tumor
chr11	30193849	1577	KCNA4(dist=155272),FSHB(dist=58714)	intergenic	HIVID	Yang et al .2018	30271481	Tumor
chr11	46860165	1936	CKAP5	intronic	HIVID	Yang et al .2018	30271481	Tumor
chr11	51567326	1802	OR4C46(dist=51115),NONE(dist=NONE)	intergenic	HIVID	Yang et al .2018	30271481	Tumor
chr11	51569712	1802	OR4C46(dist=53501),NONE(dist=NONE)	intergenic	HIVID	Yang et al .2018	30271481	Tumor
chr11	51572044	1802	OR4C46(dist=55833),NONE(dist=NONE)	intergenic	HIVID	Yang et al .2018	30271481	Tumor
chr11	51573402	1802	OR4C46(dist=57191),NONE(dist=NONE)	intergenic	HIVID	Yang et al .2018	30271481	Tumor
chr11	51576093	1802	OR4C46(dist=59882),NONE(dist=NONE)	intergenic	HIVID	Yang et al .2018	30271481	Tumor
chr11	51580015	1802	OR4C46(dist=63804),NONE(dist=NONE)	intergenic	HIVID	Yang et al .2018	30271481	Tumor
chr11	51589044	1802	OR4C46(dist=72833),NONE(dist=NONE)	intergenic	HIVID	Yang et al .2018	30271481	Tumor
chr11	89789199	22	TRIM49C(dist=14006),UBTFL1(dist=29919)	intergenic	HIVID	Yang et al .2018	30271481	Tumor
chr12	95418	1814	LOC100288778(dist=4155),FAM138D(dist=52528)	intergenic	HIVID	Yang et al .2018	30271481	Tumor
chr12	95490	1781	LOC100288778(dist=4227),FAM138D(dist=52456)	intergenic	HIVID	Yang et al .2018	30271481	Tumor
chr12	95617	1814	LOC100288778(dist=4354),FAM138D(dist=52329)	intergenic	HIVID	Yang et al .2018	30271481	Tumor
chr12	95695	1814	LOC100288778(dist=4432),FAM138D(dist=52251)	intergenic	HIVID	Yang et al .2018	30271481	Tumor
chr12	217257	1811	IQSEC3	intronic	HIVID	Yang et al .2018	30271481	Tumor
chr12	19520193	1936	PLEKHA5	intronic	HIVID	Yang et al .2018	30271481	Tumor
chr12	20691684	26	PDE3A	intronic	HIVID	Yang et al .2018	30271481	Tumor
chr12	29031735	730	CCDC91(dist=328636),FAR2(dist=344863)	intergenic	HIVID	Yang et al .2018	30271481	Tumor
chr12	50261607	1263	FAIM2	UTR3	HIVID	Yang et al .2018	30271481	Tumor
chr12	53838186	111	PRR13	intronic	HIVID	Yang et al .2018	30271481	Tumor
chr12	66451373	2750	HMGA2(dist=91302),LLPH(dist=65476)	intergenic	HIVID	Yang et al .2018	30271481	Tumor
chr12	83966200	599	TMTC2(dist=438133),SLC6A15(dist=1287067)	intergenic	HIVID	Yang et al .2018	30271481	Tumor
chr12	88431875	2946	C12orf29	intronic	HIVID	Yang et al .2018	30271481	Tumor
chr12	91235617	28	LOC338758(dist=1129888),LINC00615(dist=76183)	intergenic	HIVID	Yang et al .2018	30271481	Tumor
chr12	95136120	156	TMCC3(dist=91796),MIR492(dist=92054)	intergenic	HIVID	Yang et al .2018	30271481	Tumor
chr12	113319973	2152	RPH3A	intronic	HIVID	Yang et al .2018	30271481	Tumor
chr12	133841524	1814	ANHX(dist=29102),NONE(dist=NONE)	intergenic	HIVID	Yang et al .2018	30271481	Tumor
chr12	133841599	1814	ANHX(dist=29177),NONE(dist=NONE)	intergenic	HIVID	Yang et al .2018	30271481	Tumor
chr12	133841671	1814	ANHX(dist=29249),NONE(dist=NONE)	intergenic	HIVID	Yang et al .2018	30271481	Tumor
chr12	133841874	1813	ANHX(dist=29452),NONE(dist=NONE)	intergenic	HIVID	Yang et al .2018	30271481	Tumor
chr13	20711525	1936	GJA3	downstream	HIVID	Yang et al .2018	30271481	Tumor
chr15	33486386	1936	FMN1(dist=126301),TMCO5B(dist=42291)	intergenic	HIVID	Yang et al .2018	30271481	Tumor
chr15	50801329	1936	USP50	intronic	HIVID	Yang et al .2018	30271481	Tumor
chr15	50801368	1028	USP50	intronic	HIVID	Yang et al .2018	30271481	Tumor
chr15	76228269	216	FBXO22	downstream	HIVID	Yang et al .2018	30271481	Tumor
chr15	90454143	1829	C15orf38,C15orf38-AP3S2	intronic	HIVID	Yang et al .2018	30271481	Tumor
chr15	90475423	1815	C15orf38-AP3S2(dist=19201),ZNF710(dist=69329)	intergenic	HIVID	Yang et al .2018	30271481	Tumor
chr15	102521231	1814	DDX11L9(dist=1935),NONE(dist=NONE)	intergenic	HIVID	Yang et al .2018	30271481	Tumor
chr16	25771423	1814	HS3ST4	intronic	HIVID	Yang et al .2018	30271481	Tumor
chr16	34543979	976	UBE2MP1(dist=139217),LOC283914(dist=53808)	intergenic	HIVID	Yang et al .2018	30271481	Tumor
chr16	54926584	168	IRX3(dist=606206),CRNDE(dist=26193)	intergenic	HIVID	Yang et al .2018	30271481	Tumor
chr16	67674887	1461	CTCF(dist=1799),RLTPR(dist=4143)	intergenic	HIVID	Yang et al .2018	30271481	Tumor
chr16	75368094	1742	CFDP1	intronic	HIVID	Yang et al .2018	30271481	Tumor
chr16	75368094	1813	CFDP1	intronic	HIVID	Yang et al .2018	30271481	Tumor
chr16	75368094	1814	CFDP1	intronic	HIVID	Yang et al .2018	30271481	Tumor
chr16	81313881	1028	BCMO1	intronic	HIVID	Yang et al .2018	30271481	Tumor
chr16	81313883	1930	BCMO1	intronic	HIVID	Yang et al .2018	30271481	Tumor
chr16	84639274	523	COTL1	intronic	HIVID	Yang et al .2018	30271481	Tumor

Chromosome	Integration site in host genome	Integration site in virus genome	Gene (distance, bp)	Regions	Methods	Author	PMID	Sample
chr17	8389856	1936	MYH10	intronic	HIVID	Yang et al .2018	30271481	Tumor
chr17	22244968	1802	MTRNR2L1(dist=220977),NONE(dist=NONE)	intergenic	HIVID	Yang et al .2018	30271481	Tumor
chr17	22246497	1798	MTRNR2L1(dist=222506),NONE(dist=NONE)	intergenic	HIVID	Yang et al .2018	30271481	Tumor
chr17	22249114	1730	MTRNR2L1(dist=225123),NONE(dist=NONE)	intergenic	HIVID	Yang et al .2018	30271481	Tumor
chr17	22249725	1799	MTRNR2L1(dist=225734),NONE(dist=NONE)	intergenic	HIVID	Yang et al .2018	30271481	Tumor
chr17	22251252	1798	MTRNR2L1(dist=227261),NONE(dist=NONE)	intergenic	HIVID	Yang et al .2018	30271481	Tumor
chr17	22253631	1798	MTRNR2L1(dist=229640),NONE(dist=NONE)	intergenic	HIVID	Yang et al .2018	30271481	Tumor
chr17	22256010	1773	MTRNR2L1(dist=232019),NONE(dist=NONE)	intergenic	HIVID	Yang et al .2018	30271481	Tumor
chr17	22256860	1798	MTRNR2L1(dist=232869),NONE(dist=NONE)	intergenic	HIVID	Yang et al .2018	30271481	Tumor
chr17	22258388	1802	MTRNR2L1(dist=234397),NONE(dist=NONE)	intergenic	HIVID	Yang et al .2018	30271481	Tumor
chr17	22259238	1802	MTRNR2L1(dist=235247),NONE(dist=NONE)	intergenic	HIVID	Yang et al .2018	30271481	Tumor
chr17	22260767	1797	MTRNR2L1(dist=236776),NONE(dist=NONE)	intergenic	HIVID	Yang et al .2018	30271481	Tumor
chr17	41230407	116	BRCA1	intronic	HIVID	Yang et al .2018	30271481	Tumor
chr17	70976044	535	SLC39A11	intronic	HIVID	Yang et al .2018	30271481	Tumor
chr18	9998	1811	NONE(dist=NONE),ROCK1P1(dist=99067)	intergenic	HIVID	Yang et al .2018	30271481	Tumor
chr18	10095	1814	NONE(dist=NONE),ROCK1P1(dist=98970)	intergenic	HIVID	Yang et al .2018	30271481	Tumor
chr18	10231	1814	NONE(dist=NONE),ROCK1P1(dist=98834)	intergenic	HIVID	Yang et al .2018	30271481	Tumor
chr18	10461	1810	NONE(dist=NONE),ROCK1P1(dist=98604)	intergenic	HIVID	Yang et al .2018	30271481	Tumor
chr18	63689	1814	NONE(dist=NONE),ROCK1P1(dist=45376)	intergenic	HIVID	Yang et al .2018	30271481	Tumor
chr18	5078152	121	DLGAP1(dist=622886),C18orf42(dist=65520)	intergenic	HIVID	Yang et al .2018	30271481	Tumor
chr18	23682640	677	SS18(dist=12029),PSMA8(dist=31176)	intergenic	HIVID	Yang et al .2018	30271481	Tumor
chr18	71132732	984	LOC100505817(dist=115608),FBXO15(dist=607856)	intergenic	HIVID	Yang et al .2018	30271481	Tumor
chr18	78016248	1808	PARD6G(dist=10851),NONE(dist=NONE)	intergenic	HIVID	Yang et al .2018	30271481	Tumor
chr18	78016329	1811	PARD6G(dist=10932),NONE(dist=NONE)	intergenic	HIVID	Yang et al .2018	30271481	Tumor
chr19	3152555	1814	GNA15	intronic	HIVID	Yang et al .2018	30271481	Tumor
chr19	9777000	1028	ZNF562	intronic	HIVID	Yang et al .2018	30271481	Tumor
chr19	21786895	2992	ZNF429(dist=65816),ZNF100(dist=119948)	intergenic	HIVID	Yang et al .2018	30271481	Tumor
chr19	21786998	545	ZNF429(dist=65919),ZNF100(dist=119845)	intergenic	HIVID	Yang et al .2018	30271481	Tumor
chr19	21787923	1878	ZNF429(dist=66844),ZNF100(dist=118920)	intergenic	HIVID	Yang et al .2018	30271481	Tumor
chr19	21788218	1823	ZNF429(dist=67139),ZNF100(dist=118625)	intergenic	HIVID	Yang et al .2018	30271481	Tumor
chr19	23064091	1028	ZNF99(dist=97118),ZNF728(dist=93594)	intergenic	HIVID	Yang et al .2018	30271481	Tumor
chr19	36212557	1805	KMT2B	exonic	HIVID	Yang et al .2018	30271481	Tumor
chr19	59118902	1814	MGC2752(dist=23140),NONE(dist=NONE)	intergenic	HIVID	Yang et al .2018	30271481	Tumor
chr2	33141320	1779	LINC00486	ncRNA_intronic	HIVID	Yang et al .2018	30271481	Tumor
chr2	104956052	30	TMEM182(dist=1521914),LOC100287010(dist=39256)	intergenic	HIVID	Yang et al .2018	30271481	Tumor
chr2	108104867	73	ST6GAL2(dist=601304),LOC729121(dist=334653)	intergenic	HIVID	Yang et al .2018	30271481	Tumor
chr2	110608760	1936	RGPD5,RGPD6	intronic	HIVID	Yang et al .2018	30271481	Tumor
chr2	111277865	1936	RGPD5,RGPD6	intronic	HIVID	Yang et al .2018	30271481	Tumor
chr2	127968939	1936	CYP27C1(dist=5596),ERCC3(dist=45927)	intergenic	HIVID	Yang et al .2018	30271481	Tumor
chr2	140766256	1028	LOC647012(dist=1109512),LRP1B(dist=222740)	intergenic	HIVID	Yang et al .2018	30271481	Tumor
chr2	179564297	36	TTN	intronic	HIVID	Yang et al .2018	30271481	Tumor
chr2	188215801	44	CALCRL	intronic	HIVID	Yang et al .2018	30271481	Tumor
chr2	243152476	1813	LOC728323(dist=50007),NONE(dist=NONE)	intergenic	HIVID	Yang et al .2018	30271481	Tumor
chr2	243152552	1814	LOC728323(dist=50083),NONE(dist=NONE)	intergenic	HIVID	Yang et al .2018	30271481	Tumor
chr20	15004453	1823	MACROD2	intronic	HIVID	Yang et al .2018	30271481	Tumor
chr20	23780601	156	CST1(dist=49027),CST2(dist=23803)	intergenic	HIVID	Yang et al .2018	30271481	Tumor
chr20	30564086	1557	XKR7	intronic	HIVID	Yang et al .2018	30271481	Tumor
chr20	30564095	1483	XKR7	intronic	HIVID	Yang et al .2018	30271481	Tumor
chr20	30564095	1554	XKR7	intronic	HIVID	Yang et al .2018	30271481	Tumor
chr20	30564100	1811	XKR7	intronic	HIVID	Yang et al .2018	30271481	Tumor
chr20	56139933	115	PCK1	intronic	HIVID	Yang et al .2018	30271481	Tumor
chr20	56139936	1549	PCK1	intronic	HIVID	Yang et al .2018	30271481	Tumor
chr21	36669075	1823	RUNX1(dist=247480),MIR802(dist=423938)	intergenic	HIVID	Yang et al .2018	30271481	Tumor
chr21	40882012	66	SH3BGR	intronic	HIVID	Yang et al .2018	30271481	Tumor
chr21	41857274	103	DSCAM	intronic	HIVID	Yang et al .2018	30271481	Tumor
chr21	48119808	1814	PRMT2(dist=34653),NONE(dist=NONE)	intergenic	HIVID	Yang et al .2018	30271481	Tumor
chr21	48119885	1814	PRMT2(dist=34730),NONE(dist=NONE)	intergenic	HIVID	Yang et al .2018	30271481	Tumor
chr22	29802103	1883	AP1B1(dist=17531),RFPL1-AS1(dist=30901)	intergenic	HIVID	Yang et al .2018	30271481	Tumor
chr3	1214793	1689	CNTN6	intronic	HIVID	Yang et al .2018	30271481	Tumor
chr3	5079897	1028	BHLHE40(dist=53032),ARL8B(dist=84033)	intergenic	HIVID	Yang et al .2018	30271481	Tumor
chr3	25488292	1936	RARB	intronic	HIVID	Yang et al .2018	30271481	Tumor
chr3	112467562	168	CCDC80(dist=107585),CD200R1L(dist=66994)	intergenic	HIVID	Yang et al .2018	30271481	Tumor
chr3	141188454	2436	ZBTB38(dist=19822),RASA2(dist=17472)	intergenic	HIVID	Yang et al .2018	30271481	Tumor
chr3	157509543	1731	C3orf55(dist=190522),SHOX2(dist=304257)	intergenic	HIVID	Yang et al .2018	30271481	Tumor
chr3	166551309	1936	BCHE(dist=996056),ZBBX(dist=406768)	intergenic	HIVID	Yang et al .2018	30271481	Tumor
chr3	171139506	1028	TNIK	intronic	HIVID	Yang et al .2018	30271481	Tumor
chr3	182645896	1039	ATP11B(dist=6475),DCUN1D1(dist=14663)	intergenic	HIVID	Yang et al .2018	30271481	Tumor
chr3	192952810	1044	MB21D2(dist=316860),HRASLS(dist=6107)	intergenic	HIVID	Yang et al .2018	30271481	Tumor
chr3	192955954	104	MB21D2(dist=320004),HRASLS(dist=2963)	intergenic	HIVID	Yang et al .2018	30271481	Tumor
chr3	196645119	41	SENP5	intronic	HIVID	Yang et al .2018	30271481	Tumor
chr3	197900164	1814	FAM157A	intronic	HIVID	Yang et al .2018	30271481	Tumor
chr3	197900330	1814	FAM157A	intronic	HIVID	Yang et al .2018	30271481	Tumor
chr3	197900737	1814	FAM157A	intronic	HIVID	Yang et al .2018	30271481	Tumor
chr3	197900821	1811	FAM157A	intronic	HIVID	Yang et al .2018	30271481	Tumor
chr4	10098	1814	NONE(dist=NONE),ZNF595(dist=43129)	intergenic	HIVID	Yang et al .2018	30271481	Tumor
chr4	4449319	1936	STX18	intronic	HIVID	Yang et al .2018	30271481	Tumor
chr4	9671023	1707	MIR548I2(dist=113086),DRD5(dist=112235)	intergenic	HIVID	Yang et al .2018	30271481	Tumor
chr4	17202838	96	LDB2(dist=302414),QDPR(dist=285178)	intergenic	HIVID	Yang et al .2018	30271481	Tumor
chr4	17750121	1028	FAM184B	intronic	HIVID	Yang et al .2018	30271481	Tumor
chr4	17750121	1881	FAM184B	intronic	HIVID	Yang et al .2018	30271481	Tumor
chr4	18491766	598	LCORL(dist=468283),SLIT2(dist=1763469)	intergenic	HIVID	Yang et al .2018	30271481	Tumor
chr4	40925168	136	APBB2	intronic	HIVID	Yang et al .2018	30271481	Tumor
chr4	43179034	1926	GRXCR1(dist=146359),KCTD8(dist=996886)	intergenic	HIVID	Yang et al .2018	30271481	Tumor
chr4	43179535	1943	GRXCR1(dist=146860),KCTD8(dist=996385)	intergenic	HIVID	Yang et al .2018	30271481	Tumor
chr4	43198570	1516	GRXCR1(dist=165895),KCTD8(dist=977350)	intergenic	HIVID	Yang et al .2018	30271481	Tumor
chr4	56985038	1806	CEP135(dist=85509),KIAA1211(dist=51323)	intergenic	HIVID	Yang et al .2018	30271481	Tumor
chr4	63151772	103	LPHN3(dist=213604),TECRL(dist=1992405)	intergenic	HIVID	Yang et al .2018	30271481	Tumor
chr4	64979906	1829	NONE(dist=NONE),TECRL(dist=164271)	intergenic	HIVID	Yang et al .2018	30271481	Tumor
chr4	87225744	46	MAPK10	intronic	HIVID	Yang et al .2018	30271481	Tumor
chr4	103027989	1936	BANK1(dist=32020),SLC39A8(dist=144209)	intergenic	HIVID	Yang et al .2018	30271481	Tumor
chr4	103027991	1028	BANK1(dist=32022),SLC39A8(dist=144207)	intergenic	HIVID	Yang et al .2018	30271481	Tumor
chr4	147154367	72	LSM6(dist=43154),SLC10A7(dist=20770)	intergenic	HIVID	Yang et al .2018	30271481	Tumor
chr4	166602912	970	CPE(dist=183430),TLL1(dist=191498)	intergenic	HIVID	Yang et al .2018	30271481	Tumor
chr4	191043946	1814	DUX4L2(dist=30470),NONE(dist=NONE)	intergenic	HIVID	Yang et al .2018	30271481	Tumor

Chromosome	Integration site in host genome	Integration site in virus genome	Gene (distance, bp)	Regions	Methods	Author	PMID	Sample
chr4	191044053	1814	DUX4L2(dist=30577),NONE(dist=NONE)	intergenic	HIVID	Yang et al .2018	30271481	Tumor
chr4	191044167	1810	DUX4L2(dist=30691),NONE(dist=NONE)	intergenic	HIVID	Yang et al .2018	30271481	Tumor
chr5	10021	1814	NONE(dist=NONE),PLEKHG4B(dist=130352)	intergenic	HIVID	Yang et al .2018	30271481	Tumor
chr5	10111	1802	NONE(dist=NONE),PLEKHG4B(dist=130262)	intergenic	HIVID	Yang et al .2018	30271481	Tumor
chr5	10184	1811	NONE(dist=NONE),PLEKHG4B(dist=130189)	intergenic	HIVID	Yang et al .2018	30271481	Tumor
chr5	10255	1802	NONE(dist=NONE),PLEKHG4B(dist=130118)	intergenic	HIVID	Yang et al .2018	30271481	Tumor
chr5	10375	1814	NONE(dist=NONE),PLEKHG4B(dist=129998)	intergenic	HIVID	Yang et al .2018	30271481	Tumor
chr5	10447	1814	NONE(dist=NONE),PLEKHG4B(dist=129926)	intergenic	HIVID	Yang et al .2018	30271481	Tumor
chr5	10598	1811	NONE(dist=NONE),PLEKHG4B(dist=129775)	intergenic	HIVID	Yang et al .2018	30271481	Tumor
chr5	10826	1811	NONE(dist=NONE),PLEKHG4B(dist=129547)	intergenic	HIVID	Yang et al .2018	30271481	Tumor
chr5	10910	1811	NONE(dist=NONE),PLEKHG4B(dist=129463)	intergenic	HIVID	Yang et al .2018	30271481	Tumor
chr5	11113	1814	NONE(dist=NONE),PLEKHG4B(dist=129260)	intergenic	HIVID	Yang et al .2018	30271481	Tumor
chr5	11210	1811	NONE(dist=NONE),PLEKHG4B(dist=129163)	intergenic	HIVID	Yang et al .2018	30271481	Tumor
chr5	11366	1811	NONE(dist=NONE),PLEKHG4B(dist=129007)	intergenic	HIVID	Yang et al .2018	30271481	Tumor
chr5	11467	1802	NONE(dist=NONE),PLEKHG4B(dist=128906)	intergenic	HIVID	Yang et al .2018	30271481	Tumor
chr5	11580	1814	NONE(dist=NONE),PLEKHG4B(dist=128793)	intergenic	HIVID	Yang et al .2018	30271481	Tumor
chr5	11678	1810	NONE(dist=NONE),PLEKHG4B(dist=128695)	intergenic	HIVID	Yang et al .2018	30271481	Tumor
chr5	1295701	1813	TERT	upstream	HIVID	Yang et al .2018	30271481	Tumor
chr5	18675821	2426	LOC401177(dist=1288402),CDH18(dist=797334)	intergenic	HIVID	Yang et al .2018	30271481	Tumor
chr5	40729097	1811	TTC33	intronic	HIVID	Yang et al .2018	30271481	Tumor
chr5	43033265	57	LOC648987(dist=14352),ANXA2R(dist=5917)	intergenic	HIVID	Yang et al .2018	30271481	Tumor
chr5	68942337	1936	GUSBP3	ncRNA_intronic	HIVID	Yang et al .2018	30271481	Tumor
chr5	69797175	1936	SMA5	ncRNA_intronic	HIVID	Yang et al .2018	30271481	Tumor
chr5	70504399	1936	LOC647859(dist=115502),GUSBP9(dist=11654)	intergenic	HIVID	Yang et al .2018	30271481	Tumor
chr5	71902930	1936	ZNF366(dist=99681),TNPO1(dist=209488)	intergenic	HIVID	Yang et al .2018	30271481	Tumor
chr5	82811939	1814	VCAN	intronic	HIVID	Yang et al .2018	30271481	Tumor
chr5	87619311	66	TMEM161B-AS1	ncRNA_intronic	HIVID	Yang et al .2018	30271481	Tumor
chr5	95736525	9	PCSK1	intronic	HIVID	Yang et al .2018	30271481	Tumor
chr5	121223222	3020	FTMT(dist=34699),SRFBP1(dist=74434)	intergenic	HIVID	Yang et al .2018	30271481	Tumor
chr5	133740994	156	CDKN2AIPNL	intronic	HIVID	Yang et al .2018	30271481	Tumor
chr5	136444304	3070	SPOCK1	intronic	HIVID	Yang et al .2018	30271481	Tumor
chr5	159075340	38	LOC285627(dist=182056),ADRA1B(dist=268400)	intergenic	HIVID	Yang et al .2018	30271481	Tumor
chr5	180473053	611	BTNL9	intronic	HIVID	Yang et al .2018	30271481	Tumor
chr5	180473173	611	BTNL9	intronic	HIVID	Yang et al .2018	30271481	Tumor
chr5	180477469	1386	BTNL9	intronic	HIVID	Yang et al .2018	30271481	Tumor
chr6	16249387	1028	GMPR	intronic	HIVID	Yang et al .2018	30271481	Tumor
chr6	25126607	103	CMAHP	ncRNA_intronic	HIVID	Yang et al .2018	30271481	Tumor
chr6	47868726	51	PTCHD4	UTR3	HIVID	Yang et al .2018	30271481	Tumor
chr6	74469944	162	CD109	intronic	HIVID	Yang et al .2018	30271481	Tumor
chr6	86091086	78	TBX18(dist=617187),NT5E(dist=68216)	intergenic	HIVID	Yang et al .2018	30271481	Tumor
chr6	154477153	1433	IPCEF1	UTR3	HIVID	Yang et al .2018	30271481	Tumor
chr6	163099137	123	PARK2	intronic	HIVID	Yang et al .2018	30271481	Tumor
chr7	5503392	1936	TNRC18(dist=40215),FBXL18(dist=12036)	intergenic	HIVID	Yang et al .2018	30271481	Tumor
chr7	81277437	2417	SEMA3C(dist=728770),HGF(dist=54007)	intergenic	HIVID	Yang et al .2018	30271481	Tumor
chr7	85074004	1936	SEMA3D(dist=322757),GRM3(dist=1199226)	intergenic	HIVID	Yang et al .2018	30271481	Tumor
chr7	97445931	74	TAC1(dist=76147),ASNS(dist=35498)	intergenic	HIVID	Yang et al .2018	30271481	Tumor
chr7	103161396	1936	RELN	intronic	HIVID	Yang et al .2018	30271481	Tumor
chr7	135353961	1823	C7orf73	intronic	HIVID	Yang et al .2018	30271481	Tumor
chr7	139934292	1032	LOC100134229(dist=54852),SLC37A3(dist=99260)	intergenic	HIVID	Yang et al .2018	30271481	Tumor
chr7	141113974	99	TMEM178B	intronic	HIVID	Yang et al .2018	30271481	Tumor
chr7	148106864	156	CNTNAP2	intronic	HIVID	Yang et al .2018	30271481	Tumor
chr8	42463327	37	C8orf40(dist=55187),CHRNB3(dist=89235)	intergenic	HIVID	Yang et al .2018	30271481	Tumor
chr8	51787482	301	SNTG1(dist=82055),PXDNL(dist=444655)	intergenic	HIVID	Yang et al .2018	30271481	Tumor
chr8	61343206	103	CA8(dist=149252),RAB2A(dist=86263)	intergenic	HIVID	Yang et al .2018	30271481	Tumor
chr8	62666659	1814	MIR4470(dist=39241),NKAIN3(dist=494842)	intergenic	HIVID	Yang et al .2018	30271481	Tumor
chr8	68645430	1936	CPA6	intronic	HIVID	Yang et al .2018	30271481	Tumor
chr8	71126934	2713	NCOA2	intronic	HIVID	Yang et al .2018	30271481	Tumor
chr8	91558728	66	LINC00534(dist=158541),TMEM64(dist=75495)	intergenic	HIVID	Yang et al .2018	30271481	Tumor
chr8	96219669	9	C8orf69	ncRNA_intronic	HIVID	Yang et al .2018	30271481	Tumor
chr8	97098140	30	LOC100500773(dist=137564),GDF6(dist=56418)	intergenic	HIVID	Yang et al .2018	30271481	Tumor
chr8	132693273	1028	ADCY8(dist=640438),EFR3A(dist=223083)	intergenic	HIVID	Yang et al .2018	30271481	Tumor
chr8	132693304	1936	ADCY8(dist=640469),EFR3A(dist=223052)	intergenic	HIVID	Yang et al .2018	30271481	Tumor
chr9	28517157	1992	LINGO2	intronic	HIVID	Yang et al .2018	30271481	Tumor
chr9	28517157	2064	LINGO2	intronic	HIVID	Yang et al .2018	30271481	Tumor
chr9	67587641	11	AQP7P1(dist=298149),FAM27B(dist=205288)	intergenic	HIVID	Yang et al .2018	30271481	Tumor
chr9	68488409	542	LOC642236(dist=34034),LOC100132352(dist=238132)	intergenic	HIVID	Yang et al .2018	30271481	Tumor
chr9	74828573	11	GDA	intronic	HIVID	Yang et al .2018	30271481	Tumor
chr9	86242802	1028	IDNK	intronic	HIVID	Yang et al .2018	30271481	Tumor
chr9	93038035	1039	LOC286370(dist=234254),LOC340515(dist=186679)	intergenic	HIVID	Yang et al .2018	30271481	Tumor
chr9	98724327	1823	ERCC6L2	intronic	HIVID	Yang et al .2018	30271481	Tumor
chr9	111148036	32	KLF4(dist=895989),ACTL7B(dist=468833)	intergenic	HIVID	Yang et al .2018	30271481	Tumor
chr9	111307339	495	KLF4(dist=1055292),ACTL7B(dist=309530)	intergenic	HIVID	Yang et al .2018	30271481	Tumor
chr9	131750990	1936	NUP188	intronic	HIVID	Yang et al .2018	30271481	Tumor
chr9	133997413	64	AIF1L	UTR3	HIVID	Yang et al .2018	30271481	Tumor
chr9	141054317	1814	TUBBP5	ncRNA_intronic	HIVID	Yang et al .2018	30271481	Tumor
chrX	16428179	1936	GRPR(dist=256538),CTPS2(dist=177943)	intergenic	HIVID	Yang et al .2018	30271481	Tumor
chrX	22346863	1936	LOC100873065	ncRNA_intronic	HIVID	Yang et al .2018	30271481	Tumor
chrX	58570767	1690	ZXDA(dist=633700),NONE(dist=NONE)	intergenic	HIVID	Yang et al .2018	30271481	Tumor
chrX	61706546	1744	NONE(dist=NONE),SPIN4(dist=860561)	intergenic	HIVID	Yang et al .2018	30271481	Tumor
chrX	61708063	1802	NONE(dist=NONE),SPIN4(dist=859044)	intergenic	HIVID	Yang et al .2018	30271481	Tumor
chrX	61710685	1677	NONE(dist=NONE),SPIN4(dist=856422)	intergenic	HIVID	Yang et al .2018	30271481	Tumor
chrX	61723050	1802	NONE(dist=NONE),SPIN4(dist=844057)	intergenic	HIVID	Yang et al .2018	30271481	Tumor
chrX	73829320	1936	RLIM	intronic	HIVID	Yang et al .2018	30271481	Tumor
chrX	113144127	81	AMOT(dist=1060084),HTR2C(dist=674424)	intergenic	HIVID	Yang et al .2018	30271481	Tumor
chrX	133450727	1936	CCDC160(dist=70919),PHF6(dist=56615)	intergenic	HIVID	Yang et al .2018	30271481	Tumor
chrX	134778591	1739	DDX26B(dist=62131),CT45A1(dist=68594)	intergenic	HIVID	Yang et al .2018	30271481	Tumor
chrX	134778636	1650	DDX26B(dist=62176),CT45A1(dist=68549)	intergenic	HIVID	Yang et al .2018	30271481	Tumor
chrX	134778649	1821	DDX26B(dist=62189),CT45A1(dist=68536)	intergenic	HIVID	Yang et al .2018	30271481	Tumor
chrX	134778873	1055	DDX26B(dist=62413),CT45A1(dist=68312)	intergenic	HIVID	Yang et al .2018	30271481	Tumor
chrX	137949906	1823	FGF13	intronic	HIVID	Yang et al .2018	30271481	Tumor
chrX	140426930	29	SPANXC(dist=90284),SPANXA2-OT1(dist=163913)	intergenic	HIVID	Yang et al .2018	30271481	Tumor
chrX	155260076	1814	IL9R(dist=19594),NONE(dist=NONE)	intergenic	HIVID	Yang et al .2018	30271481	Tumor
chrX	155260160	1802	IL9R(dist=19678),NONE(dist=NONE)	intergenic	HIVID	Yang et al .2018	30271481	Tumor
chrX	155260242	1814	IL9R(dist=19760),NONE(dist=NONE)	intergenic	HIVID	Yang et al .2018	30271481	Tumor

Chromosome	Integration site in host genome	Integration site in virus genome	Gene (distance, bp)	Regions	Methods	Author	PMID	Sample
chrX	155260281	1727	IL9R(dist=19799),NONE(dist=NONE)	intergenic	HIVID	Yang et al .2018	30271481	Tumor
chrX	155260316	1814	IL9R(dist=19834),NONE(dist=NONE)	intergenic	HIVID	Yang et al .2018	30271481	Tumor
chrX	155260388	1814	IL9R(dist=19906),NONE(dist=NONE)	intergenic	HIVID	Yang et al .2018	30271481	Tumor
chrX	155260467	1814	IL9R(dist=19985),NONE(dist=NONE)	intergenic	HIVID	Yang et al .2018	30271481	Tumor
chrY	9998	1811	NONE(dist=NONE),PLCXD1(dist=138063)	intergenic	HIVID	Yang et al .2018	30271481	Tumor
chrY	18039359	1936	NLGN4Y(dist=1083511),FAM41AY1(dist=1573479)	intergenic	HIVID	Yang et al .2018	30271481	Tumor
chrY	59362904	1765	IL9R(dist=19416),NONE(dist=NONE)	intergenic	HIVID	Yang et al .2018	30271481	Tumor
chrY	59363094	1814	IL9R(dist=19606),NONE(dist=NONE)	intergenic	HIVID	Yang et al .2018	30271481	Tumor
chrY	59363240	1814	IL9R(dist=19752),NONE(dist=NONE)	intergenic	HIVID	Yang et al .2018	30271481	Tumor
chrY	59363287	1727	IL9R(dist=19799),NONE(dist=NONE)	intergenic	HIVID	Yang et al .2018	30271481	Tumor
chrY	59363287	1743	IL9R(dist=19799),NONE(dist=NONE)	intergenic	HIVID	Yang et al .2018	30271481	Tumor
chrY	59363287	1814	IL9R(dist=19799),NONE(dist=NONE)	intergenic	HIVID	Yang et al .2018	30271481	Tumor
chrY	59363358	1814	IL9R(dist=19870),NONE(dist=NONE)	intergenic	HIVID	Yang et al .2018	30271481	Tumor
chrY	59363473	1814	IL9R(dist=19985),NONE(dist=NONE)	intergenic	HIVID	Yang et al .2018	30271481	Tumor
chr1	12716236	1818	AADACL4	intronic	HIVID	Yang et al .2018	30271481	Tumor
chr1	45976721	1731	MMACHC,PRDX1	UTR3	HIVID	Yang et al .2018	30271481	Tumor
chr1	224201517	2370	TP53BP2(dist=167843),FBXO28(dist=100272)	intergenic	HIVID	Yang et al .2018	30271481	Tumor
chr1	241741597	521	KMO	intronic	HIVID	Yang et al .2018	30271481	Tumor
chr10	88512743	1837	LDB3(dist=16919),BMPR1A(dist=3653)	intergenic	HIVID	Yang et al .2018	30271481	Tumor
chr10	88512748	2325	LDB3(dist=16924),BMPR1A(dist=3648)	intergenic	HIVID	Yang et al .2018	30271481	Tumor
chr11	79307272	1330	ODZ4(dist=155577),NONE(dist=NONE)	intergenic	HIVID	Yang et al .2018	30271481	Tumor
chr12	2250290	439	CACNA1C	intronic	HIVID	Yang et al .2018	30271481	Tumor
chr12	20691809	2713	PDE3A	intronic	HIVID	Yang et al .2018	30271481	Tumor
chr15	102089067	1799	PCSK6(dist=58880),TM2D3(dist=92982)	intergenic	HIVID	Yang et al .2018	30271481	Tumor
chr18	64326428	683	CDH19(dist=55212),MIR5011(dist=422393)	intergenic	HIVID	Yang et al .2018	30271481	Tumor
chr19	8499196	903	42065	intronic	HIVID	Yang et al .2018	30271481	Tumor
chr19	8499196	1819	42065	intronic	HIVID	Yang et al .2018	30271481	Tumor
chr19	36212557	1805	KMT2B	exonic	HIVID	Yang et al .2018	30271481	Tumor
chr2	75737187	1790	FAM176A	intronic	HIVID	Yang et al .2018	30271481	Tumor
chr2	109816211	2053	SH3RF3	intronic	HIVID	Yang et al .2018	30271481	Tumor
chr2	184735556	2799	NUP35(dist=709148),ZNF804A(dist=727537)	intergenic	HIVID	Yang et al .2018	30271481	Tumor
chr3	186667311	454	ST6GAL1	intronic	HIVID	Yang et al .2018	30271481	Tumor
chr3	188803523	1818	LPP(dist=195063),TPRG1(dist=86240)	intergenic	HIVID	Yang et al .2018	30271481	Tumor
chr3	188803536	1816	LPP(dist=195076),TPRG1(dist=86227)	intergenic	HIVID	Yang et al .2018	30271481	Tumor
chr4	69033073	1796	TMPRSS11F(dist=37486),FTLP10(dist=14937)	intergenic	HIVID	Yang et al .2018	30271481	Tumor
chr4	92684362	1775	FAM190A(dist=160992),GRID2(dist=541188)	intergenic	HIVID	Yang et al .2018	30271481	Tumor
chr4	166602912	970	CPE(dist=183430),TLL1(dist=191498)	intergenic	HIVID	Yang et al .2018	30271481	Tumor
chr5	1295701	1813	TERT	upstream	HIVID	Yang et al .2018	30271481	Tumor
chr5	86979814	256	CCNH(dist=270964),TMEM161B(dist=511209)	intergenic	HIVID	Yang et al .2018	30271481	Tumor
chr6	163099137	123	PARK2	intronic	HIVID	Yang et al .2018	30271481	Tumor
chr8	1332927	784	LOC286083(dist=82100),DLGAP2(dist=116642)	intergenic	HIVID	Yang et al .2018	30271481	Tumor
chr8	51787482	301	SNTG1(dist=82055),PXDNL(dist=444655)	intergenic	HIVID	Yang et al .2018	30271481	Tumor
chr8	87940598	2330	CNBD1	intronic	HIVID	Yang et al .2018	30271481	Tumor
chr8	129524634	1800	MIR1208(dist=362200),LOC728724(dist=704079)	intergenic	HIVID	Yang et al .2018	30271481	Tumor
chr8	129524908	1612	MIR1208(dist=362474),LOC728724(dist=703805)	intergenic	HIVID	Yang et al .2018	30271481	Tumor
chrY	13137788	255	NONE(dist=NONE),GYG2P1(dist=1380127)	intergenic	HIVID	Yang et al .2018	30271481	Tumor
chrY	13869868	255	NONE(dist=NONE),GYG2P1(dist=648047)	intergenic	HIVID	Yang et al .2018	30271481	Tumor
chr3	108405548	1640	DZIP3	intronic	HIVID	Yang et al .2018	30271481	Tumor
chr3	108405548	2950	DZIP3	intronic	HIVID	Yang et al .2018	30271481	Tumor
chr5	1295179	1827	TERT	upstream	HIVID	Yang et al .2018	30271481	Tumor
chr19	35671796	2459	FXYD5(dist=11008),FAM187B(dist=43908)	intergenic	HIVID	Yang et al .2018	30271481	Tumor
chr5	1295572	1719	TERT	upstream	HIVID	Yang et al .2018	30271481	Tumor
chr19	38680643	1328	SIPA1L3	intronic	HIVID	Yang et al .2018	30271481	Tumor
chr1	9544026	1463	SPSB1(dist=114436),SLC25A33(dist=55502)	intergenic	HIVID	Yang et al .2018	30271481	Tumor
chr1	9640669	1820	SLC25A33	intronic	HIVID	Yang et al .2018	30271481	Tumor
chr1	53677670	26	CPT2	intronic	HIVID	Yang et al .2018	30271481	Tumor
chr1	62651147	1639	INADL(dist=21556),L1TD1(dist=9327)	intergenic	HIVID	Yang et al .2018	30271481	Tumor
chr1	80407608	2876	ELTD1(dist=935113),LPHN2(dist=1858474)	intergenic	HIVID	Yang et al .2018	30271481	Tumor
chr1	151571962	2588	TUFT1(dist=15903),SNX27(dist=12700)	intergenic	HIVID	Yang et al .2018	30271481	Tumor
chr1	151852568	26	THEM4	intronic	HIVID	Yang et al .2018	30271481	Tumor
chr1	158269671	2132	CD1C(dist=5107),CD1B(dist=28069)	intergenic	HIVID	Yang et al .2018	30271481	Tumor
chr1	220818842	344	MARK1	intronic	HIVID	Yang et al .2018	30271481	Tumor
chr1	248194670	2720	OR2L13	intronic	HIVID	Yang et al .2018	30271481	Tumor
chr1	248873416	26	OR14I1(dist=27811),LYPD8(dist=29301)	intergenic	HIVID	Yang et al .2018	30271481	Tumor
chr10	313210	2630	ZMYND11(dist=12633),DIP2C(dist=6920)	intergenic	HIVID	Yang et al .2018	30271481	Tumor
chr10	6493159	2132	PRKCQ	intronic	HIVID	Yang et al .2018	30271481	Tumor
chr10	30426151	26	KIAA1462(dist=77663),MTPAP(dist=172579)	intergenic	HIVID	Yang et al .2018	30271481	Tumor
chr10	38805378	2160	LOC399744(dist=64297),ACTR3BP5(dist=184349)	intergenic	HIVID	Yang et al .2018	30271481	Tumor
chr10	38874142	2174	LOC399744(dist=133061),ACTR3BP5(dist=115585)	intergenic	HIVID	Yang et al .2018	30271481	Tumor
chr10	74959369	26	FAM149B1	intronic	HIVID	Yang et al .2018	30271481	Tumor
chr10	88512739	1837	LDB3(dist=16915),BMPR1A(dist=3657)	intergenic	HIVID	Yang et al .2018	30271481	Tumor
chr10	88512748	2325	LDB3(dist=16924),BMPR1A(dist=3648)	intergenic	HIVID	Yang et al .2018	30271481	Tumor
chr10	126645297	1639	ZRANB1	intronic	HIVID	Yang et al .2018	30271481	Tumor
chr10	135524606	2588	DUX2(dist=26114),NONE(dist=NONE)	intergenic	HIVID	Yang et al .2018	30271481	Tumor
chr11	30193847	1574	KCNA4(dist=155270),FSHB(dist=58716)	intergenic	HIVID	Yang et al .2018	30271481	Tumor
chr11	68466996	2713	GAL(dist=8353),MTL5(dist=7912)	intergenic	HIVID	Yang et al .2018	30271481	Tumor
chr11	69593096	1347	FGF4(dist=2925),FGF3(dist=31640)	intergenic	HIVID	Yang et al .2018	30271481	Tumor
chr11	69608587	1843	FGF4(dist=18416),FGF3(dist=16149)	intergenic	HIVID	Yang et al .2018	30271481	Tumor
chr11	78150340	1606	NARS2	intronic	HIVID	Yang et al .2018	30271481	Tumor
chr11	103689072	2876	DYNC2H1(dist=338481),MIR4693(dist=31562)	intergenic	HIVID	Yang et al .2018	30271481	Tumor
chr12	893216	1957	WNK1	intronic	HIVID	Yang et al .2018	30271481	Tumor
chr12	893216	1957	WNK1	intronic	HIVID	Yang et al .2018	30271481	Tumor
chr12	2250290	434	CACNA1C	intronic	HIVID	Yang et al .2018	30271481	Tumor
chr12	2250303	1734	CACNA1C	intronic	HIVID	Yang et al .2018	30271481	Tumor
chr12	2250351	1363	CACNA1C	intronic	HIVID	Yang et al .2018	30271481	Tumor
chr12	3098319	2634	TEAD4	intronic	HIVID	Yang et al .2018	30271481	Tumor
chr12	5429992	2964	KCNA5(dist=274038),NTF3(dist=111288)	intergenic	HIVID	Yang et al .2018	30271481	Tumor
chr12	5430048	1858	KCNA5(dist=274094),NTF3(dist=111232)	intergenic	HIVID	Yang et al .2018	30271481	Tumor
chr12	5432581	2801	KCNA5(dist=276627),NTF3(dist=108699)	intergenic	HIVID	Yang et al .2018	30271481	Tumor
chr12	5967300	2132	ANO2	intronic	HIVID	Yang et al .2018	30271481	Tumor
chr12	7291403	2263	CLSTN3	intronic	HIVID	Yang et al .2018	30271481	Tumor
chr12	7291699	2801	CLSTN3	intronic	HIVID	Yang et al .2018	30271481	Tumor
chr12	7576194	2132	CD163L1	intronic	HIVID	Yang et al .2018	30271481	Tumor
chr12	7605105	3070	CD163L1(dist=8356),CD163(dist=18307)	intergenic	HIVID	Yang et al .2018	30271481	Tumor

Chromosome	Integration site in host genome	Integration site in virus genome	Gene (distance, bp)	Regions	Methods	Author	PMID	Sample
chr12	20691683	26	PDE3A	intronic	HIVID	Yang et al .2018	30271481	Tumor
chr12	20691805	2713	PDE3A	intronic	HIVID	Yang et al .2018	30271481	Tumor
chr12	26228833	1033	RASSF8	intronic	HIVID	Yang et al .2018	30271481	Tumor
chr12	27236556	2364	C12orf71(dist=1101),STK38L(dist=160522)	intergenic	HIVID	Yang et al .2018	30271481	Tumor
chr12	27236694	753	C12orf71(dist=1239),STK38L(dist=160384)	intergenic	HIVID	Yang et al .2018	30271481	Tumor
chr12	27240910	964	C12orf71(dist=5455),STK38L(dist=156168)	intergenic	HIVID	Yang et al .2018	30271481	Tumor
chr12	28021409	2969	KLHDC5(dist=65436),PTHLH(dist=89608)	intergenic	HIVID	Yang et al .2018	30271481	Tumor
chr12	28441850	2176	CCDC91	intronic	HIVID	Yang et al .2018	30271481	Tumor
chr12	29031684	730	CCDC91(dist=328585),FAR2(dist=344914)	intergenic	HIVID	Yang et al .2018	30271481	Tumor
chr12	29031940	1606	CCDC91(dist=328841),FAR2(dist=344658)	intergenic	HIVID	Yang et al .2018	30271481	Tumor
chr12	29035972	1686	CCDC91(dist=332873),FAR2(dist=340626)	intergenic	HIVID	Yang et al .2018	30271481	Tumor
chr12	39236639	1819	CPNE8	intronic	HIVID	Yang et al .2018	30271481	Tumor
chr12	50261607	1263	FAIM2	UTR3	HIVID	Yang et al .2018	30271481	Tumor
chr12	55956753	2876	OR6C4(dist=10813),OR2AP1(dist=11446)	intergenic	HIVID	Yang et al .2018	30271481	Tumor
chr12	55986209	2876	OR2AP1(dist=17081),OR10P1(dist=44467)	intergenic	HIVID	Yang et al .2018	30271481	Tumor
chr12	77656307	2876	E2F7(dist=196947),NAV3(dist=568762)	intergenic	HIVID	Yang et al .2018	30271481	Tumor
chr12	88431795	2850	C12orf29	intronic	HIVID	Yang et al .2018	30271481	Tumor
chr12	106570972	1819	NUAK1(dist=37161),CKAP4(dist=60687)	intergenic	HIVID	Yang et al .2018	30271481	Tumor
chr12	112782636	26	HECTD4	intronic	HIVID	Yang et al .2018	30271481	Tumor
chr12	120885965	26	GATC	intronic	HIVID	Yang et al .2018	30271481	Tumor
chr13	37648094	1639	FAM48A(dist=14244),CSNK1A1L(dist=29303)	intergenic	HIVID	Yang et al .2018	30271481	Tumor
chr13	84230337	1824	NONE(dist=NONE),SLITRK1(dist=221006)	intergenic	HIVID	Yang et al .2018	30271481	Tumor
chr13	87696849	2132	SLITRK6(dist=1323366),MIR4500HG(dist=399393)	intergenic	HIVID	Yang et al .2018	30271481	Tumor
chr14	26140573	1819	STXBP6(dist=621478),NOVA1(dist=774516)	intergenic	HIVID	Yang et al .2018	30271481	Tumor
chr14	30246983	2876	PRKD1	intronic	HIVID	Yang et al .2018	30271481	Tumor
chr14	32989035	2876	AKAP6	intronic	HIVID	Yang et al .2018	30271481	Tumor
chr14	69860652	26	ERH	intronic	HIVID	Yang et al .2018	30271481	Tumor
chr15	24400964	2876	NDN(dist=468514),PWRN2(dist=8962)	intergenic	HIVID	Yang et al .2018	30271481	Tumor
chr15	30799205	1819	CHRFAM7A(dist=113341),ULK4P1(dist=65553)	intergenic	HIVID	Yang et al .2018	30271481	Tumor
chr15	66245089	1866	MEGF11	intronic	HIVID	Yang et al .2018	30271481	Tumor
chr15	66245097	1821	MEGF11	intronic	HIVID	Yang et al .2018	30271481	Tumor
chr15	74824567	1639	LOC440288(dist=50934),ARID3B(dist=8981)	intergenic	HIVID	Yang et al .2018	30271481	Tumor
chr15	90475471	1815	C15orf38-AP3S2(dist=19249),ZNF710(dist=69281)	intergenic	HIVID	Yang et al .2018	30271481	Tumor
chr15	102089067	1799	PCSK6(dist=58880),TM2D3(dist=92982)	intergenic	HIVID	Yang et al .2018	30271481	Tumor
chr16	10434971	1639	GRIN2A(dist=158360),ATF7IP2(dist=44941)	intergenic	HIVID	Yang et al .2018	30271481	Tumor
chr16	16134584	26	ABCC1	intronic	HIVID	Yang et al .2018	30271481	Tumor
chr16	67674887	1461	CTCF(dist=1799),RLTPR(dist=4143)	intergenic	HIVID	Yang et al .2018	30271481	Tumor
chr16	67709419	1041	GFOD2	exonic	HIVID	Yang et al .2018	30271481	Tumor
chr17	1744119	1819	RPA1	intronic	HIVID	Yang et al .2018	30271481	Tumor
chr17	14984304	2354	CDRT7(dist=49030),PMP22(dist=148792)	intergenic	HIVID	Yang et al .2018	30271481	Tumor
chr17	22245615	908	MTRNR2L1(dist=221624),NONE(dist=NONE)	intergenic	HIVID	Yang et al .2018	30271481	Tumor
chr17	22247994	908	MTRNR2L1(dist=224003),NONE(dist=NONE)	intergenic	HIVID	Yang et al .2018	30271481	Tumor
chr17	22250373	908	MTRNR2L1(dist=226382),NONE(dist=NONE)	intergenic	HIVID	Yang et al .2018	30271481	Tumor
chr17	22252749	908	MTRNR2L1(dist=228758),NONE(dist=NONE)	intergenic	HIVID	Yang et al .2018	30271481	Tumor
chr17	22255128	908	MTRNR2L1(dist=231137),NONE(dist=NONE)	intergenic	HIVID	Yang et al .2018	30271481	Tumor
chr17	22257506	908	MTRNR2L1(dist=233515),NONE(dist=NONE)	intergenic	HIVID	Yang et al .2018	30271481	Tumor
chr17	22259885	908	MTRNR2L1(dist=235894),NONE(dist=NONE)	intergenic	HIVID	Yang et al .2018	30271481	Tumor
chr17	22262262	908	MTRNR2L1(dist=238271),NONE(dist=NONE)	intergenic	HIVID	Yang et al .2018	30271481	Tumor
chr17	35340571	2713	AATF	intronic	HIVID	Yang et al .2018	30271481	Tumor
chr17	53816104	26	TMEM100(dist=6622),PCTP(dist=12236)	intergenic	HIVID	Yang et al .2018	30271481	Tumor
chr17	81195037	2588	FLJ43681(dist=6464),NONE(dist=NONE)	intergenic	HIVID	Yang et al .2018	30271481	Tumor
chr18	38870375	1819	LINC00669(dist=1538416),KC6(dist=189861)	intergenic	HIVID	Yang et al .2018	30271481	Tumor
chr18	47105443	2641	LIPG	intronic	HIVID	Yang et al .2018	30271481	Tumor
chr18	64326428	683	CDH19(dist=55212),MIR5011(dist=422393)	intergenic	HIVID	Yang et al .2018	30271481	Tumor
chr18	64326464	1029	CDH19(dist=55248),MIR5011(dist=422357)	intergenic	HIVID	Yang et al .2018	30271481	Tumor
chr18	74439180	26	LOC284276(dist=167396),LOC100131655(dist=67508)	intergenic	HIVID	Yang et al .2018	30271481	Tumor
chr18	78016181	2588	PARD6G(dist=10784),NONE(dist=NONE)	intergenic	HIVID	Yang et al .2018	30271481	Tumor
chr18	78016252	2588	PARD6G(dist=10855),NONE(dist=NONE)	intergenic	HIVID	Yang et al .2018	30271481	Tumor
chr19	36212557	1808	KMT2B	exonic	HIVID	Yang et al .2018	30271481	Tumor
chr2	33141539	2661	LINC00486	ncRNA_intronic	HIVID	Yang et al .2018	30271481	Tumor
chr2	75737187	1731	FAM176A	intronic	HIVID	Yang et al .2018	30271481	Tumor
chr2	76456581	395	GCFC2(dist=518470),LRRTM4(dist=518269)	intergenic	HIVID	Yang et al .2018	30271481	Tumor
chr2	131587996	1828	FAM123C(dist=62289),ARHGEF4(dist=86228)	intergenic	HIVID	Yang et al .2018	30271481	Tumor
chr2	134837554	2065	NCKAP5(dist=511523),MIR3679(dist=47142)	intergenic	HIVID	Yang et al .2018	30271481	Tumor
chr2	134840933	1825	NCKAP5(dist=514902),MIR3679(dist=43763)	intergenic	HIVID	Yang et al .2018	30271481	Tumor
chr2	139629976	2588	NXPH2(dist=92165),LOC647012(dist=24918)	intergenic	HIVID	Yang et al .2018	30271481	Tumor
chr2	159124706	1042	CCDC148	intronic	HIVID	Yang et al .2018	30271481	Tumor
chr2	159277733	2876	CCDC148	intronic	HIVID	Yang et al .2018	30271481	Tumor
chr2	161162554	26	RBMS1	intronic	HIVID	Yang et al .2018	30271481	Tumor
chr2	184735556	2851	NUP35(dist=709148),ZNF804A(dist=727537)	intergenic	HIVID	Yang et al .2018	30271481	Tumor
chr2	184735599	2799	NUP35(dist=709191),ZNF804A(dist=727494)	intergenic	HIVID	Yang et al .2018	30271481	Tumor
chr20	29829484	268	MLLT10P1(dist=191346),DEFB115(dist=15983)	intergenic	HIVID	Yang et al .2018	30271481	Tumor
chr20	52923426	1790	PFDN4(dist=86934),DOK5(dist=168840)	intergenic	HIVID	Yang et al .2018	30271481	Tumor
chr21	10782265	2160	TEKT4P2(dist=813672),TPTE(dist=124478)	intergenic	HIVID	Yang et al .2018	30271481	Tumor
chr21	10819677	268	TEKT4P2(dist=851084),TPTE(dist=87066)	intergenic	HIVID	Yang et al .2018	30271481	Tumor
chr21	48119785	2588	PRMT2(dist=34630),NONE(dist=NONE)	intergenic	HIVID	Yang et al .2018	30271481	Tumor
chr22	34438884	1639	LARGE(dist=122468),ISX(dist=1023246)	intergenic	HIVID	Yang et al .2018	30271481	Tumor
chr22	50745111	2588	PLXNB2	intronic	HIVID	Yang et al .2018	30271481	Tumor
chr3	9305491	344	SRGAP3(dist=14122),THUMPD3(dist=99226)	intergenic	HIVID	Yang et al .2018	30271481	Tumor
chr3	48770891	2588	IP6K2(dist=16180),PRKAR2A(dist=17202)	intergenic	HIVID	Yang et al .2018	30271481	Tumor
chr3	105794626	2588	CBLB(dist=206739),LOC100302640(dist=1034011)	intergenic	HIVID	Yang et al .2018	30271481	Tumor
chr3	110552275	344	FLJ25363(dist=1338261),PVRL3-AS1(dist=211888)	intergenic	HIVID	Yang et al .2018	30271481	Tumor
chr3	110721031	344	FLJ25363(dist=1507017),PVRL3-AS1(dist=43132)	intergenic	HIVID	Yang et al .2018	30271481	Tumor
chr3	119284914	2132	CD80(dist=6433),ADPRH(dist=13609)	intergenic	HIVID	Yang et al .2018	30271481	Tumor
chr3	150539704	2767	SIAH2(dist=58441),CLRN1(dist=104246)	intergenic	HIVID	Yang et al .2018	30271481	Tumor
chr3	159430419	26	IQCJ-SCHIP1,SCHIP1	intronic	HIVID	Yang et al .2018	30271481	Tumor
chr3	183063795	344	MCF2L2	intronic	HIVID	Yang et al .2018	30271481	Tumor
chr3	185685568	1639	LOC344887	ncRNA_intronic	HIVID	Yang et al .2018	30271481	Tumor
chr3	186667311	454	ST6GAL1	intronic	HIVID	Yang et al .2018	30271481	Tumor
chr3	188803523	1818	LPP(dist=195063),TPRG1(dist=86240)	intergenic	HIVID	Yang et al .2018	30271481	Tumor
chr3	188803536	1816	LPP(dist=195076),TPRG1(dist=86227)	intergenic	HIVID	Yang et al .2018	30271481	Tumor
chr4	9671023	1707	MIR548I2(dist=113086),DRD5(dist=112235)	intergenic	HIVID	Yang et al .2018	30271481	Tumor
chr4	14723923	340	LOC152742(dist=582247),LOC441009(dist=187662)	intergenic	HIVID	Yang et al .2018	30271481	Tumor
chr4	29692228	1125	MIR4275(dist=870938),PCDH7(dist=1029802)	intergenic	HIVID	Yang et al .2018	30271481	Tumor

Supplementary Table S8 Continued

Chromosome	Integration site in host genome	Integration site in virus genome	Gene (distance, bp)	Regions	Methods	Author	PMID	Sample
chr4	30910391	344	PCDH7	intronic	HIVID	Yang et al .2018	30271481	Tumor
chr4	40802613	1639	NSUN7	intronic	HIVID	Yang et al .2018	30271481	Tumor
chr4	49110232	2160	CWH43(dist=46137),NONE(dist=NONE)	intergenic	HIVID	Yang et al .2018	30271481	Tumor
chr4	49123316	226	CWH43(dist=59221),NONE(dist=NONE)	intergenic	HIVID	Yang et al .2018	30271481	Tumor
chr4	49125552	226	CWH43(dist=61457),NONE(dist=NONE)	intergenic	HIVID	Yang et al .2018	30271481	Tumor
chr4	49130903	261	CWH43(dist=66808),NONE(dist=NONE)	intergenic	HIVID	Yang et al .2018	30271481	Tumor
chr4	49132813	226	CWH43(dist=68718),NONE(dist=NONE)	intergenic	HIVID	Yang et al .2018	30271481	Tumor
chr4	49146656	226	CWH43(dist=82561),NONE(dist=NONE)	intergenic	HIVID	Yang et al .2018	30271481	Tumor
chr4	49152483	352	CWH43(dist=88388),NONE(dist=NONE)	intergenic	HIVID	Yang et al .2018	30271481	Tumor
chr4	49639349	2160	CWH43(dist=575254),NONE(dist=NONE)	intergenic	HIVID	Yang et al .2018	30271481	Tumor
chr4	56895380	588	CEP135	intronic	HIVID	Yang et al .2018	30271481	Tumor
chr4	59148534	1835	LOC255130(dist=1077069),NONE(dist=NONE)	intergenic	HIVID	Yang et al .2018	30271481	Tumor
chr4	59148781	2744	LOC255130(dist=1077316),NONE(dist=NONE)	intergenic	HIVID	Yang et al .2018	30271481	Tumor
chr4	67590201	2075	LOC100144602(dist=1031097),CENPC1(dist=747788)	intergenic	HIVID	Yang et al .2018	30271481	Tumor
chr4	86160931	294	WDFY3-AS2(dist=232763),ARHGAP24(dist=235353)	intergenic	HIVID	Yang et al .2018	30271481	Tumor
chr4	101839948	2876	EMCN-IT3(dist=243678),PPP3CA(dist=104639)	intergenic	HIVID	Yang et al .2018	30271481	Tumor
chr4	107197646	26	TBCK	intronic	HIVID	Yang et al .2018	30271481	Tumor
chr4	108333992	344	DKK2(dist=376539),PAPSS1(dist=200830)	intergenic	HIVID	Yang et al .2018	30271481	Tumor
chr4	166602912	970	CPE(dist=183430),TLL1(dist=191498)	intergenic	HIVID	Yang et al .2018	30271481	Tumor
chr4	191043645	2588	DUX4L2(dist=30169),NONE(dist=NONE)	intergenic	HIVID	Yang et al .2018	30271481	Tumor
chr5	10358	2622	NONE(dist=NONE),PLEKHG4B(dist=130015)	intergenic	HIVID	Yang et al .2018	30271481	Tumor
chr5	10906	2728	NONE(dist=NONE),PLEKHG4B(dist=129467)	intergenic	HIVID	Yang et al .2018	30271481	Tumor
chr5	11424	2593	NONE(dist=NONE),PLEKHG4B(dist=128949)	intergenic	HIVID	Yang et al .2018	30271481	Tumor
chr5	1295699	1813	TERT	upstream	HIVID	Yang et al .2018	30271481	Tumor
chr5	1295777	1690	TERT	upstream	HIVID	Yang et al .2018	30271481	Tumor
chr5	1296148	986	TERT	upstream	HIVID	Yang et al .2018	30271481	Tumor
chr5	1297992	1758	TERT(dist=2830),MIR4457(dist=11433)	intergenic	HIVID	Yang et al .2018	30271481	Tumor
chr5	11282985	2416	CTNND2	intronic	HIVID	Yang et al .2018	30271481	Tumor
chr5	70758495	26	BDP1	intronic	HIVID	Yang et al .2018	30271481	Tumor
chr5	86979812	254	CCNH(dist=270962),TMEM161B(dist=511211)	intergenic	HIVID	Yang et al .2018	30271481	Tumor
chr5	119483046	2588	FAM170A(dist=511529),PRR16(dist=316973)	intergenic	HIVID	Yang et al .2018	30271481	Tumor
chr5	131852154	2876	IRF1(dist=25689),IL5(dist=24982)	intergenic	HIVID	Yang et al .2018	30271481	Tumor
chr5	146518108	344	PPP2R2B(dist=57075),STK32A(dist=96471)	intergenic	HIVID	Yang et al .2018	30271481	Tumor
chr5	152135092	339	NMUR2(dist=350252),GRIA1(dist=734992)	intergenic	HIVID	Yang et al .2018	30271481	Tumor
chr6	8390785	26	EEF1E1-MUTED(dist=287957),SLC35B3(dist=22516)	intergenic	HIVID	Yang et al .2018	30271481	Tumor
chr6	45364247	26	RUNX2	intronic	HIVID	Yang et al .2018	30271481	Tumor
chr6	69247639	344	NONE(dist=NONE),BAI3(dist=97993)	intergenic	HIVID	Yang et al .2018	30271481	Tumor
chr6	78455320	2876	HTR1B(dist=282200),IRAK1BP1(dist=1121869)	intergenic	HIVID	Yang et al .2018	30271481	Tumor
chr6	154477153	1433	IPCEF1	UTR3	HIVID	Yang et al .2018	30271481	Tumor
chr6	163098968	1145	PARK2	intronic	HIVID	Yang et al .2018	30271481	Tumor
chr6	167831104	1828	TCP10(dist=33106),C6orf123(dist=354115)	intergenic	HIVID	Yang et al .2018	30271481	Tumor
chr7	5371978	26	TNRC18	intronic	HIVID	Yang et al .2018	30271481	Tumor
chr7	35742815	1639	HERPUD2(dist=8043),SEPT7(dist=97781)	intergenic	HIVID	Yang et al .2018	30271481	Tumor
chr7	63378784	1639	MIR4283-1(dist=297237),LOC100506050(dist=106012)	intergenic	HIVID	Yang et al .2018	30271481	Tumor
chr7	66338486	1819	GTF2IRD1P1(dist=28673),TMEM248(dist=47717)	intergenic	HIVID	Yang et al .2018	30271481	Tumor
chr7	74403506	26	GATSL1	intronic	HIVID	Yang et al .2018	30271481	Tumor
chr7	74842895	26	GATSL2	intronic	HIVID	Yang et al .2018	30271481	Tumor
chr7	88909205	2917	ZNF804B	intronic	HIVID	Yang et al .2018	30271481	Tumor
chr7	99848755	26	GATS	intronic	HIVID	Yang et al .2018	30271481	Tumor
chr7	139016688	1819	UBN2(dist=23706),LUC7L2(dist=8417)	intergenic	HIVID	Yang et al .2018	30271481	Tumor
chr8	51787482	301	SNTG1(dist=82055),PXDNL(dist=444655)	intergenic	HIVID	Yang et al .2018	30271481	Tumor
chr8	55893017	340	RP1(dist=349623),XKR4(dist=122000)	intergenic	HIVID	Yang et al .2018	30271481	Tumor
chr8	56808182	72	LYN	intronic	HIVID	Yang et al .2018	30271481	Tumor
chr8	85317713	2132	RALYL	intronic	HIVID	Yang et al .2018	30271481	Tumor
chr9	10101	2588	NONE(dist=NONE),DDX11L5(dist=1886)	intergenic	HIVID	Yang et al .2018	30271481	Tumor
chr9	15835680	1639	CCDC171	intronic	HIVID	Yang et al .2018	30271481	Tumor
chr9	39427950	2876	FAM75A2(dist=65991),LOC653501(dist=15864)	intergenic	HIVID	Yang et al .2018	30271481	Tumor
chr9	39957192	2876	FAM74A1(dist=49952),FAM75A3(dist=743099)	intergenic	HIVID	Yang et al .2018	30271481	Tumor
chr9	68666323	2876	LOC642236(dist=211948),LOC100132352(dist=60218)	intergenic	HIVID	Yang et al .2018	30271481	Tumor
chr9	68991641	2876	LOC100132352(dist=243269),PGM5P2(dist=88603)	intergenic	HIVID	Yang et al .2018	30271481	Tumor
chr9	69005344	2876	LOC100132352(dist=256972),PGM5P2(dist=74900)	intergenic	HIVID	Yang et al .2018	30271481	Tumor
chr9	88991916	1639	ZCCHC6(dist=22514),GAS1(dist=567361)	intergenic	HIVID	Yang et al .2018	30271481	Tumor
chr9	90374888	2611	CTSL1(dist=28504),CTSL3(dist=12942)	intergenic	HIVID	Yang et al .2018	30271481	Tumor
chrX	80945600	2876	SH3BGRL(dist=391554),POU3F4(dist=1817669)	intergenic	HIVID	Yang et al .2018	30271481	Tumor
chrX	101351750	1819	ZMAT1(dist=164711),TCEAL2(dist=28910)	intergenic	HIVID	Yang et al .2018	30271481	Tumor
chrX	154672583	1639	F8A1(dist=59133),F8A1(dist=13992)	intergenic	HIVID	Yang et al .2018	30271481	Tumor
chrX	155259868	2588	IL9R(dist=19386),NONE(dist=NONE)	intergenic	HIVID	Yang et al .2018	30271481	Tumor
chrX	155260281	1814	IL9R(dist=19799),NONE(dist=NONE)	intergenic	HIVID	Yang et al .2018	30271481	Tumor
chrY	13453683	226	NONE(dist=NONE),GYG2P1(dist=1064232)	intergenic	HIVID	Yang et al .2018	30271481	Tumor
chrY	13660286	226	NONE(dist=NONE),GYG2P1(dist=857629)	intergenic	HIVID	Yang et al .2018	30271481	Tumor
chrY	59362877	2588	IL9R(dist=19389),NONE(dist=NONE)	intergenic	HIVID	Yang et al .2018	30271481	Tumor
chrY	59363287	1814	IL9R(dist=19799),NONE(dist=NONE)	intergenic	HIVID	Yang et al .2018	30271481	Tumor
chr1	58357395	2479	DAB1	intronic	HIVID	Yang et al .2018	30271481	Tumor
chr12	33260405	2238	PKP2(dist=210625),SYT10(dist=267943)	intergenic	HIVID	Yang et al .2018	30271481	Tumor
chr15	71935941	2479	THSD4	intronic	HIVID	Yang et al .2018	30271481	Tumor
chr18	68015349	1577	SOCS6(dist=17915),LOC100505776(dist=1171851)	intergenic	HIVID	Yang et al .2018	30271481	Tumor
chr18	68015361	2238	SOCS6(dist=17927),LOC100505776(dist=1171839)	intergenic	HIVID	Yang et al .2018	30271481	Tumor
chr18	68382509	2238	SOCS6(dist=385075),LOC100505776(dist=804691)	intergenic	HIVID	Yang et al .2018	30271481	Tumor
chr19	41419547	2238	CYP2G1P(dist=13134),CYP2B7P1(dist=10623)	intergenic	HIVID	Yang et al .2018	30271481	Tumor
chr4	171375349	2827	AADAT(dist=363977),HSP90AA6P(dist=127272)	intergenic	HIVID	Yang et al .2018	30271481	Tumor
chr4	171375356	2479	AADAT(dist=363984),HSP90AA6P(dist=127265)	intergenic	HIVID	Yang et al .2018	30271481	Tumor
chr5	1295173	1818	TERT	upstream	HIVID	Yang et al .2018	30271481	Tumor
chr5	1295177	1834	TERT	upstream	HIVID	Yang et al .2018	30271481	Tumor
chr5	75093621	1577	POC5(dist=80308),SV2C(dist=285684)	intergenic	HIVID	Yang et al .2018	30271481	Tumor
chr5	169247619	1513	DOCK2	intronic	HIVID	Yang et al .2018	30271481	Tumor
chr6	49244387	2238	PTCHD4(dist=1207962),MUT(dist=153686)	intergenic	HIVID	Yang et al .2018	30271481	Tumor
chrX	82542286	1513	SH3BGRL(dist=1988240),POU3F4(dist=220983)	intergenic	HIVID	Yang et al .2018	30271481	Tumor
chrX	83811534	1577	HDX(dist=54047),UBE2DNL(dist=377623)	intergenic	HIVID	Yang et al .2018	30271481	Tumor
chr1	20310425	1820	PLA2G2A(dist=3493),PLA2G5(dist=86276)	intergenic	HIVID	Yang et al .2018	30271481	Tumor
chr1	73986024	3087	NEGR1(dist=1237747),LRRIQ3(dist=505678)	intergenic	HIVID	Yang et al .2018	30271481	Tumor
chr10	18474633	1103	CACNB2	intronic	HIVID	Yang et al .2018	30271481	Tumor
chr10	88512743	1837	LDB3(dist=16919),BMPR1A(dist=3653)	intergenic	HIVID	Yang et al .2018	30271481	Tumor
chr10	88512748	2325	LDB3(dist=16924),BMPR1A(dist=3648)	intergenic	HIVID	Yang et al .2018	30271481	Tumor
chr11	30193847	1574	KCNA4(dist=155270),FSHB(dist=58716)	intergenic	HIVID	Yang et al .2018	30271481	Tumor

Chromosome	Integration site in host genome	Integration site in virus genome	Gene (distance, bp)	Regions	Methods	Author	PMID	Sample
chr11	51569918	1381	OR4C46(dist=53707),NONE(dist=NONE)	intergenic	HIVID	Yang et al .2018	30271481	Tumor
chr11	101819952	2060	KIAA1377	intronic	HIVID	Yang et al .2018	30271481	Tumor
chr11	101819954	1776	KIAA1377	intronic	HIVID	Yang et al .2018	30271481	Tumor
chr12	66451373	325	HMGA2(dist=91302),LLPH(dist=65476)	intergenic	HIVID	Yang et al .2018	30271481	Tumor
chr12	113319973	2189	RPH3A	intronic	HIVID	Yang et al .2018	30271481	Tumor
chr13	49171849	2310	LINC00462(dist=16812),CYSLTR2(dist=109102)	intergenic	HIVID	Yang et al .2018	30271481	Tumor
chr13	49171923	2310	LINC00462(dist=16886),CYSLTR2(dist=109028)	intergenic	HIVID	Yang et al .2018	30271481	Tumor
chr13	49171925	1823	LINC00462(dist=16888),CYSLTR2(dist=109026)	intergenic	HIVID	Yang et al .2018	30271481	Tumor
chr14	81014676	1821	CEP128	intronic	HIVID	Yang et al .2018	30271481	Tumor
chr14	81239081	1828	CEP128	intronic	HIVID	Yang et al .2018	30271481	Tumor
chr14	81239082	1828	CEP128	intronic	HIVID	Yang et al .2018	30271481	Tumor
chr14	81239159	1828	CEP128	intronic	HIVID	Yang et al .2018	30271481	Tumor
chr15	22333066	1836	LOC727924	ncRNA_intronic	HIVID	Yang et al .2018	30271481	Tumor
chr16	67709429	1041	GFOD2	exonic	HIVID	Yang et al .2018	30271481	Tumor
chr17	22244633	1381	MTRNR2L1(dist=220642),NONE(dist=NONE)	intergenic	HIVID	Yang et al .2018	30271481	Tumor
chr17	22247012	1381	MTRNR2L1(dist=223021),NONE(dist=NONE)	intergenic	HIVID	Yang et al .2018	30271481	Tumor
chr17	22249391	1381	MTRNR2L1(dist=225400),NONE(dist=NONE)	intergenic	HIVID	Yang et al .2018	30271481	Tumor
chr17	22251767	1381	MTRNR2L1(dist=227776),NONE(dist=NONE)	intergenic	HIVID	Yang et al .2018	30271481	Tumor
chr17	22254144	1387	MTRNR2L1(dist=230153),NONE(dist=NONE)	intergenic	HIVID	Yang et al .2018	30271481	Tumor
chr17	22256525	1381	MTRNR2L1(dist=232534),NONE(dist=NONE)	intergenic	HIVID	Yang et al .2018	30271481	Tumor
chr17	22261282	1381	MTRNR2L1(dist=237291),NONE(dist=NONE)	intergenic	HIVID	Yang et al .2018	30271481	Tumor
chr19	36212541	1548	KMT2B	exonic	HIVID	Yang et al .2018	30271481	Tumor
chr19	36212557	1808	KMT2B	exonic	HIVID	Yang et al .2018	30271481	Tumor
chr2	32151376	2794	MEMO1	intronic	HIVID	Yang et al .2018	30271481	Tumor
chr2	99818030	1010	MRPL30(dist=2010),LYG2(dist=40681)	intergenic	HIVID	Yang et al .2018	30271481	Tumor
chr2	199332341	1816	PLCL1(dist=317733),SATB2(dist=801882)	intergenic	HIVID	Yang et al .2018	30271481	Tumor
chr20	11097293	3170	JAG1(dist=442599),LOC339593(dist=150014)	intergenic	HIVID	Yang et al .2018	30271481	Tumor
chr3	183311251	2504	KLHL6(dist=37752),KLHL24(dist=42160)	intergenic	HIVID	Yang et al .2018	30271481	Tumor
chr5	1295580	2373	TERT	upstream	HIVID	Yang et al .2018	30271481	Tumor
chr5	1295701	1813	TERT	upstream	HIVID	Yang et al .2018	30271481	Tumor
chr5	1295777	1691	TERT	upstream	HIVID	Yang et al .2018	30271481	Tumor
chr5	1297259	70	TERT(dist=2097),MIR4457(dist=12166)	intergenic	HIVID	Yang et al .2018	30271481	Tumor
chr5	1297377	2977	TERT(dist=2215),MIR4457(dist=12048)	intergenic	HIVID	Yang et al .2018	30271481	Tumor
chr5	1297427	1693	TERT(dist=2265),MIR4457(dist=11998)	intergenic	HIVID	Yang et al .2018	30271481	Tumor
chr5	28755322	3170	LOC643401(dist=1258814),LSP1P3(dist=171655)	intergenic	HIVID	Yang et al .2018	30271481	Tumor
chr5	86979814	256	CCNH(dist=270964),TMEM161B(dist=511209)	intergenic	HIVID	Yang et al .2018	30271481	Tumor
chr6	23484937	1752	HDGFL1(dist=914187),NRSN1(dist=641477)	intergenic	HIVID	Yang et al .2018	30271481	Tumor
chr6	23484945	1666	HDGFL1(dist=914195),NRSN1(dist=641469)	intergenic	HIVID	Yang et al .2018	30271481	Tumor
chr6	23484988	1752	HDGFL1(dist=914238),NRSN1(dist=641426)	intergenic	HIVID	Yang et al .2018	30271481	Tumor
chr6	96604293	1811	FUT9	intronic	HIVID	Yang et al .2018	30271481	Tumor
chr6	96604358	2236	FUT9	intronic	HIVID	Yang et al .2018	30271481	Tumor
chr6	107485919	3170	PDSS2	intronic	HIVID	Yang et al .2018	30271481	Tumor
chr8	46849785	1667	NONE(dist=NONE),LINC00293(dist=902723)	intergenic	HIVID	Yang et al .2018	30271481	Tumor
chr8	46853522	1667	NONE(dist=NONE),LINC00293(dist=898986)	intergenic	HIVID	Yang et al .2018	30271481	Tumor
chr8	46855391	1667	NONE(dist=NONE),LINC00293(dist=897117)	intergenic	HIVID	Yang et al .2018	30271481	Tumor
chr8	51787482	301	SNTG1(dist=82055),PXDNL(dist=444655)	intergenic	HIVID	Yang et al .2018	30271481	Tumor
chr9	21143529	3170	IFNW1(dist=1385),IFNA21(dist=22107)	intergenic	HIVID	Yang et al .2018	30271481	Tumor
chr9	28282816	3182	LINGO2	intronic	HIVID	Yang et al .2018	30271481	Tumor
chr9	28282879	209	LINGO2	intronic	HIVID	Yang et al .2018	30271481	Tumor
chr9	28282905	45	LINGO2	intronic	HIVID	Yang et al .2018	30271481	Tumor
chr9	28283144	3096	LINGO2	intronic	HIVID	Yang et al .2018	30271481	Tumor
chr9	69005344	2876	LOC100132352(dist=256972),PGM5P2(dist=74900)	intergenic	HIVID	Yang et al .2018	30271481	Tumor
chr9	125301942	3170	OR1N1(dist=12370),OR1N2(dist=13507)	intergenic	HIVID	Yang et al .2018	30271481	Tumor
chr1	10330	660	NONE(dist=NONE),DDX11L1(dist=1544)	intergenic	HIVID	Yang et al .2018	30271481	Tumor
chr1	195244486	46	NONE(dist=NONE),KCNT2(dist=950427)	intergenic	HIVID	Yang et al .2018	30271481	Tumor
chr1	249240260	660	PGBD2(dist=26915),NONE(dist=NONE)	intergenic	HIVID	Yang et al .2018	30271481	Tumor
chr12	95545	660	LOC100288778(dist=4282),FAM138D(dist=52401)	intergenic	HIVID	Yang et al .2018	30271481	Tumor
chr12	133841512	559	ANHX(dist=29090),NONE(dist=NONE)	intergenic	HIVID	Yang et al .2018	30271481	Tumor
chr13	105083096	938	MIR548AS(dist=1148248),DAOA-AS1(dist=1028310)	intergenic	HIVID	Yang et al .2018	30271481	Tumor
chr15	28031185	550	OCA2	intronic	HIVID	Yang et al .2018	30271481	Tumor
chr15	90454089	1901	C15orf38,C15orf38-AP3S2	intronic	HIVID	Yang et al .2018	30271481	Tumor
chr17	14984304	2352	CDRT7(dist=49030),PMP22(dist=148792)	intergenic	HIVID	Yang et al .2018	30271481	Tumor
chr17	21795139	2354	C17orf51(dist=340198),FAM27L(dist=30231)	intergenic	HIVID	Yang et al .2018	30271481	Tumor
chr17	21811071	2162	C17orf51(dist=356130),FAM27L(dist=14299)	intergenic	HIVID	Yang et al .2018	30271481	Tumor
chr18	10007	660	NONE(dist=NONE),ROCK1P1(dist=99058)	intergenic	HIVID	Yang et al .2018	30271481	Tumor
chr18	18929616	2755	GREB1L	intronic	HIVID	Yang et al .2018	30271481	Tumor
chr19	55522530	562	NLRP2(dist=10020),GP6(dist=2545)	intergenic	HIVID	Yang et al .2018	30271481	Tumor
chr2	31653109	537	XDH(dist=15498),SRD5A2(dist=96547)	intergenic	HIVID	Yang et al .2018	30271481	Tumor
chr2	227552439	211	MIR5702(dist=28930),IRS1(dist=43594)	intergenic	HIVID	Yang et al .2018	30271481	Tumor
chr3	197900821	660	FAM157A	intronic	HIVID	Yang et al .2018	30271481	Tumor
chr4	9671023	1707	MIR548I2(dist=113086),DRD5(dist=112235)	intergenic	HIVID	Yang et al .2018	30271481	Tumor
chr4	11721365	2416	HS3ST1(dist=290828),HSP90AB2P(dist=1613672)	intergenic	HIVID	Yang et al .2018	30271481	Tumor
chr4	67590201	2075	LOC100144602(dist=1031097),CENPC1(dist=747788)	intergenic	HIVID	Yang et al .2018	30271481	Tumor
chr4	111684152	3110	PITX2(dist=120873),C4orf32(dist=1382401)	intergenic	HIVID	Yang et al .2018	30271481	Tumor
chr4	166602912	970	CPE(dist=183430),TLL1(dist=191498)	intergenic	HIVID	Yang et al .2018	30271481	Tumor
chr5	10358	521	NONE(dist=NONE),PLEKHG4B(dist=130015)	intergenic	HIVID	Yang et al .2018	30271481	Tumor
chr5	10976	574	NONE(dist=NONE),PLEKHG4B(dist=129397)	intergenic	HIVID	Yang et al .2018	30271481	Tumor
chr5	1295257	453	TERT	upstream	HIVID	Yang et al .2018	30271481	Tumor
chr5	1295521	1622	TERT	upstream	HIVID	Yang et al .2018	30271481	Tumor
chr5	11282463	1404	CTNND2	intronic	HIVID	Yang et al .2018	30271481	Tumor
chr5	11282976	1495	CTNND2	intronic	HIVID	Yang et al .2018	30271481	Tumor
chr5	11282982	2417	CTNND2	intronic	HIVID	Yang et al .2018	30271481	Tumor
chr6	136321985	3110	PDE7B	intronic	HIVID	Yang et al .2018	30271481	Tumor
chr8	5481710	151	CSMD1(dist=629382),LOC100287015(dist=779367)	intergenic	HIVID	Yang et al .2018	30271481	Tumor
chr8	51787482	301	SNTG1(dist=82055),PXDNL(dist=444655)	intergenic	HIVID	Yang et al .2018	30271481	Tumor
chr8	56808182	72	LYN	intronic	HIVID	Yang et al .2018	30271481	Tumor
chr8	82681089	3170	CHMP4C(dist=9341),SNX16(dist=30729)	intergenic	HIVID	Yang et al .2018	30271481	Tumor
chr8	85533031	47	RALYL	intronic	HIVID	Yang et al .2018	30271481	Tumor
chr8	85533166	3110	RALYL	intronic	HIVID	Yang et al .2018	30271481	Tumor
chr8	107565242	1934	OXR1	intronic	HIVID	Yang et al .2018	30271481	Tumor
chrX	155260406	660	IL9R(dist=19924),NONE(dist=NONE)	intergenic	HIVID	Yang et al .2018	30271481	Tumor
chrY	59363255	660	IL9R(dist=19767),NONE(dist=NONE)	intergenic	HIVID	Yang et al .2018	30271481	Tumor
chrY	59363363	660	IL9R(dist=19875),NONE(dist=NONE)	intergenic	HIVID	Yang et al .2018	30271481	Tumor
chr1	163823903	1347	NUF2(dist=498350),PBX1(dist=704694)	intergenic	HIVID	Yang et al .2018	30271481	Tumor

Chromosome	Integration site in host genome	Integration site in virus genome	Gene (distance, bp)	Regions	Methods	Author	PMID	Sample
chr10	313210	2630	ZMYND11(dist=12633),DIP2C(dist=6920)	intergenic	HIVID	Yang et al .2018	30271481	Tumor
chr10	25098085	1827	ARHGAP21(dist=85488),PRTFDC1(dist=39469)	intergenic	HIVID	Yang et al .2018	30271481	Tumor
chr11	29761177	1827	METTL15(dist=1406123),KCNA4(dist=270111)	intergenic	HIVID	Yang et al .2018	30271481	Tumor
chr11	69593071	1347	FGF4(dist=2900),FGF3(dist=31665)	intergenic	HIVID	Yang et al .2018	30271481	Tumor
chr11	69608587	1846	FGF4(dist=18416),FGF3(dist=16149)	intergenic	HIVID	Yang et al .2018	30271481	Tumor
chr11	70704028	1745	SHANK2	intronic	HIVID	Yang et al .2018	30271481	Tumor
chr12	2250290	439	CACNA1C	intronic	HIVID	Yang et al .2018	30271481	Tumor
chr12	7604957	3063	CD163L1(dist=8208),CD163(dist=18455)	intergenic	HIVID	Yang et al .2018	30271481	Tumor
chr12	27236556	2364	C12orf71(dist=1101),STK38L(dist=160522)	intergenic	HIVID	Yang et al .2018	30271481	Tumor
chr12	29031735	730	CCDC91(dist=328636),FAR2(dist=344863)	intergenic	HIVID	Yang et al .2018	30271481	Tumor
chr13	68377564	1345	PCDH9(dist=573096),LINC00550(dist=1057853)	intergenic	HIVID	Yang et al .2018	30271481	Tumor
chr15	71883384	1751	THSD4	intronic	HIVID	Yang et al .2018	30271481	Tumor
chr17	21476919	357	C17orf51(dist=21978),FAM27L(dist=348451)	intergenic	HIVID	Yang et al .2018	30271481	Tumor
chr19	36212557	1808	KMT2B	exonic	HIVID	Yang et al .2018	30271481	Tumor
chr19	36212812	1554	KMT2B	intronic	HIVID	Yang et al .2018	30271481	Tumor
chr19	36212884	1554	KMT2B	intronic	HIVID	Yang et al .2018	30271481	Tumor
chr19	36213177	1612	KMT2B	intronic	HIVID	Yang et al .2018	30271481	Tumor
chr19	38664508	1061	SIPA1L3	intronic	HIVID	Yang et al .2018	30271481	Tumor
chr19	56045075	1827	SBK2	intronic	HIVID	Yang et al .2018	30271481	Tumor
chr2	37598606	1827	QPCT	intronic	HIVID	Yang et al .2018	30271481	Tumor
chr21	26795083	1817	LINC00158	ncRNA_intronic	HIVID	Yang et al .2018	30271481	Tumor
chr5	1294821	1019	TERT	intronic	HIVID	Yang et al .2018	30271481	Tumor
chr5	159223206	2190	LOC285627(dist=329922),ADRA1B(dist=120534)	intergenic	HIVID	Yang et al .2018	30271481	Tumor
chr5	159223282	1135	LOC285627(dist=329998),ADRA1B(dist=120458)	intergenic	HIVID	Yang et al .2018	30271481	Tumor
chr6	42673725	1827	PRPH2	intronic	HIVID	Yang et al .2018	30271481	Tumor
chr6	64647234	1827	EYS	intronic	HIVID	Yang et al .2018	30271481	Tumor
chr6	76245908	1826	FILIP1(dist=42412),SENP6(dist=65714)	intergenic	HIVID	Yang et al .2018	30271481	Tumor
chr6	126943747	1713	CENPW(dist=273993),RSPO3(dist=496301)	intergenic	HIVID	Yang et al .2018	30271481	Tumor
chr6	154477153	1433	IPCEF1	UTR3	HIVID	Yang et al .2018	30271481	Tumor
chr7	91360044	1827	FZD1(dist=461912),MTERF(dist=141977)	intergenic	HIVID	Yang et al .2018	30271481	Tumor
chr8	5472557	2775	CSMD1(dist=620229),LOC100287015(dist=788520)	intergenic	HIVID	Yang et al .2018	30271481	Tumor
chr8	51787482	301	SNTG1(dist=82055),PXDNL(dist=444655)	intergenic	HIVID	Yang et al .2018	30271481	Tumor
chr8	76265793	1827	CRISPLD1(dist=319000),HNF4G(dist=186410)	intergenic	HIVID	Yang et al .2018	30271481	Tumor
chr8	85998755	1827	RALYL(dist=164677),LRRCC1(dist=20568)	intergenic	HIVID	Yang et al .2018	30271481	Tumor
chr8	129524908	1621	MIR1208(dist=362474),LOC728724(dist=703805)	intergenic	HIVID	Yang et al .2018	30271481	Tumor
chrX	92751201	1827	PCDH11X(dist=872973),NAP1L3(dist=174724)	intergenic	HIVID	Yang et al .2018	30271481	Tumor
chrY	25207765	1789	BPY2(dist=56155),DAZ1(dist=67737)	intergenic	HIVID	Yang et al .2018	30271481	Tumor
chr15	53654809	1069	ONECUT1(dist=572600),WDR72(dist=151129)	intergenic	HIVID	Yang et al .2018	30271481	Tumor
chr15	53654812	1828	ONECUT1(dist=572603),WDR72(dist=151126)	intergenic	HIVID	Yang et al .2018	30271481	Tumor
chr17	21082020	389	DHRS7B	intronic	HIVID	Yang et al .2018	30271481	Tumor
chr17	21082649	2231	DHRS7B	intronic	HIVID	Yang et al .2018	30271481	Tumor
chr17	21476919	357	C17orf51(dist=21978),FAM27L(dist=348451)	intergenic	HIVID	Yang et al .2018	30271481	Tumor
chr17	21909505	357	FLJ36000	ncRNA_exonic	HIVID	Yang et al .2018	30271481	Tumor
chr2	201722174	1795	CLK1	intronic	HIVID	Yang et al .2018	30271481	Tumor
chr2	201722184	1461	CLK1	intronic	HIVID	Yang et al .2018	30271481	Tumor
chr20	7363610	1656	BMP2(dist=602700),HAO1(dist=500021)	intergenic	HIVID	Yang et al .2018	30271481	Tumor
chr3	44902044	2878	KIF15(dist=7296),MIR564(dist=1336)	intergenic	HIVID	Yang et al .2018	30271481	Tumor
chr3	44902079	2439	KIF15(dist=7331),MIR564(dist=1301)	intergenic	HIVID	Yang et al .2018	30271481	Tumor
chr5	159223282	1135	LOC285627(dist=329998),ADRA1B(dist=120458)	intergenic	HIVID	Yang et al .2018	30271481	Tumor
chr10	59579762	2354	MIR3924(dist=515443),IPMK(dist=371516)	intergenic	HIVID	Yang et al .2018	30271481	Tumor
chr12	133841764	193	ANHX(dist=29342),NONE(dist=NONE)	intergenic	HIVID	Yang et al .2018	30271481	Tumor
chr2	243152475	193	LOC728323(dist=50006),NONE(dist=NONE)	intergenic	HIVID	Yang et al .2018	30271481	Tumor
chr7	129434110	994	MIR183(dist=19256),UBE2H(dist=36463)	intergenic	HIVID	Yang et al .2018	30271481	Tumor
chr7	129434139	2490	MIR183(dist=19285),UBE2H(dist=36434)	intergenic	HIVID	Yang et al .2018	30271481	Tumor
chr1	6122149	1750	KCNAB2	intronic	HIVID	Yang et al .2018	30271481	Tumor
chr1	37987332	1704	MEAF6(dist=6912),SNIP1(dist=12718)	intergenic	HIVID	Yang et al .2018	30271481	Tumor
chr1	83043455	2848	LPHN2(dist=585348),MIR548AP(dist=1216143)	intergenic	HIVID	Yang et al .2018	30271481	Tumor
chr1	83043578	2298	LPHN2(dist=585471),MIR548AP(dist=1216020)	intergenic	HIVID	Yang et al .2018	30271481	Tumor
chr1	91395708	3110	ZNF644	intronic	HIVID	Yang et al .2018	30271481	Tumor
chr1	154418139	1293	IL6R	intronic	HIVID	Yang et al .2018	30271481	Tumor
chr1	224412652	1818	DEGS1(dist=31510),NVL(dist=2384)	intergenic	HIVID	Yang et al .2018	30271481	Tumor
chr1	225667672	147	LBR(dist=51115),ENAH(dist=6862)	intergenic	HIVID	Yang et al .2018	30271481	Tumor
chr1	225667787	3110	LBR(dist=51230),ENAH(dist=6747)	intergenic	HIVID	Yang et al .2018	30271481	Tumor
chr1	225667795	1450	LBR(dist=51238),ENAH(dist=6739)	intergenic	HIVID	Yang et al .2018	30271481	Tumor
chr10	71354850	1445	NEUROG3(dist=21640),C10orf35(dist=35153)	intergenic	HIVID	Yang et al .2018	30271481	Tumor
chr11	692716	3110	DEAF1	intronic	HIVID	Yang et al .2018	30271481	Tumor
chr11	61092485	3110	DDB1	intronic	HIVID	Yang et al .2018	30271481	Tumor
chr11	65756076	1750	SART1(dist=8469),EIF1AD(dist=7940)	intergenic	HIVID	Yang et al .2018	30271481	Tumor
chr11	76067913	1750	PRKRIR	intronic	HIVID	Yang et al .2018	30271481	Tumor
chr11	125229809	1750	PKNOX2	intronic	HIVID	Yang et al .2018	30271481	Tumor
chr12	17767965	1811	SKP1P2(dist=624403),MIR3974(dist=58268)	intergenic	HIVID	Yang et al .2018	30271481	Tumor
chr12	50549756	1813	CERS5	intronic	HIVID	Yang et al .2018	30271481	Tumor
chr12	58340431	1750	XRCC6BP1	intronic	HIVID	Yang et al .2018	30271481	Tumor
chr12	105469187	1638	ALDH1L2	intronic	HIVID	Yang et al .2018	30271481	Tumor
chr13	45107792	1454	TSC22D1	intronic	HIVID	Yang et al .2018	30271481	Tumor
chr14	34947137	1750	SPTSSA(dist=15669),EAPP(dist=37998)	intergenic	HIVID	Yang et al .2018	30271481	Tumor
chr14	52349641	1293	GNG2	intronic	HIVID	Yang et al .2018	30271481	Tumor
chr14	57379448	1750	OTX2-AS1	ncRNA_intronic	HIVID	Yang et al .2018	30271481	Tumor
chr14	60366926	3110	RTN1(dist=29369),PCNXL4(dist=191703)	intergenic	HIVID	Yang et al .2018	30271481	Tumor
chr15	57677617	1293	CGNL1	intronic	HIVID	Yang et al .2018	30271481	Tumor
chr15	59493797	1750	MYO1E	intronic	HIVID	Yang et al .2018	30271481	Tumor
chr15	71551279	1750	THSD4	intronic	HIVID	Yang et al .2018	30271481	Tumor
chr15	89339130	1750	ISG20(dist=140251),ACAN(dist=7544)	intergenic	HIVID	Yang et al .2018	30271481	Tumor
chr16	3032502	14	PKMYT1(dist=1962),LINC00514(dist=6553)	intergenic	HIVID	Yang et al .2018	30271481	Tumor
chr16	5807377	1818	FAM86A(dist=659588),RBFOX1(dist=261755)	intergenic	HIVID	Yang et al .2018	30271481	Tumor
chr17	2129346	1750	SMG6	intronic	HIVID	Yang et al .2018	30271481	Tumor
chr17	47767022	1387	SPOP(dist=11497),SLC35B1(dist=11668)	intergenic	HIVID	Yang et al .2018	30271481	Tumor
chr19	6281498	2288	MLLT1(dist=1539),ACER1(dist=25012)	intergenic	HIVID	Yang et al .2018	30271481	Tumor
chr19	6284230	1293	MLLT1(dist=4271),ACER1(dist=22280)	intergenic	HIVID	Yang et al .2018	30271481	Tumor
chr19	6284258	1222	MLLT1(dist=4299),ACER1(dist=22252)	intergenic	HIVID	Yang et al .2018	30271481	Tumor
chr19	6284259	1293	MLLT1(dist=4300),ACER1(dist=22251)	intergenic	HIVID	Yang et al .2018	30271481	Tumor
chr19	44118903	3110	ZNF428	intronic	HIVID	Yang et al .2018	30271481	Tumor
chr19	46036558	1293	OPA3	intronic	HIVID	Yang et al .2018	30271481	Tumor
chr19	58451472	1631	ZNF256	downstream	HIVID	Yang et al .2018	30271481	Tumor

Chromosome	Integration site in host genome	Integration site in virus genome	Gene (distance, bp)	Regions	Methods	Author	PMID	Sample
chr2	59362361	1417	FLJ30838(dist=71460),MIR4432(dist=1252136)	intergenic	HIVID	Yang et al .2018	30271481	Tumor
chr2	152688151	3110	ARL5A(dist=3142),CACNB4(dist=1135)	intergenic	HIVID	Yang et al .2018	30271481	Tumor
chr20	33262879	1750	PIGU	intronic	HIVID	Yang et al .2018	30271481	Tumor
chr20	62420362	1750	ZBTB46	intronic	HIVID	Yang et al .2018	30271481	Tumor
chr22	47185316	1747	TBC1D22A	intronic	HIVID	Yang et al .2018	30271481	Tumor
chr3	101964847	1750	LOC152225(dist=248077),ZPLD1(dist=189012)	intergenic	HIVID	Yang et al .2018	30271481	Tumor
chr3	111493984	2239	PHLDB2,PLCXD2	intronic	HIVID	Yang et al .2018	30271481	Tumor
chr3	147755351	1112	ZIC1(dist=620845),AGTR1(dist=660307)	intergenic	HIVID	Yang et al .2018	30271481	Tumor
chr4	122741124	3130	CCNA2	intronic	HIVID	Yang et al .2018	30271481	Tumor
chr4	122741337	2662	CCNA2	intronic	HIVID	Yang et al .2018	30271481	Tumor
chr4	122741372	2544	CCNA2	intronic	HIVID	Yang et al .2018	30271481	Tumor
chr4	152075480	1750	SH3D19	intronic	HIVID	Yang et al .2018	30271481	Tumor
chr5	31498741	1750	DROSHA	intronic	HIVID	Yang et al .2018	30271481	Tumor
chr5	139675344	2634	PFDN1	intronic	HIVID	Yang et al .2018	30271481	Tumor
chr5	139784647	2092	ANKHD1,ANKHD1-EIF4EBP3	intronic	HIVID	Yang et al .2018	30271481	Tumor
chr5	139784760	3100	ANKHD1,ANKHD1-EIF4EBP3	intronic	HIVID	Yang et al .2018	30271481	Tumor
chr5	139962269	1818	SLC35A4(dist=13586),CD14(dist=49044)	intergenic	HIVID	Yang et al .2018	30271481	Tumor
chr5	161785596	1750	GABRG2(dist=203051),CCNG1(dist=1078981)	intergenic	HIVID	Yang et al .2018	30271481	Tumor
chr6	22283576	1730	LINC00340(dist=88960),PRL(dist=3897)	intergenic	HIVID	Yang et al .2018	30271481	Tumor
chr6	36588046	1818	SRSF3(dist=15802),MIR3925(dist=2167)	intergenic	HIVID	Yang et al .2018	30271481	Tumor
chr6	36693705	46	RAB44	exonic	HIVID	Yang et al .2018	30271481	Tumor
chr7	20677235	1750	ABCB5	intronic	HIVID	Yang et al .2018	30271481	Tumor
chr8	19001803	1750	PSD3(dist=130607),LOC100128993(dist=39383)	intergenic	HIVID	Yang et al .2018	30271481	Tumor
chr8	88048735	1293	CNBD1	intronic	HIVID	Yang et al .2018	30271481	Tumor
chr8	95721859	1488	ESRP1(dist=2165),DPY19L4(dist=10244)	intergenic	HIVID	Yang et al .2018	30271481	Tumor
chr9	75563798	1309	ALDH1A1	intronic	HIVID	Yang et al .2018	30271481	Tumor
chr9	75743294	2720	ALDH1A1(dist=175061),ANXA1(dist=23487)	intergenic	HIVID	Yang et al .2018	30271481	Tumor
chrX	16722893	1749	CTPS2	intronic	HIVID	Yang et al .2018	30271481	Tumor
chrX	27130491	1750	VENTXP1(dist=551322),SMEK3P(dist=347837)	intergenic	HIVID	Yang et al .2018	30271481	Tumor
chrX	70599160	1750	BCYRN1	ncRNA_intronic	HIVID	Yang et al .2018	30271481	Tumor
chr11	133315069	1881	OPCML	intronic	HIVID	Yang et al .2018	30271481	Tumor
chr2	198973674	1371	PLCL1	intronic	HIVID	Yang et al .2018	30271481	Tumor
chr4	61450046	992	NONE(dist=NONE),LPHN3(dist=912793)	intergenic	HIVID	Yang et al .2018	30271481	Tumor
chr5	1295801	506	TERT	upstream	HIVID	Yang et al .2018	30271481	Tumor
chr5	108077029	1765	HP07349(dist=13067),FER(dist=6494)	intergenic	HIVID	Yang et al .2018	30271481	Tumor
chr1	6761116	2055	DNAJC11	intronic	HIVID	Yang et al .2018	30271481	Tumor
chr1	185664789	285	LOC100288079(dist=360618),HMCN1(dist=38894)	intergenic	HIVID	Yang et al .2018	30271481	Tumor
chr1	185674893	1832	LOC100288079(dist=370722),HMCN1(dist=28790)	intergenic	HIVID	Yang et al .2018	30271481	Tumor
chr1	241741599	521	KMO	intronic	HIVID	Yang et al .2018	30271481	Tumor
chr10	42385614	1769	NONE(dist=NONE),LOC441666(dist=441700)	intergenic	HIVID	Yang et al .2018	30271481	Tumor
chr10	42387747	1652	NONE(dist=NONE),LOC441666(dist=439567)	intergenic	HIVID	Yang et al .2018	30271481	Tumor
chr10	42389385	1769	NONE(dist=NONE),LOC441666(dist=437929)	intergenic	HIVID	Yang et al .2018	30271481	Tumor
chr10	42393009	1816	NONE(dist=NONE),LOC441666(dist=434305)	intergenic	HIVID	Yang et al .2018	30271481	Tumor
chr10	42528075	168	NONE(dist=NONE),LOC441666(dist=299239)	intergenic	HIVID	Yang et al .2018	30271481	Tumor
chr10	42596705	1769	NONE(dist=NONE),LOC441666(dist=230609)	intergenic	HIVID	Yang et al .2018	30271481	Tumor
chr10	42598063	1816	NONE(dist=NONE),LOC441666(dist=229251)	intergenic	HIVID	Yang et al .2018	30271481	Tumor
chr10	88512748	2325	LDB3(dist=16924),BMPR1A(dist=3648)	intergenic	HIVID	Yang et al .2018	30271481	Tumor
chr10	88541379	234	BMPR1A	intronic	HIVID	Yang et al .2018	30271481	Tumor
chr12	50261607	1263	FAIM2	UTR3	HIVID	Yang et al .2018	30271481	Tumor
chr12	88431782	2920	C12orf29	intronic	HIVID	Yang et al .2018	30271481	Tumor
chr12	94998719	1776	TMCC3	intronic	HIVID	Yang et al .2018	30271481	Tumor
chr12	113319973	2189	RPH3A	intronic	HIVID	Yang et al .2018	30271481	Tumor
chr14	78981579	1828	NRXN3	intronic	HIVID	Yang et al .2018	30271481	Tumor
chr15	90475471	1815	C15orf38-AP3S2(dist=19249),ZNF710(dist=69281)	intergenic	HIVID	Yang et al .2018	30271481	Tumor
chr16	46392239	3201	NONE(dist=NONE),ANKRD26P1(dist=111010)	intergenic	HIVID	Yang et al .2018	30271481	Tumor
chr16	46398075	3201	NONE(dist=NONE),ANKRD26P1(dist=105174)	intergenic	HIVID	Yang et al .2018	30271481	Tumor
chr16	46404180	3201	NONE(dist=NONE),ANKRD26P1(dist=99069)	intergenic	HIVID	Yang et al .2018	30271481	Tumor
chr16	46427808	3201	NONE(dist=NONE),ANKRD26P1(dist=75441)	intergenic	HIVID	Yang et al .2018	30271481	Tumor
chr16	46428618	3201	NONE(dist=NONE),ANKRD26P1(dist=74631)	intergenic	HIVID	Yang et al .2018	30271481	Tumor
chr18	32989120	2127	ZNF396(dist=31819),INO80C(dist=59171)	intergenic	HIVID	Yang et al .2018	30271481	Tumor
chr18	71132732	984	LOC100505817(dist=115608),FBXO15(dist=607856)	intergenic	HIVID	Yang et al .2018	30271481	Tumor
chr18	71276484	968	LOC100505817(dist=259360),FBXO15(dist=464104)	intergenic	HIVID	Yang et al .2018	30271481	Tumor
chr19	36212541	1548	KMT2B	exonic	HIVID	Yang et al .2018	30271481	Tumor
chr19	36212557	1731	KMT2B	exonic	HIVID	Yang et al .2018	30271481	Tumor
chr19	36212557	1808	KMT2B	exonic	HIVID	Yang et al .2018	30271481	Tumor
chr2	99818030	1010	MRPL30(dist=2010),LYG2(dist=40681)	intergenic	HIVID	Yang et al .2018	30271481	Tumor
chr2	99818050	1793	MRPL30(dist=2030),LYG2(dist=40661)	intergenic	HIVID	Yang et al .2018	30271481	Tumor
chr2	112980705	942	ZC3H8	intronic	HIVID	Yang et al .2018	30271481	Tumor
chr2	171274834	1799	MYO3B	intronic	HIVID	Yang et al .2018	30271481	Tumor
chr2	171274932	1828	MYO3B	intronic	HIVID	Yang et al .2018	30271481	Tumor
chr20	30564103	1895	XKR7	intronic	HIVID	Yang et al .2018	30271481	Tumor
chr22	49082817	1917	FAM19A5	intronic	HIVID	Yang et al .2018	30271481	Tumor
chr3	145454372	2370	C3orf58(dist=1743162),PLOD2(dist=332856)	intergenic	HIVID	Yang et al .2018	30271481	Tumor
chr4	9671023	1656	MIR548I2(dist=113086),DRD5(dist=112235)	intergenic	HIVID	Yang et al .2018	30271481	Tumor
chr4	166602909	970	CPE(dist=183427),TLL1(dist=191501)	intergenic	HIVID	Yang et al .2018	30271481	Tumor
chr5	129935404	418	CHSY3(dist=413077),HINT1(dist=559471)	intergenic	HIVID	Yang et al .2018	30271481	Tumor
chr5	168125130	397	SLIT3	intronic	HIVID	Yang et al .2018	30271481	Tumor
chr6	154477145	1432	IPCEF1	UTR3	HIVID	Yang et al .2018	30271481	Tumor
chr6	154477401	2793	IPCEF1	UTR3	HIVID	Yang et al .2018	30271481	Tumor
chr7	76154878	3173	UPK3B	intronic	HIVID	Yang et al .2018	30271481	Tumor
chr8	51787482	301	SNTG1(dist=82055),PXDNL(dist=444655)	intergenic	HIVID	Yang et al .2018	30271481	Tumor
chr8	97587829	1546	SDC2	intronic	HIVID	Yang et al .2018	30271481	Tumor
chr8	97587863	1757	SDC2	intronic	HIVID	Yang et al .2018	30271481	Tumor
chr8	129524908	1621	MIR1208(dist=362474),LOC728724(dist=703805)	intergenic	HIVID	Yang et al .2018	30271481	Tumor
chrX	136312869	1029	GPR101(dist=199036),ZIC3(dist=335477)	intergenic	HIVID	Yang et al .2018	30271481	Tumor
chr1	58802824	1826	DAB1(dist=86613),OMA1(dist=143567)	intergenic	HIVID	Yang et al .2018	30271481	Tumor
chr1	121485034	2909	EMBP1(dist=171348),NONE(dist=NONE)	intergenic	HIVID	Yang et al .2018	30271481	Tumor
chr10	313210	2630	ZMYND11(dist=12633),DIP2C(dist=6920)	intergenic	HIVID	Yang et al .2018	30271481	Tumor
chr10	30503326	1826	KIAA1462(dist=154838),MTPAP(dist=95404)	intergenic	HIVID	Yang et al .2018	30271481	Tumor
chr10	88512743	1837	LDB3(dist=16919),BMPR1A(dist=3653)	intergenic	HIVID	Yang et al .2018	30271481	Tumor
chr10	97199706	1826	SORBS1	intronic	HIVID	Yang et al .2018	30271481	Tumor
chr10	131284589	1826	MGMT	intronic	HIVID	Yang et al .2018	30271481	Tumor
chr11	62368854	2323	MTA2	intronic	HIVID	Yang et al .2018	30271481	Tumor
chr11	104941699	2027	CARD16(dist=25648),CARD17(dist=21497)	intergenic	HIVID	Yang et al .2018	30271481	Tumor

Supplementary Table S8 Continued

Chromosome	Integration site in host genome	Integration site in virus genome	Gene (distance, bp)	Regions	Methods	Author	PMID	Sample
chr11	104941713	1827	CARD16(dist=25662),CARD17(dist=21483)	intergenic	HIVID	Yang et al .2018	30271481	Tumor
chr12	88431764	2911	C12orf29	intronic	HIVID	Yang et al .2018	30271481	Tumor
chr12	94998719	1776	TMCC3	intronic	HIVID	Yang et al .2018	30271481	Tumor
chr12	94998763	1986	TMCC3	intronic	HIVID	Yang et al .2018	30271481	Tumor
chr12	98192507	1826	MIR135A2(dist=234818),MIR4303(dist=196654)	intergenic	HIVID	Yang et al .2018	30271481	Tumor
chr13	68377564	1345	PCDH9(dist=573096),LINC00550(dist=1057853)	intergenic	HIVID	Yang et al .2018	30271481	Tumor
chr13	91024509	1826	MIR622(dist=140978),LINC00410(dist=518699)	intergenic	HIVID	Yang et al .2018	30271481	Tumor
chr15	70197771	1826	LINC00593(dist=62465),TLE3(dist=142772)	intergenic	HIVID	Yang et al .2018	30271481	Tumor
chr16	88812032	1826	PIEZO1	intronic	HIVID	Yang et al .2018	30271481	Tumor
chr17	19490804	2511	SLC47A1(dist=8458),ALDH3A2(dist=61260)	intergenic	HIVID	Yang et al .2018	30271481	Tumor
chr17	19494011	1776	SLC47A1(dist=11665),ALDH3A2(dist=58053)	intergenic	HIVID	Yang et al .2018	30271481	Tumor
chr17	22251282	1560	MTRNR2L1(dist=227291),NONE(dist=NONE)	intergenic	HIVID	Yang et al .2018	30271481	Tumor
chr18	18517135	2418	NONE(dist=NONE),ROCK1(dist=12568)	intergenic	HIVID	Yang et al .2018	30271481	Tumor
chr18	18519012	2402	NONE(dist=NONE),ROCK1(dist=10691)	intergenic	HIVID	Yang et al .2018	30271481	Tumor
chr18	21968645	1826	OSBPL1A	intronic	HIVID	Yang et al .2018	30271481	Tumor
chr19	21788223	1823	ZNF429(dist=67144),ZNF100(dist=118620)	intergenic	HIVID	Yang et al .2018	30271481	Tumor
chr19	36212406	1943	KMT2B	exonic	HIVID	Yang et al .2018	30271481	Tumor
chr19	36212557	1808	KMT2B	exonic	HIVID	Yang et al .2018	30271481	Tumor
chr19	36212659	2196	KMT2B	exonic	HIVID	Yang et al .2018	30271481	Tumor
chr19	36213045	1800	KMT2B	intronic	HIVID	Yang et al .2018	30271481	Tumor
chr2	110746661	729	LOC440894	ncRNA_intronic	HIVID	Yang et al .2018	30271481	Tumor
chr2	117934434	1821	DPP10(dist=1332108),DDX18(dist=637821)	intergenic	HIVID	Yang et al .2018	30271481	Tumor
chr21	17327777	1826	USP25(dist=75400),LINC00478(dist=115065)	intergenic	HIVID	Yang et al .2018	30271481	Tumor
chr3	123112869	1826	ADCY5	intronic	HIVID	Yang et al .2018	30271481	Tumor
chr4	9671023	1707	MIR548I2(dist=113086),DRD5(dist=112235)	intergenic	HIVID	Yang et al .2018	30271481	Tumor
chr4	36772300	2813	DTHD1(dist=424922),MIR4801(dist=471232)	intergenic	HIVID	Yang et al .2018	30271481	Tumor
chr4	55722219	1769	KIT(dist=115338),KDR(dist=222207)	intergenic	HIVID	Yang et al .2018	30271481	Tumor
chr4	67589460	2649	LOC100144602(dist=1030356),CENPC1(dist=748529)	intergenic	HIVID	Yang et al .2018	30271481	Tumor
chr4	67590201	2075	LOC100144602(dist=1031097),CENPC1(dist=747788)	intergenic	HIVID	Yang et al .2018	30271481	Tumor
chr4	90194719	1826	GPRIN3	intronic	HIVID	Yang et al .2018	30271481	Tumor
chr4	191043654	718	DUX4L2(dist=30178),NONE(dist=NONE)	intergenic	HIVID	Yang et al .2018	30271481	Tumor
chr5	22193069	331	CDH12	intronic	HIVID	Yang et al .2018	30271481	Tumor
chr5	40723848	1826	TTC33	intronic	HIVID	Yang et al .2018	30271481	Tumor
chr5	86979814	256	CCNH(dist=270964),TMEM161B(dist=511209)	intergenic	HIVID	Yang et al .2018	30271481	Tumor
chr5	124121771	1826	ZNF608(dist=40966),GRAMD3(dist=1574017)	intergenic	HIVID	Yang et al .2018	30271481	Tumor
chr5	126841358	1826	MEGF10(dist=44448),PRRC1(dist=11951)	intergenic	HIVID	Yang et al .2018	30271481	Tumor
chr5	134767201	1821	H2AFY(dist=31624),C5orf20(dist=12703)	intergenic	HIVID	Yang et al .2018	30271481	Tumor
chr6	138933128	1826	NHSL1(dist=39460),FLJ46906(dist=79677)	intergenic	HIVID	Yang et al .2018	30271481	Tumor
chr6	150467497	500	PPP1R14C	intronic	HIVID	Yang et al .2018	30271481	Tumor
chr7	42204743	1826	GLI3	intronic	HIVID	Yang et al .2018	30271481	Tumor
chr7	55833926	1826	FKBP9L(dist=61666),SEPT14(dist=27311)	intergenic	HIVID	Yang et al .2018	30271481	Tumor
chr8	3420958	2170	CSMD1	intronic	HIVID	Yang et al .2018	30271481	Tumor
chr8	7382935	2465	DEFB107B(dist=16102),FAM90A7P(dist=30725)	intergenic	HIVID	Yang et al .2018	30271481	Tumor
chr8	7653028	2369	FAM90A10P(dist=24193),DEFB107A(dist=16214)	intergenic	HIVID	Yang et al .2018	30271481	Tumor
chr8	81137526	1826	TPD52(dist=53690),MIR5708(dist=16098)	intergenic	HIVID	Yang et al .2018	30271481	Tumor
chrY	2484820	2131	CD99P1	ncRNA_intronic	HIVID	Yang et al .2018	30271481	Tumor
chrY	58820000	2776	NONE(dist=NONE),SPRY3(dist=280457)	intergenic	HIVID	Yang et al .2018	30271481	Tumor
chrY	58822105	2402	NONE(dist=NONE),SPRY3(dist=278352)	intergenic	HIVID	Yang et al .2018	30271481	Tumor
chrY	58823588	2776	NONE(dist=NONE),SPRY3(dist=276869)	intergenic	HIVID	Yang et al .2018	30271481	Tumor
chrY	58825663	2402	NONE(dist=NONE),SPRY3(dist=274794)	intergenic	HIVID	Yang et al .2018	30271481	Tumor
chrY	58827351	2807	NONE(dist=NONE),SPRY3(dist=273106)	intergenic	HIVID	Yang et al .2018	30271481	Tumor
chrY	58829227	2402	NONE(dist=NONE),SPRY3(dist=271230)	intergenic	HIVID	Yang et al .2018	30271481	Tumor
chrY	58832816	2402	NONE(dist=NONE),SPRY3(dist=267641)	intergenic	HIVID	Yang et al .2018	30271481	Tumor
chrY	58836350	2402	NONE(dist=NONE),SPRY3(dist=264107)	intergenic	HIVID	Yang et al .2018	30271481	Tumor
chrY	58837937	2793	NONE(dist=NONE),SPRY3(dist=262520)	intergenic	HIVID	Yang et al .2018	30271481	Tumor
chrY	58839819	2402	NONE(dist=NONE),SPRY3(dist=260638)	intergenic	HIVID	Yang et al .2018	30271481	Tumor
chrY	58843403	2402	NONE(dist=NONE),SPRY3(dist=257054)	intergenic	HIVID	Yang et al .2018	30271481	Tumor
chrY	58845159	2839	NONE(dist=NONE),SPRY3(dist=255298)	intergenic	HIVID	Yang et al .2018	30271481	Tumor
chrY	58846973	2402	NONE(dist=NONE),SPRY3(dist=253484)	intergenic	HIVID	Yang et al .2018	30271481	Tumor
chrY	58848684	2835	NONE(dist=NONE),SPRY3(dist=251773)	intergenic	HIVID	Yang et al .2018	30271481	Tumor
chrY	58850567	2402	NONE(dist=NONE),SPRY3(dist=249890)	intergenic	HIVID	Yang et al .2018	30271481	Tumor
chrY	58852106	2801	NONE(dist=NONE),SPRY3(dist=248351)	intergenic	HIVID	Yang et al .2018	30271481	Tumor
chrY	58854134	2410	NONE(dist=NONE),SPRY3(dist=246323)	intergenic	HIVID	Yang et al .2018	30271481	Tumor
chrY	58855697	2839	NONE(dist=NONE),SPRY3(dist=244760)	intergenic	HIVID	Yang et al .2018	30271481	Tumor
chrY	58857692	2402	NONE(dist=NONE),SPRY3(dist=242765)	intergenic	HIVID	Yang et al .2018	30271481	Tumor
chrY	58859417	2793	NONE(dist=NONE),SPRY3(dist=241040)	intergenic	HIVID	Yang et al .2018	30271481	Tumor
chrY	58861224	2402	NONE(dist=NONE),SPRY3(dist=239233)	intergenic	HIVID	Yang et al .2018	30271481	Tumor
chrY	58864851	2402	NONE(dist=NONE),SPRY3(dist=235606)	intergenic	HIVID	Yang et al .2018	30271481	Tumor
chrY	58866374	2834	NONE(dist=NONE),SPRY3(dist=234083)	intergenic	HIVID	Yang et al .2018	30271481	Tumor
chrY	58866454	2793	NONE(dist=NONE),SPRY3(dist=234003)	intergenic	HIVID	Yang et al .2018	30271481	Tumor
chrY	58868307	2412	NONE(dist=NONE),SPRY3(dist=232150)	intergenic	HIVID	Yang et al .2018	30271481	Tumor
chrY	58870181	2793	NONE(dist=NONE),SPRY3(dist=230276)	intergenic	HIVID	Yang et al .2018	30271481	Tumor
chrY	58872019	2402	NONE(dist=NONE),SPRY3(dist=228438)	intergenic	HIVID	Yang et al .2018	30271481	Tumor
chrY	58873755	2777	NONE(dist=NONE),SPRY3(dist=226702)	intergenic	HIVID	Yang et al .2018	30271481	Tumor
chrY	58875563	2402	NONE(dist=NONE),SPRY3(dist=224894)	intergenic	HIVID	Yang et al .2018	30271481	Tumor
chrY	58877152	2854	NONE(dist=NONE),SPRY3(dist=223305)	intergenic	HIVID	Yang et al .2018	30271481	Tumor
chrY	58879147	2402	NONE(dist=NONE),SPRY3(dist=221310)	intergenic	HIVID	Yang et al .2018	30271481	Tumor
chrY	58880631	2776	NONE(dist=NONE),SPRY3(dist=219826)	intergenic	HIVID	Yang et al .2018	30271481	Tumor
chrY	58882701	2402	NONE(dist=NONE),SPRY3(dist=217756)	intergenic	HIVID	Yang et al .2018	30271481	Tumor
chrY	58884398	2793	NONE(dist=NONE),SPRY3(dist=216059)	intergenic	HIVID	Yang et al .2018	30271481	Tumor
chrY	58886235	2402	NONE(dist=NONE),SPRY3(dist=214222)	intergenic	HIVID	Yang et al .2018	30271481	Tumor
chrY	58889819	2402	NONE(dist=NONE),SPRY3(dist=210638)	intergenic	HIVID	Yang et al .2018	30271481	Tumor
chrY	58891546	2793	NONE(dist=NONE),SPRY3(dist=208911)	intergenic	HIVID	Yang et al .2018	30271481	Tumor
chrY	58893393	2402	NONE(dist=NONE),SPRY3(dist=207064)	intergenic	HIVID	Yang et al .2018	30271481	Tumor
chrY	58894986	2793	NONE(dist=NONE),SPRY3(dist=205471)	intergenic	HIVID	Yang et al .2018	30271481	Tumor
chrY	58896977	2402	NONE(dist=NONE),SPRY3(dist=203480)	intergenic	HIVID	Yang et al .2018	30271481	Tumor
chrY	58898431	2776	NONE(dist=NONE),SPRY3(dist=202026)	intergenic	HIVID	Yang et al .2018	30271481	Tumor
chrY	58900536	2402	NONE(dist=NONE),SPRY3(dist=199921)	intergenic	HIVID	Yang et al .2018	30271481	Tumor
chrY	58902123	2793	NONE(dist=NONE),SPRY3(dist=198334)	intergenic	HIVID	Yang et al .2018	30271481	Tumor
chrY	58904097	2402	NONE(dist=NONE),SPRY3(dist=196360)	intergenic	HIVID	Yang et al .2018	30271481	Tumor
chrY	58907673	2402	NONE(dist=NONE),SPRY3(dist=192784)	intergenic	HIVID	Yang et al .2018	30271481	Tumor
chrY	58909384	2818	NONE(dist=NONE),SPRY3(dist=191073)	intergenic	HIVID	Yang et al .2018	30271481	Tumor
chrY	58911257	2402	NONE(dist=NONE),SPRY3(dist=189200)	intergenic	HIVID	Yang et al .2018	30271481	Tumor
chrY	58912774	2793	NONE(dist=NONE),SPRY3(dist=187683)	intergenic	HIVID	Yang et al .2018	30271481	Tumor

Chromosome	Integration site in host genome	Integration site in virus genome	Gene (distance, bp)	Regions	Methods	Author	PMID	Sample
chrY	58913251	2402	NONE(dist=NONE),SPRY3(dist=187206)	intergenic	HIVID	Yang et al .2018	30271481	Tumor
chrY	58914987	2777	NONE(dist=NONE),SPRY3(dist=185470)	intergenic	HIVID	Yang et al .2018	30271481	Tumor
chrY	58916806	2402	NONE(dist=NONE),SPRY3(dist=183651)	intergenic	HIVID	Yang et al .2018	30271481	Tumor
chr19	36212554	2511	KMT2B	exonic	HIVID	Yang et al .2018	30271481	Tumor
chr19	36212932	3202	KMT2B	intronic	HIVID	Yang et al .2018	30271481	Tumor
chr11	176227	722	LOC100133161(dist=44307),SCGB1C1(dist=16853)	intergenic	HIVID	Yang et al .2018	30271481	Tumor
chr16	90188357	722	PRDM7(dist=46019),NONE(dist=NONE)	intergenic	HIVID	Yang et al .2018	30271481	Tumor
chr17	81195057	722	FLJ43681(dist=6484),NONE(dist=NONE)	intergenic	HIVID	Yang et al .2018	30271481	Tumor
chr20	36225761	2688	BLCAP(dist=69428),LINC00489(dist=21939)	intergenic	HIVID	Yang et al .2018	30271481	Tumor
chr3	197900838	722	FAM157A	intronic	HIVID	Yang et al .2018	30271481	Tumor
chr3	197900983	722	FAM157A	intronic	HIVID	Yang et al .2018	30271481	Tumor
chr4	191043654	722	DUX4L2(dist=30178),NONE(dist=NONE)	intergenic	HIVID	Yang et al .2018	30271481	Tumor
chr5	11561	722	NONE(dist=NONE),PLEKHG4B(dist=128812)	intergenic	HIVID	Yang et al .2018	30271481	Tumor
chr9	24035492	544	ELAVL2(dist=209429),TUSC1(dist=1640895)	intergenic	HIVID	Yang et al .2018	30271481	Tumor
chr11	11240917	1761	LOC729013(dist=340094),GALNTL4(dist=51504)	intergenic	HIVID	Yang et al .2018	30271481	Tumor
chr13	20961881	1474	GJB6(dist=155347),CRYL1(dist=15925)	intergenic	HIVID	Yang et al .2018	30271481	Tumor
chr2	164763558	882	FIGN(dist=171045),GRB14(dist=585765)	intergenic	HIVID	Yang et al .2018	30271481	Tumor
chr2	164763584	2662	FIGN(dist=171071),GRB14(dist=585739)	intergenic	HIVID	Yang et al .2018	30271481	Tumor
chr15	84297339	2041	SH3GL3(dist=9846),ADAMTSL3(dist=25499)	intergenic	HIVID	Yang et al .2018	30271481	Tumor
chr1	154460948	418	SHE	intronic	HIVID	Yang et al .2018	30271481	Tumor
chr1	185664788	283	LOC100288079(dist=360617),HMCN1(dist=38895)	intergenic	HIVID	Yang et al .2018	30271481	Tumor
chr1	185665193	3213	LOC100288079(dist=361022),HMCN1(dist=38490)	intergenic	HIVID	Yang et al .2018	30271481	Tumor
chr1	185674892	1832	LOC100288079(dist=370721),HMCN1(dist=28791)	intergenic	HIVID	Yang et al .2018	30271481	Tumor
chr1	186940136	234	PLA2G4A	intronic	HIVID	Yang et al .2018	30271481	Tumor
chr10	42383776	1769	NONE(dist=NONE),LOC441666(dist=443538)	intergenic	HIVID	Yang et al .2018	30271481	Tumor
chr10	42384489	1673	NONE(dist=NONE),LOC441666(dist=442825)	intergenic	HIVID	Yang et al .2018	30271481	Tumor
chr10	42385614	1769	NONE(dist=NONE),LOC441666(dist=441700)	intergenic	HIVID	Yang et al .2018	30271481	Tumor
chr10	42387166	1812	NONE(dist=NONE),LOC441666(dist=440148)	intergenic	HIVID	Yang et al .2018	30271481	Tumor
chr10	42387648	1816	NONE(dist=NONE),LOC441666(dist=439666)	intergenic	HIVID	Yang et al .2018	30271481	Tumor
chr10	42387707	1096	NONE(dist=NONE),LOC441666(dist=439607)	intergenic	HIVID	Yang et al .2018	30271481	Tumor
chr10	42387723	1816	NONE(dist=NONE),LOC441666(dist=439591)	intergenic	HIVID	Yang et al .2018	30271481	Tumor
chr10	42389382	1769	NONE(dist=NONE),LOC441666(dist=437932)	intergenic	HIVID	Yang et al .2018	30271481	Tumor
chr10	42391182	1769	NONE(dist=NONE),LOC441666(dist=436132)	intergenic	HIVID	Yang et al .2018	30271481	Tumor
chr10	42391832	1587	NONE(dist=NONE),LOC441666(dist=435482)	intergenic	HIVID	Yang et al .2018	30271481	Tumor
chr10	42393006	1812	NONE(dist=NONE),LOC441666(dist=434308)	intergenic	HIVID	Yang et al .2018	30271481	Tumor
chr10	42393113	1816	NONE(dist=NONE),LOC441666(dist=434201)	intergenic	HIVID	Yang et al .2018	30271481	Tumor
chr10	42394337	1591	NONE(dist=NONE),LOC441666(dist=432977)	intergenic	HIVID	Yang et al .2018	30271481	Tumor
chr10	42394884	1744	NONE(dist=NONE),LOC441666(dist=432430)	intergenic	HIVID	Yang et al .2018	30271481	Tumor
chr10	42396259	1816	NONE(dist=NONE),LOC441666(dist=431055)	intergenic	HIVID	Yang et al .2018	30271481	Tumor
chr10	42396651	1816	NONE(dist=NONE),LOC441666(dist=430663)	intergenic	HIVID	Yang et al .2018	30271481	Tumor
chr10	42596705	1769	NONE(dist=NONE),LOC441666(dist=230609)	intergenic	HIVID	Yang et al .2018	30271481	Tumor
chr10	42598063	1769	NONE(dist=NONE),LOC441666(dist=229251)	intergenic	HIVID	Yang et al .2018	30271481	Tumor
chr10	42599294	1789	NONE(dist=NONE),LOC441666(dist=228020)	intergenic	HIVID	Yang et al .2018	30271481	Tumor
chr10	42599896	1689	NONE(dist=NONE),LOC441666(dist=227418)	intergenic	HIVID	Yang et al .2018	30271481	Tumor
chr10	88512743	1837	LDB3(dist=16919),BMPR1A(dist=3653)	intergenic	HIVID	Yang et al .2018	30271481	Tumor
chr10	88541234	1575	BMPR1A	intronic	HIVID	Yang et al .2018	30271481	Tumor
chr10	88541300	234	BMPR1A	intronic	HIVID	Yang et al .2018	30271481	Tumor
chr10	88541372	234	BMPR1A	intronic	HIVID	Yang et al .2018	30271481	Tumor
chr11	14702850	1823	PDE3B	intronic	HIVID	Yang et al .2018	30271481	Tumor
chr11	30193847	1574	KCNA4(dist=155270),FSHB(dist=58716)	intergenic	HIVID	Yang et al .2018	30271481	Tumor
chr11	121351965	312	SORL1	intronic	HIVID	Yang et al .2018	30271481	Tumor
chr12	95473	1814	LOC100288778(dist=4210),FAM138D(dist=52473)	intergenic	HIVID	Yang et al .2018	30271481	Tumor
chr12	7854409	418	GDF3(dist=6049),DPPA3(dist=9680)	intergenic	HIVID	Yang et al .2018	30271481	Tumor
chr12	20691809	2713	PDE3A	intronic	HIVID	Yang et al .2018	30271481	Tumor
chr12	27236720	753	C12orf71(dist=1265),STK38L(dist=160358)	intergenic	HIVID	Yang et al .2018	30271481	Tumor
chr12	50261607	1263	FAIM2	UTR3	HIVID	Yang et al .2018	30271481	Tumor
chr12	66451373	561	HMGA2(dist=91302),LLPH(dist=65476)	intergenic	HIVID	Yang et al .2018	30271481	Tumor
chr12	88431764	2902	C12orf29	intronic	HIVID	Yang et al .2018	30271481	Tumor
chr12	113319973	2189	RPH3A	intronic	HIVID	Yang et al .2018	30271481	Tumor
chr13	35713412	418	NBEA	intronic	HIVID	Yang et al .2018	30271481	Tumor
chr13	79985459	418	RBM26-AS1	ncRNA_intronic	HIVID	Yang et al .2018	30271481	Tumor
chr13	80983773	418	SPRY2(dist=68687),NONE(dist=NONE)	intergenic	HIVID	Yang et al .2018	30271481	Tumor
chr14	76055036	418	FLVCR2	intronic	HIVID	Yang et al .2018	30271481	Tumor
chr15	90454180	1829	C15orf38,C15orf38-AP3S2	intronic	HIVID	Yang et al .2018	30271481	Tumor
chr15	90475471	1815	C15orf38-AP3S2(dist=19249),ZNF710(dist=69281)	intergenic	HIVID	Yang et al .2018	30271481	Tumor
chr16	11586597	2002	RMI2(dist=140980),LITAF(dist=54981)	intergenic	HIVID	Yang et al .2018	30271481	Tumor
chr16	17470489	418	XYLT1	intronic	HIVID	Yang et al .2018	30271481	Tumor
chr16	65514559	1178	LOC283867	ncRNA_intronic	HIVID	Yang et al .2018	30271481	Tumor
chr16	75368094	1814	CFDP1	intronic	HIVID	Yang et al .2018	30271481	Tumor
chr17	14984304	2354	CDRT7(dist=49030),PMP22(dist=148792)	intergenic	HIVID	Yang et al .2018	30271481	Tumor
chr17	22253631	1802	MTRNR2L1(dist=229640),NONE(dist=NONE)	intergenic	HIVID	Yang et al .2018	30271481	Tumor
chr17	22256010	1802	MTRNR2L1(dist=232019),NONE(dist=NONE)	intergenic	HIVID	Yang et al .2018	30271481	Tumor
chr17	22260767	1802	MTRNR2L1(dist=236776),NONE(dist=NONE)	intergenic	HIVID	Yang et al .2018	30271481	Tumor
chr18	9373331	418	TWSG1	intronic	HIVID	Yang et al .2018	30271481	Tumor
chr18	71132681	984	LOC100505817(dist=115557),FBXO15(dist=607907)	intergenic	HIVID	Yang et al .2018	30271481	Tumor
chr18	71276484	900	LOC100505817(dist=259360),FBXO15(dist=464104)	intergenic	HIVID	Yang et al .2018	30271481	Tumor
chr18	76503164	529	GALR1(dist=1521068),SALL3(dist=237111)	intergenic	HIVID	Yang et al .2018	30271481	Tumor
chr19	10522680	418	CDC37(dist=8409),PDE4A(dist=4769)	intergenic	HIVID	Yang et al .2018	30271481	Tumor
chr19	21788223	1765	ZNF429(dist=67144),ZNF100(dist=118620)	intergenic	HIVID	Yang et al .2018	30271481	Tumor
chr19	36212576	1639	KMT2B	exonic	HIVID	Yang et al .2018	30271481	Tumor
chr2	571243	234	FAM150B(dist=282935),TMEM18(dist=96730)	intergenic	HIVID	Yang et al .2018	30271481	Tumor
chr2	15517696	1640	NBAS	intronic	HIVID	Yang et al .2018	30271481	Tumor
chr2	75970633	234	GCFC2(dist=32522),LRRTM4(dist=1004217)	intergenic	HIVID	Yang et al .2018	30271481	Tumor
chr2	90374444	1816	MIR4436A(dist=1262476),LOC654342(dist=1450265)	intergenic	HIVID	Yang et al .2018	30271481	Tumor
chr2	99817323	1028	MRPL30(dist=1303),LYG2(dist=41388)	intergenic	HIVID	Yang et al .2018	30271481	Tumor
chr2	99817979	1010	MRPL30(dist=1959),LYG2(dist=40732)	intergenic	HIVID	Yang et al .2018	30271481	Tumor
chr2	99818049	1792	MRPL30(dist=2029),LYG2(dist=40662)	intergenic	HIVID	Yang et al .2018	30271481	Tumor
chr2	99818081	1414	MRPL30(dist=2061),LYG2(dist=40630)	intergenic	HIVID	Yang et al .2018	30271481	Tumor
chr2	153875776	984	ARL6IP6(dist=258009),RPRM(dist=458076)	intergenic	HIVID	Yang et al .2018	30271481	Tumor
chr20	30564095	1557	XKR7	intronic	HIVID	Yang et al .2018	30271481	Tumor
chr20	30564101	1808	XKR7	intronic	HIVID	Yang et al .2018	30271481	Tumor
chr21	35054518	816	ITSN1	intronic	HIVID	Yang et al .2018	30271481	Tumor
chr22	49082817	1917	FAM19A5	intronic	HIVID	Yang et al .2018	30271481	Tumor
chr22	49083132	1917	FAM19A5	intronic	HIVID	Yang et al .2018	30271481	Tumor

Chromosome	Integration site in host genome	Integration site in virus genome	Gene (distance, bp)	Regions	Methods	Author	PMID	Sample
chr22	50359905	2190	PIM3(dist=2185),IL17REL(dist=73037)	intergenic	HIVID	Yang et al .2018	30271481	Tumor
chr3	141188454	2436	ZBTB38(dist=19822),RASA2(dist=17472)	intergenic	HIVID	Yang et al .2018	30271481	Tumor
chr3	192952816	1040	MB21D2(dist=316866),HRASLS(dist=6101)	intergenic	HIVID	Yang et al .2018	30271481	Tumor
chr3	196625624	1776	SENP5	intronic	HIVID	Yang et al .2018	30271481	Tumor
chr4	9671023	1707	MIR548I2(dist=113086),DRD5(dist=112235)	intergenic	HIVID	Yang et al .2018	30271481	Tumor
chr4	15840844	454	CD38	intronic	HIVID	Yang et al .2018	30271481	Tumor
chr4	18491815	598	LCORL(dist=468332),SLIT2(dist=1763420)	intergenic	HIVID	Yang et al .2018	30271481	Tumor
chr4	43179316	1991	GRXCR1(dist=146641),KCTD8(dist=996604)	intergenic	HIVID	Yang et al .2018	30271481	Tumor
chr4	43198570	1582	GRXCR1(dist=165895),KCTD8(dist=977350)	intergenic	HIVID	Yang et al .2018	30271481	Tumor
chr4	56985044	1765	CEP135(dist=85515),KIAA1211(dist=51317)	intergenic	HIVID	Yang et al .2018	30271481	Tumor
chr4	166602910	970	CPE(dist=183428),TLL1(dist=191500)	intergenic	HIVID	Yang et al .2018	30271481	Tumor
chr5	33877302	3153	ADAMTS12	intronic	HIVID	Yang et al .2018	30271481	Tumor
chr5	37957331	1076	GDNF(dist=117549),EGFLAM(dist=301180)	intergenic	HIVID	Yang et al .2018	30271481	Tumor
chr5	75889910	2170	IQGAP2	intronic	HIVID	Yang et al .2018	30271481	Tumor
chr5	86979814	256	CCNH(dist=270964),TMEM161B(dist=511209)	intergenic	HIVID	Yang et al .2018	30271481	Tumor
chr5	129935404	418	CHSY3(dist=413077),HINT1(dist=559471)	intergenic	HIVID	Yang et al .2018	30271481	Tumor
chr5	129935910	492	CHSY3(dist=413583),HINT1(dist=558965)	intergenic	HIVID	Yang et al .2018	30271481	Tumor
chr5	180477474	1386	BTNL9	intronic	HIVID	Yang et al .2018	30271481	Tumor
chr6	76418415	418	SENP6	intronic	HIVID	Yang et al .2018	30271481	Tumor
chr7	53059953	2152	COBL(dist=1675438),POM121L12(dist=43396)	intergenic	HIVID	Yang et al .2018	30271481	Tumor
chr7	71295545	418	CALN1	intronic	HIVID	Yang et al .2018	30271481	Tumor
chr8	51787482	301	SNTG1(dist=82055),PXDNL(dist=444655)	intergenic	HIVID	Yang et al .2018	30271481	Tumor
chr9	28517157	2064	LINGO2	intronic	HIVID	Yang et al .2018	30271481	Tumor
chr9	31868613	234	LOC401497(dist=1460161),ACO1(dist=515988)	intergenic	HIVID	Yang et al .2018	30271481	Tumor
chrX	134778636	1739	DDX26B(dist=62176),CT45A1(dist=68549)	intergenic	HIVID	Yang et al .2018	30271481	Tumor
chrX	134778649	1821	DDX26B(dist=62189),CT45A1(dist=68536)	intergenic	HIVID	Yang et al .2018	30271481	Tumor
chrX	155260281	1814	IL9R(dist=19799),NONE(dist=NONE)	intergenic	HIVID	Yang et al .2018	30271481	Tumor
chrY	59363287	1814	IL9R(dist=19799),NONE(dist=NONE)	intergenic	HIVID	Yang et al .2018	30271481	Tumor
chr19	36212519	1731	KMT2B	exonic	HIVID	Yang et al .2018	30271481	Tumor
chr19	36212642	1665	KMT2B	exonic	HIVID	Yang et al .2018	30271481	Tumor
chr3	2044752	1819	CNTN6(dist=599474),CNTN4(dist=95798)	intergenic	HIVID	Yang et al .2018	30271481	Tumor
chr3	2044833	1029	CNTN6(dist=599555),CNTN4(dist=95717)	intergenic	HIVID	Yang et al .2018	30271481	Tumor
chr16	33835390	130	RNU6-76(dist=272147),LINC00273(dist=125662)	intergenic	HIVID	Yang et al .2018	30271481	Tumor
chr16	46477419	130	NONE(dist=NONE),ANKRD26P1(dist=25830)	intergenic	HIVID	Yang et al .2018	30271481	Tumor
chr19	28859205	1369	LINC00662(dist=574357),LOC148145(dist=596833)	intergenic	HIVID	Yang et al .2018	30271481	Tumor
chr19	48778613	1774	ZNF114	intronic	HIVID	Yang et al .2018	30271481	Tumor
chr19	50491435	1169	VRK3	intronic	HIVID	Yang et al .2018	30271481	Tumor
chr19	50491540	1179	VRK3	intronic	HIVID	Yang et al .2018	30271481	Tumor
chr19	50814110	1722	MYH14	downstream	HIVID	Yang et al .2018	30271481	Tumor
chr2	23374360	1144	LOC645949(dist=1440836),KLHL29(dist=233938)	intergenic	HIVID	Yang et al .2018	30271481	Tumor
chr21	9467738	130	NONE(dist=NONE),MIR3648(dist=358094)	intergenic	HIVID	Yang et al .2018	30271481	Tumor
chr4	54319376	1833	FIP1L1	intronic	HIVID	Yang et al .2018	30271481	Tumor
chr5	1295700	1728	TERT	upstream	HIVID	Yang et al .2018	30271481	Tumor
chr7	53205316	130	POM121L12(dist=100698),FLJ45974(dist=517886)	intergenic	HIVID	Yang et al .2018	30271481	Tumor
chr1	7475210	2893	CAMTA1	intronic	HIVID	Yang et al .2018	30271481	Tumor
chr1	30968919	2928	PTPRU(dist=1315594),MATN1(dist=215205)	intergenic	HIVID	Yang et al .2018	30271481	Tumor
chr1	50403190	2890	AGBL4	intronic	HIVID	Yang et al .2018	30271481	Tumor
chr1	73660103	2934	NEGR1(dist=911826),LRRIQ3(dist=831599)	intergenic	HIVID	Yang et al .2018	30271481	Tumor
chr1	80407534	2876	ELTD1(dist=935039),LPHN2(dist=1858548)	intergenic	HIVID	Yang et al .2018	30271481	Tumor
chr1	80407608	2876	ELTD1(dist=935113),LPHN2(dist=1858474)	intergenic	HIVID	Yang et al .2018	30271481	Tumor
chr1	84153724	2876	LPHN2(dist=1695617),MIR548AP(dist=105874)	intergenic	HIVID	Yang et al .2018	30271481	Tumor
chr1	91834877	2880	HFM1	intronic	HIVID	Yang et al .2018	30271481	Tumor
chr1	97784313	2876	DPYD-AS1	ncRNA_intronic	HIVID	Yang et al .2018	30271481	Tumor
chr1	110288790	2892	GSTM3(dist=5130),EPS8L3(dist=3912)	intergenic	HIVID	Yang et al .2018	30271481	Tumor
chr1	121468211	1802	EMBP1(dist=154525),NONE(dist=NONE)	intergenic	HIVID	Yang et al .2018	30271481	Tumor
chr1	121484859	1837	EMBP1(dist=171173),NONE(dist=NONE)	intergenic	HIVID	Yang et al .2018	30271481	Tumor
chr1	150692847	1818	HORMAD1	intronic	HIVID	Yang et al .2018	30271481	Tumor
chr10	20787950	2884	PLXDC2(dist=218835),MIR4675(dist=52949)	intergenic	HIVID	Yang et al .2018	30271481	Tumor
chr10	42418862	2185	NONE(dist=NONE),LOC441666(dist=408452)	intergenic	HIVID	Yang et al .2018	30271481	Tumor
chr10	44605572	2880	LOC283033(dist=140217),LOC100130539(dist=182626)	intergenic	HIVID	Yang et al .2018	30271481	Tumor
chr10	44766425	2881	LOC283033(dist=301070),LOC100130539(dist=21773)	intergenic	HIVID	Yang et al .2018	30271481	Tumor
chr10	54493557	2906	DKK1(dist=416140),MBL2(dist=31583)	intergenic	HIVID	Yang et al .2018	30271481	Tumor
chr10	78608645	2890	C10orf11(dist=291519),KCNMA1(dist=20714)	intergenic	HIVID	Yang et al .2018	30271481	Tumor
chr10	88512680	1837	LDB3(dist=16856),BMPR1A(dist=3716)	intergenic	HIVID	Yang et al .2018	30271481	Tumor
chr10	88512747	2323	LDB3(dist=16923),BMPR1A(dist=3649)	intergenic	HIVID	Yang et al .2018	30271481	Tumor
chr10	88541379	234	BMPR1A	intronic	HIVID	Yang et al .2018	30271481	Tumor
chr11	6928491	2911	OR2D2(dist=14760),OR2D3(dist=13742)	intergenic	HIVID	Yang et al .2018	30271481	Tumor
chr11	25300232	2893	LUZP2(dist=196046),ANO3(dist=1053446)	intergenic	HIVID	Yang et al .2018	30271481	Tumor
chr11	30193847	1574	KCNA4(dist=155270),FSHB(dist=58716)	intergenic	HIVID	Yang et al .2018	30271481	Tumor
chr11	45147282	2907	NONE(dist=NONE),NONE(dist=NONE)	intergenic	HIVID	Yang et al .2018	30271481	Tumor
chr11	71224746	2895	NADSYN1(dist=12165),KRTAP5-7(dist=13567)	intergenic	HIVID	Yang et al .2018	30271481	Tumor
chr11	85467695	1821	SYTL2	intronic	HIVID	Yang et al .2018	30271481	Tumor
chr11	90422251	2929	MIR4490(dist=133226),FAT3(dist=1663011)	intergenic	HIVID	Yang et al .2018	30271481	Tumor
chr11	91808378	2876	MIR4490(dist=1519353),FAT3(dist=276884)	intergenic	HIVID	Yang et al .2018	30271481	Tumor
chr11	99909177	2934	CNTN5	intronic	HIVID	Yang et al .2018	30271481	Tumor
chr11	101819952	2060	KIAA1377	intronic	HIVID	Yang et al .2018	30271481	Tumor
chr11	103010421	2927	DYNC2H1	intronic	HIVID	Yang et al .2018	30271481	Tumor
chr11	103689072	2876	DYNC2H1(dist=338481),MIR4693(dist=31562)	intergenic	HIVID	Yang et al .2018	30271481	Tumor
chr11	104889554	2881	CASP5	intronic	HIVID	Yang et al .2018	30271481	Tumor
chr12	17855652	2885	MIR3974(dist=29324),RERGL(dist=378151)	intergenic	HIVID	Yang et al .2018	30271481	Tumor
chr12	20691684	26	PDE3A	intronic	HIVID	Yang et al .2018	30271481	Tumor
chr12	27236578	2364	C12orf71(dist=1123),STK38L(dist=160500)	intergenic	HIVID	Yang et al .2018	30271481	Tumor
chr12	27236720	753	C12orf71(dist=1265),STK38L(dist=160358)	intergenic	HIVID	Yang et al .2018	30271481	Tumor
chr12	29031735	730	CCDC91(dist=328636),FAR2(dist=344863)	intergenic	HIVID	Yang et al .2018	30271481	Tumor
chr12	30588776	2895	TMTC1(dist=651084),IPO8(dist=193139)	intergenic	HIVID	Yang et al .2018	30271481	Tumor
chr12	39589233	2907	CPNE8(dist=289813),KIF21A(dist=97797)	intergenic	HIVID	Yang et al .2018	30271481	Tumor
chr12	40466367	2525	SLC2A13	intronic	HIVID	Yang et al .2018	30271481	Tumor
chr12	48943997	2886	OR8S1(dist=22111),LALBA(dist=17470)	intergenic	HIVID	Yang et al .2018	30271481	Tumor
chr12	50261607	1263	FAIM2	UTR3	HIVID	Yang et al .2018	30271481	Tumor
chr12	52354016	1461	ACVR1B	intronic	HIVID	Yang et al .2018	30271481	Tumor
chr12	55956753	2876	OR6C4(dist=10813),OR2AP1(dist=11446)	intergenic	HIVID	Yang et al .2018	30271481	Tumor
chr12	55986209	2876	OR2AP1(dist=17081),OR10P1(dist=44467)	intergenic	HIVID	Yang et al .2018	30271481	Tumor
chr12	60301093	819	SLC16A7(dist=117458),FAM19A2(dist=1800936)	intergenic	HIVID	Yang et al .2018	30271481	Tumor
chr12	66451373	539	HMGA2(dist=91302),LLPH(dist=65476)	intergenic	HIVID	Yang et al .2018	30271481	Tumor

Chromosome	Integration site in host genome	Integration site in virus genome	Gene (distance, bp)	Regions	Methods	Author	PMID	Sample
chr12	77656307	2876	E2F7(dist=196947),NAV3(dist=568762)	intergenic	HIVID	Yang et al .2018	30271481	Tumor
chr12	80641334	2962	OTOGL	intronic	HIVID	Yang et al .2018	30271481	Tumor
chr12	88431764	2923	C12orf29	intronic	HIVID	Yang et al .2018	30271481	Tumor
chr12	113319973	2189	RPH3A	intronic	HIVID	Yang et al .2018	30271481	Tumor
chr13	49171778	2374	LINC00462(dist=16741),CYSLTR2(dist=109173)	intergenic	HIVID	Yang et al .2018	30271481	Tumor
chr13	49171925	1823	LINC00462(dist=16888),CYSLTR2(dist=109026)	intergenic	HIVID	Yang et al .2018	30271481	Tumor
chr14	30246983	2876	PRKD1	intronic	HIVID	Yang et al .2018	30271481	Tumor
chr14	32989035	2876	AKAP6	intronic	HIVID	Yang et al .2018	30271481	Tumor
chr14	77213332	2931	ESRRB(dist=245152),VASH1(dist=14903)	intergenic	HIVID	Yang et al .2018	30271481	Tumor
chr14	88913359	2876	SPATA7(dist=8555),PTPN21(dist=18763)	intergenic	HIVID	Yang et al .2018	30271481	Tumor
chr15	20156084	2876	NONE(dist=NONE),CHEK2P2(dist=331913)	intergenic	HIVID	Yang et al .2018	30271481	Tumor
chr15	24400964	2876	NDN(dist=468514),PWRN2(dist=8962)	intergenic	HIVID	Yang et al .2018	30271481	Tumor
chr15	42836544	432	LRRC57	intronic	HIVID	Yang et al .2018	30271481	Tumor
chr15	51863615	2876	DMXL2	intronic	HIVID	Yang et al .2018	30271481	Tumor
chr15	63912724	2876	HERC1	intronic	HIVID	Yang et al .2018	30271481	Tumor
chr15	90454180	1829	C15orf38,C15orf38-AP3S2	intronic	HIVID	Yang et al .2018	30271481	Tumor
chr16	67674887	1418	CTCF(dist=1799),RLTPR(dist=4143)	intergenic	HIVID	Yang et al .2018	30271481	Tumor
chr16	67709419	1041	GFOD2	exonic	HIVID	Yang et al .2018	30271481	Tumor
chr16	68254799	1936	NFATC3	intronic	HIVID	Yang et al .2018	30271481	Tumor
chr16	75859247	3017	TERF2IP(dist=167906),CNTNAP4(dist=451929)	intergenic	HIVID	Yang et al .2018	30271481	Tumor
chr17	22256010	1802	MTRNR2L1(dist=232019),NONE(dist=NONE)	intergenic	HIVID	Yang et al .2018	30271481	Tumor
chr17	53635743	2888	MMD(dist=136402),TMEM100(dist=161245)	intergenic	HIVID	Yang et al .2018	30271481	Tumor
chr18	14165326	2923	ZNF519(dist=32837),ANKRD20A5P(dist=13770)	intergenic	HIVID	Yang et al .2018	30271481	Tumor
chr19	21788223	1823	ZNF429(dist=67144),ZNF100(dist=118620)	intergenic	HIVID	Yang et al .2018	30271481	Tumor
chr19	36212541	1548	KMT2B	exonic	HIVID	Yang et al .2018	30271481	Tumor
chr19	36212557	1808	KMT2B	exonic	HIVID	Yang et al .2018	30271481	Tumor
chr2	23209577	2926	LOC645949(dist=1276053),KLHL29(dist=398721)	intergenic	HIVID	Yang et al .2018	30271481	Tumor
chr2	37952913	2876	CDC42EP3(dist=53235),FAM82A1(dist=199549)	intergenic	HIVID	Yang et al .2018	30271481	Tumor
chr2	66154852	2876	SPRED2(dist=495196),MIR4778(dist=430529)	intergenic	HIVID	Yang et al .2018	30271481	Tumor
chr2	73665386	2122	ALMS1	intronic	HIVID	Yang et al .2018	30271481	Tumor
chr2	92320683	1900	ACTR3BP2(dist=190187),NONE(dist=NONE)	intergenic	HIVID	Yang et al .2018	30271481	Tumor
chr2	92324079	1900	ACTR3BP2(dist=193583),NONE(dist=NONE)	intergenic	HIVID	Yang et al .2018	30271481	Tumor
chr2	92325097	1900	ACTR3BP2(dist=194601),NONE(dist=NONE)	intergenic	HIVID	Yang et al .2018	30271481	Tumor
chr2	131043286	455	TUBA3E(dist=87252),CCDC115(dist=52530)	intergenic	HIVID	Yang et al .2018	30271481	Tumor
chr2	131043292	269	TUBA3E(dist=87258),CCDC115(dist=52524)	intergenic	HIVID	Yang et al .2018	30271481	Tumor
chr2	132124244	269	WTH3DI(dist=2513),LOC389043(dist=36230)	intergenic	HIVID	Yang et al .2018	30271481	Tumor
chr2	135507432	3122	TMEM163(dist=30861),ACMSD(dist=88754)	intergenic	HIVID	Yang et al .2018	30271481	Tumor
chr2	153185425	2507	STAM2(dist=152919),FMNL2(dist=6326)	intergenic	HIVID	Yang et al .2018	30271481	Tumor
chr2	159277733	2876	CCDC148	intronic	HIVID	Yang et al .2018	30271481	Tumor
chr20	22971576	2921	FOXA2(dist=405475),SSTR4(dist=44481)	intergenic	HIVID	Yang et al .2018	30271481	Tumor
chr21	10878715	2876	TEKT4P2(dist=910122),TPTE(dist=28028)	intergenic	HIVID	Yang et al .2018	30271481	Tumor
chr21	15365166	2925	ANKRD20A11P(dist=12401),LIPI(dist=115969)	intergenic	HIVID	Yang et al .2018	30271481	Tumor
chr21	19797471	2587	TMPRSS15(dist=21501),NONE(dist=NONE)	intergenic	HIVID	Yang et al .2018	30271481	Tumor
chr21	19800394	2478	TMPRSS15(dist=24424),NONE(dist=NONE)	intergenic	HIVID	Yang et al .2018	30271481	Tumor
chr3	3939571	2880	LRRN1(dist=50184),SETMAR(dist=405417)	intergenic	HIVID	Yang et al .2018	30271481	Tumor
chr3	26429605	2910	LOC285326(dist=514419),LRRC3B(dist=234695)	intergenic	HIVID	Yang et al .2018	30271481	Tumor
chr3	29171393	2893	LOC645206(dist=371565),RBMS3(dist=151410)	intergenic	HIVID	Yang et al .2018	30271481	Tumor
chr3	53402713	2892	DCP1A(dist=21059),CACNA1D(dist=126318)	intergenic	HIVID	Yang et al .2018	30271481	Tumor
chr3	65042026	2876	ADAMTS9-AS2(dist=44883),MAGI1(dist=297880)	intergenic	HIVID	Yang et al .2018	30271481	Tumor
chr3	81103925	2876	ROBO1(dist=1286866),GBE1(dist=434925)	intergenic	HIVID	Yang et al .2018	30271481	Tumor
chr3	110740060	2876	FLJ25363(dist=1526046),PVRL3-AS1(dist=24103)	intergenic	HIVID	Yang et al .2018	30271481	Tumor
chr3	122454399	2926	PARP14(dist=4712),HSPBAP1(dist=4445)	intergenic	HIVID	Yang et al .2018	30271481	Tumor
chr3	141188454	2436	ZBTB38(dist=19822),RASA2(dist=17472)	intergenic	HIVID	Yang et al .2018	30271481	Tumor
chr3	151229953	2899	IGSF10(dist=53456),MIR5186(dist=53711)	intergenic	HIVID	Yang et al .2018	30271481	Tumor
chr3	192952816	1040	MB21D2(dist=316866),HRASLS(dist=6101)	intergenic	HIVID	Yang et al .2018	30271481	Tumor
chr4	9671023	1707	MIR548I2(dist=113086),DRD5(dist=112235)	intergenic	HIVID	Yang et al .2018	30271481	Tumor
chr4	11582535	2876	HS3ST1(dist=151998),HSP90AB2P(dist=1752502)	intergenic	HIVID	Yang et al .2018	30271481	Tumor
chr4	57200767	2876	KIAA1211(dist=3877),AASDH(dist=3690)	intergenic	HIVID	Yang et al .2018	30271481	Tumor
chr4	59147991	2710	LOC255130(dist=1076526),NONE(dist=NONE)	intergenic	HIVID	Yang et al .2018	30271481	Tumor
chr4	59148425	2509	LOC255130(dist=1076960),NONE(dist=NONE)	intergenic	HIVID	Yang et al .2018	30271481	Tumor
chr4	59148464	1835	LOC255130(dist=1076999),NONE(dist=NONE)	intergenic	HIVID	Yang et al .2018	30271481	Tumor
chr4	59148535	1836	LOC255130(dist=1077070),NONE(dist=NONE)	intergenic	HIVID	Yang et al .2018	30271481	Tumor
chr4	59148556	1927	LOC255130(dist=1077091),NONE(dist=NONE)	intergenic	HIVID	Yang et al .2018	30271481	Tumor
chr4	59148758	2744	LOC255130(dist=1077293),NONE(dist=NONE)	intergenic	HIVID	Yang et al .2018	30271481	Tumor
chr4	71286979	2893	PROL1(dist=11065),MUC7(dist=9230)	intergenic	HIVID	Yang et al .2018	30271481	Tumor
chr4	94649535	2895	GRID2	intronic	HIVID	Yang et al .2018	30271481	Tumor
chr4	99243565	2923	RAP1GDS1	intronic	HIVID	Yang et al .2018	30271481	Tumor
chr4	101839948	2876	EMCN-IT3(dist=243678),PPP3CA(dist=104639)	intergenic	HIVID	Yang et al .2018	30271481	Tumor
chr4	111682026	2925	PITX2(dist=118747),C4orf32(dist=1384527)	intergenic	HIVID	Yang et al .2018	30271481	Tumor
chr4	123033782	2924	TRPC3(dist=160873),KIAA1109(dist=57976)	intergenic	HIVID	Yang et al .2018	30271481	Tumor
chr4	143914706	2929	INPP4B(dist=147102),USP38(dist=191364)	intergenic	HIVID	Yang et al .2018	30271481	Tumor
chr4	150737315	2876	NR3C2(dist=1373643),DCLK2(dist=262111)	intergenic	HIVID	Yang et al .2018	30271481	Tumor
chr4	166602912	970	CPE(dist=183430),TLL1(dist=191498)	intergenic	HIVID	Yang et al .2018	30271481	Tumor
chr5	939263	3044	TRIP13(dist=21099),LOC100506688(dist=49162)	intergenic	HIVID	Yang et al .2018	30271481	Tumor
chr5	1295677	1813	TERT	upstream	HIVID	Yang et al .2018	30271481	Tumor
chr5	1295775	1691	TERT	upstream	HIVID	Yang et al .2018	30271481	Tumor
chr5	1296148	986	TERT	upstream	HIVID	Yang et al .2018	30271481	Tumor
chr5	15398638	2887	ANKH(dist=526751),FBXL7(dist=101667)	intergenic	HIVID	Yang et al .2018	30271481	Tumor
chr5	77878640	2876	LHFPL2	intronic	HIVID	Yang et al .2018	30271481	Tumor
chr5	86979814	198	CCNH(dist=270964),TMEM161B(dist=511209)	intergenic	HIVID	Yang et al .2018	30271481	Tumor
chr5	86979814	246	CCNH(dist=270964),TMEM161B(dist=511209)	intergenic	HIVID	Yang et al .2018	30271481	Tumor
chr5	86979911	3210	CCNH(dist=271061),TMEM161B(dist=511112)	intergenic	HIVID	Yang et al .2018	30271481	Tumor
chr5	106110058	2876	RAB9BP1(dist=1674259),EFNA5(dist=602532)	intergenic	HIVID	Yang et al .2018	30271481	Tumor
chr5	129421695	2880	CHSY3	intronic	HIVID	Yang et al .2018	30271481	Tumor
chr5	131852154	2876	IRF1(dist=25689),IL5(dist=24982)	intergenic	HIVID	Yang et al .2018	30271481	Tumor
chr5	144206300	2907	KCTD16(dist=349356),PRELID2(dist=932282)	intergenic	HIVID	Yang et al .2018	30271481	Tumor
chr5	180477474	1386	BTNL9	intronic	HIVID	Yang et al .2018	30271481	Tumor
chr6	62231775	2876	NONE(dist=NONE),KHDRBS2(dist=158090)	intergenic	HIVID	Yang et al .2018	30271481	Tumor
chr6	65888587	2876	EYS	intronic	HIVID	Yang et al .2018	30271481	Tumor
chr6	78455320	2876	HTR1B(dist=282200),IRAK1BP1(dist=1121869)	intergenic	HIVID	Yang et al .2018	30271481	Tumor
chr6	122861858	2882	PKIB	intronic	HIVID	Yang et al .2018	30271481	Tumor
chr6	161778252	1818	PARK2	intronic	HIVID	Yang et al .2018	30271481	Tumor
chr6	163098968	1145	PARK2	intronic	HIVID	Yang et al .2018	30271481	Tumor
chr7	39649077	2599	LOC646999	upstream	HIVID	Yang et al .2018	30271481	Tumor

Chromosome	Integration site in host genome	Integration site in virus genome	Gene (distance, bp)	Regions	Methods	Author	PMID	Sample
chr7	42623558	2892	GLI3(dist=346940),C7orf25(dist=325314)	intergenic	HIVID	Yang et al .2018	30271481	Tumor
chr7	88909205	2876	ZNF804B	intronic	HIVID	Yang et al .2018	30271481	Tumor
chr8	43793788	2501	POTEA(dist=575460),NONE(dist=NONE)	intergenic	HIVID	Yang et al .2018	30271481	Tumor
chr8	43821477	2478	POTEA(dist=603149),NONE(dist=NONE)	intergenic	HIVID	Yang et al .2018	30271481	Tumor
chr8	43823084	2478	POTEA(dist=604756),NONE(dist=NONE)	intergenic	HIVID	Yang et al .2018	30271481	Tumor
chr8	43824956	2478	POTEA(dist=606628),NONE(dist=NONE)	intergenic	HIVID	Yang et al .2018	30271481	Tumor
chr8	43825993	2499	POTEA(dist=607665),NONE(dist=NONE)	intergenic	HIVID	Yang et al .2018	30271481	Tumor
chr8	43826824	2478	POTEA(dist=608496),NONE(dist=NONE)	intergenic	HIVID	Yang et al .2018	30271481	Tumor
chr8	43828692	2480	POTEA(dist=610364),NONE(dist=NONE)	intergenic	HIVID	Yang et al .2018	30271481	Tumor
chr8	43830559	2478	POTEA(dist=612231),NONE(dist=NONE)	intergenic	HIVID	Yang et al .2018	30271481	Tumor
chr8	43831596	2499	POTEA(dist=613268),NONE(dist=NONE)	intergenic	HIVID	Yang et al .2018	30271481	Tumor
chr8	43832426	2485	POTEA(dist=614098),NONE(dist=NONE)	intergenic	HIVID	Yang et al .2018	30271481	Tumor
chr8	43834295	2480	POTEA(dist=615967),NONE(dist=NONE)	intergenic	HIVID	Yang et al .2018	30271481	Tumor
chr8	43835362	2512	POTEA(dist=617034),NONE(dist=NONE)	intergenic	HIVID	Yang et al .2018	30271481	Tumor
chr8	43836163	2478	POTEA(dist=617835),NONE(dist=NONE)	intergenic	HIVID	Yang et al .2018	30271481	Tumor
chr8	43837392	2512	POTEA(dist=619064),NONE(dist=NONE)	intergenic	HIVID	Yang et al .2018	30271481	Tumor
chr8	43838032	2478	POTEA(dist=619704),NONE(dist=NONE)	intergenic	HIVID	Yang et al .2018	30271481	Tumor
chr8	46839034	2478	NONE(dist=NONE),LINC00293(dist=913474)	intergenic	HIVID	Yang et al .2018	30271481	Tumor
chr8	46840902	2478	NONE(dist=NONE),LINC00293(dist=911606)	intergenic	HIVID	Yang et al .2018	30271481	Tumor
chr8	46841961	2504	NONE(dist=NONE),LINC00293(dist=910547)	intergenic	HIVID	Yang et al .2018	30271481	Tumor
chr8	46842770	2478	NONE(dist=NONE),LINC00293(dist=909738)	intergenic	HIVID	Yang et al .2018	30271481	Tumor
chr8	46844130	687	NONE(dist=NONE),LINC00293(dist=908378)	intergenic	HIVID	Yang et al .2018	30271481	Tumor
chr8	46844638	2478	NONE(dist=NONE),LINC00293(dist=907870)	intergenic	HIVID	Yang et al .2018	30271481	Tumor
chr8	46846533	2484	NONE(dist=NONE),LINC00293(dist=905975)	intergenic	HIVID	Yang et al .2018	30271481	Tumor
chr8	46848375	2478	NONE(dist=NONE),LINC00293(dist=904133)	intergenic	HIVID	Yang et al .2018	30271481	Tumor
chr8	46849461	2478	NONE(dist=NONE),LINC00293(dist=903047)	intergenic	HIVID	Yang et al .2018	30271481	Tumor
chr8	46850244	2478	NONE(dist=NONE),LINC00293(dist=902264)	intergenic	HIVID	Yang et al .2018	30271481	Tumor
chr8	46852112	2478	NONE(dist=NONE),LINC00293(dist=900396)	intergenic	HIVID	Yang et al .2018	30271481	Tumor
chr8	46853179	2512	NONE(dist=NONE),LINC00293(dist=899329)	intergenic	HIVID	Yang et al .2018	30271481	Tumor
chr8	46853981	2478	NONE(dist=NONE),LINC00293(dist=898527)	intergenic	HIVID	Yang et al .2018	30271481	Tumor
chr8	46855850	2478	NONE(dist=NONE),LINC00293(dist=896658)	intergenic	HIVID	Yang et al .2018	30271481	Tumor
chr8	51787482	301	SNTG1(dist=82055),PXDNL(dist=444655)	intergenic	HIVID	Yang et al .2018	30271481	Tumor
chr8	69191193	2876	PREX2(dist=47296),LOC286189(dist=24510)	intergenic	HIVID	Yang et al .2018	30271481	Tumor
chr8	87762285	2918	CNGB3(dist=6382),CNBD1(dist=116391)	intergenic	HIVID	Yang et al .2018	30271481	Tumor
chr8	91278995	2924	LINC00534	ncRNA_intronic	HIVID	Yang et al .2018	30271481	Tumor
chr8	103093816	2473	NCALD	intronic	HIVID	Yang et al .2018	30271481	Tumor
chr9	3619160	2937	RFX3(dist=93177),GLIS3(dist=204968)	intergenic	HIVID	Yang et al .2018	30271481	Tumor
chr9	26446178	2876	LOC100506422(dist=327772),CAAP1(dist=394505)	intergenic	HIVID	Yang et al .2018	30271481	Tumor
chr9	34935161	601	FAM205B(dist=96578),KIAA1045(dist=23031)	intergenic	HIVID	Yang et al .2018	30271481	Tumor
chr9	39427950	2876	FAM75A2(dist=65991),LOC653501(dist=15864)	intergenic	HIVID	Yang et al .2018	30271481	Tumor
chr9	39957192	2876	FAM74A1(dist=49952),FAM75A3(dist=743099)	intergenic	HIVID	Yang et al .2018	30271481	Tumor
chr9	41258288	2876	ZNF658(dist=466176),FAM75A4(dist=62819)	intergenic	HIVID	Yang et al .2018	30271481	Tumor
chr9	41572968	2876	FAM75A5(dist=66043),LOC653501(dist=15865)	intergenic	HIVID	Yang et al .2018	30271481	Tumor
chr9	43561578	2876	LOC642929(dist=416094),FAM75A6(dist=62924)	intergenic	HIVID	Yang et al .2018	30271481	Tumor
chr9	68666321	2876	LOC642236(dist=211946),LOC100132352(dist=60220)	intergenic	HIVID	Yang et al .2018	30271481	Tumor
chr9	68991641	2876	LOC100132352(dist=243269),PGM5P2(dist=88603)	intergenic	HIVID	Yang et al .2018	30271481	Tumor
chr9	69005339	2876	LOC100132352(dist=256967),PGM5P2(dist=74905)	intergenic	HIVID	Yang et al .2018	30271481	Tumor
chr9	85625266	2156	RASEF	intronic	HIVID	Yang et al .2018	30271481	Tumor
chr9	103867805	2910	LPPR1	intronic	HIVID	Yang et al .2018	30271481	Tumor
chr9	104055744	2937	LPPR1	intronic	HIVID	Yang et al .2018	30271481	Tumor
chrX	25954502	2931	ARX(dist=920437),MAGEB18(dist=201958)	intergenic	HIVID	Yang et al .2018	30271481	Tumor
chrX	41866047	2876	CASK(dist=83760),PPP1R2P9(dist=770570)	intergenic	HIVID	Yang et al .2018	30271481	Tumor
chrX	54906884	2892	MAGED2(dist=64439),TRO(dist=40365)	intergenic	HIVID	Yang et al .2018	30271481	Tumor
chrX	74469615	2880	ABCB7(dist=93483),UPRT(dist=24279)	intergenic	HIVID	Yang et al .2018	30271481	Tumor
chrX	80945606	2910	SH3BGRL(dist=391560),POU3F4(dist=1817663)	intergenic	HIVID	Yang et al .2018	30271481	Tumor
chrX	102129507	2876	LINC00630	ncRNA_intronic	HIVID	Yang et al .2018	30271481	Tumor
chrX	102278784	3008	RAB40AL(dist=85556),BEX1(dist=38797)	intergenic	HIVID	Yang et al .2018	30271481	Tumor
chrX	102540091	2919	TCEAL5(dist=8294),BEX2(dist=24183)	intergenic	HIVID	Yang et al .2018	30271481	Tumor
chrX	119320365	2876	RHOXF2B(dist=22420),NKAPP1(dist=49944)	intergenic	HIVID	Yang et al .2018	30271481	Tumor
chrX	134778636	1739	DDX26B(dist=62176),CT45A1(dist=68549)	intergenic	HIVID	Yang et al .2018	30271481	Tumor
chrX	134778649	1821	DDX26B(dist=62189),CT45A1(dist=68536)	intergenic	HIVID	Yang et al .2018	30271481	Tumor
chrX	146226930	2878	CXorf51A(dist=330681),MIR506(dist=85308)	intergenic	HIVID	Yang et al .2018	30271481	Tumor
chr1	45976941	2273	PRDX1	UTR3	HIVID	Yang et al .2018	30271481	Tumor
chr10	42528106	168	NONE(dist=NONE),LOC441666(dist=299208)	intergenic	HIVID	Yang et al .2018	30271481	Tumor
chr10	88512743	1837	LDB3(dist=16919),BMPR1A(dist=3653)	intergenic	HIVID	Yang et al .2018	30271481	Tumor
chr10	88512748	2325	LDB3(dist=16924),BMPR1A(dist=3648)	intergenic	HIVID	Yang et al .2018	30271481	Tumor
chr10	88541379	234	BMPR1A	intronic	HIVID	Yang et al .2018	30271481	Tumor
chr11	101819952	2060	KIAA1377	intronic	HIVID	Yang et al .2018	30271481	Tumor
chr12	20691684	26	PDE3A	intronic	HIVID	Yang et al .2018	30271481	Tumor
chr12	27236720	721	C12orf71(dist=1265),STK38L(dist=160358)	intergenic	HIVID	Yang et al .2018	30271481	Tumor
chr12	56638886	497	ANKRD52(NM_173595:exon23:c.2492+1T>C)	splicing	HIVID	Yang et al .2018	30271481	Tumor
chr12	113319973	2189	RPH3A	intronic	HIVID	Yang et al .2018	30271481	Tumor
chr17	14984304	2354	CDRT7(dist=49030),PMP22(dist=148792)	intergenic	HIVID	Yang et al .2018	30271481	Tumor
chr17	29563734	2686	NF1	intronic	HIVID	Yang et al .2018	30271481	Tumor
chr19	36212557	1808	KMT2B	exonic	HIVID	Yang et al .2018	30271481	Tumor
chr2	171274896	1799	MYO3B	intronic	HIVID	Yang et al .2018	30271481	Tumor
chr3	38226448	830	OXSR1	intronic	HIVID	Yang et al .2018	30271481	Tumor
chr4	9671023	1707	MIR548I2(dist=113086),DRD5(dist=112235)	intergenic	HIVID	Yang et al .2018	30271481	Tumor
chr4	27205017	2439	STIM2(dist=178014),MIR4275(dist=1616187)	intergenic	HIVID	Yang et al .2018	30271481	Tumor
chr4	59148534	1835	LOC255130(dist=1077069),NONE(dist=NONE)	intergenic	HIVID	Yang et al .2018	30271481	Tumor
chr4	59148781	2744	LOC255130(dist=1077316),NONE(dist=NONE)	intergenic	HIVID	Yang et al .2018	30271481	Tumor
chr4	64979913	1601	NONE(dist=NONE),TECRL(dist=164264)	intergenic	HIVID	Yang et al .2018	30271481	Tumor
chr4	166602912	970	CPE(dist=183430),TLL1(dist=191498)	intergenic	HIVID	Yang et al .2018	30271481	Tumor
chr5	1301353	1916	TERT(dist=6191),MIR4457(dist=8072)	intergenic	HIVID	Yang et al .2018	30271481	Tumor
chr5	1333266	1810	CLPTM1L	intronic	HIVID	Yang et al .2018	30271481	Tumor
chr5	1333844	1805	CLPTM1L	intronic	HIVID	Yang et al .2018	30271481	Tumor
chr6	66414213	21	EYS	intronic	HIVID	Yang et al .2018	30271481	Tumor
chr6	66414401	2899	EYS	intronic	HIVID	Yang et al .2018	30271481	Tumor
chr6	67055234	1263	SLC25A51P1(dist=555858),NONE(dist=NONE)	intergenic	HIVID	Yang et al .2018	30271481	Tumor
chr6	154477153	1433	IPCEF1	UTR3	HIVID	Yang et al .2018	30271481	Tumor
chr6	163098968	1145	PARK2	intronic	HIVID	Yang et al .2018	30271481	Tumor
chr7	34985094	1421	DPY19L1	intronic	HIVID	Yang et al .2018	30271481	Tumor
chr8	43826824	2478	POTEA(dist=608496),NONE(dist=NONE)	intergenic	HIVID	Yang et al .2018	30271481	Tumor
chr8	43830559	2478	POTEA(dist=612231),NONE(dist=NONE)	intergenic	HIVID	Yang et al .2018	30271481	Tumor

Chromosome	Integration site in host genome	Integration site in virus genome	Gene (distance, bp)	Regions	Methods	Author	PMID	Sample
chr8	43832427	2478	POTEA(dist=614099),NONE(dist=NONE)	intergenic	HIVID	Yang et al .2018	30271481	Tumor
chr8	43834296	2478	POTEA(dist=615968),NONE(dist=NONE)	intergenic	HIVID	Yang et al .2018	30271481	Tumor
chr8	43838032	2478	POTEA(dist=619704),NONE(dist=NONE)	intergenic	HIVID	Yang et al .2018	30271481	Tumor
chr8	51787482	301	SNTG1(dist=82055),PXDNL(dist=444655)	intergenic	HIVID	Yang et al .2018	30271481	Tumor
chr8	143846505	1344	LYNX1	exonic	HIVID	Yang et al .2018	30271481	Tumor
chr9	34935153	702	FAM205B(dist=96570),KIAA1045(dist=23039)	intergenic	HIVID	Yang et al .2018	30271481	Tumor
chr1	10370	1459	NONE(dist=NONE),DDX11L1(dist=1504)	intergenic	HIVID	Yang et al .2018	30271481	Tumor
chr1	249240249	1555	PGBD2(dist=26904),NONE(dist=NONE)	intergenic	HIVID	Yang et al .2018	30271481	Tumor
chr10	42599696	1825	NONE(dist=NONE),LOC441666(dist=227618)	intergenic	HIVID	Yang et al .2018	30271481	Tumor
chr19	3353560	2613	CELF5(dist=56487),NFIC(dist=6001)	intergenic	HIVID	Yang et al .2018	30271481	Tumor
chr19	36207835	2750	ZBTB32	UTR3	HIVID	Yang et al .2018	30271481	Tumor
chr19	36213028	1809	KMT2B	intronic	HIVID	Yang et al .2018	30271481	Tumor
chr2	89848733	1825	MIR4436A(dist=736765),LOC654342(dist=1975976)	intergenic	HIVID	Yang et al .2018	30271481	Tumor
chr3	174950759	1777	NAALADL2	intronic	HIVID	Yang et al .2018	30271481	Tumor
chr4	191044248	1574	DUX4L2(dist=30772),NONE(dist=NONE)	intergenic	HIVID	Yang et al .2018	30271481	Tumor
chr5	56417686	1809	MIER3(dist=169732),GPBP1(dist=52089)	intergenic	HIVID	Yang et al .2018	30271481	Tumor
chr5	137853788	1809	ETF1	intronic	HIVID	Yang et al .2018	30271481	Tumor
chr8	155612	1574	OR4F21(dist=38588),RPL23AP53(dist=2733)	intergenic	HIVID	Yang et al .2018	30271481	Tumor
chr8	51957231	2765	SNTG1(dist=251804),PXDNL(dist=274906)	intergenic	HIVID	Yang et al .2018	30271481	Tumor
chr8	106836106	1827	ZFPM2(dist=19339),OXR1(dist=446300)	intergenic	HIVID	Yang et al .2018	30271481	Tumor
chrY	13472851	1820	NONE(dist=NONE),GYG2P1(dist=1045064)	intergenic	HIVID	Yang et al .2018	30271481	Tumor
chrY	13479603	2322	NONE(dist=NONE),GYG2P1(dist=1038312)	intergenic	HIVID	Yang et al .2018	30271481	Tumor
chr1	121484315	168	EMBP1(dist=170629),NONE(dist=NONE)	intergenic	HIVID	Yang et al .2018	30271481	Tumor
chr1	185664789	285	LOC100288079(dist=360618),HMCN1(dist=38894)	intergenic	HIVID	Yang et al .2018	30271481	Tumor
chr1	185674893	1832	LOC100288079(dist=370722),HMCN1(dist=28790)	intergenic	HIVID	Yang et al .2018	30271481	Tumor
chr1	241741599	521	KMO	intronic	HIVID	Yang et al .2018	30271481	Tumor
chr10	313210	2630	ZMYND11(dist=12633),DIP2C(dist=6920)	intergenic	HIVID	Yang et al .2018	30271481	Tumor
chr10	23166624	970	PIP4K2A(dist=163121),ARMC3(dist=50330)	intergenic	HIVID	Yang et al .2018	30271481	Tumor
chr10	42400673	168	NONE(dist=NONE),LOC441666(dist=426641)	intergenic	HIVID	Yang et al .2018	30271481	Tumor
chr10	42528047	168	NONE(dist=NONE),LOC441666(dist=299267)	intergenic	HIVID	Yang et al .2018	30271481	Tumor
chr10	42529776	168	NONE(dist=NONE),LOC441666(dist=297538)	intergenic	HIVID	Yang et al .2018	30271481	Tumor
chr10	42532491	168	NONE(dist=NONE),LOC441666(dist=294823)	intergenic	HIVID	Yang et al .2018	30271481	Tumor
chr10	42534936	274	NONE(dist=NONE),LOC441666(dist=292378)	intergenic	HIVID	Yang et al .2018	30271481	Tumor
chr10	42535437	180	NONE(dist=NONE),LOC441666(dist=291877)	intergenic	HIVID	Yang et al .2018	30271481	Tumor
chr10	42538799	170	NONE(dist=NONE),LOC441666(dist=288515)	intergenic	HIVID	Yang et al .2018	30271481	Tumor
chr10	42541857	170	NONE(dist=NONE),LOC441666(dist=285457)	intergenic	HIVID	Yang et al .2018	30271481	Tumor
chr10	42542971	200	NONE(dist=NONE),LOC441666(dist=284343)	intergenic	HIVID	Yang et al .2018	30271481	Tumor
chr10	42544747	168	NONE(dist=NONE),LOC441666(dist=282567)	intergenic	HIVID	Yang et al .2018	30271481	Tumor
chr10	88512743	1837	LDB3(dist=16919),BMPR1A(dist=3653)	intergenic	HIVID	Yang et al .2018	30271481	Tumor
chr10	88541379	234	BMPR1A	intronic	HIVID	Yang et al .2018	30271481	Tumor
chr12	29031735	730	CCDC91(dist=328636),FAR2(dist=344863)	intergenic	HIVID	Yang et al .2018	30271481	Tumor
chr12	50261607	1204	FAIM2	UTR3	HIVID	Yang et al .2018	30271481	Tumor
chr12	50261731	587	FAIM2	UTR3	HIVID	Yang et al .2018	30271481	Tumor
chr12	66451373	489	HMGA2(dist=91302),LLPH(dist=65476)	intergenic	HIVID	Yang et al .2018	30271481	Tumor
chr12	88431764	2902	C12orf29	intronic	HIVID	Yang et al .2018	30271481	Tumor
chr12	88431859	2058	C12orf29	intronic	HIVID	Yang et al .2018	30271481	Tumor
chr12	113319972	2189	RPH3A	intronic	HIVID	Yang et al .2018	30271481	Tumor
chr14	78981628	1828	NRXN3	intronic	HIVID	Yang et al .2018	30271481	Tumor
chr15	64430428	958	SNX1	UTR3	HIVID	Yang et al .2018	30271481	Tumor
chr16	46392261	3201	NONE(dist=NONE),ANKRD26P1(dist=110988)	intergenic	HIVID	Yang et al .2018	30271481	Tumor
chr16	46404181	3201	NONE(dist=NONE),ANKRD26P1(dist=99068)	intergenic	HIVID	Yang et al .2018	30271481	Tumor
chr16	67674887	1461	CTCF(dist=1799),RLTPR(dist=4143)	intergenic	HIVID	Yang et al .2018	30271481	Tumor
chr16	67709429	1041	GFOD2	exonic	HIVID	Yang et al .2018	30271481	Tumor
chr16	75368094	1814	CFDP1	intronic	HIVID	Yang et al .2018	30271481	Tumor
chr17	14984304	2354	CDRT7(dist=49030),PMP22(dist=148792)	intergenic	HIVID	Yang et al .2018	30271481	Tumor
chr17	22259403	637	MTRNR2L1(dist=235412),NONE(dist=NONE)	intergenic	HIVID	Yang et al .2018	30271481	Tumor
chr18	71132732	984	LOC100505817(dist=115608),FBXO15(dist=607856)	intergenic	HIVID	Yang et al .2018	30271481	Tumor
chr19	27736723	168	NONE(dist=NONE),LINC00662(dist=544678)	intergenic	HIVID	Yang et al .2018	30271481	Tumor
chr19	27737740	168	NONE(dist=NONE),LINC00662(dist=543661)	intergenic	HIVID	Yang et al .2018	30271481	Tumor
chr19	36212467	1548	KMT2B	exonic	HIVID	Yang et al .2018	30271481	Tumor
chr19	36212538	1547	KMT2B	exonic	HIVID	Yang et al .2018	30271481	Tumor
chr19	36212556	1806	KMT2B	exonic	HIVID	Yang et al .2018	30271481	Tumor
chr19	36212557	1731	KMT2B	exonic	HIVID	Yang et al .2018	30271481	Tumor
chr19	36212557	1805	KMT2B	exonic	HIVID	Yang et al .2018	30271481	Tumor
chr19	36213045	1826	KMT2B	intronic	HIVID	Yang et al .2018	30271481	Tumor
chr2	99818030	1010	MRPL30(dist=2010),LYG2(dist=40681)	intergenic	HIVID	Yang et al .2018	30271481	Tumor
chr2	171274896	1799	MYO3B	intronic	HIVID	Yang et al .2018	30271481	Tumor
chr2	171274934	1826	MYO3B	intronic	HIVID	Yang et al .2018	30271481	Tumor
chr3	157181412	1818	VEPH1	intronic	HIVID	Yang et al .2018	30271481	Tumor
chr4	3977192	1656	FAM86EP(dist=20044),OTOP1(dist=213338)	intergenic	HIVID	Yang et al .2018	30271481	Tumor
chr4	9671022	1705	MIR548I2(dist=113085),DRD5(dist=112236)	intergenic	HIVID	Yang et al .2018	30271481	Tumor
chr4	9671067	1133	MIR548I2(dist=113130),DRD5(dist=112191)	intergenic	HIVID	Yang et al .2018	30271481	Tumor
chr4	53244638	301	SPATA18(dist=281180),USP46(dist=212489)	intergenic	HIVID	Yang et al .2018	30271481	Tumor
chr4	56985044	1806	CEP135(dist=85515),KIAA1211(dist=51317)	intergenic	HIVID	Yang et al .2018	30271481	Tumor
chr4	68264403	168	LOC100144602(dist=1705299),CENPC1(dist=73586)	intergenic	HIVID	Yang et al .2018	30271481	Tumor
chr4	68265531	257	LOC100144602(dist=1706427),CENPC1(dist=72458)	intergenic	HIVID	Yang et al .2018	30271481	Tumor
chr4	68266049	175	LOC100144602(dist=1706945),CENPC1(dist=71940)	intergenic	HIVID	Yang et al .2018	30271481	Tumor
chr4	166602840	970	CPE(dist=183358),TLL1(dist=191570)	intergenic	HIVID	Yang et al .2018	30271481	Tumor
chr4	166602911	970	CPE(dist=183429),TLL1(dist=191499)	intergenic	HIVID	Yang et al .2018	30271481	Tumor
chr4	168765603	1486	SPOCK3(dist=609862),ANXA10(dist=248085)	intergenic	HIVID	Yang et al .2018	30271481	Tumor
chr4	168766105	1394	SPOCK3(dist=610364),ANXA10(dist=247583)	intergenic	HIVID	Yang et al .2018	30271481	Tumor
chr5	11282981	1495	CTNND2	intronic	HIVID	Yang et al .2018	30271481	Tumor
chr5	11282985	2416	CTNND2	intronic	HIVID	Yang et al .2018	30271481	Tumor
chr7	61968930	168	NONE(dist=NONE),LOC643955(dist=782740)	intergenic	HIVID	Yang et al .2018	30271481	Tumor
chr7	61979285	168	NONE(dist=NONE),LOC643955(dist=772385)	intergenic	HIVID	Yang et al .2018	30271481	Tumor
chr7	61982079	169	NONE(dist=NONE),LOC643955(dist=769591)	intergenic	HIVID	Yang et al .2018	30271481	Tumor
chr8	51787481	297	SNTG1(dist=82054),PXDNL(dist=444656)	intergenic	HIVID	Yang et al .2018	30271481	Tumor
chr8	51788172	301	SNTG1(dist=82745),PXDNL(dist=443965)	intergenic	HIVID	Yang et al .2018	30271481	Tumor
chr8	97587863	1757	SDC2	intronic	HIVID	Yang et al .2018	30271481	Tumor
chr8	129524908	1621	MIR1208(dist=362474),LOC728724(dist=703805)	intergenic	HIVID	Yang et al .2018	30271481	Tumor
chr9	7880786	301	C9orf123(dist=80987),PTPRD(dist=433460)	intergenic	HIVID	Yang et al .2018	30271481	Tumor
chrX	134778636	1739	DDX26B(dist=62176),CT45A1(dist=68549)	intergenic	HIVID	Yang et al .2018	30271481	Tumor
chrY	58836350	2402	NONE(dist=NONE),SPRY3(dist=264107)	intergenic	HIVID	Yang et al .2018	30271481	Tumor
chrY	59363191	1809	IL9R(dist=19703),NONE(dist=NONE)	intergenic	HIVID	Yang et al .2018	30271481	Tumor

Chromosome	Integration site in host genome	Integration site in virus genome	Gene (distance, bp)	Regions	Methods	Author	PMID	Sample
chr1	121353223	637	EMBP1(dist=39537),NONE(dist=NONE)	intergenic	HIVID	Yang et al .2018	30271481	Tumor
chr17	21082020	389	DHRS7B	intronic	HIVID	Yang et al .2018	30271481	Tumor
chr17	22249941	626	MTRNR2L1(dist=225950),NONE(dist=NONE)	intergenic	HIVID	Yang et al .2018	30271481	Tumor
chr17	22252267	687	MTRNR2L1(dist=228276),NONE(dist=NONE)	intergenic	HIVID	Yang et al .2018	30271481	Tumor
chr17	22254646	687	MTRNR2L1(dist=230655),NONE(dist=NONE)	intergenic	HIVID	Yang et al .2018	30271481	Tumor
chr17	22257025	687	MTRNR2L1(dist=233034),NONE(dist=NONE)	intergenic	HIVID	Yang et al .2018	30271481	Tumor
chr17	22259403	637	MTRNR2L1(dist=235412),NONE(dist=NONE)	intergenic	HIVID	Yang et al .2018	30271481	Tumor
chr20	7363596	586	BMP2(dist=602686),HAO1(dist=500035)	intergenic	HIVID	Yang et al .2018	30271481	Tumor
chr20	7363610	1656	BMP2(dist=602700),HAO1(dist=500021)	intergenic	HIVID	Yang et al .2018	30271481	Tumor
chr22	38274778	3172	EIF3L	intronic	HIVID	Yang et al .2018	30271481	Tumor
chr3	157181403	1828	VEPH1	intronic	HIVID	Yang et al .2018	30271481	Tumor
chr3	157181412	1818	VEPH1	intronic	HIVID	Yang et al .2018	30271481	Tumor
chr4	57036284	841	KIAA1211	upstream	HIVID	Yang et al .2018	30271481	Tumor
chr4	57869988	133	POLR2B	intronic	HIVID	Yang et al .2018	30271481	Tumor
chr4	63924796	1878	LPHN3(dist=986628),TECRL(dist=1219381)	intergenic	HIVID	Yang et al .2018	30271481	Tumor
chr5	1297281	133	TERT(dist=2119),MIR4457(dist=12144)	intergenic	HIVID	Yang et al .2018	30271481	Tumor
chr5	1303990	1775	TERT(dist=8828),MIR4457(dist=5435)	intergenic	HIVID	Yang et al .2018	30271481	Tumor
chr8	57690495	1584	LOC100507632(dist=218113),IMPAD1(dist=179993)	intergenic	HIVID	Yang et al .2018	30271481	Tumor
chr1	80407534	2876	ELTD1(dist=935039),LPHN2(dist=1858548)	intergenic	HIVID	Yang et al .2018	30271481	Tumor
chr1	80407608	2876	ELTD1(dist=935113),LPHN2(dist=1858474)	intergenic	HIVID	Yang et al .2018	30271481	Tumor
chr1	158269671	2132	CD1C(dist=5107),CD1B(dist=28069)	intergenic	HIVID	Yang et al .2018	30271481	Tumor
chr10	88512738	1837	LDB3(dist=16914),BMPR1A(dist=3658)	intergenic	HIVID	Yang et al .2018	30271481	Tumor
chr10	88512748	2325	LDB3(dist=16924),BMPR1A(dist=3648)	intergenic	HIVID	Yang et al .2018	30271481	Tumor
chr10	135524667	2588	DUX2(dist=26175),NONE(dist=NONE)	intergenic	HIVID	Yang et al .2018	30271481	Tumor
chr11	85467604	1707	SYTL2	intronic	HIVID	Yang et al .2018	30271481	Tumor
chr12	893268	1957	WNK1	intronic	HIVID	Yang et al .2018	30271481	Tumor
chr12	2250290	437	CACNA1C	intronic	HIVID	Yang et al .2018	30271481	Tumor
chr12	2250418	1363	CACNA1C	intronic	HIVID	Yang et al .2018	30271481	Tumor
chr12	3098348	2634	TEAD4	intronic	HIVID	Yang et al .2018	30271481	Tumor
chr12	7291596	2801	CLSTN3	intronic	HIVID	Yang et al .2018	30271481	Tumor
chr12	7576194	2132	CD163L1	intronic	HIVID	Yang et al .2018	30271481	Tumor
chr12	20691684	26	PDE3A	intronic	HIVID	Yang et al .2018	30271481	Tumor
chr12	20691809	2713	PDE3A	intronic	HIVID	Yang et al .2018	30271481	Tumor
chr12	27236556	2364	C12orf71(dist=1101),STK38L(dist=160522)	intergenic	HIVID	Yang et al .2018	30271481	Tumor
chr12	27236720	753	C12orf71(dist=1265),STK38L(dist=160358)	intergenic	HIVID	Yang et al .2018	30271481	Tumor
chr12	27240915	964	C12orf71(dist=5460),STK38L(dist=156163)	intergenic	HIVID	Yang et al .2018	30271481	Tumor
chr12	28021444	2969	KLHDC5(dist=65471),PTHLH(dist=89573)	intergenic	HIVID	Yang et al .2018	30271481	Tumor
chr12	29031735	730	CCDC91(dist=328636),FAR2(dist=344863)	intergenic	HIVID	Yang et al .2018	30271481	Tumor
chr12	29035977	1686	CCDC91(dist=332878),FAR2(dist=340621)	intergenic	HIVID	Yang et al .2018	30271481	Tumor
chr12	55956753	2876	OR6C4(dist=10813),OR2AP1(dist=11446)	intergenic	HIVID	Yang et al .2018	30271481	Tumor
chr12	77656307	2876	E2F7(dist=196947),NAV3(dist=568762)	intergenic	HIVID	Yang et al .2018	30271481	Tumor
chr14	30246983	2876	PRKD1	intronic	HIVID	Yang et al .2018	30271481	Tumor
chr14	32989035	2876	AKAP6	intronic	HIVID	Yang et al .2018	30271481	Tumor
chr14	78981628	1828	NRXN3	intronic	HIVID	Yang et al .2018	30271481	Tumor
chr15	24400964	2876	NDN(dist=468514),PWRN2(dist=8962)	intergenic	HIVID	Yang et al .2018	30271481	Tumor
chr16	67674887	1457	CTCF(dist=1799),RLTPR(dist=4143)	intergenic	HIVID	Yang et al .2018	30271481	Tumor
chr16	67709426	1041	GFOD2	exonic	HIVID	Yang et al .2018	30271481	Tumor
chr17	81195076	2588	FLJ43681(dist=6503),NONE(dist=NONE)	intergenic	HIVID	Yang et al .2018	30271481	Tumor
chr18	78016284	2588	PARD6G(dist=10887),NONE(dist=NONE)	intergenic	HIVID	Yang et al .2018	30271481	Tumor
chr19	36212557	1808	KMT2B	exonic	HIVID	Yang et al .2018	30271481	Tumor
chr2	66154849	2876	SPRED2(dist=495193),MIR4778(dist=430532)	intergenic	HIVID	Yang et al .2018	30271481	Tumor
chr21	10878758	2876	TEKT4P2(dist=910165),TPTE(dist=27985)	intergenic	HIVID	Yang et al .2018	30271481	Tumor
chr4	86160931	344	WDFY3-AS2(dist=232763),ARHGAP24(dist=235353)	intergenic	HIVID	Yang et al .2018	30271481	Tumor
chr4	101839948	2876	EMCN-IT3(dist=243678),PPP3CA(dist=104639)	intergenic	HIVID	Yang et al .2018	30271481	Tumor
chr5	1295696	1813	TERT	upstream	HIVID	Yang et al .2018	30271481	Tumor
chr5	1295777	1691	TERT	upstream	HIVID	Yang et al .2018	30271481	Tumor
chr5	86979814	254	CCNH(dist=270964),TMEM161B(dist=511209)	intergenic	HIVID	Yang et al .2018	30271481	Tumor
chr5	131852154	2932	IRF1(dist=25689),IL5(dist=24982)	intergenic	HIVID	Yang et al .2018	30271481	Tumor
chr5	152135092	344	NMUR2(dist=350252),GRIA1(dist=734992)	intergenic	HIVID	Yang et al .2018	30271481	Tumor
chr6	62231775	2876	NONE(dist=NONE),KHDRBS2(dist=158090)	intergenic	HIVID	Yang et al .2018	30271481	Tumor
chr6	78455320	2876	HTR1B(dist=282200),IRAK1BP1(dist=1121869)	intergenic	HIVID	Yang et al .2018	30271481	Tumor
chr7	88909218	2876	ZNF804B	intronic	HIVID	Yang et al .2018	30271481	Tumor
chr8	51787482	301	SNTG1(dist=82055),PXDNL(dist=444655)	intergenic	HIVID	Yang et al .2018	30271481	Tumor
chr8	55893017	344	RP1(dist=349623),XKR4(dist=122000)	intergenic	HIVID	Yang et al .2018	30271481	Tumor
chr8	85317713	2132	RALYL	intronic	HIVID	Yang et al .2018	30271481	Tumor
chr9	41258292	2876	ZNF658(dist=466180),FAM75A4(dist=62815)	intergenic	HIVID	Yang et al .2018	30271481	Tumor
chr9	68666323	2876	LOC642236(dist=211948),LOC100132352(dist=60218)	intergenic	HIVID	Yang et al .2018	30271481	Tumor
chr9	69005344	2876	LOC100132352(dist=256972),PGM5P2(dist=74900)	intergenic	HIVID	Yang et al .2018	30271481	Tumor
chr12	61479459	2153	SLC16A7(dist=1295824),FAM19A2(dist=622570)	intergenic	HIVID	Yang et al .2018	30271481	Tumor
chr12	89298516	2380	KITLG(dist=324266),LOC728084(dist=106387)	intergenic	HIVID	Yang et al .2018	30271481	Tumor
chr15	39150040	1783	C15orf53(dist=157801),C15orf54(dist=392845)	intergenic	HIVID	Yang et al .2018	30271481	Tumor
chr15	49608516	1955	GALK2	intronic	HIVID	Yang et al .2018	30271481	Tumor
chr18	71949448	1792	CYB5A	intronic	HIVID	Yang et al .2018	30271481	Tumor
chr2	216274031	1800	FN1	intronic	HIVID	Yang et al .2018	30271481	Tumor
chr2	216274031	1850	FN1	intronic	HIVID	Yang et al .2018	30271481	Tumor
chr4	49104004	1765	CWH43(dist=39909),NONE(dist=NONE)	intergenic	HIVID	Yang et al .2018	30271481	Tumor
chr7	109144745	2513	C7orf66(dist=620108),EIF3IP1(dist=454539)	intergenic	HIVID	Yang et al .2018	30271481	Tumor
chr8	137386451	2943	KHDRBS3(dist=726603),FAM135B(dist=1755815)	intergenic	HIVID	Yang et al .2018	30271481	Tumor
chr8	137394321	1746	KHDRBS3(dist=734473),FAM135B(dist=1747945)	intergenic	HIVID	Yang et al .2018	30271481	Tumor
chr1	69911524	1916	DEPDC1(dist=948725),LRRC7(dist=314334)	intergenic	HIVID	Yang et al .2018	30271481	Tumor
chr10	20859307	1859	MIR4675(dist=18332),NEBL(dist=209596)	intergenic	HIVID	Yang et al .2018	30271481	Tumor
chr10	24792721	1624	KIAA1217	intronic	HIVID	Yang et al .2018	30271481	Tumor
chr10	26354198	1873	MYO3A	intronic	HIVID	Yang et al .2018	30271481	Tumor
chr10	43024175	1859	ZNF37BP	ncRNA_intronic	HIVID	Yang et al .2018	30271481	Tumor
chr10	93293035	1859	LOC100188947	ncRNA_intronic	HIVID	Yang et al .2018	30271481	Tumor
chr10	95284114	1867	CEP55	intronic	HIVID	Yang et al .2018	30271481	Tumor
chr11	13844124	1871	FAR1(dist=90231),SPON1(dist=140060)	intergenic	HIVID	Yang et al .2018	30271481	Tumor
chr11	30193847	1574	KCNA4(dist=155270),FSHB(dist=58716)	intergenic	HIVID	Yang et al .2018	30271481	Tumor
chr11	56494743	1859	OR8U8	intronic	HIVID	Yang et al .2018	30271481	Tumor
chr11	67634759	1873	FAM86C2P(dist=61952),UNC93B1(dist=123816)	intergenic	HIVID	Yang et al .2018	30271481	Tumor
chr11	71438210	1904	KRTAP5-11(dist=144289),FAM86C1(dist=60347)	intergenic	HIVID	Yang et al .2018	30271481	Tumor
chr11	79504111	1859	ODZ4(dist=352416),NONE(dist=NONE)	intergenic	HIVID	Yang et al .2018	30271481	Tumor
chr11	82056174	1876	MIR4300(dist=454296),FAM181B(dist=386872)	intergenic	HIVID	Yang et al .2018	30271481	Tumor
chr11	99868384	1859	CNTN5	intronic	HIVID	Yang et al .2018	30271481	Tumor

Supplementary Table S8 Continued

Chromosome	Integration site in host genome	Integration site in virus genome	Gene (distance, bp)	Regions	Methods	Author	PMID	Sample
chr11	106669004	2069	GUCY1A2	intronic	HIVID	Yang et al .2018	30271481	Tumor
chr12	23545557	1859	ETNK1(dist=701949),SOX5(dist=139674)	intergenic	HIVID	Yang et al .2018	30271481	Tumor
chr12	28790941	1859	CCDC91(dist=87842),FAR2(dist=585657)	intergenic	HIVID	Yang et al .2018	30271481	Tumor
chr12	47245977	1859	SLC38A4(dist=26197),AMIGO2(dist=223513)	intergenic	HIVID	Yang et al .2018	30271481	Tumor
chr12	47389227	1859	SLC38A4(dist=169447),AMIGO2(dist=80263)	intergenic	HIVID	Yang et al .2018	30271481	Tumor
chr12	51369920	1859	HIGD1C(dist=5631),SLC11A2(dist=3646)	intergenic	HIVID	Yang et al .2018	30271481	Tumor
chr12	96172556	1859	NTN4	intronic	HIVID	Yang et al .2018	30271481	Tumor
chr12	104966011	1859	CHST11	intronic	HIVID	Yang et al .2018	30271481	Tumor
chr12	113319973	2189	RPH3A	intronic	HIVID	Yang et al .2018	30271481	Tumor
chr13	113062177	1859	SPACA7	intronic	HIVID	Yang et al .2018	30271481	Tumor
chr14	27829265	1859	MIR4307(dist=451334),LINC00645(dist=252529)	intergenic	HIVID	Yang et al .2018	30271481	Tumor
chr15	62311005	1859	VPS13C	intronic	HIVID	Yang et al .2018	30271481	Tumor
chr15	90454180	1829	C15orf38,C15orf38-AP3S2	intronic	HIVID	Yang et al .2018	30271481	Tumor
chr15	90475262	1740	C15orf38-AP3S2(dist=19040),ZNF710(dist=69490)	intergenic	HIVID	Yang et al .2018	30271481	Tumor
chr15	100370674	1859	DNM1P46(dist=23542),ADAMTS17(dist=140969)	intergenic	HIVID	Yang et al .2018	30271481	Tumor
chr16	5920761	1875	FAM86A(dist=772972),RBFOX1(dist=148371)	intergenic	HIVID	Yang et al .2018	30271481	Tumor
chr16	17830518	1869	XYLT1(dist=265780),MIR3180-1(dist=665517)	intergenic	HIVID	Yang et al .2018	30271481	Tumor
chr16	47590697	1859	PHKB	intronic	HIVID	Yang et al .2018	30271481	Tumor
chr16	56429086	1859	AMFR	intronic	HIVID	Yang et al .2018	30271481	Tumor
chr16	60014007	1859	LOC644649(dist=224912),CDH8(dist=1671908)	intergenic	HIVID	Yang et al .2018	30271481	Tumor
chr16	85908703	1859	COX4I1(dist=68096),IRF8(dist=24071)	intergenic	HIVID	Yang et al .2018	30271481	Tumor
chr17	6789224	1859	ALOX12P2	ncRNA_intronic	HIVID	Yang et al .2018	30271481	Tumor
chr17	15701143	1859	MEIS3P1(dist=8124),ADORA2B(dist=147088)	intergenic	HIVID	Yang et al .2018	30271481	Tumor
chr17	20503802	1859	CDRT15L2(dist=19578),CCDC144NL(dist=262906)	intergenic	HIVID	Yang et al .2018	30271481	Tumor
chr17	22256010	1802	MTRNR2L1(dist=232019),NONE(dist=NONE)	intergenic	HIVID	Yang et al .2018	30271481	Tumor
chr18	43278062	1859	SLC14A2(dist=15002),SLC14A1(dist=26030)	intergenic	HIVID	Yang et al .2018	30271481	Tumor
chr18	56783255	1873	OACYLP(dist=62809),SEC11C(dist=23870)	intergenic	HIVID	Yang et al .2018	30271481	Tumor
chr19	21788223	1823	ZNF429(dist=67144),ZNF100(dist=118620)	intergenic	HIVID	Yang et al .2018	30271481	Tumor
chr19	22079398	1862	ZNF43(dist=44528),ZNF208(dist=69499)	intergenic	HIVID	Yang et al .2018	30271481	Tumor
chr19	28355957	1962	LINC00662(dist=71109),LOC148145(dist=1100081)	intergenic	HIVID	Yang et al .2018	30271481	Tumor
chr19	36212541	1548	KMT2B	exonic	HIVID	Yang et al .2018	30271481	Tumor
chr19	43221850	1895	CEACAM8(dist=122768),PSG3(dist=3944)	intergenic	HIVID	Yang et al .2018	30271481	Tumor
chr2	75199743	1859	POLE4(dist=2884),TACR1(dist=73847)	intergenic	HIVID	Yang et al .2018	30271481	Tumor
chr2	135993004	1859	ZRANB3	intronic	HIVID	Yang et al .2018	30271481	Tumor
chr2	138342448	1862	THSD7B	intronic	HIVID	Yang et al .2018	30271481	Tumor
chr2	230832612	1859	FBXO36	intronic	HIVID	Yang et al .2018	30271481	Tumor
chr3	5979009	1859	MIR4790(dist=687069),GRM7(dist=923793)	intergenic	HIVID	Yang et al .2018	30271481	Tumor
chr3	6311117	1859	MIR4790(dist=1019177),GRM7(dist=591685)	intergenic	HIVID	Yang et al .2018	30271481	Tumor
chr3	40039035	1876	MYRIP	intronic	HIVID	Yang et al .2018	30271481	Tumor
chr3	55426355	1859	CACNA2D3(dist=317771),WNT5A(dist=73388)	intergenic	HIVID	Yang et al .2018	30271481	Tumor
chr3	82422017	1859	GBE1(dist=611067),NONE(dist=NONE)	intergenic	HIVID	Yang et al .2018	30271481	Tumor
chr3	100406742	1859	GPR128	intronic	HIVID	Yang et al .2018	30271481	Tumor
chr3	129881861	1868	FAM86HP(dist=51585),COL6A4P2(dist=49802)	intergenic	HIVID	Yang et al .2018	30271481	Tumor
chr3	139763920	1876	CLSTN2	intronic	HIVID	Yang et al .2018	30271481	Tumor
chr3	148068472	1903	ZIC1(dist=933966),AGTR1(dist=347186)	intergenic	HIVID	Yang et al .2018	30271481	Tumor
chr3	154314386	1859	GPR149(dist=166882),MME(dist=483050)	intergenic	HIVID	Yang et al .2018	30271481	Tumor
chr3	157061330	2069	VEPH1	intronic	HIVID	Yang et al .2018	30271481	Tumor
chr3	160764244	1895	PPM1L	intronic	HIVID	Yang et al .2018	30271481	Tumor
chr3	192952816	1040	MB21D2(dist=316866),HRASLS(dist=6101)	intergenic	HIVID	Yang et al .2018	30271481	Tumor
chr4	9671023	1707	MIR548I2(dist=113086),DRD5(dist=112235)	intergenic	HIVID	Yang et al .2018	30271481	Tumor
chr4	15840797	2895	CD38	intronic	HIVID	Yang et al .2018	30271481	Tumor
chr4	15840843	453	CD38	intronic	HIVID	Yang et al .2018	30271481	Tumor
chr4	52803762	1859	DCUN1D4(dist=20759),LRRC66(dist=56104)	intergenic	HIVID	Yang et al .2018	30271481	Tumor
chr4	58424427	1931	LOC255130(dist=352962),NONE(dist=NONE)	intergenic	HIVID	Yang et al .2018	30271481	Tumor
chr4	61840289	1859	NONE(dist=NONE),LPHN3(dist=522550)	intergenic	HIVID	Yang et al .2018	30271481	Tumor
chr4	99648051	1859	TSPAN5(dist=68239),EIF4E(dist=151556)	intergenic	HIVID	Yang et al .2018	30271481	Tumor
chr4	106653395	1895	GSTCD	intronic	HIVID	Yang et al .2018	30271481	Tumor
chr4	166602912	970	CPE(dist=183430),TLL1(dist=191498)	intergenic	HIVID	Yang et al .2018	30271481	Tumor
chr4	171186949	1859	AADAT(dist=175577),HSP90AA6P(dist=315672)	intergenic	HIVID	Yang et al .2018	30271481	Tumor
chr5	39244788	2063	FYB	intronic	HIVID	Yang et al .2018	30271481	Tumor
chr5	49894463	1859	EMB(dist=157229),PARP8(dist=67270)	intergenic	HIVID	Yang et al .2018	30271481	Tumor
chr5	86979814	256	CCNH(dist=270964),TMEM161B(dist=511209)	intergenic	HIVID	Yang et al .2018	30271481	Tumor
chr5	97330655	1859	RIOK2(dist=811650),RGMB(dist=774344)	intergenic	HIVID	Yang et al .2018	30271481	Tumor
chr5	106237209	1859	RAB9BP1(dist=1801410),EFNA5(dist=475381)	intergenic	HIVID	Yang et al .2018	30271481	Tumor
chr5	125073066	1859	ZNF608(dist=992261),GRAMD3(dist=622722)	intergenic	HIVID	Yang et al .2018	30271481	Tumor
chr6	19224703	1859	MIR548A1(dist=652592),ID4(dist=612898)	intergenic	HIVID	Yang et al .2018	30271481	Tumor
chr6	65161397	1859	EYS	intronic	HIVID	Yang et al .2018	30271481	Tumor
chr6	85312019	2034	KIAA1009(dist=374684),TBX18(dist=132138)	intergenic	HIVID	Yang et al .2018	30271481	Tumor
chr6	92503377	1859	MIR4643(dist=271922),EPHA7(dist=1446363)	intergenic	HIVID	Yang et al .2018	30271481	Tumor
chr6	117569427	1859	RFX6(dist=316101),VGLL2(dist=17294)	intergenic	HIVID	Yang et al .2018	30271481	Tumor
chr6	125129277	1859	NKAIN2	intronic	HIVID	Yang et al .2018	30271481	Tumor
chr7	54568947	1907	HPVC1(dist=298833),VSTM2A(dist=41072)	intergenic	HIVID	Yang et al .2018	30271481	Tumor
chr7	107151987	1859	COG5	intronic	HIVID	Yang et al .2018	30271481	Tumor
chr7	116014937	1859	TES(dist=116100),CAV2(dist=124718)	intergenic	HIVID	Yang et al .2018	30271481	Tumor
chr7	152326461	1859	LOC100128822(dist=163831),XRCC2(dist=17126)	intergenic	HIVID	Yang et al .2018	30271481	Tumor
chr8	13954130	1859	SGCZ	intronic	HIVID	Yang et al .2018	30271481	Tumor
chr8	47541849	1894	NONE(dist=NONE),LINC00293(dist=210659)	intergenic	HIVID	Yang et al .2018	30271481	Tumor
chr8	51787482	301	SNTG1(dist=82055),PXDNL(dist=444655)	intergenic	HIVID	Yang et al .2018	30271481	Tumor
chr8	58480345	1998	LINC00588(dist=283055),FAM110B(dist=426768)	intergenic	HIVID	Yang et al .2018	30271481	Tumor
chr8	88314361	1859	CNBD1	intronic	HIVID	Yang et al .2018	30271481	Tumor
chr8	136166366	1859	MIR30D(dist=349178),LOC286094(dist=80008)	intergenic	HIVID	Yang et al .2018	30271481	Tumor
chr8	139457541	1859	FAM135B	intronic	HIVID	Yang et al .2018	30271481	Tumor
chr9	21896878	1859	MTAP(dist=30909),C9orf53(dist=70260)	intergenic	HIVID	Yang et al .2018	30271481	Tumor
chr9	22760048	1965	FLJ35282	ncRNA_intronic	HIVID	Yang et al .2018	30271481	Tumor
chr9	25931813	1859	TUSC1(dist=252957),LOC100506422(dist=134860)	intergenic	HIVID	Yang et al .2018	30271481	Tumor
chr9	88375179	1859	AGTPBP1(dist=18235),LOC389765(dist=45738)	intergenic	HIVID	Yang et al .2018	30271481	Tumor
chrX	15432249	1835	PIR-FIGF	ncRNA_intronic	HIVID	Yang et al .2018	30271481	Tumor
chrX	50480823	1859	SHROOM4	intronic	HIVID	Yang et al .2018	30271481	Tumor
chrX	54745328	1859	GNL3L(dist=151608),ITIH6(dist=30004)	intergenic	HIVID	Yang et al .2018	30271481	Tumor
chrX	65786565	1859	HEPH(dist=299335),EDA2R(dist=28917)	intergenic	HIVID	Yang et al .2018	30271481	Tumor
chrX	68574123	1859	PJA1(dist=188758),FAM155B(dist=150955)	intergenic	HIVID	Yang et al .2018	30271481	Tumor
chrX	74216397	1989	KIAA2022(dist=71110),ABCB7(dist=56708)	intergenic	HIVID	Yang et al .2018	30271481	Tumor
chrX	90931486	1864	PABPC5(dist=237903),PCDH11X(dist=102774)	intergenic	HIVID	Yang et al .2018	30271481	Tumor
chrX	130599116	1859	IGSF1(dist=175713),OR13H1(dist=78932)	intergenic	HIVID	Yang et al .2018	30271481	Tumor

Chromosome	Integration site in host genome	Integration site in virus genome	Gene (distance, bp)	Regions	Methods	Author	PMID	Sample
chrX	149273194	1859	LOC100272228(dist=88176),MIR2114(dist=123045)	intergenic	HIVID	Yang et al .2018	30271481	Tumor
chrY	9947087	1859	TTTY23(dist=197516),NONE(dist=NONE)	intergenic	HIVID	Yang et al .2018	30271481	Tumor
chrY	21419894	1859	TTTY14(dist=180592),BCORP1(dist=197423)	intergenic	HIVID	Yang et al .2018	30271481	Tumor
chrY	21498245	1871	TTTY14(dist=258943),BCORP1(dist=119072)	intergenic	HIVID	Yang et al .2018	30271481	Tumor
chr15	20310359	1784	NONE(dist=NONE),CHEK2P2(dist=177638)	intergenic	HIVID	Yang et al .2018	30271481	Tumor
chr15	21317410	1784	LOC348120(dist=118777),LOC646214(dist=615104)	intergenic	HIVID	Yang et al .2018	30271481	Tumor
chr17	13567973	789	HS3ST3A1(dist=62729),CDRT15P1(dist=359842)	intergenic	HIVID	Yang et al .2018	30271481	Tumor
chr2	68530090	1828	CNRIP1	intronic	HIVID	Yang et al .2018	30271481	Tumor
chr9	21798515	1843	MIR31HG(dist=238818),MTAP(dist=4120)	intergenic	HIVID	Yang et al .2018	30271481	Tumor
chr9	22047158	1799	CDKN2B-AS1	ncRNA_intronic	HIVID	Yang et al .2018	30271481	Tumor
chr9	32603632	1828	NDUFB6(dist=30450),TAF1L(dist=25820)	intergenic	HIVID	Yang et al .2018	30271481	Tumor
chr1	8084038	2355	ERRFI1	intronic	HIVID	Yang et al .2018	30271481	Tumor
chr1	55963448	172	MIR4422(dist=272052),PPAP2B(dist=996971)	intergenic	HIVID	Yang et al .2018	30271481	Tumor
chr1	121484064	2034	EMBP1(dist=170378),NONE(dist=NONE)	intergenic	HIVID	Yang et al .2018	30271481	Tumor
chr1	121484858	1839	EMBP1(dist=171172),NONE(dist=NONE)	intergenic	HIVID	Yang et al .2018	30271481	Tumor
chr1	224201314	1977	TP53BP2(dist=167640),FBXO28(dist=100475)	intergenic	HIVID	Yang et al .2018	30271481	Tumor
chr10	313209	2633	ZMYND11(dist=12632),DIP2C(dist=6921)	intergenic	HIVID	Yang et al .2018	30271481	Tumor
chr10	39147047	1887	ACTR3BP5(dist=155676),NONE(dist=NONE)	intergenic	HIVID	Yang et al .2018	30271481	Tumor
chr10	88512743	1837	LDB3(dist=16919),BMPR1A(dist=3653)	intergenic	HIVID	Yang et al .2018	30271481	Tumor
chr10	110954436	3206	RNU6-53	ncRNA_intronic	HIVID	Yang et al .2018	30271481	Tumor
chr11	5011291	1606	MMP26	intronic	HIVID	Yang et al .2018	30271481	Tumor
chr11	30193847	1574	KCNA4(dist=155270),FSHB(dist=58716)	intergenic	HIVID	Yang et al .2018	30271481	Tumor
chr11	69785064	1767	FGF3(dist=150872),ANO1(dist=139344)	intergenic	HIVID	Yang et al .2018	30271481	Tumor
chr11	73066252	1740	ARHGEF17	intronic	HIVID	Yang et al .2018	30271481	Tumor
chr11	120210369	396	ARHGEF12	intronic	HIVID	Yang et al .2018	30271481	Tumor
chr11	120210371	2397	ARHGEF12	intronic	HIVID	Yang et al .2018	30271481	Tumor
chr12	2250290	439	CACNA1C	intronic	HIVID	Yang et al .2018	30271481	Tumor
chr12	29035977	1686	CCDC91(dist=332878),FAR2(dist=340621)	intergenic	HIVID	Yang et al .2018	30271481	Tumor
chr12	50261607	1263	FAIM2	UTR3	HIVID	Yang et al .2018	30271481	Tumor
chr12	113319973	2189	RPH3A	intronic	HIVID	Yang et al .2018	30271481	Tumor
chr13	20017884	791	TPTE2	intronic	HIVID	Yang et al .2018	30271481	Tumor
chr13	93964290	1941	GPC6	intronic	HIVID	Yang et al .2018	30271481	Tumor
chr14	89686394	2310	FOXN3	intronic	HIVID	Yang et al .2018	30271481	Tumor
chr18	57129474	1616	CCBE1	intronic	HIVID	Yang et al .2018	30271481	Tumor
chr19	27732153	1950	NONE(dist=NONE),LINC00662(dist=549248)	intergenic	HIVID	Yang et al .2018	30271481	Tumor
chr19	27735568	1837	NONE(dist=NONE),LINC00662(dist=545833)	intergenic	HIVID	Yang et al .2018	30271481	Tumor
chr19	36212557	1808	KMT2B	exonic	HIVID	Yang et al .2018	30271481	Tumor
chr19	57181287	2290	ZNF835	intronic	HIVID	Yang et al .2018	30271481	Tumor
chr2	68133640	692	ETAA1(dist=496107),C1D(dist=135692)	intergenic	HIVID	Yang et al .2018	30271481	Tumor
chr2	89875629	912	MIR4436A(dist=763661),LOC654342(dist=1949080)	intergenic	HIVID	Yang et al .2018	30271481	Tumor
chr2	92281252	82	ACTR3BP2(dist=150756),NONE(dist=NONE)	intergenic	HIVID	Yang et al .2018	30271481	Tumor
chr2	109815827	1847	SH3RF3	intronic	HIVID	Yang et al .2018	30271481	Tumor
chr2	109815854	1995	SH3RF3	intronic	HIVID	Yang et al .2018	30271481	Tumor
chr2	109815855	483	SH3RF3	intronic	HIVID	Yang et al .2018	30271481	Tumor
chr2	109815904	549	SH3RF3	intronic	HIVID	Yang et al .2018	30271481	Tumor
chr2	109815912	2496	SH3RF3	intronic	HIVID	Yang et al .2018	30271481	Tumor
chr2	109815922	2496	SH3RF3	intronic	HIVID	Yang et al .2018	30271481	Tumor
chr2	109815939	549	SH3RF3	intronic	HIVID	Yang et al .2018	30271481	Tumor
chr2	109815947	2496	SH3RF3	intronic	HIVID	Yang et al .2018	30271481	Tumor
chr2	109815966	2678	SH3RF3	intronic	HIVID	Yang et al .2018	30271481	Tumor
chr2	109815969	483	SH3RF3	intronic	HIVID	Yang et al .2018	30271481	Tumor
chr2	109816029	483	SH3RF3	intronic	HIVID	Yang et al .2018	30271481	Tumor
chr2	109816031	1847	SH3RF3	intronic	HIVID	Yang et al .2018	30271481	Tumor
chr2	109816058	1388	SH3RF3	intronic	HIVID	Yang et al .2018	30271481	Tumor
chr2	109816058	549	SH3RF3	intronic	HIVID	Yang et al .2018	30271481	Tumor
chr2	109816066	2496	SH3RF3	intronic	HIVID	Yang et al .2018	30271481	Tumor
chr2	109816106	1847	SH3RF3	intronic	HIVID	Yang et al .2018	30271481	Tumor
chr2	109816115	868	SH3RF3	intronic	HIVID	Yang et al .2018	30271481	Tumor
chr2	109816124	483	SH3RF3	intronic	HIVID	Yang et al .2018	30271481	Tumor
chr2	109816138	1800	SH3RF3	intronic	HIVID	Yang et al .2018	30271481	Tumor
chr2	109816164	483	SH3RF3	intronic	HIVID	Yang et al .2018	30271481	Tumor
chr2	109816185	868	SH3RF3	intronic	HIVID	Yang et al .2018	30271481	Tumor
chr2	109816191	1847	SH3RF3	intronic	HIVID	Yang et al .2018	30271481	Tumor
chr2	109816204	483	SH3RF3	intronic	HIVID	Yang et al .2018	30271481	Tumor
chr2	160626657	887	CD302,LY75-CD302	UTR3	HIVID	Yang et al .2018	30271481	Tumor
chr20	6693452	1820	FERMT1(dist=589261),BMP2(dist=55293)	intergenic	HIVID	Yang et al .2018	30271481	Tumor
chr20	15273911	2508	MACROD2	intronic	HIVID	Yang et al .2018	30271481	Tumor
chr20	19472856	874	SLC24A3	intronic	HIVID	Yang et al .2018	30271481	Tumor
chr21	9825530	864	MIR3648,MIR3687	upstream	HIVID	Yang et al .2018	30271481	Tumor
chr21	16259772	1821	LOC388813(dist=244344),NRIP1(dist=73784)	intergenic	HIVID	Yang et al .2018	30271481	Tumor
chr21	35254544	731	ITSN1	intronic	HIVID	Yang et al .2018	30271481	Tumor
chr21	47191315	2083	SLC19A1(dist=228930),LOC100129027(dist=56440)	intergenic	HIVID	Yang et al .2018	30271481	Tumor
chr3	115968479	1767	LSAMP	intronic	HIVID	Yang et al .2018	30271481	Tumor
chr4	9671023	1707	MIR548I2(dist=113086),DRD5(dist=112235)	intergenic	HIVID	Yang et al .2018	30271481	Tumor
chr4	18491815	598	LCORL(dist=468332),SLIT2(dist=1763420)	intergenic	HIVID	Yang et al .2018	30271481	Tumor
chr4	25709592	1767	SLC34A2(dist=29224),SEL1L3(dist=39457)	intergenic	HIVID	Yang et al .2018	30271481	Tumor
chr4	49637975	1673	CWH43(dist=573880),NONE(dist=NONE)	intergenic	HIVID	Yang et al .2018	30271481	Tumor
chr4	62445480	2492	LPHN3	intronic	HIVID	Yang et al .2018	30271481	Tumor
chr4	166602912	970	CPE(dist=183430),TLL1(dist=191498)	intergenic	HIVID	Yang et al .2018	30271481	Tumor
chr4	178387590	1822	AGA(dist=23933),LOC285501(dist=262321)	intergenic	HIVID	Yang et al .2018	30271481	Tumor
chr4	178395436	148	AGA(dist=31779),LOC285501(dist=254475)	intergenic	HIVID	Yang et al .2018	30271481	Tumor
chr4	190907928	1718	FRG1(dist=23569),LOC100288255(dist=37595)	intergenic	HIVID	Yang et al .2018	30271481	Tumor
chr5	1295519	1622	TERT	upstream	HIVID	Yang et al .2018	30271481	Tumor
chr5	1296139	986	TERT	upstream	HIVID	Yang et al .2018	30271481	Tumor
chr5	11282985	2416	CTNND2	intronic	HIVID	Yang et al .2018	30271481	Tumor
chr5	36609066	722	SLC1A3	UTR3	HIVID	Yang et al .2018	30271481	Tumor
chr5	44827857	2780	MRPS30(dist=12239),HCN1(dist=427195)	intergenic	HIVID	Yang et al .2018	30271481	Tumor
chr5	112449264	2625	MCC	intronic	HIVID	Yang et al .2018	30271481	Tumor
chr6	115877348	478	HS3ST5(dist=1493307),FRK(dist=385345)	intergenic	HIVID	Yang et al .2018	30271481	Tumor
chr6	163098967	1137	PARK2	intronic	HIVID	Yang et al .2018	30271481	Tumor
chr6	163098968	3212	PARK2	intronic	HIVID	Yang et al .2018	30271481	Tumor
chr6	163099122	142	PARK2	intronic	HIVID	Yang et al .2018	30271481	Tumor
chr6	163099137	53	PARK2	intronic	HIVID	Yang et al .2018	30271481	Tumor
chr7	55716984	1784	VOPP1(dist=76784),FKBP9L(dist=31783)	intergenic	HIVID	Yang et al .2018	30271481	Tumor

Supplementary Table S8 Continued

Chromosome	Integration site in host genome	Integration site in virus genome	Gene (distance, bp)	Regions	Methods	Author	PMID	Sample
chr8	51787482	301	SNTG1(dist=82055),PXDNL(dist=444655)	intergenic	HIVID	Yang et al .2018	30271481	Tumor
chr9	67320759	82	AQP7P1(dist=31267),FAM27B(dist=472170)	intergenic	HIVID	Yang et al .2018	30271481	Tumor
chrX	34887192	1128	TMEM47(dist=211787),FAM47B(dist=73721)	intergenic	HIVID	Yang et al .2018	30271481	Tumor
chrX	123220317	1422	STAG2	intronic	HIVID	Yang et al .2018	30271481	Tumor
chrY	13137856	483	NONE(dist=NONE),GYG2P1(dist=1380059)	intergenic	HIVID	Yang et al .2018	30271481	Tumor
chr1	192472370	35	RGS21(dist=135956),RGS1(dist=72487)	intergenic	HIVID	Yang et al .2018	30271481	Tumor
chr2	33141618	2403	LINC00486	ncRNA_intronic	HIVID	Yang et al .2018	30271481	Tumor
chr3	34696173	11	PDCD6IP(dist=784974),LOC101928135(dist=221116)	intergenic	HIVID	Yang et al .2018	30271481	Tumor
chr3	43108938	46	FAM198A(dist=9731),POMGNT2(dist=11783)	intergenic	HIVID	Yang et al .2018	30271481	Tumor
chr3	90386070	11	EPHA3(dist=854786),NONE(dist=NONE)	intergenic	HIVID	Yang et al .2018	30271481	Tumor
chr3	108921567	11	LINC00488(dist=17459),DPPA2(dist=91068)	intergenic	HIVID	Yang et al .2018	30271481	Tumor
chr3	162957435	4	LINC01192	ncRNA_intronic	HIVID	Yang et al .2018	30271481	Tumor
chr3	197900131	1703	FAM157A	intronic	HIVID	Yang et al .2018	30271481	Tumor
chr4	3374704	2881	RGS12	intronic	HIVID	Yang et al .2018	30271481	Tumor
chr4	14759585	11	LINC01085(dist=617909),CPEB2-AS1(dist=152000)	intergenic	HIVID	Yang et al .2018	30271481	Tumor
chr4	26926133	11	STIM2	intronic	HIVID	Yang et al .2018	30271481	Tumor
chr4	79271759	26	FRAS1	intronic	HIVID	Yang et al .2018	30271481	Tumor
chr4	130940783	28	LOC101927282(dist=248150),LOC101927305(dist=1745210)	intergenic	HIVID	Yang et al .2018	30271481	Tumor
chr4	146293205	53	OTUD4(dist=192373),SMAD1(dist=109746)	intergenic	HIVID	Yang et al .2018	30271481	Tumor
chr5	45349323	27	HCN1	intronic	HIVID	Yang et al .2018	30271481	Tumor
chr6	48450996	2269	PTCHD4(dist=414571),MUT(dist=947077)	intergenic	HIVID	Yang et al .2018	30271481	Tumor
chr6	81511029	11	BCKDHB(dist=455042),FAM46A(dist=944418)	intergenic	HIVID	Yang et al .2018	30271481	Tumor
chr8	121363275	30	COL14A1	intronic	HIVID	Yang et al .2018	30271481	Tumor
chr9	113556040	111	MUSK	intronic	HIVID	Yang et al .2018	30271481	Tumor
chr10	10002179	5	LOC101928272(dist=664623),LOC101928298(dist=98506)	intergenic	HIVID	Yang et al .2018	30271481	Tumor
chr10	93265697	33	HECTD2-AS1	ncRNA_intronic	HIVID	Yang et al .2018	30271481	Tumor
chr10	127636445	1	FANK1	intronic	HIVID	Yang et al .2018	30271481	Tumor
chr10	135524648	1703	FRG2B(dist=84349),NONE(dist=NONE)	intergenic	HIVID	Yang et al .2018	30271481	Tumor
chr12	4452746	53	C12orf5	intronic	HIVID	Yang et al .2018	30271481	Tumor
chr12	55185203	11	DCD(dist=142926),MUCL1(dist=63096)	intergenic	HIVID	Yang et al .2018	30271481	Tumor
chr12	55740744	50	OR6C3(dist=14324),OR6C75(dist=18151)	intergenic	HIVID	Yang et al .2018	30271481	Tumor
chr12	133841551	1703	ANHX(dist=29129),NONE(dist=NONE)	intergenic	HIVID	Yang et al .2018	30271481	Tumor
chr14	27930950	4	LOC101927081(dist=547001),LINC00645(dist=150844)	intergenic	HIVID	Yang et al .2018	30271481	Tumor
chr14	48659134	11	LINC00648(dist=394917),RPS29(dist=1384256)	intergenic	HIVID	Yang et al .2018	30271481	Tumor
chr14	71016819	146	ADAM20(dist=15087),MED6(dist=33119)	intergenic	HIVID	Yang et al .2018	30271481	Tumor
chr14	84108197	55	NONE(dist=NONE),LINC00911(dist=1752026)	intergenic	HIVID	Yang et al .2018	30271481	Tumor
chr14	84108317	2	NONE(dist=NONE),LINC00911(dist=1751906)	intergenic	HIVID	Yang et al .2018	30271481	Tumor
chr14	86354786	11	FLRT2(dist=260516),LOC101928767(dist=46236)	intergenic	HIVID	Yang et al .2018	30271481	Tumor
chr15	64621528	53	CSNK1G1	intronic	HIVID	Yang et al .2018	30271481	Tumor
chr16	916918	3200	LMF1	intronic	HIVID	Yang et al .2018	30271481	Tumor
chr18	32788412	53	MAPRE2(dist=64980),ZNF397(dist=32582)	intergenic	HIVID	Yang et al .2018	30271481	Tumor
chr18	78016240	1703	PARD6G(dist=10843),NONE(dist=NONE)	intergenic	HIVID	Yang et al .2018	30271481	Tumor
chr18	78016316	1703	PARD6G(dist=10919),NONE(dist=NONE)	intergenic	HIVID	Yang et al .2018	30271481	Tumor
chr19	56336467	2	NLRP11	intronic	HIVID	Yang et al .2018	30271481	Tumor
chr21	48119869	1703	PRMT2(dist=34833),NONE(dist=NONE)	intergenic	HIVID	Yang et al .2018	30271481	Tumor
chrX	108359206	53	LOC101928358(dist=377073),GUCY2F(dist=256929)	intergenic	HIVID	Yang et al .2018	30271481	Tumor
chrX	155259822	1703	DDX11L16(dist=1974),NONE(dist=NONE)	intergenic	HIVID	Yang et al .2018	30271481	Tumor
chrX	155259893	1703	DDX11L16(dist=2045),NONE(dist=NONE)	intergenic	HIVID	Yang et al .2018	30271481	Tumor
chrX	155260014	1703	DDX11L16(dist=2166),NONE(dist=NONE)	intergenic	HIVID	Yang et al .2018	30271481	Tumor
chrX	155260310	1703	DDX11L16(dist=2462),NONE(dist=NONE)	intergenic	HIVID	Yang et al .2018	30271481	Tumor
chrY	59362828	1703	DDX11L16(dist=1974),NONE(dist=NONE)	intergenic	HIVID	Yang et al .2018	30271481	Tumor
chrY	59362899	1703	DDX11L16(dist=2045),NONE(dist=NONE)	intergenic	HIVID	Yang et al .2018	30271481	Tumor
chrY	59363315	1703	DDX11L16(dist=2461),NONE(dist=NONE)	intergenic	HIVID	Yang et al .2018	30271481	Tumor
chr1	35034978	27	C1orf94(dist=350247),GJB5(dist=185670)	intergenic	HIVID	Yang et al .2018	30271481	Tumor
chr1	174350107	11	RABGAP1L	intronic	HIVID	Yang et al .2018	30271481	Tumor
chr1	174815656	111	RABGAP1L	intronic	HIVID	Yang et al .2018	30271481	Tumor
chr1	187316205	28	LINC01036	ncRNA_intronic	HIVID	Yang et al .2018	30271481	Tumor
chr1	248333160	118	OR2M5(dist=23772),OR2M2(dist=10128)	intergenic	HIVID	Yang et al .2018	30271481	Tumor
chr2	103185998	11	SLC9A4(dist=35567),SLC9A2(dist=50168)	intergenic	HIVID	Yang et al .2018	30271481	Tumor
chr2	113264573	11	TTL	intronic	HIVID	Yang et al .2018	30271481	Tumor
chr2	161616871	37	RBMS1(dist=266553),TANK(dist=376595)	intergenic	HIVID	Yang et al .2018	30271481	Tumor
chr2	194080327	11	PCGEM1(dist=438702),LOC101927406(dist=1128666)	intergenic	HIVID	Yang et al .2018	30271481	Tumor
chr3	24134196	5	NR1D2(dist=112087),LINC00691(dist=7269)	intergenic	HIVID	Yang et al .2018	30271481	Tumor
chr3	34457918	56	PDCD6IP(dist=546719),LOC101928135(dist=459371)	intergenic	HIVID	Yang et al .2018	30271481	Tumor
chr3	89398025	53	EPHA3	intronic	HIVID	Yang et al .2018	30271481	Tumor
chr3	107607029	114	LINC00636	ncRNA_intronic	HIVID	Yang et al .2018	30271481	Tumor
chr3	132668033	11	NPHP3-AS1(dist=74983),TMEM108(dist=89099)	intergenic	HIVID	Yang et al .2018	30271481	Tumor
chr3	187145448	46	RTP4(dist=56079),SST(dist=241246)	intergenic	HIVID	Yang et al .2018	30271481	Tumor
chr4	10169	1703	NONE(dist=NONE),ZNF595(dist=43010)	intergenic	HIVID	Yang et al .2018	30271481	Tumor
chr4	103129369	111	BANK1(dist=133400),SLC39A8(dist=42829)	intergenic	HIVID	Yang et al .2018	30271481	Tumor
chr4	107495506	26	GIMD1(dist=206914),DKK2(dist=347453)	intergenic	HIVID	Yang et al .2018	30271481	Tumor
chr4	108131010	11	DKK2(dist=173557),PAPSS1(dist=403812)	intergenic	HIVID	Yang et al .2018	30271481	Tumor
chr4	127987003	41	MIR2054(dist=1558541),INTU(dist=567084)	intergenic	HIVID	Yang et al .2018	30271481	Tumor
chr5	10279	1703	NONE(dist=NONE),PLEKHG4B(dist=130094)	intergenic	HIVID	Yang et al .2018	30271481	Tumor
chr5	10501	1703	NONE(dist=NONE),PLEKHG4B(dist=129872)	intergenic	HIVID	Yang et al .2018	30271481	Tumor
chr5	10687	1703	NONE(dist=NONE),PLEKHG4B(dist=129686)	intergenic	HIVID	Yang et al .2018	30271481	Tumor
chr5	1296677	581	TERT(dist=1515),MIR4457(dist=12748)	intergenic	HIVID	Yang et al .2018	30271481	Tumor
chr5	1296678	509	TERT(dist=1516),MIR4457(dist=12747)	intergenic	HIVID	Yang et al .2018	30271481	Tumor
chr5	1296678	581	TERT(dist=1516),MIR4457(dist=12747)	intergenic	HIVID	Yang et al .2018	30271481	Tumor
chr5	1297002	2390	TERT(dist=1840),MIR4457(dist=12423)	intergenic	HIVID	Yang et al .2018	30271481	Tumor
chr5	50629828	51	LOC100287592(dist=363807),LOC642366(dist=38743)	intergenic	HIVID	Yang et al .2018	30271481	Tumor
chr6	133793412	11	EYA4	intronic	HIVID	Yang et al .2018	30271481	Tumor
chr6	156649297	11	NOX3(dist=872260),ARID1B(dist=449767)	intergenic	HIVID	Yang et al .2018	30271481	Tumor
chr9	122386897	28	BRINP1(dist=255158),MIR147A(dist=620360)	intergenic	HIVID	Yang et al .2018	30271481	Tumor
chr10	18322863	27	SLC39A12	intronic	HIVID	Yang et al .2018	30271481	Tumor
chr10	22416114	111	DNAJC1(dist=123464),EBLN1(dist=81629)	intergenic	HIVID	Yang et al .2018	30271481	Tumor
chr10	25807482	11	GPR158	intronic	HIVID	Yang et al .2018	30271481	Tumor
chr10	111575404	2	RNU6-53P(dist=575914),XPNPEP1(dist=49120)	intergenic	HIVID	Yang et al .2018	30271481	Tumor
chr11	48872429	11	OR4A47(dist=361155),TRIM49B(dist=180723)	intergenic	HIVID	Yang et al .2018	30271481	Tumor
chr12	3611589	123	PRMT8	intronic	HIVID	Yang et al .2018	30271481	Tumor
chr12	13547722	11	LINC01559(dist=18043),GRIN2B(dist=166688)	intergenic	HIVID	Yang et al .2018	30271481	Tumor
chr14	26251285	53	STXBP6(dist=732190),NOVA1(dist=663804)	intergenic	HIVID	Yang et al .2018	30271481	Tumor
chr16	48052676	27	LOC101927132(dist=110334),ABCC12(dist=64208)	intergenic	HIVID	Yang et al .2018	30271481	Tumor
chr18	105574	1750	LOC102723376(dist=89644),ROCK1P1(dist=3491)	intergenic	HIVID	Yang et al .2018	30271481	Tumor

Chromosome	Integration site in host genome	Integration site in virus genome	Gene (distance, bp)	Regions	Methods	Author	PMID	Sample
chr18	8060728	5	PTPRM	intronic	HIVID	Yang et al .2018	30271481	Tumor
chr18	13979042	11	MC2R(dist=63336),NONE(dist=NONE)	intergenic	HIVID	Yang et al .2018	30271481	Tumor
chr18	25999785	56	CDH2(dist=242340),MIR302F(dist=1879091)	intergenic	HIVID	Yang et al .2018	30271481	Tumor
chr18	37770827	32	LINC01477(dist=91630),KC6(dist=1289409)	intergenic	HIVID	Yang et al .2018	30271481	Tumor
chr21	10770051	30	TEKT4P2(dist=801457),TPTE(dist=136136)	intergenic	HIVID	Yang et al .2018	30271481	Tumor
chrX	66147965	41	EDA2R(dist=288825),AR(dist=615909)	intergenic	HIVID	Yang et al .2018	30271481	Tumor
chrX	110835215	55	LINC00890(dist=69588),ALG13(dist=89131)	intergenic	HIVID	Yang et al .2018	30271481	Tumor
chrX	110882534	28	LINC00890(dist=116907),ALG13(dist=41812)	intergenic	HIVID	Yang et al .2018	30271481	Tumor
chrY	5477394	11	PCDH11Y	intronic	HIVID	Yang et al .2018	30271481	Tumor
chr11	116264252	1823	LOC283143(dist=633334),BUD13(dist=354634)	intergenic	HIVID	Yang et al .2018	30271481	Tumor
chr11	116264255	1905	LOC283143(dist=633337),BUD13(dist=354631)	intergenic	HIVID	Yang et al .2018	30271481	Tumor
chr4	33213605	337		intergenic	single-cell genome sequenci	Duan et al. 2018	29327728	Tumor
chr5	1295570	1813		intergenic	single-cell genome sequenci	Duan et al. 2018	29327728	Tumor
chr10	12429944	1843	CAMK1D	intronic	single-cell genome sequenci	Duan et al. 2018	29327728	Tumor
chr10	12470206	2355	CAMK1D	intronic	single-cell genome sequenci	Duan et al. 2018	29327728	Tumor
chr14	55413673	279		intergenic	single-cell genome sequenci	Duan et al. 2018	29327728	Tumor
chr20	1021142	1573		intergenic	single-cell genome sequenci	Duan et al. 2018	29327728	Tumor
chr5	1295938	1400		intergenic	single-cell genome sequenci	Duan et al. 2018	29327728	Tumor
chr10	87089429	2636		intergenic	single-cell genome sequenci	Duan et al. 2018	29327728	Tumor
chr17	19830214	985		intergenic	single-cell genome sequenci	Duan et al. 2018	29327728	Tumor
chr17	20338154	2390		intergenic	single-cell genome sequenci	Duan et al. 2018	29327728	Tumor
chr17	20631658	345		intergenic	single-cell genome sequenci	Duan et al. 2018	29327728	Tumor
			TERT		Virome capture sequence	Tatsuno et al. 2019	31320595	Tumor
			KMT2B		Virome capture sequence	Tatsuno et al. 2019	31320595	Tumor
			LINC00486		Virome capture sequence	Tatsuno et al. 2019	31320595	Tumor
			RNR2		Virome capture sequence	Tatsuno et al. 2019	31320595	Tumor
			FN1		Virome capture sequence	Tatsuno et al. 2019	31320595	Tumor
			HS6ST3		Virome capture sequence	Tatsuno et al. 2019	31320595	Tumor
			KNG1		Virome capture sequence	Tatsuno et al. 2019	31320595	Tumor
			LINC00333		Virome capture sequence	Tatsuno et al. 2019	31320595	Tumor
			MAGI2		Virome capture sequence	Tatsuno et al. 2019	31320595	Tumor
			ROCK1		Virome capture sequence	Tatsuno et al. 2019	31320595	Tumor
			CWH43		Virome capture sequence	Tatsuno et al. 2019	31320595	Tumor
			DDX11L1		Virome capture sequence	Tatsuno et al. 2019	31320595	Tumor
			DDX11L16		Virome capture sequence	Tatsuno et al. 2019	31320595	Tumor
			DDX11L2		Virome capture sequence	Tatsuno et al. 2019	31320595	Tumor
			EYA1		Virome capture sequence	Tatsuno et al. 2019	31320595	Tumor
			MACROD2		Virome capture sequence	Tatsuno et al. 2019	31320595	Tumor
			CDH12		Virome capture sequence	Tatsuno et al. 2019	31320595	Tumor
			DBET		Virome capture sequence	Tatsuno et al. 2019	31320595	Tumor
			DCC		Virome capture sequence	Tatsuno et al. 2019	31320595	Tumor
			PTPRD		Virome capture sequence	Tatsuno et al. 2019	31320595	Tumor
			RALYL		Virome capture sequence	Tatsuno et al. 2019	31320595	Tumor
			ZFPM2-AS1		Virome capture sequence	Tatsuno et al. 2019	31320595	Tumor
			ZNF595		Virome capture sequence	Tatsuno et al. 2019	31320595	Tumor
			CCNE1		Virome capture sequence	Tatsuno et al. 2019	31320595	Tumor
			CCNA2		Virome capture sequence	Tatsuno et al. 2019	31320595	Tumor
			HGF		Virome capture sequence	Tatsuno et al. 2019	31320595	Tumor
			CSMD3		Virome capture sequence	Tatsuno et al. 2019	31320595	Tumor
			AR		Virome capture sequence	Tatsuno et al. 2019	31320595	Tumor
			NF2		Virome capture sequence	Tatsuno et al. 2019	31320595	Tumor
			TERT	promoter	Deep transcriptome sequenc	Jin et al. 2019	31429776	Tumor
			TERT	promoter	Deep transcriptome sequenc	Jin et al. 2019	31429776	Tumor
			TERT	promoter	Deep transcriptome sequenc	Jin et al. 2019	31429776	Tumor
			GATA3	non-coding exon	Deep transcriptome sequenc	Jin et al. 2019	31429776	Tumor
			GATA3	non-coding exon	Deep transcriptome sequenc	Jin et al. 2019	31429776	Tumor
			DTNA	intron	Deep transcriptome sequenc	Jin et al. 2019	31429776	Tumor
			DTNA	intron	Deep transcriptome sequenc	Jin et al. 2019	31429776	Tumor
			SCO1	intron	Deep transcriptome sequenc	Jin et al. 2019	31429776	Tumor
			SCO1	intron	Deep transcriptome sequenc	Jin et al. 2019	31429776	Tumor
			AHCYL1	intron	Deep transcriptome sequenc	Jin et al. 2019	31429776	Tumor
			ADPRM	intron	Deep transcriptome sequenc	Jin et al. 2019	31429776	Tumor
			KMT2B	intron	Deep transcriptome sequenc	Jin et al. 2019	31429776	Tumor
			DISP1	intron	Deep transcriptome sequenc	Jin et al. 2019	31429776	Tumor
			PARP6	intron	Deep transcriptome sequenc	Jin et al. 2019	31429776	Tumor
			TGM2	intron	Deep transcriptome sequenc	Jin et al. 2019	31429776	Tumor
			WWOX	intron	Deep transcriptome sequenc	Jin et al. 2019	31429776	Tumor
			ZC3H3	intron	Deep transcriptome sequenc	Jin et al. 2019	31429776	Tumor
			AIP	intron	Deep transcriptome sequenc	Jin et al. 2019	31429776	Tumor
			ATRNL1	intron	Deep transcriptome sequenc	Jin et al. 2019	31429776	Tumor
			ATRNL1	intron	Deep transcriptome sequenc	Jin et al. 2019	31429776	Tumor
			DDX3X	intron	Deep transcriptome sequenc	Jin et al. 2019	31429776	Tumor
			EEF2KMT	intron	Deep transcriptome sequenc	Jin et al. 2019	31429776	Tumor
			MARCH8	intron	Deep transcriptome sequenc	Jin et al. 2019	31429776	Tumor
			RAPGEF5	intron	Deep transcriptome sequenc	Jin et al. 2019	31429776	Tumor
			FAS	intron	Deep transcriptome sequenc	Jin et al. 2019	31429776	Tumor
			KRT32	coding exon	Deep transcriptome sequenc	Jin et al. 2019	31429776	Tumor
			PHACTR4	3'UTR	Deep transcriptome sequenc	Jin et al. 2019	31429776	Tumor
			SON	3'UTR	Deep transcriptome sequenc	Jin et al. 2019	31429776	Tumor
			GATA3-AS1	non-coding exon	Deep transcriptome sequenc	Jin et al. 2019	31429776	Tumor
1			COP1		Alu-PCR	Wong et al.2019	31473360	Tumor
4			CCNA2		Alu-PCR	Wong et al.2019	31473360	Tumor
5			TERT		Alu-PCR	Wong et al.2019	31473360	Tumor
5			TERT		Alu-PCR	Wong et al.2019	31473360	Tumor
17			CNTNAP1		Alu-PCR	Wong et al.2019	31473360	Tumor
19			KMT2B		Alu-PCR	Wong et al.2019	31473360	Tumor
19			ZNF699		Alu-PCR	Wong et al.2019	31473360	Tumor
1	50330828	1263	AGBL4	intron	WGS	Li et al. 2019	32350851	Tumor
1	34654686		*C1orf94*	*intron*	WGS	Li et al. 2019	32350851	Tumor
1	10188	2154	DDX11L1	promoter	WGS	Li et al. 2019	32350851	Tumor
1	67674894	2491	*IL23R*	*intron*	WGS	Li et al. 2019	32350851	Tumor
1	121484896	799	NA	intergenic	WGS	Li et al. 2019	32350851	Tumor
1	87682833	885	NA	intergenic	WGS	Li et al. 2019	32350851	Tumor
1	106922020		NA	intergenic	WGS	Li et al. 2019	32350851	Tumor

Chromosome	Integration site in host genome	Integration site in virus genome	Gene (distance, bp)	Regions	Methods	Author	PMID	Sample
1	103100123		NA	intergenic	WGS	Li et al. 2019	32350851	Tumor
1	121485350		NA	intergenic	WGS	Li et al. 2019	32350851	Tumor
1	1709148		NADK	intron	WGS	Li et al. 2019	32350851	Tumor
1	120569613	1600	*NOTCH2*	intron	WGS	Li et al. 2019	32350851	Tumor
1	34654566				WGS	Li et al. 2019	32350851	Tumor
1	2043347				WGS	Li et al. 2019	32350851	Tumor
1	67675711	602			WGS	Li et al. 2019	32350851	Tumor
1	121485245	1433			WGS	Li et al. 2019	32350851	Tumor
1	121485152	1512			WGS	Li et al. 2019	32350851	Tumor
1	27382118	1646			WGS	Li et al. 2019	32350851	Tumor
1	249239195	1646			WGS	Li et al. 2019	32350851	Tumor
1	50330984	1739			WGS	Li et al. 2019	32350851	Tumor
1	121484066	1914			WGS	Li et al. 2019	32350851	Tumor
1	27389289	1951			WGS	Li et al. 2019	32350851	Tumor
1	207996150	2698			WGS	Li et al. 2019	32350851	Tumor
1	20433377	2899			WGS	Li et al. 2019	32350851	Tumor
1	121485174	2881			WGS	Li et al. 2019	32350851	Tumor
1	40435609	2977			WGS	Li et al. 2019	32350851	Tumor
1	67675595	3002			WGS	Li et al. 2019	32350851	Tumor
2	182177901	1160	*AC104820.2*	*intron*	WGS	Li et al. 2019	32350851	Tumor
2	168890582	1293	STK39	intron	WGS	Li et al. 2019	32350851	Tumor
2	216499657	786	*LINC00607*	intron	WGS	Li et al. 2019	32350851	Tumor
2	155885023	576	NA	intergenic	WGS	Li et al. 2019	32350851	Tumor
2	117332228	1887	NA	intergenic	WGS	Li et al. 2019	32350851	Tumor
2	89872076	1988	NA	intergenic	WGS	Li et al. 2019	32350851	Tumor
2	223120652	2572	*PAX3*	intron	WGS	Li et al. 2019	32350851	Tumor
2	230692544	1825	*TRIP12*	*intron*	WGS	Li et al. 2019	32350851	Tumor
2	44104986				WGS	Li et al. 2019	32350851	Tumor
2	44105036				WGS	Li et al. 2019	32350851	Tumor
2	13301723				WGS	Li et al. 2019	32350851	Tumor
2	18099795	391			WGS	Li et al. 2019	32350851	Tumor
2	133036670	1825			WGS	Li et al. 2019	32350851	Tumor
2	80278606	1803			WGS	Li et al. 2019	32350851	Tumor
2	182826285	1978			WGS	Li et al. 2019	32350851	Tumor
2	230692661	2092			WGS	Li et al. 2019	32350851	Tumor
2	147479237	2165			WGS	Li et al. 2019	32350851	Tumor
2	133026214	2215			WGS	Li et al. 2019	32350851	Tumor
2	100247186	2747			WGS	Li et al. 2019	32350851	Tumor
2	147479098	2886			WGS	Li et al. 2019	32350851	Tumor
3	54760922	2958	*CACNA2D3*	intron	WGS	Li et al. 2019	32350851	Tumor
3	12961899	1581	CCNA2	*intron*	WGS	Li et al. 2019	32350851	Tumor
3	32943638	916	NA	intergenic	WGS	Li et al. 2019	32350851	Tumor
3	180293326	1206	NA	intergenic	WGS	Li et al. 2019	32350851	Tumor
3	164949864	1761	LINC01322	intron	WGS	Li et al. 2019	32350851	Tumor
3	170702075		NA	intergenic	WGS	Li et al. 2019	32350851	Tumor
3	170759596		NA	intergenic	WGS	Li et al. 2019	32350851	Tumor
3	80940378	331	NA	intergenic	WGS	Li et al. 2019	32350851	Tumor
3	161101161	574	NA	intergenic	WGS	Li et al. 2019	32350851	Tumor
3	32957535	1836	NA	intergenic	WGS	Li et al. 2019	32350851	Tumor
3	67302718	1915	NA	intergenic	WGS	Li et al. 2019	32350851	Tumor
3	13832541	2183	NA	intergenic	WGS	Li et al. 2019	32350851	Tumor
3	72613950	2494	NA	intergenic	WGS	Li et al. 2019	32350851	Tumor
3	46755960	2100	*PRSS50*	intron	WGS	Li et al. 2019	32350851	Tumor
3	57897477	1928	*SLMAP*	intron	WGS	Li et al. 2019	32350851	Tumor
3	136095899	1918	STAG1	intron	WGS	Li et al. 2019	32350851	Tumor
3	63414906	1493	SYNPR	intron	WGS	Li et al. 2019	32350851	Tumor
3	86998358	2605	VGLL3	intron	WGS	Li et al. 2019	32350851	Tumor
3	46755825	211			WGS	Li et al. 2019	32350851	Tumor
3	136116904	1130			WGS	Li et al. 2019	32350851	Tumor
3	164950050	1703			WGS	Li et al. 2019	32350851	Tumor
3	37934714	1825			WGS	Li et al. 2019	32350851	Tumor
3	13832399	1826			WGS	Li et al. 2019	32350851	Tumor
3	80259073	1932			WGS	Li et al. 2019	32350851	Tumor
3	111653263	2032			WGS	Li et al. 2019	32350851	Tumor
3	37934556	2198			WGS	Li et al. 2019	32350851	Tumor
3	80256102	2612			WGS	Li et al. 2019	32350851	Tumor
3	111653157	2779			WGS	Li et al. 2019	32350851	Tumor
3	57896486	2965			WGS	Li et al. 2019	32350851	Tumor
4	122752481	703	*BBS7*	intron	WGS	Li et al. 2019	32350851	Tumor
4	122757725	3188	*BBS7*	intron	WGS	Li et al. 2019	32350851	Tumor
4	122742824	1812	CCNA2	intron	WGS	Li et al. 2019	32350851	Tumor
4	122744611	1824	*CCNA2*	intron	WGS	Li et al. 2019	32350851	Tumor
4	122745690	1214	IQSEC1	intron	WGS	Li et al. 2019	32350851	Tumor
4	49112770		NA	intergenic	WGS	Li et al. 2019	32350851	Tumor
4	49140157		NA	intergenic	WGS	Li et al. 2019	32350851	Tumor
4	49128598	218	NA	intergenic	WGS	Li et al. 2019	32350851	Tumor
4	58087958	1701	NA	intergenic	WGS	Li et al. 2019	32350851	Tumor
4	58808766	1685	NA	intergenic	WGS	Li et al. 2019	32350851	Tumor
4	49105924	1777	NA	intergenic	WGS	Li et al. 2019	32350851	Tumor
4	153509605	1823	NA	intergenic	WGS	Li et al. 2019	32350851	Tumor
4	64534520	2544	NA	intergenic	WGS	Li et al. 2019	32350851	Tumor
4	181354871	3000	NA	intergenic	WGS	Li et al. 2019	32350851	Tumor
4	49615843	3083	NA	intergenic	WGS	Li et al. 2019	32350851	Tumor
4	57776936		REST	promoter	WGS	Li et al. 2019	32350851	Tumor
4	58024239		RP11-12A1.1	intron	WGS	Li et al. 2019	32350851	Tumor
4	77372793	790	*SHROOM3*	intron	WGS	Li et al. 2019	32350851	Tumor
4	77613236	2036	*SHROOM3*	*intron*	WGS	Li et al. 2019	32350851	Tumor
4	349125	1948	*ZNF141*	Intron	WGS	Li et al. 2019	32350851	Tumor
4	58030365				WGS	Li et al. 2019	32350851	Tumor
4	122742704				WGS	Li et al. 2019	32350851	Tumor
4	58083241	500			WGS	Li et al. 2019	32350851	Tumor
4	49104824	1384		intergenic	WGS	Li et al. 2019	32350851	Tumor
4	57183174	1718			WGS	Li et al. 2019	32350851	Tumor
4	10777081	1825			WGS	Li et al. 2019	32350851	Tumor

Chromosome	Integration site in host genome	Integration site in virus genome	Gene (distance, bp)	Regions	Methods	Author	PMID	Sample
4	32806427	1918			WGS	Li et al. 2019	32350851	Tumor
4	64534768	1915			WGS	Li et al. 2019	32350851	Tumor
4	122742975	1918			WGS	Li et al. 2019	32350851	Tumor
4	110533994	2007			WGS	Li et al. 2019	32350851	Tumor
4	122757615	2147			WGS	Li et al. 2019	32350851	Tumor
4	57771893	2709			WGS	Li et al. 2019	32350851	Tumor
4	122742862	2770			WGS	Li et al. 2019	32350851	Tumor
4	61065376	2999			WGS	Li et al. 2019	32350851	Tumor
5	165072150	2259	*CTC-535M15.2*	intron	WGS	Li et al. 2019	32350851	Tumor
5	1295050	2070	*CTDSPL*	*intron*	WGS	Li et al. 2019	32350851	Tumor
5	11683411		CTNND2	intron	WGS	Li et al. 2019	32350851	Tumor
5	709736	1182	NA	intergenic	WGS	Li et al. 2019	32350851	Tumor
5	10478	3188	NA	intergenic	WGS	Li et al. 2019	32350851	Tumor
5	1057845	1674	SLC12A7	intron	WGS	Li et al. 2019	32350851	Tumor
5	538388	1768	*MIR4456*	promoter	WGS	Li et al. 2019	32350851	Tumor
5	172831836		NA	intergenic	WGS	Li et al. 2019	32350851	Tumor
5	27434883	558	NA	intergenic	WGS	Li et al. 2019	32350851	Tumor
5	81768360	1049	NA	intergenic	WGS	Li et al. 2019	32350851	Tumor
5	3968806	1827	NA	intergenic	WGS	Li et al. 2019	32350851	Tumor
5	86214426	1914	NA	intergenic	WGS	Li et al. 2019	32350851	Tumor
5	71146771	2141	NA	intergenic	WGS	Li et al. 2019	32350851	Tumor
5	8223492	2213	NA	intergenic	WGS	Li et al. 2019	32350851	Tumor
5	70964752	2999	NA	intergenic	WGS	Li et al. 2019	32350851	Tumor
5	1295554	1802	RP11-6N13.1	intron	WGS	Li et al. 2019	32350851	Tumor
5	175721194	3010	*SIMC1*	intron	WGS	Li et al. 2019	32350851	Tumor
5	1295295				WGS	Li et al. 2019	32350851	Tumor
5	1293219				WGS	Li et al. 2019	32350851	Tumor
5	1301243				WGS	Li et al. 2019	32350851	Tumor
5	1295201	299			WGS	Li et al. 2019	32350851	Tumor
5	1295433	514			WGS	Li et al. 2019	32350851	Tumor
5	1295358	625			WGS	Li et al. 2019	32350851	Tumor
5	1304265	662			WGS	Li et al. 2019	32350851	Tumor
5	27432713	653			WGS	Li et al. 2019	32350851	Tumor
5	1296144	799			WGS	Li et al. 2019	32350851	Tumor
5	1296242	1153			WGS	Li et al. 2019	32350851	Tumor
5	1295583	1713			WGS	Li et al. 2019	32350851	Tumor
5	1295309	1804			WGS	Li et al. 2019	32350851	Tumor
5	1276248	1792			WGS	Li et al. 2019	32350851	Tumor
5	1295270	1801			WGS	Li et al. 2019	32350851	Tumor
5	11689	1797			WGS	Li et al. 2019	32350851	Tumor
5	1295050	1826			WGS	Li et al. 2019	32350851	Tumor
5	1302095	1826			WGS	Li et al. 2019	32350851	Tumor
5	104045584	1808			WGS	Li et al. 2019	32350851	Tumor
5	1295367	1818			WGS	Li et al. 2019	32350851	Tumor
5	1295109	1813			WGS	Li et al. 2019	32350851	Tumor
5	1295081	1800			WGS	Li et al. 2019	32350851	Tumor
5	71074985	1908		NA	WGS	Li et al. 2019	32350851	Tumor
5	1295199	1913			WGS	Li et al. 2019	32350851	Tumor
5	1295265	1919			WGS	Li et al. 2019	32350851	Tumor
5	1295687	1912			WGS	Li et al. 2019	32350851	Tumor
5	1295060	1939			WGS	Li et al. 2019	32350851	Tumor
5	104045697	1987			WGS	Li et al. 2019	32350851	Tumor
5	1297416	2057			WGS	Li et al. 2019	32350851	Tumor
5	1295176	2179			WGS	Li et al. 2019	32350851	Tumor
5	1295047	2417			WGS	Li et al. 2019	32350851	Tumor
5	1295188	2497			WGS	Li et al. 2019	32350851	Tumor
5	1296005	2542			WGS	Li et al. 2019	32350851	Tumor
5	1292437	3018			WGS	Li et al. 2019	32350851	Tumor
5	1295060	1849	TERT	Promoter	WGS	Li et al. 2019	32350851	Tumor
5	1295201	213	TERT	Promoter	WGS	Li et al. 2019	32350851	Tumor
5	1296218	1697	TERT	Promoter	WGS	Li et al. 2019	32350851	Tumor
5	1296855	1708	TERT	Promoter	WGS	Li et al. 2019	32350851	Tumor
5	1297577	1171	TERT	Promoter	WGS	Li et al. 2019	32350851	Tumor
5	1295270	1703	TERT	Promoter	WGS	Li et al. 2019	32350851	Tumor
5	1295367	1721	TERT	Promoter	WGS	Li et al. 2019	32350851	Tumor
5	1295882	159	TERT	Promoter	WGS	Li et al. 2019	32350851	Tumor
5	1295074	1730	TERT	Promoter	WGS	Li et al. 2019	32350851	Tumor
5	1295295	2416	TERT	Promoter	WGS	Li et al. 2019	32350851	Tumor
5	1295102	3123	TERT	Promoter	WGS	Li et al. 2019	32350851	Tumor
5	1295180	1828	TERT	Promoter	WGS	Li et al. 2019	32350851	Tumor
5	1293219	1078	TERT	Promoter	WGS	Li et al. 2019	32350851	Tumor
5	1293348	3154	TERT	Promoter	WGS	Li et al. 2019	32350851	Tumor
5	1303322	1842	TERT	Promoter	WGS	Li et al. 2019	32350851	Tumor
5	1295109	1722	TERT	Promoter	WGS	Li et al. 2019	32350851	Tumor
5	1295265	1828	TERT	Promoter	WGS	Li et al. 2019	32350851	Tumor
5	1295554	1714	TERT	Promoter	WGS	Li et al. 2019	32350851	Tumor
5	1295687	1828	TERT	Promoter	WGS	Li et al. 2019	32350851	Tumor
5	1301243	2705	TERT	Promoter	WGS	Li et al. 2019	32350851	Tumor
5	1302095	1714	TERT	Promoter	WGS	Li et al. 2019	32350851	Tumor
5	1295882	2981	TERT	Promoter	WGS	Li et al. 2019	32350851	Tumor
5	1296005	2435	TERT	Promoter	WGS	Li et al. 2019	32350851	Tumor
5	1295176	2063	TERT	Promoter	WGS	Li et al. 2019	32350851	Tumor
5	1295050	1957	TERT	Promoter	WGS	Li et al. 2019	32350851	Tumor
5	1295309	1690	TERT	Promoter	WGS	Li et al. 2019	32350851	Tumor
5	1295433	400	TERT	Promoter	WGS	Li et al. 2019	32350851	Tumor
5	1292437	2904	TERT	Promoter	WGS	Li et al. 2019	32350851	Tumor
5	1292327	2641	TERT	Promoter	WGS	Li et al. 2019	32350851	Tumor
5	1304265	547	TERT	Promoter	WGS	Li et al. 2019	32350851	Tumor
5	1295583	1599	TERT	Promoter	WGS	Li et al. 2019	32350851	Tumor
5	1297416	1965	TERT	Promoter	WGS	Li et al. 2019	32350851	Tumor
5	1295612	1735	TERT	Promoter	WGS	Li et al. 2019	32350851	Tumor
5	1296144	709	TERT	Promoter	WGS	Li et al. 2019	32350851	Tumor
5	1296242	1076	TERT	Promoter	WGS	Li et al. 2019	32350851	Tumor
5	1295333	1713	TERT	Promoter	WGS	Li et al. 2019	32350851	Tumor

Chromosome	Integration site in host genome	Integration site in virus genome	Gene (distance, bp)	Regions	Methods	Author	PMID	Sample
5	1295459	1826	TERT	Promoter	WGS	Li et al. 2019	32350851	Tumor
5	1289756	1818	TERT	intron	WGS	Li et al. 2019	32350851	Tumor
5	1292536	3112	TERT	intron	WGS	Li et al. 2019	32350851	Tumor
5	1276248	1702	TERT	intron	WGS	Li et al. 2019	32350851	Tumor
5	1276320	2615	TERT	intron	WGS	Li et al. 2019	32350851	Tumor
5	1295081	1756	TERT	exon	WGS	Li et al. 2019	32350851	Tumor
5	1295039	2241	TERT	exon	WGS	Li et al. 2019	32350851	Tumor
5	1295047	2303	TERT	exon	WGS	Li et al. 2019	32350851	Tumor
5	1295188	2381	TERT	exon	WGS	Li et al. 2019	32350851	Tumor
5	1295050	1713	TERT	exon	WGS	Li et al. 2019	32350851	Tumor
5	1295199	1819	TERT	exon	WGS	Li et al. 2019	32350851	Tumor
5	1295035	1710	TERT	exon	WGS	Li et al. 2019	32350851	Tumor
5	1295358	512	TERT	exon	WGS	Li et al. 2019	32350851	Tumor
6	58778406	2826	NA	intergenic	WGS	Li et al. 2019	32350851	Tumor
6	16920350		NA	intergenic	WGS	Li et al. 2019	32350851	Tumor
6	143294083		RP11-439L18.3	intron	WGS	Li et al. 2019	32350851	Tumor
6	139027319	1725			WGS	Li et al. 2019	32350851	Tumor
6	139027468	2749			WGS	Li et al. 2019	32350851	Tumor
6	107657496	2888			WGS	Li et al. 2019	32350851	Tumor
7	90318864	1939	*CDK14*	*intron*	WGS	Li et al. 2019	32350851	Tumor
7	45668032	1806	ADCY1	intron	WGS	Li et al. 2019	32350851	Tumor
7	139163776	2945	*KLRG2*	*intron*	WGS	Li et al. 2019	32350851	Tumor
7	44080861	1248	*LINC00957*	intron	WGS	Li et al. 2019	32350851	Tumor
7	152115452	1982	*MLL3*	intron	WGS	Li et al. 2019	32350851	Tumor
7	152182157		NA	intergenic	WGS	Li et al. 2019	32350851	Tumor
7	108512812	944	NA	intergenic	WGS	Li et al. 2019	32350851	Tumor
7	121925348	1275	NA	intergenic	WGS	Li et al. 2019	32350851	Tumor
7	13122438	1822	NA	intergenic	WGS	Li et al. 2019	32350851	Tumor
7	108513085	1926	NA	intergenic	WGS	Li et al. 2019	32350851	Tumor
7	45855545	1910	NA	intergenic	WGS	Li et al. 2019	32350851	Tumor
7	44132757	1910	NA	intergenic	WGS	Li et al. 2019	32350851	Tumor
7	38279314	1732	*TRGC2*	intron	WGS	Li et al. 2019	32350851	Tumor
7	129487915		UBE2H	intron	WGS	Li et al. 2019	32350851	Tumor
7	129690292		ZC3HC1	exon	WGS	Li et al. 2019	32350851	Tumor
7	8393048	377			WGS	Li et al. 2019	32350851	Tumor
7	70233375	904			WGS	Li et al. 2019	32350851	Tumor
7	121925348	1275			WGS	Li et al. 2019	32350851	Tumor
7	152115372	1443			WGS	Li et al. 2019	32350851	Tumor
7	90318961	1633			WGS	Li et al. 2019	32350851	Tumor
7	14131064	1773			WGS	Li et al. 2019	32350851	Tumor
7	64001596	1937			WGS	Li et al. 2019	32350851	Tumor
7	70268168	2110			WGS	Li et al. 2019	32350851	Tumor
8	141543720	1319	*AGO2*	intron	WGS	Li et al. 2019	32350851	Tumor
8	135130538	2069	*CALCOCO2*	*intron*	WGS	Li et al. 2019	32350851	Tumor
8	94115512	1718	CTNNA2	intron	WGS	Li et al. 2019	32350851	Tumor
8	94729084	2485	*FAM92A1*	intron	WGS	Li et al. 2019	32350851	Tumor
8	38269180	944	*FGFR1*	intron	WGS	Li et al. 2019	32350851	Tumor
8	46843668	1507	NA	intergenic	WGS	Li et al. 2019	32350851	Tumor
8	43831540	2337	NA	intergenic	WGS	Li et al. 2019	32350851	Tumor
8	36491778		NA	intergenic	WGS	Li et al. 2019	32350851	Tumor
8	36052210		NA	intergenic	WGS	Li et al. 2019	32350851	Tumor
8	40306702	159	NA	intergenic	WGS	Li et al. 2019	32350851	Tumor
8	57756143	190	NA	intergenic	WGS	Li et al. 2019	32350851	Tumor
8	55721980	281	NA	intergenic	WGS	Li et al. 2019	32350851	Tumor
8	41264358	508	NA	intergenic	WGS	Li et al. 2019	32350851	Tumor
8	37068433	2770	NA	intergenic	WGS	Li et al. 2019	32350851	Tumor
8	111470459	828	NA	intergenic	WGS	Li et al. 2019	32350851	Tumor
8	122999	1400	NA	intergenic	WGS	Li et al. 2019	32350851	Tumor
8	43827051	1910	NA	intergenic	WGS	Li et al. 2019	32350851	Tumor
8	111700494	1811	NA	intergenic	WGS	Li et al. 2019	32350851	Tumor
8	43094783	1900	NA	intergenic	WGS	Li et al. 2019	32350851	Tumor
8	43821030	2417	NA	intergenic	WGS	Li et al. 2019	32350851	Tumor
8	84907675	1888	NA	intergenic	WGS	Li et al. 2019	32350851	Tumor
8	94115323	2327	PHLDB2	intron	WGS	Li et al. 2019	32350851	Tumor
8	57092724		PLAG1	intron	WGS	Li et al. 2019	32350851	Tumor
8	22340300	1822	*PPP3CC*	*intron*	WGS	Li et al. 2019	32350851	Tumor
8	87092485	1462	PSKH2	intron	WGS	Li et al. 2019	32350851	Tumor
8	64986083	1933	RP11-32K4.1	intron	WGS	Li et al. 2019	32350851	Tumor
8	145089377		SPATC1	promoter	WGS	Li et al. 2019	32350851	Tumor
8	135130773	1795	*TJP2*	*exon*	WGS	Li et al. 2019	32350851	Tumor
8	59886547		TOX	intron	WGS	Li et al. 2019	32350851	Tumor
8	35111281	1932	*UNC5D*	intron	WGS	Li et al. 2019	32350851	Tumor
8	40722785	575	*ZMAT4*	intron	WGS	Li et al. 2019	32350851	Tumor
8	43092875	1885			WGS	Li et al. 2019	32350851	Tumor
8	22350344	1909			WGS	Li et al. 2019	32350851	Tumor
8	36838914	2369			WGS	Li et al. 2019	32350851	Tumor
9	38585072	3119	*ANKRD18A*	intron	WGS	Li et al. 2019	32350851	Tumor
9	99921871	1911	*ANKRD18CP*	intron	WGS	Li et al. 2019	32350851	Tumor
9	97560257		C9orf3	intron	WGS	Li et al. 2019	32350851	Tumor
9	97656709	207	*C9orf3*	intron	WGS	Li et al. 2019	32350851	Tumor
9	21993692	1273	*CDKN2A*	intron	WGS	Li et al. 2019	32350851	Tumor
9	124439455	3150	*DAB2IP*	intron	WGS	Li et al. 2019	32350851	Tumor
9	141114326	261	*FAM157B*	intron	WGS	Li et al. 2019	32350851	Tumor
9	7008542	3161	KDM4C	intron	WGS	Li et al. 2019	32350851	Tumor
9	66833101		NA	intergenic	WGS	Li et al. 2019	32350851	Tumor
9	132141356		NA	intergenic	WGS	Li et al. 2019	32350851	Tumor
9	44331538	841	NA	intergenic	WGS	Li et al. 2019	32350851	Tumor
9	12085884	1178	NA	intergenic	WGS	Li et al. 2019	32350851	Tumor
9	25037310	1294	NA	intergenic	WGS	Li et al. 2019	32350851	Tumor
9	16393992	1450	NA	intergenic	WGS	Li et al. 2019	32350851	Tumor
9	42223657	1772	NA	intergenic	WGS	Li et al. 2019	32350851	Tumor
9	34855114	1934	NA	intergenic	WGS	Li et al. 2019	32350851	Tumor
9	71863018	2870	NA	intergenic	WGS	Li et al. 2019	32350851	Tumor
9	36064434	3032	RECK	intron	WGS	Li et al. 2019	32350851	Tumor

Chromosome	Integration site in host genome	Integration site in virus genome	Gene (distance, bp)	Regions	Methods	Author	PMID	Sample
9	116740689		ZNF618	intron	WGS	Li et al. 2019	32350851	Tumor
9	33572432	627		intron	WGS	Li et al. 2019	32350851	Tumor
9	32531477	1228			WGS	Li et al. 2019	32350851	Tumor
9	71862896	1821			WGS	Li et al. 2019	32350851	Tumor
9	119332721	1821		intron	WGS	Li et al. 2019	32350851	Tumor
9	119259605	1909			WGS	Li et al. 2019	32350851	Tumor
9	66820240	3017			WGS	Li et al. 2019	32350851	Tumor
10	114101936	2326	GUCY2GP	intron	WGS	Li et al. 2019	32350851	Tumor
10	10992138	737	LINC00710	intron	WGS	Li et al. 2019	32350851	Tumor
10	91111521	2116	LIPA	*intron*	WGS	Li et al. 2019	32350851	Tumor
10	61786242	582	NA	intergenic	WGS	Li et al. 2019	32350851	Tumor
10	42534522	713	NA	intergenic	WGS	Li et al. 2019	32350851	Tumor
10	42385524	1057	NA	intergenic	WGS	Li et al. 2019	32350851	Tumor
10	42385980	1195	NA	intergenic	WGS	Li et al. 2019	32350851	Tumor
10	42599978	1195	NA	intergenic	WGS	Li et al. 2019	32350851	Tumor
10	42387000	1910	NA	intergenic	WGS	Li et al. 2019	32350851	Tumor
10	42385987	2166	NA	intergenic	WGS	Li et al. 2019	32350851	Tumor
10	30212820	2385	NA	intergenic	WGS	Li et al. 2019	32350851	Tumor
10	42393049	3006	NA	intergenic	WGS	Li et al. 2019	32350851	Tumor
10	81521897	2176	*RP11-119F19.2*	*intron*	WGS	Li et al. 2019	32350851	Tumor
10	109784433	1827	RP11-215N21.1	*intron*	WGS	Li et al. 2019	32350851	Tumor
10	42360529	1689			WGS	Li et al. 2019	32350851	Tumor
10	42385198	1714			WGS	Li et al. 2019	32350851	Tumor
10	135524654	1769		intergenic	WGS	Li et al. 2019	32350851	Tumor
10	103536382	1826			WGS	Li et al. 2019	32350851	Tumor
10	108180352	1827			WGS	Li et al. 2019	32350851	Tumor
10	16260314	1866			WGS	Li et al. 2019	32350851	Tumor
10	103536506	1961			WGS	Li et al. 2019	32350851	Tumor
10	81521876	2230			WGS	Li et al. 2019	32350851	Tumor
10	135524661	2554			WGS	Li et al. 2019	32350851	Tumor
10	114041512	2735			WGS	Li et al. 2019	32350851	Tumor
11	70000541	3188	*ANO1*	Intron	WGS	Li et al. 2019	32350851	Tumor
11	188554	563	*BET1L*	intron	WGS	Li et al. 2019	32350851	Tumor
11	175461	3191	BET1L	intron	WGS	Li et al. 2019	32350851	Tumor
11	122788740		C11orf63	intron	WGS	Li et al. 2019	32350851	Tumor
11	105762235		GRIA4	intron	WGS	Li et al. 2019	32350851	Tumor
11	29207796	1579	*KMT2B*	*intron*	WGS	Li et al. 2019	32350851	Tumor
11	81285243		NA	intergenic	WGS	Li et al. 2019	32350851	Tumor
11	68982963	1975	NA	intergenic	WGS	Li et al. 2019	32350851	Tumor
11	76306304	2775	NA	intergenic	WGS	Li et al. 2019	32350851	Tumor
11	74113839		PGM2L1	promoter	WGS	Li et al. 2019	32350851	Tumor
11	51568509	387			WGS	Li et al. 2019	32350851	Tumor
11	102024641	1810			WGS	Li et al. 2019	32350851	Tumor
11	29207683	1910			WGS	Li et al. 2019	32350851	Tumor
11	76306181	1970			WGS	Li et al. 2019	32350851	Tumor
12	28716771		CCDC91	intron	WGS	Li et al. 2019	32350851	Tumor
12	29326687	1684	*FAR2*	intron	WGS	Li et al. 2019	32350851	Tumor
12	57856358	2283	*GLI1*	promoter	WGS	Li et al. 2019	32350851	Tumor
12	231668		IQSEC3	intron	WGS	Li et al. 2019	32350851	Tumor
12	128338638		NA	intergenic	WGS	Li et al. 2019	32350851	Tumor
12	69706614		NA	intergenic	WGS	Li et al. 2019	32350851	Tumor
12	30573669	2231	NA	intergenic	WGS	Li et al. 2019	32350851	Tumor
12	24431417	234	*RP11-444D3.1*	intron	WGS	Li et al. 2019	32350851	Tumor
12	113830885	1824	*SDS*	exon	WGS	Li et al. 2019	32350851	Tumor
12	12634785				WGS	Li et al. 2019	32350851	Tumor
12	12634922				WGS	Li et al. 2019	32350851	Tumor
12	24431316	1384			WGS	Li et al. 2019	32350851	Tumor
12	34569759	1023			WGS	Li et al. 2019	32350851	Tumor
12	5381317	1046			WGS	Li et al. 2019	32350851	Tumor
12	127650866	1071			WGS	Li et al. 2019	32350851	Tumor
12	57856179	1275			WGS	Li et al. 2019	32350851	Tumor
12	820335	1785			WGS	Li et al. 2019	32350851	Tumor
12	5403676	1803			WGS	Li et al. 2019	32350851	Tumor
12	5402781	1941			WGS	Li et al. 2019	32350851	Tumor
13	41137287	2942	FOXO1	intron	WGS	Li et al. 2019	32350851	Tumor
13	68656614		NA	intergenic	WGS	Li et al. 2019	32350851	Tumor
13	85616297		NA	intergenic	WGS	Li et al. 2019	32350851	Tumor
13	54500268		NA	intergenic	WGS	Li et al. 2019	32350851	Tumor
13	87912097		NA	intergenic	WGS	Li et al. 2019	32350851	Tumor
13	88959641		NA	intergenic	WGS	Li et al. 2019	32350851	Tumor
13	63341487	252	NA	intergenic	WGS	Li et al. 2019	32350851	Tumor
13	112814718	620	NA	intergenic	WGS	Li et al. 2019	32350851	Tumor
13	61538340	1824	NA	intergenic	WGS	Li et al. 2019	32350851	Tumor
13	103983816	1933	NA	intergenic	WGS	Li et al. 2019	32350851	Tumor
13	54873791	2053	NA	intergenic	WGS	Li et al. 2019	32350851	Tumor
13	66106535	2809	NA	intergenic	WGS	Li et al. 2019	32350851	Tumor
13	84038710	3188	NA	intergenic	WGS	Li et al. 2019	32350851	Tumor
13	85640386	1815	*RP11-531P20.1*	intron	WGS	Li et al. 2019	32350851	Tumor
13	54873691	313			WGS	Li et al. 2019	32350851	Tumor
13	74010423	1250		intergenic	WGS	Li et al. 2019	32350851	Tumor
13	104255564	1730			WGS	Li et al. 2019	32350851	Tumor
13	34655043	1806			WGS	Li et al. 2019	32350851	Tumor
13	34654944	1842			WGS	Li et al. 2019	32350851	Tumor
13	104255463	1917			WGS	Li et al. 2019	32350851	Tumor
13	41137258	1967			WGS	Li et al. 2019	32350851	Tumor
14	66018814	2367	FUT8	intron	WGS	Li et al. 2019	32350851	Tumor
14	35632301		KIAA0391	intron	WGS	Li et al. 2019	32350851	Tumor
14	85848849	801	*LINC02329*	intron	WGS	Li et al. 2019	32350851	Tumor
14	106159965		NA	intergenic	WGS	Li et al. 2019	32350851	Tumor
14	47132428		NA	intergenic	WGS	Li et al. 2019	32350851	Tumor
14	52591733	2970	NA	intergenic	WGS	Li et al. 2019	32350851	Tumor
14	67900387	124			WGS	Li et al. 2019	32350851	Tumor
14	67900571	691			WGS	Li et al. 2019	32350851	Tumor
14	43221919	883			WGS	Li et al. 2019	32350851	Tumor

Chromosome	Integration site in host genome	Integration site in virus genome	Gene (distance, bp)	Regions	Methods	Author	PMID	Sample
14	43221813	2089			WGS	Li et al. 2019	32350851	Tumor
15	29222125		APBA2	intron	WGS	Li et al. 2019	32350851	Tumor
15	81732100	2710	*CTD-2034I4.1*	*intron*	WGS	Li et al. 2019	32350851	Tumor
15	97235293	701	NA	intergenic	WGS	Li et al. 2019	32350851	Tumor
15	55166634		NA	intergenic	WGS	Li et al. 2019	32350851	Tumor
15	88526608	1736	*NTRK3*	intron	WGS	Li et al. 2019	32350851	Tumor
15	36336876		RP11-184D12.1	intron	WGS	Li et al. 2019	32350851	Tumor
15	27443402	1931			WGS	Li et al. 2019	32350851	Tumor
15	81731997	2596			WGS	Li et al. 2019	32350851	Tumor
16	46388321	751	NA	intergenic	WGS	Li et al. 2019	32350851	Tumor
16	30351918	1685	NA	intergenic	WGS	Li et al. 2019	32350851	Tumor
16	72220524		NA	intergenic	WGS	Li et al. 2019	32350851	Tumor
16	46396633		NA	intergenic	WGS	Li et al. 2019	32350851	Tumor
16	8052784		NA	intergenic	WGS	Li et al. 2019	32350851	Tumor
16	46414400		NA	intergenic	WGS	Li et al. 2019	32350851	Tumor
16	46403659	732	NA	intergenic	WGS	Li et al. 2019	32350851	Tumor
16	10374834	348	NA	intergenic	WGS	Li et al. 2019	32350851	Tumor
16	46394462	532	NA	intergenic	WGS	Li et al. 2019	32350851	Tumor
16	25578079	1274	NA	intergenic	WGS	Li et al. 2019	32350851	Tumor
16	62879334	1818	NA	intergenic	WGS	Li et al. 2019	32350851	Tumor
16	46393435	1811	NA	intergenic	WGS	Li et al. 2019	32350851	Tumor
16	46403018	1819	NA	intergenic	WGS	Li et al. 2019	32350851	Tumor
16	46386535	1921	NA	intergenic	WGS	Li et al. 2019	32350851	Tumor
16	85976716	2761	NA	intergenic	WGS	Li et al. 2019	32350851	Tumor
16	46407343	2964	NA	intergenic	WGS	Li et al. 2019	32350851	Tumor
16	46390640	3030	NA	intergenic	WGS	Li et al. 2019	32350851	Tumor
16	90139803	1818	*PRDM7*	intron	WGS	Li et al. 2019	32350851	Tumor
16	46386632				WGS	Li et al. 2019	32350851	Tumor
16	10367584				WGS	Li et al. 2019	32350851	Tumor
16	85976600				WGS	Li et al. 2019	32350851	Tumor
16	62879485	1789			WGS	Li et al. 2019	32350851	Tumor
16	62879415	1818			WGS	Li et al. 2019	32350851	Tumor
16	46388200	2971			WGS	Li et al. 2019	32350851	Tumor
17	19811184	854	*AKAP10*	intron	WGS	Li et al. 2019	32350851	Tumor
17	11674103	1229	*DNAH9*	intron	WGS	Li et al. 2019	32350851	Tumor
17	17987605	2153	DRG2	promoter	WGS	Li et al. 2019	32350851	Tumor
17	80045713		FASN	exon	WGS	Li et al. 2019	32350851	Tumor
17	42501744	1735	GPATCH8	exon	WGS	Li et al. 2019	32350851	Tumor
17	22245498	809	NA	intergenic	WGS	Li et al. 2019	32350851	Tumor
17	63521190		NA	intergenic	WGS	Li et al. 2019	32350851	Tumor
17	20721368		NA	intergenic	WGS	Li et al. 2019	32350851	Tumor
17	12183934	1313	NA	intergenic	WGS	Li et al. 2019	32350851	Tumor
17	12216447	1350	NA	intergenic	WGS	Li et al. 2019	32350851	Tumor
17	19004769	1785	NA	intergenic	WGS	Li et al. 2019	32350851	Tumor
17	22249926	1801	NA	intergenic	WGS	Li et al. 2019	32350851	Tumor
17	12443245	2933	NA	intergenic	WGS	Li et al. 2019	32350851	Tumor
17	12260903	911	NA	intergenic	WGS	Li et al. 2019	32350851	Tumor
17	44498179		NSFP1	intron	WGS	Li et al. 2019	32350851	Tumor
17	8794231	1466	*PIK3R5*	intron	WGS	Li et al. 2019	32350851	Tumor
17	11383781	2433	*SHISA6*	intron	WGS	Li et al. 2019	32350851	Tumor
17	19595291	3089	SLC47A2	intron	WGS	Li et al. 2019	32350851	Tumor
17	9319934		STX8	intron	WGS	Li et al. 2019	32350851	Tumor
17	9319420				WGS	Li et al. 2019	32350851	Tumor
17	22251311	426			WGS	Li et al. 2019	32350851	Tumor
17	12256276	442			WGS	Li et al. 2019	32350851	Tumor
17	19811173	868			WGS	Li et al. 2019	32350851	Tumor
17	11383296	1751			WGS	Li et al. 2019	32350851	Tumor
17	12183728	1775			WGS	Li et al. 2019	32350851	Tumor
17	21794211	1785			WGS	Li et al. 2019	32350851	Tumor
17	46912043	1815			WGS	Li et al. 2019	32350851	Tumor
17	46912227	1947			WGS	Li et al. 2019	32350851	Tumor
17	42501633	2389			WGS	Li et al. 2019	32350851	Tumor
17	19004656	2790			WGS	Li et al. 2019	32350851	Tumor
18	18518727		NA	intergenic	WGS	Li et al. 2019	32350851	Tumor
18	535047	1826	NA	intergenic	WGS	Li et al. 2019	32350851	Tumor
18	1861366	2852	NA	intergenic	WGS	Li et al. 2019	32350851	Tumor
18	78001924	2738	*PARD6G*		WGS	Li et al. 2019	32350851	Tumor
19	36212759	1627	ASTN2	intron	WGS	Li et al. 2019	32350851	Tumor
19	30301164	1925	*CCNE1*	*promoter*	WGS	Li et al. 2019	32350851	Tumor
19	30298336	2003	*CCNE1*	*promoter*	WGS	Li et al. 2019	32350851	Tumor
19	16284690		CIB3	promoter	WGS	Li et al. 2019	32350851	Tumor
19	59116312	326	NA	intergenic	WGS	Li et al. 2019	32350851	Tumor
19	30303002	1663	CCNE1	intron	WGS	Li et al. 2019	32350851	Tumor
19	35628104	139	*LGI4*	promoter	WGS	Li et al. 2019	32350851	Tumor
19	36212916		KMT2B	intron	WGS	Li et al. 2019	32350851	Tumor
19	36212775		KMT2B	intron	WGS	Li et al. 2019	32350851	Tumor
19	36212696	1621	*KMT2B*	exon	WGS	Li et al. 2019	32350851	Tumor
19	36213848	1639	*KMT2B*	intron	WGS	Li et al. 2019	32350851	Tumor
19	36212601	1713	*KMT2B*	exon	WGS	Li et al. 2019	32350851	Tumor
19	36212939	1710	*KMT2B*	intron	WGS	Li et al. 2019	32350851	Tumor
19	36212564	1760	*KMT2B*	*exon*	WGS	Li et al. 2019	32350851	Tumor
19	36213805	1795	*KMT2B*	*intron*	WGS	Li et al. 2019	32350851	Tumor
19	36212966	1826	*KMT2B*	intron	WGS	Li et al. 2019	32350851	Tumor
19	36213700	1848	KMT2B	exon	WGS	Li et al. 2019	32350851	Tumor
19	36213171	2417	*KMT2B*	*intron*	WGS	Li et al. 2019	32350851	Tumor
19	45500580	2846	KMT2B	intron	WGS	Li et al. 2019	32350851	Tumor
19	20360936		NA	intergenic	WGS	Li et al. 2019	32350851	Tumor
19	20779739		NA	intergenic	WGS	Li et al. 2019	32350851	Tumor
19	54160010	2680	NA	intergenic	WGS	Li et al. 2019	32350851	Tumor
19	47371162	1702	NA	intergenic	WGS	Li et al. 2019	32350851	Tumor
19	45500475	1725	RELB	promoter	WGS	Li et al. 2019	32350851	Tumor
19	36190500		ZBTB32	promoter	WGS	Li et al. 2019	32350851	Tumor
19	3029240				WGS	Li et al. 2019	32350851	Tumor
19	36213066				WGS	Li et al. 2019	32350851	Tumor

Chromosome	Integration site in host genome	Integration site in virus genome	Gene (distance, bp)	Regions	Methods	Author	PMID	Sample
19	30301076				WGS	Li et al. 2019	32350851	Tumor
19	36212898				WGS	Li et al. 2019	32350851	Tumor
19	36211821	153			WGS	Li et al. 2019	32350851	Tumor
19	30303064	329			WGS	Li et al. 2019	32350851	Tumor
19	36212468	345			WGS	Li et al. 2019	32350851	Tumor
19	58811230	628			WGS	Li et al. 2019	32350851	Tumor
19	47370723	805			WGS	Li et al. 2019	32350851	Tumor
19	27732102	949			WGS	Li et al. 2019	32350851	Tumor
19	36213685	1085			WGS	Li et al. 2019	32350851	Tumor
19	36213423	1572			WGS	Li et al. 2019	32350851	Tumor
19	36212694	1631			WGS	Li et al. 2019	32350851	Tumor
19	35628238	1719			WGS	Li et al. 2019	32350851	Tumor
19	36213550	1827			WGS	Li et al. 2019	32350851	Tumor
19	36213810	1809			WGS	Li et al. 2019	32350851	Tumor
19	30298426	1816			WGS	Li et al. 2019	32350851	Tumor
19	30292509	1873			WGS	Li et al. 2019	32350851	Tumor
19	30303202	2004			WGS	Li et al. 2019	32350851	Tumor
19	36212889	2035			WGS	Li et al. 2019	32350851	Tumor
19	27736532	2368			WGS	Li et al. 2019	32350851	Tumor
19	36213739	2494			WGS	Li et al. 2019	32350851	Tumor
19	36212902	2687			WGS	Li et al. 2019	32350851	Tumor
19	58810806	2832			WGS	Li et al. 2019	32350851	Tumor
19	30303134	2951			WGS	Li et al. 2019	32350851	Tumor
19	36212556	3018			WGS	Li et al. 2019	32350851	Tumor
20	5321872		DOK5	intron	WGS	Li et al. 2019	32350851	Tumor
20	49070987		NA	intergenic	WGS	Li et al. 2019	32350851	Tumor
20	20553076		RALGAPA2	intron	WGS	Li et al. 2019	32350851	Tumor
20	47181763	469			WGS	Li et al. 2019	32350851	Tumor
20	55648237	521			WGS	Li et al. 2019	32350851	Tumor
21	45148209	1119	PDXK	intron	WGS	Li et al. 2019	32350851	Tumor
22	2551418		KIAA167	intron	WGS	Li et al. 2019	32350851	Tumor
22	22627200	1616	NA	intergenic	WGS	Li et al. 2019	32350851	Tumor
22	42878002	2356	NA	intergenic	WGS	Li et al. 2019	32350851	Tumor
737	782 15	13	SNRPN intron		WGS	Li et al. 2019	32350851	Tumor
X	146427592	1487	NA	intergenic	WGS	Li et al. 2019	32350851	Tumor
X	129330421		NA	intergenic	WGS	Li et al. 2019	32350851	Tumor
X	89714251	950	NA	intergenic	WGS	Li et al. 2019	32350851	Tumor
X	115815118	2484	NA	intergenic	WGS	Li et al. 2019	32350851	Tumor
X	22076590	1556	*PHEX*	intron	WGS	Li et al. 2019	32350851	Tumor
X	106350003	1727	*RBM41*	intron	WGS	Li et al. 2019	32350851	Tumor
Y	59363025	2610	NA	intergenic	WGS	Li et al. 2019	32350851	Tumor
chr5	1246962	3121	TERT	downstream	targeted sequencing	Sze et al. 2020	32170761	Tumor
chr5	1269385	822	TERT	intronic	targeted sequencing	Sze et al. 2020	32170761	Tumor
chr5	1269538	1951	TERT	intronic	targeted sequencing	Sze et al. 2020	32170761	Tumor
chr5	1280239	2040	TERT	intronic	targeted sequencing	Sze et al. 2020	32170761	Tumor
chr5	1280384	1909	TERT	exonic	targeted sequencing	Sze et al. 2020	32170761	Tumor
chr5	1295135	1822	TERT	UTR5	targeted sequencing	Sze et al. 2020	32170761	Tumor
chr5	1295169	1822	TERT	upstream	targeted sequencing	Sze et al. 2020	32170761	Tumor
chr5	1295175	1826	TERT	upstream	targeted sequencing	Sze et al. 2020	32170761	Tumor
chr5	1295176	1821	TERT	upstream	targeted sequencing	Sze et al. 2020	32170761	Tumor
chr5	1295196	86	TERT	upstream	targeted sequencing	Sze et al. 2020	32170761	Tumor
chr5	1295266	3215	TERT	upstream	targeted sequencing	Sze et al. 2020	32170761	Tumor
chr5	1295309	1553	TERT	upstream	targeted sequencing	Sze et al. 2020	32170761	Tumor
chr5	1295329	3215	TERT	upstream	targeted sequencing	Sze et al. 2020	32170761	Tumor
chr5	1295345	1802	TERT	upstream	targeted sequencing	Sze et al. 2020	32170761	Tumor
chr5	1295394	734	TERT	upstream	targeted sequencing	Sze et al. 2020	32170761	Tumor
chr5	1295397	1814	TERT	upstream	targeted sequencing	Sze et al. 2020	32170761	Tumor
chr5	1295411	1745	TERT	upstream	targeted sequencing	Sze et al. 2020	32170761	Tumor
chr5	1295469	1779	TERT	upstream	targeted sequencing	Sze et al. 2020	32170761	Tumor
chr5	1295475	1792	TERT	upstream	targeted sequencing	Sze et al. 2020	32170761	Tumor
chr5	1295483	1788	TERT	upstream	targeted sequencing	Sze et al. 2020	32170761	Tumor
chr5	1295500	1843	TERT	upstream	targeted sequencing	Sze et al. 2020	32170761	Tumor
chr5	1295509	1840	TERT	upstream	targeted sequencing	Sze et al. 2020	32170761	Tumor
chr5	1295529	1814	TERT	upstream	targeted sequencing	Sze et al. 2020	32170761	Tumor
chr5	1295562	1363	TERT	upstream	targeted sequencing	Sze et al. 2020	32170761	Tumor
chr5	1295563	1720	TERT	upstream	targeted sequencing	Sze et al. 2020	32170761	Tumor
chr5	1295573	1945	TERT	upstream	targeted sequencing	Sze et al. 2020	32170761	Tumor
chr5	1295599	2976	TERT	upstream	targeted sequencing	Sze et al. 2020	32170761	Tumor
chr5	1295603	1826	TERT	upstream	targeted sequencing	Sze et al. 2020	32170761	Tumor
chr5	1295671	603	TERT	upstream	targeted sequencing	Sze et al. 2020	32170761	Tumor
chr5	1295681	1817	TERT	upstream	targeted sequencing	Sze et al. 2020	32170761	Tumor
chr5	1295712	1774	TERT	upstream	targeted sequencing	Sze et al. 2020	32170761	Tumor
chr5	1295721	1519	TERT	upstream	targeted sequencing	Sze et al. 2020	32170761	Tumor
chr5	1295733	1817	TERT	upstream	targeted sequencing	Sze et al. 2020	32170761	Tumor
chr5	1295746	1113	TERT	upstream	targeted sequencing	Sze et al. 2020	32170761	Tumor
chr5	1295889	1811	TERT	upstream	targeted sequencing	Sze et al. 2020	32170761	Tumor
chr5	1296411	3034	TERT	upstream	targeted sequencing	Sze et al. 2020	32170761	Tumor
chr5	1296460	1061	TERT	upstream	targeted sequencing	Sze et al. 2020	32170761	Tumor
chr5	1297128	1634	TERT	upstream	targeted sequencing	Sze et al. 2020	32170761	Tumor
chr5	1297474	718	TERT	upstream	targeted sequencing	Sze et al. 2020	32170761	Tumor
chr5	1297798	1428	TERT	upstream	targeted sequencing	Sze et al. 2020	32170761	Tumor
chr5	1298772	32	TERT	upstream	targeted sequencing	Sze et al. 2020	32170761	Tumor
chr5	1298814	1826	TERT	upstream	targeted sequencing	Sze et al. 2020	32170761	Tumor
chr5	1299013	1079	TERT	upstream	targeted sequencing	Sze et al. 2020	32170761	Tumor
chr5	1300875	1792	TERT	upstream	targeted sequencing	Sze et al. 2020	32170761	Tumor
chr5	1301193	1138	TERT	upstream	targeted sequencing	Sze et al. 2020	32170761	Tumor
chr5	1305072	1823	TERT	upstream	targeted sequencing	Sze et al. 2020	32170761	Tumor
chr5	NA	NA	TERT	Promoter	targeted sequencing	Jang et al. 2021	34209079	Tumor
chr5	NA	NA	TERT	Promoter	targeted sequencing	Jang et al. 2021	34209079	Tumor
chr5	NA	NA	TERT	Promoter	targeted sequencing	Jang et al. 2021	34209079	Tumor
chr5	NA	NA	TERT	Promoter	targeted sequencing	Jang et al. 2021	34209079	Tumor
chr5	NA	NA	TERT	Promoter	targeted sequencing	Jang et al. 2021	34209079	Tumor
chr5	NA	NA	TERT	Promoter	targeted sequencing	Jang et al. 2021	34209079	Tumor
chr5	NA	NA	TERT	Promoter	targeted sequencing	Jang et al. 2021	34209079	Tumor

Chromosome	Integration site in host genome	Integration site in virus genome	Gene (distance, bp)	Regions	Methods	Author	PMID	Sample
chr5	NA	NA	TERT	Promoter	targeted sequencing	Jang et al. 2021	34209079	Tumor
chr5	NA	NA	TERT	Promoter	targeted sequencing	Jang et al. 2021	34209079	Tumor
chr5	NA	NA	TERT	Promoter	targeted sequencing	Jang et al. 2021	34209079	Tumor
chr5	NA	NA	TERT	Promoter	targeted sequencing	Jang et al. 2021	34209079	Tumor
chr5	NA	NA	TERT	Promoter	targeted sequencing	Jang et al. 2021	34209079	Tumor
chr5	NA	NA	TERT	Promoter	targeted sequencing	Jang et al. 2021	34209079	Tumor
chr5	NA	NA	TERT	Promoter	targeted sequencing	Jang et al. 2021	34209079	Tumor
chr5	NA	NA	TERT	Intron	targeted sequencing	Jang et al. 2021	34209079	Tumor
chr19	NA	NA	KMT2B	Intron	targeted sequencing	Jang et al. 2021	34209079	Tumor
chr19	NA	NA	KMT2B	Intron	targeted sequencing	Jang et al. 2021	34209079	Tumor
chr19	NA	NA	KMT2B	Exon	targeted sequencing	Jang et al. 2021	34209079	Tumor
chr19	NA	NA	KMT2B	Intron	targeted sequencing	Jang et al. 2021	34209079	Tumor
chr8	NA	NA	PREX2	Intron	targeted sequencing	Jang et al. 2021	34209079	Tumor
chr8	NA	NA	PREX2	Intron	targeted sequencing	Jang et al. 2021	34209079	Tumor
chr4	NA	NA	SCFD2	Intron	targeted sequencing	Jang et al. 2021	34209079	Tumor
chr4	NA	NA	SCFD2	Intron	targeted sequencing	Jang et al. 2021	34209079	Tumor
chr10	NA	NA	ADAM12	Intron	targeted sequencing	Jang et al. 2021	34209079	Tumor
chr10	NA	NA	ADAM12	Intron	targeted sequencing	Jang et al. 2021	34209079	Tumor
chr1	197008633	1601	CFHR5	exon (NM_030787, exon 10 of 10)	Long-read sequencing	Zhuo et al. 2021	34642322	Tumor
chr1	95184405	389	LOC101928118	intron (NR_125948, intron 3 of 4)	Long-read sequencing	Zhuo et al. 2021	34642322	Tumor
chr1	232955613	502	NTPCR	non-coding (NR_138027, exon 2 of 5)	Long-read sequencing	Zhuo et al. 2021	34642322	Tumor
chr1	148981417	979	PDE4DIP	intron (NM_001198832, intron 24 of 45)	Long-read sequencing	Zhuo et al. 2021	34642322	Tumor
chr1	67374099	1720	SERBP1	intron (NM_001319233, intron 13 of 15)	Long-read sequencing	Zhuo et al. 2021	34642322	Tumor
chr1	210135567	2542	SERTAD4	intron (NM_153262, intron 6 of 7)	Long-read sequencing	Zhuo et al. 2021	34642322	Tumor
chr1	34828181	1821	SMIM12	Intergenic	Long-read sequencing	Zhuo et al. 2021	34642322	Tumor
chr1	210002252	1720	SYT14	intron (NM_001146261, intron 3 of 9)	Long-read sequencing	Zhuo et al. 2021	34642322	Tumor
chr1	27061063	2802	TENT5B	Intergenic	Long-read sequencing	Zhuo et al. 2021	34642322	Tumor
chr1	29060482	498	TMEM200B	exon (NM_203342, exon 15 of 21)	Long-read sequencing	Zhuo et al. 2021	34642322	Tumor
chr10	51203070	1682	MIR605	intron (NM_001098512, intron 2 of 17)	Long-read sequencing	Zhuo et al. 2021	34642322	Tumor
chr10	95299175	1826	PDLIM1	Intergenic	Long-read sequencing	Zhuo et al. 2021	34642322	Tumor
chr10	95461715	1801	SORBS1	intron (NM_001290295, intron 2 of 24)	Long-read sequencing	Zhuo et al. 2021	34642322	Tumor
chr10	112509988	1778	ZDHHC6	intron (NM_001365712, intron 3 of 8)	Long-read sequencing	Zhuo et al. 2021	34642322	Tumor
chr11	116836046	557	APOA1-AS	promoter-TSS (NR_126362)	Long-read sequencing	Zhuo et al. 2021	34642322	Tumor
chr11	6431456	181	HPX	exon (NM_000613, exon 10 of 10)	Long-read sequencing	Zhuo et al. 2021	34642322	Tumor
chr11	38868026	889	LINC01493	Intergenic	Long-read sequencing	Zhuo et al. 2021	34642322	Tumor
chr11	106375881	1821	LOC101928535	Intergenic	Long-read sequencing	Zhuo et al. 2021	34642322	Tumor
chr11	91218255	1782	MIR1261	Intergenic	Long-read sequencing	Zhuo et al. 2021	34642322	Tumor
chr11	119018344	352	RPS25	promoter-TSS (NM_016146).2	Long-read sequencing	Zhuo et al. 2021	34642322	Tumor
chr12	2496290	403	CACNA1C-AS2	intron (NM_199460, intron 7 of 49).2	Long-read sequencing	Zhuo et al. 2021	34642322	Tumor
chr12	50925210	330	METTL7A	exon (NM_014033, exon 1 of 2)	Long-read sequencing	Zhuo et al. 2021	34642322	Tumor
chr13	68045881	1826	LINC00364	Intergenic	Long-read sequencing	Zhuo et al. 2021	34642322	Tumor
chr13	90398023	1264	LINC01049	Intergenic	Long-read sequencing	Zhuo et al. 2021	34642322	Tumor
chr14	34945645	1826	IGBP1P1	intron (NR_151701, intron 2 of 4)	Long-read sequencing	Zhuo et al. 2021	34642322	Tumor
chr14	94290316	1549	SERPINA10	exon (NM_001100607, exon 2 of 5)	Long-read sequencing	Zhuo et al. 2021	34642322	Tumor
chr17	72423515	338	LINC02003	intron (NR_152516, intron 2 of 3)	Long-read sequencing	Zhuo et al. 2021	34642322	Tumor
chr17	72423514	342	LINC02003	intron (NR_152516, intron 2 of 3)	Long-read sequencing	Zhuo et al. 2021	34642322	Tumor
chr18	25819309	1812	SS18	Intergenic	Long-read sequencing	Zhuo et al. 2021	34642322	Tumor
chr18	245132	1593	THOC1	intron (NM_005131, intron 11 of 20)	Long-read sequencing	Zhuo et al. 2021	34642322	Tumor
chr19	44907953	751	APOE	intron (NM_000041, intron 3 of 3)	Long-read sequencing	Zhuo et al. 2021	34642322	Tumor
chr19	29835827	343	CCNE1	Intergenic	Long-read sequencing	Zhuo et al. 2021	34642322	Tumor
chr19	29838018	2373	CCNE1	Intergenic	Long-read sequencing	Zhuo et al. 2021	34642322	Tumor
chr19	29835820	2447	CCNE1	Intergenic	Long-read sequencing	Zhuo et al. 2021	34642322	Tumor
chr19	29822327	339	CCNE1	exon (NM_001322261, exon 9 of 11)	Long-read sequencing	Zhuo et al. 2021	34642322	Tumor
chr19	29835822	3018	CCNE1	Intergenic	Long-read sequencing	Zhuo et al. 2021	34642322	Tumor
chr19	29840357	2574	CCNE1	Intergenic	Long-read sequencing	Zhuo et al. 2021	34642322	Tumor
chr19	29835821	339	CCNE1	Intergenic	Long-read sequencing	Zhuo et al. 2021	34642322	Tumor
chr19	4052355	1114	ZBTB7A	intron (NM_015898, intron 2 of 2)	Long-read sequencing	Zhuo et al. 2021	34642322	Tumor
chr2	210658650	846	CPS1-IT1	exon (NM_001122633, exon 32 of 39)	Long-read sequencing	Zhuo et al. 2021	34642322	Tumor
chr2	170985141	1828	GORASP2	Intergenic	Long-read sequencing	Zhuo et al. 2021	34642322	Tumor
chr2	170985174	1548	GORASP2	Intergenic	Long-read sequencing	Zhuo et al. 2021	34642322	Tumor
chr2	193411009	1812	PCGEM1	Intergenic	Long-read sequencing	Zhuo et al. 2021	34642322	Tumor
chr20	38225600	1787	LOC149684	intron (NM_001348708, intron 6 of 11)	Long-read sequencing	Zhuo et al. 2021	34642322	Tumor
chr20	17660056	1547	RRBP1	exon (NM_004587, exon 2 of 25)	Long-read sequencing	Zhuo et al. 2021	34642322	Tumor
chr22	33519360	1550	MIR4764	intron (NM_133642, intron 6 of 14)	Long-read sequencing	Zhuo et al. 2021	34642322	Tumor
chr3	52790747	331	ITIH3	intron (NM_002215, intron 19 of 21)	Long-read sequencing	Zhuo et al. 2021	34642322	Tumor
chr3	186727255	520	KNG1	exon (NM_000893, exon 5 of 11)	Long-read sequencing	Zhuo et al. 2021	34642322	Tumor
chr3	118759230	1821	LOC105374060	intron (NR_135547, intron 1 of 3)	Long-read sequencing	Zhuo et al. 2021	34642322	Tumor
chr3	197088886	234	MELTF	intron (NM_001366216, intron 14 of 23)	Long-read sequencing	Zhuo et al. 2021	34642322	Tumor
chr3	18235817	1795	SATB1	intron (NR_161333, intron 4 of 4)	Long-read sequencing	Zhuo et al. 2021	34642322	Tumor
chr3	133778638	356	SRPRB	3' UTR (NM_001354703, exon 23 of 23)	Long-read sequencing	Zhuo et al. 2021	34642322	Tumor
chr3	143025560	1385	U2SURP	intron (NM_001320222, intron 14 of 28)	Long-read sequencing	Zhuo et al. 2021	34642322	Tumor
chr4	73484390	380	AFM	exon (NM_001133, exon 3 of 15)	Long-read sequencing	Zhuo et al. 2021	34642322	Tumor
chr4	73405146	666	ALB	exon (NM_000477, exon 2 of 15)	Long-read sequencing	Zhuo et al. 2021	34642322	Tumor
chr4	73404442	1789	ALB	intron (NM_000477, intron 1 of 14)	Long-read sequencing	Zhuo et al. 2021	34642322	Tumor
chr4	73409455	157	ALB	exon (NM_000477, exon 5 of 15)	Long-read sequencing	Zhuo et al. 2021	34642322	Tumor
chr4	73418168	435	ALB	exon (NM_000477, exon 12 of 15)	Long-read sequencing	Zhuo et al. 2021	34642322	Tumor
chr4	73417606	1015	ALB	exon (NM_000477, exon 11 of 15)	Long-read sequencing	Zhuo et al. 2021	34642322	Tumor
chr4	35421035	1652	ARAP2	Intergenic	Long-read sequencing	Zhuo et al. 2021	34642322	Tumor
chr4	154584180	178	FGA	exon (NM_000508, exon 6 of 6)	Long-read sequencing	Zhuo et al. 2021	34642322	Tumor
chr4	154571484	1805	FGB	3' UTR (NM_001184741, exon 8 of 8)	Long-read sequencing	Zhuo et al. 2021	34642322	Tumor
chr4	154567637	507	FGB	exon (NM_001184741, exon 4 of 8)	Long-read sequencing	Zhuo et al. 2021	34642322	Tumor
chr4	136496956	1425	LINC00613	Intergenic	Long-read sequencing	Zhuo et al. 2021	34642322	Tumor
chr4	188492316	1822	LINC01060	intron (NR_033869, intron 4 of 4)	Long-read sequencing	Zhuo et al. 2021	34642322	Tumor
chr4	34691477	1827	LINC02484	Intergenic	Long-read sequencing	Zhuo et al. 2021	34642322	Tumor
chr5	159895085	2887	ADRA1B	Intergenic	Long-read sequencing	Zhuo et al. 2021	34642322	Tumor
chr5	79115831	928	BHMT	exon (NM_001713, exon 2 of 8)	Long-read sequencing	Zhuo et al. 2021	34642322	Tumor
chr5	66355288	1725	LINC02065	Intergenic	Long-read sequencing	Zhuo et al. 2021	34642322	Tumor
chr5	50924903	3215	LINC02106	Intergenic	Long-read sequencing	Zhuo et al. 2021	34642322	Tumor
chr5	50924912	518	LINC02106	Intergenic	Long-read sequencing	Zhuo et al. 2021	34642322	Tumor
chr5	168122493	1808	MIR12125	intron (NM_001368145, intron 9 of 26)	Long-read sequencing	Zhuo et al. 2021	34642322	Tumor
chr5	168111152	1806	MIR12125	intron (NM_001368145, intron 8 of 26)	Long-read sequencing	Zhuo et al. 2021	34642322	Tumor
chr5	1304552	531	MIR4457	Intergenic	Long-read sequencing	Zhuo et al. 2021	34642322	Tumor
chr5	1295069	1900	TERT	promoter-TSS (NM_001193376)	Long-read sequencing	Zhuo et al. 2021	34642322	Tumor
chr5	16479761	1817	ZNF622	intron (NM_019000, intron 3 of 6)	Long-read sequencing	Zhuo et al. 2021	34642322	Tumor

Chromosome	Integration site in host genome	Integration site in virus genome	Gene (distance, bp)	Regions	Methods	Author	PMID	Sample
chr5	16479782	2511	ZNF622	intron (NM_019000, intron 3 of 6)	Long-read sequencing	Zhuo et al. 2021	34642322	Tumor
chr6	69782091	2336	LMBRD1	intron (NM_001367272, intron 2 of 15)	Long-read sequencing	Zhuo et al. 2021	34642322	Tumor
chr6	157966823	1817	SYNJ2	Intergenic	Long-read sequencing	Zhuo et al. 2021	34642322	Tumor
chr6	52673908	693	TMEM14A	intron (NM_014051, intron 1 of 4)	Long-read sequencing	Zhuo et al. 2021	34642322	Tumor
chr6	52673899	1824	TMEM14A	intron (NM_014051, intron 1 of 4)	Long-read sequencing	Zhuo et al. 2021	34642322	Tumor
chr7	136528637	1821	CHRM2	Intergenic	Long-read sequencing	Zhuo et al. 2021	34642322	Tumor
chr7	144519873	1821	NOBOX	intron (NM_001042482, intron 7 of 7)	Long-read sequencing	Zhuo et al. 2021	34642322	Tumor
chr7	99866628	135	OR2AE1	TTS (NM_022820)	Long-read sequencing	Zhuo et al. 2021	34642322	Tumor
chr7	44759488	2821	ZMIZ2	intron (NM_174929, intron 6 of 16)	Long-read sequencing	Zhuo et al. 2021	34642322	Tumor
chr8	139993954	1826	PEG13	intron (NM_001160372, intron 18 of 22)	Long-read sequencing	Zhuo et al. 2021	34642322	Tumor
chr8	52657773	970	RB1CC1	exon (NM_014781, exon 15 of 24)	Long-read sequencing	Zhuo et al. 2021	34642322	Tumor
chr9	35704719	1570	MIR6852	exon (NM_006289, exon 44 of 57)	Long-read sequencing	Zhuo et al. 2021	34642322	Tumor
chr17	21526569	1759	C17orf51	-71627	targeted sequencing	Péneau et al. 2021	33563643	Tumor
chr1	17067040	1790	FAM231A|FAM231C	-6053	targeted sequencing	Péneau et al. 2021	33563643	Tumor
chr1	17067141	171	FAM231A|FAM231C	-6053	targeted sequencing	Péneau et al. 2021	33563643	Tumor
chr8	93599480	366	LOC102724710	0	targeted sequencing	Péneau et al. 2021	33563643	Tumor
chr14	35025465	171	SNX6	4526	targeted sequencing	Péneau et al. 2021	33563643	Tumor
chr14	35025566	1790	SNX6	4526	targeted sequencing	Péneau et al. 2021	33563643	Tumor
chr5	1295436	1810	TERT	0	targeted sequencing	Péneau et al. 2021	33563643	Tumor
chr5	1295478	1070	TERT	0	targeted sequencing	Péneau et al. 2021	33563643	Tumor
chr7	137659742	2717	CREB3L2	0	targeted sequencing	Péneau et al. 2021	33563643	Tumor
chr9	88712716	628	GOLM1	0	targeted sequencing	Péneau et al. 2021	33563643	Tumor
chr12	94317158	1791	LOC105369911	0	targeted sequencing	Péneau et al. 2021	33563643	Tumor
chr9	134818426	2121	MED27	0	targeted sequencing	Péneau et al. 2021	33563643	Tumor
chr14	81932115	1807	SEL1L	5707	targeted sequencing	Péneau et al. 2021	33563643	Tumor
chr14	81932117	1834	SEL1L	5707	targeted sequencing	Péneau et al. 2021	33563643	Tumor
chr10	60158072	1914	TFAM	0	targeted sequencing	Péneau et al. 2021	33563643	Tumor
chr4	70390896	1842	UGT2B4	0	targeted sequencing	Péneau et al. 2021	33563643	Tumor
chr4	179148021	1879	LINC01098	-235378	targeted sequencing	Péneau et al. 2021	33563643	Tumor
chr4	179151059	3043	LINC01098	-239151	targeted sequencing	Péneau et al. 2021	33563643	Tumor
chr2	31264256	1883	GALNT14	0	targeted sequencing	Péneau et al. 2021	33563643	Tumor
chr2	31264267	1830	GALNT14	0	targeted sequencing	Péneau et al. 2021	33563643	Tumor
chr5	1303841	1926	MIR4457	5582	targeted sequencing	Péneau et al. 2021	33563643	Tumor
chr11	51579542	2744	OR4C46	-63220	targeted sequencing	Péneau et al. 2021	33563643	Tumor
chr11	51590598	562	OR4C46	-74075	targeted sequencing	Péneau et al. 2021	33563643	Tumor
chr5	32363272	99	ZFR	0	targeted sequencing	Péneau et al. 2021	33563643	Tumor
chr19	27733199	1808	LINC00662	547932	targeted sequencing	Péneau et al. 2021	33563643	Tumor
chr14	93369848	811	LOC101929002	2194	targeted sequencing	Péneau et al. 2021	33563643	Tumor
chr2	4344876	246	LOC105373394	-322006	targeted sequencing	Péneau et al. 2021	33563643	Tumor
chr2	243152581	1831	LOC728323	-50006	targeted sequencing	Péneau et al. 2021	33563643	Tumor
chrY	13654	1769	PLCXD1	129114	targeted sequencing	Péneau et al. 2021	33563643	Tumor
chrY	23455	1797	PLCXD1	119536	targeted sequencing	Péneau et al. 2021	33563643	Tumor
chr6	155432540	2341	TIAM2	0	targeted sequencing	Péneau et al. 2021	33563643	Tumor
chr14	96342886	2514	TUNAR	0	targeted sequencing	Péneau et al. 2021	33563643	Tumor
chr4	30454	1799	ZNF595	22161	targeted sequencing	Péneau et al. 2021	33563643	Tumor
chr8	73818707	1789	KCNB2	0	targeted sequencing	Péneau et al. 2021	33563643	Tumor
chr8	36161970	1672	KCNU1	479281	targeted sequencing	Péneau et al. 2021	33563643	Tumor
chr5	43938293	1833	NNT	-232043	targeted sequencing	Péneau et al. 2021	33563643	Tumor
chr3	15161315	2003	RBSN	-20651	targeted sequencing	Péneau et al. 2021	33563643	Tumor
chr1	45006610	1104	RNF220	0	targeted sequencing	Péneau et al. 2021	33563643	Tumor
chr4	83630123	2384	SCD5	0	targeted sequencing	Péneau et al. 2021	33563643	Tumor
chr9	39229229	1668	CNTNAP3	0	targeted sequencing	Péneau et al. 2021	33563643	Tumor
chr9	39229233	1881	CNTNAP3	0	targeted sequencing	Péneau et al. 2021	33563643	Tumor
chr1	10095	1884	DDX11L1	1431	targeted sequencing	Péneau et al. 2021	33563643	Tumor
chr2	14754528	698	FAM84A	17760	targeted sequencing	Péneau et al. 2021	33563643	Tumor
chr20	62918546	1544	LINC00266-1	3078	targeted sequencing	Péneau et al. 2021	33563643	Tumor
chr10	42596976	1174	LOC441666	230102	targeted sequencing	Péneau et al. 2021	33563643	Tumor
chr2	184665916	2936	MIR548AE1	577238	targeted sequencing	Péneau et al. 2021	33563643	Tumor
chr8	11640829	2123	NEIL2	0	targeted sequencing	Péneau et al. 2021	33563643	Tumor
chr2	159400924	2663	PKP4	0	targeted sequencing	Péneau et al. 2021	33563643	Tumor
chr9	19758493	1399	SLC24A2	0	targeted sequencing	Péneau et al. 2021	33563643	Tumor
chr5	1400480	2940	SLC6A3	0	targeted sequencing	Péneau et al. 2021	33563643	Tumor
chr18	53163448	1601	TCF4	0	targeted sequencing	Péneau et al. 2021	33563643	Tumor
chr5	1297155	1762	TERT	-1167	targeted sequencing	Péneau et al. 2021	33563643	Tumor
chr5	1298719	843	TERT	-3556	targeted sequencing	Péneau et al. 2021	33563643	Tumor
chrY	9959566	949	TTTY23|TTTY23B	-209896	targeted sequencing	Péneau et al. 2021	33563643	Tumor
chr9	25453239	1611	TUSC1	223148	targeted sequencing	Péneau et al. 2021	33563643	Tumor
chr5	114678598	2722	CCDC112	-45967	targeted sequencing	Péneau et al. 2021	33563643	Tumor
chr8	87835883	1836	CNBD1	42352	targeted sequencing	Péneau et al. 2021	33563643	Tumor
chr1	37016941	2665	CSF3R	-67892	targeted sequencing	Péneau et al. 2021	33563643	Tumor
chr4	49104593	1401	CWH43	-40498	targeted sequencing	Péneau et al. 2021	33563643	Tumor
chr1	204120659	1812	ETNK2	0	targeted sequencing	Péneau et al. 2021	33563643	Tumor
chr4	8553366	162	GPR78	28406	targeted sequencing	Péneau et al. 2021	33563643	Tumor
chr4	8553368	2651	GPR78	28406	targeted sequencing	Péneau et al. 2021	33563643	Tumor
chr5	5734844	1826	ICE1	-244497	targeted sequencing	Péneau et al. 2021	33563643	Tumor
chr10	4441544	1610	LINC00703	0	targeted sequencing	Péneau et al. 2021	33563643	Tumor
chr3	162673323	22	LINC01192	221708	targeted sequencing	Péneau et al. 2021	33563643	Tumor
chr10	9658279	2596	LOC101928272	-320542	targeted sequencing	Péneau et al. 2021	33563643	Tumor
chr2	156653394	421	LOC101929378	223653	targeted sequencing	Péneau et al. 2021	33563643	Tumor
chr10	3522238	1821	LOC105376360	0	targeted sequencing	Péneau et al. 2021	33563643	Tumor
chr2	148835235	1252	MBD5	0	targeted sequencing	Péneau et al. 2021	33563643	Tumor
chr2	172256289	2032	METTL8	0	targeted sequencing	Péneau et al. 2021	33563643	Tumor
chr14	34099896	1932	NPAS3	0	targeted sequencing	Péneau et al. 2021	33563643	Tumor
chr13	25071140	3151	PARP4	0	targeted sequencing	Péneau et al. 2021	33563643	Tumor
chr13	33353503	1927	PDS5B	-1074	targeted sequencing	Péneau et al. 2021	33563643	Tumor
chr6	79807461	1852	PHIP	-18842	targeted sequencing	Péneau et al. 2021	33563643	Tumor
chr15	24107562	1822	PWRN4	112380	targeted sequencing	Péneau et al. 2021	33563643	Tumor
chr15	24107572	1932	PWRN4	112380	targeted sequencing	Péneau et al. 2021	33563643	Tumor
chr14	70243355	2294	SLC10A1	0	targeted sequencing	Péneau et al. 2021	33563643	Tumor
chr14	70243375	1341	SLC10A1	0	targeted sequencing	Péneau et al. 2021	33563643	Tumor
chr12	24101103	1799	SOX5	0	targeted sequencing	Péneau et al. 2021	33563643	Tumor
chr12	24101125	1820	SOX5	0	targeted sequencing	Péneau et al. 2021	33563643	Tumor
chrX	24351805	2513	SUPT20HL2	-20372	targeted sequencing	Péneau et al. 2021	33563643	Tumor
chrX	24351963	3206	SUPT20HL2	-20372	targeted sequencing	Péneau et al. 2021	33563643	Tumor

Chromosome	Integration site in host genome	Integration site in virus genome	Gene (distance, bp)	Regions	Methods	Author	PMID	Sample
chr4	48256775	365	TEC	0	targeted sequencing	Péneau et al. 2021	33563643	Tumor
chr12	83472518	2322	TMTC2	0	targeted sequencing	Péneau et al. 2021	33563643	Tumor
chrY	7920895	1221	TTTY12	-242172	targeted sequencing	Péneau et al. 2021	33563643	Tumor
chrY	7937776	1807	TTTY12	-258538	targeted sequencing	Péneau et al. 2021	33563643	Tumor
chr15	100599016	1704	ADAMTS17	0	targeted sequencing	Péneau et al. 2021	33563643	Tumor
chr1	32240571	1864	ADGRB2	-10907	targeted sequencing	Péneau et al. 2021	33563643	Tumor
chr6	47070448	2779	ADGRF1	-59976	targeted sequencing	Péneau et al. 2021	33563643	Tumor
chr1	15924374	675	AGMAT	-12448	targeted sequencing	Péneau et al. 2021	33563643	Tumor
chr7	15280133	1024	AGMO	0	targeted sequencing	Péneau et al. 2021	33563643	Tumor
chr6	131628643	1820	AKAP7	-23968	targeted sequencing	Péneau et al. 2021	33563643	Tumor
chr16	56410762	2464	AMFR	0	targeted sequencing	Péneau et al. 2021	33563643	Tumor
chr12	101419905	1916	ANO4	0	targeted sequencing	Péneau et al. 2021	33563643	Tumor
chr5	77415568	437	AP3B1	0	targeted sequencing	Péneau et al. 2021	33563643	Tumor
chr22	36577352	2188	APOL4	7824	targeted sequencing	Péneau et al. 2021	33563643	Tumor
chr4	114866993	2227	ARSJ	0	targeted sequencing	Péneau et al. 2021	33563643	Tumor
chr17	32026712	2507	ASIC2	0	targeted sequencing	Péneau et al. 2021	33563643	Tumor
chr17	40679496	2004	ATP6V0A1	-4899	targeted sequencing	Péneau et al. 2021	33563643	Tumor
chr18	77141105	2494	ATP9B	-2743	targeted sequencing	Péneau et al. 2021	33563643	Tumor
chr17	79014413	2904	BAIAP2	0	targeted sequencing	Péneau et al. 2021	33563643	Tumor
chr17	59227873	1829	BCAS3	0	targeted sequencing	Péneau et al. 2021	33563643	Tumor
chr6	81615195	448	BCKDHB	-559058	targeted sequencing	Péneau et al. 2021	33563643	Tumor
chr2	60724141	315	BCL11A	0	targeted sequencing	Péneau et al. 2021	33563643	Tumor
chr7	112557946	477	BMT2	0	targeted sequencing	Péneau et al. 2021	33563643	Tumor
chr15	83992811	1826	BNC1	-39224	targeted sequencing	Péneau et al. 2021	33563643	Tumor
chr14	36359780	433	BRMS1L	-18412	targeted sequencing	Péneau et al. 2021	33563643	Tumor
chr15	75504525	656	C15orf39	0	targeted sequencing	Péneau et al. 2021	33563643	Tumor
chr15	39528135	44	C15orf54	14598	targeted sequencing	Péneau et al. 2021	33563643	Tumor
chr1	221293252	1492	C1orf140	209815	targeted sequencing	Péneau et al. 2021	33563643	Tumor
chr19	13453862	1202	CACNA1A	0	targeted sequencing	Péneau et al. 2021	33563643	Tumor
chr3	105606402	1566	CBLB	-17906	targeted sequencing	Péneau et al. 2021	33563643	Tumor
chr2	56749688	241	CCDC85A	-136020	targeted sequencing	Péneau et al. 2021	33563643	Tumor
chr1	160833978	317	CD244	-1152	targeted sequencing	Péneau et al. 2021	33563643	Tumor
chr2	174284807	1815	CDCA7	-51089	targeted sequencing	Péneau et al. 2021	33563643	Tumor
chr16	64199510	769	CDH11	778147	targeted sequencing	Péneau et al. 2021	33563643	Tumor
chr5	22505993	1731	CDH12	0	targeted sequencing	Péneau et al. 2021	33563643	Tumor
chr16	83744994	1214	CDH13	0	targeted sequencing	Péneau et al. 2021	33563643	Tumor
chr10	73539930	522	CDH23	0	targeted sequencing	Péneau et al. 2021	33563643	Tumor
chr20	48823779	2048	CEBPB	-14552	targeted sequencing	Péneau et al. 2021	33563643	Tumor
chr22	17641177	361	CECR5|CECR5-AS1	0	targeted sequencing	Péneau et al. 2021	33563643	Tumor
chr4	10513948	2066	CLNK	0	targeted sequencing	Péneau et al. 2021	33563643	Tumor
chr10	104828897	1820	CNNM2	0	targeted sequencing	Péneau et al. 2021	33563643	Tumor
chr10	104828916	1826	CNNM2	0	targeted sequencing	Péneau et al. 2021	33563643	Tumor
chr2	97497162	2063	CNNM3	0	targeted sequencing	Péneau et al. 2021	33563643	Tumor
chr7	52118674	1800	COBL	-734159	targeted sequencing	Péneau et al. 2021	33563643	Tumor
chr1	230757062	3027	COG2	20989	targeted sequencing	Péneau et al. 2021	33563643	Tumor
chr9	101696963	2297	COL15A1	9032	targeted sequencing	Péneau et al. 2021	33563643	Tumor
chr6	55858472	1827	COL21A1	62715	targeted sequencing	Péneau et al. 2021	33563643	Tumor
chr2	190128003	1823	COL5A2	-83398	targeted sequencing	Péneau et al. 2021	33563643	Tumor
chr6	49634383	1058	CRISP2	25525	targeted sequencing	Péneau et al. 2021	33563643	Tumor
chr1	17235701	1817	CROCC	12547	targeted sequencing	Péneau et al. 2021	33563643	Tumor
chr1	17235709	1089	CROCC	12547	targeted sequencing	Péneau et al. 2021	33563643	Tumor
chr12	58236363	316	CTDSP2	0	targeted sequencing	Péneau et al. 2021	33563643	Tumor
chr2	80784935	2763	CTNNA2	0	targeted sequencing	Péneau et al. 2021	33563643	Tumor
chr6	84572567	2834	CYB5R4	0	targeted sequencing	Péneau et al. 2021	33563643	Tumor
chr16	50906502	1821	CYLD	-70552	targeted sequencing	Péneau et al. 2021	33563643	Tumor
chr1	47481237	2343	CYP4X1	0	targeted sequencing	Péneau et al. 2021	33563643	Tumor
chr13	72072350	334	DACH1	0	targeted sequencing	Péneau et al. 2021	33563643	Tumor
chr2	3911090	907	DCDC2C	-15912	targeted sequencing	Péneau et al. 2021	33563643	Tumor
chr11	31005506	2832	DCDC5	0	targeted sequencing	Péneau et al. 2021	33563643	Tumor
chr1	20984304	1287	DDOST	0	targeted sequencing	Péneau et al. 2021	33563643	Tumor
chr9	118285098	740	DEC1	-120175	targeted sequencing	Péneau et al. 2021	33563643	Tumor
chr8	6925191	157	DEFA5	-10805	targeted sequencing	Péneau et al. 2021	33563643	Tumor
chr4	24605923	1834	DHX15	-19739	targeted sequencing	Péneau et al. 2021	33563643	Tumor
chr1	232164673	1821	DISC1|TSNAX-DISC1	0	targeted sequencing	Péneau et al. 2021	33563643	Tumor
chr1	15867528	1789	DNAJC16	0	targeted sequencing	Péneau et al. 2021	33563643	Tumor
chr1	97541893	436	DPYD	1195	targeted sequencing	Péneau et al. 2021	33563643	Tumor
chr14	19642216	1538	DUXAP10	7816	targeted sequencing	Péneau et al. 2021	33563643	Tumor
chr17	28402762	2795	EFCAB5	0	targeted sequencing	Péneau et al. 2021	33563643	Tumor
chr8	132742741	2023	EFR3A	173424	targeted sequencing	Péneau et al. 2021	33563643	Tumor
chr7	36902953	594	ELMO1	0	targeted sequencing	Péneau et al. 2021	33563643	Tumor
chr1	121484484	1823	EMBP1	-170741	targeted sequencing	Péneau et al. 2021	33563643	Tumor
chr8	72193698	431	EYA1	0	targeted sequencing	Péneau et al. 2021	33563643	Tumor
chr13	108315260	674	FAM155A	0	targeted sequencing	Péneau et al. 2021	33563643	Tumor
chr5	119153892	347	FAM170A	-182255	targeted sequencing	Péneau et al. 2021	33563643	Tumor
chr1	166165645	337	FAM78B	-29546	targeted sequencing	Péneau et al. 2021	33563643	Tumor
chr1	172722379	2999	FASLG	-86257	targeted sequencing	Péneau et al. 2021	33563643	Tumor
chr2	48108864	2333	FBXO11	0	targeted sequencing	Péneau et al. 2021	33563643	Tumor
chr9	95805377	764	FGD3	-6859	targeted sequencing	Péneau et al. 2021	33563643	Tumor
chr5	176531699	1777	FGFR4	-6359	targeted sequencing	Péneau et al. 2021	33563643	Tumor
chr16	35038436	338	FLJ26245	-47441	targeted sequencing	Péneau et al. 2021	33563643	Tumor
chr15	33321017	536	FMN1	0	targeted sequencing	Péneau et al. 2021	33563643	Tumor
chr1	240537815	2050	FMN2	0	targeted sequencing	Péneau et al. 2021	33563643	Tumor
chr12	10375062	2144	GABARAPL1	0	targeted sequencing	Péneau et al. 2021	33563643	Tumor
chr12	10375150	2247	GABARAPL1	0	targeted sequencing	Péneau et al. 2021	33563643	Tumor
chr14	88415288	456	GALC	0	targeted sequencing	Péneau et al. 2021	33563643	Tumor
chr2	155083736	757	GALNT13	0	targeted sequencing	Péneau et al. 2021	33563643	Tumor
chr1	230230300	395	GALNT2	0	targeted sequencing	Péneau et al. 2021	33563643	Tumor
chr4	72743354	764	GC	-72117	targeted sequencing	Péneau et al. 2021	33563643	Tumor
chr2	233564185	3008	GIGYF2	0	targeted sequencing	Péneau et al. 2021	33563643	Tumor
chr2	233682245	642	GIGYF2	0	targeted sequencing	Péneau et al. 2021	33563643	Tumor
chr4	44725976	256	GNPDA2	0	targeted sequencing	Péneau et al. 2021	33563643	Tumor
chr3	121476877	1024	GOLGB1	-8086	targeted sequencing	Péneau et al. 2021	33563643	Tumor
chr13	92425285	266	GPC5	0	targeted sequencing	Péneau et al. 2021	33563643	Tumor
chr6	110289740	724	GPR6	9719	targeted sequencing	Péneau et al. 2021	33563643	Tumor

Chromosome	Integration site in host genome	Integration site in virus genome	Gene (distance, bp)	Regions	Methods	Author	PMID	Sample
chr6	52787104	2598	GSTA3	-12437	targeted sequencing	Péneau et al. 2021	33563643	Tumor
chr7	66294152	1615	GTF2IRD1P1	0	targeted sequencing	Péneau et al. 2021	33563643	Tumor
chrX	83808287	2472	HDX	-50800	targeted sequencing	Péneau et al. 2021	33563643	Tumor
chr1	210757996	319	HHAT	0	targeted sequencing	Péneau et al. 2021	33563643	Tumor
chr6	55334768	1396	HMGCLL1	0	targeted sequencing	Péneau et al. 2021	33563643	Tumor
chr6	122684471	3176	HSF2	36060	targeted sequencing	Péneau et al. 2021	33563643	Tumor
chr6	87589839	1837	HTR1E	57185	targeted sequencing	Péneau et al. 2021	33563643	Tumor
chr3	9965945	3034	IL17RC	0	targeted sequencing	Péneau et al. 2021	33563643	Tumor
chr16	19734774	1807	IQCK	0	targeted sequencing	Péneau et al. 2021	33563643	Tumor
chr16	19768934	332	IQCK	0	targeted sequencing	Péneau et al. 2021	33563643	Tumor
chr6	15511249	2957	JARID2	0	targeted sequencing	Péneau et al. 2021	33563643	Tumor
chr3	124288289	1591	KALRN	0	targeted sequencing	Péneau et al. 2021	33563643	Tumor
chr8	111393944	318	KCNV1	-405868	targeted sequencing	Péneau et al. 2021	33563643	Tumor
chr2	8918683	2751	KIDINS220	0	targeted sequencing	Péneau et al. 2021	33563643	Tumor
chr1	44567892	2342	KLF17	16499	targeted sequencing	Péneau et al. 2021	33563643	Tumor
chr12	52867490	3015	KRT6C	0	targeted sequencing	Péneau et al. 2021	33563643	Tumor
chr9	130903913	1627	LCN2	7572	targeted sequencing	Péneau et al. 2021	33563643	Tumor
chr17	76969528	3006	LGALS3BP	0	targeted sequencing	Péneau et al. 2021	33563643	Tumor
chr1	202257944	715	LGR6	0	targeted sequencing	Péneau et al. 2021	33563643	Tumor
chr21	42505990	2049	LINC00323	7437	targeted sequencing	Péneau et al. 2021	33563643	Tumor
chr13	106760485	315	LINC00460	268426	targeted sequencing	Péneau et al. 2021	33563643	Tumor
chr14	101773791	2046	LINC00524	98533	targeted sequencing	Péneau et al. 2021	33563643	Tumor
chr14	48723946	917	LINC00648	-459536	targeted sequencing	Péneau et al. 2021	33563643	Tumor
chr1	194421648	2932	LINC01031	-1086565	targeted sequencing	Péneau et al. 2021	33563643	Tumor
chr10	133655134	1679	LINC01164	-32364	targeted sequencing	Péneau et al. 2021	33563643	Tumor
chr2	235578967	2802	LINC01173	12345	targeted sequencing	Péneau et al. 2021	33563643	Tumor
chr3	162482276	2949	LINC01192	412755	targeted sequencing	Péneau et al. 2021	33563643	Tumor
chr4	190411037	558	LINC01262	169723	targeted sequencing	Péneau et al. 2021	33563643	Tumor
chr1	234681765	2385	LINC01354	-14240	targeted sequencing	Péneau et al. 2021	33563643	Tumor
chr4	118481813	1008	LINC01378	0	targeted sequencing	Péneau et al. 2021	33563643	Tumor
chr14	82630279	1049	LINC01467	-540641	targeted sequencing	Péneau et al. 2021	33563643	Tumor
chr6	82559397	849	LINC01526	-35488	targeted sequencing	Péneau et al. 2021	33563643	Tumor
chr5	96440398	1820	LIX1	0	targeted sequencing	Péneau et al. 2021	33563643	Tumor
chr3	8451310	301	LMCD1-AS1	0	targeted sequencing	Péneau et al. 2021	33563643	Tumor
chr12	25682811	3025	LMNTD1	0	targeted sequencing	Péneau et al. 2021	33563643	Tumor
chr21	47107877	785	LOC100129027	139724	targeted sequencing	Péneau et al. 2021	33563643	Tumor
chrUn_gl000220	124322	665	LOC100507412	0	targeted sequencing	Péneau et al. 2021	33563643	Tumor
chr15	22340848	250	LOC101927079|LOC727924	0	targeted sequencing	Péneau et al. 2021	33563643	Tumor
chr2	186467545	2039	LOC101927196	116941	targeted sequencing	Péneau et al. 2021	33563643	Tumor
chr4	132565032	1820	LOC101927305	120961	targeted sequencing	Péneau et al. 2021	33563643	Tumor
chr16	50987190	934	LOC101927334	64332	targeted sequencing	Péneau et al. 2021	33563643	Tumor
chr10	106277914	1958	LOC101927523	-37670	targeted sequencing	Péneau et al. 2021	33563643	Tumor
chr6	113671760	2224	LOC101927686	272817	targeted sequencing	Péneau et al. 2021	33563643	Tumor
chr2	68078350	1225	LOC101927701	-25344	targeted sequencing	Péneau et al. 2021	33563643	Tumor
chrX	4393705	2956	LOC101928201	151536	targeted sequencing	Péneau et al. 2021	33563643	Tumor
chr6	69286119	1031	LOC101928307	56091	targeted sequencing	Péneau et al. 2021	33563643	Tumor
chr1	105642884	1781	LOC101928476	489304	targeted sequencing	Péneau et al. 2021	33563643	Tumor
chr1	177744129	646	LOC101928778	-64487	targeted sequencing	Péneau et al. 2021	33563643	Tumor
chr2	151726368	2484	LOC101929282	-234313	targeted sequencing	Péneau et al. 2021	33563643	Tumor
chr11	127754978	2070	LOC101929497	-548050	targeted sequencing	Péneau et al. 2021	33563643	Tumor
chr2	165718680	2741	LOC101929633	-12774	targeted sequencing	Péneau et al. 2021	33563643	Tumor
chr6	138016968	3155	LOC102723649	-21277	targeted sequencing	Péneau et al. 2021	33563643	Tumor
chr21	34352732	2364	LOC102724502	-19860	targeted sequencing	Péneau et al. 2021	33563643	Tumor
chr2	213618617	1674	LOC102725079	41438	targeted sequencing	Péneau et al. 2021	33563643	Tumor
chr2	213670352	1824	LOC102725079	0	targeted sequencing	Péneau et al. 2021	33563643	Tumor
chr3	117824711	2520	LOC105374060	402356	targeted sequencing	Péneau et al. 2021	33563643	Tumor
chr18	62283454	2636	LOC284294	-192627	targeted sequencing	Péneau et al. 2021	33563643	Tumor
chr8	20830112	1067	LOC286114	1385	targeted sequencing	Péneau et al. 2021	33563643	Tumor
chr4	188208822	1108	LOC339975	16415	targeted sequencing	Péneau et al. 2021	33563643	Tumor
chr7	136725402	483	LOC349160	0	targeted sequencing	Péneau et al. 2021	33563643	Tumor
chr18	74432541	2610	LOC400661	-26829	targeted sequencing	Péneau et al. 2021	33563643	Tumor
chr5	18033538	449	LOC646241	-102940	targeted sequencing	Péneau et al. 2021	33563643	Tumor
chr5	177303220	2299	LOC728554	0	targeted sequencing	Péneau et al. 2021	33563643	Tumor
chr3	188196492	2618	LPP	0	targeted sequencing	Péneau et al. 2021	33563643	Tumor
chr14	43005642	2475	LRFN5	-631715	targeted sequencing	Péneau et al. 2021	33563643	Tumor
chr3	66637068	1789	LRIG1	-86223	targeted sequencing	Péneau et al. 2021	33563643	Tumor
chr20	39285061	1789	MAFB	29287	targeted sequencing	Péneau et al. 2021	33563643	Tumor
chr7	77694529	677	MAGI2	0	targeted sequencing	Péneau et al. 2021	33563643	Tumor
chr10	99461772	27	MARVELD1	11530	targeted sequencing	Péneau et al. 2021	33563643	Tumor
chr8	98921326	1002	MATN2	0	targeted sequencing	Péneau et al. 2021	33563643	Tumor
chr2	63856433	1915	MDH1	-22103	targeted sequencing	Péneau et al. 2021	33563643	Tumor
chr3	196751411	1813	MELTF	0	targeted sequencing	Péneau et al. 2021	33563643	Tumor
chr12	95895724	1224	METAP2	0	targeted sequencing	Péneau et al. 2021	33563643	Tumor
chr10	131305365	1822	MGMT	0	targeted sequencing	Péneau et al. 2021	33563643	Tumor
chr10	131305831	1975	MGMT	0	targeted sequencing	Péneau et al. 2021	33563643	Tumor
chr10	131394886	1821	MGMT	0	targeted sequencing	Péneau et al. 2021	33563643	Tumor
chr1	67409541	342	MIER1	0	targeted sequencing	Péneau et al. 2021	33563643	Tumor
chr6	120376451	353	MIR3144	-39933	targeted sequencing	Péneau et al. 2021	33563643	Tumor
chr9	112319293	3012	MIR3927	-45352	targeted sequencing	Péneau et al. 2021	33563643	Tumor
chr4	29011821	356	MIR4275	-190531	targeted sequencing	Péneau et al. 2021	33563643	Tumor
chr5	7037506	373	MIR4278	-209472	targeted sequencing	Péneau et al. 2021	33563643	Tumor
chr4	163650495	1609	MIR4454	364096	targeted sequencing	Péneau et al. 2021	33563643	Tumor
chr9	36827901	2888	MIR4475	-4135	targeted sequencing	Péneau et al. 2021	33563643	Tumor
chr15	69317955	1015	MIR548H4|NOX5	0	targeted sequencing	Péneau et al. 2021	33563643	Tumor
chr1	35149441	197	MIR552	-13912	targeted sequencing	Péneau et al. 2021	33563643	Tumor
chr12	66409418	643	MIR6074	7982	targeted sequencing	Péneau et al. 2021	33563643	Tumor
chr18	11654134	255	MIR7153	750	targeted sequencing	Péneau et al. 2021	33563643	Tumor
chr12	88039677	1369	MKRN9P	136986	targeted sequencing	Péneau et al. 2021	33563643	Tumor
chr8	89876585	323	MMP16	-536766	targeted sequencing	Péneau et al. 2021	33563643	Tumor
chr6	132691304	2473	MOXD1	0	targeted sequencing	Péneau et al. 2021	33563643	Tumor
chr8	72555465	2737	MSC	198179	targeted sequencing	Péneau et al. 2021	33563643	Tumor
chr16	56597069	2996	MT4	1892	targeted sequencing	Péneau et al. 2021	33563643	Tumor
chr13	109353701	2956	MYO16	0	targeted sequencing	Péneau et al. 2021	33563643	Tumor
chr2	204069801	1822	NBEAL1	0	targeted sequencing	Péneau et al. 2021	33563643	Tumor

Chromosome	Integration site in host genome	Integration site in virus genome	Gene (distance, bp)	Regions	Methods	Author	PMID	Sample
chr1	16908787	2153	NBPF1	0	targeted sequencing	Péneau et al. 2021	33563643	Tumor
chr2	134303784	1819	NCKAP5	0	targeted sequencing	Péneau et al. 2021	33563643	Tumor
chr12	124962129	347	NCOR2	0	targeted sequencing	Péneau et al. 2021	33563643	Tumor
chr4	116525024	2592	NDST4	-489869	targeted sequencing	Péneau et al. 2021	33563643	Tumor
chr1	41211303	1819	NFYC	0	targeted sequencing	Péneau et al. 2021	33563643	Tumor
chr3	52508041	3101	NISCH	0	targeted sequencing	Péneau et al. 2021	33563643	Tumor
chr16	50770041	485	NOD2	-3053	targeted sequencing	Péneau et al. 2021	33563643	Tumor
chr5	92277669	431	NR2F1-AS1	467393	targeted sequencing	Péneau et al. 2021	33563643	Tumor
chrX	105048533	895	NRK	18003	targeted sequencing	Péneau et al. 2021	33563643	Tumor
chr1	107893871	2482	NTNG1	0	targeted sequencing	Péneau et al. 2021	33563643	Tumor
chr2	220433255	624	OBSL1	0	targeted sequencing	Péneau et al. 2021	33563643	Tumor
chr11	51590843	1828	OR4C46	-74632	targeted sequencing	Péneau et al. 2021	33563643	Tumor
chr2	146919882	1828	PABPC1P2	424555	targeted sequencing	Péneau et al. 2021	33563643	Tumor
chr2	147751349	1020	PABPC1P2	-402791	targeted sequencing	Péneau et al. 2021	33563643	Tumor
chr8	81851121	1928	PAG1	28925	targeted sequencing	Péneau et al. 2021	33563643	Tumor
chr4	80777473	3009	PCAT4	0	targeted sequencing	Péneau et al. 2021	33563643	Tumor
chr7	82298195	2863	PCLO	84949	targeted sequencing	Péneau et al. 2021	33563643	Tumor
chr2	70488884	3005	PCYOX1	0	targeted sequencing	Péneau et al. 2021	33563643	Tumor
chr6	166085959	734	PDE10A	-10215	targeted sequencing	Péneau et al. 2021	33563643	Tumor
chr5	31866768	566	PDZD2	0	targeted sequencing	Péneau et al. 2021	33563643	Tumor
chr12	41802689	298	PDZRN4	0	targeted sequencing	Péneau et al. 2021	33563643	Tumor
chr6	46686408	359	PLA2G7	0	targeted sequencing	Péneau et al. 2021	33563643	Tumor
chr11	16955273	1608	PLEKHA7	0	targeted sequencing	Péneau et al. 2021	33563643	Tumor
chr7	132265186	2023	PLXNA4	0	targeted sequencing	Péneau et al. 2021	33563643	Tumor
chr12	102618221	595	PMCH	-26378	targeted sequencing	Péneau et al. 2021	33563643	Tumor
chr6	89748115	1749	PNRC1	42314	targeted sequencing	Péneau et al. 2021	33563643	Tumor
chr13	29230661	2467	POMP	2480	targeted sequencing	Péneau et al. 2021	33563643	Tumor
chr2	44369557	1823	PPM1B	26226	targeted sequencing	Péneau et al. 2021	33563643	Tumor
chr5	120055948	2317	PRR16	-32813	targeted sequencing	Péneau et al. 2021	33563643	Tumor
chr13	57936412	1722	PRR20A|PRR20B|PRR20C|PRR20D|PRR20E	-192060	targeted sequencing	Péneau et al. 2021	33563643	Tumor
chr8	18447364	625	PSD3	0	targeted sequencing	Péneau et al. 2021	33563643	Tumor
chr9	66495095	835	PTGER4P2-CDK2AP2P2	0	targeted sequencing	Péneau et al. 2021	33563643	Tumor
chr12	15747710	1845	PTPRO	0	targeted sequencing	Péneau et al. 2021	33563643	Tumor
chr4	122413807	1822	QRFPR	-111626	targeted sequencing	Péneau et al. 2021	33563643	Tumor
chr7	140114574	135	RAB19	0	targeted sequencing	Péneau et al. 2021	33563643	Tumor
chr5	58071794	345	RAB3C	0	targeted sequencing	Péneau et al. 2021	33563643	Tumor
chr9	3235728	294	RFX3	0	targeted sequencing	Péneau et al. 2021	33563643	Tumor
chr1	25625532	1783	RHD|RSRP1	0	targeted sequencing	Péneau et al. 2021	33563643	Tumor
chrM	5475	1939	RNR2	-2245	targeted sequencing	Péneau et al. 2021	33563643	Tumor
chrM	6043	3018	RNR2	-2245	targeted sequencing	Péneau et al. 2021	33563643	Tumor
chrM	9191	1603	RNR2	-5961	targeted sequencing	Péneau et al. 2021	33563643	Tumor
chrM	10071	1099	RNR2	-5961	targeted sequencing	Péneau et al. 2021	33563643	Tumor
chrM	10896	329	RNR2	-7527	targeted sequencing	Péneau et al. 2021	33563643	Tumor
chrM	11262	747	RNR2	-7527	targeted sequencing	Péneau et al. 2021	33563643	Tumor
chrM	13083	1151	RNR2	-9807	targeted sequencing	Péneau et al. 2021	33563643	Tumor
chrM	14396	1197	RNR2	-11021	targeted sequencing	Péneau et al. 2021	33563643	Tumor
chrM	15338	3179	RNR2	-12014	targeted sequencing	Péneau et al. 2021	33563643	Tumor
chrM	15949	356	RNR2	-12014	targeted sequencing	Péneau et al. 2021	33563643	Tumor
chr3	80314566	2508	ROBO1	-497275	targeted sequencing	Péneau et al. 2021	33563643	Tumor
chr20	36703778	1830	RPRD1B	0	targeted sequencing	Péneau et al. 2021	33563643	Tumor
chr11	10209294	921	SBF2	0	targeted sequencing	Péneau et al. 2021	33563643	Tumor
chr19	4579574	3014	SEMA6B	-19655	targeted sequencing	Péneau et al. 2021	33563643	Tumor
chr12	132297832	2069	SFSWAP	-13549	targeted sequencing	Péneau et al. 2021	33563643	Tumor
chr9	91771881	2320	SHC3	0	targeted sequencing	Péneau et al. 2021	33563643	Tumor
chr20	1611924	2026	SIRPG	0	targeted sequencing	Péneau et al. 2021	33563643	Tumor
chr11	2939113	1137	SLC22A18	0	targeted sequencing	Péneau et al. 2021	33563643	Tumor
chr14	92785228	3196	SLC24A4	3519	targeted sequencing	Péneau et al. 2021	33563643	Tumor
chr19	19175879	584	SLC25A42	0	targeted sequencing	Péneau et al. 2021	33563643	Tumor
chr9	86975164	889	SLC28A3	0	targeted sequencing	Péneau et al. 2021	33563643	Tumor
chr17	71006608	1682	SLC39A11	0	targeted sequencing	Péneau et al. 2021	33563643	Tumor
chr4	72302635	2630	SLC4A4	0	targeted sequencing	Péneau et al. 2021	33563643	Tumor
chr5	101317397	1820	SLCO4C1	252176	targeted sequencing	Péneau et al. 2021	33563643	Tumor
chr13	37498829	483	SMAD9	-4420	targeted sequencing	Péneau et al. 2021	33563643	Tumor
chr6	11120493	313	SMIM13	0	targeted sequencing	Péneau et al. 2021	33563643	Tumor
chr1	99274969	3117	SNX7	-48913	targeted sequencing	Péneau et al. 2021	33563643	Tumor
chr16	1033435	2903	SOX8	0	targeted sequencing	Péneau et al. 2021	33563643	Tumor
chrX	140479154	313	SPANXA2-OT1	111689	targeted sequencing	Péneau et al. 2021	33563643	Tumor
chr15	44860836	2071	SPG11	0	targeted sequencing	Péneau et al. 2021	33563643	Tumor
chrX	154868783	2501	SPRY3	0	targeted sequencing	Péneau et al. 2021	33563643	Tumor
chr15	92909456	1752	ST8SIA2	27661	targeted sequencing	Péneau et al. 2021	33563643	Tumor
chr6	147390342	1869	STXBP5-AS1	0	targeted sequencing	Péneau et al. 2021	33563643	Tumor
chr1	172601146	1873	SUCO	-20171	targeted sequencing	Péneau et al. 2021	33563643	Tumor
chr6	44797310	2948	SUPT3H	0	targeted sequencing	Péneau et al. 2021	33563643	Tumor
chr20	24390166	3206	SYNDIG1	59669	targeted sequencing	Péneau et al. 2021	33563643	Tumor
chr14	64419946	2077	SYNE2	0	targeted sequencing	Péneau et al. 2021	33563643	Tumor
chr12	79574623	334	SYT1	0	targeted sequencing	Péneau et al. 2021	33563643	Tumor
chr11	78395808	2874	TENM4	0	targeted sequencing	Péneau et al. 2021	33563643	Tumor
chr9	103137468	350	TEX10	-22209	targeted sequencing	Péneau et al. 2021	33563643	Tumor
chr20	55277639	1699	TFAP2C	-63156	targeted sequencing	Péneau et al. 2021	33563643	Tumor
chr14	77807610	3150	TMED8	0	targeted sequencing	Péneau et al. 2021	33563643	Tumor
chr11	87143635	2009	TMEM135	-103759	targeted sequencing	Péneau et al. 2021	33563643	Tumor
chr20	48764907	2476	TMEM189|TMEM189-UBE2V1	0	targeted sequencing	Péneau et al. 2021	33563643	Tumor
chr10	81865663	351	TMEM254	-13356	targeted sequencing	Péneau et al. 2021	33563643	Tumor
chr12	83051483	1839	TMTC2	29451	targeted sequencing	Péneau et al. 2021	33563643	Tumor
chr7	144702121	325	TPK1	-168975	targeted sequencing	Péneau et al. 2021	33563643	Tumor
chr3	14058401	2804	TPRXL	0	targeted sequencing	Péneau et al. 2021	33563643	Tumor
chr9	77512071	1019	TRPM6	-9061	targeted sequencing	Péneau et al. 2021	33563643	Tumor
chrX	38485770	2040	TSPAN7	0	targeted sequencing	Péneau et al. 2021	33563643	Tumor
chr15	99682514	2604	TTC23	0	targeted sequencing	Péneau et al. 2021	33563643	Tumor
chr18	54134691	676	TXNL1	135362	targeted sequencing	Péneau et al. 2021	33563643	Tumor
chr11	88865393	1789	TYR	45443	targeted sequencing	Péneau et al. 2021	33563643	Tumor
chr17	74438955	558	UBE2O	0	targeted sequencing	Péneau et al. 2021	33563643	Tumor
chr15	54647664	2068	UNC13C	0	targeted sequencing	Péneau et al. 2021	33563643	Tumor
chr1	215850937	264	USH2A	0	targeted sequencing	Péneau et al. 2021	33563643	Tumor

Chromosome	Integration site in host genome	Integration site in virus genome	Gene (distance, bp)	Regions	Methods	Author	PMID	Sample
chrX	155173007	2243	VAMP7	0	targeted sequencing	Péneau et al. 2021	33563643	Tumor
chr2	37036735	1846	VIT	0	targeted sequencing	Péneau et al. 2021	33563643	Tumor
chr15	62285013	1000	VPS13C	0	targeted sequencing	Péneau et al. 2021	33563643	Tumor
chr11	124007271	1835	VWA5A	0	targeted sequencing	Péneau et al. 2021	33563643	Tumor
chr6	169817975	362	WDR27	39328	targeted sequencing	Péneau et al. 2021	33563643	Tumor
chr8	56150855	51	XKR4	0	targeted sequencing	Péneau et al. 2021	33563643	Tumor
chr16	17483668	1818	XYLT1	0	targeted sequencing	Péneau et al. 2021	33563643	Tumor
chr3	183443287	1203	YEATS2	0	targeted sequencing	Péneau et al. 2021	33563643	Tumor
chr16	29797267	2100	ZG16|KIF22	4362	targeted sequencing	Péneau et al. 2021	33563643	Tumor
chr16	49584579	299	ZNF423	0	targeted sequencing	Péneau et al. 2021	33563643	Tumor
chr19	58992775	110	ZNF446	0	targeted sequencing	Péneau et al. 2021	33563643	Tumor
chr18	74136678	1716	ZNF516	0	targeted sequencing	Péneau et al. 2021	33563643	Tumor
chr1	247206876	995	ZNF670|ZNF670-ZNF695	0	targeted sequencing	Péneau et al. 2021	33563643	Tumor
chr4	146822391	2739	ZNF827	0	targeted sequencing	Péneau et al. 2021	33563643	Tumor
chr20	57802975	2449	ZNF831	0	targeted sequencing	Péneau et al. 2021	33563643	Tumor
chr5	327485	1859	AHRR	0	targeted sequencing	Péneau et al. 2021	33563643	Tumor
chr5	1315047	778	CLPTM1L	2384	targeted sequencing	Péneau et al. 2021	33563643	Tumor
chr11	107957853	325	CUL5	0	targeted sequencing	Péneau et al. 2021	33563643	Tumor
chr11	107961625	774	CUL5	0	targeted sequencing	Péneau et al. 2021	33563643	Tumor
chr13	99748227	3063	DOCK9-AS2	-7170	targeted sequencing	Péneau et al. 2021	33563643	Tumor
chrX	150888347	1832	FATE1	0	targeted sequencing	Péneau et al. 2021	33563643	Tumor
chrX	150888367	1629	FATE1	0	targeted sequencing	Péneau et al. 2021	33563643	Tumor
chr8	138592653	1275	LOC101927915	-166705	targeted sequencing	Péneau et al. 2021	33563643	Tumor
chr1	37621788	684	MIR4255	5260	targeted sequencing	Péneau et al. 2021	33563643	Tumor
chr5	1680701	2392	MIR4277	28198	targeted sequencing	Péneau et al. 2021	33563643	Tumor
chr5	1750287	1568	MIR4277	-41303	targeted sequencing	Péneau et al. 2021	33563643	Tumor
chr4	61285119	2601	MIR548AG1	503218	targeted sequencing	Péneau et al. 2021	33563643	Tumor
chr11	133228542	905	OPCML	0	targeted sequencing	Péneau et al. 2021	33563643	Tumor
chr4	120156246	2797	USP53	0	targeted sequencing	Péneau et al. 2021	33563643	Tumor
chr1	197788456	1255	DENND1B	-43712	targeted sequencing	Péneau et al. 2021	33563643	Tumor
chr8	50318682	1821	LOC100507464	89935	targeted sequencing	Péneau et al. 2021	33563643	Tumor
chr8	50318688	1644	LOC100507464	89935	targeted sequencing	Péneau et al. 2021	33563643	Tumor
chr20	48659108	1808	TRERNA1	-276	targeted sequencing	Péneau et al. 2021	33563643	Tumor
chr20	48659132	1891	TRERNA1	-276	targeted sequencing	Péneau et al. 2021	33563643	Tumor
chr14	60921793	2221	C14orf39	0	targeted sequencing	Péneau et al. 2021	33563643	Tumor
chr8	87784081	1616	CNGB3	-28178	targeted sequencing	Péneau et al. 2021	33563643	Tumor
chr3	130118675	424	COL6A5	0	targeted sequencing	Péneau et al. 2021	33563643	Tumor
chr1	16426361	1628	EPHA2	24471	targeted sequencing	Péneau et al. 2021	33563643	Tumor
chr4	161351217	673	FSTL5	953173	targeted sequencing	Péneau et al. 2021	33563643	Tumor
chr4	161351308	1842	FSTL5	953173	targeted sequencing	Péneau et al. 2021	33563643	Tumor
chr19	27738470	2912	LINC00662	542830	targeted sequencing	Péneau et al. 2021	33563643	Tumor
chr19	27738518	2462	LINC00662	542830	targeted sequencing	Péneau et al. 2021	33563643	Tumor
chr9	66833432	647	LOC101928381	89430	targeted sequencing	Péneau et al. 2021	33563643	Tumor
chr10	42388414	1826	LOC441666	438498	targeted sequencing	Péneau et al. 2021	33563643	Tumor
chr10	42395975	1986	LOC441666	430292	targeted sequencing	Péneau et al. 2021	33563643	Tumor
chr4	126511420	1172	MIR2054	-82958	targeted sequencing	Péneau et al. 2021	33563643	Tumor
chr21	20026266	1793	MIR548XHG	0	targeted sequencing	Péneau et al. 2021	33563643	Tumor
chr21	20114374	1825	MIR548XHG	0	targeted sequencing	Péneau et al. 2021	33563643	Tumor
chr17	22261575	1765	MTRNR2L1	-237584	targeted sequencing	Péneau et al. 2021	33563643	Tumor
chr13	29769631	862	MTUS2	0	targeted sequencing	Péneau et al. 2021	33563643	Tumor
chr13	29769643	2708	MTUS2	0	targeted sequencing	Péneau et al. 2021	33563643	Tumor
chr5	51751407	1821	PELO	332367	targeted sequencing	Péneau et al. 2021	33563643	Tumor
chr5	51752688	1672	PELO	332367	targeted sequencing	Péneau et al. 2021	33563643	Tumor
chr5	100101444	1242	ST8SIA4	41195	targeted sequencing	Péneau et al. 2021	33563643	Tumor
chr21	17003059	1239	USP25	99186	targeted sequencing	Péneau et al. 2021	33563643	Tumor
chrX	155260452	1934	DDX11L16	-2502	targeted sequencing	Péneau et al. 2021	33563643	Tumor
chr9	10366	1574	DDX11L5	1578	targeted sequencing	Péneau et al. 2021	33563643	Tumor
chr3	128673847	2858	KIAA1257	15743	targeted sequencing	Péneau et al. 2021	33563643	Tumor
chr12	48032389	930	RPAP3	22473	targeted sequencing	Péneau et al. 2021	33563643	Tumor
chr12	48032408	175	RPAP3	22473	targeted sequencing	Péneau et al. 2021	33563643	Tumor
chr5	1295136	1461	TERT	0	targeted sequencing	Péneau et al. 2021	33563643	Tumor
chr11	109420429	1309	C11orf87	-120536	targeted sequencing	Péneau et al. 2021	33563643	Tumor
chr2	26381626	780	GAREM2	14334	targeted sequencing	Péneau et al. 2021	33563643	Tumor
chr2	121348471	2947	LINC01101	-124343	targeted sequencing	Péneau et al. 2021	33563643	Tumor
chr5	38875322	2142	OSMR	0	targeted sequencing	Péneau et al. 2021	33563643	Tumor
chr10	121668239	429	SEC23IP	0	targeted sequencing	Péneau et al. 2021	33563643	Tumor
chr17	18627700	1827	TRIM16L	0	targeted sequencing	Péneau et al. 2021	33563643	Tumor
chr17	18627701	1819	TRIM16L	0	targeted sequencing	Péneau et al. 2021	33563643	Tumor
chr10	29156866	3024	C10orf126	0	targeted sequencing	Péneau et al. 2021	33563643	Tumor
chr8	96323328	2570	C8orf37-AS1	0	targeted sequencing	Péneau et al. 2021	33563643	Tumor
chr16	76369558	1731	CNTNAP4	0	targeted sequencing	Péneau et al. 2021	33563643	Tumor
chr16	76397779	1834	CNTNAP4	0	targeted sequencing	Péneau et al. 2021	33563643	Tumor
chr2	164566904	1017	FIGN	0	targeted sequencing	Péneau et al. 2021	33563643	Tumor
chr1	193818874	92	LINC01031	-483791	targeted sequencing	Péneau et al. 2021	33563643	Tumor
chr19	32475920	346	LINC01533	40848	targeted sequencing	Péneau et al. 2021	33563643	Tumor
chr15	76472555	1801	LOC101929439|TMEM266	0	targeted sequencing	Péneau et al. 2021	33563643	Tumor
chr15	76472573	2250	LOC101929439|TMEM266	0	targeted sequencing	Péneau et al. 2021	33563643	Tumor
chr17	78434070	2732	NPTX1	6563	targeted sequencing	Péneau et al. 2021	33563643	Tumor
chr1	205683641	1795	NUCKS1	0	targeted sequencing	Péneau et al. 2021	33563643	Tumor
chr1	205683641	2010	NUCKS1	0	targeted sequencing	Péneau et al. 2021	33563643	Tumor
chr8	107205391	1828	OXR1	77015	targeted sequencing	Péneau et al. 2021	33563643	Tumor
chr8	107476801	1599	OXR1	0	targeted sequencing	Péneau et al. 2021	33563643	Tumor
chr5	10629	1744	PLEKHG4B	128948	targeted sequencing	Péneau et al. 2021	33563643	Tumor
chr7	157626096	1828	PTPRN2	0	targeted sequencing	Péneau et al. 2021	33563643	Tumor
chr7	157629922	1801	PTPRN2	0	targeted sequencing	Péneau et al. 2021	33563643	Tumor
chr12	118870065	155	SUDS3	-13863	targeted sequencing	Péneau et al. 2021	33563643	Tumor
chr5	1295380	1807	TERT	0	targeted sequencing	Péneau et al. 2021	33563643	Tumor
chr5	1295403	2216	TERT	0	targeted sequencing	Péneau et al. 2021	33563643	Tumor
chr17	57492694	1833	YPEL2	-13599	targeted sequencing	Péneau et al. 2021	33563643	Tumor
chr2	201728550	1159	CLK1	0	targeted sequencing	Péneau et al. 2021	33563643	Tumor
chr13	105470103	948	DAOA-AS1	640517	targeted sequencing	Péneau et al. 2021	33563643	Tumor
chr4	191044276	2080	DBET	-54861	targeted sequencing	Péneau et al. 2021	33563643	Tumor
chr20	53644368	1776	DOK5	-376658	targeted sequencing	Péneau et al. 2021	33563643	Tumor
chr18	28285109	3015	DSC3	284222	targeted sequencing	Péneau et al. 2021	33563643	Tumor

Chromosome	Integration site in host genome	Integration site in virus genome	Gene (distance, bp)	Regions	Methods	Author	PMID	Sample
chr3	55988656	1420	ERC2	0	targeted sequencing	Péneau et al. 2021	33563643	Tumor
chr4	170842052	2305	LOC100506085	0	targeted sequencing	Péneau et al. 2021	33563643	Tumor
chr17	17356935	1781	MED9	22654	targeted sequencing	Péneau et al. 2021	33563643	Tumor
chr9	20581024	2746	MLLT3	0	targeted sequencing	Péneau et al. 2021	33563643	Tumor
chr6	154185114	29	OPRM1	146517	targeted sequencing	Péneau et al. 2021	33563643	Tumor
chrM	3707	1192	RNR2	-477	targeted sequencing	Péneau et al. 2021	33563643	Tumor
chr12	21465905	3127	SLCO1A2	0	targeted sequencing	Péneau et al. 2021	33563643	Tumor
chrUn_gl000218	138355	1862	LOC100233156	-40747	targeted sequencing	Péneau et al. 2021	33563643	Tumor
chr5	77221141	1828	LOC101929154	0	targeted sequencing	Péneau et al. 2021	33563643	Tumor
chr18	76520013	2408	SALL3	220262	targeted sequencing	Péneau et al. 2021	33563643	Tumor
chr4	68627810	1818	GNRHR	-5398	targeted sequencing	Péneau et al. 2021	33563643	Tumor
chr4	68627811	855	GNRHR	-5398	targeted sequencing	Péneau et al. 2021	33563643	Tumor
chr4	44573741	2651	YIPF7	50404	targeted sequencing	Péneau et al. 2021	33563643	Tumor
chr10	96505980	1779	CYP2C18	-9514	targeted sequencing	Péneau et al. 2021	33563643	Tumor
chr10	96505982	1824	CYP2C18	-9514	targeted sequencing	Péneau et al. 2021	33563643	Tumor
chr10	42599616	NA	LOC441666	227495	targeted sequencing	Péneau et al. 2021	33563643	Tumor
chr12	72165740	2713	RAB21	0	targeted sequencing	Péneau et al. 2021	33563643	Tumor
chr12	72165796	2240	RAB21	0	targeted sequencing	Péneau et al. 2021	33563643	Tumor
chr4	74270154	1827	ALB	0	targeted sequencing	Péneau et al. 2021	33563643	Tumor
chr4	74270171	2330	ALB	0	targeted sequencing	Péneau et al. 2021	33563643	Tumor
chr5	10827	2176	PLEKHG4B	129443	targeted sequencing	Péneau et al. 2021	33563643	Tumor
chr10	27526535	103	ACBD5	0	targeted sequencing	Péneau et al. 2021	33563643	Tumor
chr8	108130194	1824	ANGPT1	131056	targeted sequencing	Péneau et al. 2021	33563643	Tumor
chr8	108137339	540	ANGPT1	124371	targeted sequencing	Péneau et al. 2021	33563643	Tumor
chr9	33533102	2627	ANKRD18B	0	targeted sequencing	Péneau et al. 2021	33563643	Tumor
chr11	26493035	2473	ANO3	0	targeted sequencing	Péneau et al. 2021	33563643	Tumor
chr17	34001164	696	AP2B1	0	targeted sequencing	Péneau et al. 2021	33563643	Tumor
chr20	57031199	574	APCDD1L	2961	targeted sequencing	Péneau et al. 2021	33563643	Tumor
chr4	79705343	1459	BMP2K	0	targeted sequencing	Péneau et al. 2021	33563643	Tumor
chr14	96478561	458	C14orf132	26961	targeted sequencing	Péneau et al. 2021	33563643	Tumor
chr17	69107735	699	CASC17	0	targeted sequencing	Péneau et al. 2021	33563643	Tumor
chr8	95171838	1320	CDH17	0	targeted sequencing	Péneau et al. 2021	33563643	Tumor
chr4	40381121	2674	CHRNA9	-23887	targeted sequencing	Péneau et al. 2021	33563643	Tumor
chr13	20894558	2138	CRYL1	83248	targeted sequencing	Péneau et al. 2021	33563643	Tumor
chr10	126952226	1501	CTBP2	-102475	targeted sequencing	Péneau et al. 2021	33563643	Tumor
chr19	15696007	414	CYP4F8	29868	targeted sequencing	Péneau et al. 2021	33563643	Tumor
chr8	120951948	1739	DEPTOR	0	targeted sequencing	Péneau et al. 2021	33563643	Tumor
chr18	67267709	2614	DOK6	0	targeted sequencing	Péneau et al. 2021	33563643	Tumor
chr6	93189359	646	EPHA7	760381	targeted sequencing	Péneau et al. 2021	33563643	Tumor
chr5	128019419	1758	FBN2	-145684	targeted sequencing	Péneau et al. 2021	33563643	Tumor
chr15	26757328	851	GABRB3	31366	targeted sequencing	Péneau et al. 2021	33563643	Tumor
chr8	75266746	1664	GDAP1	0	targeted sequencing	Péneau et al. 2021	33563643	Tumor
chr10	113635451	1960	GPAM	274027	targeted sequencing	Péneau et al. 2021	33563643	Tumor
chr7	74518013	2351	GTF2IRD2|GTF2IRD2B	0	targeted sequencing	Péneau et al. 2021	33563643	Tumor
chr8	43092981	2834	HGSNAT	-34905	targeted sequencing	Péneau et al. 2021	33563643	Tumor
chr19	34772747	565	KIAA0355	0	targeted sequencing	Péneau et al. 2021	33563643	Tumor
chr1	187957466	460	LINC01037	-511112	targeted sequencing	Péneau et al. 2021	33563643	Tumor
chrX	116088009	2901	LOC100126447	-136181	targeted sequencing	Péneau et al. 2021	33563643	Tumor
chr4	34889710	526	LOC101928622	-848195	targeted sequencing	Péneau et al. 2021	33563643	Tumor
chr1	546906	1535	LOC101928626	15751	targeted sequencing	Péneau et al. 2021	33563643	Tumor
chr12	43191866	1328	LOC105369739	-80945	targeted sequencing	Péneau et al. 2021	33563643	Tumor
chr14	87689914	2380	LOC283585	-300241	targeted sequencing	Péneau et al. 2021	33563643	Tumor
chr8	105629883	1808	LRP12	-28236	targeted sequencing	Péneau et al. 2021	33563643	Tumor
chr8	125591051	1493	MTSS1	0	targeted sequencing	Péneau et al. 2021	33563643	Tumor
chr8	1978767	2523	MYOM2	13683	targeted sequencing	Péneau et al. 2021	33563643	Tumor
chr8	1978787	1896	MYOM2	13683	targeted sequencing	Péneau et al. 2021	33563643	Tumor
chr11	119480378	382	NECTIN1	28430	targeted sequencing	Péneau et al. 2021	33563643	Tumor
chr9	33369841	2579	NFX1	0	targeted sequencing	Péneau et al. 2021	33563643	Tumor
chr8	110293397	986	NUDCD1	0	targeted sequencing	Péneau et al. 2021	33563643	Tumor
chr10	36469342	2895	PCAT5	-379311	targeted sequencing	Péneau et al. 2021	33563643	Tumor
chr2	101150364	780	PDCL3	29054	targeted sequencing	Péneau et al. 2021	33563643	Tumor
chr1	56597667	1709	PLPP3	362752	targeted sequencing	Péneau et al. 2021	33563643	Tumor
chr5	145949839	1805	PPP2R2B	19228	targeted sequencing	Péneau et al. 2021	33563643	Tumor
chr8	85502675	1136	RALYL	0	targeted sequencing	Péneau et al. 2021	33563643	Tumor
chr3	18631444	759	SATB1-AS1	-59613	targeted sequencing	Péneau et al. 2021	33563643	Tumor
chr8	53064358	3103	ST18	0	targeted sequencing	Péneau et al. 2021	33563643	Tumor
chr5	1295366	1819	TERT	0	targeted sequencing	Péneau et al. 2021	33563643	Tumor
chr5	1295368	447	TERT	0	targeted sequencing	Péneau et al. 2021	33563643	Tumor
chr8	110132000	1090	TRHR	0	targeted sequencing	Péneau et al. 2021	33563643	Tumor
chr6	27360767	608	ZNF391	0	targeted sequencing	Péneau et al. 2021	33563643	Tumor
chr6	27360795	858	ZNF391	0	targeted sequencing	Péneau et al. 2021	33563643	Tumor
chr7	66999076	1832	LINC01372	-193964	targeted sequencing	Péneau et al. 2021	33563643	Tumor
chr8	86966821	1833	PSKH2	91771	targeted sequencing	Péneau et al. 2021	33563643	Tumor
chr17	9799849	1604	RCVRN	595	targeted sequencing	Péneau et al. 2021	33563643	Tumor
chr6	69756230	1842	ADGRB3	0	targeted sequencing	Péneau et al. 2021	33563643	Tumor
chr8	47978187	1810	LOC100287846	122169	targeted sequencing	Péneau et al. 2021	33563643	Tumor
chr15	66377389	723	MEGF11	0	targeted sequencing	Péneau et al. 2021	33563643	Tumor
chr2	5300828	1207	LINC01248	473445	targeted sequencing	Péneau et al. 2021	33563643	Tumor
chr5	1295138	1968	TERT	0	targeted sequencing	Péneau et al. 2021	33563643	Tumor
chr21	44515024	2095	U2AF1|U2AF1L5	0	targeted sequencing	Péneau et al. 2021	33563643	Tumor
chr5	110607342	2777	CAMK4	0	targeted sequencing	Péneau et al. 2021	33563643	Tumor
chr2	204147533	1432	CYP20A1	0	targeted sequencing	Péneau et al. 2021	33563643	Tumor
chr13	107925566	1778	FAM155A	0	targeted sequencing	Péneau et al. 2021	33563643	Tumor
chr4	1677727	2553	FAM53A	0	targeted sequencing	Péneau et al. 2021	33563643	Tumor
chr4	162810045	2706	FSTL5	0	targeted sequencing	Péneau et al. 2021	33563643	Tumor
chr2	171820153	1659	GORASP2	0	targeted sequencing	Péneau et al. 2021	33563643	Tumor
chr2	171820160	1771	GORASP2	0	targeted sequencing	Péneau et al. 2021	33563643	Tumor
chr8	130793203	2573	GSDMC	0	targeted sequencing	Péneau et al. 2021	33563643	Tumor
chr12	70596568	2003	LINC01481	19402	targeted sequencing	Péneau et al. 2021	33563643	Tumor
chr2	240732329	1074	LOC150935	-9543	targeted sequencing	Péneau et al. 2021	33563643	Tumor
chr1	36930099	2695	MRPS15	-59	targeted sequencing	Péneau et al. 2021	33563643	Tumor
chr3	172413930	2424	NCEH1	0	targeted sequencing	Péneau et al. 2021	33563643	Tumor
chr4	111514886	2328	PITX2	23551	targeted sequencing	Péneau et al. 2021	33563643	Tumor
chr4	6358249	1256	PPP2R2C	0	targeted sequencing	Péneau et al. 2021	33563643	Tumor

Chromosome	Integration site in host genome	Integration site in virus genome	Gene (distance, bp)	Regions	Methods	Author	PMID	Sample
chr14	29782571	1826	PRKD1	263115	targeted sequencing	Péneau et al. 2021	33563643	Tumor
chr2	227778896	245	RHBDD1	0	targeted sequencing	Péneau et al. 2021	33563643	Tumor
chr17	20196747	1376	SPECC1	0	targeted sequencing	Péneau et al. 2021	33563643	Tumor
chr5	1295172	1817	TERT	0	targeted sequencing	Péneau et al. 2021	33563643	Tumor
chr5	1295187	285	TERT	0	targeted sequencing	Péneau et al. 2021	33563643	Tumor
chr1	28799815	2135	PHACTR4	0	targeted sequencing	Péneau et al. 2021	33563643	Tumor
chr1	28799834	1650	PHACTR4	0	targeted sequencing	Péneau et al. 2021	33563643	Tumor
chr5	1295177	1804	TERT	0	targeted sequencing	Péneau et al. 2021	33563643	Tumor
chr5	1295197	684	TERT	0	targeted sequencing	Péneau et al. 2021	33563643	Tumor
chr9	128450793	1099	MAPKAP1	0	targeted sequencing	Péneau et al. 2021	33563643	Tumor
chr9	20472074	489	MLLT3	0	targeted sequencing	Péneau et al. 2021	33563643	Tumor
chr8	97262911	1531	MTERF3	0	targeted sequencing	Péneau et al. 2021	33563643	Tumor
chr8	97263298	373	MTERF3	0	targeted sequencing	Péneau et al. 2021	33563643	Tumor
chr10	6433836	916	PRKCQ	2223	targeted sequencing	Péneau et al. 2021	33563643	Tumor
chr21	48118820	NA	PRMT2	-33713	targeted sequencing	Péneau et al. 2021	33563643	Tumor
chr4	191043871	852	DBET	-54274	targeted sequencing	Péneau et al. 2021	33563643	Tumor
chr3	197900324	2245	FAM157A	0	targeted sequencing	Péneau et al. 2021	33563643	Tumor
chr1	236807463	572	HEATR1	-39622	targeted sequencing	Péneau et al. 2021	33563643	Tumor
chr13	90202952	350	LINC00353	0	targeted sequencing	Péneau et al. 2021	33563643	Tumor
chr18	1856160	3141	LINC00470	-495840	targeted sequencing	Péneau et al. 2021	33563643	Tumor
chr12	94733	2201	LOC100288778	-3377	targeted sequencing	Péneau et al. 2021	33563643	Tumor
chr9	110133559	817	RAD23B	-38874	targeted sequencing	Péneau et al. 2021	33563643	Tumor
chr5	1300713	377	TERT	-5551	targeted sequencing	Péneau et al. 2021	33563643	Tumor
chrUn_gl000226	984	2159	.	-1	targeted sequencing	Péneau et al. 2021	33563643	Tumor
chr11	116705214	1819	APOA1	1022	targeted sequencing	Péneau et al. 2021	33563643	Tumor
chr11	116705234	2316	APOA1	1022	targeted sequencing	Péneau et al. 2021	33563643	Tumor
chr1	181484045	1816	CACNA1E	0	targeted sequencing	Péneau et al. 2021	33563643	Tumor
chr1	181484045	1827	CACNA1E	0	targeted sequencing	Péneau et al. 2021	33563643	Tumor
chr4	165486751	1820	MARCH1	-181707	targeted sequencing	Péneau et al. 2021	33563643	Tumor
chr8	27926748	1607	NUGGC	0	targeted sequencing	Péneau et al. 2021	33563643	Tumor
chrUn_gl000220	159672	2923	RNA5-8S5	-3368	targeted sequencing	Péneau et al. 2021	33563643	Tumor
chrUn_gl000220	159699	1801	RNA5-8S5	-3368	targeted sequencing	Péneau et al. 2021	33563643	Tumor
chr5	1295143	1813	TERT	0	targeted sequencing	Péneau et al. 2021	33563643	Tumor
chr17	71268967	2089	CDC42EP4	10078	targeted sequencing	Péneau et al. 2021	33563643	Tumor
chr4	191044049	2964	DBET	-54658	targeted sequencing	Péneau et al. 2021	33563643	Tumor
chr3	197900736	2840	FAM157A	0	targeted sequencing	Péneau et al. 2021	33563643	Tumor
chr17	14774078	1814	LOC101928475	144044	targeted sequencing	Péneau et al. 2021	33563643	Tumor
chr4	106872929	1755	NPNT	0	targeted sequencing	Péneau et al. 2021	33563643	Tumor
chr1	249240352	2482	PGBD2	-26560	targeted sequencing	Péneau et al. 2021	33563643	Tumor
chr15	52098738	1842	TMOD2	0	targeted sequencing	Péneau et al. 2021	33563643	Tumor
chr15	52099088	2404	TMOD2	0	targeted sequencing	Péneau et al. 2021	33563643	Tumor
chrX	131946265	2903	HS6ST2	0	targeted sequencing	Péneau et al. 2021	33563643	Tumor
chr8	137390691	1464	KHDRBS3	-730721	targeted sequencing	Péneau et al. 2021	33563643	Tumor
chr6	16135434	2282	MYLIP	0	targeted sequencing	Péneau et al. 2021	33563643	Tumor
chr4	23705500	2154	PPARGC1A	87932	targeted sequencing	Péneau et al. 2021	33563643	Tumor
chr6	111627367	1653	REV3L	0	targeted sequencing	Péneau et al. 2021	33563643	Tumor
chr10	22671681	1834	SPAG6	0	targeted sequencing	Péneau et al. 2021	33563643	Tumor
chr10	22671691	1631	SPAG6	0	targeted sequencing	Péneau et al. 2021	33563643	Tumor
chr5	1295128	1819	TERT	0	targeted sequencing	Péneau et al. 2021	33563643	Tumor
chr5	1302055	2898	TERT|MIR4457	6667	targeted sequencing	Péneau et al. 2021	33563643	Tumor
chr4	10067	2245	ZNF595	42950	targeted sequencing	Péneau et al. 2021	33563643	Tumor
chr19	30306571	2563	CCNE1	0	targeted sequencing	Péneau et al. 2021	33563643	Tumor
chr19	30306577	255	CCNE1	0	targeted sequencing	Péneau et al. 2021	33563643	Tumor
chr6	63465765	1836	KHDRBS2	-468937	targeted sequencing	Péneau et al. 2021	33563643	Tumor
chr6	63465773	1904	KHDRBS2	-468937	targeted sequencing	Péneau et al. 2021	33563643	Tumor
chr19	3332475	1868	NFIC	26276	targeted sequencing	Péneau et al. 2021	33563643	Tumor
chr19	3385022	1820	NFIC	0	targeted sequencing	Péneau et al. 2021	33563643	Tumor
chr16	4962768	2048	PPL	0	targeted sequencing	Péneau et al. 2021	33563643	Tumor
chr16	4962803	58	PPL	0	targeted sequencing	Péneau et al. 2021	33563643	Tumor
chr5	1295173	1603	TERT	0	targeted sequencing	Péneau et al. 2021	33563643	Tumor
chr5	1295174	2854	TERT	0	targeted sequencing	Péneau et al. 2021	33563643	Tumor
chr1	150866263	1742	ARNT	-16832	targeted sequencing	Péneau et al. 2021	33563643	Tumor
chr1	150866263	1825	ARNT	-16832	targeted sequencing	Péneau et al. 2021	33563643	Tumor
chr7	153629480	1826	DPP6	0	targeted sequencing	Péneau et al. 2021	33563643	Tumor
chr9	118742885	1828	LINC00474	-55258	targeted sequencing	Péneau et al. 2021	33563643	Tumor
chr10	2326615	1799	LINC00701	15493	targeted sequencing	Péneau et al. 2021	33563643	Tumor
chr7	66821586	1821	LINC01372	-16574	targeted sequencing	Péneau et al. 2021	33563643	Tumor
chr3	61348	1733	LOC102723448	3281	targeted sequencing	Péneau et al. 2021	33563643	Tumor
chr3	61516	2193	LOC102723448	3281	targeted sequencing	Péneau et al. 2021	33563643	Tumor
chr4	8920519	1579	LOC650293	30432	targeted sequencing	Péneau et al. 2021	33563643	Tumor
chr4	8920550	1881	LOC650293	30432	targeted sequencing	Péneau et al. 2021	33563643	Tumor
chr2	185021367	1612	MIR548AE1	221916	targeted sequencing	Péneau et al. 2021	33563643	Tumor
chr2	185021367	1909	MIR548AE1	221916	targeted sequencing	Péneau et al. 2021	33563643	Tumor
chr19	4772902	1462	MIR7-3HG	-1	targeted sequencing	Péneau et al. 2021	33563643	Tumor
chr16	4519393	564	NMRAL1	0	targeted sequencing	Péneau et al. 2021	33563643	Tumor
chr1	248353636	733	OR2M2	-9305	targeted sequencing	Péneau et al. 2021	33563643	Tumor
chr4	96909149	2424	PDHA2	-146165	targeted sequencing	Péneau et al. 2021	33563643	Tumor
chr8	74235981	1839	RDH10|RDH10-AS1	0	targeted sequencing	Péneau et al. 2021	33563643	Tumor
chr11	64902841	1812	SYVN1	-697	targeted sequencing	Péneau et al. 2021	33563643	Tumor
chr11	64902848	1914	SYVN1	-697	targeted sequencing	Péneau et al. 2021	33563643	Tumor
chrUn_gl000224	162506	2221	LOC100505874	-51783	targeted sequencing	Péneau et al. 2021	33563643	Tumor
chrUn_gl000220	124160	2057	LOC100507412	0	targeted sequencing	Péneau et al. 2021	33563643	Tumor
chr1	60239686	2250	LOC101926944	0	targeted sequencing	Péneau et al. 2021	33563643	Tumor
chr16	52881345	2382	LOC105371267	188257	targeted sequencing	Péneau et al. 2021	33563643	Tumor
chr22	18929244	1568	PRODH	-5177	targeted sequencing	Péneau et al. 2021	33563643	Tumor
chr5	103721933	2893	RAB9BP1	712716	targeted sequencing	Péneau et al. 2021	33563643	Tumor
chr17	81175884	3058	RPL23AP87	0	targeted sequencing	Péneau et al. 2021	33563643	Tumor
chr2	92319802	2691	ACTR3BP2	-189306	targeted sequencing	Péneau et al. 2021	33563643	Tumor
chr20	44838602	444	CDH22	0	targeted sequencing	Péneau et al. 2021	33563643	Tumor
chrX	155260300	1232	DDX11L16	-2358	targeted sequencing	Péneau et al. 2021	33563643	Tumor
chr1	159733522	1060	DUSP23	16773	targeted sequencing	Péneau et al. 2021	33563643	Tumor
chrX	151190796	79	GABRE	-47459	targeted sequencing	Péneau et al. 2021	33563643	Tumor
chr1	62737757	342	KANK4	0	targeted sequencing	Péneau et al. 2021	33563643	Tumor
chr1	73947448	1971	LINC01360	-142598	targeted sequencing	Péneau et al. 2021	33563643	Tumor

Chromosome	Integration site in host genome	Integration site in virus genome	Gene (distance, bp)	Regions	Methods	Author	PMID	Sample
chr1	73949023	3101	LINC01360	-144463	targeted sequencing	Péneau et al. 2021	33563643	Tumor
chr22	27484303	382	LOC284898	-27690	targeted sequencing	Péneau et al. 2021	33563643	Tumor
chr17	22257664	2379	MTRNR2L1	-233673	targeted sequencing	Péneau et al. 2021	33563643	Tumor
chr17	22260043	2950	MTRNR2L1	-236052	targeted sequencing	Péneau et al. 2021	33563643	Tumor
chr17	22260947	1310	MTRNR2L1	-236956	targeted sequencing	Péneau et al. 2021	33563643	Tumor
chr1	230629006	2901	PGBD5	-67162	targeted sequencing	Péneau et al. 2021	33563643	Tumor
chr15	24078567	115	PWRN4	141727	targeted sequencing	Péneau et al. 2021	33563643	Tumor
chr5	115798624	620	SEMA6A|SEMA6A-AS1	0	targeted sequencing	Péneau et al. 2021	33563643	Tumor
chr11	121567767	546	SORL1	-63296	targeted sequencing	Péneau et al. 2021	33563643	Tumor
chr15	57440612	2267	TCF12	0	targeted sequencing	Péneau et al. 2021	33563643	Tumor
chr19	21963531	2572	ZNF100	-12942	targeted sequencing	Péneau et al. 2021	33563643	Tumor
chr1	68597150	2604	GNG12-AS1|WLS	0	targeted sequencing	Péneau et al. 2021	33563643	Tumor
chr1	224156272	684	GTF2IP20	0	targeted sequencing	Péneau et al. 2021	33563643	Tumor
chr8	97545571	NA	SDC2	0	targeted sequencing	Péneau et al. 2021	33563643	Tumor
chr5	1293251	1427	TERT	0	targeted sequencing	Péneau et al. 2021	33563643	Tumor
chr5	1295461	1826	TERT	0	targeted sequencing	Péneau et al. 2021	33563643	Tumor
chr22	38123171	786	TRIOBP	0	targeted sequencing	Péneau et al. 2021	33563643	Tumor
chr8	62258188	295	CLVS1	0	targeted sequencing	Péneau et al. 2021	33563643	Tumor
chr8	46842531	1817	LINC00293	909365	targeted sequencing	Péneau et al. 2021	33563643	Tumor
chr9	66852486	2957	LOC101928381	70376	targeted sequencing	Péneau et al. 2021	33563643	Tumor
chr13	65221520	2986	LOC102723968	-803260	targeted sequencing	Péneau et al. 2021	33563643	Tumor
chr13	65231237	1938	LOC102723968	-812463	targeted sequencing	Péneau et al. 2021	33563643	Tumor
chr2	174150646	2918	MLK7-AS1	-3882	targeted sequencing	Péneau et al. 2021	33563643	Tumor
chr8	89190883	1259	MMP16	0	targeted sequencing	Péneau et al. 2021	33563643	Tumor
chr5	1289797	2332	TERT	0	targeted sequencing	Péneau et al. 2021	33563643	Tumor
chr5	1296015	581	TERT	-157	targeted sequencing	Péneau et al. 2021	33563643	Tumor
chr17	17810274	1822	TOM1L2	0	targeted sequencing	Péneau et al. 2021	33563643	Tumor
chr22	39504999	1769	APOBEC3H	-4925	targeted sequencing	Péneau et al. 2021	33563643	Tumor
chr4	49639119	199	CWH43	-574839	targeted sequencing	Péneau et al. 2021	33563643	Tumor
chr4	118865949	NA	NDST3	89551	targeted sequencing	Péneau et al. 2021	33563643	Tumor
chr2	206963513	2644	INO80D	-12607	targeted sequencing	Péneau et al. 2021	33563643	Tumor
chr4	34548615	2332	LOC101928622	-506967	targeted sequencing	Péneau et al. 2021	33563643	Tumor
chr8	96567680	933	C8orf37-AS1	0	targeted sequencing	Péneau et al. 2021	33563643	Tumor
chr20	53283075	2414	DOK5	-15061	targeted sequencing	Péneau et al. 2021	33563643	Tumor
chr20	53283078	1758	DOK5	-15061	targeted sequencing	Péneau et al. 2021	33563643	Tumor
chr8	129787902	1881	LINC00824	-210591	targeted sequencing	Péneau et al. 2021	33563643	Tumor
chr8	129787963	1803	LINC00824	-210591	targeted sequencing	Péneau et al. 2021	33563643	Tumor
chr1	235176372	1729	LOC101927851	-75969	targeted sequencing	Péneau et al. 2021	33563643	Tumor
chr1	235176385	1829	LOC101927851	-75969	targeted sequencing	Péneau et al. 2021	33563643	Tumor
chr17	22258088	1546	MTRNR2L1	-234097	targeted sequencing	Péneau et al. 2021	33563643	Tumor
chr8	43826988	1683	POTEA	-608660	targeted sequencing	Péneau et al. 2021	33563643	Tumor
chr17	57873921	1667	VMP1	0	targeted sequencing	Péneau et al. 2021	33563643	Tumor
chr5	77526916	2759	AP3B1	0	targeted sequencing	Péneau et al. 2021	33563643	Tumor
chr3	56955111	2371	ARHGEF3	0	targeted sequencing	Péneau et al. 2021	33563643	Tumor
chr3	56958333	1483	ARHGEF3	0	targeted sequencing	Péneau et al. 2021	33563643	Tumor
chr4	122735865	1701	EXOSC9	0	targeted sequencing	Péneau et al. 2021	33563643	Tumor
chr4	122735873	1825	EXOSC9	0	targeted sequencing	Péneau et al. 2021	33563643	Tumor
chr8	95961375	216	TP53INP1	0	targeted sequencing	Péneau et al. 2021	33563643	Tumor
chr8	103334127	1334	UBR5	0	targeted sequencing	Péneau et al. 2021	33563643	Tumor
chrY	13833351	1831	GYG2P1	684564	targeted sequencing	Péneau et al. 2021	33563643	Tumor
chr4	180156993	1438	LINC01098	-1244484	targeted sequencing	Péneau et al. 2021	33563643	Tumor
chr5	120404136	2771	LOC102467226	253664	targeted sequencing	Péneau et al. 2021	33563643	Tumor
chr10	42597195	2795	LOC441666	229732	targeted sequencing	Péneau et al. 2021	33563643	Tumor
chr10	42599553	2283	LOC441666	227063	targeted sequencing	Péneau et al. 2021	33563643	Tumor
chr10	42599856	2068	LOC441666	227063	targeted sequencing	Péneau et al. 2021	33563643	Tumor
chr8	32197876	1821	NRG1	0	targeted sequencing	Péneau et al. 2021	33563643	Tumor
chr8	57104833	1264	PLAG1	0	targeted sequencing	Péneau et al. 2021	33563643	Tumor
chr5	1295178	1819	TERT	0	targeted sequencing	Péneau et al. 2021	33563643	Tumor
chr5	1295201	1990	TERT	0	targeted sequencing	Péneau et al. 2021	33563643	Tumor
chr1	184201883	2426	C1orf21	154084	targeted sequencing	Péneau et al. 2021	33563643	Tumor
chr7	135018995	2383	CNOT4	27337	targeted sequencing	Péneau et al. 2021	33563643	Tumor
chr2	165606277	2489	COBLL1	0	targeted sequencing	Péneau et al. 2021	33563643	Tumor
chrY	59362640	791	DDX11L16	-1738	targeted sequencing	Péneau et al. 2021	33563643	Tumor
chr14	44821843	2738	FSCB	151356	targeted sequencing	Péneau et al. 2021	33563643	Tumor
chr12	94733	2137	LOC100288778	-2990	targeted sequencing	Péneau et al. 2021	33563643	Tumor
chr10	42597119	1393	LOC441666	229670	targeted sequencing	Péneau et al. 2021	33563643	Tumor
chr6	136787832	2420	MAP7	0	targeted sequencing	Péneau et al. 2021	33563643	Tumor
chr16	61256963	2870	MIR4426	-167067	targeted sequencing	Péneau et al. 2021	33563643	Tumor
chr11	60140010	437	MS4A7	5948	targeted sequencing	Péneau et al. 2021	33563643	Tumor
chr6	57090214	2137	RAB23	-3102	targeted sequencing	Péneau et al. 2021	33563643	Tumor
chr6	45049916	2743	SUPT3H	0	targeted sequencing	Péneau et al. 2021	33563643	Tumor
chr9	136817527	1541	VAV2	0	targeted sequencing	Péneau et al. 2021	33563643	Tumor
chr16	46432682	823	ANKRD26P1	70404	targeted sequencing	Péneau et al. 2021	33563643	Tumor
chr10	95459780	1824	FRA10AC1	0	targeted sequencing	Péneau et al. 2021	33563643	Tumor
chr10	95459801	2397	FRA10AC1	0	targeted sequencing	Péneau et al. 2021	33563643	Tumor
chr4	162825323	1756	FSTL5	0	targeted sequencing	Péneau et al. 2021	33563643	Tumor
chr4	162825380	2468	FSTL5	0	targeted sequencing	Péneau et al. 2021	33563643	Tumor
chr7	74144895	1857	GTF2I	0	targeted sequencing	Péneau et al. 2021	33563643	Tumor
chr7	74144923	1525	GTF2I	0	targeted sequencing	Péneau et al. 2021	33563643	Tumor
chr8	43094886	2223	HGSNAT	-36788	targeted sequencing	Péneau et al. 2021	33563643	Tumor
chr12	71935113	1792	LGR5	0	targeted sequencing	Péneau et al. 2021	33563643	Tumor
chrUn_gl000220	115719	2963	LOC100507412|RNA45S5	0	targeted sequencing	Péneau et al. 2021	33563643	Tumor
chr8	31541222	188	NRG1	0	targeted sequencing	Péneau et al. 2021	33563643	Tumor
chr8	31541227	1824	NRG1	0	targeted sequencing	Péneau et al. 2021	33563643	Tumor
chr11	51590339	155	OR4C46	-74128	targeted sequencing	Péneau et al. 2021	33563643	Tumor
chr13	114947561	657	RASA3	-49199	targeted sequencing	Péneau et al. 2021	33563643	Tumor
chr5	1293295	1550	TERT	0	targeted sequencing	Péneau et al. 2021	33563643	Tumor
chr5	1293321	604	TERT	0	targeted sequencing	Péneau et al. 2021	33563643	Tumor
chr3	147945487	2270	LOC100507461	-139458	targeted sequencing	Péneau et al. 2021	33563643	Tumor
chr3	147946221	1817	LOC100507461	-140405	targeted sequencing	Péneau et al. 2021	33563643	Tumor
chr2	90460035	2922	MIR4436A	-1348067	targeted sequencing	Péneau et al. 2021	33563643	Tumor
chr5	128689437	2836	MIR4460	43318	targeted sequencing	Péneau et al. 2021	33563643	Tumor
chr1	40206372	1790	PPIE	0	targeted sequencing	Péneau et al. 2021	33563643	Tumor
chr1	40206400	808	PPIE	0	targeted sequencing	Péneau et al. 2021	33563643	Tumor

Chromosome	Integration site in host genome	Integration site in virus genome	Gene (distance, bp)	Regions	Methods	Author	PMID	Sample
chr21	34074990	899	SYNJ1	0	targeted sequencing	Péneau et al. 2021	33563643	Tumor
chr7	61968980	1103	ZNF733P	781138	targeted sequencing	Péneau et al. 2021	33563643	Tumor
chr7	61969013	2120	ZNF733P	781138	targeted sequencing	Péneau et al. 2021	33563643	Tumor
chr20	56613342	95	C20orf85	112507	targeted sequencing	Péneau et al. 2021	33563643	Tumor
chr9	69266804	1279	CBWD5	-4058	targeted sequencing	Péneau et al. 2021	33563643	Tumor
chr9	11038	3044	DDX11L5	814	targeted sequencing	Péneau et al. 2021	33563643	Tumor
chr10	46930602	279	FAM35BP	0	targeted sequencing	Péneau et al. 2021	33563643	Tumor
chr10	46930618	1820	FAM35BP	0	targeted sequencing	Péneau et al. 2021	33563643	Tumor
chr9	74736010	755	GDA	0	targeted sequencing	Péneau et al. 2021	33563643	Tumor
chrY	13489164	1476	GYG2P1	1028751	targeted sequencing	Péneau et al. 2021	33563643	Tumor
chr3	177669480	38	KCCAT211	-52119	targeted sequencing	Péneau et al. 2021	33563643	Tumor
chr3	178428955	1536	KCNMB2|KCNMB2-AS1	0	targeted sequencing	Péneau et al. 2021	33563643	Tumor
chr19	36213510	1263	KMT2B	0	targeted sequencing	Péneau et al. 2021	33563643	Tumor
chr13	91663602	2357	LINC00380	75262	targeted sequencing	Péneau et al. 2021	33563643	Tumor
chr12	65877912	582	LOC100507065	0	targeted sequencing	Péneau et al. 2021	33563643	Tumor
chrUn_gl000220	113107	1848	LOC100507412|RNA45S5	0	targeted sequencing	Péneau et al. 2021	33563643	Tumor
chr7	119497704	2428	LVCAT5	0	targeted sequencing	Péneau et al. 2021	33563643	Tumor
chr11	86303263	493	ME3	0	targeted sequencing	Péneau et al. 2021	33563643	Tumor
chr20	43576970	491	PABPC1L|TOMM34	0	targeted sequencing	Péneau et al. 2021	33563643	Tumor
chr8	48869737	835	PRKDC	0	targeted sequencing	Péneau et al. 2021	33563643	Tumor
chr21	10731633	1826	TPTE	173641	targeted sequencing	Péneau et al. 2021	33563643	Tumor
chr16	88686997	815	ZC3H18	0	targeted sequencing	Péneau et al. 2021	33563643	Tumor
chr11	26391924	1358	ANO3	0	targeted sequencing	Péneau et al. 2021	33563643	Tumor
chr2	117779287	2987	DDX18	792362	targeted sequencing	Péneau et al. 2021	33563643	Tumor
chr2	118259889	1817	DDX18	312366	targeted sequencing	Péneau et al. 2021	33563643	Tumor
chr11	32590303	2445	EIF3M	15010	targeted sequencing	Péneau et al. 2021	33563643	Tumor
chrY	13867052	2507	GYG2P1	650566	targeted sequencing	Péneau et al. 2021	33563643	Tumor
chr13	69477397	1937	LINC00550	-17327	targeted sequencing	Péneau et al. 2021	33563643	Tumor
chr13	69477409	461	LINC00550	-17327	targeted sequencing	Péneau et al. 2021	33563643	Tumor
chr12	95513	3091	LOC100288778	-4173	targeted sequencing	Péneau et al. 2021	33563643	Tumor
chr10	42529420	1914	LOC441666	296934	targeted sequencing	Péneau et al. 2021	33563643	Tumor
chr5	1298884	1825	TERT	-3050	targeted sequencing	Péneau et al. 2021	33563643	Tumor
chr10	67981735	1761	CTNNA3	0	targeted sequencing	Péneau et al. 2021	33563643	Tumor
chr10	96750155	1787	CYP2C9	-904	targeted sequencing	Péneau et al. 2021	33563643	Tumor
chr17	20630595	815	LOC100287072	0	targeted sequencing	Péneau et al. 2021	33563643	Tumor
chr5	50355452	1870	LOC100287592	-89431	targeted sequencing	Péneau et al. 2021	33563643	Tumor
chr13	55638699	1164	MIR5007	109701	targeted sequencing	Péneau et al. 2021	33563643	Tumor
chr8	115465570	1542	TRPS1	954624	targeted sequencing	Péneau et al. 2021	33563643	Tumor
chr13	41242982	444	FOXO1	-2040	targeted sequencing	Péneau et al. 2021	33563643	Tumor
chrUn_gl000220	24607	366	LOC100507412	72522	targeted sequencing	Péneau et al. 2021	33563643	Tumor
chr17	16196124	2511	PIGL	0	targeted sequencing	Péneau et al. 2021	33563643	Tumor
chr14	69069546	812	RAD51B	0	targeted sequencing	Péneau et al. 2021	33563643	Tumor
chr14	69069548	1806	RAD51B	0	targeted sequencing	Péneau et al. 2021	33563643	Tumor
chr16	29333329	637	SNX29P2	0	targeted sequencing	Péneau et al. 2021	33563643	Tumor
chr18	54559262	1587	WDR7	0	targeted sequencing	Péneau et al. 2021	33563643	Tumor
chr5	1307794	164	MIR4457	1216	targeted sequencing	Péneau et al. 2021	33563643	Tumor
chr7	72363273	2404	POM121	0	targeted sequencing	Péneau et al. 2021	33563643	Tumor
chr7	72363280	1001	POM121	0	targeted sequencing	Péneau et al. 2021	33563643	Tumor
chr5	1295885	1585	TERT	0	targeted sequencing	Péneau et al. 2021	33563643	Tumor
chr3	64974791	2811	ADAMTS9-AS2	0	targeted sequencing	Péneau et al. 2021	33563643	Tumor
chr7	968268	352	ADAP1	0	targeted sequencing	Péneau et al. 2021	33563643	Tumor
chr7	968273	1826	ADAP1	0	targeted sequencing	Péneau et al. 2021	33563643	Tumor
chr10	66559195	317	ANXA2P3	26090	targeted sequencing	Péneau et al. 2021	33563643	Tumor
chr4	106447758	1551	ARHGEF38	25665	targeted sequencing	Péneau et al. 2021	33563643	Tumor
chr2	237185497	1820	ASB18	-12249	targeted sequencing	Péneau et al. 2021	33563643	Tumor
chr17	31334131	1862	ASIC2	5975	targeted sequencing	Péneau et al. 2021	33563643	Tumor
chr9	119456122	290	ASTN2|TRIM32	0	targeted sequencing	Péneau et al. 2021	33563643	Tumor
chr2	44889330	2000	CAMKMT	0	targeted sequencing	Péneau et al. 2021	33563643	Tumor
chr22	29120774	176	CHEK2	0	targeted sequencing	Péneau et al. 2021	33563643	Tumor
chr19	50220298	2435	CPT1C	-3136	targeted sequencing	Péneau et al. 2021	33563643	Tumor
chr6	12312610	1869	EDN1	-15025	targeted sequencing	Péneau et al. 2021	33563643	Tumor
chr2	222716620	311	EPHA4	-277538	targeted sequencing	Péneau et al. 2021	33563643	Tumor
chr8	43095164	147	HGSNAT	-37194	targeted sequencing	Péneau et al. 2021	33563643	Tumor
chr16	33974751	403	LINC00273	-12119	targeted sequencing	Péneau et al. 2021	33563643	Tumor
chr13	48046274	1660	LINC00562	458014	targeted sequencing	Péneau et al. 2021	33563643	Tumor
chr12	103585070	1729	LOC101929058	-22984	targeted sequencing	Péneau et al. 2021	33563643	Tumor
chr1	200649706	1863	LOC101929224	0	targeted sequencing	Péneau et al. 2021	33563643	Tumor
chr10	20868625	957	MIR4675	-27650	targeted sequencing	Péneau et al. 2021	33563643	Tumor
chr4	71829938	1821	MOB1B	0	targeted sequencing	Péneau et al. 2021	33563643	Tumor
chr17	22254787	959	MTRNR2L1	-230110	targeted sequencing	Péneau et al. 2021	33563643	Tumor
chr17	22254939	1275	MTRNR2L1	-230110	targeted sequencing	Péneau et al. 2021	33563643	Tumor
chr7	142227332	665	MTRNR2L6	146591	targeted sequencing	Péneau et al. 2021	33563643	Tumor
chr17	3283542	1495	OR1E1	17218	targeted sequencing	Péneau et al. 2021	33563643	Tumor
chr8	107279699	2924	OXR1	2505	targeted sequencing	Péneau et al. 2021	33563643	Tumor
chr5	23613817	667	PRDM9	-85111	targeted sequencing	Péneau et al. 2021	33563643	Tumor
chr16	8187760	3048	TMEM114	388594	targeted sequencing	Péneau et al. 2021	33563643	Tumor
chr22	28495172	858	TTC28	0	targeted sequencing	Péneau et al. 2021	33563643	Tumor
chr8	17127932	2372	VPS37A	0	targeted sequencing	Péneau et al. 2021	33563643	Tumor
chr14	36405967	2474	BRMS1L	-64693	targeted sequencing	Péneau et al. 2021	33563643	Tumor
chr17	13485066	872	HS3ST3A1	0	targeted sequencing	Péneau et al. 2021	33563643	Tumor
chr17	13496294	2687	HS3ST3A1	0	targeted sequencing	Péneau et al. 2021	33563643	Tumor
chr14	63166074	2587	KCNH5	7113	targeted sequencing	Péneau et al. 2021	33563643	Tumor
chr12	95460	2078	LOC100288778	-4197	targeted sequencing	Péneau et al. 2021	33563643	Tumor
chr2	127596098	1611	LOC101929926	60364	targeted sequencing	Péneau et al. 2021	33563643	Tumor
chr8	37042183	844	MIR1268A	0	targeted sequencing	Péneau et al. 2021	33563643	Tumor
chr8	37042924	1510	MIR1268A	0	targeted sequencing	Péneau et al. 2021	33563643	Tumor
chr5	32854086	339	NPR3	-62145	targeted sequencing	Péneau et al. 2021	33563643	Tumor
chr18	78008266	132	PARD6G	-2242	targeted sequencing	Péneau et al. 2021	33563643	Tumor
chr8	37749903	3123	RAB11FIP1	0	targeted sequencing	Péneau et al. 2021	33563643	Tumor
chr1	57427233	1805	C8B	0	targeted sequencing	Péneau et al. 2021	33563643	Tumor
chr1	57427234	1137	C8B	0	targeted sequencing	Péneau et al. 2021	33563643	Tumor
chr3	180734578	NA	DNAJC19	-27016	targeted sequencing	Péneau et al. 2021	33563643	Tumor
chr20	24292309	2676	FLJ33581	-87085	targeted sequencing	Péneau et al. 2021	33563643	Tumor
chr19	4094404	445	MAP2K2	0	targeted sequencing	Péneau et al. 2021	33563643	Tumor

Chromosome	Integration site in host genome	Integration site in virus genome	Gene (distance, bp)	Regions	Methods	Author	PMID	Sample
chr4	75958386	410	PARM1	0	targeted sequencing	Péneau et al. 2021	33563643	Tumor
chr8	85808468	1238	RALYL	0	targeted sequencing	Péneau et al. 2021	33563643	Tumor
chr5	127449967	997	SLC12A2	0	targeted sequencing	Péneau et al. 2021	33563643	Tumor
chr17	15205500	2334	TEKT3	1491	targeted sequencing	Péneau et al. 2021	33563643	Tumor
chr8	91358119	1	LINC00534	0	targeted sequencing	Péneau et al. 2021	33563643	Tumor
chr7	97889511	1879	TECPR1	-7458	targeted sequencing	Péneau et al. 2021	33563643	Tumor
chr7	97889532	1815	TECPR1	-7458	targeted sequencing	Péneau et al. 2021	33563643	Tumor
chr5	1295115	127	TERT	0	targeted sequencing	Péneau et al. 2021	33563643	Tumor
chr5	1295122	1677	TERT	0	targeted sequencing	Péneau et al. 2021	33563643	Tumor
chr2	112627768	3180	ANAPC1	0	targeted sequencing	Péneau et al. 2021	33563643	Tumor
chrX	66776410	1718	AR	0	targeted sequencing	Péneau et al. 2021	33563643	Tumor
chr5	24413008	1826	CDH10	73367	targeted sequencing	Péneau et al. 2021	33563643	Tumor
chr5	24413030	2744	CDH10	73367	targeted sequencing	Péneau et al. 2021	33563643	Tumor
chr7	146077181	1462	CNTNAP2	0	targeted sequencing	Péneau et al. 2021	33563643	Tumor
chr12	89684050	2100	DUSP6	57552	targeted sequencing	Péneau et al. 2021	33563643	Tumor
chr14	86104803	45	FLRT2	-9355	targeted sequencing	Péneau et al. 2021	33563643	Tumor
chr9	37719166	1784	FRMPD1	0	targeted sequencing	Péneau et al. 2021	33563643	Tumor
chr4	58679209	1004	LOC101928851	-346624	targeted sequencing	Péneau et al. 2021	33563643	Tumor
chr4	58680046	1412	LOC101928851	-346624	targeted sequencing	Péneau et al. 2021	33563643	Tumor
chrUn_gl000220	151175	1807	MIR6724-1|MIR6724-2|MIR6724-3|MIR6724-4	-1719	targeted sequencing	Péneau et al. 2021	33563643	Tumor
chrUn_gl000220	151191	1847	MIR6724-1|MIR6724-2|MIR6724-3|MIR6724-4	-1719	targeted sequencing	Péneau et al. 2021	33563643	Tumor
chr16	16567256	1842	NPIPA7|NPIPA8	-79427	targeted sequencing	Péneau et al. 2021	33563643	Tumor
chr5	145345578	1602	SH3RF2	0	targeted sequencing	Péneau et al. 2021	33563643	Tumor
chr5	1295115	1808	TERT	0	targeted sequencing	Péneau et al. 2021	33563643	Tumor
chr5	1295166	1813	TERT	0	targeted sequencing	Péneau et al. 2021	33563643	Tumor
chr17	62282425	2031	TEX2	0	targeted sequencing	Péneau et al. 2021	33563643	Tumor
chr8	68168994	1530	ARFGEF1	0	targeted sequencing	Péneau et al. 2021	33563643	Tumor
chr8	68169019	1621	ARFGEF1	0	targeted sequencing	Péneau et al. 2021	33563643	Tumor
chr6	64680583	112	EYS	0	targeted sequencing	Péneau et al. 2021	33563643	Tumor
chr1	68505369	197	GNG12-AS1	0	targeted sequencing	Péneau et al. 2021	33563643	Tumor
chr6	135411886	2740	HBS1L	-35850	targeted sequencing	Péneau et al. 2021	33563643	Tumor
chr6	135411886	2741	HBS1L	-35850	targeted sequencing	Péneau et al. 2021	33563643	Tumor
chr12	123159152	2459	HCAR2	26280	targeted sequencing	Péneau et al. 2021	33563643	Tumor
chr20	43062349	2630	HNF4A	-342	targeted sequencing	Péneau et al. 2021	33563643	Tumor
chr20	43062351	1811	HNF4A	-342	targeted sequencing	Péneau et al. 2021	33563643	Tumor
chr1	146990000	1255	LINC00624	-301	targeted sequencing	Péneau et al. 2021	33563643	Tumor
chr4	182612146	1662	LOC90768	447667	targeted sequencing	Péneau et al. 2021	33563643	Tumor
chr5	44989326	1250	MRPS30	-173197	targeted sequencing	Péneau et al. 2021	33563643	Tumor
chr17	22247274	1808	MTRNR2L1	-222764	targeted sequencing	Péneau et al. 2021	33563643	Tumor
chr3	31778685	52	OSBPL10	0	targeted sequencing	Péneau et al. 2021	33563643	Tumor
chrX	38244866	72	OTC	0	targeted sequencing	Péneau et al. 2021	33563643	Tumor
chr2	154369973	423	RPRM	-34651	targeted sequencing	Péneau et al. 2021	33563643	Tumor
chr19	37463894	2584	ZNF568	0	targeted sequencing	Péneau et al. 2021	33563643	Tumor
chr19	37463900	1798	ZNF568	0	targeted sequencing	Péneau et al. 2021	33563643	Tumor
chr5	42938784	1998	FLJ32255	46715	targeted sequencing	Péneau et al. 2021	33563643	Tumor
chr5	42939890	2716	FLJ32255	45611	targeted sequencing	Péneau et al. 2021	33563643	Tumor
chr6	2283911	112	GMDS-AS1	0	targeted sequencing	Péneau et al. 2021	33563643	Tumor
chr8	43092875	3128	HGSNAT	-34823	targeted sequencing	Péneau et al. 2021	33563643	Tumor
chr8	43092984	750	HGSNAT	-34823	targeted sequencing	Péneau et al. 2021	33563643	Tumor
chr8	43094744	1576	HGSNAT	-36774	targeted sequencing	Péneau et al. 2021	33563643	Tumor
chr8	43094862	3157	HGSNAT	-36774	targeted sequencing	Péneau et al. 2021	33563643	Tumor
chr2	66995995	284	LOC101927577	-65631	targeted sequencing	Péneau et al. 2021	33563643	Tumor
chr12	73314580	869	LOC101928137	238390	targeted sequencing	Péneau et al. 2021	33563643	Tumor
chr13	88821869	2703	LOC105370306	0	targeted sequencing	Péneau et al. 2021	33563643	Tumor
chr5	28058759	1551	LOC105374698	226986	targeted sequencing	Péneau et al. 2021	33563643	Tumor
chr10	42388561	1465	LOC441666	438753	targeted sequencing	Péneau et al. 2021	33563643	Tumor
chr10	42389383	1725	LOC441666	437931	targeted sequencing	Péneau et al. 2021	33563643	Tumor
chr2	52308533	167	LOC730100	0	targeted sequencing	Péneau et al. 2021	33563643	Tumor
chr5	16173160	1889	MARCH11	0	targeted sequencing	Péneau et al. 2021	33563643	Tumor
chr5	163205914	3212	MAT2B	-259555	targeted sequencing	Péneau et al. 2021	33563643	Tumor
chr6	120756108	697	MIR3144	-419705	targeted sequencing	Péneau et al. 2021	33563643	Tumor
chr21	9825862	1965	MIR3648-1|MIR3648-2	0	targeted sequencing	Péneau et al. 2021	33563643	Tumor
chr21	9826890	2078	MIR3687-1|MIR3687-2	-458	targeted sequencing	Péneau et al. 2021	33563643	Tumor
chr12	55600882	405	OR10A7	13738	targeted sequencing	Péneau et al. 2021	33563643	Tumor
chr5	31777770	1659	PDZD2	0	targeted sequencing	Péneau et al. 2021	33563643	Tumor
chr5	1101920	2259	SLC12A7	0	targeted sequencing	Péneau et al. 2021	33563643	Tumor
chr17	9451115	2174	STX8	0	targeted sequencing	Péneau et al. 2021	33563643	Tumor
chr5	1295723	1177	TERT	0	targeted sequencing	Péneau et al. 2021	33563643	Tumor
chr6	80736877	612	TTK	0	targeted sequencing	Péneau et al. 2021	33563643	Tumor
chr5	37495214	1263	WDR70	0	targeted sequencing	Péneau et al. 2021	33563643	Tumor
chr5	818645	1828	ZDHHC11	0	targeted sequencing	Péneau et al. 2021	33563643	Tumor
chr7	61968962	3098	ZNF733P	782026	targeted sequencing	Péneau et al. 2021	33563643	Tumor
chr7	61970291	840	ZNF733P	781079	targeted sequencing	Péneau et al. 2021	33563643	Tumor
chr19	56761967	1820	ZSCAN5A	0	targeted sequencing	Péneau et al. 2021	33563643	Tumor
chr19	56761982	2581	ZSCAN5A	0	targeted sequencing	Péneau et al. 2021	33563643	Tumor
chr11	97888565	1683	CNTN5	1002162	targeted sequencing	Péneau et al. 2021	33563643	Tumor
chr11	97888718	2431	CNTN5	1002162	targeted sequencing	Péneau et al. 2021	33563643	Tumor
chr19	36213092	1252	KMT2B	0	targeted sequencing	Péneau et al. 2021	33563643	Tumor
chr19	36213118	1828	KMT2B	0	targeted sequencing	Péneau et al. 2021	33563643	Tumor
chr2	178653257	2795	PDE11A	0	targeted sequencing	Péneau et al. 2021	33563643	Tumor
chr5	31777770	NA	PDZD2	0	targeted sequencing	Péneau et al. 2021	33563643	Tumor
chr3	47140590	1362	SETD2	0	targeted sequencing	Péneau et al. 2021	33563643	Tumor
chr7	134565297	2504	CALD1	0	targeted sequencing	Péneau et al. 2021	33563643	Tumor
chr19	27738515	1867	LINC00662	542831	targeted sequencing	Péneau et al. 2021	33563643	Tumor
chr4	151597758	2668	LRBA	0	targeted sequencing	Péneau et al. 2021	33563643	Tumor
chr4	151597767	841	LRBA	0	targeted sequencing	Péneau et al. 2021	33563643	Tumor
chrX	72337840	474	NAP1L6	7707	targeted sequencing	Péneau et al. 2021	33563643	Tumor
chrX	72337849	946	NAP1L6	7707	targeted sequencing	Péneau et al. 2021	33563643	Tumor
chr11	45329533	21	SYT13	-20976	targeted sequencing	Péneau et al. 2021	33563643	Tumor
chr11	45329544	2646	SYT13	-20976	targeted sequencing	Péneau et al. 2021	33563643	Tumor
chr5	1297782	1812	TERT	-1951	targeted sequencing	Péneau et al. 2021	33563643	Tumor
chr15	53811642	1336	WDR72	0	targeted sequencing	Péneau et al. 2021	33563643	Tumor
chr8	135239268	362	ZFAT	250267	targeted sequencing	Péneau et al. 2021	33563643	Tumor
chr12	38224142	1696	ALG10B	486415	targeted sequencing	Péneau et al. 2021	33563643	Tumor

Supplementary Table S8 Continued

Chromosome	Integration site in host genome	Integration site in virus genome	Gene (distance, bp)	Regions	Methods	Author	PMID	Sample
chr1	200891303	579	C1orf106	-6326	targeted sequencing	Péneau et al. 2021	33563643	Tumor
chr21	18873161	1172	CXADR	11890	targeted sequencing	Péneau et al. 2021	33563643	Tumor
chrX	77576696	3114	CYSLTR1	0	targeted sequencing	Péneau et al. 2021	33563643	Tumor
chr2	228791301	2983	DAW1	-2242	targeted sequencing	Péneau et al. 2021	33563643	Tumor
chr9	37719166	1784	FRMPD1	0	targeted sequencing	Péneau et al. 2021	33563643	Tumor
chr4	42839879	1253	GRXCR1	55404	targeted sequencing	Péneau et al. 2021	33563643	Tumor
chr5	45540180	356	HCN1	0	targeted sequencing	Péneau et al. 2021	33563643	Tumor
chr8	43092939	1576	HGSNAT	-34905	targeted sequencing	Péneau et al. 2021	33563643	Tumor
chr13	19127030	1846	LINC00417	184728	targeted sequencing	Péneau et al. 2021	33563643	Tumor
chr13	19127051	1795	LINC00417	184728	targeted sequencing	Péneau et al. 2021	33563643	Tumor
chr1	73429878	1822	LINC01360	341857	targeted sequencing	Péneau et al. 2021	33563643	Tumor
chr5	16173160	1904	MARCH11	0	targeted sequencing	Péneau et al. 2021	33563643	Tumor
chr5	112442271	565	MCC	0	targeted sequencing	Péneau et al. 2021	33563643	Tumor
chr5	31777770	NA	PDZD2	0	targeted sequencing	Péneau et al. 2021	33563643	Tumor
chr17	16172102	545	PIGL	0	targeted sequencing	Péneau et al. 2021	33563643	Tumor
chr7	127281681	7	SND1	10362	targeted sequencing	Péneau et al. 2021	33563643	Tumor
chr2	139066756	1775	SPOPL	192594	targeted sequencing	Péneau et al. 2021	33563643	Tumor
chr5	1295568	1826	TERT	0	targeted sequencing	Péneau et al. 2021	33563643	Tumor
chr5	1295595	2812	TERT	0	targeted sequencing	Péneau et al. 2021	33563643	Tumor
chr3	180734718	2335	DNAJC19	-26405	targeted sequencing	Péneau et al. 2021	33563643	Tumor
chr4	33344067	581	LOC101928622	553549	targeted sequencing	Péneau et al. 2021	33563643	Tumor
chr3	180771898	2115	SOX2-OT	2047	targeted sequencing	Péneau et al. 2021	33563643	Tumor
chr4	106455382	2482	ARHGEF38	18220	targeted sequencing	Péneau et al. 2021	33563643	Tumor
chr1	121484777	1693	EMBP1	-170801	targeted sequencing	Péneau et al. 2021	33563643	Tumor
chr1	121485184	922	EMBP1	-170801	targeted sequencing	Péneau et al. 2021	33563643	Tumor
chr7	45933134	3020	IGFBP1	0	targeted sequencing	Péneau et al. 2021	33563643	Tumor
chr7	45933136	1800	IGFBP1	0	targeted sequencing	Péneau et al. 2021	33563643	Tumor
chr17	21304102	1159	KCNJ12	0	targeted sequencing	Péneau et al. 2021	33563643	Tumor
chr10	78740120	555	KCNMA1	0	targeted sequencing	Péneau et al. 2021	33563643	Tumor
chr4	189538832	575	LINC01060	-15592	targeted sequencing	Péneau et al. 2021	33563643	Tumor
chr22	46515324	488	MIRLET7BHG	-5478	targeted sequencing	Péneau et al. 2021	33563643	Tumor
chr22	46515331	1961	MIRLET7BHG	-5478	targeted sequencing	Péneau et al. 2021	33563643	Tumor
chr8	9836939	2257	MSRA	74840	targeted sequencing	Péneau et al. 2021	33563643	Tumor
chr6	133249609	1132	RPS12	-110906	targeted sequencing	Péneau et al. 2021	33563643	Tumor
chr5	1295410	1789	TERT	0	targeted sequencing	Péneau et al. 2021	33563643	Tumor
chr1	121485414	1940	EMBP1	-171493	targeted sequencing	Péneau et al. 2021	33563643	Tumor
chr5	42938782	1998	FLJ32255	46717	targeted sequencing	Péneau et al. 2021	33563643	Tumor
chr4	151597767	831	LRBA	0	targeted sequencing	Péneau et al. 2021	33563643	Tumor
chr11	45329533	NA	SYT13	-21418	targeted sequencing	Péneau et al. 2021	33563643	Tumor
chr8	135239268	338	ZFAT	250625	targeted sequencing	Péneau et al. 2021	33563643	Tumor
chr5	74925694	2635	ANKDD1B	0	targeted sequencing	Péneau et al. 2021	33563643	Tumor
chr2	160228774	710	BAZ2B	0	targeted sequencing	Péneau et al. 2021	33563643	Tumor
chr2	241532031	369	CAPN10	0	targeted sequencing	Péneau et al. 2021	33563643	Tumor
chr3	180333583	443	CCDC39|TTC14	0	targeted sequencing	Péneau et al. 2021	33563643	Tumor
chr14	66421050	905	FUT8	-209994	targeted sequencing	Péneau et al. 2021	33563643	Tumor
chr11	8275897	1777	LMO1	0	targeted sequencing	Péneau et al. 2021	33563643	Tumor
chr2	65109157	221	LOC101927438	-18392	targeted sequencing	Péneau et al. 2021	33563643	Tumor
chr12	23345879	2184	LOC101928441	-1358	targeted sequencing	Péneau et al. 2021	33563643	Tumor
chr12	87741095	1776	LOC105369879	-14221	targeted sequencing	Péneau et al. 2021	33563643	Tumor
chr12	87741105	1905	LOC105369879	-14221	targeted sequencing	Péneau et al. 2021	33563643	Tumor
chr10	42385195	999	LOC441666	441663	targeted sequencing	Péneau et al. 2021	33563643	Tumor
chr4	127266710	2574	MIR2054	-837960	targeted sequencing	Péneau et al. 2021	33563643	Tumor
chr22	48597191	333	MIR3201	72985	targeted sequencing	Péneau et al. 2021	33563643	Tumor
chr2	133668064	310	NCKAP5	0	targeted sequencing	Péneau et al. 2021	33563643	Tumor
chr22	30022456	702	NF2	0	targeted sequencing	Péneau et al. 2021	33563643	Tumor
chrX	81673952	410	POU3F4	1089114	targeted sequencing	Péneau et al. 2021	33563643	Tumor
chr14	36206994	738	RALGAPA1|RALGAPA1P1	0	targeted sequencing	Péneau et al. 2021	33563643	Tumor
chr5	155538938	2176	SGCD	214829	targeted sequencing	Péneau et al. 2021	33563643	Tumor
chr13	23799749	2067	SGCG	0	targeted sequencing	Péneau et al. 2021	33563643	Tumor
chr9	12916386	1436	SNORD137	56041	targeted sequencing	Péneau et al. 2021	33563643	Tumor
chr4	177241346	858	SPCS3	0	targeted sequencing	Péneau et al. 2021	33563643	Tumor
chr9	79862592	1848	VPS13A	0	targeted sequencing	Péneau et al. 2021	33563643	Tumor
chr19	9247949	1063	ZNF317	2209	targeted sequencing	Péneau et al. 2021	33563643	Tumor
chr7	61970283	1417	ZNF733P	781220	targeted sequencing	Péneau et al. 2021	33563643	Tumor
chr12	89391702	1290	LOC728084	13056	targeted sequencing	Péneau et al. 2021	33563643	Tumor
chr12	85437113	2887	LRRIQ1	0	targeted sequencing	Péneau et al. 2021	33563643	Tumor
chr2	33377846	1548	LTBP1	0	targeted sequencing	Péneau et al. 2021	33563643	Tumor
chr1	12071485	1788	MFN2	0	targeted sequencing	Péneau et al. 2021	33563643	Tumor
chr1	12071494	1925	MFN2	0	targeted sequencing	Péneau et al. 2021	33563643	Tumor
chr1	207227796	207	PFKFB2	0	targeted sequencing	Péneau et al. 2021	33563643	Tumor
chr15	90274037	2190	WDR93	0	targeted sequencing	Péneau et al. 2021	33563643	Tumor
chr9_gl000198_random	1	1526	.	-1	targeted sequencing	Péneau et al. 2021	33563643	Tumor
chr9	131860232	2916	CRAT	0	targeted sequencing	Péneau et al. 2021	33563643	Tumor
chr4	74874922	1637	CXCL5	-10476	targeted sequencing	Péneau et al. 2021	33563643	Tumor
chr3	185744836	2773	ETV5	19270	targeted sequencing	Péneau et al. 2021	33563643	Tumor
chr9	41080	1726	FAM138C	-4527	targeted sequencing	Péneau et al. 2021	33563643	Tumor
chr10	71113364	475	HK1	0	targeted sequencing	Péneau et al. 2021	33563643	Tumor
chr20	13215006	1626	ISM1	0	targeted sequencing	Péneau et al. 2021	33563643	Tumor
chr8	46841696	1926	LINC00293	910649	targeted sequencing	Péneau et al. 2021	33563643	Tumor
chr8	46854150	1390	LINC00293	897559	targeted sequencing	Péneau et al. 2021	33563643	Tumor
chr4	33116276	3077	LOC101927363	-761434	targeted sequencing	Péneau et al. 2021	33563643	Tumor
chr2	128327483	2188	LOC105373609|MYO7B	0	targeted sequencing	Péneau et al. 2021	33563643	Tumor
chr4	75078654	2055	MTHFD2L	0	targeted sequencing	Péneau et al. 2021	33563643	Tumor
chr17	22235996	3077	MTRNR2L1	-211007	targeted sequencing	Péneau et al. 2021	33563643	Tumor
chr17	22253729	2647	MTRNR2L1	-229238	targeted sequencing	Péneau et al. 2021	33563643	Tumor
chr2	50541795	2922	NRXN1	0	targeted sequencing	Péneau et al. 2021	33563643	Tumor
chr2	50543699	1603	NRXN1	0	targeted sequencing	Péneau et al. 2021	33563643	Tumor
chr3	111499328	2328	PHLDB2|PLCXD2	0	targeted sequencing	Péneau et al. 2021	33563643	Tumor
chr8	43827752	2080	POTEA	-609424	targeted sequencing	Péneau et al. 2021	33563643	Tumor
chr6	111627367	1655	REV3L	0	targeted sequencing	Péneau et al. 2021	33563643	Tumor
chr6	111627372	1476	REV3L	0	targeted sequencing	Péneau et al. 2021	33563643	Tumor
chr4	19860074	2959	SLIT2	393454	targeted sequencing	Péneau et al. 2021	33563643	Tumor
chr4	19870362	2734	SLIT2	382963	targeted sequencing	Péneau et al. 2021	33563643	Tumor
chr15	30006345	1789	TJP1	0	targeted sequencing	Péneau et al. 2021	33563643	Tumor

Chromosome	Integration site in host genome	Integration site in virus genome	Gene (distance, bp)	Regions	Methods	Author	PMID	Sample
chr15	30006351	2505	TJP1	0	targeted sequencing	Péneau et al. 2021	33563643	Tumor
chr7	61968962	3130	ZNF733P	782708	targeted sequencing	Péneau et al. 2021	33563643	Tumor
chr5	42938784	NA	FLJ32255	46717	targeted sequencing	Péneau et al. 2021	33563643	Tumor
chr3	82885462	1668	GBE1	-1074298	targeted sequencing	Péneau et al. 2021	33563643	Tumor
chr1	200944753	1889	KIF21B	0	targeted sequencing	Péneau et al. 2021	33563643	Tumor
chr14	47629697	1703	MDGA2	0	targeted sequencing	Péneau et al. 2021	33563643	Tumor
chr14	47629701	1809	MDGA2	0	targeted sequencing	Péneau et al. 2021	33563643	Tumor
chr2	122881146	1029	TSN	-355718	targeted sequencing	Péneau et al. 2021	33563643	Tumor
chr22	21268641	684	CRKL	3073	targeted sequencing	Péneau et al. 2021	33563643	Tumor
chr8	71896926	1483	EYA1	212742	targeted sequencing	Péneau et al. 2021	33563643	Tumor
chr4	8850467	1831	HMX1	0	targeted sequencing	Péneau et al. 2021	33563643	Tumor
chr4	8850496	1819	HMX1	0	targeted sequencing	Péneau et al. 2021	33563643	Tumor
chr1	195900869	1869	KCNT2	293884	targeted sequencing	Péneau et al. 2021	33563643	Tumor
chr1	27278006	1172	KDF1	0	targeted sequencing	Péneau et al. 2021	33563643	Tumor
chr3	191210853	638	LINCR-0002	0	targeted sequencing	Péneau et al. 2021	33563643	Tumor
chr2	81816002	2067	LOC100507201	-121932	targeted sequencing	Péneau et al. 2021	33563643	Tumor
chr3	148002032	2326	LOC100507461	-196216	targeted sequencing	Péneau et al. 2021	33563643	Tumor
chr2	129358062	2626	LOC101927881	263914	targeted sequencing	Péneau et al. 2021	33563643	Tumor
chr14	54403173	172	MIR5580	11811	targeted sequencing	Péneau et al. 2021	33563643	Tumor
chr10	120899813	2970	SFXN4	612	targeted sequencing	Péneau et al. 2021	33563643	Tumor
chr8	14811849	2959	SGCZ	0	targeted sequencing	Péneau et al. 2021	33563643	Tumor
chr18	45154824	2535	SMAD2	204642	targeted sequencing	Péneau et al. 2021	33563643	Tumor
chr9	117010220	1825	COL27A1	0	targeted sequencing	Péneau et al. 2021	33563643	Tumor
chr9	117010259	1817	COL27A1	0	targeted sequencing	Péneau et al. 2021	33563643	Tumor
chr5	1295152	1796	TERT	0	targeted sequencing	Péneau et al. 2021	33563643	Tumor
chr5	1295157	2076	TERT	0	targeted sequencing	Péneau et al. 2021	33563643	Tumor
chr4	191044276	1900	DBET	-55143	targeted sequencing	Péneau et al. 2021	33563643	Tumor
chr22	47905925	1900	LL22NC03-75H12.2	-22889	targeted sequencing	Péneau et al. 2021	33563643	Tumor
chr6	48499248	1974	PTCHD4	-462823	targeted sequencing	Péneau et al. 2021	33563643	Tumor
chr5	1295717	1826	TERT	0	targeted sequencing	Péneau et al. 2021	33563643	Tumor
chr6	25838006	1801	SLC17A1	-5085	targeted sequencing	Péneau et al. 2021	33563643	Tumor
chr6	25838046	1825	SLC17A1	-5085	targeted sequencing	Péneau et al. 2021	33563643	Tumor
chr8	35768398	1902	UNC5D	-114330	targeted sequencing	Péneau et al. 2021	33563643	Tumor
chr2	236888349	1022	AGAP1	0	targeted sequencing	Péneau et al. 2021	33563643	Tumor
chr13	63617352	569	LINC00376	139779	targeted sequencing	Péneau et al. 2021	33563643	Tumor
chr4	125300788	2103	LOC101927087	120078	targeted sequencing	Péneau et al. 2021	33563643	Tumor
chr4	1295419	1332	MAEA	0	targeted sequencing	Péneau et al. 2021	33563643	Tumor
chr6	165382324	1826	MEAT6	-146771	targeted sequencing	Péneau et al. 2021	33563643	Tumor
chr6	165396088	597	MEAT6	-159950	targeted sequencing	Péneau et al. 2021	33563643	Tumor
chr6	165399303	1817	MEAT6	-163751	targeted sequencing	Péneau et al. 2021	33563643	Tumor
chr6	165455029	1578	MEAT6	-218796	targeted sequencing	Péneau et al. 2021	33563643	Tumor
chr3	145308603	2020	PLOD2	478511	targeted sequencing	Péneau et al. 2021	33563643	Tumor
chr13	32032682	425	B3GLCT	-126111	targeted sequencing	Péneau et al. 2021	33563643	Tumor
chr8	46841713	790	LINC00293	910649	targeted sequencing	Péneau et al. 2021	33563643	Tumor
chrUn_gl000220	125717	1277	LOC100507412	0	targeted sequencing	Péneau et al. 2021	33563643	Tumor
chr10	42597514	318	LOC441666	229749	targeted sequencing	Péneau et al. 2021	33563643	Tumor
chr10	42599785	742	LOC441666	227267	targeted sequencing	Péneau et al. 2021	33563643	Tumor
chr11	131813113	1563	NTM	0	targeted sequencing	Péneau et al. 2021	33563643	Tumor
chr2	92269569	965	ACTR3BP2	-138944	targeted sequencing	Péneau et al. 2021	33563643	Tumor
chr7	2752575	504	AMZ1	0	targeted sequencing	Péneau et al. 2021	33563643	Tumor
chr16	46391672	2365	ANKRD26P1	111577	targeted sequencing	Péneau et al. 2021	33563643	Tumor
chr16	46398589	1635	ANKRD26P1	104426	targeted sequencing	Péneau et al. 2021	33563643	Tumor
chr16	46427964	2045	ANKRD26P1	75285	targeted sequencing	Péneau et al. 2021	33563643	Tumor
chr15	29400388	1052	APBA2	0	targeted sequencing	Péneau et al. 2021	33563643	Tumor
chr11	13318867	477	ARNTL	0	targeted sequencing	Péneau et al. 2021	33563643	Tumor
chr3	105972202	11	CBLB	-383936	targeted sequencing	Péneau et al. 2021	33563643	Tumor
chr3	1993602	749	CNTN4	146738	targeted sequencing	Péneau et al. 2021	33563643	Tumor
chr13	105675860	1555	DAOA-AS1	435379	targeted sequencing	Péneau et al. 2021	33563643	Tumor
chr4	191043514	548	DBET	-54177	targeted sequencing	Péneau et al. 2021	33563643	Tumor
chr1	10359	940	DDX11L1	1409	targeted sequencing	Péneau et al. 2021	33563643	Tumor
chrX	155260280	1396	DDX11L16	-1970	targeted sequencing	Péneau et al. 2021	33563643	Tumor
chr22	38962222	1513	DMC1	0	targeted sequencing	Péneau et al. 2021	33563643	Tumor
chr12	124300409	384	DNAH10	0	targeted sequencing	Péneau et al. 2021	33563643	Tumor
chr8	28040497	506	ELP3	0	targeted sequencing	Péneau et al. 2021	33563643	Tumor
chr1	121352301	1338	EMBP1	-38545	targeted sequencing	Péneau et al. 2021	33563643	Tumor
chr1	121355026	1826	EMBP1	-41185	targeted sequencing	Péneau et al. 2021	33563643	Tumor
chr1	121355105	1298	EMBP1	-41185	targeted sequencing	Péneau et al. 2021	33563643	Tumor
chr6	46082055	445	ENPP4	15646	targeted sequencing	Péneau et al. 2021	33563643	Tumor
chr12	29134884	867	FAR2	166900	targeted sequencing	Péneau et al. 2021	33563643	Tumor
chr2	48622723	2175	FOXN2	-16289	targeted sequencing	Péneau et al. 2021	33563643	Tumor
chr1	153788184	2086	GATAD2B	0	targeted sequencing	Péneau et al. 2021	33563643	Tumor
chr6	102518900	1821	GRIK2	-479	targeted sequencing	Péneau et al. 2021	33563643	Tumor
chr6	102519556	2354	GRIK2	-1598	targeted sequencing	Péneau et al. 2021	33563643	Tumor
chr5	62813320	1144	HTR1A	442394	targeted sequencing	Péneau et al. 2021	33563643	Tumor
chr7	46154209	2118	IGFBP3	-193177	targeted sequencing	Péneau et al. 2021	33563643	Tumor
chr13	21553237	127	LATS2	0	targeted sequencing	Péneau et al. 2021	33563643	Tumor
chr8	117220845	3168	LINC00536	0	targeted sequencing	Péneau et al. 2021	33563643	Tumor
chr6	58511140	2022	LINC00680|LINC00680-GUSBP4	-223301	targeted sequencing	Péneau et al. 2021	33563643	Tumor
chr10	7029655	2445	LINC00707	-144787	targeted sequencing	Péneau et al. 2021	33563643	Tumor
chr1	99587935	2228	LOC100129620	0	targeted sequencing	Péneau et al. 2021	33563643	Tumor
chr1	222112835	1737	LOC101929771	-98827	targeted sequencing	Péneau et al. 2021	33563643	Tumor
chr16	86863939	1687	LOC102724344	-92341	targeted sequencing	Péneau et al. 2021	33563643	Tumor
chr17	49459500	3098	LOC440446	-39425	targeted sequencing	Péneau et al. 2021	33563643	Tumor
chr1	240862587	146	MIR1273E	0	targeted sequencing	Péneau et al. 2021	33563643	Tumor
chr4	127065001	1822	MIR2054	-636539	targeted sequencing	Péneau et al. 2021	33563643	Tumor
chr11	28822407	2912	MIR8068	-323195	targeted sequencing	Péneau et al. 2021	33563643	Tumor
chr20	10359522	1207	MKKS	25723	targeted sequencing	Péneau et al. 2021	33563643	Tumor
chr17	22253121	1684	MTRNR2L1	-227151	targeted sequencing	Péneau et al. 2021	33563643	Tumor
chr17	22253975	1785	MTRNR2L1	-227151	targeted sequencing	Péneau et al. 2021	33563643	Tumor
chr17	22260954	1535	MTRNR2L1	-231996	targeted sequencing	Péneau et al. 2021	33563643	Tumor
chr12	125096766	2075	NCOR2	-44756	targeted sequencing	Péneau et al. 2021	33563643	Tumor
chrX	67543964	410	OPHN1	0	targeted sequencing	Péneau et al. 2021	33563643	Tumor
chr4	134783672	3110	PABPC4L	333817	targeted sequencing	Péneau et al. 2021	33563643	Tumor
chr13	98361480	655	RAP2A	-241058	targeted sequencing	Péneau et al. 2021	33563643	Tumor

Chromosome	Integration site in host genome	Integration site in virus genome	Gene (distance, bp)	Regions	Methods	Author	PMID	Sample
chr13	21717169	1194	SAP18	0	targeted sequencing	Péneau et al. 2021	33563643	Tumor
chr3	47140590	1336	SETD2	0	targeted sequencing	Péneau et al. 2021	33563643	Tumor
chr3	47140621	1930	SETD2	0	targeted sequencing	Péneau et al. 2021	33563643	Tumor
chr3	4319144	1418	SETMAR	25666	targeted sequencing	Péneau et al. 2021	33563643	Tumor
chr2	172729393	2994	SLC25A12	0	targeted sequencing	Péneau et al. 2021	33563643	Tumor
chr11	65152245	2354	SLC25A45	-855	targeted sequencing	Péneau et al. 2021	33563643	Tumor
chr9	1910632	1556	SMARCA2	104445	targeted sequencing	Péneau et al. 2021	33563643	Tumor
chr7	88069039	2052	STEAP4	-132446	targeted sequencing	Péneau et al. 2021	33563643	Tumor
chr1	28114626	2997	STX12	0	targeted sequencing	Péneau et al. 2021	33563643	Tumor
chr12	118681857	1556	TAOK3	0	targeted sequencing	Péneau et al. 2021	33563643	Tumor
chr12	115163517	590	TBX3	-41548	targeted sequencing	Péneau et al. 2021	33563643	Tumor
chr5	1300483	181	TERT	-4428	targeted sequencing	Péneau et al. 2021	33563643	Tumor
chr5	1300493	1828	TERT	-4428	targeted sequencing	Péneau et al. 2021	33563643	Tumor
chr15	76339730	188	TMEM266	12398	targeted sequencing	Péneau et al. 2021	33563643	Tumor
chr12	72720431	77	TRHDE	0	targeted sequencing	Péneau et al. 2021	33563643	Tumor
chr1	248043244	2625	TRIM58	0	targeted sequencing	Péneau et al. 2021	33563643	Tumor
chr14	51573403	389	TRIM9	-10981	targeted sequencing	Péneau et al. 2021	33563643	Tumor
chr9	73097024	2883	TRPM3	52670	targeted sequencing	Péneau et al. 2021	33563643	Tumor
chr15	50786949	585	USP8	0	targeted sequencing	Péneau et al. 2021	33563643	Tumor
chr7	55085559	2396	EGFR	337	targeted sequencing	Péneau et al. 2021	33563643	Tumor
chr7	55085574	630	EGFR	337	targeted sequencing	Péneau et al. 2021	33563643	Tumor
chr4	139403263	507	LINC00499	-57765	targeted sequencing	Péneau et al. 2021	33563643	Tumor
chr8	129978395	731	LINC00977	249884	targeted sequencing	Péneau et al. 2021	33563643	Tumor
chr8	112053498	1629	LINC01608	-13836	targeted sequencing	Péneau et al. 2021	33563643	Tumor
chr5	26636	514	PLEKHG4B	113737	targeted sequencing	Péneau et al. 2021	33563643	Tumor
chr5	29767	2502	PLEKHG4B	110605	targeted sequencing	Péneau et al. 2021	33563643	Tumor
chr17	59836034	1133	BRIP1	0	targeted sequencing	Péneau et al. 2021	33563643	Tumor
chr17	59836034	1234	BRIP1	0	targeted sequencing	Péneau et al. 2021	33563643	Tumor
chr7	71800245	6	CALN1	0	targeted sequencing	Péneau et al. 2021	33563643	Tumor
chr17	30120923	881	COPRS	57846	targeted sequencing	Péneau et al. 2021	33563643	Tumor
chr8	114397526	1594	CSMD3	0	targeted sequencing	Péneau et al. 2021	33563643	Tumor
chr8	114397912	1167	CSMD3	0	targeted sequencing	Péneau et al. 2021	33563643	Tumor
chr8	114399261	1139	CSMD3	0	targeted sequencing	Péneau et al. 2021	33563643	Tumor
chr8	114399799	1256	CSMD3	0	targeted sequencing	Péneau et al. 2021	33563643	Tumor
chr5	83878280	808	EDIL3	-197595	targeted sequencing	Péneau et al. 2021	33563643	Tumor
chr4	153145887	2089	FBXW7	96523	targeted sequencing	Péneau et al. 2021	33563643	Tumor
chr4	94303137	157	GRID2	0	targeted sequencing	Péneau et al. 2021	33563643	Tumor
chr1	233923492	2493	KCNK1	-115234	targeted sequencing	Péneau et al. 2021	33563643	Tumor
chr2	64721949	2566	LOC101927402	27374	targeted sequencing	Péneau et al. 2021	33563643	Tumor
chr8	2653095	937	LOC101927815	-66906	targeted sequencing	Péneau et al. 2021	33563643	Tumor
chr6	4240207	1213	LOC102724096	-49805	targeted sequencing	Péneau et al. 2021	33563643	Tumor
chr6	4240207	1774	LOC102724096	-49805	targeted sequencing	Péneau et al. 2021	33563643	Tumor
chr20	15545310	1835	MACROD2	0	targeted sequencing	Péneau et al. 2021	33563643	Tumor
chr20	15545830	1798	MACROD2	0	targeted sequencing	Péneau et al. 2021	33563643	Tumor
chr3	163634443	1139	MIR1263	251467	targeted sequencing	Péneau et al. 2021	33563643	Tumor
chr3	163634667	1162	MIR1263	254168	targeted sequencing	Péneau et al. 2021	33563643	Tumor
chr3	163634696	1384	MIR1263	254168	targeted sequencing	Péneau et al. 2021	33563643	Tumor
chr3	163637182	72	MIR1263	251467	targeted sequencing	Péneau et al. 2021	33563643	Tumor
chr6	4336115	1039	MIR7641-2	91963	targeted sequencing	Péneau et al. 2021	33563643	Tumor
chr8	89377983	1808	MMP16	-37982	targeted sequencing	Péneau et al. 2021	33563643	Tumor
chr8	89378233	1207	MMP16	-37982	targeted sequencing	Péneau et al. 2021	33563643	Tumor
chr6	141709967	509	NMBR	685479	targeted sequencing	Péneau et al. 2021	33563643	Tumor
chr2	51006182	1207	NRXN1	0	targeted sequencing	Péneau et al. 2021	33563643	Tumor
chr15	31919613	1618	OTUD7A	0	targeted sequencing	Péneau et al. 2021	33563643	Tumor
chrM	5356	391	RNR2	-1953	targeted sequencing	Péneau et al. 2021	33563643	Tumor
chr14	25687115	1239	STXBP6	-167435	targeted sequencing	Péneau et al. 2021	33563643	Tumor
chr20	43510518	301	YWHAB	3722	targeted sequencing	Péneau et al. 2021	33563643	Tumor
chr3	75920142	347	ZNF717	-85262	targeted sequencing	Péneau et al. 2021	33563643	Tumor
chrUn_gl000226	6764	2495	.	-1	targeted sequencing	Péneau et al. 2021	33563643	Tumor
chrUn_gl000226	7046	775	.	-1	targeted sequencing	Péneau et al. 2021	33563643	Tumor
chrUn_gl000226	13226	2735	.	-1	targeted sequencing	Péneau et al. 2021	33563643	Tumor
chrUn_gl000226	13859	1792	.	-1	targeted sequencing	Péneau et al. 2021	33563643	Tumor
chr2	92314037	777	ACTR3BP2	-183331	targeted sequencing	Péneau et al. 2021	33563643	Tumor
chr5	64397335	1441	ADAMTS6	47021	targeted sequencing	Péneau et al. 2021	33563643	Tumor
chr4	61957182	2529	ADGRL3	109656	targeted sequencing	Péneau et al. 2021	33563643	Tumor
chr5	14880076	1742	ANKH	-7704	targeted sequencing	Péneau et al. 2021	33563643	Tumor
chr16	46387064	1828	ANKRD26P1	116185	targeted sequencing	Péneau et al. 2021	33563643	Tumor
chr16	46393797	1042	ANKRD26P1	108215	targeted sequencing	Péneau et al. 2021	33563643	Tumor
chr16	46395034	1831	ANKRD26P1	108215	targeted sequencing	Péneau et al. 2021	33563643	Tumor
chr16	46404349	2329	ANKRD26P1	97476	targeted sequencing	Péneau et al. 2021	33563643	Tumor
chr16	46405119	1796	ANKRD26P1	97476	targeted sequencing	Péneau et al. 2021	33563643	Tumor
chr16	46406807	153	ANKRD26P1	96295	targeted sequencing	Péneau et al. 2021	33563643	Tumor
chr16	46423555	289	ANKRD26P1	79197	targeted sequencing	Péneau et al. 2021	33563643	Tumor
chr19	47380832	1725	AP2S1	-26278	targeted sequencing	Péneau et al. 2021	33563643	Tumor
chr4	35179857	2674	ARAP2	887763	targeted sequencing	Péneau et al. 2021	33563643	Tumor
chr4	35181699	2621	ARAP2	885921	targeted sequencing	Péneau et al. 2021	33563643	Tumor
chr10	18961126	474	ARL5B	0	targeted sequencing	Péneau et al. 2021	33563643	Tumor
chr12	27022871	1778	ASUN	35241	targeted sequencing	Péneau et al. 2021	33563643	Tumor
chr7	70160782	1815	AUTS2	0	targeted sequencing	Péneau et al. 2021	33563643	Tumor
chr7	70219007	2479	AUTS2	0	targeted sequencing	Péneau et al. 2021	33563643	Tumor
chr10	78379741	2465	C10orf11	-62608	targeted sequencing	Péneau et al. 2021	33563643	Tumor
chr11	109420429	1307	C11orf87	-120536	targeted sequencing	Péneau et al. 2021	33563643	Tumor
chr5	56000023	1827	C5orf67	-97964	targeted sequencing	Péneau et al. 2021	33563643	Tumor
chr8	96554219	204	C8orf37-AS1	0	targeted sequencing	Péneau et al. 2021	33563643	Tumor
chr12	28306678	2547	CCDC91	36682	targeted sequencing	Péneau et al. 2021	33563643	Tumor
chr4	76539733	1731	CDKL2	0	targeted sequencing	Péneau et al. 2021	33563643	Tumor
chr4	76540217	1657	CDKL2	0	targeted sequencing	Péneau et al. 2021	33563643	Tumor
chr4	56885269	2228	CEP135	0	targeted sequencing	Péneau et al. 2021	33563643	Tumor
chr5	98200830	1414	CHD1	0	targeted sequencing	Péneau et al. 2021	33563643	Tumor
chr21	19570837	2141	CHODL	0	targeted sequencing	Péneau et al. 2021	33563643	Tumor
chr1	153607692	1824	CHTOP	0	targeted sequencing	Péneau et al. 2021	33563643	Tumor
chr5	115442224	2374	COMMD10	0	targeted sequencing	Péneau et al. 2021	33563643	Tumor
chr5	115451019	2841	COMMD10	0	targeted sequencing	Péneau et al. 2021	33563643	Tumor
chr4	47740688	1358	CORIN	0	targeted sequencing	Péneau et al. 2021	33563643	Tumor

Chromosome	Integration site in host genome	Integration site in virus genome	Gene (distance, bp)	Regions	Methods	Author	PMID	Sample
chr16	21342151	1400	CRYM-AS1	-12122	targeted sequencing	Péneau et al. 2021	33563643	Tumor
chr4	70950469	1592	CSN1S2AP	0	targeted sequencing	Péneau et al. 2021	33563643	Tumor
chr10	68914412	1210	CTNNA3	0	targeted sequencing	Péneau et al. 2021	33563643	Tumor
chr21	24733561	1820	D21S2088E	0	targeted sequencing	Péneau et al. 2021	33563643	Tumor
chr12	31256480	1833	DDX11	0	targeted sequencing	Péneau et al. 2021	33563643	Tumor
chr12	100646756	782	DEPDC4	0	targeted sequencing	Péneau et al. 2021	33563643	Tumor
chrX	97365201	574	DIAPH2	-509604	targeted sequencing	Péneau et al. 2021	33563643	Tumor
chrX	97365379	1815	DIAPH2	-509604	targeted sequencing	Péneau et al. 2021	33563643	Tumor
chr5	118502482	2228	DMXL1	0	targeted sequencing	Péneau et al. 2021	33563643	Tumor
chr5	118177877	1811	DTWD2	0	targeted sequencing	Péneau et al. 2021	33563643	Tumor
chr1	53367704	1583	ECHDC2	0	targeted sequencing	Péneau et al. 2021	33563643	Tumor
chr1	121485060	1738	EMBP1	-170743	targeted sequencing	Péneau et al. 2021	33563643	Tumor
chr1	121485350	2446	EMBP1	-170743	targeted sequencing	Péneau et al. 2021	33563643	Tumor
chr2	207690537	235	FASTKD2	-29493	targeted sequencing	Péneau et al. 2021	33563643	Tumor
chr5	150921377	2534	FAT2	0	targeted sequencing	Péneau et al. 2021	33563643	Tumor
chr18	71584260	93	FBXO15	156328	targeted sequencing	Péneau et al. 2021	33563643	Tumor
chr5	114765746	1845	FEM1C	90862	targeted sequencing	Péneau et al. 2021	33563643	Tumor
chr11	69516137	1821	FGF19	0	targeted sequencing	Péneau et al. 2021	33563643	Tumor
chr11	69520968	814	FGF19	-1862	targeted sequencing	Péneau et al. 2021	33563643	Tumor
chr11	69572815	250	FGF4	14982	targeted sequencing	Péneau et al. 2021	33563643	Tumor
chr1	60160578	1449	FGGY	0	targeted sequencing	Péneau et al. 2021	33563643	Tumor
chr2	183700240	2740	FRZB	0	targeted sequencing	Péneau et al. 2021	33563643	Tumor
chr6	96672642	493	FUT9	-9154	targeted sequencing	Péneau et al. 2021	33563643	Tumor
chr13	93040109	1264	GPC5	0	targeted sequencing	Péneau et al. 2021	33563643	Tumor
chr12	12809885	1699	GPR19	3892	targeted sequencing	Péneau et al. 2021	33563643	Tumor
chr17	65182795	1659	HELZ	0	targeted sequencing	Péneau et al. 2021	33563643	Tumor
chr5	62720493	1623	HTR1A	535382	targeted sequencing	Péneau et al. 2021	33563643	Tumor
chr12	26984870	1840	ITPR2	0	targeted sequencing	Péneau et al. 2021	33563643	Tumor
chr4	71521760	1622	JCHAIN	0	targeted sequencing	Péneau et al. 2021	33563643	Tumor
chr10	78517209	1801	KCNMA1	111682	targeted sequencing	Péneau et al. 2021	33563643	Tumor
chr10	78962945	1824	KCNMA1	0	targeted sequencing	Péneau et al. 2021	33563643	Tumor
chr10	79232020	1815	KCNMA1	0	targeted sequencing	Péneau et al. 2021	33563643	Tumor
chr10	79244143	467	KCNMA1	0	targeted sequencing	Péneau et al. 2021	33563643	Tumor
chr19	10594424	1599	KEAP1	2190	targeted sequencing	Péneau et al. 2021	33563643	Tumor
chr4	57072011	1337	KIAA1211	0	targeted sequencing	Péneau et al. 2021	33563643	Tumor
chr13	73786182	2007	KLF5	-134392	targeted sequencing	Péneau et al. 2021	33563643	Tumor
chr4	39142700	1510	KLHL5	-14847	targeted sequencing	Péneau et al. 2021	33563643	Tumor
chr19	40294650	1375	LEUTX	-17673	targeted sequencing	Péneau et al. 2021	33563643	Tumor
chr8	46844373	1821	LINC00293	907976	targeted sequencing	Péneau et al. 2021	33563643	Tumor
chr8	46853555	931	LINC00293	898753	targeted sequencing	Péneau et al. 2021	33563643	Tumor
chr6	133477418	1820	LINC00326	-49491	targeted sequencing	Péneau et al. 2021	33563643	Tumor
chr13	112559639	3207	LINC00354	-3997	targeted sequencing	Péneau et al. 2021	33563643	Tumor
chr13	81949643	916	LINC00564	-148525	targeted sequencing	Péneau et al. 2021	33563643	Tumor
chr19	27732212	738	LINC00662	548672	targeted sequencing	Péneau et al. 2021	33563643	Tumor
chr19	27736420	41	LINC00662	544890	targeted sequencing	Péneau et al. 2021	33563643	Tumor
chr19	27736493	1825	LINC00662	544890	targeted sequencing	Péneau et al. 2021	33563643	Tumor
chr19	27738412	1583	LINC00662	542832	targeted sequencing	Péneau et al. 2021	33563643	Tumor
chr19	27738564	2217	LINC00662	542832	targeted sequencing	Péneau et al. 2021	33563643	Tumor
chr1	188010390	2507	LINC01037	-563835	targeted sequencing	Péneau et al. 2021	33563643	Tumor
chr7	152919073	1610	LINC01287	177512	targeted sequencing	Péneau et al. 2021	33563643	Tumor
chr7	152921877	3197	LINC01287	175018	targeted sequencing	Péneau et al. 2021	33563643	Tumor
chr6	85064608	894	LINC01611	66036	targeted sequencing	Péneau et al. 2021	33563643	Tumor
chr11	71582339	2843	LOC100133315	0	targeted sequencing	Péneau et al. 2021	33563643	Tumor
chr5	39665067	1204	LOC101926940	-140257	targeted sequencing	Péneau et al. 2021	33563643	Tumor
chr5	159063630	2287	LOC101927766	140152	targeted sequencing	Péneau et al. 2021	33563643	Tumor
chr8	138570406	2057	LOC101927915	-144408	targeted sequencing	Péneau et al. 2021	33563643	Tumor
chr11	69872812	2220	LOC101928443	29524	targeted sequencing	Péneau et al. 2021	33563643	Tumor
chr6	74677226	1180	LOC101928516	101941	targeted sequencing	Péneau et al. 2021	33563643	Tumor
chr11	80910200	2706	LOC101928944	-436354	targeted sequencing	Péneau et al. 2021	33563643	Tumor
chr5	105566470	1803	LOC102467213	584428	targeted sequencing	Péneau et al. 2021	33563643	Tumor
chr12	90934606	1400	LOC105369893	-240131	targeted sequencing	Péneau et al. 2021	33563643	Tumor
chr16	32412628	2653	LOC390705	-111263	targeted sequencing	Péneau et al. 2021	33563643	Tumor
chr16	32412648	1573	LOC390705	-111263	targeted sequencing	Péneau et al. 2021	33563643	Tumor
chr10	42385731	1198	LOC441666	441583	targeted sequencing	Péneau et al. 2021	33563643	Tumor
chr10	42396369	2230	LOC441666	430816	targeted sequencing	Péneau et al. 2021	33563643	Tumor
chr10	42599200	959	LOC441666	227490	targeted sequencing	Péneau et al. 2021	33563643	Tumor
chr14	41802605	343	LOC644919	-192173	targeted sequencing	Péneau et al. 2021	33563643	Tumor
chr7	118824180	1592	LVCAT5	434719	targeted sequencing	Péneau et al. 2021	33563643	Tumor
chr5	56126688	1199	MAP3K1	0	targeted sequencing	Péneau et al. 2021	33563643	Tumor
chr2	102498482	775	MAP4K4	0	targeted sequencing	Péneau et al. 2021	33563643	Tumor
chr12	116022566	1826	MED13L	373815	targeted sequencing	Péneau et al. 2021	33563643	Tumor
chr18	35542405	1453	MIR4318	-305123	targeted sequencing	Péneau et al. 2021	33563643	Tumor
chr1	22533609	1822	MIR4418	58926	targeted sequencing	Péneau et al. 2021	33563643	Tumor
chr21	24034721	2944	MIR6130	416885	targeted sequencing	Péneau et al. 2021	33563643	Tumor
chr20	26310005	2436	MIR663AHG	-120136	targeted sequencing	Péneau et al. 2021	33563643	Tumor
chr20	26310689	1512	MIR663AHG	-120136	targeted sequencing	Péneau et al. 2021	33563643	Tumor
chrUn_gl000220	145174	287	MIR6724-1|MIR6724-2|MIR6724-3|MIR6724-4	3530	targeted sequencing	Péneau et al. 2021	33563643	Tumor
chr14	45874337	2775	MIS18BP1	-151732	targeted sequencing	Péneau et al. 2021	33563643	Tumor
chr13	28047663	1826	MTIF3	-22924	targeted sequencing	Péneau et al. 2021	33563643	Tumor
chr17	22004866	1152	MTRNR2L1	17141	targeted sequencing	Péneau et al. 2021	33563643	Tumor
chr17	22258810	1076	MTRNR2L1	-234292	targeted sequencing	Péneau et al. 2021	33563643	Tumor
chr10	57679244	141	MTRNR2L5	-318757	targeted sequencing	Péneau et al. 2021	33563643	Tumor
chr11	69043981	1826	MYEOV	17461	targeted sequencing	Péneau et al. 2021	33563643	Tumor
chr11	69143138	1803	MYEOV	-78384	targeted sequencing	Péneau et al. 2021	33563643	Tumor
chr5	53072131	1858	NDUFS4	-92552	targeted sequencing	Péneau et al. 2021	33563643	Tumor
chr1	6582793	2594	NOL9	0	targeted sequencing	Péneau et al. 2021	33563643	Tumor
chr10	84536192	2854	NRG3	0	targeted sequencing	Péneau et al. 2021	33563643	Tumor
chr8	59641251	1821	NSMAF	-68847	targeted sequencing	Péneau et al. 2021	33563643	Tumor
chr2	226396893	667	NYAP2	0	targeted sequencing	Péneau et al. 2021	33563643	Tumor
chr5	141190647	1111	PCDH1	41815	targeted sequencing	Péneau et al. 2021	33563643	Tumor
chr4	39833810	764	PDS5A	0	targeted sequencing	Péneau et al. 2021	33563643	Tumor
chr4	39849182	1606	PDS5A	0	targeted sequencing	Péneau et al. 2021	33563643	Tumor
chr4	39895138	1964	PDS5A	0	targeted sequencing	Péneau et al. 2021	33563643	Tumor
chr4	39897333	2235	PDS5A	0	targeted sequencing	Péneau et al. 2021	33563643	Tumor

Chromosome	Integration site in host genome	Integration site in virus genome	Gene (distance, bp)	Regions	Methods	Author	PMID	Sample
chr5	51487161	1537	PELO	596257	targeted sequencing	Péneau et al. 2021	33563643	Tumor
chr5	51919437	3136	PELO	164214	targeted sequencing	Péneau et al. 2021	33563643	Tumor
chr5	54808610	1656	PLPP1	0	targeted sequencing	Péneau et al. 2021	33563643	Tumor
chr5	75052446	1797	POC5	-38902	targeted sequencing	Péneau et al. 2021	33563643	Tumor
chr10	129807550	3051	PTPRE	0	targeted sequencing	Péneau et al. 2021	33563643	Tumor
chrY	9014728	2726	RBMY1A3P	139942	targeted sequencing	Péneau et al. 2021	33563643	Tumor
chr10	79833677	639	RPS24	-16877	targeted sequencing	Péneau et al. 2021	33563643	Tumor
chr10	79833974	2841	RPS24	-16877	targeted sequencing	Péneau et al. 2021	33563643	Tumor
chr13	78050705	1824	SCEL	58957	targeted sequencing	Péneau et al. 2021	33563643	Tumor
chr20	44668434	1378	SLC12A5	0	targeted sequencing	Péneau et al. 2021	33563643	Tumor
chr4	72077775	1425	SLC4A4	0	targeted sequencing	Péneau et al. 2021	33563643	Tumor
chr12	20879541	605	SLCO1C1	0	targeted sequencing	Péneau et al. 2021	33563643	Tumor
chrY	58822313	1367	SPRY3	277974	targeted sequencing	Péneau et al. 2021	33563643	Tumor
chrY	58822330	1865	SPRY3	277974	targeted sequencing	Péneau et al. 2021	33563643	Tumor
chrY	58836343	1677	SPRY3	263729	targeted sequencing	Péneau et al. 2021	33563643	Tumor
chr3	140797926	2209	SPSB4	0	targeted sequencing	Péneau et al. 2021	33563643	Tumor
chr6	144532349	1817	STX11	-19273	targeted sequencing	Péneau et al. 2021	33563643	Tumor
chr4	64666784	2131	TECRL	477393	targeted sequencing	Péneau et al. 2021	33563643	Tumor
chr4	64721134	869	TECRL	422866	targeted sequencing	Péneau et al. 2021	33563643	Tumor
chr5	1295165	1827	TERT	0	targeted sequencing	Péneau et al. 2021	33563643	Tumor
chr6	169694626	1152	THBS2	-40417	targeted sequencing	Péneau et al. 2021	33563643	Tumor
chr1	173184009	1535	TNFSF4	-7396	targeted sequencing	Péneau et al. 2021	33563643	Tumor
chr11	68849206	1589	TPCN2	0	targeted sequencing	Péneau et al. 2021	33563643	Tumor
chr11	35715006	2774	TRIM44	0	targeted sequencing	Péneau et al. 2021	33563643	Tumor
chr3	160154640	2489	TRIM59	0	targeted sequencing	Péneau et al. 2021	33563643	Tumor
chr5	949489	1803	TRIP13	-31325	targeted sequencing	Péneau et al. 2021	33563643	Tumor
chr4	39536830	1822	UGDH-AS1	0	targeted sequencing	Péneau et al. 2021	33563643	Tumor
chr12	101758402	987	UTP20	0	targeted sequencing	Péneau et al. 2021	33563643	Tumor
chr7	92941629	423	VPS50	0	targeted sequencing	Péneau et al. 2021	33563643	Tumor
chr11	32398676	165	WT1	10527	targeted sequencing	Péneau et al. 2021	33563643	Tumor
chr6	74168521	2129	MTO1	2353	targeted sequencing	Péneau et al. 2021	33563643	Tumor
chr6	74168523	1761	MTO1	2353	targeted sequencing	Péneau et al. 2021	33563643	Tumor
chrY	8276501	1588	LINC00279	229834	targeted sequencing	Péneau et al. 2021	33563643	Tumor
chr7	35407797	2854	LOC401324	0	targeted sequencing	Péneau et al. 2021	33563643	Tumor
chr8	31330656	1828	NRG1	165968	targeted sequencing	Péneau et al. 2021	33563643	Tumor
chr4	42404659	1811	SHISA3	0	targeted sequencing	Péneau et al. 2021	33563643	Tumor
chrM	16200	2752	RNR2	-12970	targeted sequencing	Péneau et al. 2021	33563643	Tumor
chrX	84380277	1821	SATL1	-16300	targeted sequencing	Péneau et al. 2021	33563643	Tumor
chr14	50702801	1761	SOS2	-3657	targeted sequencing	Péneau et al. 2021	33563643	Tumor
chr12	55068612	171	DCD	-26331	targeted sequencing	Péneau et al. 2021	33563643	Tumor
chr6	80648686	1362	ELOVL4	0	targeted sequencing	Péneau et al. 2021	33563643	Tumor
chr12	71732176	836	LGR5	100766	targeted sequencing	Péneau et al. 2021	33563643	Tumor
chr12	71732178	1877	LGR5	100766	targeted sequencing	Péneau et al. 2021	33563643	Tumor
chr11	64330144	2851	SLC22A11	0	targeted sequencing	Péneau et al. 2021	33563643	Tumor
chr6	49526654	189	C6orf141	-6458	targeted sequencing	Péneau et al. 2021	33563643	Tumor
chr19	30291247	1834	CCNE1	11084	targeted sequencing	Péneau et al. 2021	33563643	Tumor
chr19	30291287	2844	CCNE1	11084	targeted sequencing	Péneau et al. 2021	33563643	Tumor
chr9	130983369	2590	DNM1	0	targeted sequencing	Péneau et al. 2021	33563643	Tumor
chrX	141579114	2758	MAGEC2	-285893	targeted sequencing	Péneau et al. 2021	33563643	Tumor
chr6	49411817	2224	MUT	0	targeted sequencing	Péneau et al. 2021	33563643	Tumor
chr18	78016222	881	PARD6G	-10786	targeted sequencing	Péneau et al. 2021	33563643	Tumor
chr12	79363502	1789	SYT1	0	targeted sequencing	Péneau et al. 2021	33563643	Tumor
chr5	1295207	2846	TERT	0	targeted sequencing	Péneau et al. 2021	33563643	Tumor
chr5	1295275	1827	TERT	0	targeted sequencing	Péneau et al. 2021	33563643	Tumor
chr5	15794247	2419	FBXL7	0	targeted sequencing	Péneau et al. 2021	33563643	Tumor
chr21	9825693	1828	MIR3648-1|MIR3648-2	139	targeted sequencing	Péneau et al. 2021	33563643	Tumor
chr21	9826963	1858	MIR3687-1|MIR3687-2	-700	targeted sequencing	Péneau et al. 2021	33563643	Tumor
chr6	49126113	1505	MUT	271592	targeted sequencing	Péneau et al. 2021	33563643	Tumor
chr6	49126115	466	MUT	271592	targeted sequencing	Péneau et al. 2021	33563643	Tumor
chr8	39474455	3045	ADAM18	0	targeted sequencing	Péneau et al. 2021	33563643	Tumor
chr2	130780761	187	FAR2P1	2684	targeted sequencing	Péneau et al. 2021	33563643	Tumor
chr7	67556624	610	LOC102723427	-58947	targeted sequencing	Péneau et al. 2021	33563643	Tumor
chr18	19477338	343	MIB1	-26420	targeted sequencing	Péneau et al. 2021	33563643	Tumor
chr10	102141753	693	OLMALINC	0	targeted sequencing	Péneau et al. 2021	33563643	Tumor
chr9	96682929	2543	BARX1	30980	targeted sequencing	Péneau et al. 2021	33563643	Tumor
chr11	10625785	2297	MRVI1	0	targeted sequencing	Péneau et al. 2021	33563643	Tumor
chr15	72229923	2730	MYO9A	0	targeted sequencing	Péneau et al. 2021	33563643	Tumor
chr19	33495203	2158	RHPN2	0	targeted sequencing	Péneau et al. 2021	33563643	Tumor
chr19	33495203	2178	RHPN2	0	targeted sequencing	Péneau et al. 2021	33563643	Tumor
chr5	1295172	1814	TERT	0	targeted sequencing	Péneau et al. 2021	33563643	Tumor
chr5	1295176	2502	TERT	0	targeted sequencing	Péneau et al. 2021	33563643	Tumor
chr15	50796212	588	USP50	0	targeted sequencing	Péneau et al. 2021	33563643	Tumor
chr13	54052076	1945	LINC01065	-326040	targeted sequencing	Péneau et al. 2021	33563643	Tumor
chr4	155863866	2789	RBM46	-113370	targeted sequencing	Péneau et al. 2021	33563643	Tumor
chr5	1295733	1828	TERT	0	targeted sequencing	Péneau et al. 2021	33563643	Tumor
chr5	1295793	1876	TERT	0	targeted sequencing	Péneau et al. 2021	33563643	Tumor
chr8	106455501	3128	ZFPM2	0	targeted sequencing	Péneau et al. 2021	33563643	Tumor
chr8	106456846	211	ZFPM2	0	targeted sequencing	Péneau et al. 2021	33563643	Tumor
chr20	32891369	1820	AHCY	0	targeted sequencing	Péneau et al. 2021	33563643	Tumor
chr20	32945331	1818	ITCH	5710	targeted sequencing	Péneau et al. 2021	33563643	Tumor
chr7	111875376	2952	ZNF277	0	targeted sequencing	Péneau et al. 2021	33563643	Tumor
chr7	38685048	1829	AMPH	-12908	targeted sequencing	Péneau et al. 2021	33563643	Tumor
chr17	13094124	2001	ELAC2	-172743	targeted sequencing	Péneau et al. 2021	33563643	Tumor
chr7	30797455	815	INMT|INMT-FAM188B	0	targeted sequencing	Péneau et al. 2021	33563643	Tumor
chr5	3679920	1249	IRX1	-77542	targeted sequencing	Péneau et al. 2021	33563643	Tumor
chr3	19422686	416	KCNH8	0	targeted sequencing	Péneau et al. 2021	33563643	Tumor
chr20	62038463	560	KCNQ2	0	targeted sequencing	Péneau et al. 2021	33563643	Tumor
chr5	3432484	1657	LINC01019	0	targeted sequencing	Péneau et al. 2021	33563643	Tumor
chr5	3432642	1538	LINC01019	0	targeted sequencing	Péneau et al. 2021	33563643	Tumor
chr5	12964384	1290	LINC01194	-158315	targeted sequencing	Péneau et al. 2021	33563643	Tumor
chr7	131468221	507	LOC101928782	126758	targeted sequencing	Péneau et al. 2021	33563643	Tumor
chr4	55432809	3157	LOC339978	36456	targeted sequencing	Péneau et al. 2021	33563643	Tumor
chr5	6864675	3063	MIR4278	-36641	targeted sequencing	Péneau et al. 2021	33563643	Tumor
chr17	81195078	260	RPL23AP87	-6391	targeted sequencing	Péneau et al. 2021	33563643	Tumor

Chromosome	Integration site in host genome	Integration site in virus genome	Gene (distance, bp)	Regions	Methods	Author	PMID	Sample
chr4	25869558	729	SEL1L3	-4341	targeted sequencing	Péneau et al. 2021	33563643	Tumor
chr4	177316428	457	SPCS3	-63032	targeted sequencing	Péneau et al. 2021	33563643	Tumor
chr5	1295998	1124	TERT	0	targeted sequencing	Péneau et al. 2021	33563643	Tumor
chr9	103070830	933	TEX10	0	targeted sequencing	Péneau et al. 2021	33563643	Tumor
chr8	36800168	1855	KCNU1	-5863	targeted sequencing	Péneau et al. 2021	33563643	Tumor
chr8	36800199	1819	KCNU1	-5863	targeted sequencing	Péneau et al. 2021	33563643	Tumor
chr11	11898165	2636	USP47	0	targeted sequencing	Péneau et al. 2021	33563643	Tumor
chr20	6286072	2064	CASC20	121306	targeted sequencing	Péneau et al. 2021	33563643	Tumor
chr9	98710169	1839	ERCC6L2	0	targeted sequencing	Péneau et al. 2021	33563643	Tumor
chr9	98710174	1828	ERCC6L2	0	targeted sequencing	Péneau et al. 2021	33563643	Tumor
chr6	62274673	1813	MTRNR2L9	9333	targeted sequencing	Péneau et al. 2021	33563643	Tumor
chr8	22146030	2866	PIWIL2	0	targeted sequencing	Péneau et al. 2021	33563643	Tumor
chr9	38371801	3229	ALDH1B1	20183	targeted sequencing	Péneau et al. 2021	33563643	Tumor
chrY	59362897	1542	DDX11L16	-1981	targeted sequencing	Péneau et al. 2021	33563643	Tumor
chrY	59363351	2251	DDX11L16	-1981	targeted sequencing	Péneau et al. 2021	33563643	Tumor
chr9	37095004	800	EBLN3P	-3775	targeted sequencing	Péneau et al. 2021	33563643	Tumor
chrY	13479554	1929	GYG2P1	1038239	targeted sequencing	Péneau et al. 2021	33563643	Tumor
chr6	27860659	2349	HIST1H2AM	0	targeted sequencing	Péneau et al. 2021	33563643	Tumor
chr6	27874175	1760	OR2B2	4662	targeted sequencing	Péneau et al. 2021	33563643	Tumor
chr18	78016288	1406	PARD6G	-10793	targeted sequencing	Péneau et al. 2021	33563643	Tumor
chr11	36450801	1815	PRR5L	0	targeted sequencing	Péneau et al. 2021	33563643	Tumor
chr11	36450845	2685	PRR5L	0	targeted sequencing	Péneau et al. 2021	33563643	Tumor
chr7	48574513	1976	ABCA13	0	targeted sequencing	Péneau et al. 2021	33563643	Tumor
chr7	45654027	3134	ADCY1	0	targeted sequencing	Péneau et al. 2021	33563643	Tumor
chr8	131975033	2640	ADCY8	0	targeted sequencing	Péneau et al. 2021	33563643	Tumor
chr3	105059000	2565	ALCAM	26429	targeted sequencing	Péneau et al. 2021	33563643	Tumor
chr16	46396702	2395	ANKRD26P1	106387	targeted sequencing	Péneau et al. 2021	33563643	Tumor
chr11	46702553	540	ARHGAP1	0	targeted sequencing	Péneau et al. 2021	33563643	Tumor
chr12	108169705	1542	ASCL4	0	targeted sequencing	Péneau et al. 2021	33563643	Tumor
chr17	58898710	391	BCAS3	0	targeted sequencing	Péneau et al. 2021	33563643	Tumor
chrX	95282898	408	BRDTP1	308977	targeted sequencing	Péneau et al. 2021	33563643	Tumor
chr2	119883045	1911	C1QL2	30774	targeted sequencing	Péneau et al. 2021	33563643	Tumor
chr17	50462912	1894	CA10	-225535	targeted sequencing	Péneau et al. 2021	33563643	Tumor
chr10	18718024	1299	CACNB2	0	targeted sequencing	Péneau et al. 2021	33563643	Tumor
chr8	128563143	1819	CASC8	-68759	targeted sequencing	Péneau et al. 2021	33563643	Tumor
chr12	82714255	360	CCDC59	31672	targeted sequencing	Péneau et al. 2021	33563643	Tumor
chr9	22170504	2175	CDKN2B-AS1	-49411	targeted sequencing	Péneau et al. 2021	33563643	Tumor
chr15	78859002	272	CHRNA5	0	targeted sequencing	Péneau et al. 2021	33563643	Tumor
chr16	81764044	1726	CMIP	-18473	targeted sequencing	Péneau et al. 2021	33563643	Tumor
chr2	125845974	1890	CNTNAP5	-172819	targeted sequencing	Péneau et al. 2021	33563643	Tumor
chr17	30175069	2784	COPRS	3815	targeted sequencing	Péneau et al. 2021	33563643	Tumor
chr17	28736429	1405	CPD	0	targeted sequencing	Péneau et al. 2021	33563643	Tumor
chr8	5313335	2013	CSMD1	-460904	targeted sequencing	Péneau et al. 2021	33563643	Tumor
chr8	114128324	1553	CSMD3	0	targeted sequencing	Péneau et al. 2021	33563643	Tumor
chr12	64023091	780	DPY19L2	0	targeted sequencing	Péneau et al. 2021	33563643	Tumor
chr17	17883467	2793	DRC3	0	targeted sequencing	Péneau et al. 2021	33563643	Tumor
chr18	28329359	1907	DSC3	239819	targeted sequencing	Péneau et al. 2021	33563643	Tumor
chr1	221926292	1931	DUSP10	-10666	targeted sequencing	Péneau et al. 2021	33563643	Tumor
chr6	53268980	2696	ELOVL5	-54824	targeted sequencing	Péneau et al. 2021	33563643	Tumor
chrX	129842480	1522	ENOX2	0	targeted sequencing	Péneau et al. 2021	33563643	Tumor
chr5	64008179	1442	FAM159B	0	targeted sequencing	Péneau et al. 2021	33563643	Tumor
chr15	59756136	390	FAM81A	0	targeted sequencing	Péneau et al. 2021	33563643	Tumor
chr11	71908814	1596	FOLR1	-1447	targeted sequencing	Péneau et al. 2021	33563643	Tumor
chr1	89910124	736	GBP1P1	-19363	targeted sequencing	Péneau et al. 2021	33563643	Tumor
chrX	121800085	1936	GRIA3	518011	targeted sequencing	Péneau et al. 2021	33563643	Tumor
chr12	67161059	391	GRIP1	-87906	targeted sequencing	Péneau et al. 2021	33563643	Tumor
chr3	7285280	2944	GRM7	0	targeted sequencing	Péneau et al. 2021	33563643	Tumor
chr1	221033486	1089	HLX-AS1	0	targeted sequencing	Péneau et al. 2021	33563643	Tumor
chr3	129005698	3048	HMCES	0	targeted sequencing	Péneau et al. 2021	33563643	Tumor
chr2	24440552	2632	ITSN2	0	targeted sequencing	Péneau et al. 2021	33563643	Tumor
chr1	196037532	302	KCNT2	157378	targeted sequencing	Péneau et al. 2021	33563643	Tumor
chr19	18368035	1894	KIAA1683	0	targeted sequencing	Péneau et al. 2021	33563643	Tumor
chr5	154801099	2547	KIF4B	-403261	targeted sequencing	Péneau et al. 2021	33563643	Tumor
chr12	89129410	2615	KITLG	-155160	targeted sequencing	Péneau et al. 2021	33563643	Tumor
chr8	47772496	374	LINC00293	-5089	targeted sequencing	Péneau et al. 2021	33563643	Tumor
chr7	85416050	2797	LINC00972	-297396	targeted sequencing	Péneau et al. 2021	33563643	Tumor
chr9	22614942	55	LINC01239	31089	targeted sequencing	Péneau et al. 2021	33563643	Tumor
chr9	82755542	1185	LINC01507	-105901	targeted sequencing	Péneau et al. 2021	33563643	Tumor
chr8	111496830	3168	LINC01608	453089	targeted sequencing	Péneau et al. 2021	33563643	Tumor
chr2	176589242	249	LNPK	199206	targeted sequencing	Péneau et al. 2021	33563643	Tumor
chr1	104688791	421	LOC100129138	-68993	targeted sequencing	Péneau et al. 2021	33563643	Tumor
chr12	66090558	2769	LOC100507065	-54293	targeted sequencing	Péneau et al. 2021	33563643	Tumor
chr3	125618388	NA	LOC101927056	-13241	targeted sequencing	Péneau et al. 2021	33563643	Tumor
chr4	68002701	838	LOC101927237	280176	targeted sequencing	Péneau et al. 2021	33563643	Tumor
chr4	134000894	2015	LOC101927359	14015	targeted sequencing	Péneau et al. 2021	33563643	Tumor
chr1	84200702	461	LOC101927587	0	targeted sequencing	Péneau et al. 2021	33563643	Tumor
chr5	164712207	1647	LOC102546299	-742072	targeted sequencing	Péneau et al. 2021	33563643	Tumor
chr17	25266947	630	LOC105371703	132468	targeted sequencing	Péneau et al. 2021	33563643	Tumor
chr6	22953132	1821	LOC105374972	-234868	targeted sequencing	Péneau et al. 2021	33563643	Tumor
chr19	37275774	2227	LOC728485	-7794	targeted sequencing	Péneau et al. 2021	33563643	Tumor
chr14	75052071	1282	LTBP2	0	targeted sequencing	Péneau et al. 2021	33563643	Tumor
chr20	13984097	1672	MACROD2	0	targeted sequencing	Péneau et al. 2021	33563643	Tumor
chr12	116306288	2986	MED13L	90093	targeted sequencing	Péneau et al. 2021	33563643	Tumor
chr16	76804657	1627	MIR4719	98176	targeted sequencing	Péneau et al. 2021	33563643	Tumor
chr1	566389	821	MIR6723	1316	targeted sequencing	Péneau et al. 2021	33563643	Tumor
chrUn_gl000220	145619	1818	MIR6724-1|MIR6724-2|MIR6724-3|MIR6724-4	3085	targeted sequencing	Péneau et al. 2021	33563643	Tumor
chr9	114041315	1117	MIR7702	-7821	targeted sequencing	Péneau et al. 2021	33563643	Tumor
chr11	73556056	116	MRPL48	0	targeted sequencing	Péneau et al. 2021	33563643	Tumor
chr10	135220593	620	MTG1	0	targeted sequencing	Péneau et al. 2021	33563643	Tumor
chr17	22254938	1629	MTRNR2L1	-230947	targeted sequencing	Péneau et al. 2021	33563643	Tumor
chr1	236126184	446	NID1	12948	targeted sequencing	Péneau et al. 2021	33563643	Tumor
chr14	33874100	782	NPAS3	0	targeted sequencing	Péneau et al. 2021	33563643	Tumor
chr6	23640058	3110	NRSN1	486356	targeted sequencing	Péneau et al. 2021	33563643	Tumor
chr5	41479671	1993	PLCXD3	0	targeted sequencing	Péneau et al. 2021	33563643	Tumor

Chromosome	Integration site in host genome	Integration site in virus genome	Gene (distance, bp)	Regions	Methods	Author	PMID	Sample
chr10	115520726	1173	PLEKHS1	0	targeted sequencing	Péneau et al. 2021	33563643	Tumor
chr3	144880691	1800	PLOD2	906429	targeted sequencing	Péneau et al. 2021	33563643	Tumor
chr12	102637459	1403	PMCH	-45836	targeted sequencing	Péneau et al. 2021	33563643	Tumor
chr14	45573347	569	PRPF39	0	targeted sequencing	Péneau et al. 2021	33563643	Tumor
chr17	65333497	2441	PSMD12	535	targeted sequencing	Péneau et al. 2021	33563643	Tumor
chr12	70923138	682	PTPRB	0	targeted sequencing	Péneau et al. 2021	33563643	Tumor
chr1	174869239	2640	RABGAP1L	0	targeted sequencing	Péneau et al. 2021	33563643	Tumor
chr19	6059698	2843	RFX2	0	targeted sequencing	Péneau et al. 2021	33563643	Tumor
chr18	40732660	2759	RIT2	-36856	targeted sequencing	Péneau et al. 2021	33563643	Tumor
chrM	6814	780	RNR2	-3373	targeted sequencing	Péneau et al. 2021	33563643	Tumor
chr1	64381745	2552	ROR1	0	targeted sequencing	Péneau et al. 2021	33563643	Tumor
chr1	48598433	1363	SKINT1L	0	targeted sequencing	Péneau et al. 2021	33563643	Tumor
chr8	117985142	1855	SLC30A8	0	targeted sequencing	Péneau et al. 2021	33563643	Tumor
chr12	119504280	2789	SRRM4	0	targeted sequencing	Péneau et al. 2021	33563643	Tumor
chr1	77445157	1905	ST6GALNAC5	0	targeted sequencing	Péneau et al. 2021	33563643	Tumor
chr1	172567841	730	SUCO	0	targeted sequencing	Péneau et al. 2021	33563643	Tumor
chr17	80745879	320	TBCD	0	targeted sequencing	Péneau et al. 2021	33563643	Tumor
chr2	121999157	1025	TFCP2L1	0	targeted sequencing	Péneau et al. 2021	33563643	Tumor
chr7	115749041	960	TFEC	-78073	targeted sequencing	Péneau et al. 2021	33563643	Tumor
chr15	76451596	899	TMEM266	0	targeted sequencing	Péneau et al. 2021	33563643	Tumor
chr21	10492985	821	TPTE	413202	targeted sequencing	Péneau et al. 2021	33563643	Tumor
chr12	49614965	563	TUBA1C	6744	targeted sequencing	Péneau et al. 2021	33563643	Tumor
chr11	18598214	1428	UEVLD	0	targeted sequencing	Péneau et al. 2021	33563643	Tumor
chr9	2401219	1878	VLDLR-AS1	134300	targeted sequencing	Péneau et al. 2021	33563643	Tumor
chr10	111194681	1240	XPNPEP1	429694	targeted sequencing	Péneau et al. 2021	33563643	Tumor
chr4	125700816	1420	ANKRD50	-66483	targeted sequencing	Péneau et al. 2021	33563643	Tumor
chr13	37131989	3098	CCNA1	-114494	targeted sequencing	Péneau et al. 2021	33563643	Tumor
chr4	155044735	1851	DCHS2	110693	targeted sequencing	Péneau et al. 2021	33563643	Tumor
chr1	121484554	485	EMBP1	-170868	targeted sequencing	Péneau et al. 2021	33563643	Tumor
chr10	42363387	NA	LOC441666	463820	targeted sequencing	Péneau et al. 2021	33563643	Tumor
chr10	42363489	NA	LOC441666	463820	targeted sequencing	Péneau et al. 2021	33563643	Tumor
chr4	29301326	1804	MIR4275	-480036	targeted sequencing	Péneau et al. 2021	33563643	Tumor
chr2	89879977	NA	MIR4436A	-767902	targeted sequencing	Péneau et al. 2021	33563643	Tumor
chr11	63486129	903	RTN3	0	targeted sequencing	Péneau et al. 2021	33563643	Tumor
chr8	53019140	2536	ST18	3799	targeted sequencing	Péneau et al. 2021	33563643	Tumor
chr14	93837133	2747	UNC79	0	targeted sequencing	Péneau et al. 2021	33563643	Tumor
chr13	52315889	1818	WDFY2	0	targeted sequencing	Péneau et al. 2021	33563643	Tumor
chr4	164945816	1450	MARCH1	0	targeted sequencing	Péneau et al. 2021	33563643	Tumor
chr4	164945817	3110	MARCH1	0	targeted sequencing	Péneau et al. 2021	33563643	Tumor
chr5	1295673	1745	TERT	0	targeted sequencing	Péneau et al. 2021	33563643	Tumor
chr5	1295696	1745	TERT	0	targeted sequencing	Péneau et al. 2021	33563643	Tumor
chr13	27142990	1819	WASF3	0	targeted sequencing	Péneau et al. 2021	33563643	Tumor
chr13	27142993	1879	WASF3	0	targeted sequencing	Péneau et al. 2021	33563643	Tumor
chrUn_gl000214	62578	1637	.	-1	targeted sequencing	Péneau et al. 2021	33563643	Tumor
chr16	48185826	1099	ABCC12	-4999	targeted sequencing	Péneau et al. 2021	33563643	Tumor
chr2	92291599	836	ACTR3BP2	-160855	targeted sequencing	Péneau et al. 2021	33563643	Tumor
chr5	162248701	1573	CCNG1	615876	targeted sequencing	Péneau et al. 2021	33563643	Tumor
chr12	77508534	1820	E2F7	-48588	targeted sequencing	Péneau et al. 2021	33563643	Tumor
chr4	110898596	3159	EGF	0	targeted sequencing	Péneau et al. 2021	33563643	Tumor
chr1	185843990	1859	HMCN1	0	targeted sequencing	Péneau et al. 2021	33563643	Tumor
chr13	103996604	2	LINC01309	80797	targeted sequencing	Péneau et al. 2021	33563643	Tumor
chr9	66971147	2166	LOC101928195|LOC101929583	0	targeted sequencing	Péneau et al. 2021	33563643	Tumor
chr9	69964123	1257	LOC101928381	-206203	targeted sequencing	Péneau et al. 2021	33563643	Tumor
chr5	4754361	2842	LOC101929153	19233	targeted sequencing	Péneau et al. 2021	33563643	Tumor
chr1	11635302	1918	LOC101929181	34286	targeted sequencing	Péneau et al. 2021	33563643	Tumor
chr14	85459010	1071	LOC105370605	394422	targeted sequencing	Péneau et al. 2021	33563643	Tumor
chr11	25075030	2652	LUZP2	0	targeted sequencing	Péneau et al. 2021	33563643	Tumor
chr22	50817588	2951	PPP6R2	0	targeted sequencing	Péneau et al. 2021	33563643	Tumor
chrM	7769	1355	RNR2	-4417	targeted sequencing	Péneau et al. 2021	33563643	Tumor
chr12	7051224	1752	ATN1	0	targeted sequencing	Péneau et al. 2021	33563643	Tumor
chr1	7695366	2157	CAMTA1	0	targeted sequencing	Péneau et al. 2021	33563643	Tumor
chr10	60733660	2225	LINC00844	25618	targeted sequencing	Péneau et al. 2021	33563643	Tumor
chr13	25776477	1824	LINC01053	-10964	targeted sequencing	Péneau et al. 2021	33563643	Tumor
chr13	25777130	1756	LINC01053	-12335	targeted sequencing	Péneau et al. 2021	33563643	Tumor
chr15	95096240	2463	MCTP2	-68803	targeted sequencing	Péneau et al. 2021	33563643	Tumor
chrX	145111213	1881	MIR891A	-1821	targeted sequencing	Péneau et al. 2021	33563643	Tumor
chr2	100083112	1004	REV1	0	targeted sequencing	Péneau et al. 2021	33563643	Tumor
chr2	92324210	2334	ACTR3BP2	-193326	targeted sequencing	Péneau et al. 2021	33563643	Tumor
chr12	131513160	1662	ADGRD1	0	targeted sequencing	Péneau et al. 2021	33563643	Tumor
chr7	134325921	2227	BPGM	5610	targeted sequencing	Péneau et al. 2021	33563643	Tumor
chr22	49826786	1307	C22orf34	0	targeted sequencing	Péneau et al. 2021	33563643	Tumor
chr2	152768772	169	CACNB4	0	targeted sequencing	Péneau et al. 2021	33563643	Tumor
chr14	80967859	1172	CEP128	0	targeted sequencing	Péneau et al. 2021	33563643	Tumor
chr1	9854863	2941	CLSTN1	0	targeted sequencing	Péneau et al. 2021	33563643	Tumor
chr8	140244719	1768	COL22A1	-318470	targeted sequencing	Péneau et al. 2021	33563643	Tumor
chr9	89943955	1082	DAPK1	168060	targeted sequencing	Péneau et al. 2021	33563643	Tumor
chr4	191043810	38	DBET	-54585	targeted sequencing	Péneau et al. 2021	33563643	Tumor
chr8	117601687	1388	EIF3H	55045	targeted sequencing	Péneau et al. 2021	33563643	Tumor
chr5	111785701	195	EPB41L4A-AS2	-28448	targeted sequencing	Péneau et al. 2021	33563643	Tumor
chr5	111785720	664	EPB41L4A-AS2	-28448	targeted sequencing	Péneau et al. 2021	33563643	Tumor
chr13	108405793	501	FAM155A	0	targeted sequencing	Péneau et al. 2021	33563643	Tumor
chr10	135524742	7	FRG2B	-84322	targeted sequencing	Péneau et al. 2021	33563643	Tumor
chr4	22651278	1784	GBA3	43259	targeted sequencing	Péneau et al. 2021	33563643	Tumor
chr4	44814023	775	GNPDA2	-85203	targeted sequencing	Péneau et al. 2021	33563643	Tumor
chr18	19018055	1844	GREB1L	0	targeted sequencing	Péneau et al. 2021	33563643	Tumor
chr8	43094880	1389	HGSNAT	-36755	targeted sequencing	Péneau et al. 2021	33563643	Tumor
chr8	43095180	1244	HGSNAT	-36755	targeted sequencing	Péneau et al. 2021	33563643	Tumor
chr7	110752092	687	IMMP2L|LRRN3	0	targeted sequencing	Péneau et al. 2021	33563643	Tumor
chr7	110752097	86	IMMP2L|LRRN3	0	targeted sequencing	Péneau et al. 2021	33563643	Tumor
chr3	49815028	325	IP6K1	0	targeted sequencing	Péneau et al. 2021	33563643	Tumor
chr10	126185493	1545	LHPP	0	targeted sequencing	Péneau et al. 2021	33563643	Tumor
chr8	46848300	2695	LINC00293	903912	targeted sequencing	Péneau et al. 2021	33563643	Tumor
chr8	46849187	790	LINC00293	902959	targeted sequencing	Péneau et al. 2021	33563643	Tumor
chr8	58214082	1380	LINC00588	-16657	targeted sequencing	Péneau et al. 2021	33563643	Tumor

Chromosome	Integration site in host genome	Integration site in virus genome	Gene (distance, bp)	Regions	Methods	Author	PMID	Sample
chr19	28782016	986	LOC100420587	144091	targeted sequencing	Péneau et al. 2021	33563643	Tumor
chr8	80792904	402	LOC101927040	-8813	targeted sequencing	Péneau et al. 2021	33563643	Tumor
chr9	132019585	1749	LOC101929331	25110	targeted sequencing	Péneau et al. 2021	33563643	Tumor
chr10	42393247	2134	LOC441666	433878	targeted sequencing	Péneau et al. 2021	33563643	Tumor
chr10	42600079	582	LOC441666	227235	targeted sequencing	Péneau et al. 2021	33563643	Tumor
chr1	70235043	1488	LRRC7	0	targeted sequencing	Péneau et al. 2021	33563643	Tumor
chr1	25397109	602	MIR4425	-46856	targeted sequencing	Péneau et al. 2021	33563643	Tumor
chr16	48496851	1609	MIR5095	0	targeted sequencing	Péneau et al. 2021	33563643	Tumor
chrUn_gl000220	140567	2860	MIR6724-1|MIR6724-2|MIR6724-3|MIR6724-4	7971	targeted sequencing	Péneau et al. 2021	33563643	Tumor
chr8	89239452	993	MMP16	0	targeted sequencing	Péneau et al. 2021	33563643	Tumor
chr22	50564423	1827	MOV10L1	0	targeted sequencing	Péneau et al. 2021	33563643	Tumor
chr21	33659213	608	MRAP	4911	targeted sequencing	Péneau et al. 2021	33563643	Tumor
chr12	78564244	360	NAV3	0	targeted sequencing	Péneau et al. 2021	33563643	Tumor
chr10	64769136	714	NRBF2	123871	targeted sequencing	Péneau et al. 2021	33563643	Tumor
chr4	37365647	350	NWD2	0	targeted sequencing	Péneau et al. 2021	33563643	Tumor
chr20	8158519	295	PLCB1	0	targeted sequencing	Péneau et al. 2021	33563643	Tumor
chr18	51827357	952	POLI	-2753	targeted sequencing	Péneau et al. 2021	33563643	Tumor
chr14	29691052	630	PRKD1	354497	targeted sequencing	Péneau et al. 2021	33563643	Tumor
chr10	53227535	200	PRKG1	0	targeted sequencing	Péneau et al. 2021	33563643	Tumor
chr8	85781571	1808	RALYL	0	targeted sequencing	Péneau et al. 2021	33563643	Tumor
chr3	76587596	322	ROBO2	501554	targeted sequencing	Péneau et al. 2021	33563643	Tumor
chrX	20337627	730	RPS6KA3	-52877	targeted sequencing	Péneau et al. 2021	33563643	Tumor
chrX	61716847	2115	SPIN4	849597	targeted sequencing	Péneau et al. 2021	33563643	Tumor
chrX	61717278	3090	SPIN4	849597	targeted sequencing	Péneau et al. 2021	33563643	Tumor
chr5	147560429	851	SPINK14	-5247	targeted sequencing	Péneau et al. 2021	33563643	Tumor
chr6	152753297	1414	SYNE1	0	targeted sequencing	Péneau et al. 2021	33563643	Tumor
chr1	234533630	1747	TARBP1	0	targeted sequencing	Péneau et al. 2021	33563643	Tumor
chrX	109740102	1140	TDGF1P3	23438	targeted sequencing	Péneau et al. 2021	33563643	Tumor
chr4	2758249	1779	TNIP2	-146	targeted sequencing	Péneau et al. 2021	33563643	Tumor
chr1	40909658	965	ZFP69B	6679	targeted sequencing	Péneau et al. 2021	33563643	Tumor
chr19	48792428	1479	ZNF114	-1420	targeted sequencing	Péneau et al. 2021	33563643	Tumor
chr19	21289533	1481	ZNF714	0	targeted sequencing	Péneau et al. 2021	33563643	Tumor
chr14	37739890	1808	MIPOL1	0	targeted sequencing	Péneau et al. 2021	33563643	Tumor
chr14	37739895	1837	MIPOL1	0	targeted sequencing	Péneau et al. 2021	33563643	Tumor
chr3	153066929	NA	C3orf79	135355	targeted sequencing	Péneau et al. 2021	33563643	Tumor
chr4	81855207	1935	C4orf22	0	targeted sequencing	Péneau et al. 2021	33563643	Tumor
chr4	81855222	1813	C4orf22	0	targeted sequencing	Péneau et al. 2021	33563643	Tumor
chr5	21034534	1821	GUSBP1	425055	targeted sequencing	Péneau et al. 2021	33563643	Tumor
chr6	97475791	1819	KLHL32	0	targeted sequencing	Péneau et al. 2021	33563643	Tumor
chr6	97477111	2239	KLHL32	0	targeted sequencing	Péneau et al. 2021	33563643	Tumor
chr13	54205051	1819	LINC00558	184503	targeted sequencing	Péneau et al. 2021	33563643	Tumor
chr2	128327483	2227	LOC105373609|MYO7B	0	targeted sequencing	Péneau et al. 2021	33563643	Tumor
chr12	93365992	1422	LOC643339	31258	targeted sequencing	Péneau et al. 2021	33563643	Tumor
chr18	57624698	1972	PMAIP1	-53160	targeted sequencing	Péneau et al. 2021	33563643	Tumor
chr8	70485320	438	SULF1	0	targeted sequencing	Péneau et al. 2021	33563643	Tumor
chr8	70485352	1171	SULF1	0	targeted sequencing	Péneau et al. 2021	33563643	Tumor
chr5	1295173	1743	TERT	0	targeted sequencing	Péneau et al. 2021	33563643	Tumor
chr5	1295198	2457	TERT	0	targeted sequencing	Péneau et al. 2021	33563643	Tumor
chr11	57496347	2261	TMX2|TMX2-CTNND1	0	targeted sequencing	Péneau et al. 2021	33563643	Tumor
chr11	57496803	2734	TMX2|TMX2-CTNND1	0	targeted sequencing	Péneau et al. 2021	33563643	Tumor
chr18	66116030	599	TMX3	224895	targeted sequencing	Péneau et al. 2021	33563643	Tumor
chr6	94810047	454	TSG1	-323643	targeted sequencing	Péneau et al. 2021	33563643	Tumor
chr9_gl000199_random	34440	1564	.	-1	targeted sequencing	Péneau et al. 2021	33563643	Tumor
chr9_gl000199_random	167152	1942	.	-1	targeted sequencing	Péneau et al. 2021	33563643	Tumor
chr6	63780426	894	LGSN	204667	targeted sequencing	Péneau et al. 2021	33563643	Tumor
chr6	63780429	881	LGSN	204667	targeted sequencing	Péneau et al. 2021	33563643	Tumor
chr4	12486355	578	LOC101929019	-233446	targeted sequencing	Péneau et al. 2021	33563643	Tumor
chr2	230505491	2605	DNER	0	targeted sequencing	Péneau et al. 2021	33563643	Tumor
chr1	226008218	916	EPHX1	0	targeted sequencing	Péneau et al. 2021	33563643	Tumor
chr4	174204815	607	GALNT7	0	targeted sequencing	Péneau et al. 2021	33563643	Tumor
chr4	174204818	1472	GALNT7	0	targeted sequencing	Péneau et al. 2021	33563643	Tumor
chr14	86547072	3170	LOC101928767	0	targeted sequencing	Péneau et al. 2021	33563643	Tumor
chr11	48703017	2368	OR4A47	-191180	targeted sequencing	Péneau et al. 2021	33563643	Tumor
chr11	51579635	1581	OR4C46	-63167	targeted sequencing	Péneau et al. 2021	33563643	Tumor
chr15	87898606	1825	LINC00052	220836	targeted sequencing	Péneau et al. 2021	33563643	Tumor
chr15	87898614	1828	LINC00052	220836	targeted sequencing	Péneau et al. 2021	33563643	Tumor
chr9	71578345	3041	LOC101927069|PIP5K1B	0	targeted sequencing	Péneau et al. 2021	33563643	Tumor
chr9	71578376	1783	LOC101927069|PIP5K1B	0	targeted sequencing	Péneau et al. 2021	33563643	Tumor
chr9	111542592	1258	ACTL7B	73746	targeted sequencing	Péneau et al. 2021	33563643	Tumor
chr9	111542603	1594	ACTL7B	73746	targeted sequencing	Péneau et al. 2021	33563643	Tumor
chr9	13716420	435	LINC00583	211520	targeted sequencing	Péneau et al. 2021	33563643	Tumor
chr17	8764501	698	PIK3R6	0	targeted sequencing	Péneau et al. 2021	33563643	Tumor
chr14	89379594	739	TTC8	-34596	targeted sequencing	Péneau et al. 2021	33563643	Tumor
chr14	89400057	455	TTC8	-55184	targeted sequencing	Péneau et al. 2021	33563643	Tumor
chr21	47561354	2744	FTCD	0	targeted sequencing	Péneau et al. 2021	33563643	Tumor
chr8	34835347	1782	LINC01288	-113031	targeted sequencing	Péneau et al. 2021	33563643	Tumor
chr18	37400688	1864	MIR924HG	-68614	targeted sequencing	Péneau et al. 2021	33563643	Tumor
chr1	249240391	1625	PGBD2	-26882	targeted sequencing	Péneau et al. 2021	33563643	Tumor
chr5	120304038	250	PRR16	-281013	targeted sequencing	Péneau et al. 2021	33563643	Tumor
chr3	157821110	1813	SHOX2	0	targeted sequencing	Péneau et al. 2021	33563643	Tumor
chr5	1295264	1807	TERT	0	targeted sequencing	Péneau et al. 2021	33563643	Tumor
chr5	1295270	681	TERT	0	targeted sequencing	Péneau et al. 2021	33563643	Tumor
chr5	14771771	1828	ANKH	0	targeted sequencing	Péneau et al. 2021	33563643	Tumor
chr1	64902110	1569	CACHD1	34366	targeted sequencing	Péneau et al. 2021	33563643	Tumor
chr19	30300749	2979	CCNE1	2152	targeted sequencing	Péneau et al. 2021	33563643	Tumor
chr19	30301935	1856	CCNE1	275	targeted sequencing	Péneau et al. 2021	33563643	Tumor
chr14	90044536	1738	FOXN3	0	targeted sequencing	Péneau et al. 2021	33563643	Tumor
chr14	90132717	1828	FOXN3	-46765	targeted sequencing	Péneau et al. 2021	33563643	Tumor
chr5	1026641	587	NKD2	0	targeted sequencing	Péneau et al. 2021	33563643	Tumor
chr6	57498757	880	PRIM2	0	targeted sequencing	Péneau et al. 2021	33563643	Tumor
chr5	1295211	1496	TERT	0	targeted sequencing	Péneau et al. 2021	33563643	Tumor
chr2	114360363	1786	DDX11L2	0	targeted sequencing	Péneau et al. 2021	33563643	Tumor
chr8	69868987	1166	LINC01592	0	targeted sequencing	Péneau et al. 2021	33563643	Tumor
chr8	69869029	1238	LINC01592	0	targeted sequencing	Péneau et al. 2021	33563643	Tumor

Chromosome	Integration site in host genome	Integration site in virus genome	Gene (distance, bp)	Regions	Methods	Author	PMID	Sample
chr1	120586797	1417	NOTCH2	0	targeted sequencing	Péneau et al. 2021	33563643	Tumor
chr7	12959631	1823	ARL4A	-229073	targeted sequencing	Péneau et al. 2021	33563643	Tumor
chr21	42650329	1839	BACE2	0	targeted sequencing	Péneau et al. 2021	33563643	Tumor
chr1	58750886	939	DAB1	-34553	targeted sequencing	Péneau et al. 2021	33563643	Tumor
chr4	84694036	263	GPAT3	-166910	targeted sequencing	Péneau et al. 2021	33563643	Tumor
chr7	81402756	1822	HGF	-2473	targeted sequencing	Péneau et al. 2021	33563643	Tumor
chr7	81423201	1827	HGF	-23687	targeted sequencing	Péneau et al. 2021	33563643	Tumor
chr11	122925965	1828	HSPA8	2235	targeted sequencing	Péneau et al. 2021	33563643	Tumor
chr8	78185467	2999	LOC102724874	177275	targeted sequencing	Péneau et al. 2021	33563643	Tumor
chr9	85323879	2461	RASEF	270444	targeted sequencing	Péneau et al. 2021	33563643	Tumor
chr5	39058187	2815	RICTOR	0	targeted sequencing	Péneau et al. 2021	33563643	Tumor
chrM	1792	558	RNR2	0	targeted sequencing	Péneau et al. 2021	33563643	Tumor
chr5	171866667	1825	SH3PXD2B	0	targeted sequencing	Péneau et al. 2021	33563643	Tumor
chr5	171866713	1819	SH3PXD2B	0	targeted sequencing	Péneau et al. 2021	33563643	Tumor
chr9	139276169	3033	SNAPC4	0	targeted sequencing	Péneau et al. 2021	33563643	Tumor
chr1	175257558	2859	TNR	34377	targeted sequencing	Péneau et al. 2021	33563643	Tumor
chr19	30277765	344	CCNE1	24530	targeted sequencing	Péneau et al. 2021	33563643	Tumor
chr19	30277783	2592	CCNE1	24530	targeted sequencing	Péneau et al. 2021	33563643	Tumor
chr11	90528548	2785	DISC1FP1	0	targeted sequencing	Péneau et al. 2021	33563643	Tumor
chr8	17214555	201	MTMR7	0	targeted sequencing	Péneau et al. 2021	33563643	Tumor
chr10	115394751	2899	NRAP	0	targeted sequencing	Péneau et al. 2021	33563643	Tumor
chr7	83385331	2805	SEMA3E	-106852	targeted sequencing	Péneau et al. 2021	33563643	Tumor
chr2	83944257	339	FUNDC2P2	573549	targeted sequencing	Péneau et al. 2021	33563643	Tumor
chr2	113896762	1816	IL1RN	-4971	targeted sequencing	Péneau et al. 2021	33563643	Tumor
chr2	113896783	1849	IL1RN	-4971	targeted sequencing	Péneau et al. 2021	33563643	Tumor
chr17	60095540	1787	MED13	0	targeted sequencing	Péneau et al. 2021	33563643	Tumor
chr3	133923472	NA	RYK	0	targeted sequencing	Péneau et al. 2021	33563643	Tumor
chr11	9090467	1835	SCUBE2	0	targeted sequencing	Péneau et al. 2021	33563643	Tumor
chr1	2940924	2338	ACTRT2	-1332	targeted sequencing	Péneau et al. 2021	33563643	Tumor
chr16	46405392	1657	ANKRD26P1	97287	targeted sequencing	Péneau et al. 2021	33563643	Tumor
chr16	46405573	1826	ANKRD26P1	97287	targeted sequencing	Péneau et al. 2021	33563643	Tumor
chrX	62927823	159	ARHGEF9	0	targeted sequencing	Péneau et al. 2021	33563643	Tumor
chr9	97629923	2058	C9orf3	0	targeted sequencing	Péneau et al. 2021	33563643	Tumor
chr4	191040854	369	DBET	-51683	targeted sequencing	Péneau et al. 2021	33563643	Tumor
chr4	191044166	369	DBET	-55030	targeted sequencing	Péneau et al. 2021	33563643	Tumor
chr2	26624134	238	DRC1	646	targeted sequencing	Péneau et al. 2021	33563643	Tumor
chr1	236598273	6	EDARADD	0	targeted sequencing	Péneau et al. 2021	33563643	Tumor
chr4	22802100	2825	GBA3	0	targeted sequencing	Péneau et al. 2021	33563643	Tumor
chr4	82862142	1001	HNRNPD	412325	targeted sequencing	Péneau et al. 2021	33563643	Tumor
chrX	132021288	2700	HS6ST2	0	targeted sequencing	Péneau et al. 2021	33563643	Tumor
chrX	113773974	1492	HTR2C	44381	targeted sequencing	Péneau et al. 2021	33563643	Tumor
chr8	12807487	1834	KIAA1456	0	targeted sequencing	Péneau et al. 2021	33563643	Tumor
chr8	12807500	1806	KIAA1456	0	targeted sequencing	Péneau et al. 2021	33563643	Tumor
chr2	35138859	386	LINC01320	-191229	targeted sequencing	Péneau et al. 2021	33563643	Tumor
chr16	51623612	1659	LINC01571	172818	targeted sequencing	Péneau et al. 2021	33563643	Tumor
chr12	95490	246	LOC100288778	-3923	targeted sequencing	Péneau et al. 2021	33563643	Tumor
chr7	20007547	1532	LOC101927668	0	targeted sequencing	Péneau et al. 2021	33563643	Tumor
chr1	116674004	1270	MAB21L3	0	targeted sequencing	Péneau et al. 2021	33563643	Tumor
chr14	38013705	2310	MIPOL1	0	targeted sequencing	Péneau et al. 2021	33563643	Tumor
chr2	56186932	2740	MIR217HG	3443	targeted sequencing	Péneau et al. 2021	33563643	Tumor
chrX	120934674	1699	MIR3672	-429767	targeted sequencing	Péneau et al. 2021	33563643	Tumor
chr18	35682340	1725	MIR4318	-444358	targeted sequencing	Péneau et al. 2021	33563643	Tumor
chr18	35686078	2866	MIR4318	-448900	targeted sequencing	Péneau et al. 2021	33563643	Tumor
chr12	62942336	1075	MON2	0	targeted sequencing	Péneau et al. 2021	33563643	Tumor
chr8	71090821	2150	NCOA2	0	targeted sequencing	Péneau et al. 2021	33563643	Tumor
chr1	162032706	36	NOS1AP	6721	targeted sequencing	Péneau et al. 2021	33563643	Tumor
chr1	164705812	2062	PBX1	0	targeted sequencing	Péneau et al. 2021	33563643	Tumor
chr8	79043857	2274	PKIA	384479	targeted sequencing	Péneau et al. 2021	33563643	Tumor
chr1	171578076	2110	PRRC2C	-15034	targeted sequencing	Péneau et al. 2021	33563643	Tumor
chr21	44780995	2007	TCONS_00029157	0	targeted sequencing	Péneau et al. 2021	33563643	Tumor
chr2	62801512	1031	TMEM17	-67728	targeted sequencing	Péneau et al. 2021	33563643	Tumor
chr1	112993524	1825	CTTNBP2NL	0	targeted sequencing	Péneau et al. 2021	33563643	Tumor
chr1	112995366	1900	CTTNBP2NL	0	targeted sequencing	Péneau et al. 2021	33563643	Tumor
chr18	67242131	1824	DOK6	0	targeted sequencing	Péneau et al. 2021	33563643	Tumor
chr5	137894962	1819	HSPA9	0	targeted sequencing	Péneau et al. 2021	33563643	Tumor
chr13	98588350	1127	IPO5	17207	targeted sequencing	Péneau et al. 2021	33563643	Tumor
chrX	46901777	2292	JADE3	0	targeted sequencing	Péneau et al. 2021	33563643	Tumor
chr3	123903874	1796	KALRN	0	targeted sequencing	Péneau et al. 2021	33563643	Tumor
chr3	123903887	2078	KALRN	0	targeted sequencing	Péneau et al. 2021	33563643	Tumor
chr15	57598167	1778	LINC00926	0	targeted sequencing	Péneau et al. 2021	33563643	Tumor
chr15	57601482	2190	LINC00926	-1515	targeted sequencing	Péneau et al. 2021	33563643	Tumor
chr3	27820618	1805	LOC100996624	51395	targeted sequencing	Péneau et al. 2021	33563643	Tumor
chr3	27820641	1771	LOC100996624	51395	targeted sequencing	Péneau et al. 2021	33563643	Tumor
chr5	70384349	1727	LOC647859	0	targeted sequencing	Péneau et al. 2021	33563643	Tumor
chr14	38003035	2422	MIPOL1	0	targeted sequencing	Péneau et al. 2021	33563643	Tumor
chr5	68835219	47	OCLN	0	targeted sequencing	Péneau et al. 2021	33563643	Tumor
chrUn_gl000220	155949	2886	RNA5-8S5	48	targeted sequencing	Péneau et al. 2021	33563643	Tumor
chr14	38110484	1481	TTC6	0	targeted sequencing	Péneau et al. 2021	33563643	Tumor
chr1	20519472	1829	UBXN10	0	targeted sequencing	Péneau et al. 2021	33563643	Tumor
chr1	20528464	1451	UBXN10	-5923	targeted sequencing	Péneau et al. 2021	33563643	Tumor
chr5	148575276	511	ABLIM3	0	targeted sequencing	Péneau et al. 2021	33563643	Tumor
chr1	82220362	1480	ADGRL2	0	targeted sequencing	Péneau et al. 2021	33563643	Tumor
chr14	94427302	1928	ASB2	0	targeted sequencing	Péneau et al. 2021	33563643	Tumor
chr4	169915840	153	CBR4	0	targeted sequencing	Péneau et al. 2021	33563643	Tumor
chr5	19334366	2853	CDH18	138657	targeted sequencing	Péneau et al. 2021	33563643	Tumor
chr5	26529139	780	CDH9	351570	targeted sequencing	Péneau et al. 2021	33563643	Tumor
chr9	93443455	503	DIRAS2	-37970	targeted sequencing	Péneau et al. 2021	33563643	Tumor
chr2	242618599	1166	DTYMK	0	targeted sequencing	Péneau et al. 2021	33563643	Tumor
chr10	85718280	512	GHITM	180693	targeted sequencing	Péneau et al. 2021	33563643	Tumor
chr3	34473155	2960	LOC101928135	444134	targeted sequencing	Péneau et al. 2021	33563643	Tumor
chr6	68873032	498	LOC101928280	63603	targeted sequencing	Péneau et al. 2021	33563643	Tumor
chr4	12376638	380	LOC101929019	-123729	targeted sequencing	Péneau et al. 2021	33563643	Tumor
chr11	29092363	1809	MIR8068	-592626	targeted sequencing	Péneau et al. 2021	33563643	Tumor
chr11	29092366	2291	MIR8068	-592626	targeted sequencing	Péneau et al. 2021	33563643	Tumor

Chromosome	Integration site in host genome	Integration site in virus genome	Gene (distance, bp)	Regions	Methods	Author	PMID	Sample
chr9	109029763	1188	MIR8081	333446	targeted sequencing	Péneau et al. 2021	33563643	Tumor
chr1	32698553	570	MTMR9LP	0	targeted sequencing	Péneau et al. 2021	33563643	Tumor
chr11	51579636	228	OR4C46	-63314	targeted sequencing	Péneau et al. 2021	33563643	Tumor
chr2	208930562	2765	PLEKHM3	-40131	targeted sequencing	Péneau et al. 2021	33563643	Tumor
chr7	39538742	2503	POU6F2	-33699	targeted sequencing	Péneau et al. 2021	33563643	Tumor
chr7	39538831	1928	POU6F2	-33699	targeted sequencing	Péneau et al. 2021	33563643	Tumor
chr5	145954357	3057	PPP2R2B	14489	targeted sequencing	Péneau et al. 2021	33563643	Tumor
chr10	98819640	3052	SLIT1	0	targeted sequencing	Péneau et al. 2021	33563643	Tumor
chr13	86445840	1524	SLITRK6	-72357	targeted sequencing	Péneau et al. 2021	33563643	Tumor
chr3	4413021	163	SUMF1	0	targeted sequencing	Péneau et al. 2021	33563643	Tumor
chr8	33414976	1756	RNF122	0	targeted sequencing	Péneau et al. 2021	33563643	Tumor
chr8	33414988	2765	RNF122	0	targeted sequencing	Péneau et al. 2021	33563643	Tumor
chr2	40844109	2148	SLC8A1	-104025	targeted sequencing	Péneau et al. 2021	33563643	Tumor
chr2	40844166	1870	SLC8A1	-104025	targeted sequencing	Péneau et al. 2021	33563643	Tumor
chr19	22418824	1852	ZNF676	-38481	targeted sequencing	Péneau et al. 2021	33563643	Tumor
chr19	22418863	1819	ZNF676	-38481	targeted sequencing	Péneau et al. 2021	33563643	Tumor
chr19	49468588	1912	FTL	0	targeted sequencing	Péneau et al. 2021	33563643	Tumor
chr19	49468821	789	FTL	0	targeted sequencing	Péneau et al. 2021	33563643	Tumor
chr19	36213724	2709	KMT2B	0	targeted sequencing	Péneau et al. 2021	33563643	Tumor
chr19	36213744	1828	KMT2B	0	targeted sequencing	Péneau et al. 2021	33563643	Tumor
chr2	82242937	1980	LOC100507201	-548228	targeted sequencing	Péneau et al. 2021	33563643	Tumor
chr2	82242938	2064	LOC100507201	-548228	targeted sequencing	Péneau et al. 2021	33563643	Tumor
chr22	20655921	2420	LOC101927859|LOC729461	0	targeted sequencing	Péneau et al. 2021	33563643	Tumor
chr5	8070686	1805	MTRR	-168803	targeted sequencing	Péneau et al. 2021	33563643	Tumor
chr10	51518497	277	TIMM23B	0	targeted sequencing	Péneau et al. 2021	33563643	Tumor
chr4	184663656	1786	TRAPPC11	-28212	targeted sequencing	Péneau et al. 2021	33563643	Tumor
chr4	184663741	1835	TRAPPC11	-28212	targeted sequencing	Péneau et al. 2021	33563643	Tumor
chr8	39339457	1833	ADAM3A	0	targeted sequencing	Péneau et al. 2021	33563643	Tumor
chrX	155260299	2526	DDX11L16	-2353	targeted sequencing	Péneau et al. 2021	33563643	Tumor
chr10	135524749	1284	FRG2B	-84304	targeted sequencing	Péneau et al. 2021	33563643	Tumor
chr10	42599692	1622	LOC441666	227622	targeted sequencing	Péneau et al. 2021	33563643	Tumor
chr18	10117937	1695	VAPA	-157785	targeted sequencing	Péneau et al. 2021	33563643	Tumor
chr18	10117937	2309	VAPA	-157785	targeted sequencing	Péneau et al. 2021	33563643	Tumor
chr1	196604078	443	CFH	16818	targeted sequencing	Péneau et al. 2021	33563643	Tumor
chr17	13216586	1222	HS3ST3A1	180577	targeted sequencing	Péneau et al. 2021	33563643	Tumor
chr17	29733864	128	RAB11FIP4	0	targeted sequencing	Péneau et al. 2021	33563643	Tumor
chr17	29733864	3182	RAB11FIP4	0	targeted sequencing	Péneau et al. 2021	33563643	Tumor
chr17	5261894	622	RABEP1	0	targeted sequencing	Péneau et al. 2021	33563643	Tumor
chr1	246023401	1805	SMYD3	0	targeted sequencing	Péneau et al. 2021	33563643	Tumor
chr1	246023411	2472	SMYD3	0	targeted sequencing	Péneau et al. 2021	33563643	Tumor
chr12	109588923	639	ACACB	0	targeted sequencing	Péneau et al. 2021	33563643	Tumor
chr8	41598095	846	ANK1	0	targeted sequencing	Péneau et al. 2021	33563643	Tumor
chr10	35623330	2087	CCNY	0	targeted sequencing	Péneau et al. 2021	33563643	Tumor
chr10	35623333	1830	CCNY	0	targeted sequencing	Péneau et al. 2021	33563643	Tumor
chr7	111675576	1308	DOCK4	0	targeted sequencing	Péneau et al. 2021	33563643	Tumor
chr2	48483428	547	FOXN2	58232	targeted sequencing	Péneau et al. 2021	33563643	Tumor
chr12	75187491	1893	KCNC2	246230	targeted sequencing	Péneau et al. 2021	33563643	Tumor
chr2	202938850	2688	KIAA2012	0	targeted sequencing	Péneau et al. 2021	33563643	Tumor
chr10	42599696	1045	LOC441666	227509	targeted sequencing	Péneau et al. 2021	33563643	Tumor
chr2	140817735	1831	LRP1B	171261	targeted sequencing	Péneau et al. 2021	33563643	Tumor
chr6	12562917	1656	PHACTR1	153851	targeted sequencing	Péneau et al. 2021	33563643	Tumor
chr9	98331098	562	PTCH1	-51851	targeted sequencing	Péneau et al. 2021	33563643	Tumor
chr3	11029255	1463	SLC6A1	4963	targeted sequencing	Péneau et al. 2021	33563643	Tumor
chr5	75611413	526	SV2C	0	targeted sequencing	Péneau et al. 2021	33563643	Tumor
chr5	1293360	1496	TERT	0	targeted sequencing	Péneau et al. 2021	33563643	Tumor
chr5	1295131	1805	TERT	0	targeted sequencing	Péneau et al. 2021	33563643	Tumor
chr5	1295163	990	TERT	0	targeted sequencing	Péneau et al. 2021	33563643	Tumor
chr8	41661615	1840	ANK1	0	targeted sequencing	Péneau et al. 2021	33563643	Tumor
chr4	186350310	1761	C4orf47	0	targeted sequencing	Péneau et al. 2021	33563643	Tumor
chr4	186350319	1845	C4orf47	0	targeted sequencing	Péneau et al. 2021	33563643	Tumor
chr8	69765891	156	C8orf34	-34631	targeted sequencing	Péneau et al. 2021	33563643	Tumor
chr1	34439587	2507	CSMD2	0	targeted sequencing	Péneau et al. 2021	33563643	Tumor
chr1	34439600	1801	CSMD2	0	targeted sequencing	Péneau et al. 2021	33563643	Tumor
chr8	43093769	1606	HGSNAT	-35799	targeted sequencing	Péneau et al. 2021	33563643	Tumor
chr19	27732296	1784	LINC00662	549105	targeted sequencing	Péneau et al. 2021	33563643	Tumor
chr17	22261070	928	MTRNR2L1	-237079	targeted sequencing	Péneau et al. 2021	33563643	Tumor
chr13	57150964	1852	PRR20A|PRR20B|PRR20C|PRR20D|PRR20E	563903	targeted sequencing	Péneau et al. 2021	33563643	Tumor
chr11	10019010	3195	SBF2	0	targeted sequencing	Péneau et al. 2021	33563643	Tumor
chr2	686834	1627	TMEM18	-9395	targeted sequencing	Péneau et al. 2021	33563643	Tumor
chr11	115231502	2746	CADM1	0	targeted sequencing	Péneau et al. 2021	33563643	Tumor
chr11	125444021	2169	EI24|STT3A-AS1	0	targeted sequencing	Péneau et al. 2021	33563643	Tumor
chrX	73287696	447	FTX|JPX	0	targeted sequencing	Péneau et al. 2021	33563643	Tumor
chr7	126003828	14	GRM8	74824	targeted sequencing	Péneau et al. 2021	33563643	Tumor
chr7	126006964	1755	GRM8	70855	targeted sequencing	Péneau et al. 2021	33563643	Tumor
chr17	2398488	1387	METTL16	0	targeted sequencing	Péneau et al. 2021	33563643	Tumor
chr5	1295363	1824	TERT	0	targeted sequencing	Péneau et al. 2021	33563643	Tumor
chr5	1295555	2330	TERT	0	targeted sequencing	Péneau et al. 2021	33563643	Tumor
chr2	121952657	1807	TFCP2L1	20806	targeted sequencing	Péneau et al. 2021	33563643	Tumor
chr2	121952660	2247	TFCP2L1	20806	targeted sequencing	Péneau et al. 2021	33563643	Tumor
chr2	96643318	1815	ANKRD36C	0	targeted sequencing	Péneau et al. 2021	33563643	Tumor
chr1	249239964	2192	PGBD2	-26158	targeted sequencing	Péneau et al. 2021	33563643	Tumor
chr9_gl000199_random	88084	2686	.	-1	targeted sequencing	Péneau et al. 2021	33563643	Tumor
chr21	11142761	1826	BAGE	-43382	targeted sequencing	Péneau et al. 2021	33563643	Tumor
chr21	11143650	654	BAGE	-44713	targeted sequencing	Péneau et al. 2021	33563643	Tumor
chr1	121484654	654	EMBP1	-170968	targeted sequencing	Péneau et al. 2021	33563643	Tumor
chr2	75954667	1122	GCFC2	-16023	targeted sequencing	Péneau et al. 2021	33563643	Tumor
chr2	75954703	1225	GCFC2	-16023	targeted sequencing	Péneau et al. 2021	33563643	Tumor
chr14	93483547	1826	ITPK1	0	targeted sequencing	Péneau et al. 2021	33563643	Tumor
chr8	83233839	2672	SNX16	-479210	targeted sequencing	Péneau et al. 2021	33563643	Tumor
chr8	83233844	1827	SNX16	-479210	targeted sequencing	Péneau et al. 2021	33563643	Tumor
chr5	1295337	1822	TERT	0	targeted sequencing	Péneau et al. 2021	33563643	Tumor
chr5	1295347	154	TERT	0	targeted sequencing	Péneau et al. 2021	33563643	Tumor
chrX	64158483	1250	ZC4H2	0	targeted sequencing	Péneau et al. 2021	33563643	Tumor
chr2	92316779	1857	ACTR3BP2	-185681	targeted sequencing	Péneau et al. 2021	33563643	Tumor

Chromosome	Integration site in host genome	Integration site in virus genome	Gene (distance, bp)	Regions	Methods	Author	PMID	Sample
chr17	6910194	1845	ALOX12|ALOX12-AS1	0	targeted sequencing	Péneau et al. 2021	33563643	Tumor
chr17	6910248	1890	ALOX12|ALOX12-AS1	0	targeted sequencing	Péneau et al. 2021	33563643	Tumor
chr17	7006174	1826	ASGR2	0	targeted sequencing	Péneau et al. 2021	33563643	Tumor
chr10	18668613	1821	CACNB2	0	targeted sequencing	Péneau et al. 2021	33563643	Tumor
chr10	18668623	1821	CACNB2	0	targeted sequencing	Péneau et al. 2021	33563643	Tumor
chr2	211442777	1723	CPS1	0	targeted sequencing	Péneau et al. 2021	33563643	Tumor
chr12	77811850	1658	E2F7	-352490	targeted sequencing	Péneau et al. 2021	33563643	Tumor
chr5	93216078	791	FAM172A	0	targeted sequencing	Péneau et al. 2021	33563643	Tumor
chr2	178037767	672	HNRNPA3	39340	targeted sequencing	Péneau et al. 2021	33563643	Tumor
chr13	84943249	2063	LINC00333	0	targeted sequencing	Péneau et al. 2021	33563643	Tumor
chr19	27833635	1807	LINC00662	447664	targeted sequencing	Péneau et al. 2021	33563643	Tumor
chr6	113428780	1807	LOC101927686	515141	targeted sequencing	Péneau et al. 2021	33563643	Tumor
chr6	113429109	1818	LOC101927686	515141	targeted sequencing	Péneau et al. 2021	33563643	Tumor
chr5	1309652	2718	MIR4457	-160	targeted sequencing	Péneau et al. 2021	33563643	Tumor
chr5	1313167	2972	MIR4457	-3231	targeted sequencing	Péneau et al. 2021	33563643	Tumor
chr11	30583371	1882	MPPED2	0	targeted sequencing	Péneau et al. 2021	33563643	Tumor
chr11	30583374	1828	MPPED2	0	targeted sequencing	Péneau et al. 2021	33563643	Tumor
chr4	71337498	1819	MUC7	0	targeted sequencing	Péneau et al. 2021	33563643	Tumor
chr8	63248198	1617	NKAIN3	0	targeted sequencing	Péneau et al. 2021	33563643	Tumor
chr5	1295248	1826	TERT	0	targeted sequencing	Péneau et al. 2021	33563643	Tumor
chr4	167188073	1964	TLL1	-161897	targeted sequencing	Péneau et al. 2021	33563643	Tumor
chr5	776875	1600	ZDHHC11	18034	targeted sequencing	Péneau et al. 2021	33563643	Tumor
chr8	43094764	NA	HGSNAT	-36794	targeted sequencing	Péneau et al. 2021	33563643	Tumor
chrUn_gl000214	117482	2601	.	-1	targeted sequencing	Péneau et al. 2021	33563643	Tumor
chr10	39136802	1197	ACTR3BP5	-145431	targeted sequencing	Péneau et al. 2021	33563643	Tumor
chr5	426409	952	AHRR	0	targeted sequencing	Péneau et al. 2021	33563643	Tumor
chr6	16446222	2427	ATXN1	0	targeted sequencing	Péneau et al. 2021	33563643	Tumor
chr10	128281041	1815	C10orf90	-70812	targeted sequencing	Péneau et al. 2021	33563643	Tumor
chr2	118161356	398	DDX18	410747	targeted sequencing	Péneau et al. 2021	33563643	Tumor
chr12	50964543	923	DIP2B	0	targeted sequencing	Péneau et al. 2021	33563643	Tumor
chr2	203600236	841	FAM117B	0	targeted sequencing	Péneau et al. 2021	33563643	Tumor
chr10	51777516	3050	FAM21EP	3273	targeted sequencing	Péneau et al. 2021	33563643	Tumor
chr6	1483848	1987	FOXF2	-87897	targeted sequencing	Péneau et al. 2021	33563643	Tumor
chr8	43092989	2408	HGSNAT	-34815	targeted sequencing	Péneau et al. 2021	33563643	Tumor
chr8	43095189	2510	HGSNAT	-36786	targeted sequencing	Péneau et al. 2021	33563643	Tumor
chr8	136794621	602	KHDRBS3	-134613	targeted sequencing	Péneau et al. 2021	33563643	Tumor
chr9	12800890	1385	LURAP1L|LURAP1L-AS1	0	targeted sequencing	Péneau et al. 2021	33563643	Tumor
chr11	113026907	1130	NCAM1	0	targeted sequencing	Péneau et al. 2021	33563643	Tumor
chr2	134550653	2658	NCKAP5	-224441	targeted sequencing	Péneau et al. 2021	33563643	Tumor
chr5	22150	476	PLEKHG4B	117371	targeted sequencing	Péneau et al. 2021	33563643	Tumor
chr8	48831227	1872	PRKDC	0	targeted sequencing	Péneau et al. 2021	33563643	Tumor
chrX	23053518	1399	PTCHD1-AS	0	targeted sequencing	Péneau et al. 2021	33563643	Tumor
chr14	61058456	1747	SALRNA1	47478	targeted sequencing	Péneau et al. 2021	33563643	Tumor
chr5	1295720	2129	TERT	0	targeted sequencing	Péneau et al. 2021	33563643	Tumor
chr15	37819395	456	TMCO5A	407282	targeted sequencing	Péneau et al. 2021	33563643	Tumor
chr11	3416162	688	TSSC2	0	targeted sequencing	Péneau et al. 2021	33563643	Tumor
chr1	90561821	250	ZNF326	-60610	targeted sequencing	Péneau et al. 2021	33563643	Tumor
chr7	149330693	1760	ZNF767P	-8712	targeted sequencing	Péneau et al. 2021	33563643	Tumor
chr16	33496862	905	ENPP7P13	74981	targeted sequencing	Péneau et al. 2021	33563643	Tumor
chr1	195710098	147	KCNT2	484812	targeted sequencing	Péneau et al. 2021	33563643	Tumor
chr6	74824933	1806	LOC101928516	0	targeted sequencing	Péneau et al. 2021	33563643	Tumor
chr3	48354036	1964	SPINK8	0	targeted sequencing	Péneau et al. 2021	33563643	Tumor
chr7	15370796	2365	AGMO	0	targeted sequencing	Péneau et al. 2021	33563643	Tumor
chr7	15370803	1823	AGMO	0	targeted sequencing	Péneau et al. 2021	33563643	Tumor
chr16	46408263	1758	ANKRD26P1	94782	targeted sequencing	Péneau et al. 2021	33563643	Tumor
chr4	92406278	1818	CCSER1	0	targeted sequencing	Péneau et al. 2021	33563643	Tumor
chr4	92407316	1819	CCSER1	0	targeted sequencing	Péneau et al. 2021	33563643	Tumor
chr8	33949164	1818	DUSP26	-491540	targeted sequencing	Péneau et al. 2021	33563643	Tumor
chr1	120774894	1787	FAM72B	63956	targeted sequencing	Péneau et al. 2021	33563643	Tumor
chr3	51862237	1832	IQCF3	0	targeted sequencing	Péneau et al. 2021	33563643	Tumor
chr3	51862248	1808	IQCF3	0	targeted sequencing	Péneau et al. 2021	33563643	Tumor
chr1	18842243	1798	KLHDC7A	-29535	targeted sequencing	Péneau et al. 2021	33563643	Tumor
chr1	18842258	1863	KLHDC7A	-29535	targeted sequencing	Péneau et al. 2021	33563643	Tumor
chr13	65705989	1770	LINC01052	733684	targeted sequencing	Péneau et al. 2021	33563643	Tumor
chr13	65706003	1913	LINC01052	733684	targeted sequencing	Péneau et al. 2021	33563643	Tumor
chr6	67791739	1801	LOC102723883	973352	targeted sequencing	Péneau et al. 2021	33563643	Tumor
chr6	67791752	1889	LOC102723883	973352	targeted sequencing	Péneau et al. 2021	33563643	Tumor
chr8	123208187	1825	LOC105375734	218187	targeted sequencing	Péneau et al. 2021	33563643	Tumor
chr9	94293221	1083	MIR3910-1	105187	targeted sequencing	Péneau et al. 2021	33563643	Tumor
chr7	9173700	1796	NXPH1	-380802	targeted sequencing	Péneau et al. 2021	33563643	Tumor
chr7	9173708	1818	NXPH1	-380802	targeted sequencing	Péneau et al. 2021	33563643	Tumor
chr14	20225292	1001	OR4Q3	-8214	targeted sequencing	Péneau et al. 2021	33563643	Tumor
chr11	82538403	1828	PRCP	0	targeted sequencing	Péneau et al. 2021	33563643	Tumor
chr4	90732075	1820	SNCA	0	targeted sequencing	Péneau et al. 2021	33563643	Tumor
chr4	90732082	1825	SNCA	0	targeted sequencing	Péneau et al. 2021	33563643	Tumor
chr1	206500317	1824	SRGAP2	15638	targeted sequencing	Péneau et al. 2021	33563643	Tumor
chr4	166923250	1779	TLL1	0	targeted sequencing	Péneau et al. 2021	33563643	Tumor
chr4	166923264	1825	TLL1	0	targeted sequencing	Péneau et al. 2021	33563643	Tumor
chr3	23122679	1838	UBE2E2-AS1	113720	targeted sequencing	Péneau et al. 2021	33563643	Tumor
chr3	23122747	1824	UBE2E2-AS1	113720	targeted sequencing	Péneau et al. 2021	33563643	Tumor
chr8	125983166	1419	ZNF572	2373	targeted sequencing	Péneau et al. 2021	33563643	Tumor
chr3	52438139	2802	BAP1	0	targeted sequencing	Péneau et al. 2021	33563643	Tumor
chr19	36212906	3162	KMT2B	0	targeted sequencing	Péneau et al. 2021	33563643	Tumor
chr19	36212949	1791	KMT2B	0	targeted sequencing	Péneau et al. 2021	33563643	Tumor
chr2	52942153	1729	MIR4431	-11858	targeted sequencing	Péneau et al. 2021	33563643	Tumor
chr12	23674511	2121	SOX5	7220	targeted sequencing	Péneau et al. 2021	33563643	Tumor
chrY	9060562	1816	RBMY1A3P	93559	targeted sequencing	Péneau et al. 2021	33563643	Tumor
chrY	9060565	1823	RBMY1A3P	93559	targeted sequencing	Péneau et al. 2021	33563643	Tumor
chr2	92320137	184	ACTR3BP2	-189302	targeted sequencing	Péneau et al. 2021	33563643	Tumor
chr8	76906776	2046	LINC01111	411533	targeted sequencing	Péneau et al. 2021	33563643	Tumor
chr6	127482464	2168	RSPO3	0	targeted sequencing	Péneau et al. 2021	33563643	Tumor
chr7	82946903	875	SEMA3E	46069	targeted sequencing	Péneau et al. 2021	33563643	Tumor
chr20	3432483	291	ATRN	18853	targeted sequencing	Péneau et al. 2021	33563643	Tumor
chr8	26252008	1824	BNIP3L	0	targeted sequencing	Péneau et al. 2021	33563643	Tumor

Chromosome	Integration site in host genome	Integration site in virus genome	Gene (distance, bp)	Regions	Methods	Author	PMID	Sample
chr7	7241052	1731	C1GALT1	0	targeted sequencing	Péneau et al. 2021	33563643	Tumor
chr4	122742755	1786	CCNA2	0	targeted sequencing	Péneau et al. 2021	33563643	Tumor
chr4	122742792	1927	CCNA2	0	targeted sequencing	Péneau et al. 2021	33563643	Tumor
chr16	84954089	64	CRISPLD2	-10973	targeted sequencing	Péneau et al. 2021	33563643	Tumor
chr4	191033672	1822	DBET	-44653	targeted sequencing	Péneau et al. 2021	33563643	Tumor
chr8	26479251	432	DPYSL2	0	targeted sequencing	Péneau et al. 2021	33563643	Tumor
chr1	121484542	1541	EMBP1	-170855	targeted sequencing	Péneau et al. 2021	33563643	Tumor
chr20	42939726	976	FITM2	0	targeted sequencing	Péneau et al. 2021	33563643	Tumor
chr14	100107786	2881	HHIPL1	0	targeted sequencing	Péneau et al. 2021	33563643	Tumor
chr9	29951035	1819	LINC01242	437480	targeted sequencing	Péneau et al. 2021	33563643	Tumor
chr9	29951081	1046	LINC01242	437480	targeted sequencing	Péneau et al. 2021	33563643	Tumor
chr5	81899202	159	MIR3977	236772	targeted sequencing	Péneau et al. 2021	33563643	Tumor
chr5	81902434	322	MIR3977	233540	targeted sequencing	Péneau et al. 2021	33563643	Tumor
chr15	32064412	1819	OTUD7A	0	targeted sequencing	Péneau et al. 2021	33563643	Tumor
chr12	133196975	318	P2RX2	0	targeted sequencing	Péneau et al. 2021	33563643	Tumor
chr11	45194656	56	PRDM11	0	targeted sequencing	Péneau et al. 2021	33563643	Tumor
chr17	64776077	1820	PRKCA	0	targeted sequencing	Péneau et al. 2021	33563643	Tumor
chr17	64776099	1822	PRKCA	0	targeted sequencing	Péneau et al. 2021	33563643	Tumor
chr15	63139579	1372	TLN2	-2750	targeted sequencing	Péneau et al. 2021	33563643	Tumor
chr15	63180501	1769	TLN2	-43334	targeted sequencing	Péneau et al. 2021	33563643	Tumor
chr11	74645453	1127	XRRA1	0	targeted sequencing	Péneau et al. 2021	33563643	Tumor
chr16	84994669	216	ZDHHC7	13398	targeted sequencing	Péneau et al. 2021	33563643	Tumor
chr9	11017	1822	DDX11L5	488	targeted sequencing	Péneau et al. 2021	33563643	Tumor
chr5	1303638	1923	MIR4457	5787	targeted sequencing	Péneau et al. 2021	33563643	Tumor
chr5	1306288	1646	MIR4457	2649	targeted sequencing	Péneau et al. 2021	33563643	Tumor
chr1	186860595	840	PLA2G4A	0	targeted sequencing	Péneau et al. 2021	33563643	Tumor
chr3	35679510	1755	ARPP21	436	targeted sequencing	Péneau et al. 2021	33563643	Tumor
chr3	35679521	1825	ARPP21	436	targeted sequencing	Péneau et al. 2021	33563643	Tumor
chr7	44877949	NA	H2AFV	0	targeted sequencing	Péneau et al. 2021	33563643	Tumor
chr13	30503881	1234	LINC00572	-2457	targeted sequencing	Péneau et al. 2021	33563643	Tumor
chr13	33348880	493	PDS5B	0	targeted sequencing	Péneau et al. 2021	33563643	Tumor
chr21	47740234	2843	C21orf58	0	targeted sequencing	Péneau et al. 2021	33563643	Tumor
chr5	66641487	733	CD180	-148711	targeted sequencing	Péneau et al. 2021	33563643	Tumor
chr21	47655214	3141	MCM3AP|MCM3AP-AS1	0	targeted sequencing	Péneau et al. 2021	33563643	Tumor
chr8	54317879	429	OPRK1	-153016	targeted sequencing	Péneau et al. 2021	33563643	Tumor
chr8	54317925	2834	OPRK1	-153016	targeted sequencing	Péneau et al. 2021	33563643	Tumor
chr5	11647	512	PLEKHG4B	128599	targeted sequencing	Péneau et al. 2021	33563643	Tumor
chr5	1295318	1831	TERT	0	targeted sequencing	Péneau et al. 2021	33563643	Tumor
chr5	1295383	1826	TERT	0	targeted sequencing	Péneau et al. 2021	33563643	Tumor
chr4	47397	1821	ZNF595	5202	targeted sequencing	Péneau et al. 2021	33563643	Tumor
chr19	10594424	1599	KEAP1	2190	targeted sequencing	Péneau et al. 2021	33563643	Tumor
chr2	115856504	1691	DPP10	0	targeted sequencing	Péneau et al. 2021	33563643	Tumor
chr19	18499083	1645	GDF15	0	targeted sequencing	Péneau et al. 2021	33563643	Tumor
chr6_cox_hap2	24633	1417	GPX5	0	targeted sequencing	Péneau et al. 2021	33563643	Tumor
chr3	26103501	209	LINC00692	-188315	targeted sequencing	Péneau et al. 2021	33563643	Tumor
chr21	30518792	2247	MAP3K7CL	0	targeted sequencing	Péneau et al. 2021	33563643	Tumor
chr3	182970085	2600	MCF2L2	0	targeted sequencing	Péneau et al. 2021	33563643	Tumor
chr9	91339413	1765	MIR4289	20785	targeted sequencing	Péneau et al. 2021	33563643	Tumor
chr9	91339449	1795	MIR4289	20785	targeted sequencing	Péneau et al. 2021	33563643	Tumor
chr3	173257995	2890	NLGN1	0	targeted sequencing	Péneau et al. 2021	33563643	Tumor
chr16	51135403	1894	SALL1	34483	targeted sequencing	Péneau et al. 2021	33563643	Tumor
chr5	1295146	2937	TERT	0	targeted sequencing	Péneau et al. 2021	33563643	Tumor
chr5	1295153	1412	TERT	0	targeted sequencing	Péneau et al. 2021	33563643	Tumor
chr16	53331730	257	CHD9	0	targeted sequencing	Péneau et al. 2021	33563643	Tumor
chr11	84376571	3110	DLG2	0	targeted sequencing	Péneau et al. 2021	33563643	Tumor
chr5	1295374	1805	TERT	0	targeted sequencing	Péneau et al. 2021	33563643	Tumor
chr5	1295384	1092	TERT	0	targeted sequencing	Péneau et al. 2021	33563643	Tumor
chr12	102946341	3169	IGF1	-71114	targeted sequencing	Péneau et al. 2021	33563643	Tumor
chr12	102946398	1782	IGF1	-71114	targeted sequencing	Péneau et al. 2021	33563643	Tumor
chr13	51561768	367	GUCY1B2	6343	targeted sequencing	Péneau et al. 2021	33563643	Tumor
chr13	51561801	1618	GUCY1B2	6343	targeted sequencing	Péneau et al. 2021	33563643	Tumor
chr10	42596923	1107	LOC441666	230098	targeted sequencing	Péneau et al. 2021	33563643	Tumor
chr2	243153317	1799	LOC728323	-50754	targeted sequencing	Péneau et al. 2021	33563643	Tumor
chr4	74285653	395	ALB	0	targeted sequencing	Péneau et al. 2021	33563643	Tumor
chr4	74285665	1048	ALB	0	targeted sequencing	Péneau et al. 2021	33563643	Tumor
chr1	121484598	85	EMBP1	-170372	targeted sequencing	Péneau et al. 2021	33563643	Tumor
chr1	121484848	1356	EMBP1	-170372	targeted sequencing	Péneau et al. 2021	33563643	Tumor
chr14	100349209	457	EML1	0	targeted sequencing	Péneau et al. 2021	33563643	Tumor
chr16	85600880	540	GSE1	44149	targeted sequencing	Péneau et al. 2021	33563643	Tumor
chr8	43094787	1803	HGSNAT	-36778	targeted sequencing	Péneau et al. 2021	33563643	Tumor
chr8	43097093	2018	HGSNAT	-39012	targeted sequencing	Péneau et al. 2021	33563643	Tumor
chr4	58938047	23	LOC105377671	452453	targeted sequencing	Péneau et al. 2021	33563643	Tumor
chr7	72399490	2427	POM121	0	targeted sequencing	Péneau et al. 2021	33563643	Tumor
chr7	72399503	2232	POM121	0	targeted sequencing	Péneau et al. 2021	33563643	Tumor
chr17	1663736	NA	SERPINF1	1483	targeted sequencing	Péneau et al. 2021	33563643	Tumor
chr22	41229508	1845	ST13	0	targeted sequencing	Péneau et al. 2021	33563643	Tumor
chr22	41229528	1717	ST13	0	targeted sequencing	Péneau et al. 2021	33563643	Tumor
chr17	74567335	233	ST6GALNAC2	0	targeted sequencing	Péneau et al. 2021	33563643	Tumor
chr2	137809804	1676	THSD7B	0	targeted sequencing	Péneau et al. 2021	33563643	Tumor
chr3	64373736	2194	ADAMTS9	127487	targeted sequencing	Péneau et al. 2021	33563643	Tumor
chr19	17115923	2465	CPAMD8	0	targeted sequencing	Péneau et al. 2021	33563643	Tumor
chr5	11027294	1375	CTNND2	0	targeted sequencing	Péneau et al. 2021	33563643	Tumor
chr19	39324570	810	ECH1	-1755	targeted sequencing	Péneau et al. 2021	33563643	Tumor
chr6	55176088	3053	GFRAL	16179	targeted sequencing	Péneau et al. 2021	33563643	Tumor
chr9	98804706	2612	LINC00092	-20669	targeted sequencing	Péneau et al. 2021	33563643	Tumor
chrX	50868347	1207	LINC01284	0	targeted sequencing	Péneau et al. 2021	33563643	Tumor
chr6	6594711	1681	LY86|LY86-AS1	0	targeted sequencing	Péneau et al. 2021	33563643	Tumor
chr18	10980	2975	MIR4318	-133091	targeted sequencing	Péneau et al. 2021	33563643	Tumor
chr18	35370849	2946	MIR4318	-133091	targeted sequencing	Péneau et al. 2021	33563643	Tumor
chr5	1304787	2194	MIR4457	4637	targeted sequencing	Péneau et al. 2021	33563643	Tumor
chr9	14194667	3053	NFIB	0	targeted sequencing	Péneau et al. 2021	33563643	Tumor
chr1	249240047	672	PGBD2	-26320	targeted sequencing	Péneau et al. 2021	33563643	Tumor
chr20	58113528	1103	PHACTR3	39036	targeted sequencing	Péneau et al. 2021	33563643	Tumor
chr10	3284909	283	PITRM1	-69007	targeted sequencing	Péneau et al. 2021	33563643	Tumor

Chromosome	Integration site in host genome	Integration site in virus genome	Gene (distance, bp)	Regions	Methods	Author	PMID	Sample
chrUn_gl000220	156839	1361	RNA5-8S5	-268	targeted sequencing	Péneau et al. 2021	33563643	Tumor
chr6	118365921	342	SLC35F1	0	targeted sequencing	Péneau et al. 2021	33563643	Tumor
chrX	79331425	1566	TBX22	-44022	targeted sequencing	Péneau et al. 2021	33563643	Tumor
chr3	102480067	2271	ZPLD1	-281382	targeted sequencing	Péneau et al. 2021	33563643	Tumor
chrUn_gl000214	64225	1552	.	-1	targeted sequencing	Péneau et al. 2021	33563643	Tumor
chr4	100237029	1905	ADH1B	0	targeted sequencing	Péneau et al. 2021	33563643	Tumor
chr17	41210489	2927	BRCA1	0	targeted sequencing	Péneau et al. 2021	33563643	Tumor
chr17	41210505	1613	BRCA1	0	targeted sequencing	Péneau et al. 2021	33563643	Tumor
chr3	131377795	1870	CPNE4	0	targeted sequencing	Péneau et al. 2021	33563643	Tumor
chr3	131378422	2016	CPNE4	0	targeted sequencing	Péneau et al. 2021	33563643	Tumor
chr5	12167208	1826	CTNND2	-262651	targeted sequencing	Péneau et al. 2021	33563643	Tumor
chr5	12167209	1118	CTNND2	-262651	targeted sequencing	Péneau et al. 2021	33563643	Tumor
chr16	67573935	29	FAM65A	0	targeted sequencing	Péneau et al. 2021	33563643	Tumor
chr2	216255429	1789	FN1	0	targeted sequencing	Péneau et al. 2021	33563643	Tumor
chr2	216255443	2690	FN1	0	targeted sequencing	Péneau et al. 2021	33563643	Tumor
chr17	53379200	1822	HLF	0	targeted sequencing	Péneau et al. 2021	33563643	Tumor
chr17	53379216	585	HLF	0	targeted sequencing	Péneau et al. 2021	33563643	Tumor
chr20	16563412	1800	KIF16B	-9300	targeted sequencing	Péneau et al. 2021	33563643	Tumor
chr20	16563419	1821	KIF16B	-9300	targeted sequencing	Péneau et al. 2021	33563643	Tumor
chr2	143448656	502	KYNU	186066	targeted sequencing	Péneau et al. 2021	33563643	Tumor
chr2	749508	1808	LINC01115	30329	targeted sequencing	Péneau et al. 2021	33563643	Tumor
chr7	66992919	1019	LINC01372	-187907	targeted sequencing	Péneau et al. 2021	33563643	Tumor
chr5	36118796	1579	LMBRD2	0	targeted sequencing	Péneau et al. 2021	33563643	Tumor
chr5	36130522	1832	LMBRD2	0	targeted sequencing	Péneau et al. 2021	33563643	Tumor
chr16	32414675	1712	LOC390705	-113373	targeted sequencing	Péneau et al. 2021	33563643	Tumor
chr10	42387198	2587	LOC441666	439952	targeted sequencing	Péneau et al. 2021	33563643	Tumor
chr2	60455578	2141	MIR4432HG	130476	targeted sequencing	Péneau et al. 2021	33563643	Tumor
chr2	60455580	1651	MIR4432HG	130476	targeted sequencing	Péneau et al. 2021	33563643	Tumor
chr5	68845819	82	OCLN	0	targeted sequencing	Péneau et al. 2021	33563643	Tumor
chr1	249240278	852	PGBD2	-26832	targeted sequencing	Péneau et al. 2021	33563643	Tumor
chr3	51652329	2134	RAD54L2	0	targeted sequencing	Péneau et al. 2021	33563643	Tumor
chr3	51652342	1820	RAD54L2	0	targeted sequencing	Péneau et al. 2021	33563643	Tumor
chr8	108727424	1571	RSPO2	183911	targeted sequencing	Péneau et al. 2021	33563643	Tumor
chr8	108727429	2580	RSPO2	183911	targeted sequencing	Péneau et al. 2021	33563643	Tumor
chr7	40277590	1667	SUGCT	0	targeted sequencing	Péneau et al. 2021	33563643	Tumor
chr7	40277600	2407	SUGCT	0	targeted sequencing	Péneau et al. 2021	33563643	Tumor
chr5	1300842	249	TERT	-5171	targeted sequencing	Péneau et al. 2021	33563643	Tumor
chr5	1300901	1799	TERT	-5171	targeted sequencing	Péneau et al. 2021	33563643	Tumor
chr4	10045	1879	ZNF595	43023	targeted sequencing	Péneau et al. 2021	33563643	Tumor
chr17	14138411	2670	CDRT15	762	targeted sequencing	Péneau et al. 2021	33563643	Tumor
chr17	13929710	2098	CDRT15P1	-655	targeted sequencing	Péneau et al. 2021	33563643	Tumor
chr17	14113953	2977	COX10	-1693	targeted sequencing	Péneau et al. 2021	33563643	Tumor
chr4	191044096	1216	DBET	-55076	targeted sequencing	Péneau et al. 2021	33563643	Tumor
chr1	202207430	387	LGR6	0	targeted sequencing	Péneau et al. 2021	33563643	Tumor
chr12	95336	742	LOC100288778	-4042	targeted sequencing	Péneau et al. 2021	33563643	Tumor
chr2	186356463	2232	LOC101927196	227623	targeted sequencing	Péneau et al. 2021	33563643	Tumor
chr2	186356465	2009	LOC101927196	227623	targeted sequencing	Péneau et al. 2021	33563643	Tumor
chr3	16024750	1671	MIR563	-108908	targeted sequencing	Péneau et al. 2021	33563643	Tumor
chr3	174663455	1190	NAALADL2	0	targeted sequencing	Péneau et al. 2021	33563643	Tumor
chr8	32007414	2505	NRG1	0	targeted sequencing	Péneau et al. 2021	33563643	Tumor
chr15	68345712	1017	PIAS1	805	targeted sequencing	Péneau et al. 2021	33563643	Tumor
chr5	11760	738	PLEKHG4B	128577	targeted sequencing	Péneau et al. 2021	33563643	Tumor
chr6	64193613	128	PTP4A1	88302	targeted sequencing	Péneau et al. 2021	33563643	Tumor
chr17	78688138	3020	RPTOR	0	targeted sequencing	Péneau et al. 2021	33563643	Tumor
chr5	1295322	1826	TERT	0	targeted sequencing	Péneau et al. 2021	33563643	Tumor
chr21	32748716	2200	TIAM1	0	targeted sequencing	Péneau et al. 2021	33563643	Tumor
chr4	66555735	300	EPHA5-AS1	0	targeted sequencing	Péneau et al. 2021	33563643	Tumor
chr10	42596982	1370	LOC441666	230086	targeted sequencing	Péneau et al. 2021	33563643	Tumor
chr22	28286724	911	PITPNB	0	targeted sequencing	Péneau et al. 2021	33563643	Tumor
chr5	1299504	1781	TERT	-3727	targeted sequencing	Péneau et al. 2021	33563643	Tumor
chr12	105586807	1408	APPL2	0	targeted sequencing	Péneau et al. 2021	33563643	Tumor
chr8	22036372	1395	BMP1	0	targeted sequencing	Péneau et al. 2021	33563643	Tumor
chr13	30934520	1180	LINC00426	0	targeted sequencing	Péneau et al. 2021	33563643	Tumor
chr3	166372426	434	LOC105374194	243865	targeted sequencing	Péneau et al. 2021	33563643	Tumor
chr6	78300817	2612	MEI4	59136	targeted sequencing	Péneau et al. 2021	33563643	Tumor
chr5	131466329	2830	P4HA2-AS1	54083	targeted sequencing	Péneau et al. 2021	33563643	Tumor
chr5	131466485	1099	P4HA2-AS1	54083	targeted sequencing	Péneau et al. 2021	33563643	Tumor
chr1	249240136	1316	PGBD2	-26570	targeted sequencing	Péneau et al. 2021	33563643	Tumor
chr6	150203525	1369	RAET1E-AS1	0	targeted sequencing	Péneau et al. 2021	33563643	Tumor
chr15	74166702	2626	TBC1D21	0	targeted sequencing	Péneau et al. 2021	33563643	Tumor
chr5	1296010	1825	TERT	0	targeted sequencing	Péneau et al. 2021	33563643	Tumor
chr12	30301238	2539	TMTC1	-363430	targeted sequencing	Péneau et al. 2021	33563643	Tumor
chr17	18629039	501	TRIM16L	0	targeted sequencing	Péneau et al. 2021	33563643	Tumor
chr13	32952612	1828	BRCA2	0	targeted sequencing	Péneau et al. 2021	33563643	Tumor
chr13	32952621	1924	BRCA2	0	targeted sequencing	Péneau et al. 2021	33563643	Tumor
chr7	90623793	2192	CDK14	0	targeted sequencing	Péneau et al. 2021	33563643	Tumor
chr18	71534411	2351	FBXO15	206176	targeted sequencing	Péneau et al. 2021	33563643	Tumor
chr18	71608999	3065	FBXO15	131095	targeted sequencing	Péneau et al. 2021	33563643	Tumor
chr1	6478464	683	HES2	0	targeted sequencing	Péneau et al. 2021	33563643	Tumor
chr13	98695613	247	IPO5	-18949	targeted sequencing	Péneau et al. 2021	33563643	Tumor
chr1	196357720	316	KCNT2	0	targeted sequencing	Péneau et al. 2021	33563643	Tumor
chr19	27738544	2237	LINC00662	542729	targeted sequencing	Péneau et al. 2021	33563643	Tumor
chr19	27738670	799	LINC00662	542729	targeted sequencing	Péneau et al. 2021	33563643	Tumor
chr5	1304094	1753	MIR4457	4820	targeted sequencing	Péneau et al. 2021	33563643	Tumor
chr5	1304100	992	MIR4457	4820	targeted sequencing	Péneau et al. 2021	33563643	Tumor
chr21	32996021	2732	SOD1	35534	targeted sequencing	Péneau et al. 2021	33563643	Tumor
chr5	90105600	1388	ADGRV1	0	targeted sequencing	Péneau et al. 2021	33563643	Tumor
chr7	91930296	81	ANKIB1	0	targeted sequencing	Péneau et al. 2021	33563643	Tumor
chr11	116688339	1819	APOA4	3079	targeted sequencing	Péneau et al. 2021	33563643	Tumor
chr8	82651381	1090	CHMP4C	0	targeted sequencing	Péneau et al. 2021	33563643	Tumor
chr6	117872964	1910	DCBLD1	0	targeted sequencing	Péneau et al. 2021	33563643	Tumor
chr7	154264272	2595	DPP6	0	targeted sequencing	Péneau et al. 2021	33563643	Tumor
chr8	109170961	2912	EIF3E	43011	targeted sequencing	Péneau et al. 2021	33563643	Tumor
chr2	221579437	1812	EPHA4	702768	targeted sequencing	Péneau et al. 2021	33563643	Tumor

Supplementary Table S8 Continued

Chromosome	Integration site in host genome	Integration site in virus genome	Gene (distance, bp)	Regions	Methods	Author	PMID	Sample
chr2	221579443	2056	EPHA4	702768	targeted sequencing	Péneau et al. 2021	33563643	Tumor
chr16	35247682	1366	FLJ26245	-256100	targeted sequencing	Péneau et al. 2021	33563643	Tumor
chr16	35250459	1844	FLJ26245	-258769	targeted sequencing	Péneau et al. 2021	33563643	Tumor
chr5	161012533	3182	GABRB2	-37403	targeted sequencing	Péneau et al. 2021	33563643	Tumor
chr12	13302489	3177	GSG1	-45761	targeted sequencing	Péneau et al. 2021	33563643	Tumor
chr12	66575513	3062	IRAK3	7361	targeted sequencing	Péneau et al. 2021	33563643	Tumor
chr17	68133440	1201	KCNJ16	-1694	targeted sequencing	Péneau et al. 2021	33563643	Tumor
chr1	37780061	2254	LINC01137	140419	targeted sequencing	Péneau et al. 2021	33563643	Tumor
chr16	60062777	1180	LOC101927580	0	targeted sequencing	Péneau et al. 2021	33563643	Tumor
chr10	42597024	60	LOC441666	230129	targeted sequencing	Péneau et al. 2021	33563643	Tumor
chr10	42602991	NA	LOC441666	224323	targeted sequencing	Péneau et al. 2021	33563643	Tumor
chr12	12314025	1528	LRP6	0	targeted sequencing	Péneau et al. 2021	33563643	Tumor
chr1	46417287	1180	MAST2	0	targeted sequencing	Péneau et al. 2021	33563643	Tumor
chr10	54985577	292	MBL2	-454011	targeted sequencing	Péneau et al. 2021	33563643	Tumor
chr9	68280228	1056	MIR4477A|MIR4477B	134975	targeted sequencing	Péneau et al. 2021	33563643	Tumor
chr9	68280228	2254	MIR4477A|MIR4477B	134975	targeted sequencing	Péneau et al. 2021	33563643	Tumor
chr1	149035095	1638	NBPF25P	0	targeted sequencing	Péneau et al. 2021	33563643	Tumor
chr3	189655025	1180	P3H2	19492	targeted sequencing	Péneau et al. 2021	33563643	Tumor
chr6	163069530	2603	PARK2	0	targeted sequencing	Péneau et al. 2021	33563643	Tumor
chr10	23001495	1094	PIP4K2A	0	targeted sequencing	Péneau et al. 2021	33563643	Tumor
chr1	183657184	1944	RGL1	0	targeted sequencing	Péneau et al. 2021	33563643	Tumor
chr5	155770192	1171	SGCD	0	targeted sequencing	Péneau et al. 2021	33563643	Tumor
chr7	99894888	1180	SPDYE3	10320	targeted sequencing	Péneau et al. 2021	33563643	Tumor
chr12	119480398	2395	SRRM4	0	targeted sequencing	Péneau et al. 2021	33563643	Tumor
chr21	10439362	2777	TPTE	466703	targeted sequencing	Péneau et al. 2021	33563643	Tumor
chr6	144970226	1826	UTRN	0	targeted sequencing	Péneau et al. 2021	33563643	Tumor
chr6	144970228	2333	UTRN	0	targeted sequencing	Péneau et al. 2021	33563643	Tumor
chr4	108951496	2249	HADH	0	targeted sequencing	Péneau et al. 2021	33563643	Tumor
chr4	108951530	1623	HADH	0	targeted sequencing	Péneau et al. 2021	33563643	Tumor
chr4	157299180	597	PDGFC	383583	targeted sequencing	Péneau et al. 2021	33563643	Tumor
chr5	1295727	1828	TERT	0	targeted sequencing	Péneau et al. 2021	33563643	Tumor
chr1	49348553	1856	AGBL4	0	targeted sequencing	Péneau et al. 2021	33563643	Tumor
chr1	217367251	2434	ESRRG	-56154	targeted sequencing	Péneau et al. 2021	33563643	Tumor
chr6	57875873	1826	GUSBP4	358957	targeted sequencing	Péneau et al. 2021	33563643	Tumor
chr8	133351385	247	KCNQ3	0	targeted sequencing	Péneau et al. 2021	33563643	Tumor
chr17	20278054	1789	CCDC144CP	0	targeted sequencing	Péneau et al. 2021	33563643	Tumor
chr17	20279865	1601	CCDC144CP	0	targeted sequencing	Péneau et al. 2021	33563643	Tumor
chr12	95184	1783	LOC100288778	-3921	targeted sequencing	Péneau et al. 2021	33563643	Tumor
chr12	73951104	1659	LOC101928137	-349007	targeted sequencing	Péneau et al. 2021	33563643	Tumor
chr17	21210104	93	MAP2K3	0	targeted sequencing	Péneau et al. 2021	33563643	Tumor
chr18	24473302	1123	AQP4-AS1	0	targeted sequencing	Péneau et al. 2021	33563643	Tumor
chr18	24473320	2791	AQP4-AS1	0	targeted sequencing	Péneau et al. 2021	33563643	Tumor
chr11	71945868	1238	INPPL1	0	targeted sequencing	Péneau et al. 2021	33563643	Tumor
chr22	18878537	1914	LOC100996432	0	targeted sequencing	Péneau et al. 2021	33563643	Tumor
chr22	18878555	1124	LOC100996432	0	targeted sequencing	Péneau et al. 2021	33563643	Tumor
chr18	12918538	1825	SEH1L	29445	targeted sequencing	Péneau et al. 2021	33563643	Tumor
chr9_gl000199_random	34522	2400	.	-1	targeted sequencing	Péneau et al. 2021	33563643	Tumor
chr4	191043793	1869	DBET	-54689	targeted sequencing	Péneau et al. 2021	33563643	Tumor
chr5	1295743	1810	TERT	0	targeted sequencing	Péneau et al. 2021	33563643	Tumor
chr9	100940295	1782	CORO2A	intronic	conventional PCR and Sang	Ruan et al. 2019	30535432	Tumor
chr10	34836578	2360	PARD3	intronic	conventional PCR and Sang	Ruan et al. 2019	30535432	Tumor
chr13	29623309	1752	MTUS2	intronic	HBV-CapSeq	Mayuko et al. 2018	29861854	Tumor
chr5	1275390	1836	TERT	intronic	HBV-CapSeq	Mayuko et al. 2018	29861854	Tumor
chr5	1275381	1783	TERT	intronic	HBV-CapSeq	Mayuko et al. 2018	29861854	Tumor
chr1	10191186	2341	UBE4B	intronic	HBV-CapSeq	Mayuko et al. 2018	29861854	Tumor
chr1	174968592	1820	CACYBP	intronic	HBV-CapSeq	Mayuko et al. 2018	29861854	Tumor
chr1	174968601	3215	CACYBP	intronic	HBV-CapSeq	Mayuko et al. 2018	29861854	Tumor
chr1	218607404	1896	TGFB2	intronic	HBV-CapSeq	Mayuko et al. 2018	29861854	Tumor
chr1	218607379	1788	TGFB2	intronic	HBV-CapSeq	Mayuko et al. 2018	29861854	Tumor
chr11	7850010	1822	OR5P3	promoter	HBV-CapSeq	Mayuko et al. 2018	29861854	Tumor
chr12	6769179	2566	ING4	intronic	HBV-CapSeq	Mayuko et al. 2018	29861854	Tumor
chr12	6769179	1817	ING4	intronic	HBV-CapSeq	Mayuko et al. 2018	29861854	Tumor
chr13	30073292	1828	MTUS2	intronic	HBV-CapSeq	Mayuko et al. 2018	29861854	Tumor
chr17	80062089	1807	CCDC57	intronic	HBV-CapSeq	Mayuko et al. 2018	29861854	Tumor
chr2	224774901	1702	WDFY1	intronic	HBV-CapSeq	Mayuko et al. 2018	29861854	Tumor
chr2	224774921	1816	WDFY1	intronic	HBV-CapSeq	Mayuko et al. 2018	29861854	Tumor
chr5	139347517	2476	NRG2	intronic	HBV-CapSeq	Mayuko et al. 2018	29861854	Tumor
chr7	91671908	2746	AKAP9	intronic	HBV-CapSeq	Mayuko et al. 2018	29861854	Tumor
chr7	155443986	2063	RBM33	intronic	HBV-CapSeq	Mayuko et al. 2018	29861854	Tumor
chr7	155444002	1695	RBM33	intronic	HBV-CapSeq	Mayuko et al. 2018	29861854	Tumor
chr8	49642747	2741	EFCAB1	intronic	HBV-CapSeq	Mayuko et al. 2018	29861854	Tumor
chr1	26784061	1847	DHDDS	intronic	HBV-CapSeq	Mayuko et al. 2018	29861854	Tumor
chr19	36212781	987	KMT2B	intronic	HBV-CapSeq	Mayuko et al. 2018	29861854	Tumor
chr19	36212800	1827	KMT2B	intronic	HBV-CapSeq	Mayuko et al. 2018	29861854	Tumor
chr20	35170199	2376	DLGAP4-AS1 MYL9	intronic	HBV-CapSeq	Mayuko et al. 2018	29861854	Tumor
chr3	24378343	1257	THRB	intronic	HBV-CapSeq	Mayuko et al. 2018	29861854	Tumor
chr3	24499331	2140	THRB	intronic	HBV-CapSeq	Mayuko et al. 2018	29861854	Tumor
chr3	24499357	2156	THRB	intronic	HBV-CapSeq	Mayuko et al. 2018	29861854	Tumor
chr3	84718506	2807	LINC00971	exonic	HBV-CapSeq	Mayuko et al. 2018	29861854	Tumor
chr3	85542081	1572	CADM2	intronic	HBV-CapSeq	Mayuko et al. 2018	29861854	Tumor
chr7	23553423	1	TRA2A	intronic	HBV-CapSeq	Mayuko et al. 2018	29861854	Tumor
chr9	115157931	1397	HSDL2	intronic	HBV-CapSeq	Mayuko et al. 2018	29861854	Tumor
AP011098	1829	91770215	---	intronic	HBV-CapSeq	Mayuko et al. 2018	29861854	Tumor
chr2	202708330	1610	CDK15	intronic	HBV-CapSeq	Mayuko et al. 2018	29861854	Tumor
chr2	202749962	1603	CDK15	intronic	HBV-CapSeq	Mayuko et al. 2018	29861854	Tumor
chr4	74108392	1820	ANKRD17	intronic	HBV-CapSeq	Mayuko et al. 2018	29861854	Tumor
chr17	58147100	2569	HEATR6	exonic	HBV-CapSeq	Mayuko et al. 2018	29861854	Tumor
chr10	7312353	1992	SFMBT2	intronic	HBV-CapSeq	Mayuko et al. 2018	29861854	Tumor
chr11	92193725	1826	FAT3	intronic	HBV-CapSeq	Mayuko et al. 2018	29861854	Tumor
chr17	61415494	1774	TANC2	intronic	HBV-CapSeq	Mayuko et al. 2018	29861854	Tumor
chr2	216299860	1262	FN1	intronic	HBV-CapSeq	Mayuko et al. 2018	29861854	Tumor
chr2	239979162	1569	HDAC4	intronic	HBV-CapSeq	Mayuko et al. 2018	29861854	Tumor
chr20	53170161	1817	DOK5	intronic	HBV-CapSeq	Mayuko et al. 2018	29861854	Tumor
chr8	63280969	1786	NKAIN3	intronic	HBV-CapSeq	Mayuko et al. 2018	29861854	Tumor

Chromosome	Integration site in host genome	Integration site in virus genome	Gene (distance, bp)	Regions	Methods	Author	PMID	Sample
chr1	31503630	1834	PUM1	intronic	HBV-CapSeq	Mayuko et al. 2018	29861854	Tumor
chr1	245547461	1774	KIF26B	intronic	HBV-CapSeq	Mayuko et al. 2018	29861854	Tumor
chr1	245659641	2356	KIF26B	intronic	HBV-CapSeq	Mayuko et al. 2018	29861854	Tumor
chr19	36212667	1818	KMT2B	exonic	HBV-CapSeq	Mayuko et al. 2018	29861854	Tumor
chr19	36212665	1754	KMT2B	exonic	HBV-CapSeq	Mayuko et al. 2018	29861854	Tumor
chr13	96965850	1794	HS6ST3	intronic	HBV-CapSeq	Mayuko et al. 2018	29861854	Tumor
chr5	1295451	937	---	promoter	HBV-CapSeq	Mayuko et al. 2018	29861854	Tumor
chr5	1295436	1771	---	promoter	HBV-CapSeq	Mayuko et al. 2018	29861854	Tumor
chr17	15243373	1818	TEKT3	exonic	HBV-CapSeq	Mayuko et al. 2018	29861854	Tumor
chr20	53170161	1817	DOK5	intronic	HBV-CapSeq	Mayuko et al. 2018	29861854	Tumor
chr20	53170216	2376	DOK5	intronic	HBV-CapSeq	Mayuko et al. 2018	29861854	Tumor
chr5	64503436	2758	ADAMTS6	intronic	HBV-CapSeq	Mayuko et al. 2018	29861854	Tumor
chr2	105457560	1674	LINC01158	intronic	HBV-CapSeq	Mayuko et al. 2018	29861854	Tumor
chr2	105457534	50	LINC01158	intronic	HBV-CapSeq	Mayuko et al. 2018	29861854	Tumor
chr4	122742409	1639	CCNA2	intronic	HBV-CapSeq	Mayuko et al. 2018	29861854	Tumor
chr8	53617388	1741	RB1CC1	intronic	HBV-CapSeq	Mayuko et al. 2018	29861854	Tumor
chr8	53617387	2609	RB1CC1	intronic	HBV-CapSeq	Mayuko et al. 2018	29861854	Tumor
chr13	96965849	1793	HS6ST3	intronic	HBV-CapSeq	Mayuko et al. 2018	29861854	Tumor
chr15	71645727	1820	THSD4	intronic	HBV-CapSeq	Mayuko et al. 2018	29861854	Tumor
chr19	4558121	2471	SEMA6B	exonic	HBV-CapSeq	Mayuko et al. 2018	29861854	Tumor
chr2	105457531	50	LINC01158	intronic	HBV-CapSeq	Mayuko et al. 2018	29861854	Tumor
chr2	105457560	1674	LINC01158	intronic	HBV-CapSeq	Mayuko et al. 2018	29861854	Tumor
chr4	122742412	1808	CCNA2	intronic	HBV-CapSeq	Mayuko et al. 2018	29861854	Tumor
chr4	122742414	1639	CCNA2	intronic	HBV-CapSeq	Mayuko et al. 2018	29861854	Tumor
chr1	35838761	1743	ZMYM4	intronic	HBV-CapSeq	Mayuko et al. 2018	29861854	Tumor
chr2	42456652	952	EML4	intronic	HBV-CapSeq	Mayuko et al. 2018	29861854	Tumor
chr2	42456686	1821	EML4	intronic	HBV-CapSeq	Mayuko et al. 2018	29861854	Tumor
chr5	673521	2558	TPPP	intronic	HBV-CapSeq	Mayuko et al. 2018	29861854	Tumor
chr5	676981	1751	TPPP	intronic	HBV-CapSeq	Mayuko et al. 2018	29861854	Tumor
chr5	69001501	2775	GUSBP3	intronic	HBV-CapSeq	Mayuko et al. 2018	29861854	Tumor
chr5	69876812	2775	SMA5 GUSBP3	intronic	HBV-CapSeq	Mayuko et al. 2018	29861854	Tumor
chr6	26919478	2775	GUSBP2	intronic	HBV-CapSeq	Mayuko et al. 2018	29861854	Tumor
chr19	36212648	1800	KMT2B	exonic	HBV-CapSeq	Mayuko et al. 2018	29861854	Tumor
chr19	36212637	1848	KMT2B	exonic	HBV-CapSeq	Mayuko et al. 2018	29861854	Tumor
chr4	3421873	2382	RGS12	intronic	HBV-CapSeq	Mayuko et al. 2018	29861854	Tumor
chr1	57257058	1495	C1orf168	intronic	HBV-CapSeq	Mayuko et al. 2018	29861854	Tumor
chr16	72095190	1783	---	promoter	HBV-CapSeq	Mayuko et al. 2018	29861854	Tumor
chr16	72095216	2028	---	promoter	HBV-CapSeq	Mayuko et al. 2018	29861854	Tumor
chr10	20525538	163	PLXDC2	intronic	HBV-CapSeq	Mayuko et al. 2018	29861854	Tumor
chr19	36214046	1343	KMT2B	exonic	HBV-CapSeq	Mayuko et al. 2018	29861854	Tumor
chr19	36214043	1964	KMT2B	exonic	HBV-CapSeq	Mayuko et al. 2018	29861854	Tumor
chr8	68545532	1805	CPA6	intronic	HBV-CapSeq	Mayuko et al. 2018	29861854	Tumor
chr19	36213941	1572	KMT2B	exonic	HBV-CapSeq	Mayuko et al. 2018	29861854	Tumor
chr22	24151748	2929	SMARCB1	intronic	HBV-CapSeq	Mayuko et al. 2018	29861854	Tumor
chr5	1295333	1828	---	promoter	HBV-CapSeq	Mayuko et al. 2018	29861854	Tumor
chr5	1295376	139	---	promoter	HBV-CapSeq	Mayuko et al. 2018	29861854	Tumor
chr5	66120930	1822	MAST4	intronic	HBV-CapSeq	Mayuko et al. 2018	29861854	Tumor
chr10	93786860	2619	BTAF1	intronic	HBV-CapSeq	Mayuko et al. 2018	29861854	Tumor
chr5	1298126	2609	---	promoter	HBV-CapSeq	Mayuko et al. 2018	29861854	Tumor
chr5	1298223	1786	---	promoter	HBV-CapSeq	Mayuko et al. 2018	29861854	Tumor
chrAP011098	41	150837860	---	intronic	HBV-CapSeq	Mayuko et al. 2018	29861854	Tumor
chr5	1295650	533	---	promoter	HBV-CapSeq	Mayuko et al. 2018	29861854	Tumor
chr5	1295748	3215	---	promoter	HBV-CapSeq	Mayuko et al. 2018	29861854	Tumor
chr5	1296159	638	---	promoter	HBV-CapSeq	Mayuko et al. 2018	29861854	Tumor
chr7	729320	1837	PRKAR1B	intronic	HBV-CapSeq	Mayuko et al. 2018	29861854	Tumor
chr7	729358	1754	PRKAR1B	intronic	HBV-CapSeq	Mayuko et al. 2018	29861854	Tumor
chr19	1840632	1815	REXO1	intronic	HBV-CapSeq	Mayuko et al. 2018	29861854	Tumor
chr19	36213863	1897	KMT2B	intronic	HBV-CapSeq	Mayuko et al. 2018	29861854	Tumor
chr19	36213879	1515	KMT2B	intronic	HBV-CapSeq	Mayuko et al. 2018	29861854	Tumor
AP011098	816	130843837	---	intronic	HBV-CapSeq	Mayuko et al. 2018	29861854	Tumor
chr2	186622918	1999	FSIP2	intronic	HBV-CapSeq	Mayuko et al. 2018	29861854	Tumor
AP011098	1792	14927990	---	intronic	HBV-CapSeq	Mayuko et al. 2018	29861854	Tumor
AP011098	1989	14927992	---	intronic	HBV-CapSeq	Mayuko et al. 2018	29861854	Tumor
chr11	64570855	1913	---	promoter	HBV-CapSeq	Mayuko et al. 2018	29861854	Tumor
chr5	1295600	1819	---	promoter	HBV-CapSeq	Mayuko et al. 2018	29861854	Tumor
chr5	1296717	1457	---	promoter	HBV-CapSeq	Mayuko et al. 2018	29861854	Tumor
chr7	80512239	3017	SEMA3C	intronic	HBV-CapSeq	Mayuko et al. 2018	29861854	Tumor
AP011098	41	150837860	---	intronic	HBV-CapSeq	Mayuko et al. 2018	29861854	Tumor
chr5	1294880	1836	TERT	intronic	HBV-CapSeq	Mayuko et al. 2018	29861854	Tumor
chr5	1294860	1850	TERT	intronic	HBV-CapSeq	Mayuko et al. 2018	29861854	Tumor
chr1	49996775	1125	AGBL4	intronic	HBV-CapSeq	Mayuko et al. 2018	29861854	Tumor
chr19	1840628	1817	REXO1	intronic	HBV-CapSeq	Mayuko et al. 2018	29861854	Tumor
chr19	1840659	1456	REXO1	intronic	HBV-CapSeq	Mayuko et al. 2018	29861854	Tumor
chr2	191997039	2644	STAT4	intronic	HBV-CapSeq	Mayuko et al. 2018	29861854	Tumor
chr4	2869472	1094	ADD1	intronic	HBV-CapSeq	Mayuko et al. 2018	29861854	Tumor
chr17	14933983	1154	---	promoter	HBV-CapSeq	Mayuko et al. 2018	29861854	Tumor
chr7	128665044	2744	TNPO3	intronic	HBV-CapSeq	Mayuko et al. 2018	29861854	Tumor
chr1	31503626	1831	PUM1 intron	intronic	WGS	Akihiro et al. 2016	27230686	Tumor
chr1	35838747	1743	ZMYM4 intron	intronic	WGS	Akihiro et al. 2016	27230686	Tumor
chr1	53589332	2794	SLC1A7 intron	intronic	WGS	Akihiro et al. 2016	27230686	Tumor
chr1	53589378	759	SLC1A7 intron	intronic	WGS	Akihiro et al. 2016	27230686	Tumor
chr1	57033100	246	PPAP2B intron, LOC100288348 intron	intronic	WGS	Akihiro et al. 2016	27230686	Tumor
chr1	57257046	1451	C1orf168 intron	intronic	WGS	Akihiro et al. 2016	27230686	Tumor
chr1	80892944	899	-		WGS	Akihiro et al. 2016	27230686	Tumor
chr1	80892946	1824	-		WGS	Akihiro et al. 2016	27230686	Tumor
chr1	80934653	2052	-		WGS	Akihiro et al. 2016	27230686	Tumor
chr1	80934672	1760	-		WGS	Akihiro et al. 2016	27230686	Tumor
chr1	106996329	1849	-		WGS	Akihiro et al. 2016	27230686	Tumor
chr1	106996401	1458	-		WGS	Akihiro et al. 2016	27230686	Tumor
chr1	161091640	1645	DEDD UTR3, NIT1 FLANK3, PFDN2 promoter	promoter	WGS	Akihiro et al. 2016	27230686	Tumor
chr1	220821611	680	MARK1 intron	intronic	WGS	Akihiro et al. 2016	27230686	Tumor
chr1	220821613	2445	MARK1 intron	intronic	WGS	Akihiro et al. 2016	27230686	Tumor
chr1	234101885	1757	SLC35F3 intron	intronic	WGS	Akihiro et al. 2016	27230686	Tumor
chr1	234103097	2890	SLC35F3 intron	intronic	WGS	Akihiro et al. 2016	27230686	Tumor

Chromosome	Integration site in host genome	Integration site in virus genome	Gene (distance, bp)	Regions	Methods	Author	PMID	Sample
chr1	245547453	1774	KIF26B intron	intronic	WGS	Akihiro et al. 2016	27230686	Tumor
chr1	245659642	2355	KIF26B intron	intronic	WGS	Akihiro et al. 2016	27230686	Tumor
chr2	14604475	981	-		WGS	Akihiro et al. 2016	27230686	Tumor
chr2	42456657	944	EML4 intron	intronic	WGS	Akihiro et al. 2016	27230686	Tumor
chr2	42456683	1822	EML4 intron	intronic	WGS	Akihiro et al. 2016	27230686	Tumor
chr2	105457536	68	-		WGS	Akihiro et al. 2016	27230686	Tumor
chr2	105457561	1680	-		WGS	Akihiro et al. 2016	27230686	Tumor
chr2	186622914	1998	-		WGS	Akihiro et al. 2016	27230686	Tumor
chr2	186641089	984	-		WGS	Akihiro et al. 2016	27230686	Tumor
chr2	202708327	1627	PFTK2 intron	intronic	WGS	Akihiro et al. 2016	27230686	Tumor
chr2	202750060	1603	PFTK2 intron	intronic	WGS	Akihiro et al. 2016	27230686	Tumor
chr2	217055351	2529	XRCC5 intron	intronic	WGS	Akihiro et al. 2016	27230686	Tumor
chr2	217055411	743	XRCC5 intron	intronic	WGS	Akihiro et al. 2016	27230686	Tumor
chr3	21222313	504	-		WGS	Akihiro et al. 2016	27230686	Tumor
chr3	24001271	2133	NR1D2 CDS		WGS	Akihiro et al. 2016	27230686	Tumor
chr3	24001495	2479	NR1D2 intron	intronic	WGS	Akihiro et al. 2016	27230686	Tumor
chr3	24499339	2087	THRB intron	intronic	WGS	Akihiro et al. 2016	27230686	Tumor
chr3	162048378	2645	LOC100132484 pGENE		WGS	Akihiro et al. 2016	27230686	Tumor
chr3	162048405	2132	LOC100132484 pGENE		WGS	Akihiro et al. 2016	27230686	Tumor
chr3	169023909	193	MDS1 intron	intronic	WGS	Akihiro et al. 2016	27230686	Tumor
chr4	3421893	2416	RGS12 intron	intronic	WGS	Akihiro et al. 2016	27230686	Tumor
chr4	19502309	140	-		WGS	Akihiro et al. 2016	27230686	Tumor
chr4	33650718	2763	-		WGS	Akihiro et al. 2016	27230686	Tumor
chr4	42950229	1793	GRXCR1 intron	intronic	WGS	Akihiro et al. 2016	27230686	Tumor
chr4	54834797	483	-		WGS	Akihiro et al. 2016	27230686	Tumor
chr4	55244162	643	-		WGS	Akihiro et al. 2016	27230686	Tumor
chr4	63879681	1741	-		WGS	Akihiro et al. 2016	27230686	Tumor
chr4	74108318	17	ANKRD17 intron	intronic	WGS	Akihiro et al. 2016	27230686	Tumor
chr4	74108456	1749	ANKRD17 intron	intronic	WGS	Akihiro et al. 2016	27230686	Tumor
chr4	74901473	2935	CXCL3 FLANK3		WGS	Akihiro et al. 2016	27230686	Tumor
chr4	79048008	1809	FRAS1 intron	intronic	WGS	Akihiro et al. 2016	27230686	Tumor
chr4	79048013	1819	FRAS1 intron	intronic	WGS	Akihiro et al. 2016	27230686	Tumor
chr4	122742221	1817	CCNA2 CDS		WGS	Akihiro et al. 2016	27230686	Tumor
chr4	122742581	1639	CCNA2 intron	intronic	WGS	Akihiro et al. 2016	27230686	Tumor
chr4	151954237	1829	-		WGS	Akihiro et al. 2016	27230686	Tumor
chr4	180753995	1040	-		WGS	Akihiro et al. 2016	27230686	Tumor
chr4	181085995	2712	-		WGS	Akihiro et al. 2016	27230686	Tumor
chr4	190730779	822	-		WGS	Akihiro et al. 2016	27230686	Tumor
chr5	673522	2558	LOC100132605 intron, TPPP intron	intronic	WGS	Akihiro et al. 2016	27230686	Tumor
chr5	677082	1707	TPPP intron	intronic	WGS	Akihiro et al. 2016	27230686	Tumor
chr5	1275339	1780	TERT intron	intronic	WGS	Akihiro et al. 2016	27230686	Tumor
chr5	1275389	1832	TERT intron	intronic	WGS	Akihiro et al. 2016	27230686	Tumor
chr5	1293405	1827	TERT intron	intronic	WGS	Akihiro et al. 2016	27230686	Tumor
chr5	1293413	2236	TERT intron	intronic	WGS	Akihiro et al. 2016	27230686	Tumor
chr5	1294680	1854	TERT CDS		WGS	Akihiro et al. 2016	27230686	Tumor
chr5	1294807	2021	TERT intron	intronic	WGS	Akihiro et al. 2016	27230686	Tumor
chr5	1294907	1866	TERT CDS		WGS	Akihiro et al. 2016	27230686	Tumor
chr5	1295079	1801	TERT CDS		WGS	Akihiro et al. 2016	27230686	Tumor
chr5	1295158	1851	TERT UTR5	5UTR	WGS	Akihiro et al. 2016	27230686	Tumor
chr5	1295183	1474	TERT FLANK5, TERT promoter	promoter	WGS	Akihiro et al. 2016	27230686	Tumor
chr5	1295221	19	TERT FLANK5, TERT promoter	promoter	WGS	Akihiro et al. 2016	27230686	Tumor
chr5	1295231	153	TERT FLANK5, TERT promoter	promoter	WGS	Akihiro et al. 2016	27230686	Tumor
chr5	1295249	1722	TERT FLANK5, TERT promoter	promoter	WGS	Akihiro et al. 2016	27230686	Tumor
chr5	1295286	2758	TERT FLANK5, TERT promoter	promoter	WGS	Akihiro et al. 2016	27230686	Tumor
chr5	1295438	1753	TERT FLANK5, TERT promoter	promoter	WGS	Akihiro et al. 2016	27230686	Tumor
chr5	1295443	1025	TERT FLANK5, TERT promoter	promoter	WGS	Akihiro et al. 2016	27230686	Tumor
chr5	1295735	3097	TERT FLANK5, TERT promoter	promoter	WGS	Akihiro et al. 2016	27230686	Tumor
chr5	1296151	634	TERT FLANK5, TERT promoter	promoter	WGS	Akihiro et al. 2016	27230686	Tumor
chr5	1296404	1783	TERT promoter	promoter	WGS	Akihiro et al. 2016	27230686	Tumor
chr5	1296487	2494	TERT promoter	promoter	WGS	Akihiro et al. 2016	27230686	Tumor
chr5	1296714	1459	TERT promoter	promoter	WGS	Akihiro et al. 2016	27230686	Tumor
chr5	1298118	2605	TERT promoter	promoter	WGS	Akihiro et al. 2016	27230686	Tumor
chr5	1298227	1787	TERT promoter	promoter	WGS	Akihiro et al. 2016	27230686	Tumor
chr5	1298631	1753	TERT promoter	promoter	WGS	Akihiro et al. 2016	27230686	Tumor
chr5	1310429	1416	-		WGS	Akihiro et al. 2016	27230686	Tumor
chr5	1553015	3215	-		WGS	Akihiro et al. 2016	27230686	Tumor
chr5	16264250	418	-		WGS	Akihiro et al. 2016	27230686	Tumor
chr5	19301244	36	-		WGS	Akihiro et al. 2016	27230686	Tumor
chr5	36749272	1182	-		WGS	Akihiro et al. 2016	27230686	Tumor
chr5	41551275	2368	-		WGS	Akihiro et al. 2016	27230686	Tumor
chr5	62473247	1134	-		WGS	Akihiro et al. 2016	27230686	Tumor
chr5	62576191	3060	-		WGS	Akihiro et al. 2016	27230686	Tumor
chr5	62576199	3	-		WGS	Akihiro et al. 2016	27230686	Tumor
chr5	64503439	2754	ADAMTS6	intronic	WGS	Akihiro et al. 2016	27230686	Tumor
chr5	88432444	1735	-		WGS	Akihiro et al. 2016	27230686	Tumor
chr5	88754904	687	-		WGS	Akihiro et al. 2016	27230686	Tumor
chr5	129990968	1820	-		WGS	Akihiro et al. 2016	27230686	Tumor
chr5	129991025	2115	-		WGS	Akihiro et al. 2016	27230686	Tumor
chr5	152215061	1752	-		WGS	Akihiro et al. 2016	27230686	Tumor
chr5	173125611	1892	-		WGS	Akihiro et al. 2016	27230686	Tumor
chr5	173174734	2503	-		WGS	Akihiro et al. 2016	27230686	Tumor
chr5	173175423	364	-		WGS	Akihiro et al. 2016	27230686	Tumor
chr6	43307979	1375	ZNF318 CDS		WGS	Akihiro et al. 2016	27230686	Tumor
chr6	43307993	1178	ZNF318 CDS		WGS	Akihiro et al. 2016	27230686	Tumor
chr6	44270937	1744	TCTE1	promoter	WGS	Akihiro et al. 2016	27230686	Tumor
chr6	44270941	1834	TCTE1	promoter	WGS	Akihiro et al. 2016	27230686	Tumor
chr6	44270937	1744	AARS2	intronic	WGS	Akihiro et al. 2016	27230686	Tumor
chr6	44270941	1834	AARS2	intronic	WGS	Akihiro et al. 2016	27230686	Tumor
chr6	68664016	1489	-		WGS	Akihiro et al. 2016	27230686	Tumor
chr6	152891524	2659	SYNE1	intronic	WGS	Akihiro et al. 2016	27230686	Tumor
chr6	162294328	1855	PARK2	intronic	WGS	Akihiro et al. 2016	27230686	Tumor
chr6	162298236	1466	PARK2	intronic	WGS	Akihiro et al. 2016	27230686	Tumor
chr6	164970001	3212	-		WGS	Akihiro et al. 2016	27230686	Tumor
chr7	729323	1837	PRKAR1B	intronic	WGS	Akihiro et al. 2016	27230686	Tumor

Chromosome	Integration site in host genome	Integration site in virus genome	Gene (distance, bp)	Regions	Methods	Author	PMID	Sample
chr7	52802406	1507	-		WGS	Akihiro et al. 2016	27230686	Tumor
chr7	71198387	1873	-		WGS	Akihiro et al. 2016	27230686	Tumor
chr7	71198442	1786	-		WGS	Akihiro et al. 2016	27230686	Tumor
chr7	80512272	3005	SEMA3C intron	intronic	WGS	Akihiro et al. 2016	27230686	Tumor
chr7	91671863	1821	AKAP9 intron	intronic	WGS	Akihiro et al. 2016	27230686	Tumor
chr7	91671910	2741	AKAP9 intron	intronic	WGS	Akihiro et al. 2016	27230686	Tumor
chr7	103802548	2066	ORC5L intron	intronic	WGS	Akihiro et al. 2016	27230686	Tumor
chr7	121520007	1761	PTPRZ1 intron	intronic	WGS	Akihiro et al. 2016	27230686	Tumor
chr7	128664866	277	TNPO3 intron, LOC100132905 pIVS	intronic	WGS	Akihiro et al. 2016	27230686	Tumor
chr7	145783066	2478	-		WGS	Akihiro et al. 2016	27230686	Tumor
chr7	145783086	1823	-		WGS	Akihiro et al. 2016	27230686	Tumor
chr7	154265433	3084	-		WGS	Akihiro et al. 2016	27230686	Tumor
chr7	154265737	1102	-		WGS	Akihiro et al. 2016	27230686	Tumor
chr7	155443943	987	RBM33 intron	intronic	WGS	Akihiro et al. 2016	27230686	Tumor
chr7	155443949	2021	RBM33 intron	intronic	WGS	Akihiro et al. 2016	27230686	Tumor
chr7	155444002	1634	RBM33 intron	intronic	WGS	Akihiro et al. 2016	27230686	Tumor
chr8	37337657	1025	-		WGS	Akihiro et al. 2016	27230686	Tumor
chr8	39200454	1771	ADAM5P pIVS		WGS	Akihiro et al. 2016	27230686	Tumor
chr8	39369790	2484	ADAM3A pIVS		WGS	Akihiro et al. 2016	27230686	Tumor
chr8	41308707	2507	-		WGS	Akihiro et al. 2016	27230686	Tumor
chr8	49642751	2745	EFCAB1 intron	intronic	WGS	Akihiro et al. 2016	27230686	Tumor
chr8	52216280	816	-		WGS	Akihiro et al. 2016	27230686	Tumor
chr8	53617384	1726	RB1CC1 intron	intronic	WGS	Akihiro et al. 2016	27230686	Tumor
chr8	53617385	2625	RB1CC1 intron	intronic	WGS	Akihiro et al. 2016	27230686	Tumor
chr8	57507944	2009	-		WGS	Akihiro et al. 2016	27230686	Tumor
chr8	63280963	2472	NKAIN3 intron	intronic	WGS	Akihiro et al. 2016	27230686	Tumor
chr8	63280990	1788	NKAIN3 intron	intronic	WGS	Akihiro et al. 2016	27230686	Tumor
chr8	68545529	1676	CPA6 intron	intronic	WGS	Akihiro et al. 2016	27230686	Tumor
chr8	89649912	1829	-		WGS	Akihiro et al. 2016	27230686	Tumor
chr8	132473199	3212	-		WGS	Akihiro et al. 2016	27230686	Tumor
chr9	24184261	1961	-		WGS	Akihiro et al. 2016	27230686	Tumor
chr9	24396871	1802	-		WGS	Akihiro et al. 2016	27230686	Tumor
chr9	105333812	1820	-		WGS	Akihiro et al. 2016	27230686	Tumor
chr10	1883909	2654	-		WGS	Akihiro et al. 2016	27230686	Tumor
chr10	1883933	1626	-		WGS	Akihiro et al. 2016	27230686	Tumor
chr10	7312349	2016	SFMBT2 intron	intronic	WGS	Akihiro et al. 2016	27230686	Tumor
chr10	20525580	163	PLXDC2 intron	intronic	WGS	Akihiro et al. 2016	27230686	Tumor
chr10	112718174	1823	SHOC2 intron	intronic	WGS	Akihiro et al. 2016	27230686	Tumor
chr11	65023717	1839	POLA2 promoter	promoter	WGS	Akihiro et al. 2016	27230686	Tumor
chr11	83120874	1232	-		WGS	Akihiro et al. 2016	27230686	Tumor
chr11	87553687	1862	LOC100289080 pGENE		WGS	Akihiro et al. 2016	27230686	Tumor
chr11	122939930	1819	HSPA8 promoter	promoter	WGS	Akihiro et al. 2016	27230686	Tumor
chr12	23811443	2806	SOX5 intron	intronic	WGS	Akihiro et al. 2016	27230686	Tumor
chr12	23811547	672	SOX5 intron	intronic	WGS	Akihiro et al. 2016	27230686	Tumor
chr12	24671602	801	SOX5 intron	intronic	WGS	Akihiro et al. 2016	27230686	Tumor
chr12	30301884	2419	-		WGS	Akihiro et al. 2016	27230686	Tumor
chr12	30643805	1801	-		WGS	Akihiro et al. 2016	27230686	Tumor
chr12	30643899	1837	-		WGS	Akihiro et al. 2016	27230686	Tumor
chr12	58550781	1755	-		WGS	Akihiro et al. 2016	27230686	Tumor
chr12	58550804	1535	-		WGS	Akihiro et al. 2016	27230686	Tumor
chr12	69723801	1761	-		WGS	Akihiro et al. 2016	27230686	Tumor
chr12	93019067	368	-		WGS	Akihiro et al. 2016	27230686	Tumor
chr12	95169110	637	-		WGS	Akihiro et al. 2016	27230686	Tumor
chr12	121439103	1198	HNF1A UTR5	5UTR	WGS	Akihiro et al. 2016	27230686	Tumor
chr13	38591682	1488	-		WGS	Akihiro et al. 2016	27230686	Tumor
chr13	81850218	1821	-		WGS	Akihiro et al. 2016	27230686	Tumor
chr13	82130234	1801	-		WGS	Akihiro et al. 2016	27230686	Tumor
chr13	96965850	1794	HS6ST3 intron	intronic	WGS	Akihiro et al. 2016	27230686	Tumor
chr13	97585857	1837	-		WGS	Akihiro et al. 2016	27230686	Tumor
chr13	97586002	1636	-		WGS	Akihiro et al. 2016	27230686	Tumor
chr13	103554268	731	-		WGS	Akihiro et al. 2016	27230686	Tumor
chr13	104161305	2391	-		WGS	Akihiro et al. 2016	27230686	Tumor
chr13	104214794	2996	-		WGS	Akihiro et al. 2016	27230686	Tumor
chr13	104280513	3015	-		WGS	Akihiro et al. 2016	27230686	Tumor
chr13	104316887	2307	-		WGS	Akihiro et al. 2016	27230686	Tumor
chr13	104473938	816	-		WGS	Akihiro et al. 2016	27230686	Tumor
chr13	104474088	2725	-		WGS	Akihiro et al. 2016	27230686	Tumor
chr14	96181078	1805	TCL1A FLANK5, TCL1A promoter	promoter	WGS	Akihiro et al. 2016	27230686	Tumor
chr14	96181117	2856	TCL1A FLANK5, TCL1A promoter	promoter	WGS	Akihiro et al. 2016	27230686	Tumor
chr15	37994956	1943	-		WGS	Akihiro et al. 2016	27230686	Tumor
chr15	37994979	1944	-		WGS	Akihiro et al. 2016	27230686	Tumor
chr15	48094848	2729	-		WGS	Akihiro et al. 2016	27230686	Tumor
chr15	48094912	1818	-		WGS	Akihiro et al. 2016	27230686	Tumor
chr15	71645717	1820	THSD4	intronic	WGS	Akihiro et al. 2016	27230686	Tumor
chr16	22353630	649	-		WGS	Akihiro et al. 2016	27230686	Tumor
chr16	22353678	1818	-		WGS	Akihiro et al. 2016	27230686	Tumor
chr16	62325166	1598	-		WGS	Akihiro et al. 2016	27230686	Tumor
chr16	72095254	2026	HPR promoter, HP FLANK3		WGS	Akihiro et al. 2016	27230686	Tumor
chr16	81543548	428	CMIP	intronic	WGS	Akihiro et al. 2016	27230686	Tumor
chr17	14634042	2052	-		WGS	Akihiro et al. 2016	27230686	Tumor
chr17	14933986	1139	CDRT7	promoter	WGS	Akihiro et al. 2016	27230686	Tumor
chr17	15243475	1751	TEKT3	intronic	WGS	Akihiro et al. 2016	27230686	Tumor
chr17	15412636	1681	FAM18B2	intronic	WGS	Akihiro et al. 2016	27230686	Tumor
chr17	37006422	2413	C17orf98	promoter	WGS	Akihiro et al. 2016	27230686	Tumor
chr17	58147104	2585	HEATR6 CDS		WGS	Akihiro et al. 2016	27230686	Tumor
chr17	58157689	809	HEATR6	promoter	WGS	Akihiro et al. 2016	27230686	Tumor
chr18	40716596	1057	-		WGS	Akihiro et al. 2016	27230686	Tumor
chr18	70874012	12	-		WGS	Akihiro et al. 2016	27230686	Tumor
chr18	70874013	1774	-		WGS	Akihiro et al. 2016	27230686	Tumor
chr19	1840615	1754	REXO1	intronic	WGS	Akihiro et al. 2016	27230686	Tumor
chr19	1840674	1419	REXO1	intronic	WGS	Akihiro et al. 2016	27230686	Tumor
chr19	4558119	2446	SEMA6B CDS		WGS	Akihiro et al. 2016	27230686	Tumor
chr19	8315414	1613	LASS4	intronic	WGS	Akihiro et al. 2016	27230686	Tumor
chr19	8315644	1333	LASS4	intronic	WGS	Akihiro et al. 2016	27230686	Tumor

Chromosome	Integration site in host genome	Integration site in virus genome	Gene (distance, bp)	Regions	Methods	Author	PMID	Sample
chr19	29087653	1830	-		WGS	Akihiro et al. 2016	27230686	Tumor
chr19	30304909	2040	CCNE1	intronic	WGS	Akihiro et al. 2016	27230686	Tumor
chr19	30305243	1808	CCNE1	intronic	WGS	Akihiro et al. 2016	27230686	Tumor
chr19	33771843	2495	-		WGS	Akihiro et al. 2016	27230686	Tumor
chr19	36212624	1949	KMT2B CDS		WGS	Akihiro et al. 2016	27230686	Tumor
chr19	36212784	1811	KMT2B	intronic	WGS	Akihiro et al. 2016	27230686	Tumor
chr19	36212806	1800	KMT2B	intronic	WGS	Akihiro et al. 2016	27230686	Tumor
chr19	36213149	1823	KMT2B	intronic	WGS	Akihiro et al. 2016	27230686	Tumor
chr19	36213865	212	KMT2B	intronic	WGS	Akihiro et al. 2016	27230686	Tumor
chr19	36213947	1577	KMT2B CDS		WGS	Akihiro et al. 2016	27230686	Tumor
chr19	36214023	1945	KMT2B CDS		WGS	Akihiro et al. 2016	27230686	Tumor
chr20	30502086	2734	TTLL9	intronic	WGS	Akihiro et al. 2016	27230686	Tumor
chr20	53170149	1817	DOK5	intronic	WGS	Akihiro et al. 2016	27230686	Tumor
chr20	53170212	2376	DOK5	intronic	WGS	Akihiro et al. 2016	27230686	Tumor
chr21	47547287	2922	LOC100288508	intronic	WGS	Akihiro et al. 2016	27230686	Tumor
chr21	47547287	2922	COL6A2	intronic	WGS	Akihiro et al. 2016	27230686	Tumor
chr21	47547409	1978	LOC100288508	intronic	WGS	Akihiro et al. 2016	27230686	Tumor
chr21	47547409	1978	COL6A2	intronic	WGS	Akihiro et al. 2016	27230686	Tumor
chrX	25471062	1810	-		WGS	Akihiro et al. 2016	27230686	Tumor
chrX	150837858	49	-		WGS	Akihiro et al. 2016	27230686	Tumor
chrY	14927954	1736	-		WGS	Akihiro et al. 2016	27230686	Tumor
chrY	14928007	1979	-		WGS	Akihiro et al. 2016	27230686	Tumor
chr3	169023909	193	MDS1	intronic	WGS	Akihiro et al. 2016	25636086	Tumor
chr16	81543548	428	CMIP	intronic	WGS	Akihiro et al. 2016	25636086	Tumor
X	25471062	1810	CMIP	intronic	WGS	Akihiro et al. 2016	25636086	Tumor
chr19	36214027	1742	KMT2B	intronic	RNA-seq	Khoury et al. 2013	23740984	Tumor
chr10	129391432	1245	FOXI2	5UTR	RNA-seq	Khoury et al. 2013	23740984	Tumor
chr10	129391427	1419	FOXI2	5UTR	RNA-seq	Khoury et al. 2013	23740984	Tumor
chr16	31402241	282	ITGAD	5UTR	RNA-seq	Khoury et al. 2013	23740984	Tumor
chr16	31413434	1991	ITGAD	intronic	RNA-seq	Khoury et al. 2013	23740984	Tumor
chr16	31413431	1883	ITGAD	intronic	RNA-seq	Khoury et al. 2013	23740984	Tumor
chr11	12711205	1748	TEAD1	intronic	RNA-seq	Khoury et al. 2013	23740984	Tumor
chr4	63651241	1359	TECRL	3UTR	RNA-seq	Khoury et al. 2013	23740984	Tumor
chr4	63647552	372	TECRL	3UTR	RNA-seq	Khoury et al. 2013	23740984	Tumor
chr4	63651170	1373	TECRL	3UTR	RNA-seq	Khoury et al. 2013	23740984	Tumor
chr13	96216582	211	CLDN10	intronic	RNA-seq	Khoury et al. 2013	23740984	Tumor
chr13	96229482	2943	CLDN10	intronic	RNA-seq	Khoury et al. 2013	23740984	Tumor
chr13	96216538	42	CLDN10	intronic	RNA-seq	Khoury et al. 2013	23740984	Tumor
chr13	96229477	2290	CLDN10	intronic	RNA-seq	Khoury et al. 2013	23740984	Tumor
chr7	35111018	1540	DPY19L2P1	3UTR	RNA-seq	Khoury et al. 2013	23740984	Tumor
chr1	210977324	341	KCNH1	intronic	RNA-seq	Khoury et al. 2013	23740984	Tumor
chr1	211058920	1800	KCNH1	intronic	RNA-seq	Khoury et al. 2013	23740984	Tumor
chr19	36255648	337	C19orf55	intronic	RNA-seq	Khoury et al. 2013	23740984	Tumor
chr19	36213891	420	KMT2B	intronic	RNA-seq	Khoury et al. 2013	23740984	Tumor
chr19	36212301	2097	KMT2B	Exon 3	RNA-seq	Khoury et al. 2013	23740984	Tumor
chr2	121585830	2403	GLI2	intronic	RNA-seq	Khoury et al. 2013	23740984	Tumor
chr2	121585623	412	GLI2	intronic	RNA-seq	Khoury et al. 2013	23740984	Tumor
chr2	121585885	23	GLI2	intronic	RNA-seq	Khoury et al. 2013	23740984	Tumor
chr2	121585602	1966	GLI2	intronic	RNA-seq	Khoury et al. 2013	23740984	Tumor
chr5	1291950	25	TERT	intronic	RNA-seq	Khoury et al. 2013	23740984	Tumor
chr2	57459913	1675	VRK2	5UTR	RNA-seq	Khoury et al. 2013	23740984	Tumor
chr2	216279398	386	FN1	intronic	RNA-seq	Khoury et al. 2013	23740984	Tumor
chr4	173136585	2006	GALNTL6	intronic	RNA-seq	Khoury et al. 2013	23740984	Tumor
chr10	1863160	1659	LOC282980	3UTR	RNA-seq	Khoury et al. 2013	23740984	Tumor
chr12	124912988	1684	NCOR2	intronic	RNA-seq	Khoury et al. 2013	23740984	Tumor
chr5	1294653	406	TERT	intronic	RNA-seq	Khoury et al. 2013	23740984	Tumor
chr5	1295462	1369	MIR4457	3UTR	RNA-seq	Khoury et al. 2013	23740984	Tumor
chr5	1295516	1373	MIR4457	3UTR	RNA-seq	Khoury et al. 2013	23740984	Tumor
chr2	192364684	433	OBFC2A	5UTR	RNA-seq	Khoury et al. 2013	23740984	Tumor
chr2	192367406	1730	OBFC2A	5UTR	RNA-seq	Khoury et al. 2013	23740984	Tumor
chr5	1294676	1569	TERT	intronic	RNA-seq	Khoury et al. 2013	23740984	Tumor
chr7	10697705	1562	NDUFA4	3UTR	RNA-seq	Khoury et al. 2013	23740984	Tumor
chr7	22735275	1782	IL-6	5UTR	RNA-seq	Khoury et al. 2013	23740984	Tumor
chr5	1295037	1711	TERT	intronic	RNA-seq	Khoury et al. 2013	23740984	Tumor
chr2	118543485	1330	DDX18	5UTR	RNA-seq	Khoury et al. 2013	23740984	Tumor
chr2	118543232	1584	DDX18	5UTR	RNA-seq	Khoury et al. 2013	23740984	Tumor
chr9	137028935	2735	RNU6ATAC	5UTR	RNA-seq	Khoury et al. 2013	23740984	Tumor
chr9	137029164	1635	RNU6ATAC	3UTR	RNA-seq	Khoury et al. 2013	23740984	Tumor
chr5	1272107	2391	TERT	intronic	RNA-seq	Khoury et al. 2013	23740984	Tumor
chr5	1272167	406	TERT	intronic	RNA-seq	Khoury et al. 2013	23740984	Tumor
chr5	1272106	1577	TERT	intronic	RNA-seq	Khoury et al. 2013	23740984	Tumor
chr5	1272250	2035	TERT	intronic	RNA-seq	Khoury et al. 2013	23740984	Tumor
chr2	202709075	1503	CDK15	intronic	RNA-seq	Khoury et al. 2013	23740984	Tumor
chr1	178593988	1660	MIR4424	5UTR	RNA-seq	Khoury et al. 2013	23740984	Tumor
chr2	11183657		SPG4	intronic	RNA-seq	Hidenori et al. 2008	18023912	Tumor
chr13	19337933		DIAPH3	intronic	RNA-seq	Hidenori et al. 2008	18023912	Tumor
chr8	4155242		FDFT1	intronic	RNA-seq	Hidenori et al. 2008	18023912	Tumor
chr19	23275592		SBBI54	intronic	RNA-seq	Hidenori et al. 2008	18023912	Tumor
chr1	9911738		MACF1	intronic	RNA-seq	Hidenori et al. 2008	18023912	Tumor
chr4	122743189	1807	CCNA2	intronic	RNA-seq	Bayard et al. 2018	30531861	Tumor
chr4	122742792	1928	CCNA2	intronic	RNA-seq	Bayard et al. 2018	30531861	Tumor
chr19	30291247	1674	CCNE1	intronic	RNA-seq	Bayard et al. 2018	30531861	Tumor
chr19	30300450	885	CCNE1	intronic	RNA-seq	Bayard et al. 2018	30531861	Tumor
chr19	30302064	1830	CCNE1	intronic	RNA-seq	Bayard et al. 2018	30531861	Tumor
chr13	96229483-96229	1056;1032;2449	DZIP1|22873;CLDN10|9071	intronic	RNA-seq	Cancer Genome Atlas Research Network. 2017	28622513	Tumor
chr1	210977510-2110	458;1826	KCNH1|3756	intronic	RNA-seq	Cancer Genome Atlas Research Network. 2017	28622513	Tumor
chr15	32109798	960	OTUD7A|161725	intronic	RNA-seq	Cancer Genome Atlas Research Network. 2017	28622513	Tumor
chr1	180715843	926	XPR1|9213	intronic	RNA-seq	Cancer Genome Atlas Research Network. 2017	28622513	Tumor
chr3	115386949	626	GAP43|2596	intronic	RNA-seq	Cancer Genome Atlas Research Network. 2017	28622513	Tumor
chr9	6935868	208	KDM4C|23081	intronic	RNA-seq	Cancer Genome Atlas Research Network. 2017	28622513	Tumor
chrX	55766879-55766	205;380	RRAGB|10325	intronic	RNA-seq	Cancer Genome Atlas Research Network. 2017	28622513	Tumor
chr20	18419643	292	C20orf12|55184	intronic	RNA-seq	Cancer Genome Atlas Research Network. 2017	28622513	Tumor
chr19	36214026	1824	KMT2B|9757	intronic	RNA-seq	Cancer Genome Atlas Research Network. 2017	28622513	Tumor
chr17	29549341	901	NF1|4763	intronic	RNA-seq	Cancer Genome Atlas Research Network. 2017	28622513	Tumor

Chromosome	Integration site in host genome	Integration site in virus genome	Gene (distance, bp)	Regions	Methods	Author	PMID	Sample
chr5	64041555	3068	SFRS12IP1|285672	intronic	RNA-seq	Cancer Genome Atlas Research Network. 2017	28622513	Tumor
chr12	75454741	1731	KCNC2|3747	intronic	RNA-seq	Cancer Genome Atlas Research Network. 2017	28622513	Tumor
chr16	31413434-31414	2067;2087	ITGAD|3681	intronic	RNA-seq	Cancer Genome Atlas Research Network. 2017	28622513	Tumor
chr11	12711305-12711	1824;2984	TEAD1|7003	intronic	RNA-seq	Cancer Genome Atlas Research Network. 2017	28622513	Tumor
chr11	116701286	1344	APOC3|345	intronic	RNA-seq	Cancer Genome Atlas Research Network. 2017	28622513	Tumor
chr19	18125295	832	ARRDC2|27106	intronic	RNA-seq	Cancer Genome Atlas Research Network. 2017	28622513	Tumor
chr17	63547188	1750	AXIN2|8313	intronic	RNA-seq	Cancer Genome Atlas Research Network. 2017	28622513	Tumor
chr17	11550725	1752	DNAH9|1770	intronic	RNA-seq	Cancer Genome Atlas Research Network. 2017	28622513	Tumor
chr2	109127516	969	GCC2|9648	intronic	RNA-seq	Cancer Genome Atlas Research Network. 2017	28622513	Tumor
chr15	71313528	846	LRRC49|54839	intronic	RNA-seq	Cancer Genome Atlas Research Network. 2017	28622513	Tumor
chr18	31504573	1717	NOL4|8715	intronic	RNA-seq	Cancer Genome Atlas Research Network. 2017	28622513	Tumor
chr11	120165340	781	POU2F3|25833	intronic	RNA-seq	Cancer Genome Atlas Research Network. 2017	28622513	Tumor
chr6	57348303	832	PRIM2|5558	intronic	RNA-seq	Cancer Genome Atlas Research Network. 2017	28622513	Tumor
chr20	17625224	1716	RRBP1|6238	intronic	RNA-seq	Cancer Genome Atlas Research Network. 2017	28622513	Tumor
chr5	1268747-127239	459;1717;1628;	TERT|7015	intronic	RNA-seq	Cancer Genome Atlas Research Network. 2017	28622513	Tumor
chr12	75454741	1731	KCNC2|3747	intronic	RNA-seq	Cancer Genome Atlas Research Network. 2017	28622513	Tumor
chr19	36212563	386	KMT2B|9757;ZBTB32|27033	intronic	RNA-seq	Cancer Genome Atlas Research Network. 2017	28622513	Tumor
chr15	74619350	1799	CCDC33|80125	intronic	RNA-seq	Cancer Genome Atlas Research Network. 2017	28622513	Tumor
chr17	17039280	1684	MPRIP|23164	intronic	RNA-seq	Cancer Genome Atlas Research Network. 2017	28622513	Tumor
chr4	113184622	832	AP1AR|55435	intronic	RNA-seq	Cancer Genome Atlas Research Network. 2017	28622513	Tumor
chr3	107429566	1092	BBX|56987	intronic	RNA-seq	Cancer Genome Atlas Research Network. 2017	28622513	Tumor
chr20	18419643	292	C20orf12|55184	intronic	RNA-seq	Cancer Genome Atlas Research Network. 2017	28622513	Tumor
chr10	123356431	1092	FGFR2|2263	intronic	RNA-seq	Cancer Genome Atlas Research Network. 2017	28622513	Tumor
chr12	75454741-75454	1707;1731	KCNC2|3747	intronic	RNA-seq	Cancer Genome Atlas Research Network. 2017	28622513	Tumor
chr4	113184622	832	AP1AR|55435	intronic	RNA-seq	Cancer Genome Atlas Research Network. 2017	28622513	Tumor
chr20	18419643	292	C20orf12|55184	intronic	RNA-seq	Cancer Genome Atlas Research Network. 2017	28622513	Tumor
chr2	30955553	1724	CAPN13|92291	intronic	RNA-seq	Cancer Genome Atlas Research Network. 2017	28622513	Tumor
chr12	75454741-75454	1707;1731	KCNC2|3747	intronic	RNA-seq	Cancer Genome Atlas Research Network. 2017	28622513	Tumor
chr1	19050821-19056	397;398	PAX7|5081	intronic	RNA-seq	Cancer Genome Atlas Research Network. 2017	28622513	Tumor
chr14	95115881	832	SERPINA13|388007	intronic	RNA-seq	Cancer Genome Atlas Research Network. 2017	28622513	Tumor
chr17	19469277	1769	SLC47A1|55244	intronic	RNA-seq	Cancer Genome Atlas Research Network. 2017	28622513	Tumor
chr20	45913863	1631	ZMYND8|23613	intronic	RNA-seq	Cancer Genome Atlas Research Network. 2017	28622513	Tumor
chrX	32675191-32675	813;834	DMD|1756	intronic	RNA-seq	Cancer Genome Atlas Research Network. 2017	28622513	Tumor
chr12	22475007	3537;1717	ST8SIA1|6489	intronic	RNA-seq	Cancer Genome Atlas Research Network. 2017	28622513	Tumor
chr22	36633639-36633	454;2066;2446	APOL2|23780	intronic	RNA-seq	Cancer Genome Atlas Research Network. 2017	28622513	Tumor
chr12	75454741	1731	KCNC2|3747	intronic	RNA-seq	Cancer Genome Atlas Research Network. 2017	28622513	Tumor
chr19	36255837	2135;312	C19orf55|148137	intronic	RNA-seq	Cancer Genome Atlas Research Network. 2017	28622513	Tumor
chr19	36212661	2072	KMT2B|9757;ZBTB32|27033	intronic	RNA-seq	Cancer Genome Atlas Research Network. 2017	28622513	Tumor
chr14	34240716	1120	NPAS3|64067	intronic	RNA-seq	Cancer Genome Atlas Research Network. 2017	28622513	Tumor
chr15	49190536	448	SHC4|399694	intronic	RNA-seq	Cancer Genome Atlas Research Network. 2017	28622513	Tumor
chr8	77633300	590	ZFHX4|79776	intronic	RNA-seq	Cancer Genome Atlas Research Network. 2017	28622513	Tumor
chr6	64948589	2798	EYS|346007	intronic	RNA-seq	Cancer Genome Atlas Research Network. 2017	28622513	Tumor
chr2	121585968-1216	460;2449;461;3	GLI2|2736	intronic	RNA-seq	Cancer Genome Atlas Research Network. 2017	28622513	Tumor
chr18	66429147	1807	CCDC102B|79839	intronic	RNA-seq	Cancer Genome Atlas Research Network. 2017	28622513	Tumor
chr4	173136734	1884;3698	GALNTL6|442117	intronic	RNA-seq	Cancer Genome Atlas Research Network. 2017	28622513	Tumor
chr8	67750916	1805	SGK3|23678	intronic	RNA-seq	Cancer Genome Atlas Research Network. 2017	28622513	Tumor
chr3	58969841	1408;1384	C3orf67|200844	intronic	RNA-seq	Cancer Genome Atlas Research Network. 2017	28622513	Tumor
chr11	904136	1459	CHID1|66005	intronic	RNA-seq	Cancer Genome Atlas Research Network. 2017	28622513	Tumor
chr3	19290210	1844	KCNH8|131096	intronic	RNA-seq	Cancer Genome Atlas Research Network. 2017	28622513	Tumor
chr12	124913291	1761	NCOR2|9612	intronic	RNA-seq	Cancer Genome Atlas Research Network. 2017	28622513	Tumor
chr11	74011666	832	P4HA3|283208	intronic	RNA-seq	Cancer Genome Atlas Research Network. 2017	28622513	Tumor
chr13	73439922	1121	PIBF1|10464	intronic	RNA-seq	Cancer Genome Atlas Research Network. 2017	28622513	Tumor
chr4	25315502	832	ZCCHC4|29063	intronic	RNA-seq	Cancer Genome Atlas Research Network. 2017	28622513	Tumor
chr19	2510793	342	GNG7|2788	intronic	RNA-seq	Cancer Genome Atlas Research Network. 2017	28622513	Tumor
chr19	36212707	485;461	KMT2B|9757;ZBTB32|27033	intronic	RNA-seq	Cancer Genome Atlas Research Network. 2017	28622513	Tumor
chr4	113184622	832	AP1AR|55435	intronic	RNA-seq	Cancer Genome Atlas Research Network. 2017	28622513	Tumor
chr4	95687099	1631	BMPR1B|658	intronic	RNA-seq	Cancer Genome Atlas Research Network. 2017	28622513	Tumor
chr2	30955553	1724	CAPN13|92291	intronic	RNA-seq	Cancer Genome Atlas Research Network. 2017	28622513	Tumor
chr19	30303438	2446;2470	CCNE1|898	intronic	RNA-seq	Cancer Genome Atlas Research Network. 2017	28622513	Tumor
chr12	75454741	1731	KCNC2|3747	intronic	RNA-seq	Cancer Genome Atlas Research Network. 2017	28622513	Tumor
chr18	2540834	1800	METTL4|64863	intronic	RNA-seq	Cancer Genome Atlas Research Network. 2017	28622513	Tumor
chr7	11180567	937	PHF14|9678	intronic	RNA-seq	Cancer Genome Atlas Research Network. 2017	28622513	Tumor
chr8	119363679	1631	SAMD12|401474	intronic	RNA-seq	Cancer Genome Atlas Research Network. 2017	28622513	Tumor
chr14	95115881	832	SERPINA13|388007	intronic	RNA-seq	Cancer Genome Atlas Research Network. 2017	28622513	Tumor
chr20	45913863	1631	ZMYND8|23613	intronic	RNA-seq	Cancer Genome Atlas Research Network. 2017	28622513	Tumor
chr20	18419643	292	C20orf12|55184	intronic	RNA-seq	Cancer Genome Atlas Research Network. 2017	28622513	Tumor
chr2	30955553	1724	CAPN13|92291	intronic	RNA-seq	Cancer Genome Atlas Research Network. 2017	28622513	Tumor
chr12	75454741	1731	KCNC2|3747	intronic	RNA-seq	Cancer Genome Atlas Research Network. 2017	28622513	Tumor
chr20	14066735	1093	MACROD2|140733	intronic	RNA-seq	Cancer Genome Atlas Research Network. 2017	28622513	Tumor
chr3	73658712	748	PDZRN3|23024	intronic	RNA-seq	Cancer Genome Atlas Research Network. 2017	28622513	Tumor
chr14	95115881	832	SERPINA13|388007	intronic	RNA-seq	Cancer Genome Atlas Research Network. 2017	28622513	Tumor
chr10	73193269	49	CDH23|64072	intronic	RNA-seq	Cancer Genome Atlas Research Network. 2017	28622513	Tumor
chr6	114179070	50	MARCKS|4082	intronic	RNA-seq	Cancer Genome Atlas Research Network. 2017	28622513	Tumor
chr7	143809744	787	OR2A2|442361	intronic	RNA-seq	Cancer Genome Atlas Research Network. 2017	28622513	Tumor
chr22	44545197	50	PARVB|29780	intronic	RNA-seq	Cancer Genome Atlas Research Network. 2017	28622513	Tumor
chr4	119247052	1137	PRSS12|8492	intronic	RNA-seq	Cancer Genome Atlas Research Network. 2017	28622513	Tumor
chr17	37007198	1725	RPL23|9349;SNORA21|619505	intronic	RNA-seq	Cancer Genome Atlas Research Network. 2017	28622513	Tumor
chrX	80464441	1658;1727	SH3BGRL|6451	intronic	RNA-seq	Cancer Genome Atlas Research Network. 2017	28622513	Tumor
chr16	84213911	1329	TAF1C|9013;LRRC50|123872	intronic	RNA-seq	Cancer Genome Atlas Research Network. 2017	28622513	Tumor
chr3	24420592	1658;1727	THRB|7068	intronic	RNA-seq	Cancer Genome Atlas Research Network. 2017	28622513	Tumor
chrX	32675191	813;834	DMD|1756	intronic	RNA-seq	Cancer Genome Atlas Research Network. 2017	28622513	Tumor
chr9	130085590	368	GARNL3|84253	intronic	RNA-seq	Cancer Genome Atlas Research Network. 2017	28622513	Tumor
chr12	112275572	50	MAPKAPK5|8550;C12orf47|51275	intronic	RNA-seq	Cancer Genome Atlas Research Network. 2017	28622513	Tumor
chr7	143809744	787	OR2A2|442361	intronic	RNA-seq	Cancer Genome Atlas Research Network. 2017	28622513	Tumor
chr9	137029133	2734;1801;1682	WDR5|11091;RNU6ATAC|100151684	intronic	RNA-seq	Cancer Genome Atlas Research Network. 2017	28622513	Tumor
chr2	111572783	573	ACOXL|55289	intronic	RNA-seq	Cancer Genome Atlas Research Network. 2017	28622513	Tumor
chr17	41477603	1434	ARL4D|379	intronic	RNA-seq	Cancer Genome Atlas Research Network. 2017	28622513	Tumor
chr20	18419643	292	C20orf12|55184	intronic	RNA-seq	Cancer Genome Atlas Research Network. 2017	28622513	Tumor
chr10	65180738	831;832	JMJD1C|221037	intronic	RNA-seq	Cancer Genome Atlas Research Network. 2017	28622513	Tumor
chr2	8906551	534	KIDINS220|57498	intronic	RNA-seq	Cancer Genome Atlas Research Network. 2017	28622513	Tumor
chr7	78922167	874;875	MAGI2|9863	intronic	RNA-seq	Cancer Genome Atlas Research Network. 2017	28622513	Tumor
chr8	54156479	1048	OPRK1|4986	intronic	RNA-seq	Cancer Genome Atlas Research Network. 2017	28622513	Tumor
chr3	60163954	1204	FHIT|2272	intronic	RNA-seq	Cancer Genome Atlas Research Network. 2017	28622513	Tumor
chr8	106473002	2696	ZFPM2|23414	intronic	RNA-seq	Cancer Genome Atlas Research Network. 2017	28622513	Tumor

Supplementary Table S8 Continued

Chromosome	Integration site in host genome	Integration site in virus genome	Gene (distance, bp)	Regions	Methods	Author	PMID	Sample
chr5	1295333	2754;1794;1796	TERT|7015	intronic	RNA-seq	Cancer Genome Atlas Research Network. 2017	28622513	Tumor
chr17	65896158	480	BPTF|2186	intronic	RNA-seq	Cancer Genome Atlas Research Network. 2017	28622513	Tumor
chr20	18419644	291	C20orf12|55184	intronic	RNA-seq	Cancer Genome Atlas Research Network. 2017	28622513	Tumor
chr5	40942739	2496	C7|730	intronic	RNA-seq	Cancer Genome Atlas Research Network. 2017	28622513	Tumor
chr8	4018111	1020	CSMD1|64478	intronic	RNA-seq	Cancer Genome Atlas Research Network. 2017	28622513	Tumor
chr10	113944411	2988	GPAM|57678	intronic	RNA-seq	Cancer Genome Atlas Research Network. 2017	28622513	Tumor
chr18	33043576	832	INO80C|125476	intronic	RNA-seq	Cancer Genome Atlas Research Network. 2017	28622513	Tumor
chr4	151261450	1116	LRBA|987	intronic	RNA-seq	Cancer Genome Atlas Research Network. 2017	28622513	Tumor
chr2	178127822	2655;2561	NFE2L2|4780	intronic	RNA-seq	Cancer Genome Atlas Research Network. 2017	28622513	Tumor
chr11	69466787	711;1807;1745;	ORAOV1|220064;CCND1|595	intronic	RNA-seq	Cancer Genome Atlas Research Network. 2017	28622513	Tumor
chr7	540077	3006	PDGFA|5154	intronic	RNA-seq	Cancer Genome Atlas Research Network. 2017	28622513	Tumor
chr3	73652513	2549	PDZRN3|23024	intronic	RNA-seq	Cancer Genome Atlas Research Network. 2017	28622513	Tumor
chr12	65006013	1499	RASSF3|283349	intronic	RNA-seq	Cancer Genome Atlas Research Network. 2017	28622513	Tumor
chr17	12890676	1770	RICH2|9912	intronic	RNA-seq	Cancer Genome Atlas Research Network. 2017	28622513	Tumor
chr10	75041785	1771	TTC18|118491	intronic	RNA-seq	Cancer Genome Atlas Research Network. 2017	28622513	Tumor
chr8	77633300	590	ZFHX4|79776	intronic	RNA-seq	Cancer Genome Atlas Research Network. 2017	28622513	Tumor
chr12	75454741	1731	KCNC2|3747	intronic	RNA-seq	Cancer Genome Atlas Research Network. 2017	28622513	Tumor
chr19	36213171	1800;3056	KMT2B|9757	intronic	RNA-seq	Cancer Genome Atlas Research Network. 2017	28622513	Tumor
chr13	96216538		CLDN10,	intronic	RNA-seq	Tang et al. 2013	24085110	Tumor
chr13	96229481		CLDN10,	intronic	RNA-seq	Tang et al. 2013	24085110	Tumor
chr13	96230150		CLDN10,	intronic	RNA-seq	Tang et al. 2013	24085110	Tumor
chr1	210977323		KCNH1,	intronic	RNA-seq	Tang et al. 2013	24085110	Tumor
chr1	211058710		KCNH1,	intronic	RNA-seq	Tang et al. 2013	24085110	Tumor
chr2	216248049		FN1,	intronic	RNA-seq	Tang et al. 2013	24085110	Tumor
chr2	216248745		FN1,	intronic	RNA-seq	Tang et al. 2013	24085110	Tumor
chr2	216251435		FN1,	intronic	RNA-seq	Tang et al. 2013	24085110	Tumor
chr2	216252176		FN1,	intronic	RNA-seq	Tang et al. 2013	24085110	Tumor
chr9	104362859		GRIN3A,	intronic	RNA-seq	Tang et al. 2013	24085110	Tumor
chr4	44210458		KCTD8,	intronic	RNA-seq	Tang et al. 2013	24085110	Tumor
chr2	216871084		PECR	intronic	RNA-seq	Tang et al. 2013	24085110	Tumor
chr2	216871084		MREG	intronic	RNA-seq	Tang et al. 2013	24085110	Tumor
chr11	120176798		POU2F3	intronic	RNA-seq	Tang et al. 2013	24085110	Tumor
chr5	64878406		PPWD1,	intronic	RNA-seq	Tang et al. 2013	24085110	Tumor
chr16	70196260		RP11-296I10.3.1,	intronic	RNA-seq	Tang et al. 2013	24085110	Tumor
chr19	36213906		KMT2B.1,	intronic	RNA-seq	Tang et al. 2013	24085110	Tumor
chr16	31413434		ITGAD,	intronic	RNA-seq	Tang et al. 2013	24085110	Tumor
chr16	31418854		ITGAD,	intronic	RNA-seq	Tang et al. 2013	24085110	Tumor
chr16	31414820		ITGAD	intronic	RNA-seq	Tang et al. 2013	24085110	Tumor
chr11	12711204		TEAD1,	intronic	RNA-seq	Tang et al. 2013	24085110	Tumor
chr19	36213761		KMT2B.1,	intronic	RNA-seq	Tang et al. 2013	24085110	Tumor
chr19	36212301		KMT2B.1,	intronic	RNA-seq	Tang et al. 2013	24085110	Tumor
chr1	36339292		EIF2C1,	intronic	RNA-seq	Tang et al. 2013	24085110	Tumor
chr17	31175299		MYO1D,	intronic	RNA-seq	Tang et al. 2013	24085110	Tumor
chr14	23429865		RP11-298I3.5.1,	intronic	RNA-seq	Tang et al. 2013	24085110	Tumor
chrX	38167314		RP5-972B16.2.1	intronic	RNA-seq	Tang et al. 2013	24085110	Tumor
chrX	38167314		RPGR		RNA-seq	Tang et al. 2013	24085110	Tumor
chrX	38167314		SNORA31,		RNA-seq	Tang et al. 2013	24085110	Tumor
chr12	83256165		TMTC2,	intronic	RNA-seq	Tang et al. 2013	24085110	Tumor
chr2	121585486		GLI2,	intronic	RNA-seq	Tang et al. 2013	24085110	Tumor
chr2	121589220		GLI2,	intronic	RNA-seq	Tang et al. 2013	24085110	Tumor
chr2	121592792		GLI2,	intronic	RNA-seq	Tang et al. 2013	24085110	Tumor
chr2	121648973		GLI2,	intronic	RNA-seq	Tang et al. 2013	24085110	Tumor
chr2	121684934		GLI2,	intronic	RNA-seq	Tang et al. 2013	24085110	Tumor
chr2	121708817		GLI2,	intronic	RNA-seq	Tang et al. 2013	24085110	Tumor
chr2	211421474		CPS1,	intronic	RNA-seq	Tang et al. 2013	24085110	Tumor
chr2	216248758		FN1,	intronic	RNA-seq	Tang et al. 2013	24085110	Tumor
chr2	216249582		FN1,	intronic	RNA-seq	Tang et al. 2013	24085110	Tumor
chr3	3747245		SUMF1,	intronic	RNA-seq	Tang et al. 2013	24085110	Tumor
chr11	123521882		SCN3B,	intronic	RNA-seq	Tang et al. 2013	24085110	Tumor
chr12	124912988		NCOR2,	intronic	RNA-seq	Tang et al. 2013	24085110	Tumor
chr1	176694014		PAPPA2,	intronic	RNA-seq	Tang et al. 2013	24085110	Tumor
chr12	71213384		PTPRR,	intronic	RNA-seq	Tang et al. 2013	24085110	Tumor
chr14	70245899		SLC10A1,	intronic	RNA-seq	Tang et al. 2013	24085110	Tumor
chr14	70252814		SLC10A1,	intronic	RNA-seq	Tang et al. 2013	24085110	Tumor
chr18		1594	PARD6G		Alu-PCR	Pollicino et al. 2013	22989571	Tumor

Supplementary Table S9. HBV co-target genes in pancreatic cancer and hepatocellular carcinoma

Gene	Chr. location	PDAC		HCC		Gene function
		Integration No.	Insertion site (No.)	Integration No.	Insertion site (No.)	
KMT2B	19q	1	Intron 3 (1)	230	Intron 3 (100), Intron 5 (9), Exon 3 (78), Exon 4 (2), Exon 5 (14), Exon 6 (12), Unknown (15).	Histone methyltransferase
EYS	6q	1	Intron 5 (1)	5	Intron 15 (2), Intron 12 (1), Intron 1 (1), Intron 6 (1).	Integrity of photoreceptor cells
BBS7	4q	1	Intron 1 (1)	5	Intron 12 (2), Intron 13 (2), Intron 15 (1).	Specific membrane proteins to the primary cilia
AGBL4	4q	1	Intron 4(1)	5	Intron 1 (2), Intron 3 (1), Intron 5 (2)	Antiviral activity of CGAS
CDH13	16q	1	Intron 2 (1)	3	Intron 3 (1), Intron 8 (1), Intron 10 (1).	Calcium-dependent cell adhesion proteins
COL15A1	9q	1	Intron 38 (1)	2	Intron 24 (1), Intergenic (1).	Stabilization of microvessels and muscle cells
LRP1B	2q	1	Intron 37 (1)	2	Intron 51 (1)	Protein homodimerization activity and kynureninase activity
LRRFIP1	2q	1	Intron 5 (1)	2	Promoter (2)	Receptor-mediated endocytosis
PTPRG	3q	1	Intron 2 (1)	3	Intron 14 (2), Intron 2 (1).	Tyrosine phosphatase regulator
SLC25A21	15q	1	Intron 1 (1)	2	Intron 7 (1), Intron 1 (1)	Function unknown
SUDS3	12q	1	UTR3 (1)	2	Promoter (1), Intergenic (1).	Apoptosis

Supplementary Table S9 Continued

Gene	Chr. location	PDAC		HCC		Gene function
		Integration No.	Insertion site (No.)	Integration No.	Insertion site (No.)	
DTD1	20p	1	Intron 4 (1)	1	Intron 4 (1)	DNA replication
CELSR1	22q	1	Intron 9 (1)	1	Intron 5 (1)	Cell signaling during nervous system formation
FARS2	6p	1	Intron 6 (1)	1	Intron 6 (1)	Mitochondrial translation
PRDM6	5q	1	Intron 3 (1)	1	Intron 7 (1)	Transcriptional repressor of smooth muscle gene expression
PTPRT	20q	1	Intron 6 (1)	1	Intron 1 (1)	Signal transduction and cellular adhesion in the CNS
RIMBP2	12q	1	Intron 2 (1)	1	Exon 9 (1)	Synaptic transmission
TBC1D2B	15q	1	Intron 6 (1)	1	Intron 12 (1)	GTPase-activating protein
TRIO	5p	1	Intron 1 (1)	1	Intron 1 (1)	Cell migration and growth
XPO6	16p	1	Intron 12 (1)	1	Intron 7 (1)	Nuclear export of actin and profilin-actin complexes
SLC22A11	11q	1	Intron 3 (1)	1	Intron 1 (1)	Mediating the transport of conjugated steroids and organic anions
LOC100507412	chrUn_gl000220	12	Downstream (2), Exon 3 (1), Intergenic (9)	5	Intron 5 (2), Intron 2 (2), Intron 6 (1).	lncRNA function(unknown)
DTNA	18q	1	Intron 17 (1)	2	Intron 2 (2)	Formation and stability of synapses and the clustering of nicotinic acetylcholine receptors
DIRC3	2q	1	Intergenic (1)	1	Exon 1 (1)	lncRNA function (hereditary renal carcinoma and breast cancer receptors)
FOXO1	13q	1	Intron 1 (1)	2	Intron 1 (1) Intergenic (1)	Transcription factor targeted by insulin signaling

Supplementary Table S9 Continued

Gene	Chr. location	PDAC		HCC		Gene function
		Integration No.	Insertion site (No.)	Integration No.	Insertion site (No.)	
XPR1	1q	1	Intron 1 (1)	2	Intron 2 (2)	Phosphate homeostasis
CCDC178	18q	1	Intron 14 (1)	2	Intron 21 (2)	Function unknown
NRG3	10q	1	Intron 2 (1)	1	Intron 1 (1), Intron 2 (1), Intron 3 (1).	Ligand for the ERBB4 tyrosine kinase receptor
ATXN1	6p	1	Intron 3 (1)	1	Intron 7 (1)	Akt signaling
TRPM6	9q	1	Intron 1 (1)	1	Intergenic (1)	Magnesium homeostasis in gut and kidney
KCNJ16	17q	1	Exon (1)	1	Intergenic (1)	Potassium inward rectifying
MATN2	8q	1	Intron 6 (1)	1	Intron 2 (1)	Function unknown
SIRPG	20p	1	Intergenic (1)	1	Intron 4 (1)	Negative regulation of RTK coupled signaling
PLCB1	20p	1	Intron 32 (1)	1	Intron 2 (1)	Angiotensin activation of ERK
CPNE4	3q	1	Intron 2 (1)	2	Intron 7 (2)	Membrane trafficking, mitogenesis and development
SACM1L	3p	1	Intergenic (1)	1	Exon 20 (1)	Phosphoinositide phosphatase
HFM1	1p	1	Intergenic (1)	3	Intron 4 (2) Intron 13 (1)	An ATP-dependent DNA helicase
ITGAM	16p	1	Intergenic (1)	1	Exon 6 (1)	Apoptotic and integrin pathway
C11orf24	13q	1	Intergenic (1)	1	Exon 4 (1)	Chromosome 11 Open Reading Frame 24
NSUN7	4p	1	Intron 8 (1)	1	Intron 10 (1)	Methylation

The HBV targeted genes in HCC and PDAC are defined as those genes located within 10 kb of the integration site.
PDAC: pancreatic ductal adenocarcinoma; HCC: hepatocellular carcinoma; Chr.: Chromosome; No.: number.
